# The genetic landscape of metaplastic breast cancers and uterine carcinosarcomas

**DOI:** 10.1002/1878-0261.12813

**Published:** 2021-02-19

**Authors:** Lea A. Moukarzel, Lorenzo Ferrando, Arnaud Da Cruz Paula, David N. Brown, Felipe C. Geyer, Fresia Pareja, Salvatore Piscuoglio, Anastasios D. Papanastasiou, Nicola Fusco, Caterina Marchiò, Nadeem R. Abu‐Rustum, Rajmohan Murali, Edi Brogi, Hannah Y. Wen, Larry Norton, Robert A. Soslow, Anne Vincent‐Salomon, Jorge S. Reis‐Filho, Britta Weigelt

**Affiliations:** ^1^ Department of Surgery Memorial Sloan Kettering Cancer Center New York NY USA; ^2^ Department of Pathology Memorial Sloan Kettering Cancer Center New York NY USA; ^3^ Department of Internal Medicine University of Genoa Italy; ^4^ Visceral Surgery Research Laboratory, Clarunis Department of Biomedicine University of Basel Switzerland; ^5^ Department of Biomedical Sciences University of West Attica Athens Greece; ^6^ Division of Pathology Fondazione IRCCS Ca' Grande – Ospedale Maggiore Policlinico Milan Italy; ^7^ Department of Medical Sciences University of Turin Italy; ^8^ Department of Medicine Memorial Sloan Kettering Cancer Center New York NY USA; ^9^ Department of Pathology Institut Curie Paris France

**Keywords:** breast cancer, carcinosarcoma, homologous recombination DNA repair, metaplastic, uterine cancer, whole‐exome sequencing

## Abstract

Metaplastic breast carcinoma (MBC) and uterine carcinosarcoma (UCS) are rare aggressive cancers, characterized by an admixture of adenocarcinoma and areas displaying mesenchymal/sarcomatoid differentiation. We sought to define whether MBCs and UCSs harbor similar patterns of genetic alterations, and whether the different histologic components of MBCs and UCSs are clonally related. Whole‐exome sequencing (WES) data from MBCs (*n* = 35) and UCSs (*n* = 57, The Cancer Genome Atlas) were reanalyzed to define somatic genetic alterations, altered signaling pathways, mutational signatures, and genomic features of homologous recombination DNA repair deficiency (HRD). In addition, the carcinomatous and sarcomatous components of an additional cohort of MBCs (*n* = 11) and UCSs (*n* = 6) were microdissected separately and subjected to WES, and their clonal relatedness was assessed. MBCs and UCSs harbored recurrent genetic alterations affecting *TP53*, *PIK3CA*, and *PTEN*, similar patterns of gene copy number alterations, and an enrichment in alterations affecting the epithelial‐to‐mesenchymal transition (EMT)‐related Wnt and Notch signaling pathways. Differences were observed, however, including a significantly higher prevalence of *FAT3* and *FAT1* somatic mutations in MBCs compared to UCSs, and conversely, UCSs significantly more frequently harbored somatic mutations affecting *FBXW7* and *PPP2R1A* as well as *HER2* amplification than MBCs. Genomic features of HRD and biallelic alterations affecting *bona fide* HRD‐related genes were found to be more prevalent in MBCs than in UCSs. The distinct histologic components of MBCs and UCSs were clonally related in all cases, with the sarcoma component likely stemming from a minor subclone of the carcinoma component in the samples with interpretable chronology of clonal evolution. Despite the similar histologic features and pathways affected by genetic alterations, UCSs differ from MBCs on the basis of *FBXW7* and *PPP2R1A* mutations, *HER2* amplification, and lack of HRD, supporting the notion that these entities are more than mere phenocopies of the same tumor type in different anatomical sites.

## Graphical abstract

Metaplastic breast carcinoma (MBC) and uterine carcinosarcoma (UCS) are rare aggressive cancers, characterized by an admixture of adenocarcinoma and areas displaying mesenchymal/sarcomatoid differentiation. In this study, we investigate whether MBCs and UCSs harbor similar patterns of genetic alterations and mutational signatures using whole‐exome sequencing data. In addition, we examine whether the different histologic components of MBCs and UCSs are clonally related.

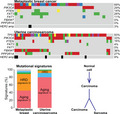

**Abbreviations**
CCF: cancer cell fraction
CI: clonality index
CNA: copy number alteration
EMT: epithelial‐to‐mesenchymal transition
HRD: homologous recombination DNA repair deficiency
LST: large‐scale state transition
MBC: metaplastic breast carcinoma
NtAI: numerical telomeric allelic imbalance
SNV: single nucleotide variant
TCGA: The Cancer Genome Atlas
TNBC: triple‐negative breast cancer
UCS: uterine carcinosarcoma
WES: whole‐exome sequencing

## 1 Introduction

Metaplastic breast carcinoma (MBC) is a rare histologic form of breast cancer, usually of triple‐negative phenotype, accounting for 0.2–5% of breast cancers [1]. These tumors are characterized by differentiation of malignant epithelium into squamous and/or mesenchymal elements, such as spindle, chondroid, osseous, or rhabdoid cells [1]. We and others have previously shown that the histologic heterogeneity of MBCs is paralleled by heterogeneity at the genomic and transcriptomic levels [2, 3, 4, 5, 6], and provided evidence that the histologically distinct components of each MBC are almost uniformly clonally related [7, 8, 9, 10, 11]. Given their clonal nature, it has been postulated that in MBCs with mesenchymal elements, epithelial‐to‐mesenchymal transition (EMT) may play a role in the development of the metaplastic component [12, 13, 14]. Consistent with this notion, these tumors are often transcriptomically classified as claudin‐low or mesenchymal‐like subtypes [4, 5], and display overexpression of cellular migration‐ and extracellular matrix formation‐related genes [4, 5, 15]. At the genetic level, MBCs are characterized by recurrent mutations affecting *TP53* and genes related to the PI3K/AKT/mTOR, MAPK, Wnt, and Notch signaling pathways [2, 4, 8, 16, 17].

Uterine carcinosarcomas (UCSs), previously called malignant mixed Müllerian tumors (MMMTs), are rare aggressive tumors composed of high‐grade malignant carcinomatous and sarcomatous/mesenchymal elements, accounting for < 5% of uterine cancers and 15% of uterine cancer‐associated deaths in the United States [18, 19, 20]. The mesenchymal component of UCSs may consist of histologic elements native to the uterus (homologous) or of heterologous components, such as rhabdomyosarcoma or chondrosarcoma [20]. A number of studies have been conducted to identify pathways altered in UCSs and potential therapeutic targets. Akin to MBCs, UCSs have been found to harbor recurrent mutations affecting *TP53* and the PI3K/AKT/mTOR signaling pathway [19] as well as mutations in chromatin remodeling and core histone genes [21, 22, 23].

Given their histologic similarities, we posited that MBCs and UCSs would constitute counterparts of the same tumor type in different anatomical sites, that these tumors would be underpinned by similar genetic alterations, and that the distinct histologic components of individual MBCs and UCSs would be clonally related. Hence, in this study, we have reanalyzed data previously published by our team [2] and The Cancer Genome Atlas (TCGA) [19] to compare the repertoire of genetic alterations and pathways altered in MBCs and UCSs. We have also sequenced independently microdissected carcinomatous and sarcomatous components of 11 MBCs and 6 UCSs to infer bioinformatically the chronology of the development of the histologically distinct components within MBCs and UCSs.

## 2 Materials and methods

### 2.1 Cases

**Fig. 1:**
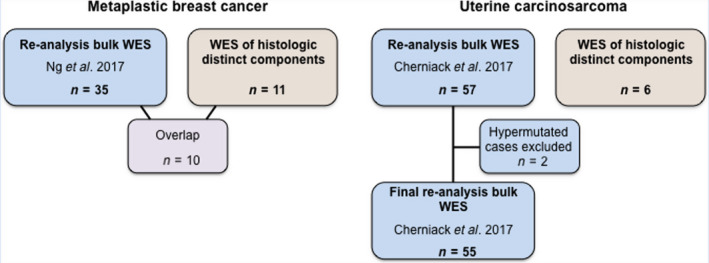
Schematic representation of the metaplastic breast carcinomas and uterine carcinosarcomas included in this study. WES data of metaplastic breast cancers (MBCs; *n* = 35) from Ng *et al*. [2] and uterine carcinosarcomas (UCSs; *n* = 55, *n* = 2 hypermutated cases were excluded) from Cherniack *et al*./The Cancer Genome Atlas [19] were reanalyzed. In addition, the epithelial and mesenchymal components of 11 MBCs, of which 10 overlapped with those from Ng *et al*. [2], and of 6 UCSs were separately microdissected and subjected to WES.

This study was approved by the Institutional Review Boards (IRBs) of the authors' institutions, and patient consents were obtained as required by the protocols approved by the IRBs. This study is in compliance with the Declaration of Helsinki. Formalin‐fixed paraffin‐embedded (FFPE) tissue blocks of 11 MBCs (including 10 cases reported in Ng *et al*. [2]) and 6 fresh‐frozen (FF) UCSs were retrieved (Table S1). All 11 microdissected MBCs displayed a triple‐negative [i.e., estrogen receptor (ER), progesterone receptor (PR), and HER2‐negative] phenotype (Table S1). Samples were deidentified prior to analysis. All cases were reviewed by pathologists with expertise and experience in breast pathology (FCG, AV‐S, and JSR‐F) and gynecologic pathology (RM and RAS). The histologically distinct components of these MBCs and UCSs (i.e., epithelial and mesenchymal) were independently microdissected and subjected to whole‐exome sequencing (WES; Fig. 1).

In addition, we retrieved the whole‐exome raw sequence data (BAM files) from 35 MBCs included in our previous study by Ng *et al*. [2] as well as from 57 UCSs reported by Cherniack *et al*. [19] (TCGA) from the NCI GDC portal (https://portal.gdc.cancer.gov/). Clinicopathologic characteristics of the MBCs and UCSs were retrieved from our previous study [2], Cherniack *et al*. [19], and from patient medical records (Table S1).

### 2.2 DNA extraction

For the microdissection of the distinct epithelial and mesenchymal components of a given MBC or UCS, we performed high‐molecular‐weight cytokeratin immunohistochemistry of the first and last sections as a guide. The distinct epithelial and mesenchymal components of MBCs (*n* = 11) were microdissected from 8‐µm‐thick representative FFPE sections with a needle under a stereomicroscope, as previously described [5, 24, 25]. For UCSs (*n* = 6), the distinct epithelial and mesenchymal components were microdissected from 8‐µm‐thick representative FF sections either with a needle under a stereomicroscope [5, 24, 25] or using laser microdissection, as previously described by our group [26], on a Leica LMD 6500 System (Leica Microsystems Inc., Buffalo Grove, IL, USA). All microdissections were performed by pathologists (FCG, ADP, NF, CM, and JSR‐F). Genomic DNA was extracted from tumor and matched normal tissues using the DNeasy Blood and Tissue Kit (Qiagen, Germantown, MD, USA), according to manufacturer's instructions, and quantified using the Qubit Fluorometer (Invitrogen, Thermo Fisher Scientific, Waltham, MA, USA).

### 2.3 WES and targeted amplicon resequencing

DNA samples from the histologically distinct components of each of the 11 MBCs and six UCSs and their respective normal samples were subjected to WES at MSKCC's Integrated Genomics Operation (IGO) following validated protocols [27, 28]. Sequencing data of the separately microdissected components, as well as of the 35 bulk MBCs [2] and 57 UCSs (TCGA) [19], were analyzed as previously described [27, 28] (Data S1). Mutation hotspots were determined according to Chang *et al*. [29]. A somatic mutation was defined as pathogenic if it affected a mutational hotspot or was deleterious/loss‐of‐function (in the case of tumor suppressor genes). For the 17 multicomponent cases, in addition to the identification of somatic mutations in individual samples, any mutation detected in one of the histological component of a given case was subsequently queried in the other matched component using samtools mpileup (v1.2) [30]. Allele‐specific copy number alterations (CNAs), tumor purity, and ploidy were obtained from the WES data using facets [31]. The cancer cell fractions (CCFs) of putative somatic mutations identified were computed using absolute (v1.0.6) [32], as previously described [27, 28]. The fraction of the genome altered was computed from the CNAs obtained from facets (Data S1).

Selected putative somatic mutations identified in MBCs (*n* = 11) and UCSs (*n* = 5) by WES were subjected to orthogonal validation using a custom‐designed AmpliSeq panel, as previously described [33]; 98% (444/451) of the nonsynonymous mutations subjected to orthogonal resequencing were validated in the MBCs and 97% (60/62) of the private nonsynonymous mutations were validated in the UCSs (Table S2). Somatic mutations that were not validated were excluded from the downstream analyses.

### 2.4 Microsatellite instability

The presence of microsatellite instability (MSI) was defined in the paired tumor‐normal WES data using MSIsensor [34], as previously described [35], and samples with MSIsensor scores ≥ 3.5 were considered MSI high [34].

### 2.5 Homologous recombination DNA repair defects and mutational signatures

Homologous recombination DNA repair deficiency (HRD) was assessed by defining large‐scale state transition (LST) scores, numerical telomeric allelic imbalance (NtAI) scores, mutational signature 3, microhomology‐mediated deletions, and the length of small deletions. LSTs and NtAIs were computed from the results of facets using the WES data according to Popova *et al*. [36] and Birkbak *et al*. [37], with a cutoff of ≥ 15 for LST high, as previously described [38]. Mutational signatures were inferred from both synonymous and nonsynonymous somatic mutations in MBCs and UCSs with at least 20 single nucleotide variants (SNVs) using DeconstructSigs [39] with default parameters, based on the set of mutational signatures represented in version 2 as part of COSMIC release v89 (https://cancer.sanger.ac.uk/cosmic/signatures_v2), as previously described [35]. All but two MBCs (META55 and META61) had ≥ 20 SNVs for mutational signature analysis, and the dominant mutational signature of a given case is reported. Given that tumors with deficient HR have been shown to have an enrichment for small deletions ≥ 5bp and microhomology‐mediated deletions [40, 41], the length of small deletions and the presence of deletions with microhomology were assessed in the samples analyzed, as described [35, 40, 41]. Finally, raw methylation data (Illumina Infinium MethylationEPIC BeadChips) from all 57 UCSs from TCGA [19] were retrieved from the TCGA NCI GDC portal (https://portal.gdc.cancer.gov/) and analyzed as previously described [42], and the methylation status of the promoter regions of *RAD51C* and *BRCA1* in the UCSs was assessed.

### 2.6 Clonal relatedness

To infer the clonal relatedness between the histologically distinct components of each MBC (*n* = 11) and UCS (*n* = 6), we defined the ‘clonality index’ (CI) as the probability of two lesions sharing mutations not expected to have co‐occurred by chance based on a previously validated method [43] (Data S1).

### 2.7 Clonal decomposition

To define the clonal architecture and composition of the histologically distinct and independently microdissected components of the MBCs (*n* = 11) and UCSs (*n* = 6) included in this study, the somatic mutations identified were analyzed using pyclone [44]. Somatic mutations were excluded from the clonal decomposition analysis if they affected loci with (a) low total depth (< 20×) in the matched normal, (b) low total depth (< 50×) in any tumor component of a given case, (c) where the tumor variant allele fractions (VAFs) of both components of a given case were lower than five times the normal VAF, and (d) where the total tumor depth exceeds 1500× in any component of a given case. This usually corresponds to regions of the human genome with low‐sequence complexity (e.g., telomeres, centromeres, pseudogenes), which may lead to misaligned sequence reads and false‐positive mutations. Estimates of tumor purity and absolute copy numbers were obtained from the VAF of somatic mutations and Log_2_ ratios derived from WES data using absolute [32]. These were used as input for pyclone [44] with the beta‐binomial model, run through 20000 MCMC iterations with a burn‐in of 10000 iterations, total copy number prior, and a beta‐binomial precision value of 500, as previously described [43]. The resulting CCFs were used to categorize mutations as truncal or branch. Truncal mutations were defined as those displaying a modal clonal frequency/CCF in the clonally related mesenchymal and carcinoma components of a given case, whereas branch mutations were defined as all nontruncal mutations.

### 2.8 Pathway analyses

A DAVID pathway analysis was conducted based on genes affected by nonsynonymous somatic mutations, amplifications, or homozygous deletions [45]. Pathways found to be significantly enriched (*P* < 0.01) in MBCs or UCSs and previously curated and reported in Sanchez‐Vega *et al*. [46] were selected. The list of genes and interactions constituting the canonical versions of these pathways was retrieved from pathwaymapper [47].

### 2.9 Comparative and statistical analyses

For comparisons of MBCs and UCSs, hypermutated cases defined as those with ≥ 1000 somatic mutations were excluded [28]. Two of the 57 UCSs from TCGA but none of the MBCs were hypermutated. Comparisons of continuous and categorical variables were performed using the Mann–Whitney *U* and Fisher's exact tests, respectively, and adjusted for multiple testing using the false discovery rate (FDR), whenever appropriate. An FDR < 0.05 was considered statistically significant. All tests were two‐sided. Unless otherwise stated, all statistical analyses were performed using r/bioconductor (https://www.bioconductor.org/).

## 3 Results

### 3.1 Repertoire of somatic genetic alterations in MBCs and UCSs

**Fig. 2:**
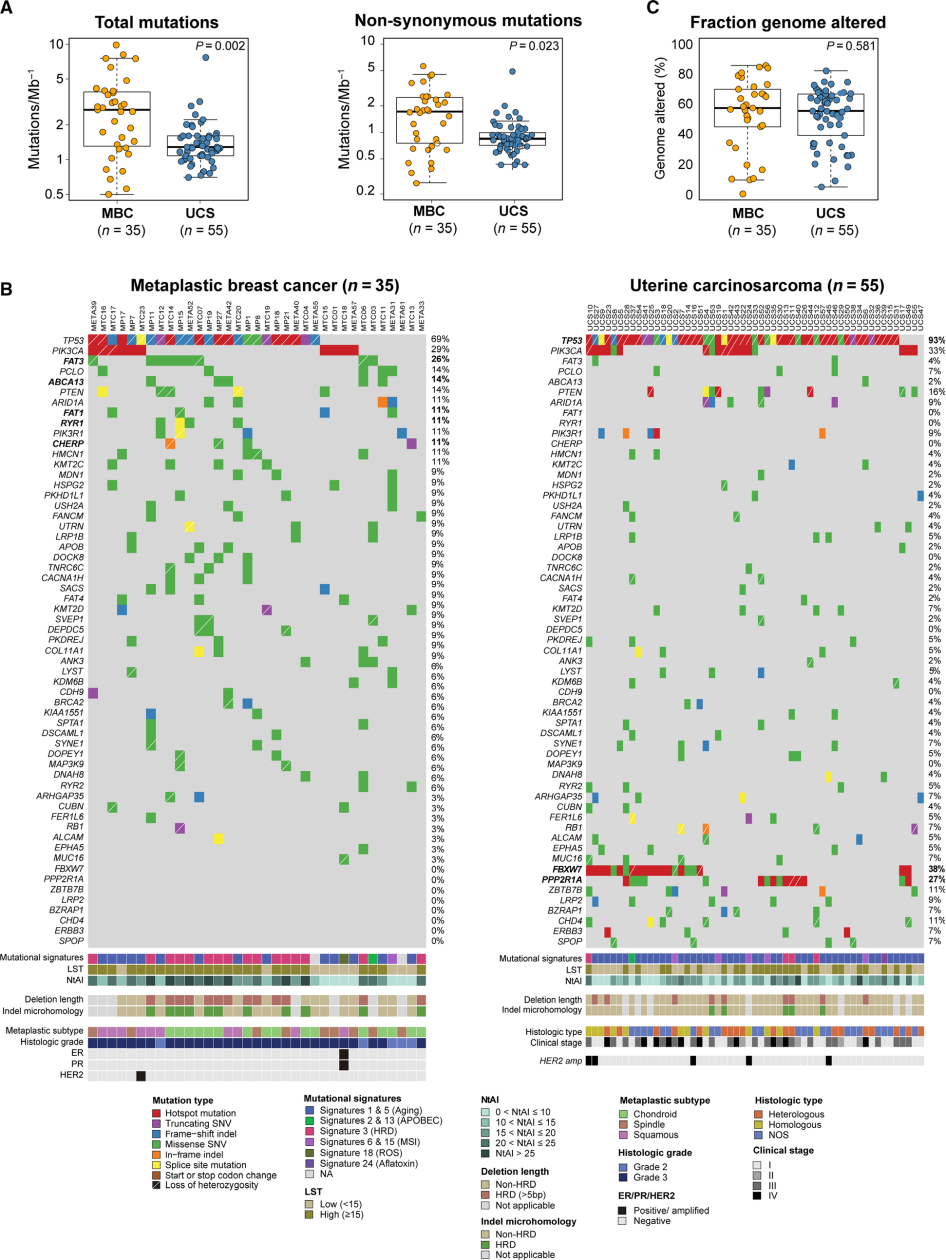
Repertoire of somatic mutations in metaplastic breast carcinomas and uterine carcinosarcomas. (A) Total number of somatic mutations and nonsynonymous somatic mutations per Mb in metaplastic breast cancers (MBCs) reanalyzed from Ng *et al*. [2] and uterine carcinosarcomas (UCSs) reanalyzed from The Cancer Genome Atlas (TCGA) [19]. Mann–Whitney *U*‐test employed. (B) Nonsynonymous somatic mutations identified in WES data from MBCs reanalyzed from Ng *et al*. [2], left, and UCSs reanalyzed from TCGA [19], right. Cases are shown in columns and genes in rows. Mutation types, mutational signatures, LSTs, NtAIs, small deletion length, small insertion and deletion (indel) microhomology, and clinicopathologic factors are color‐coded according to the legend. . (C) Fraction of the genome altered in MBCs reanalyzed from Ng *et al*. [2] and UCSs reanalyzed from TCGA [19]. Mann–Whitney *U*‐test employed.

Reanalysis of WES data from 35 MBCs reported in our previous study by Ng *et al*. [2] and of 55 nonhypermutated UCSs retrieved from TCGA [19] (Fig. 1) revealed that MBCs had a higher median number of somatic mutations and nonsynonymous somatic mutations than UCSs (MBCs: median of 2.9 (range 0.5–10) and 1.6 (range 0.25–5.4) of total and nonsynonymous somatic mutations per Mb, respectively; UCSs: median of 1.3 (range 0.7–7.9) and 0.8 (range 0.4–4.7) of total and nonsynonymous somatic mutations per Mb, respectively; *P* < 0.05; Mann–Whitney *U*‐test; Fig. 2A). Despite the higher mutational burden in MBCs, the repertoire of somatic mutations in MBCs and UCSs shared many similarities (Fig. 2B), including alterations affecting *PIK3CA* (29%, 10/35 MBCs vs 33%, 18/55 UCSs, *P* = 0.816, Fisher's exact test) and *PTEN* (14%, 5/35 MBCs vs 16%, 9/55 UCSs, *P* = 1, Fisher's exact test). Important differences were observed, however; MBCs more frequently displayed somatic mutations in *FAT3* (26% vs 4%, *P* = 0.0028, Fisher's exact test), *ABCA13* (14% vs 2%, *P* = 0.031, Fisher's exact test), *FAT1, CHERP*, and *RYR1* (each, 11% vs 0%, *P* = 0.02; Fisher's exact test) than UCSs. Conversely, UCSs significantly more frequently harbored somatic mutations affecting *FBXW7* (38% vs 0%, *P* < 0.01; Fisher's exact test) and *PPP2R1A* (27% vs 0%, *P* < 0.01; Fisher's exact test) than MBCs (Fig. 2B). In addition, although *TP53* mutations were common in both tumor types, they were significantly more frequently found in UCSs than in MBCs (93% vs 69%; *P* = 0.004, Fisher's exact test).

MBCs and UCSs displayed high levels of copy number alterations (CNAs), with similar fractions of the genome altered (MBC, median 58%, range 0–81%; UCS, median 55%, range 5–82%, *P* = 0.581; Mann–Whitney *U*‐test; Fig. 2C, Fig. S1a). Recurrent CNAs included gains of 1q (43%, 15/35 MBCs; 28%, 16/55 UCSs), 3q (23%, 8/35 MBCs; 18%, 10/55 UCSs), and 8q (46%, 16/35 MBCs; 47%, 27/55 UCSs), and losses of 3p (20%, 7/35 MBCs; 19%, 11/55 UCSs) and 8p (34%, 12/35 MBCs; 37%, 21/55 UCSs), which did not differ between the MBCs and UCSs (all *P* > 0.05). In addition, we observed recurrent 8q12.1 and 8q24.1‐22 amplifications in both MBCs and UCSs, encompassing the *CHCHD7* (9%, 3/35 MBCs; 9%, 5/55 UCSs), *PLAG1* (9%, 3/35 MBCs; 9%, 5/55 UCSs), *MYC* (26%, 9/35 MBCs; 11%, 6/55 UCSs), and *NDRG1* (23%, 8/35 MBCs; 9%, 5/55 UCSs) oncogenes (Fig. S1a). In contrast, however, while MBCs are generally of triple‐negative phenotype and only 1/35 (3%) of the MBCs studied here were HER2‐positive, 5/55 (9%) UCSs were found to display a *HER2* amplification (*P* = 0.40, Fishers' exact test; Fig. 2B).

### 3.2 MBCs and UCSs harbor recurrent somatic genetic alterations affecting the p53, PI3K, Wnt, and Notch pathways

**Fig. 3:**
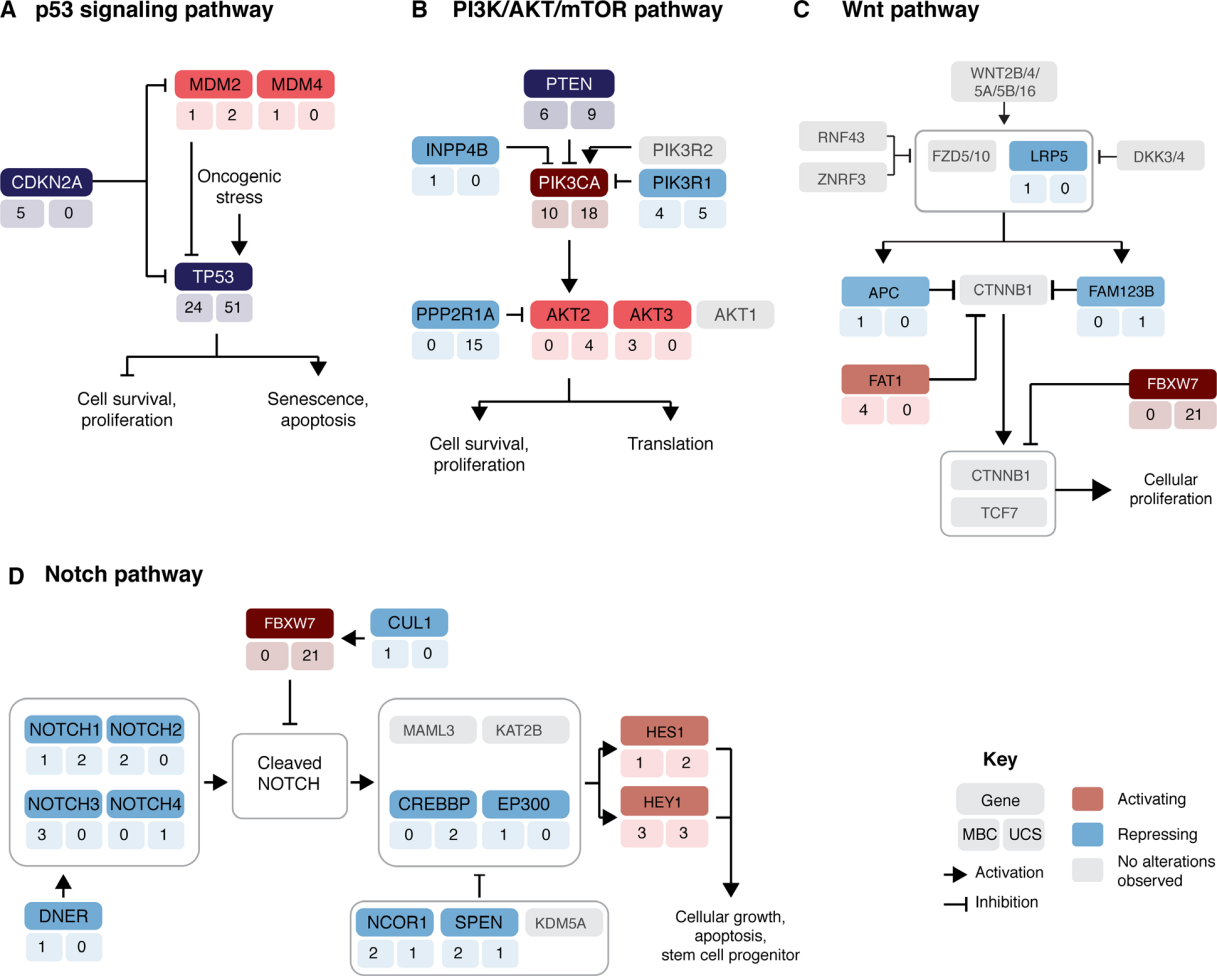
Metaplastic breast carcinomas and uterine carcinosarcomas harbor genetic alterations affecting similar signaling pathways. Frequency of activating (red) or loss‐of‐function (blue) somatic genetic alterations affecting genes in the canonical (A) p53, (B) PI3K/AKT/mTOR, (C) Wnt, and (D) Notch signaling pathways. The number of metaplastic breast cancers (MBCs, left) and uterine carcinosarcomas (UCSs, right) harboring a given somatic mutations or gene copy number alterations is depicted under the gene name. Pathways found to be significantly enriched (*P* < 0.01) in MCBs or UCSs and previously reported in Sanchez‐Vega *et al*. [46] are shown.

Given the similarities in the repertoire of somatic genetic alterations detected in MBCs and UCSs, we sought to compare the signaling pathways targeted by somatic genetic alterations in these tumors. A pathway analysis based on the somatic mutations and CNAs revealed an enrichment of genetic alterations targeting the canonical p53, PI3K/AKT/mTOR, Wnt, and Notch pathways, as defined by Sanchez‐Vega *et al*. [46], in both MBCs and UCSs (Fig. 3, Table S3); however, the target genes in these pathways varied according to the cancer type. The most frequently affected genes of the p53 signaling pathway were *TP53* and *MDM2/4* in both MBCs and UCSs (Fig. 3A); however, *CDKN2A* alterations were solely found in MBCs (14% MBCs vs 0% UCSs, *P* = 0.007, Fisher's exact test). Although *PIK3CA* (29% MBCs and 33% UCSs), *PTEN* (17% MBCs and 16% UCSs), and *PIK3R1* (11% MBCs and 9% UCSs; all *P* > 0.05, Fisher's exact test) were PI3K signaling pathway components frequently affected by somatic mutations or CNAs in both MBCs and UCSs, other genes of the PI3K pathway such as *PPP2R1A* (27% UCSs vs 0% MBCs, *P* < 0.001; Fisher's exact test) and *AKT2* (7% UCSs vs 0% MBCs, *P* = 0.154; Fisher's exact test) were affected exclusively in UCSs, whereas genetic alterations affecting *AKT3* (9% MBCs vs 0% UCSs, *P* = 0.055; Fisher's exact test) and *INPP4B* (3% MBCs vs 0% UCSs, *P* = 0.389; Fisher's exact test) were uniquely found in MBCs (Fig. 3B, Fig. S1b).

Several lines of evidence suggest that epithelial‐to‐mesenchymal transition (EMT)‐related processes might underpin MBCs and UCSs [2, 4, 12, 13, 14, 19, 48, 49]. Our analyses revealed that 43% (15/35) of MBCs and 53% (29/55) of UCSs harbored somatic genetic alterations affecting at least one gene of the canonical Wnt signaling pathway, of which 73% (11/15) of MBCs and 79% (23/29) of UCSs had at least one pathogenic mutation, amplification, or homozygous deletion (Fig. 3C). The Wnt pathway genes most frequently affected by somatic mutations or CNAs among MBCs and UCSs were *ARID1A* (11% MBCs vs 9% UCSs, *P* = 0.731, Fisher's exact test) and *MYC* (26% MBCs vs 11% UCSs, *P* = 0.08; Fisher's exact test). Importantly, however, genetic alterations affecting *FBXW7* were found exclusively in UCSs (38% UCSs vs 0% MBCs, *P* < 0.01; Fisher's exact test), whereas *FAT1* (11% MBCs vs 0% UCSs, *P* = 0.02; Fisher's exact test) and *APC* (3% MBCs vs 0% UCSs, *P* = 0.389; Fisher's exact test) were altered in MBCs but not in UCSs (Fig. 3C, Fig. S1b). Likewise, 43% (15/35) of MBCs and 56% (31/55) of UCSs harbored somatic genetic alterations affecting at least one gene of the canonical Notch signaling pathway, of which 73% (11/15) of MBCs and 81% (25/31) of UCSs were affected by at least one pathogenic mutation, amplification, or homozygous deletion (Fig. 3D). The genes of the Notch signaling pathway most frequently affected by genetic alterations in MBCs and UCSs were *HEY1* (9% MBCs vs 5% UCSs), *NOTCH1* (3% MBCs vs 4% UCSs), and *HES1* (3% MBCs vs 4% UCSs; all *P* > 0.05, Fisher's exact test). Mutations affecting *NOTCH2* (6%)*, NOTCH3* (9%)*, DNER* (3%)*, EP300* (3%)*, and CUL1* (3%) were found in MBCs, whereas *NOTCH4* (2%) alterations were only detected in UCSs (Fig. 3D, Fig. S1b).

### 3.3 MBCs more frequently display genomic features consistent with HRD than UCSs

**Fig. 4:**
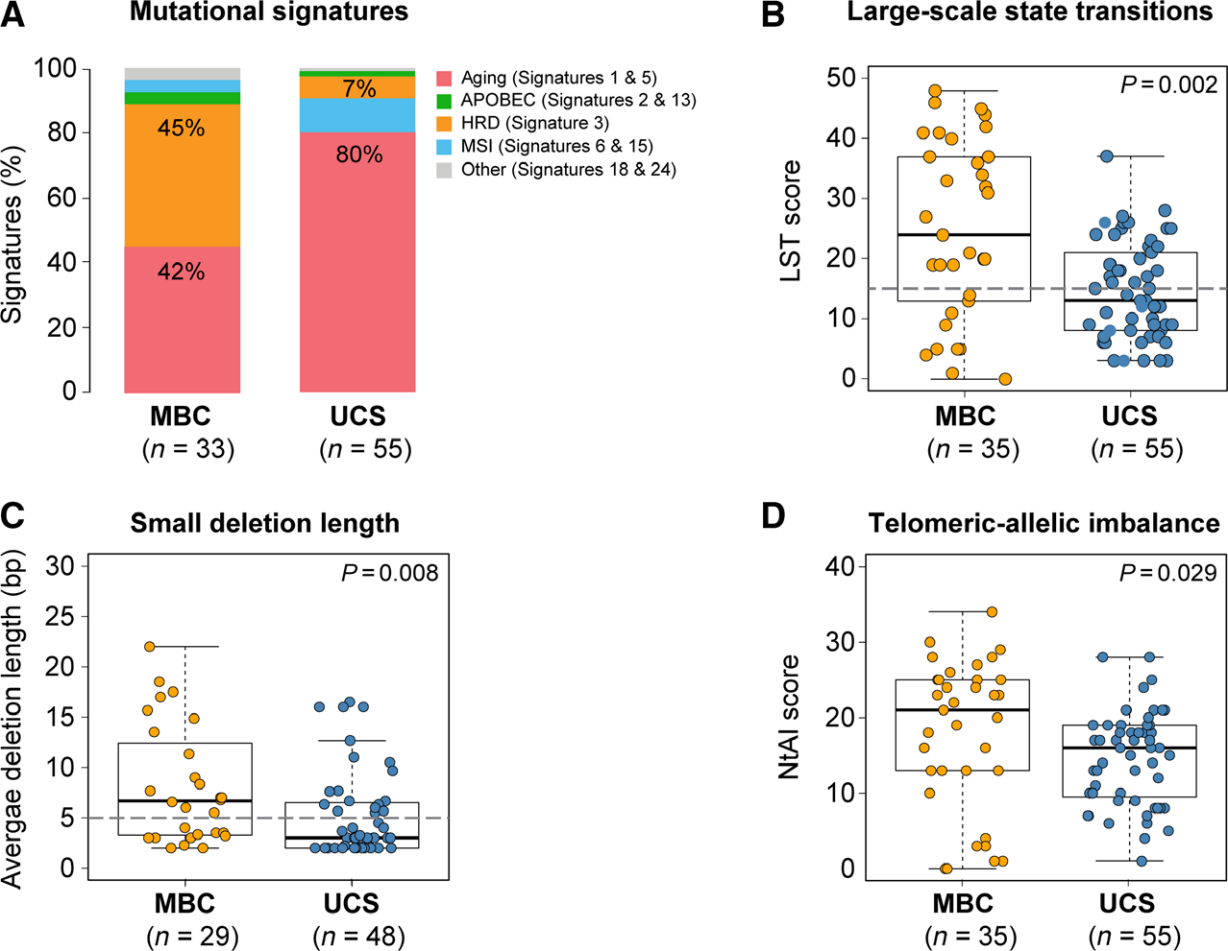
Genomic features of homologous recombination repair deficiency in metaplastic breast carcinomas and uterine carcinosarcomas. (A) Mutational signatures in metaplastic breast cancers (MBCs) from Ng *et al*. [2] and nonhypermutated uterine carcinosarcomas (UCSs) from TCGA [19] identified using DeconstructSigs [39]. Mutational signatures are color‐coded according to the legend and were only performed for samples ≥ 20 SNVs. (B) LST scores in MBCs from Ng *et al*. [2] and nonhypermutated UCSs from TCGA [19]. The gray line depicts the cutoff for LST high (≥ 15) [36]. (C) Small deletion length in MBCs from Ng *et al*. [2] and nonhypermutated UCSs from TCGA [19] according to Alexandrov *et al*. [40], which in HRD‐defective tumors has been found to be ≥ 5 nucleotides (gray line). (D) NtAI score in MBCs from Ng *et al*. [2] and nonhypermutated UCSs from TCGA [19] according to Morganella *et al*. [41]. Mann–Whitney *U*‐test was performed for comparisons in (B), (C), and (D). MBC, metaplastic breast cancer.

MBCs have been reported to display frequent homologous recombination DNA repair (HRD) defects [2]. Hence, we sought to investigate whether the UCSs studied here would display similar genomic features suggestive of HRD or other biological processes that would result in genetic instability. Our analyses revealed the presence of a dominant mutational signature 3 associated with HRD in 45% (15/33) of MBCs. In contrast, only 7% of UCSs (4/55) displayed a dominant signature 3 (*P* < 0.001; Fisher's exact test). Instead, the majority (80%; 44/55) of UCSs displayed a dominant signature 1 or signature 5 [35], which have been ascribed to aging [50], compared to 42% (14/33) of MBCs (*P* < 0.001; Fisher's exact test; Figs 2A and 4A). Consistent with these findings, the median LST scores (24 vs 13, *P* < 0.002, Mann–Whitney *U*‐test), NtAI scores (21 vs 16, *P* = 0.029, Mann–Whitney *U*‐test), and deletion length of ≥ 5 bp (*P* = 0.008, Mann–Whitney *U*‐test) in MBCs were statistically significantly higher than those in UCSs (Fig. 4B–D). All MBCs (15/33) with a dominant mutational signature 3 displayed other genomic features suggestive of HRD, such as high LST scores (> 15), NtAI scores > 16, average small deletion length ≥ 5 bp, and deletions with microhomology in 100% (15/15), 80% (12/15), and 73% (11/15) of cases, respectively (Fig. 2B). The four UCSs displaying a dominant mutation signature 3 also had high LST scores, with two of them being associated with long deletions as well as deletions with microhomology (Fig. 2B).

We next sought to identify the underlying genetic basis for HRD in the 45% of MBCs and 7% of UCSs displaying genomic features suggestive of HRD. Our analyses revealed that of the 15 MBCs with genomic features suggestive of HRD, 9 demonstrated biallelic inactivation of HRD‐related genes [38, 51]. Eight MBCs harbored germline mutations associated either with loss‐of‐heterozygosity or a second somatic mutation (*BRCA1*, *n* = 6; *BRCA2*, *n* = 1; and *RBBP8*, *n* = 1), and one MBC displayed a *BRCA2* homozygous deletion (Table S4). None of the MBCs with a dominant aging‐related mutational signature were found to harbor biallelic genetic alterations in HRD‐related genes. Of the four UCSs displaying genomic features of HRD, UCS11 and UCS12 were found to harbor homozygous deletions in *USP11* and *FANCA*, respectively (Table S4). In addition, analysis of the promoters of *BRCA1* and *RAD51C*, whose methylation has been shown to be associated with HRD in breast and ovarian cancer [51], revealed that UCS10 and UCS12 displayed *RAD51C* promoter hypermethylation.

### 3.4 The epithelial and mesenchymal components of MBCs and UCSs are clonally related

There are multiple lines of evidence to support the contention that the different histologic components of MBCs and UCSs are clonally related [3, 7, 8, 9, 10, 19, 52], but there is also evidence to suggest that in a small subset of MBCs, the histologically distinct components may be genetically independent and/or collision tumors (e.g., case 5 from Geyer *et al*. [9]).

**Fig. 5:**
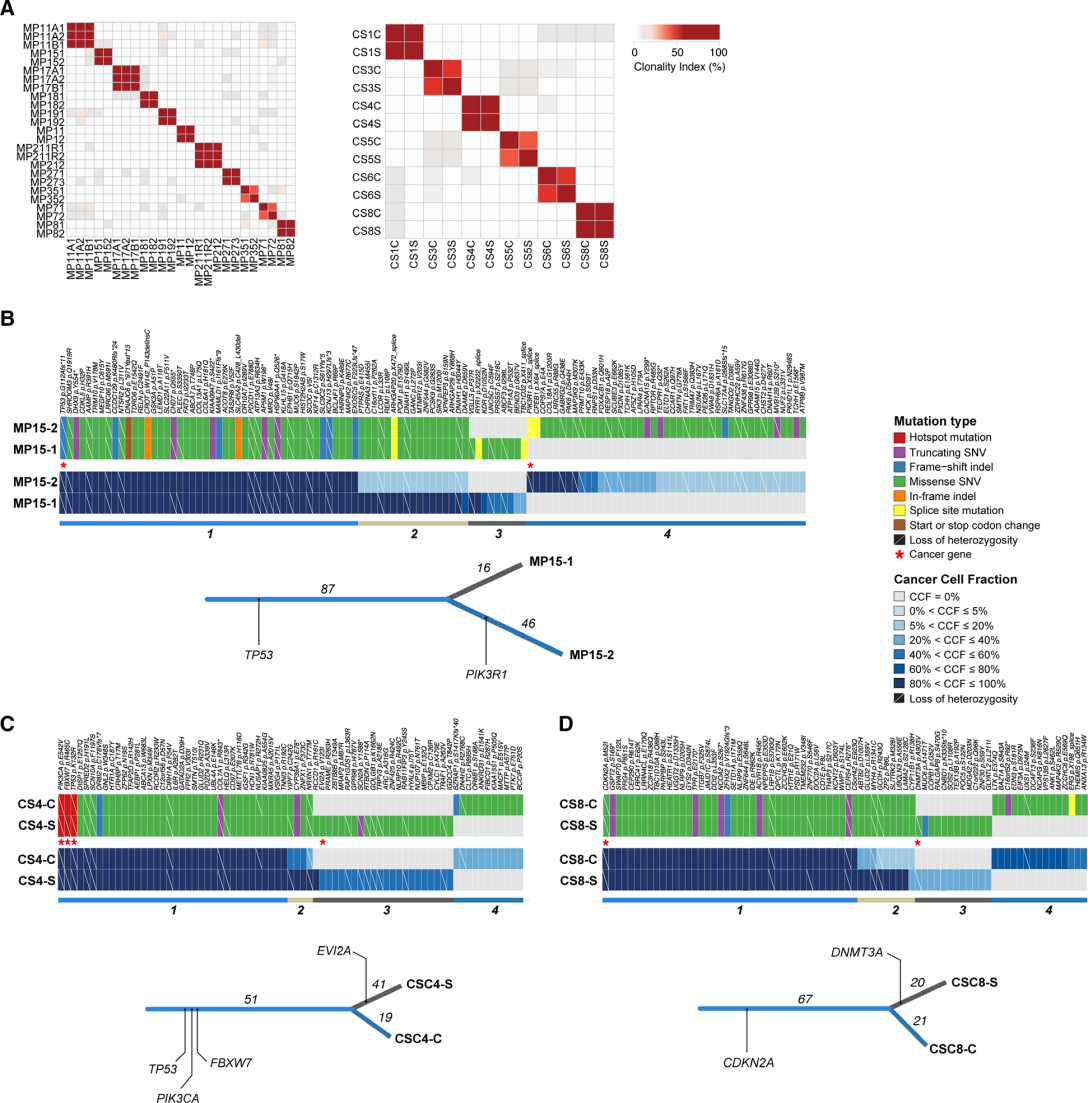
Clonal relatedness and decomposition of the epithelial and mesenchymal components of metaplastic breast carcinomas and uterine carcinosarcomas. (A) Clonality index of the epithelial and mesenchymal components of metaplastic breast cancers (MBCs, left) and of the epithelial and mesenchymal components of uterine carcinosarcomas (UCSs, right) subjected to WES based on somatic mutations. The histologic components are clonally related in all cases. (B) Cancer cell fractions (CCFs) of the somatic mutations identified in the epithelial and mesenchymal histologic components by WES in the metaplastic breast carcinoma MP15, (C) in the UCS CS4, and (D) UCS CS8. Mutations are grouped by their CCF as inferred by pyclone [44]. Cluster memberships are depicted below the heatmaps, and the corresponding phylogenetic trees are displayed. The length of the trunk and branches represent the number of shared and private somatic mutations identified in the different histologic components.

To define whether the histologically distinct components of MBCs and UCSs would be clonally related, we applied a previously validated approach to define clonal relatedness between tumor samples [43] (Data S1) based on the somatic mutations present in the histologically distinct microdissected components from 11 MBCs and 6 UCSs. Of these 11 MBCs, 10 were subjected to bulk WES previously described in Ng *et al*. [2] and reanalyzed in this study (Fig. 1; Fig. S2). This analysis revealed that the epithelial and mesenchymal components of all MBCs and UCSs studied here were clonally related, formally corroborating the notion that in the vast majority of MBCs and UCSs, the histologically distinct components originate from the same clone (Fig. 5A; Table S5).

Given that in all MBCs and UCSs analyzed here, the histologically distinct components were clonally related and that, as a group, MBCs and UCSs were found to harbor genetic alterations affecting genes related to EMT, we posited that the mesenchymal component would stem from the epithelial component. Clonal decomposition using pyclone [44] revealed that in MBC15, a minor subclone of the ductal component became dominant in the mesenchymal (chondroid) component (Fig. 5B), consistent with the notion that in this case, the chondroid component originated from a minor subclone of the epithelial (i.e., ductal) component. Likewise, clonal decomposition of the six UCSs revealed evidence of clonal selection in CS4 and CS8 (Fig. 5C,D), in which the sarcoma component appeared to have stemmed from a minor subclone of the carcinoma. In the remaining MBCs and UCSs, the chronology of the development of the different components could not be inferred based on the sequencing results, given that no clonal enrichment in the carcinomatous or sarcomatous component was observed on the basis of mutations affecting protein‐coding genes and/or CNAs (Fig. S3a,b). No differences in the mutational signatures were observed between the two distinct histologic components in any given case (Table S5).

Among the truncal mutations across all 11 MBCs and 6 UCSs analyzed, *TP53* somatic mutations were found to be clonal and truncal in all but 3 UCSs. In addition, UCS6 harbored a *TP53* homozygous deletion (data not shown). These findings are supportive of the role of *TP53* mutations as early drivers in the development of these cancers. No gene was found to be recurrently exclusively mutated in either the epithelial or mesenchymal components of the MBCs and UCSs analyzed (Fig. 5, Fig. S3a,b), suggesting that alterations other than somatic mutations or gene CNAs (e.g., epigenetic changes, somatic genetic alterations affecting regulatory elements) may account for the histologic diversity characteristic of these cancers.

## 4 Discussion

Here, we demonstrate that MBCs and UCSs harbor recurrent genetic alterations affecting *TP53*, *PIK3CA*, and *PTEN*, consistent with prior studies [2, 5, 14, 17, 19], and that these tumors display overall similar patterns of gene CNAs. Despite differences in the repertoire of somatic mutations observed between MBCs and UCSs, our analyses revealed an enrichment of genetic alterations affecting genes of the Wnt and Notch signaling pathways, which play pivotal roles in EMT [53, 54]. In fact, several of the genetic alterations that were distinct between MBCs and UCSs affected the same pathway (e.g., such as *FAT1* and *FBXW7*, which were restricted to MBCs and UCSs, respectively, but signal through the Wnt pathway). In addition, we have also provided evidence that the histologically distinct components of MBC and UCS analyzed here were clonally related and that the mesenchymal components likely stemmed from the epithelial component in cases where the chronology of the development of the components could be inferred. Given that these tumors display recurrent alterations affecting Wnt, Notch, and other EMT‐related pathways, one could posit that EMT may play a role in the development of the histologic diversity that characterizes MBCs and UCSs.

Despite the molecular similarities, in particular the high frequency of *TP53* mutations and high levels of chromosomal instability found between MBCs and UCSs, important differences were observed. In the datasets analyzed, MBC patients (median age 53, range 34–82) were significantly younger at diagnosis than UCS patients (median age 68, range 51–90; *P* < 0.0001, Mann–Whitney *U*‐test, Fig. S2c), which is consistent with the reported ages of diagnosis of MBCs and UCSs [11, 55]. Although MBCs were diagnosed at younger ages, we observed that 42% of cases had a dominant aging‐related mutational signature, akin to common‐type triple‐negative breast cancers [56, 57], and genomic features of HRD were present in 45% of the MBCs analyzed; conversely, only 7% of the UCSs were found to have HRD features, and 80% of the UCSs harbored dominant mutational signatures related to aging (i.e., mutational signatures 1 and 5). We further demonstrate that, in agreement with previous observations by our group [38] and others [51], biallelic alterations affecting canonical homologous recombination DNA repair‐related genes were the likely cause of HRD in the majority of MBCs and UCSs analyzed here. Furthermore, we identified *RAD51C* promoter hypermethylation in UCSs displaying HRD features (Table S4). Intriguingly, despite the evidence of HRD in MBCs, and unlike other forms of triple‐negative breast cancers, they appear to be resistant to conventional genotoxic chemotherapy [58]. As opposed to common forms of triple‐negative disease, where the rates of pathologic complete response (pCR) following neoadjuvant chemotherapy are > 40% [59], the reported pCR rates for MBCs range from 0% to 17% [11, 58, 60, 61]. Our findings may provide a molecular basis for this clinical conundrum, given that despite the high prevalence of HRD in MBCs, these tumors were found to display alterations in EMT‐related pathways, which may result in an intrinsic resistance to conventional genotoxic therapies [62]. Further studies are warranted to define the type of DNA repair defects playing a role in UCSs, given that based on WES analysis, the vast majority of UCSs displayed a dominant aging mutational signature, followed by HRD (i.e., signature 3 in 7% cases) and microsatellite instability (i.e., two cases excluded from the comparisons due to their hypermutated phenotype).

While genomic features of HRD were rare in UCSs, we did identify a subset harboring *HER2* amplification. The addition of trastuzumab to chemotherapy is now recommended for the treatment of HER2‐positive advanced or recurrent uterine serous carcinomas [63]. Given the clinically aggressive behavior of UCSs and limited treatment options [64], exploring targeting HER2 in this subset of HER2‐amplified UCSs may be warranted [65]. Likewise, therapeutic strategies based on synthetic lethality to target tumors with *FBXW7* mutations have emerged [66, 67]; given the relatively high frequency of *FBXW7* mutations in UCSs (30%), further studies testing this potential treatment strategy might be entertained.

Consistent with previous work by Joneja *et al*. [68], we found *TP53* (69% this study, 56% Joneja *et al*.) and *PIK3CA* (29% this study, 23% Joneja *et al*.) to be the most commonly mutated genes in MBCs. Previous work by Hayes *et al*. [69] reported on the presence of identical frameshift *WISP3* somatic mutations in five out of 27 MBCs; however, none of the MBCs studied here had mutations affecting *WISP3* even after inspection and manual curation of the sequencing results. Furthermore, Krings and Chen [70] demonstrated that 25% of MBCs harbored *TERT* promoter mutations. *TERT* promoter mutations could not be investigated in this series as they are not included in the genomic footprint of the targeted WES panel utilized in this study. Further studies are required to confirm the frequency of *TERT* promoter mutations in this rare type of breast cancer.

Our clonal decomposition analysis revealed that the epithelial and mesenchymal components of MBCs and UCSs are clonally related and display marked genetic heterogeneity. We observed that the mesenchymal component of at least a subset of MBCs and UCSs stemmed from a subclone of the epithelial component, following a clonal selection evolutionary pattern. Nonetheless, in the majority of MCBs and UCSs analyzed, the epithelial and mesenchymal components appear to have diverged somewhat early in the evolution of the tumors. It is possible that the different histologic components of these tumors evolved from a common histologic precursor and acquired either genetic alterations affecting genes other than protein‐coding genes or epigenetic alterations that resulted in the acquisition of mesenchymal features.

Our study has important limitations. WES was the basis for the genomic characterization of the MBCs and UCSs and their microdissected histologically distinct components analyzed here. Although orthogonal high‐depth validation of the mutations employed for clonal decomposition was performed, WES data do not allow for the characterization of mutations affecting noncoding regulatory elements and structural variants. In addition, given the greater accuracy of whole‐genome sequencing (WGS) for the detection of HRD and its causes, the potential for the identification of defects in other DNA repair mechanisms, and the greater data density for clonal decomposition analyses, further WGS studies of larger series of these tumors are warranted. Finally, we cannot rule out FFPE‐based sequencing artifacts in the subset of FFPE MBCs analyzed; however, no biallelic genetic alterations in HRD‐related genes were identified in MBCs with a dominant aging‐related mutational signature, and no enrichment in aging‐related mutational signatures in FFPE vs fresh‐frozen MBCs was found.

## 5 Conclusions

Here, we demonstrate that MBCs and UCSs harbor recurrent somatic genetic alterations affecting *TP53* and genes related to the PI3K, Wnt, and Notch pathways. The histologically distinct components present in MBCs and UCSs were found to be clonally related, and, at least in a subset of cases, the mesenchymal component likely originated from the epithelial component. Despite some differences in terms of specific genetic alterations between MBCs and UCSs, the pathways targeted by these alterations are remarkably similar in these tumors. Genomic features of HRD were found to be significantly more prevalent in MBCs than in UCSs, whereas known therapeutic targets, such as *HER2* gene amplification and *FBXW7* mutations, were found to be significantly more frequent in UCSs than MBCs. Hence, despite the histologic similarities and similar pathways being affected by somatic genetic alterations, MBCs and UCSs are more than mere phenocopies of the same tumors in different anatomical sites.

## Conflict of interest

JS Reis‐Filho is a consultant of Paige.AI, REPARE Therapeutics and Goldman Sachs, a member of the Board of Directors of Grupo Oncoclinicas, a member of the scientific advisory board of Volition RX, Paige.AI, and REPARE Therapeutics, and an ad hoc member of the advisory boards of Roche Tissue Diagnostics, Novartis, Roche, Genentech, and InVicro, all outside the submitted work. NR Abu‐Rustum reports institutional grants from Stryker/Novadaq, Olympus, and GRAIL, outside the submitted work. The remaining authors have no conflicts of interest to declare.

## Author contributions

BW and JSR‐F conceived the study. FCG, FP, RM, EB, HYW, RAS, AV‐S, and JSR‐F performed pathology review. FCG, FP, SP, ADP, NF, CM, and JSR‐F performed microdissection and DNA extraction. LF, ADCP, and DNB performed bioinformatics analysis. LM, LF, ADCP, DNB, FCG, NRA‐R, RM, EB, HYW, LN, RAS, AV‐S, JSR‐F, and BW interpreted data. LM, LF, ADCP, DNB, FP, JSR‐F, and BW wrote the first draft of the manuscript, which was read and approved by all authors.

## Supporting information


**Data S1.** Supplementary Methods.
**Fig. S1.** Copy number alterations and somatic mutations affecting selected signaling pathways in metaplastic breast cancers and uterine carcinosarcomas.
**Fig. S2.** Representative micrographs of the histologic components of the metaplastic breast cancers subjected to bulk whole‐exome sequencing as well as to whole‐exome sequencing of the distinct microdissected components.
**Fig. S3.** Clonal decomposition of the epithelial and mesenchymal components of the metaplastic breast carcinomas and uterine carcinosarcomas.
**Table S1.** Clinico‐pathologic information of the 35 metaplastic breast cancers reanalyzed from Ng et al (Clin Cancer Res 2017), 57 uterine carcinosarcomas from Cherniack et al (TCGA, Cancer Cell 2017), 11 metaplastic breast cancers with separately analyzed histologic components (this study), and 6 uterine carcinosarcomas with separately analyzed histologic components (this study).
**Table S2.** Non‐synonymous somatic mutations identified in the epithelial and mesenchymal components of 11 metaplastic breast cancers and 6 uterine carcinosarcomas subjected to whole‐exome sequencing.
**Table S3.** DAVID pathway analysis in 35 metaplastic breast cancers from Ng et al. (Clin Cancer Res 2017) and 55 non‐hypermutated uterine carcinosarcomas from Cherniack et al (TCGA, 2017).
**Table S4.** Genetic alterations affecting homologous recombination genes in metaplastic breast carcinomas and uterine carcinosarcomas.
**Table S5.** Number of shared and unique mutations, mutational signatures and clonal relatedness index of the histologically distinct components of 11 metaplastic breast cancers and 6 uterine carcinosarcomas subjected to whole‐exome sequencing.
