# Supplementary material for: The genetic landscape of metaplastic breast cancers and uterine carcinosarcomas
Source: Mol Oncol. 2021 Feb 19;15(4):1024–39. doi: 10.1002/1878-0261.12813 (PMC8024717; doi:10.1002/1878-0261.12813)
Supplement: Supplementary file 1 — Data S1. Supplementary Methods. Fig. S1. Copy number alterations and somatic mutations affecting selected signaling pathways in metaplastic breast cancers and uterine carcinosarcomas. Fig. S2. Representative micrographs of the histologic components of the metaplastic breast cancers subjected to bulk whole‐exome sequencing as well as to whole‐exome sequencing of the distinct microdissected components. Fig. S3. Clonal decomposition of the epithelial and mesenchymal components of the metaplastic breast carcinomas and uterine carcinosarcomas. Table S1. Clinico‐pathologic information of the 35 metaplastic breast cancers reanalyzed from Ng et al (Clin Cancer Res 2017), 57 uterine carcinosarcomas from Cherniack et al (TCGA, Cancer Cell 2017), 11 metaplastic breast cancers with separately analyzed histologic components (this study), and 6 uterine carcinosarcomas with separately analyzed histologic components (this study). Table S2. Non‐synonymous somatic mutations identified in the epithelial and mesenchymal components of 11 metaplastic breast cancers and 6 uterine carcinosarcomas subjected to whole‐exome sequencing. Table S3. DAVID pathway analysis in 35 metaplastic breast cancers from Ng et al. (Clin Cancer Res 2017) and 55 non‐hypermutated uterine carcinosarcomas from Cherniack et al (TCGA, 2017). Table S4. Genetic alterations affecting homologous recombination genes in metaplastic breast carcinomas and uterine carcinosarcomas. Table S5. Number of shared and unique mutations, mutational signatures and clonal relatedness index of the histologically distinct components of 11 metaplastic breast cancers and 6 uterine carcinosarcomas subjected to whole‐exome sequencing. [file MOL2-15-1024-s001.pdf]

SUPPLEMENTARY MATERIALS

The Genetic Landscape of Metaplastic Breast Cancers and Uterine Carcinosarcomas

Moukarzel et al.

Supplementary Methods

Supplementary Figures S1-S3

Supplementary Tables S1-S5

SUPPLEMENTARY METHODS

Whole-exome sequencing analysis

DNA samples from each the histologically distinct components of each of the 11 metaplastic breast cancers (MBCs) and six uterine carcinosarcomas (UCSs) and their respective normal samples were subjected to whole-exome sequencing (WES) at MSKCC's Integrated Genomics Operation (IGO) following validated protocols (1,2). Sequencing data of the separately microdissected components, as well as of the 35 bulk MBCs (Ng et al, 2017)(3) and 57 UCSs (The Cancer Genome Atlas, TCGA)(4) were analyzed as previously described (1,2). In brief, somatic single nucleotide variants (SNVs) were identified using MuTect (v.1.1.4) (5), and small insertions and deletions (indels) were detected using a combination of callers (i.e. VarScan2 (v2.3.6)(6), Strelka (v3.1.1)(7), Lancet (8), Platypus (9) and Scalpel (10)) as previously described (1). SNVs and indels outside the intersection of the two WES capture designs (i.e. Ng et al (3) and TCGA (4)) were filtered out, as were SNVs and indels for which the variant allele fraction (VAF) in the tumor sample was <5 times the VAF of the paired normal tissue, SNVs and indels found at >5% global minor allele frequency of dbSNP (build 146) or found at >1% global allele frequency in ExAC or gnomAD (11), as previously described (1,12).

Fraction of the genome altered

Allele specific copy number aberrations (CNAs), tumor purity and ploidy were obtained from the WES data using FACETS (13). The fraction of the genome altered was computed from the CNAs obtained from FACETS. Segments with an absolute copy number above or below the integer ploidy or were affected by copy neutral loss of heterozygosity were considered altered. The footprint of genome covered by these segments was reported as a function of the total footprint of genome covered by the WES capture design, as described (12).

Clonal relatedness

To infer the clonal relatedness between the histologically distinct components of each MBC and UCS, we defined the "clonality index" (CI) as the probability of two lesions sharing mutations not expected to have co-occurred by chance based on a previously validated method (14,15). Briefly, all somatic mutations were included and at least one non-synonymous mutation was required to be shared in two samples being compared. Given that this analysis is potentially confounded by the presence of highly recurrent somatic mutations, the CI provides an adjustment for the presence and frequency of a given somatic mutation, including synonymous and non-synonymous, in the triple-negative breast cancer (TNBC) dataset from TCGA (n=123)(16), given that all MBCs included in this study were TNBCs, the 35 MBCs from Ng et al. (3), and the 57 UCSs retrieved from TCGA (4).

Adopting previous approach (14,15), we defined $CI = -log_{10} \prod_{m=1}^{M} P(X)_m$. Given the repertoire of mutations of two samples, the probability of observing a given mutation in both samples is defined by the binomial probability $P(X) = C_n^k p^k (1-p)^{n-k}$, *n*=2, *k*=2, where *p* is the frequency of a given mutation in the combined 158 TNBCs and MBCs for the analysis of the 11 multicomponent MBCs, or the 55 non-hypermutated UCSs cases from TCGA for the analysis of the 6 multicomponent UCSs, and *n* is the number of shared mutations between a pair of lesions or the average number of mutations found in the two samples in the target regions divided by the size of the target regions. Thus, the probability of observing a given set of *M* identical mutations in the two samples is given by $\prod_{m=1}^{M} P(X)_m$. To objectively define a cut-off for clonal relatedness among the MBC cases, we used the mutational data from the 123 TNBCs from TCGA (16) and the 35 MBCs from Ng et al. (3). As the positive control (i.e. clonally related), we randomly selected 40%, 60% and 80% of the set of mutations from the 158 unrelated cases in duplicate to simulate heterogeneity between biologically related samples. As the negative control (i.e. unrelated), we randomly selected an equivalent number of pairs (i.e. 3x158=474) of unrelated cases from TCGA. To define the optimum cut-offs, the R package 'ROCR' was used to maximize accuracy. To avoid over-fitting of data, the above procedures were repeated 100 times to define the median and 95% confidence interval of the optimum cut-off. The median optimum cut-off was 18.78 (95% confidence interval 18.71-18.86), the median accuracy was 82.3% (95% confidence interval 80.1%-83.8%), the median sensitivity was 78.9% (95% confidence interval 75.9%-81.9%), and the median specificity was 85.7% (95% confidence interval 85.3%-86.0%).

A similar analysis was performed to define a cut-off for clonal relatedness among the UCS cases, using the mutational data from the 55 non-hypermutated UCSs from TCGA (4). The median optimum cut-off was 15.3 (95% confidence interval 15.0-15.6), the median accuracy was 93.1% (95% confidence interval 91.9%-94.4%), the median sensitivity was 89.1% (95% confidence interval 86.8%-91.4%), and the median specificity was 97.1% (95% confidence interval 96.7%-97.6%).

Supplementary References

1. Pareja F, Brandes AH, Basili T, Selenica P, Geyer FC, Fan D, et al. Loss-of-function mutations in ATP6AP1 and ATP6AP2 in granular cell tumors. Nat Commun. 2018;9:3533.
2. Geyer FC, Li A, Papanastasiou AD, Smith A, Selenica P, Burke KA, et al. Recurrent hotspot mutations in HRAS Q61 and PI3K-AKT pathway genes as drivers of breast adenomyoepitheliomas. Nat Commun. 2018;9:1816.
3. Ng CKY, Piscuoglio S, Geyer FC, Burke KA, Pareja F, Eberle CA, et al. The Landscape of Somatic Genetic Alterations in Metaplastic Breast Carcinomas. Clin Cancer Res. 2017;23:3859-70.

4. Cherniack AD, Shen H, Walter V, Stewart C, Murray BA, Bowlby R, et al. Integrated Molecular Characterization of Uterine Carcinosarcoma. Cancer Cell. 2017;31:411-23.
5. Cibulskis K, Lawrence MS, Carter SL, Sivachenko A, Jaffe D, Sougnez C, et al. Sensitive detection of somatic point mutations in impure and heterogeneous cancer samples. Nat Biotechnol. 2013;31:213-9.
6. Koboldt DC, Zhang Q, Larson DE, Shen D, McLellan MD, Lin L, et al. VarScan 2: somatic mutation and copy number alteration discovery in cancer by exome sequencing. Genome Res. 2012;22:568-76.
7. Saunders CT, Wong WS, Swamy S, Becq J, Murray LJ, Cheetham RK. Strelka: accurate somatic small-variant calling from sequenced tumor-normal sample pairs. Bioinformatics. 2012;28:1811-7.
8. Narzisi G, Corvelo A, Arora K, Bergmann EA, Shah M, Musunuri R, et al. Genome-wide somatic variant calling using localized colored de Bruijn graphs. Commun Biol. 2018;1:20.
9. Rimmer A, Phan H, Mathieson I, Iqbal Z, Twigg SRF, Consortium WGS, et al. Integrating mapping-, assembly- and haplotype-based approaches for calling variants in clinical sequencing applications. Nat Genet. 2014;46:912-8.
10. Fang H, Bergmann EA, Arora K, Vacic V, Zody MC, Iossifov I, et al. Indel variant analysis of short-read sequencing data with Scalpel. Nat Protoc. 2016;11:2529-48.
11. Lek M, Karczewski KJ, Minikel EV, Samocha KE, Banks E, Fennell T, et al. Analysis of protein-coding genetic variation in 60,706 humans. Nature. 2016;536:285-91.
12. Weigelt B, Bi R, Kumar R, Blecua P, Mandelker DL, Geyer FC, et al. The Landscape of Somatic Genetic Alterations in Breast Cancers From ATM Germline Mutation Carriers. J Natl Cancer Inst. 2018;110:1030-4.
13. Shen R, Seshan VE. FACETS: allele-specific copy number and clonal heterogeneity analysis tool for high-throughput DNA sequencing. Nucleic Acids Res. 2016;44:e131.
14. Schultheis AM, Ng CK, De Filippo MR, Piscuoglio S, Macedo GS, Gatius S, et al. Massively Parallel Sequencing-Based Clonality Analysis of Synchronous Endometrioid Endometrial and Ovarian Carcinomas. J Natl Cancer Inst. 2016;108:djv427.
15. Lee JY, Schizas M, Geyer FC, Selenica P, Piscuoglio S, Sakr RA, et al. Lobular Carcinomas In Situ Display Intralesion Genetic Heterogeneity and Clonal Evolution in the Progression to Invasive Lobular Carcinoma. Clin Cancer Res. 2019;25:674-86.
16. Ciriello G, Gatza ML, Beck AH, Wilkerson MD, Rhie SK, Pastore A, et al. Comprehensive Molecular Portraits of Invasive Lobular Breast Cancer. Cell. 2015;163:506-19.

Supplementary Figure S1

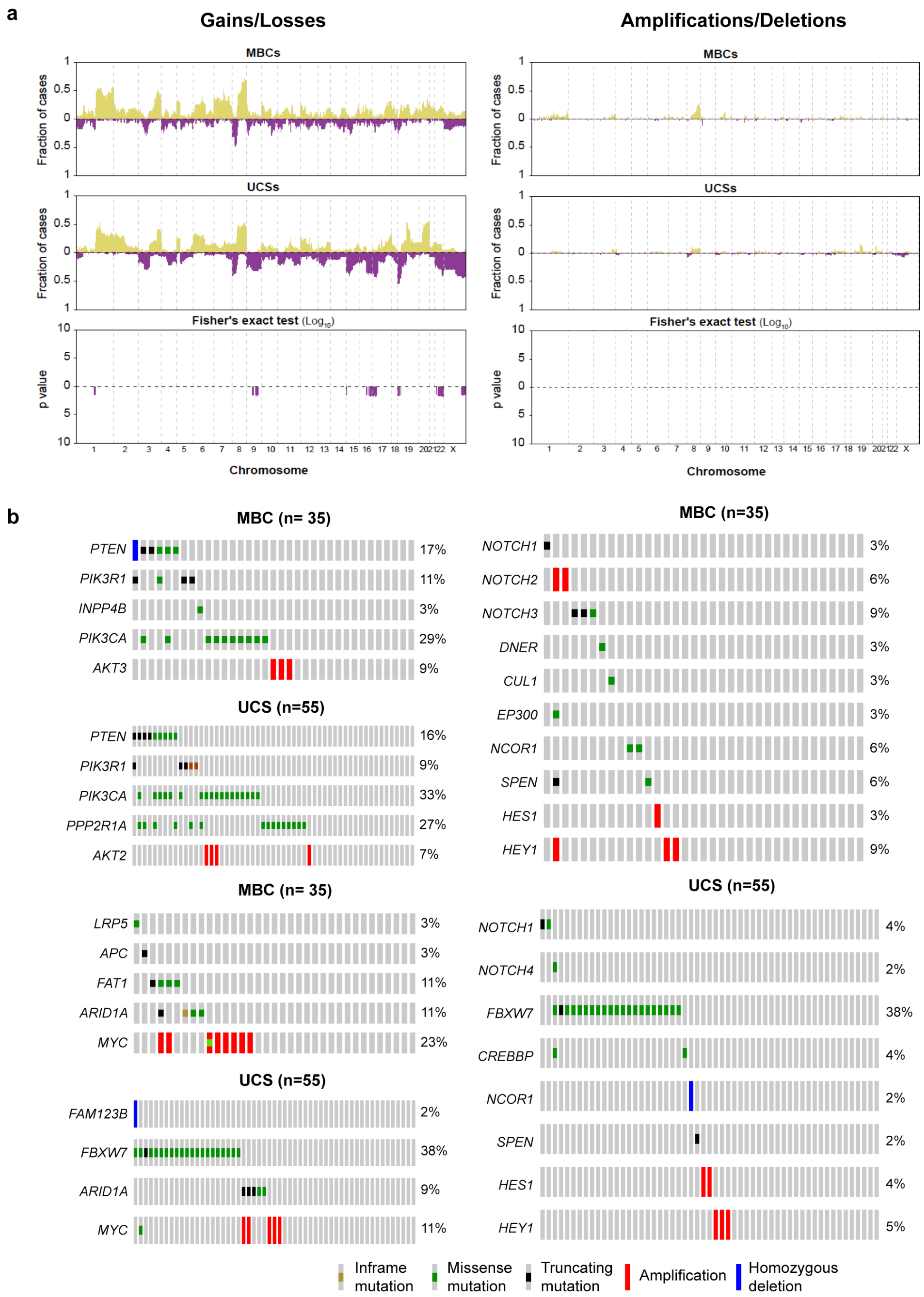

Supplementary Figure S1. Copy number alterations and somatic mutations affecting selected signaling pathways in metaplastic breast cancers and uterine carcinosarcomas. (**a**) Comparisons of the frequencies of copy number alterations (gains and losses, left; amplifications and homozygous deletions, right) metaplastic breast cancers (MBCs, n=35, top) and uterine carcinosarcomas (UCSs, n=55, middle). Fisher's exact test is shown in the bottom. (**b**) Genetic alterations in key pathways. Oncoprint of altered genes of the canonical PI3K/AKT/mTOR, Wnt and Notch pathways in metaplastic breast cancers (MBC, n=35) and uterine carcinosarcomas (UCSs, n=55). Cases are shown in columns and genes in rows. Somatic mutations and copy number alterations are color-coded according to the legend.

Supplementary Figure S2

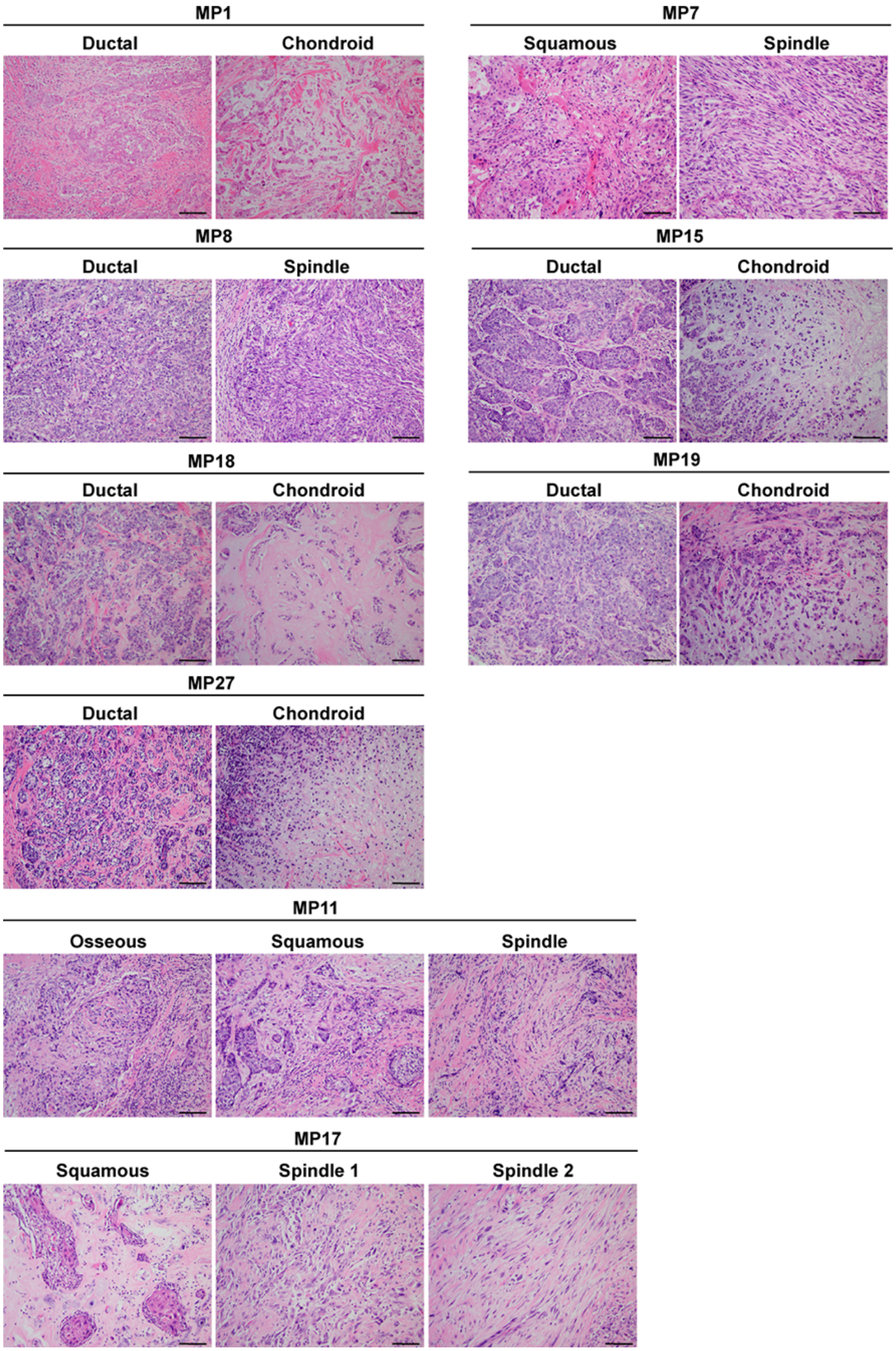

Supplementary Figure S2. Representative micrographs of the histologic components of the metaplastic breast cancers subjected to bulk whole-exome sequencing as well as to whole-exome sequencing of the distinct microdissected components. Please note that for MP21 micrographs were not available.

Supplementary Figure S3

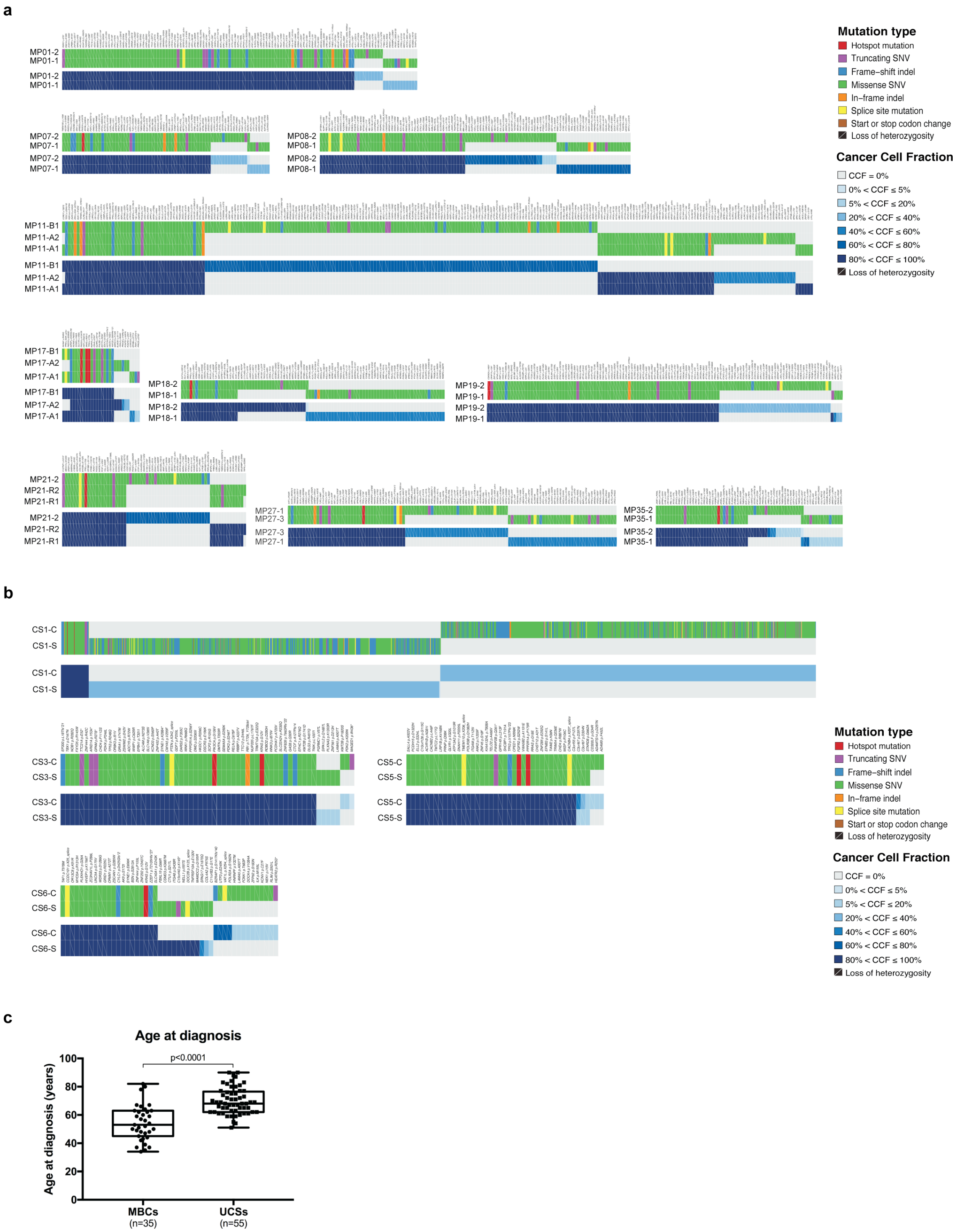

Supplementary Figure S3. Clonal decomposition of the epithelial and mesenchymal components of the metaplastic breast carcinomas and uterine carcinosarcomas. Clonal frequency heatmaps of mutations in the epithelial and mesenchymal histologic components of (**a**) MP1, MP7, MP8, MP11, MP17, MP18, MP19, MP21, MP27, MP35 and of (**b**) CS1, CS3, CS5, and CS6. Mutations are shown grouped by their clonal structure as inferred by PyClone. (**c**) Age at diagnosis of patients with metaplastic breast cancer (MBC, median 53 years, range 34-82, n=35) and with uterine carcinosarcoma (UCS, median 68 years, range 51-90, n=55). Mann-Whitney *U* test.

Supplementary Table S1. Clinico-pathologic information of the 35 metaplastic breast cancers re-analyzed from Ng et al (Clin Cancer Res 2017), 57 uterine carcinosarcomas from Cherniack et al (TCGA, Cancer Cell 2017), 11 metaplastic breast cancers with separately analyzed histologic components (this study), and 6 uterine carcinosarcomas with separately analyzed histologic components (this study).

METAPLASTIC BREAST CANCERS (BULK WHOLE-EXOME SEQUENCING)

Sample ID	Diagnosis	Matrix producing	Predominant Cellular Type	Histologic grade*	ER**	PR	HER2	Triple-negative phenotype	Age at diagnosis (years)	Tumor size (cm)	pT stage	pN stage
META31	Carcinoma with mesenchymal elements	Yes	Chondroid	2	-	-	-	Yes	59	1.2	T1c	N0
META33	Carcinoma with mesenchymal elements	No	Chondroid	3	-	-	-	Yes	44	0.7	T1b	N0
META39	Carcinoma with mesenchymal elements	No	Spindle	3	-	-	-	Yes	45	5.5	T3	N0
META40	Spindle cell carcinoma	No	Squamous	3	-	-	-	Yes	42	4	T2	N0
META42	Carcinoma with squamous metaplasia	No	Squamous	3	-	-	-	Yes	48	3.1	T2	N0
META52	Carcinoma with mesenchymal elements	No	Chondroid	3	-	-	-	Yes	59	3.3	T2	N0
META55	Carcinoma with mesenchymal elements	Yes	Chondroid	3	-	-	-	Yes	50	9	T3	N1a
META57	Spindle cell carcinoma	No	Spindle	3	-	-	-	Yes	56	2.3	T2	N0
META61	Spindle cell carcinoma	No	Spindle	2	-	-	-	Yes	67	1.8	T1c	N1mi
MP1	Carcinoma with mesenchymal elements	Yes	Chondroid	3	-	-	-	Yes	50	1.4	T1c	N0
MP11	Carcinoma with squamous metaplasia	No	Squamous	3	-	-	-	Yes	37	2.2	T2	N0
MP15	Carcinoma with mesenchymal elements	Yes	Chondroid	3	-	-	-	Yes	63	2.7	T2	N1mi
MP17	Spindle cell carcinoma	No	Spindle	3	-	-	-	Yes	66	14	T3	N/A
MP18	Carcinoma with mesenchymal elements	Yes	Chondroid	3	-	-	-	Yes	39	1.7	T1c	N0
MP19	Carcinoma with mesenchymal elements	Yes	Chondroid	3	-	-	-	Yes	52	1.3	T1c	N0
MP21	Spindle cell carcinoma	No	Spindle	3	-	-	-	Yes	37	2.4	T2	N0
MP27	Carcinoma with mesenchymal elements	Yes	Chondroid	3	-	-	-	Yes	57	1.1	T1c	N0
MP7	Spindle cell carcinoma	No	Spindle	3	-	-	-	Yes	51	2.5	T2	N0
MP8	Spindle cell carcinoma	No	Spindle	3	-	-	-	Yes	34	2.9	T2	N0
MTC01	Spindle cell carcinoma	No	Spindle	3	-	-	-	Yes	67	3.6	T2	N0
MTC03	Spindle cell carcinoma	No	Spindle	3	-	-	-	Yes	43	3.2	T2	N0
MTC04	Carcinoma with mesenchymal elements	Yes	Chondroid	3	-	-	-	Yes	48	2.3	T2	N0
MTC06	Carcinoma with mesenchymal elements	Yes	Chondroid	2	-	-	-	Yes	53	1	T1b	N0
MTC07	Carcinoma with mesenchymal elements	Yes	Chondroid	3	-	-	-	Yes	60	2.2	T2	N0
MTC11	Carcinoma with mesenchymal elements	No	Chondroid	3	-	-	-	Yes	63	4	T2	N3
MTC12	Carcinoma with squamous metaplasia	No	Squamous	2	-	-	-	Yes	62	3.5	T2	N0
MTC13	Carcinoma with mesenchymal elements	Yes	Chondroid	2	-	-	-	Yes	47	4	T2	N0
MTC14	Carcinoma with mesenchymal elements	Yes	Chondroid	3	-	-	-	Yes	35	3.1	T2	N0
MTC15	Spindle cell carcinoma	No	Spindle	3	-	-	-	Yes	80	5	T2	N0
MTC16	Carcinoma with squamous metaplasia	No	Squamous	3	-	-	-	Yes	54	3.2	T2	N0
MTC17	Carcinoma with squamous metaplasia	No	Squamous	3	-	-	-	Yes	82	7.8	T3	N3
MTC18	Carcinoma with squamous metaplasia	No	Squamous	3	+ (1% weak)	+ (20% weak)	-	No	62	5.2	T3	N0
MTC19	Carcinoma with mesenchymal elements	Yes	Chondroid	3	-	-	-	Yes	64	3	T2	N0
MTC20	Carcinoma with squamous metaplasia	No	Squamous	3	-	-	-	Yes	78	3	T2	N0
MTC23	Carcinoma with squamous metaplasia	No	Squamous	3	-	-	HER2/CEP17 FISH ratio 2.3	No	49	12	T3	N2

UTERINE CARCINOSARCOMAS (BULK WHOLE-EXOME SEQUENCING)

Sample ID	Histological Type	Clinical Stage	Age at diagnosis (years)	Frozen Sarcoma (%)	Frozen Carcinoma (%)	Serous (%)	Endometroid (%)	Undifferentiated Sarcoma (%)	Leiomyosarcoma (%)	Heterologous cartilage (%)	Heterologous rhabdomyosarcoma (%)	Histologic classification
TCGA-N5-A4R8	Uterine Carcinosarcoma/ MMMT: Heterologous Type	Stage IA	65	N/A	N/A	0	15	25	0	0	60	Serous-like
TCGA-N5-A4RA	Uterine Carcinosarcoma/ MMMT: Heterologous Type	Stage IIIC2	63	98	2	5	0	95	0	0	0	Serous-like
TCGA-N5-A4RD	Uterine Carcinosarcoma/ MMMT: Heterologous Type	Stage IVB	69	95	5	30	0	70	0	0	0	Serous-like
TCGA-N5-A4RF	Uterine Carcinosarcoma/ MMMT: Homologous Type	Stage IA	68	98	2	0	40	60	0	0	0	Endometrioid-like
TCGA-N5-A4RJ	Uterine Carcinosarcoma/ MMMT: Heterologous Type	Stage IVB	61	70	30	40	0	60	0	0	0	Serous-like
TCGA-N5-A4RM	Uterine Carcinosarcoma/ MMMT: Heterologous Type	Stage IVB	62	90	10	0	20	80	0	0	0	Serous-like
TCGA-N5-A4RN	Uterine Carcinosarcoma/ MMMT: Homologous Type	Stage IVB	71	40	60	0	30	70	0	0	0	Serous-like
TCGA-N5-A4RO	Uterine Carcinosarcoma/ MMMT: Homologous Type	Stage IIIC2	67	90	10	50	0	50	0	0	0	Serous-like
TCGA-N5-A4RS	Uterine Carcinosarcoma/ MMMT: Homologous Type	Stage IB	65	50	50	70	0	30	0	0	0	Serous-like
TCGA-N5-A4RT	Uterine Carcinosarcoma/ MMMT: Homologous Type	Stage IIIC1	69	80	20	80	0	20	0	0	0	Serous-like
TCGA-N5-A4RU	Uterine Carcinosarcoma/ MMMT: Homologous Type	Stage IIIC2	54	70	30	60	0	40	0	0	0	Serous-like
TCGA-N5-A4RV	Uterine Carcinosarcoma/ MMMT: Homologous Type	Stage IA	62	100	0	20	0	70	0	0	0	Serous-like
TCGA-N5-A59E	Uterine Carcinosarcoma/ MMMT: Heterologous Type	Stage IVB	62	90	10	0	0	75	0	0	0	Serous-like
TCGA-N5-A59F	Uterine Carcinosarcoma/ MMMT: Homologous Type	Stage IB	65	60	40	80	0	20	0	0	0	Serous-like
TCGA-N6-A4V9	Uterine Carcinosarcoma/ Malignant Mixed Mullerian Tumor (MMMT): NOS	Stage IA	59	80	20	0	0	85	0	0	0	Serous-like
TCGA-N6-A4VC	Uterine Carcinosarcoma/ Malignant Mixed Mullerian Tumor (MMMT): NOS	Stage III	83	40	60	0	70	30	0	0	0	Serous-like
TCGA-N6-A4VD	Uterine Carcinosarcoma/ Malignant Mixed Mullerian Tumor (MMMT): NOS	Stage IIIA	81	60	40	20	20	60	0	0	0	Endometrioid-like
TCGA-N6-A4VE	Uterine Carcinosarcoma/ Malignant Mixed Mullerian Tumor (MMMT): NOS	Stage IIIC	84	30	70	0	0	30	0	40	0	Serous-like
TCGA-N6-A4VF	Uterine Carcinosarcoma/ Malignant Mixed Mullerian Tumor (MMMT): NOS	Stage IB	51	70	30	0	0	70	0	0	0	Endometrioid-like
TCGA-N6-A4VG	Uterine Carcinosarcoma/ Malignant Mixed Mullerian Tumor (MMMT): NOS	Stage IVB	77	60	40	30	0	70	0	0	0	Serous-like
TCGA-N7-A4Y0	Uterine Carcinosarcoma/ MMMT: Heterologous Type	Stage IIIC	65	N/A	N/A	0	30	0	0	30	0	Endometrioid-like
TCGA-N7-A4Y5	Uterine Carcinosarcoma/ MMMT: Heterologous Type	Stage IIB	87	N/A	N/A	40	0	0	0	0	60	Serous-like
TCGA-N7-A4Y8	Uterine Carcinosarcoma/ MMMT: Heterologous Type	Stage IVB	72	100	N/A	0	0	0	0	0	0	Serous-like
TCGA-N7-A59B	Uterine Carcinosarcoma/ MMMT: Homologous Type	Stage IA	69	N/A	100	0	0	30	0	0	0	Serous-like
TCGA-N8-A4PI	Uterine Carcinosarcoma/ MMMT: Heterologous Type	Stage IA	71	60	40	20	10	70	0	0	0	Endometrioid-like
TCGA-N8-A4PL	Uterine Carcinosarcoma/ Malignant Mixed Mullerian Tumor (MMMT): NOS	Stage IA	82	50	50	30	0	70	0	0	0	Serous-like
TCGA-N8-A4PM	Uterine Carcinosarcoma/ MMMT: Homologous Type	Stage IB	73	60	40	40	0	60	0	0	0	Serous-like
TCGA-N8-A4PN	Uterine Carcinosarcoma/ MMMT: Homologous Type	Stage IIIC1	68	100	N/A	15	0	80	0	0	0	Serous-like
TCGA-N8-A4PO	Uterine Carcinosarcoma/ MMMT: Heterologous Type	Stage II	66	70	30	30	0	70	0	0	0	Serous-like
TCGA-N8-A4PP	Uterine Carcinosarcoma/ MMMT: Homologous Type	Stage IIIC1	88	N/A	N/A	15	0	85	0	0	0	Serous-like
TCGA-N8-A4PQ	Uterine Carcinosarcoma/ MMMT: Heterologous Type	Stage IIIC2	76	100	N/A	10	0	88	0	0	0	Serous-like
TCGA-N8-A56S	Uterine Carcinosarcoma/ MMMT: Heterologous Type	Stage IB	75	50	50	40	0	50	0	10	0	Serous-like
TCGA-N9-A4PZ	Uterine Carcinosarcoma/ Malignant Mixed Mullerian Tumor (MMMT): NOS	Stage IIIC2	77	40	60	0	0	70	0	0	0	Serous-like
TCGA-N9-A4Q1	Uterine Carcinosarcoma/ Malignant Mixed Mullerian Tumor (MMMT): NOS	Stage II	70	N/A	N/A	0	0	65	0	10	0	Serous-like
TCGA-N9-A4Q3	Uterine Carcinosarcoma/ Malignant Mixed Mullerian Tumor (MMMT): NOS	Stage IA	65	40	60	0	0	30	0	0	0	Serous-like
TCGA-N9-A4Q4	Uterine Carcinosarcoma/ Malignant Mixed Mullerian Tumor (MMMT): NOS	Stage IIIC	61	80	20	0	0	10	0	0	85	Serous-like
TCGA-N9-A4Q7	Uterine Carcinosarcoma/ Malignant Mixed Mullerian Tumor (MMMT): NOS	Stage IA	69	30	70	0	0	0	0	0	0	Serous-like
TCGA-N9-A4Q8	Uterine Carcinosarcoma/ Malignant Mixed Mullerian Tumor (MMMT): NOS	Stage IA	68	30	70	30	0	70	0	0	0	Serous-like
TCGA-NA-A4QV	Uterine Carcinosarcoma/ Malignant Mixed Mullerian Tumor (MMMT): NOS	Stage IB	72	90	10	0	0	50	0	0	0	Serous-like
TCGA-NA-A4QW	Uterine Carcinosarcoma/ Malignant Mixed Mullerian Tumor (MMMT): NOS	Stage IIA	61	100	0	0	0	0	0	0	90	Serous-like
TCGA-NA-A4QX	Uterine Carcinosarcoma/ Malignant Mixed Mullerian Tumor (MMMT): NOS	Stage IVB	60	100	0	20	0	80	0	0	0	Serous-like
TCGA-NA-A4QY	Uterine Carcinosarcoma/ MMMT: Heterologous Type	Stage III	62	70	30	0	0	1	0	0	0	Endometrioid-like
TCGA-NA-A4R0	Uterine Carcinosarcoma/ MMMT: Heterologous Type	Stage IVB	80	0	20	100	0	0	0	0	0	Serous-like
TCGA-NA-A4R1	Uterine Carcinosarcoma/ Malignant Mixed Mullerian Tumor (MMMT): NOS	Stage IIIC1	75	0	0	20	0	50	10	0	0	Endometrioid-like
TCGA-NA-A5I1	Uterine Carcinosarcoma/ Malignant Mixed Mullerian Tumor (MMMT): NOS	Stage IIIB	80	70	30	30	0	30	0	20	0	Serous-like
TCGA-ND-A4W6	Uterine Carcinosarcoma/ MMMT: Heterologous Type	Stage IIIC	67	70	30	40	0	60	0	0	0	Endometrioid-like
TCGA-ND-A4WA	Uterine Carcinosarcoma/ MMMT: Heterologous Type	Stage IC	90	0	0	20	0	25	0	25	0	None
TCGA-ND-A4WC	Uterine Carcinosarcoma/ MMMT: Homologous Type	Stage IB	83	0	30	80	0	20	0	0	0	None
TCGA-ND-A4WF	Uterine Carcinosarcoma/ MMMT: Heterologous Type	Stage IIIA	77	100	0	1	0	99	0	0	0	Serous-like
TCGA-NF-A4WU	Uterine Carcinosarcoma/ Malignant Mixed Mullerian Tumor (MMMT): NOS	Stage IA	59	0	100	90	0	10	0	0	0	Serous-like
TCGA-NF-A4WX	Uterine Carcinosarcoma/ MMMT: Heterologous Type	Stage IV	61	50	50	70	0	30	0	0	0	Serous-like
TCGA-NF-A4X2	Uterine Carcinosarcoma/ MMMT: Heterologous Type	Stage II	59	50	0	0	0	30	0	0	0	Endometrioid-like
TCGA-NF-A5CP	Uterine Carcinosarcoma/ Malignant Mixed Mullerian Tumor (MMMT): NOS	Stage IIIC2	90	60	40	40	0	20	40	0	0	Endometrioid-like
TCGA-NG-A4VU	Uterine Carcinosarcoma/ Malignant Mixed Mullerian Tumor (MMMT): NOS	Stage IIIB	63	100	0	30	0	60	0	0	10	Serous-like
TCGA-NG-A4VW	Uterine Carcinosarcoma/ Malignant Mixed Mullerian Tumor (MMMT): NOS	Stage IC	74	0	0	60	0	40	0	0	0	Endometrioid-like
TCGA-QM-A5NM	Uterine Carcinosarcoma/ Malignant Mixed Mullerian Tumor (MMMT): NOS	Stage IA	67	0	0	0	40	60	0	0	0	Endometrioid-like
TCGA-QN-A5NN	Uterine Carcinosarcoma/ Malignant Mixed Mullerian Tumor (MMMT): NOS	Stage IB	55	100	0	0	0	90	0	0	0	Serous-like

METAPLASTIC BREAST CANCERS: TWO/ THREE MICRODISSECTED COMPONENTS - WHOLE EXOME SEQUENCING

Case ID	Component	Component subtype	ER/PR/HER2**	Histologic Grade*	Age at diagnosis (years)	Stage
MP01	MP01-1	Chondroid	-/-/-	3	50	IC
	MP01-2	Ductal				
MP07	MP07-1	Spindle	-/-/-	3	51	II
	MP07-2	Squamous				
MP08	MP08-1	Spindle	-/-/-	3	34	II
	MP08-2	Ductal				
MP11	MP11-A1	Squamous	-/-/-	3	37	II
	MP11-A2	Spindle				
	MP11-B1	Osseous				
MP15	MP15-1	Chondroid	-/-/-	3	63	II
	MP15-2	Ductal				
MP17	MP17-A1	Spindle	-/-/-	3	66	III
	MP17-A2	Spindle				
	MP17-B1	Squamous				
MP18	MP18-1	Chondroid	-/-/-	3	39	IC
	MP18-2	Ductal				
MP19	MP19-1	Chondroid	-/-/-	3	52	IC
	MP19-2	Ductal				
MP21	MP21-R1	Spindle	-/-/-	3	37	II
	MP21-R2	Spindle				
	MP21-2	Ductal				
MP27	MP27-1	Chondroid	-/-/-	3	57	IC
	MP27-3	Ductal				
MP35	MP35-1	Chondroid	-/-/-	3	52	II
	MP35-2	Ductal				

UTERINE CARCINOSARCOMAS: TWO MICRODISSECTED COMPONENTS - WHOLE EXOME SEQUENCING

Case	Component	Component subtype	Histology	Age at diagnosis (years)	Stage
CS1	CS1-C	Carcinoma	Heterologous (Rhabdomyosarcoma)	84	IB
	CS1-S	Sarcoma			
CS3	CS3-C	Carcinoma	Homologous	68	IA
	CS3-S	Sarcoma			
CS4	CS4-C	Carcinoma	Heterologous	73	IIIA
	CS4-S	Sarcoma			
CS5	CS5-C	Carcinoma	Heterologous	88	IIIC
	CS5-S	Sarcoma			
CS6	CS5-C	Carcinoma	Homologous	79	IIIC
	CS5-S	Sarcoma			
CS8	CS5-C	Carcinoma	Homologous	82	IIIC
	CS5-S	Sarcoma			

* ER- estrogen receptor expression; PR- progesterone receptor expression; HER2- overexpression/amplification
** according to the Nottingham grading system

Supplementary Table S2: Non-synonymous somatic mutations identified in the epithelial and mesenchymal components of 11 metaplastic breast cancers and 6 uterine carcinosarcomas subjected to whole exome sequencing.

METAPLASTIC BREAST CANCERS - TWO/ THREE COMPONENTS

Sample ID	Histologic component	Gene symbol	Amino acid change	Shared/Private Mutations	Chromosome	Genomic position	Reference allele	Alternate allele	Type of mutation	Depth at mutation (x)	Mutant allele fraction	HOTSPOT	Cancer Cell Fraction (Pyclone)	Clonal Status	Mutation Taster	CHASM (breast)	FATHMM	Pathogenecity	Loss of heterozygosity (LOH)	Cancer Gene Census	Kandoth	Cancer5000-S genes (Lawrence et al)	Validated by AmpliSeq
MP1-1	Chondroid	ACSBG2	p.I431M	Private	19	6183254	A	G	Missense_Mutation	106	0.047169811	No	0.325	Subclonal	Neutral	Passenger	Neutral	passenger	Yes	No	No	No	Yes
MP1-1	Chondroid	ADCK1	p.D275G	Shared	14	78374228	A	G	Missense_Mutation	158	0.417721519	No	1	Clonal	Neutral	Passenger	Neutral	passenger	No	No	No	No	Not tested
MP1-2	Ductal	ADCK1	p.D275G	Shared	14	78374228	A	G	Missense_Mutation	172	0.290697674	No	1	Clonal	Neutral	Passenger	Neutral	passenger	No	No	No	No	Not tested
MP1-1	Chondroid	ADCY4	p.S499I	Shared	14	24798295	C	A	Missense_Mutation	211	0.27014218	No	1	Clonal	Disease_causing	Passenger	Neutral	passenger	Yes	No	No	No	Not tested
MP1-2	Ductal	ADCY4	p.S499I	Shared	14	24798295	C	A	Missense_Mutation	247	0.210526316	No	1	Clonal	Disease_causing	Passenger	Neutral	passenger	Yes	No	No	No	Not tested
MP1-1	Chondroid	ADNP	p.E787K	Shared	20	49508892	C	T	Missense_Mutation	258	0.282945736	No	1	Clonal	Disease_causing	Passenger	Disease_causing	likely_pathogenic	Yes	No	No	Yes	Not tested
MP1-1	Chondroid	ADNP	p.E785K	Shared	20	49508898	C	T	Missense_Mutation	256	0.27734375	No	1	Clonal	Disease_causing	Passenger	Disease_causing	likely_pathogenic	Yes	No	No	Yes	Not tested
MP1-1	Chondroid	ADNP	p.W796*	Shared	20	49508863	C	T	Nonsense_Mutation	267	0.31835206	No	1	Clonal	Disease_causing	.	.	likely_pathogenic	Yes	No	No	Yes	Not tested
MP1-1	Chondroid	ADNP	p.D1070N	Shared	20	49508043	C	T	Missense_Mutation	381	0.312335958	No	1	Clonal	Disease_causing	Passenger	.	passenger	Yes	No	No	Yes	Not tested
MP1-1	Chondroid	ADNP	p.E888Q	Shared	20	49508589	C	G	Missense_Mutation	374	0.262032086	No	1	Clonal	Neutral	Passenger	.	passenger	Yes	No	No	Yes	Not tested
MP1-1	Chondroid	ADNP	p.E747K	Shared	20	49509012	C	T	Missense_Mutation	298	0.325503356	No	1	Clonal	Disease_causing	Passenger	.	passenger	Yes	No	No	Yes	Not tested
MP1-2	Ductal	ADNP	p.E787K	Shared	20	49508892	C	T	Missense_Mutation	336	0.270833333	No	1	Clonal	Disease_causing	Passenger	Disease_causing	likely_pathogenic	Yes	No	No	Yes	Not tested
MP1-2	Ductal	ADNP	p.E785K	Shared	20	49508898	C	T	Missense_Mutation	328	0.274390244	No	1	Clonal	Disease_causing	Passenger	Disease_causing	likely_pathogenic	Yes	No	No	Yes	Not tested
MP1-2	Ductal	ADNP	p.W796*	Shared	20	49508863	C	T	Nonsense_Mutation	370	0.248648649	No	1	Clonal	Disease_causing	.	.	likely_pathogenic	Yes	No	No	Yes	Not tested
MP1-2	Ductal	ADNP	p.D1070N	Shared	20	49508043	C	T	Missense_Mutation	447	0.237136465	No	1	Clonal	Disease_causing	Passenger	.	passenger	Yes	No	No	Yes	Not tested
MP1-2	Ductal	ADNP	p.E888Q	Shared	20	49508589	C	G	Missense_Mutation	439	0.257403189	No	1	Clonal	Neutral	Passenger	.	passenger	Yes	No	No	Yes	Not tested
MP1-2	Ductal	ADNP	p.E747K	Shared	20	49509012	C	T	Missense_Mutation	362	0.262430939	No	1	Clonal	Disease_causing	Passenger	.	passenger	Yes	No	No	Yes	Not tested
MP1-1	Chondroid	AP3B2	p.K1014N	Shared	15	83328653	T	G	Missense_Mutation	157	0.305732484	No	1	Clonal	Disease_causing	Passenger	Neutral	passenger	Yes	No	No	No	Not tested
MP1-2	Ductal	AP3B2	p.K1014N	Shared	15	83328653	T	G	Missense_Mutation	247	0.226720648	No	1	Clonal	Disease_causing	Passenger	Neutral	passenger	Yes	No	No	No	Not tested
MP1-2	Ductal	ARMC3	p.K74N	Private	10	23244791	G	T	Missense_Mutation	236	0.06779661	No	0.561	Subclonal	Disease_causing	Passenger	Neutral	passenger	Yes	No	No	No	Yes
MP1-1	Chondroid	ATF6	p.C467*	Shared	1	161821590	TTGTCAGCCCC	T	Frame_Shift_Del	252	0.067460317	No	0.599	Subclonal	.	.	.	.	No	No	No	No	Not tested
MP1-2	Ductal	ATF6	p.C467*	Shared	1	161821590	TTGTCAGCCCC	T	Frame_Shift_Del	278	0.068345324	No	0.702	Subclonal	.	.	.	.	No	No	No	No	Not tested
MP1-1	Chondroid	ATG7	p.I229V	Shared	3	11372820	A	G	Missense_Mutation	156	0.185897436	No	1	Clonal	Neutral	Passenger	Neutral	passenger	No	No	No	No	Not tested
MP1-2	Ductal	ATG7	p.I229V	Shared	3	11372820	A	G	Missense_Mutation	208	0.173076923	No	1	Clonal	Neutral	Passenger	Neutral	passenger	No	No	No	No	Not tested
MP1-1	Chondroid	AUTS2	p.X634_splice	Private	7	70239086	G	A	Splice_Site	70	0.071428571	No	0.42	Subclonal	Disease_causing	.	.	passenger	Yes	No	No	No	Yes
MP1-1	Chondroid	BCLAF1	p.G278E	Shared	6	136599186	C	T	Missense_Mutation	179	0.134078212	No	0.789	Clonal	Disease_causing	Driver	Neutral	likely_pathogenic	Yes	No	No	Yes	Not tested
MP1-2	Ductal	BCLAF1	p.G278E	Shared	6	136599186	C	T	Missense_Mutation	192	0.109375	No	0.905	Clonal	Disease_causing	Driver	Neutral	likely_pathogenic	Yes	No	No	Yes	Not tested
MP1-1	Chondroid	BCR	p.R592Q	Shared	22	23610617	G	A	Missense_Mutation	93	0.290322581	No	1	Clonal	Disease_causing	Passenger	Neutral	passenger	Yes	Yes	No	No	Yes
MP1-2	Ductal	BCR	p.R592Q	Shared	22	23610617	G	A	Missense_Mutation	98	0.275510204	No	1	Clonal	Disease_causing	Passenger	Neutral	passenger	Yes	Yes	No	No	Yes
MP1-1	Chondroid	BRCA2	p.E2226Sfs*6	Shared	13	32915166	CAG	C	Frame_Shift_Del	200	0.22	No	1	Clonal	.	.	.	.	Yes	Yes	Yes	No	Not tested
MP1-2	Ductal	BRCA2	p.E2226Sfs*6	Shared	13	32915166	CAG	C	Frame_Shift_Del	301	0.176079734	No	1	Clonal	.	.	.	.	Yes	Yes	Yes	No	Not tested
MP1-1	Chondroid	BRSK1	p.P362A	Shared	19	55814734	C	G	Missense_Mutation	160	0.26875	No	1	Clonal	Disease_causing	Passenger	Neutral	passenger	Yes	No	No	No	Not tested
MP1-2	Ductal	BRSK1	p.P362A	Shared	19	55814734	C	G	Missense_Mutation	217	0.138248848	No	1	Clonal	Disease_causing	Passenger	Neutral	passenger	Yes	No	No	No	Not tested
MP1-2	Ductal	C1orf112	p.I167M	Private	1	169775167	C	G	Missense_Mutation	292	0.058219178	No	0.598	Subclonal	Disease_causing	Passenger	Neutral	passenger	No	No	No	No	Yes
MP1-1	Chondroid	CACNA1H	p.E2210Q	Shared	16	1270560	G	C	Missense_Mutation	79	0.278481013	No	1	Clonal	Neutral	Passenger	Neutral	passenger	Yes	No	No	No	Not tested
MP1-2	Ductal	CACNA1H	p.E2210Q	Shared	16	1270560	G	C	Missense_Mutation	93	0.247311828	No	1	Clonal	Neutral	Passenger	Neutral	passenger	Yes	No	No	No	Not tested
MP1-1	Chondroid	CCDC33	p.L338F	Shared	15	74573131	C	T	Missense_Mutation	57	0.192982456	No	1	Clonal	Neutral	Passenger	Neutral	passenger	Yes	No	No	No	Not tested
MP1-2	Ductal	CCDC33	p.L338F	Shared	15	74573131	C	T	Missense_Mutation	119	0.243697479	No	1	Clonal	Neutral	Passenger	Neutral	passenger	Yes	No	No	No	Not tested
MP1-1	Chondroid	CCHCR1	p.Q261E	Shared	6	31122293	G	C	Missense_Mutation	824	0.319174757	No	1	Clonal	Neutral	Passenger	Neutral	passenger	Yes	No	No	No	Not tested
MP1-2	Ductal	CCHCR1	p.Q261E	Shared	6	31122293	G	C	Missense_Mutation	967	0.215098242	No	1	Clonal	Neutral	Passenger	Neutral	passenger	Yes	No	No	No	Not tested
MP1-1	Chondroid	CD300E	p.E39D	Shared	17	72613528	C	G	Missense_Mutation	364	0.115384615	No	0.91	Clonal	Neutral	Passenger	Neutral	passenger	Yes	No	No	No	Not tested
MP1-2	Ductal	CD300E	p.E39D	Shared	17	72613528	C	G	Missense_Mutation	478	0.119246862	No	1	Clonal	Neutral	Passenger	Neutral	passenger	Yes	No	No	No	Not tested
MP1-1	Chondroid	CD320	p.D144Y	Shared	19	8368811	C	A	Missense_Mutation	318	0.273584906	No	1	Clonal	Neutral	Passenger	Disease_causing	passenger	Yes	No	No	No	Not tested
MP1-2	Ductal	CD320	p.D144Y	Shared	19	8368811	C	A	Missense_Mutation	345	0.176811594	No	1	Clonal	Neutral	Passenger	Disease_causing	passenger	Yes	No	No	No	Not tested
MP1-1	Chondroid	CD84	p.I343T	Shared	1	160518056	A	G	Missense_Mutation	505	0.152475248	No	1	Clonal	Neutral	Passenger	Neutral	passenger	No	No	No	No	Not tested
MP1-2	Ductal	CD84	p.I343T	Shared	1	160518056	A	G	Missense_Mutation	561	0.137254902	No	1	Clonal	Neutral	Passenger	Neutral	passenger	No	No	No	No	Not tested
MP1-1	Chondroid	CDH5	p.G167W	Shared	16	66421000	G	T	Missense_Mutation	103	0.252427184	No	1	Clonal	Disease_causing	Passenger	Neutral	passenger	Yes	No	No	No	Not tested
MP1-2	Ductal	CDH5	p.G167W	Shared	16	66421000	G	T	Missense_Mutation	84	0.226190476	No	1	Clonal	Disease_causing	Passenger	Neutral	passenger	Yes	No	No	No	Not tested
MP1-1	Chondroid	CENPP	p.G109E	Shared	9	95099859	G	A	Missense_Mutation	147	0.197278912	No	1	Clonal	Disease_causing	Passenger	.	passenger	Yes	No	No	No	Not tested
MP1-2	Ductal	CENPP	p.G109E	Shared	9	95099859	G	A	Missense_Mutation	200	0.235	No	1	Clonal	Disease_causing	Passenger	.	passenger	Yes	No	No	No	Not tested
MP1-1	Chondroid	CHEK2	p.W93S	Shared	22	29130432	C	G	Missense_Mutation	247	0.319838057	No	1	Clonal	Disease_causing	Passenger	Neutral	passenger	Yes	Yes	Yes	No	Yes
MP1-2	Ductal	CHEK2	p.W93S	Shared	22	29130432	C	G	Missense_Mutation	313	0.207667732	No	1	Clonal	Disease_causing	Passenger	Neutral	passenger	Yes	Yes	Yes	No	Yes
MP1-1	Chondroid	CHERP	p.D166N	Shared	19	16643848	C	T	Missense_Mutation	382	0.230366492	No	1	Clonal	Disease_causing	Passenger	Neutral	passenger	Yes	No	No	No	Yes
MP1-2	Ductal	CHERP	p.D166N	Shared	19	16643848	C	T	Missense_Mutation	453	0.181015453	No	1	Clonal	Disease_causing	Passenger	Neutral	passenger	Yes	No	No	No	Yes
MP1-1	Chondroid	COL18A1	p.W202_P208del	Shared	21	46876042	AGGCCCTGGGCACCACTCACGG	A	In_Frame_Del	293	0.116040956	No	0.799	Clonal	.	.	.	.	Yes	No	No	No	Not tested
MP1-2	Ductal	COL18A1	p.W202_P208del	Shared	21	46876042	AGGCCCTGGGCACCACTCACGG	A	In_Frame_Del	344	0.066860465	No	0.62	Clonal	.	.	.	.	Yes	No	No	No	Not tested
MP1-1	Chondroid	COL6A2	p.D739Sfs*13	Shared	21	47545943	GGACGATGACC	G	Frame_Shift_Del	284	0.073943662	No	0.509	Subclonal	.	.	.	.	Yes	No	No	No	Not tested
MP1-2	Ductal	COL6A2	p.D739Sfs*13	Shared	21	47545943	GGACGATGACC	G	Frame_Shift_Del	339	0.038348083	No	0.355	Subclonal	.	.	.	.	Yes	No	No	No	Not tested
MP1-1	Chondroid	CPB1	p.F28C	Shared	3	148545800	T	G	Missense_Mutation	378	0.164021164	No	1	Clonal	Disease_causing	Passenger	Neutral	passenger	No	No	No	No	Not tested
MP1-2	Ductal	CPB1	p.F28C	Shared	3	148545800	T	G	Missense_Mutation	430	0.118604651	No	1	Clonal	Disease_causing	Passenger	Neutral	passenger	No	No	No	No	Not tested
MP1-1	Chondroid	CPEB4	p.A518V	Shared	5	173376969	C	T	Missense_Mutation	116	0.448275862	No	1	Clonal	Disease_causing	Passenger	Neutral	passenger	No	No	No	No	Not tested
MP1-2	Ductal	CPEB4	p.A518V	Shared	5	173376969	C	T	Missense_Mutation	168	0.333333333	No	1	Clonal	Disease_causing	Passenger	Neutral	passenger	No	No	No	No	Not tested
MP1-1	Chondroid	CPQ	p.H104N	Shared	8	97797435	C	A	Missense_Mutation	207	0.251207729	No	1	Clonal	Disease_causing	Passenger	Neutral	passenger	Yes	No	No	No	Not tested
MP1-2	Ductal	CPQ	p.H104N	Shared	8	97797435	C	A	Missense_Mutation	310	0.270967742	No	1	Clonal	Disease_causing	Passenger	Neutral	passenger	Yes	No	No	No	Not tested
MP1-1	Chondroid	CXXC1	p.R439G	Shared	18	47810374	G	C	Missense_Mutation	374	0.259358289	No	1	Clonal	Disease_causing	Passenger	Neutral	passenger	Yes	No	No	No	Not tested
MP1-2	Ductal	CXXC1	p.R439G	Shared	18	47810374	G	C	Missense_Mutation	405	0.234567901	No	1	Clonal	Disease_causing	Passenger	Neutral	passenger	Yes	No	No	No	Not tested
MP1-1	Chondroid	DCAF12L2	p.R337H	Shared	X	125298898	C	T	Missense_Mutation	316	0.389240506	No	1	Clonal	Neutral	Passenger	Neutral	passenger	Yes	No	No	No	Not tested
MP1-2	Ductal	DCAF12L2	p.R337H	Shared	X	125298898	C	T	Missense_Mutation	384	0.260416667	No	1	Clonal	Neutral	Passenger	Neutral	passenger	Yes	No	No	No	Not tested
MP1-2	Ductal	DCBLD2	p.N418K	Private	3	98531285	A	T	Missense_Mutation	226	0.110619469	No	1	Subclonal	Disease_causing	Passenger	Disease_causing	likely_pathogenic	No	No	No	No	Not tested
MP1-1	Chondroid	DEFB125	p.X20_splice	Shared	20	76645	G	A	Splice_Site	109	0.330275229	No	1	Clonal	Disease_causing	.	.	passenger	Yes	No	No	No	Not tested
MP1-2	Ductal	DEFB125	p.X20_splice	Shared	20	76645	G	A	Splice_Site	139	0.251798561	No	1	Clonal	Disease_causing	.	.	passenger	Yes	No	No	No	Not tested
MP1-1	Chondroid	DENND5A	p.S433A	Shared	11	9202472	A	C	Missense_Mutation	242	0.272727273	No	1	Clonal	Disease_causing	Passenger	Neutral	passenger	Yes	No	No	No	Not tested
MP1-2	Ductal	DENND5A	p.S433A	Shared	11	9202472	A	C	Missense_Mutation	298	0.201342282	No	1	Clonal	Disease_causing	Passenger	Neutral	passenger	Yes	No	No	No	Not tested
MP1-1	Chondroid	DNAH6	p.F711Lfs*43	Shared	2	84806703	TC	T	Frame_Shift_Del	204	0.25	No	1	Clonal	.	.	.	.	No	No	No	No	Not tested
MP1-2	Ductal	DNAH6	p.F711Lfs*43	Shared	2	84806703	TC	T	Frame_Shift_Del	269	0.241635688	No	1	Clonal	.	.	.	.	No	No	No	No	Not tested
MP1-1	Chondroid	DOCK8	p.R2033C	Shared	9	463545	C	T	Missense_Mutation	226	0.221238938	No	1	Clonal	Disease_causing	Passenger	Neutral	passenger	Yes	No	No	No	Not tested
MP1-2	Ductal	DOCK8	p.R2033C	Shared	9	463545	C	T	Missense_Mutation	312	0.144230769	No	1	Clonal	Disease_causing	Passenger	Neutral	passenger	Yes	No	No	No	Not tested
MP1-1	Chondroid	ENPEP	p.S68*	Shared	4	111397773	C	G	Nonsense_Mutation	212	0.20754717	No	1	Clonal	Neutral	.	.	passenger	Yes	No	No	No	Not tested
MP1-2	Ductal	ENPEP	p.S68*	Shared	4	111397773	C	G	Nonsense_Mutation	234	0.286324786	No	1	Clonal	Neutral	.	.	passenger	Yes	No	No	No	Not tested
MP1-1	Chondroid	ENTPD1	p.Q354*	Shared	10	97607413	C	T	Nonsense_Mutation	237	0.261603376	No	1	Clonal	Neutral	.	.	passenger	Yes	No	No	No	Not tested
MP1-2	Ductal	ENTPD1	p.Q354*	Shared	10	97607413	C	T	Nonsense_Mutation	330	0.184848485	No	1	Clonal	Neutral	.	.	passenger	Yes	No	No	No	Not tested
MP1-1	Chondroid	EPHA4	p.P787T	Shared	2	222298999	G	T	Missense_Mutation	80	0.2375	No	1	Clonal	Disease_causing	Driver	Neutral	likely_pathogenic	No	No	No	No	Not tested
MP1-2	Ductal	EPHA4	p.P787T	Shared	2	222298999	G	T	Missense_Mutation	107	0.252336449	No	1	Clonal	Disease_causing	Driver	Neutral	likely_pathogenic	No	No	No	No	Not tested
MP1-1	Chondroid	ERMAP	p.L18V	Shared	1	43296171	C	G	Missense_Mutation	546	0.217948718	No	1	Clonal	Neutral	Passenger	Neutral	passenger	Yes	No	No	No	Not tested
MP1-2	Ductal	ERMAP	p.L18V	Shared	1	43296171	C	G	Missense_Mutation	798	0.180451128	No	1	Clonal	Neutral	Passenger	Neutral	passenger	Yes	No	No	No	Not tested
MP1-1	Chondroid	ESPL1	p.W530C	Shared	12	53668684	G	C	Missense_Mutation	174	0.25862069	No	1	Clonal	Disease_causing	Passenger	Neutral	passenger	Yes	No	No	No	Not tested
MP1-2	Ductal	ESPL1	p.W530C	Shared	12	53668684	G	C	Missense_Mutation	202	0.252475248	No	1	Clonal	Disease_causing	Passenger	Neutral	passenger	Yes	No	No	No	Not tested
MP1-1	Chondroid	ETV2	p.V308L	Shared	19	36135647	G	C	Missense_Mutation	205	0.317073171	No	1	Clonal	Disease_causing	Passenger	Neutral	passenger	Yes	No	No	No	Yes
MP1-2	Ductal	ETV2	p.V308L	Shared	19	36135647	G	C	Missense_Mutation	274	0.218978102	No	1	Clonal	Disease_causing	Passenger	Neutral	passenger	Yes	No	No	No	Yes
MP1-1	Chondroid	F5	p.M1909I	Shared	1	169494136	C	T	Missense_Mutation	128	0.125	No	1	Clonal	Neutral	Passenger	Disease_causing	passenger	No	No	No	No	Not tested
MP1-2	Ductal	F5	p.M1909I	Shared	1	169494136	C	T	Missense_Mutation	132	0.113636364	No	1	Clonal	Neutral	Passenger	Disease_causing	passenger	No	No	No	No	Not tested
MP1-1	Chondroid	FBXO10	p.E146*	Shared	9	37541330	C	A	Nonsense_Mutation	411	0.291970803	No	1	Clonal	Disease_causing	.	.	passenger	Yes	No	No	No	Not tested
MP1-2	Ductal	FBXO10	p.E146*	Shared	9	37541330	C	A	Nonsense_Mutation	473	0.190274841	No	1	Clonal	Disease_causing	.	.	passenger	Yes	No	No	No	Not tested
MP1-1	Chondroid	GEMIN4	p.F159V	Shared	17	650808	A	C	Missense_Mutation	304	0.417763158	No	1	Clonal	Disease_causing	Passenger	Neutral	passenger	No	No	No	No	Not tested
MP1-2	Ductal	GEMIN4	p.F159V	Shared	17	650808	A	C	Missense_Mutation	361	0.315789474	No	1	Clonal	Disease_causing	Passenger	Neutral	passenger	No	No	No	No	Not tested
MP1-1	Chondroid	GLRA4	p.R93W	Private	X	102979223	G	A	Missense_Mutation	63	0.142857143	No	0.841	Clonal	Disease_causing	Passenger	Neutral	passenger	Yes	No	No	No	Not tested
MP1-1	Chondroid	GREM2	p.K66N	Shared	1	240656578	C	G	Missense_Mutation	571	0.192644483	No	1	Clonal	Disease_causing	Passenger	Neutral	passenger	No	No	No	No	Not tested
MP1-2	Ductal	GREM2	p.K66N	Shared	1	240656578	C	G	Missense_Mutation	542	0.127306273	No	1	Clonal	Disease_causing	Passenger	Neutral	passenger	No	No	No	No	Not tested
MP1-2	Ductal	GRID2	p.S25W	Private	4	93225881	C	G	Missense_Mutation	333	0.036036036	No	0.298	Subclonal	Disease_causing	Passenger	Disease_causing	likely_pathogenic	Yes	No	No	No	Yes
MP1-1	Chondroid	HECTD4	p.R3706G	Shared	12	112613580	T	C	Missense_Mutation	125	0.272	No	1	Clonal	Disease_causing	Driver	Neutral	likely_pathogenic	Yes	No	No	No	Yes
MP1-2	Ductal	HECTD4	p.R3706G	Shared	12	112613580	T	C	Missense_Mutation	187	0.22459893	No	1	Clonal	Disease_causing	Driver	Neutral	likely_pathogenic	Yes	No	No	No	Yes
MP1-1	Chondroid	HMCN1	p.P4788T	Private	1	186114630	C	A	Missense_Mutation	261	0.103448276	No	0.712	Subclonal	Neutral	Passenger	Neutral	passenger	No	No	No	No	Yes
MP1-1	Chondroid	HR	p.R940Q	Shared	8	21977644	C	T	Missense_Mutation	356	0.376404494	No	1	Clonal	Neutral	Passenger	Neutral	passenger	Yes	No	No	No	Not tested
MP1-2	Ductal	HR	p.R940Q	Shared	8	21977644	C	T	Missense_Mutation	441	0.319727891	No	1	Clonal	Neutral	Passenger	Neutral	passenger	Yes	No	No	No	Not tested
MP1-1	Chondroid	HTR1E	p.T135M	Shared	6	87725456	C	T	Missense_Mutation	328	0.320121951	No	1	Clonal	Disease_causing	Passenger	Neutral	passenger	Yes	No	No	No	Not tested
MP1-2	Ductal	HTR1E	p.T135M	Shared	6	87725456	C	T	Missense_Mutation	340	0.223529412	No	1	Clonal	Disease_causing	Passenger	Neutral	passenger	Yes	No	No	No	Not tested
MP1-1	Chondroid	IKZF5	p.L19Q	Shared	10	124758086	A	T	Missense_Mutation	305	0.281967213	No	1	Clonal	Disease_causing	Passenger	Neutral	passenger	Yes	No	No	No	Not tested
MP1-2	Ductal	IKZF5	p.L19Q	Shared	10	124758086	A	T	Missense_Mutation	372	0.23655914	No	1	Clonal	Disease_causing	Passenger	Neutral	passenger	Yes	No	No	No	Not tested
MP1-1	Chondroid	IPO13	p.G953R	Shared	1	44433319	G	C	Missense_Mutation	332	0.388554217	No	1	Clonal	Disease_causing	Passenger	.	passenger	Yes	No	No	No	Not tested
MP1-2	Ductal	IPO13	p.G953R	Shared	1	44433319	G	C	Missense_Mutation	504	0.402777778	No	1	Clonal	Disease_causing	Passenger	.	passenger	Yes	No	No	No	Not tested
MP1-1	Chondroid	IRS4	p.Q158H	Shared	X	107979101	C	G	Missense_Mutation	241	0.157676349	No	0.928	Clonal	Neutral	Passenger	Neutral	passenger	Yes	No	No	No	Not tested
MP1-2	Ductal	IRS4	p.Q158H	Shared	X	107979101	C	G	Missense_Mutation	262	0.148854962	No	1	Clonal	Neutral	Passenger	Neutral	passenger	Yes	No	No	No	Not tested
MP1-1	Chondroid	ITPR3	p.A1013V	Private	6	33641477	C	T	Missense_Mutation	190	0.036842105	No	0.217	Subclonal	Disease_causing	Driver	Neutral	likely_pathogenic	Yes	No	No	No	Yes
MP1-1	Chondroid	IVD	p.A217_V219del	Shared	15	40703840	CTGGCTGCTG	C	In_Frame_Del	201	0.149253731	No	0.878	Clonal	.	.	.	.	Yes	No	No	No	Not tested
MP1-2	Ductal	IVD	p.A217_V219del	Shared	15	40703840	CTGGCTGCTG	C	In_Frame_Del	267	0.119850187	No	0.991	Clonal	.	.	.	.	Yes	No	No	No	Not tested
MP1-1	Chondroid	KDM6A	p.R519Dfs*29	Shared	X	44922693	AC	A	Frame_Shift_Del	470	0.242553191	No	1	Clonal	.	.	.	.	Yes	Yes	Yes	Yes	Yes
MP1-2	Ductal	KDM6A	p.R519Dfs*29	Shared	X	44922693	AC	A	Frame_Shift_Del	630	0.174603175	No	1	Clonal	.	.	.	.	Yes	Yes	Yes	Yes	Yes
MP1-1	Chondroid	KIAA0232	p.S1114F	Shared	4	6865450	C	T	Missense_Mutation	175	0.388571429	No	1	Clonal	Disease_causing	Passenger	.	passenger	No	No	No	No	Not tested
MP1-2	Ductal	KIAA0232	p.S1114F	Shared	4	6865450	C	T	Missense_Mutation	240	0.2875	No	1	Clonal	Disease_causing	Passenger	.	passenger	No	No	No	No	Not tested
MP1-1	Chondroid	KIAA1549	p.A1496Sfs*52	Shared	7	138555958	ACGGGAGGTGC	A	Frame_Shift_Del	311	0.279742765	No	1	Clonal	.	.	.	.	Yes	No	No	No	Not tested
MP1-2	Ductal	KIAA1549	p.A1496Sfs*52	Shared	7	138555958	ACGGGAGGTGC	A	Frame_Shift_Del	358	0.215083799	No	1	Clonal	.	.	.	.	Yes	No	No	No	Not tested
MP1-2	Ductal	KLF11	p.D221A	Private	2	10188126	A	C	Missense_Mutation	569	0.033391916	No	0.31	Subclonal	Neutral	Passenger	Neutral	passenger	No	No	No	No	Not tested
MP1-1	Chondroid	KRT9	p.G615R	Shared	17	39723554	C	T	Missense_Mutation	244	0.307377049	No	1	Clonal	Neutral	Passenger	Disease_causing	passenger	Yes	No	No	No	Not tested
MP1-2	Ductal	KRT9	p.G615R	Shared	17	39723554	C	T	Missense_Mutation	235	0.217021277	No	1	Clonal	Neutral	Passenger	Disease_causing	passenger	Yes	No	No	No	Not tested
MP1-1	Chondroid	L3MBTL3	p.S31R	Shared	6	130363937	T	A	Missense_Mutation	327	0.256880734	No	1	Clonal	Disease_causing	Driver	Neutral	likely_pathogenic	Yes	No	No	No	Not tested
MP1-2	Ductal	L3MBTL3	p.S31R	Shared	6	130363937	T	A	Missense_Mutation	404	0.193069307	No	1	Clonal	Disease_causing	Driver	Neutral	likely_pathogenic	Yes	No	No	No	Not tested
MP1-1	Chondroid	MAFB	p.C298W	Shared	20	39316597	G	C	Missense_Mutation	206	0.22815534	No	1	Clonal	Disease_causing	Passenger	Disease_causing	likely_pathogenic	Yes	Yes	No	No	Not tested
MP1-2	Ductal	MAFB	p.C298W	Shared	20	39316597	G	C	Missense_Mutation	213	0.230046948	No	1	Clonal	Disease_causing	Passenger	Disease_causing	likely_pathogenic	Yes	Yes	No	No	Not tested
MP1-1	Chondroid	MAML2	p.A5G	Shared	11	96075046	G	C	Missense_Mutation	268	0.436567164	No	1	Clonal	Disease_causing	Passenger	Neutral	passenger	No	Yes	No	No	Yes
MP1-2	Ductal	MAML2	p.A5G	Shared	11	96075046	G	C	Missense_Mutation	326	0.309815951	No	1	Clonal	Disease_causing	Passenger	Neutral	passenger	No	Yes	No	No	Yes
MP1-1	Chondroid	MED12	p.S402Y	Private	X	70342153	C	A	Missense_Mutation	279	0.053763441	No	0.316	Subclonal	Disease_causing	Passenger	Neutral	passenger	Yes	Yes	No	Yes	Yes
MP1-1	Chondroid	MED12	p.Q415K	Private	X	70342191	C	A	Missense_Mutation	282	0.056737589	No	0.334	Subclonal	Disease_causing	Passenger	Neutral	passenger	Yes	Yes	No	Yes	Yes
MP1-1	Chondroid	MME	p.R385*	Shared	3	154860084	C	T	Nonsense_Mutation	331	0.129909366	No	1	Clonal	Neutral	.	.	passenger	No	No	No	No	Not tested
MP1-2	Ductal	MME	p.R385*	Shared	3	154860084	C	T	Nonsense_Mutation	359	0.114206128	No	1	Clonal	Neutral	.	.	passenger	No	No	No	No	Not tested
MP1-1	Chondroid	MOB1B	p.Y31*	Private	4	71824654	T	G	Nonsense_Mutation	147	0.054421769	No	0.32	Subclonal	Neutral	.	.	passenger	Yes	No	No	No	Yes
MP1-1	Chondroid	MROH7	p.Q36P	Shared	1	55118706	A	C	Missense_Mutation	170	0.394117647	No	1	Clonal	Neutral	Passenger	Neutral	passenger	No	No	No	No	Not tested
MP1-2	Ductal	MROH7	p.Q36P	Shared	1	55118706	A	C	Missense_Mutation	241	0.34439834	No	1	Clonal	Neutral	Passenger	Neutral	passenger	No	No	No	No	Not tested
MP1-1	Chondroid	MRPL14	p.R37Q	Shared	6	44081908	C	T	Missense_Mutation	298	0.32885906	No	1	Clonal	Disease_causing	Passenger	.	passenger	Yes	No	No	No	Not tested
MP1-2	Ductal	MRPL14	p.R37Q	Shared	6	44081908	C	T	Missense_Mutation	362	0.223756906	No	1	Clonal	Disease_causing	Passenger	.	passenger	Yes	No	No	No	Not tested
MP1-2	Ductal	NAT16	p.D291Y	Private	7	100815599	C	A	Missense_Mutation	169	0.082840237	No	0.685	Subclonal	Disease_causing	Passenger	Neutral	passenger	Yes	No	No	No	Yes
MP1-1	Chondroid	NCAN	p.W817L	Shared	19	19338879	G	T	Missense_Mutation	216	0.217592593	No	1	Clonal	Neutral	Passenger	Disease_causing	passenger	Yes	No	No	No	Not tested
MP1-2	Ductal	NCAN	p.W817L	Shared	19	19338879	G	T	Missense_Mutation	241	0.182572614	No	1	Clonal	Neutral	Passenger	Disease_causing	passenger	Yes	No	No	No	Not tested
MP1-2	Ductal	NECAB2	p.D329Y	Private	16	84034351	G	T	Missense_Mutation	164	0.06097561	No	0.504	Subclonal	Disease_causing	Passenger	Neutral	passenger	Yes	No	No	No	Not tested
MP1-1	Chondroid	NEO1	p.L916F	Shared	15	73562686	G	C	Missense_Mutation	191	0.188481675	No	1	Clonal	Disease_causing	Passenger	Neutral	passenger	Yes	No	No	No	Not tested
MP1-2	Ductal	NEO1	p.L916F	Shared	15	73562686	G	C	Missense_Mutation	240	0.195833333	No	1	Clonal	Disease_causing	Passenger	Neutral	passenger	Yes	No	No	No	Not tested
MP1-1	Chondroid	NLRC3	p.G566S	Shared	16	3613383	C	T	Missense_Mutation	155	0.258064516	No	1	Clonal	Disease_causing	Passenger	Disease_causing	likely_pathogenic	Yes	No	No	No	Not tested
MP1-2	Ductal	NLRC3	p.G566S	Shared	16	3613383	C	T	Missense_Mutation	182	0.181318681	No	1	Clonal	Disease_causing	Passenger	Disease_causing	likely_pathogenic	Yes	No	No	No	Not tested
MP1-1	Chondroid	NOTCH3	p.R2234Qfs*2	Shared	19	15271721	TGGGAACCCGCAGGAAGC	T	Frame_Shift_Del	197	0.228426396	No	1	Clonal	.	.	.	.	Yes	No	No	No	Yes
MP1-2	Ductal	NOTCH3	p.R2234Qfs*2	Shared	19	15271721	TGGGAACCCGCAGGAAGC	T	Frame_Shift_Del	252	0.23015873	No	1	Clonal	.	.	.	.	Yes	No	No	No	Yes
MP1-1	Chondroid	OSBPL1A	p.I716Sfs*17	Shared	18	21752396	G	GAT	Frame_Shift_Ins	194	0.216494845	No	1	Clonal	.	.	.	.	Yes	No	No	No	Not tested
MP1-2	Ductal	OSBPL1A	p.I716Sfs*17	Shared	18	21752396	G	GAT	Frame_Shift_Ins	246	0.215447154	No	1	Clonal	.	.	.	.	Yes	No	No	No	Not tested
MP1-1	Chondroid	PALD1	p.Y428D	Shared	10	72297621	T	G	Missense_Mutation	57	0.263157895	No	1	Clonal	Disease_causing	Driver	Neutral	likely_pathogenic	Yes	No	No	No	Not tested
MP1-2	Ductal	PALD1	p.Y428D	Shared	10	72297621	T	G	Missense_Mutation	97	0.18556701	No	1	Clonal	Disease_causing	Driver	Neutral	likely_pathogenic	Yes	No	No	No	Not tested
MP1-1	Chondroid	PCSK1	p.R740G	Shared	5	95728749	G	C	Missense_Mutation	286	0.272727273	No	1	Clonal	Disease_causing	Passenger	Neutral	passenger	Yes	No	No	No	Not tested
MP1-2	Ductal	PCSK1	p.R740G	Shared	5	95728749	G	C	Missense_Mutation	345	0.197101449	No	1	Clonal	Disease_causing	Passenger	Neutral	passenger	Yes	No	No	No	Not tested
MP1-1	Chondroid	PIK3R1	p.S652Lfs*28	Private	5	67592135	AGCAGTAAACAGGGCTGCTAT	A	Frame_Shift_Del	258	0.15503876	No	0.913	Clonal	.	.	.	.	Yes	Yes	Yes	Yes	Yes
MP1-1	Chondroid	PNPO	p.L22V	Shared	17	46019105	C	G	Missense_Mutation	384	0.3046875	No	1	Clonal	Neutral	Passenger	Neutral	passenger	Yes	No	No	No	Not tested
MP1-2	Ductal	PNPO	p.L22V	Shared	17	46019105	C	G	Missense_Mutation	461	0.264642082	No	1	Clonal	Neutral	Passenger	Neutral	passenger	Yes	No	No	No	Not tested
MP1-1	Chondroid	PPARGC1B	p.S994L	Shared	5	149227030	C	T	Missense_Mutation	371	0.285714286	No	1	Clonal	Neutral	Passenger	Neutral	passenger	Yes	No	No	No	Not tested
MP1-2	Ductal	PPARGC1B	p.S994L	Shared	5	149227030	C	T	Missense_Mutation	513	0.230019493	No	1	Clonal	Neutral	Passenger	Neutral	passenger	Yes	No	No	No	Not tested
MP1-1	Chondroid	PPP1R3C	p.D274Y	Shared	10	93389818	C	A	Missense_Mutation	296	0.307432432	No	1	Clonal	Neutral	Passenger	Neutral	passenger	Yes	No	No	No	Not tested
MP1-2	Ductal	PPP1R3C	p.D274Y	Shared	10	93389818	C	A	Missense_Mutation	364	0.258241758	No	1	Clonal	Neutral	Passenger	Neutral	passenger	Yes	No	No	No	Not tested
MP1-1	Chondroid	RGS16	p.E138K	Shared	1	182569624	C	T	Missense_Mutation	151	0.463576159	No	1	Clonal	Disease_causing	Passenger	Neutral	passenger	No	No	No	No	Not tested
MP1-2	Ductal	RGS16	p.E138K	Shared	1	182569624	C	T	Missense_Mutation	121	0.388429752	No	1	Clonal	Disease_causing	Passenger	Neutral	passenger	No	No	No	No	Not tested
MP1-1	Chondroid	RSRC1	p.S70*	Shared	3	157841669	C	A	Nonsense_Mutation	355	0.563380282	No	1	Clonal	Neutral	.	.	passenger	No	No	No	No	Not tested
MP1-2	Ductal	RSRC1	p.S70*	Shared	3	157841669	C	A	Nonsense_Mutation	419	0.443914081	No	1	Clonal	Neutral	.	.	passenger	No	No	No	No	Not tested
MP1-1	Chondroid	SCRIB	p.L186M	Shared	8	144895492	G	T	Missense_Mutation	105	0.228571429	No	1	Clonal	Disease_causing	Passenger	Neutral	passenger	Yes	No	No	No	Not tested
MP1-2	Ductal	SCRIB	p.L186M	Shared	8	144895492	G	T	Missense_Mutation	154	0.318181818	No	1	Clonal	Disease_causing	Passenger	Neutral	passenger	Yes	No	No	No	Not tested
MP1-1	Chondroid	SLC25A29	p.C234F	Shared	14	100758831	C	A	Missense_Mutation	180	0.383333333	No	1	Clonal	Disease_causing	Driver	Neutral	likely_pathogenic	No	No	No	No	Not tested
MP1-2	Ductal	SLC25A29	p.C234F	Shared	14	100758831	C	A	Missense_Mutation	212	0.325471698	No	1	Clonal	Disease_causing	Driver	Neutral	likely_pathogenic	No	No	No	No	Not tested
MP1-1	Chondroid	SLC32A1	p.K164N	Shared	20	37356196	G	T	Missense_Mutation	352	0.258522727	No	1	Clonal	Disease_causing	Passenger	Neutral	passenger	Yes	No	No	No	Not tested
MP1-2	Ductal	SLC32A1	p.K164N	Shared	20	37356196	G	T	Missense_Mutation	412	0.242718447	No	1	Clonal	Disease_causing	Passenger	Neutral	passenger	Yes	No	No	No	Not tested
MP1-1	Chondroid	SPOCD1	p.A642G	Shared	1	32264146	G	C	Missense_Mutation	212	0.169811321	No	0.999	Clonal	Neutral	Passenger	Neutral	passenger	Yes	No	No	No	Not tested
MP1-2	Ductal	SPOCD1	p.A642G	Shared	1	32264146	G	C	Missense_Mutation	403	0.11662531	No	1	Clonal	Neutral	Passenger	Neutral	passenger	Yes	No	No	No	Not tested
MP1-1	Chondroid	SRRM5	p.Q341E	Shared	19	44117294	C	G	Missense_Mutation	263	0.326996198	No	1	Clonal	Neutral	Passenger	.	passenger	Yes	No	No	No	Not tested
MP1-2	Ductal	SRRM5	p.Q341E	Shared	19	44117294	C	G	Missense_Mutation	312	0.266025641	No	1	Clonal	Neutral	Passenger	.	passenger	Yes	No	No	No	Not tested
MP1-1	Chondroid	STAMBPL1	p.D360Y	Private	10	90681076	G	T	Missense_Mutation	442	0.255656109	No	1	Clonal	Disease_causing	Passenger	Disease_causing	likely_pathogenic	Yes	No	No	No	Not tested
MP1-1	Chondroid	STRA6	p.Q74H	Shared	15	74494504	C	G	Missense_Mutation	109	0.311926606	No	1	Clonal	Neutral	Passenger	Neutral	passenger	Yes	No	No	No	Not tested
MP1-2	Ductal	STRA6	p.Q74H	Shared	15	74494504	C	G	Missense_Mutation	182	0.258241758	No	1	Clonal	Neutral	Passenger	Neutral	passenger	Yes	No	No	No	Not tested
MP1-1	Chondroid	SYN3	p.D24G	Shared	22	33402577	T	C	Missense_Mutation	144	0.236111111	No	1	Clonal	Disease_causing	Passenger	Neutral	passenger	Yes	No	No	No	Not tested
MP1-2	Ductal	SYN3	p.D24G	Shared	22	33402577	T	C	Missense_Mutation	182	0.230769231	No	1	Clonal	Disease_causing	Passenger	Neutral	passenger	Yes	No	No	No	Not tested
MP1-1	Chondroid	SYT3	p.E191Q	Shared	19	51135646	C	G	Missense_Mutation	235	0.285106383	No	1	Clonal	Neutral	Passenger	Neutral	passenger	Yes	No	No	No	Not tested
MP1-2	Ductal	SYT3	p.E191Q	Shared	19	51135646	C	G	Missense_Mutation	249	0.224899598	No	1	Clonal	Neutral	Passenger	Neutral	passenger	Yes	No	No	No	Not tested
MP1-1	Chondroid	TAS2R38	p.R8C	Shared	7	141673468	G	A	Missense_Mutation	225	0.395555556	No	1	Clonal	Neutral	Passenger	Neutral	passenger	No	No	No	No	Not tested
MP1-2	Ductal	TAS2R38	p.R8C	Shared	7	141673468	G	A	Missense_Mutation	299	0.301003344	No	1	Clonal	Neutral	Passenger	Neutral	passenger	No	No	No	No	Not tested
MP1-1	Chondroid	TCEANC	p.R328T	Shared	X	13681520	G	C	Missense_Mutation	235	0.438297872	No	1	Clonal	Disease_causing	Passenger	Neutral	passenger	Yes	No	No	No	Not tested
MP1-2	Ductal	TCEANC	p.R328T	Shared	X	13681520	G	C	Missense_Mutation	369	0.27100271	No	1	Clonal	Disease_causing	Passenger	Neutral	passenger	Yes	No	No	No	Not tested
MP1-1	Chondroid	TGM1	p.E558Q	Shared	14	24724433	C	G	Missense_Mutation	260	0.323076923	No	1	Clonal	Disease_causing	Passenger	Disease_causing	likely_pathogenic	Yes	No	No	No	Not tested
MP1-2	Ductal	TGM1	p.E558Q	Shared	14	24724433	C	G	Missense_Mutation	293	0.269624573	No	1	Clonal	Disease_causing	Passenger	Disease_causing	likely_pathogenic	Yes	No	No	No	Not tested
MP1-1	Chondroid	THAP4	p.E537Q	Private	2	242541316	C	G	Missense_Mutation	114	0.043859649	No	0.302	Subclonal	Disease_causing	Passenger	Disease_causing	likely_pathogenic	No	No	No	No	Yes
MP1-1	Chondroid	THBS2	p.D1142_Y1145del	Shared	6	169620369	GTAGGTTTGGTCA	G	In_Frame_Del	112	0.214285714	No	1	Clonal	.	.	.	.	Yes	No	No	No	Not tested
MP1-2	Ductal	THBS2	p.D1142_Y1145del	Shared	6	169620369	GTAGGTTTGGTCA	G	In_Frame_Del	141	0.113475177	No	0.938	Clonal	.	.	.	.	Yes	No	No	No	Not tested
MP1-2	Ductal	TLR1	p.Q783L	Private	4	38798105	T	A	Missense_Mutation	395	0.037974684	No	0.314	Subclonal	Neutral	Passenger	Neutral	passenger	Yes	No	No	No	Yes
MP1-1	Chondroid	TNRC6C	p.N356K	Shared	17	76046211	T	A	Missense_Mutation	300	0.196666667	No	1	Clonal	Disease_causing	Passenger	Neutral	passenger	Yes	No	No	No	Not tested
MP1-2	Ductal	TNRC6C	p.N356K	Shared	17	76046211	T	A	Missense_Mutation	376	0.17287234	No	1	Clonal	Disease_causing	Passenger	Neutral	passenger	Yes	No	No	No	Not tested
MP1-1	Chondroid	TP53	p.R110P	Shared	17	7579358	C	G	Missense_Mutation	135	0.340740741	No	1	Clonal	Neutral	Passenger	Disease_causing	passenger	No	Yes	Yes	Yes	Yes
MP1-2	Ductal	TP53	p.R110P	Shared	17	7579358	C	G	Missense_Mutation	207	0.285024155	No	1	Clonal	Neutral	Passenger	Disease_causing	passenger	No	Yes	Yes	Yes	Yes
MP1-2	Ductal	TRPM1	p.S1400*	Private	15	31294755	G	C	Nonsense_Mutation	376	0.085106383	No	0.704	Subclonal	Disease_causing	.	.	passenger	Yes	No	No	No	Yes
MP1-1	Chondroid	TTBK2	p.G98C	Private	15	43122276	C	A	Missense_Mutation	65	0.230769231	No	1	Clonal	Disease_causing	Passenger	Neutral	passenger	Yes	No	No	No	Yes
MP1-1	Chondroid	TTC16	p.L124S	Shared	9	130479996	T	C	Missense_Mutation	139	0.517985612	No	1	Clonal	Neutral	Passenger	Neutral	passenger	No	No	No	No	Not tested
MP1-2	Ductal	TTC16	p.L124S	Shared	9	130479996	T	C	Missense_Mutation	178	0.286516854	No	1	Clonal	Neutral	Passenger	Neutral	passenger	No	No	No	No	Not tested
MP1-1	Chondroid	TTC31	p.G371A	Shared	2	74719523	G	C	Missense_Mutation	371	0.315363881	No	1	Clonal	Disease_causing	Passenger	Neutral	passenger	No	No	No	No	Not tested
MP1-2	Ductal	TTC31	p.G371A	Shared	2	74719523	G	C	Missense_Mutation	459	0.252723312	No	1	Clonal	Disease_causing	Passenger	Neutral	passenger	No	No	No	No	Not tested
MP1-1	Chondroid	USP27X	p.R131W	Shared	X	49645301	C	T	Missense_Mutation	522	0.08045977	No	0.634	Subclonal	.	Passenger	.	passenger	Yes	No	No	No	Not tested
MP1-2	Ductal	USP27X	p.R131W	Shared	X	49645301	C	T	Missense_Mutation	661	0.143721634	No	1	Subclonal	.	Passenger	.	passenger	Yes	No	No	No	Not tested
MP1-1	Chondroid	VAV1	p.D476H	Shared	19	6832129	G	C	Missense_Mutation	136	0.213235294	No	1	Clonal	Disease_causing	Passenger	Neutral	passenger	Yes	No	No	No	Not tested
MP1-2	Ductal	VAV1	p.D476H	Shared	19	6832129	G	C	Missense_Mutation	152	0.078947368	No	0.732	Clonal	Disease_causing	Passenger	Neutral	passenger	Yes	No	No	No	Not tested
MP1-1	Chondroid	VEGFA	p.R170G	Shared	6	43748554	C	G	Missense_Mutation	109	0.256880734	No	1	Clonal	Disease_causing	Passenger	.	passenger	Yes	No	No	No	Not tested
MP1-2	Ductal	VEGFA	p.R170G	Shared	6	43748554	C	G	Missense_Mutation	127	0.236220472	No	1	Clonal	Disease_causing	Passenger	.	passenger	Yes	No	No	No	Not tested
MP1-1	Chondroid	WHSC1	p.D1123H	Shared	4	1962873	G	C	Missense_Mutation	128	0.4609375	No	1	Clonal	Disease_causing	Passenger	Disease_causing	likely_pathogenic	No	Yes	No	No	Yes
MP1-2	Ductal	WHSC1	p.D1123H	Shared	4	1962873	G	C	Missense_Mutation	152	0.309210526	No	1	Clonal	Disease_causing	Passenger	Disease_causing	likely_pathogenic	No	Yes	No	No	Yes
MP1-2	Ductal	WNK4	p.P1042A	Private	17	40947744	C	G	Missense_Mutation	408	0.046568627	No	0.385	Subclonal	Neutral	Passenger	Neutral	passenger	Yes	No	No	No	Yes
MP1-1	Chondroid	ZC3H14	p.S330I	Shared	14	89041164	G	T	Missense_Mutation	153	0.431372549	No	1	Clonal	Disease_causing	Driver	.	likely_pathogenic	No	No	No	No	Not tested
MP1-2	Ductal	ZC3H14	p.S330I	Shared	14	89041164	G	T	Missense_Mutation	223	0.32735426	No	1	Clonal	Disease_causing	Driver	.	likely_pathogenic	No	No	No	No	Not tested
MP1-1	Chondroid	ZFP92	p.P319R	Shared	X	152686791	C	G	Missense_Mutation	185	0.205405405	No	1	Clonal	.	Passenger	.	passenger	Yes	No	No	No	Not tested
MP1-2	Ductal	ZFP92	p.P319R	Shared	X	152686791	C	G	Missense_Mutation	270	0.17037037	No	1	Clonal	.	Passenger	.	passenger	Yes	No	No	No	Not tested
MP1-1	Chondroid	ZFPM1	p.G850R	Shared	16	88600914	G	C	Missense_Mutation	69	0.304347826	No	1	Clonal	Neutral	Passenger	Neutral	passenger	Yes	No	No	No	Not tested
MP1-2	Ductal	ZFPM1	p.G850R	Shared	16	88600914	G	C	Missense_Mutation	105	0.257142857	No	1	Clonal	Neutral	Passenger	Neutral	passenger	Yes	No	No	No	Not tested
MP1-1	Chondroid	ZIC4	p.K181N	Shared	3	147113934	C	A	Missense_Mutation	982	0.176171079	No	1	Clonal	Disease_causing	Passenger	Neutral	passenger	No	No	No	No	Not tested
MP1-2	Ductal	ZIC4	p.K181N	Shared	3	147113934	C	A	Missense_Mutation	920	0.181521739	No	1	Clonal	Disease_causing	Passenger	Neutral	passenger	No	No	No	No	Not tested
MP1-1	Chondroid	ZNF41	p.E234*	Shared	X	47308469	C	A	Nonsense_Mutation	221	0.285067873	No	1	Clonal	Disease_causing	.	.	passenger	Yes	No	No	No	Not tested
MP1-2	Ductal	ZNF41	p.E234*	Shared	X	47308469	C	A	Nonsense_Mutation	290	0.182758621	No	1	Clonal	Disease_causing	.	.	passenger	Yes	No	No	No	Not tested
MP1-1	Chondroid	ZNF763	p.A391T	Shared	19	12089901	G	A	Missense_Mutation	165	0.4	No	1	Clonal	Neutral	Passenger	Neutral	passenger	Yes	No	No	No	Not tested
MP1-2	Ductal	ZNF763	p.A391T	Shared	19	12089901	G	A	Missense_Mutation	245	0.428571429	No	1	Clonal	Neutral	Passenger	Neutral	passenger	Yes	No	No	No	Not tested
MP1-1	Chondroid	ZNF853	p.E28*	Shared	7	6656890	G	T	Nonsense_Mutation	123	0.260162602	No	1	Clonal	.	.	.	passenger	Yes	No	No	No	Not tested
MP1-2	Ductal	ZNF853	p.E28*	Shared	7	6656890	G	T	Nonsense_Mutation	198	0.186868687	No	1	Clonal	.	.	.	passenger	Yes	No	No	No	Not tested
MP7-1	Spindle	ANKHD1	p.S2109_R2111del	Shared	5	139908854	CATCACCCAG	C	In_Frame_Del	182	0.153846154	No	0.68	Subclonal	.	.	.	.	No	No	No	No	Not tested
MP7-2	Squamous	ANKHD1	p.S2109_R2111del	Shared	5	139908854	CATCACCCAG	C	In_Frame_Del	172	0.156976744	No	0.932	Subclonal	.	.	.	.	No	No	No	No	Not tested
MP7-2	Squamous	APLP1	p.S235I	Private	19	36362792	G	T	Missense_Mutation	369	0.08401084	No	0.751	Subclonal	Neutral	Passenger	Disease_causing	passenger	No	No	No	No	Yes
MP7-1	Spindle	APOB	p.S2854A	Shared	2	21231180	A	C	Missense_Mutation	251	0.386454183	No	1	Clonal	Disease_causing	Passenger	Neutral	passenger	Yes	No	No	No	Not tested
MP7-2	Squamous	APOB	p.S2854A	Shared	2	21231180	A	C	Missense_Mutation	201	0.587064677	No	1	Clonal	Disease_causing	Passenger	Neutral	passenger	Yes	No	No	No	Not tested
MP7-1	Spindle	ATRN	p.L1291I	Shared	20	3614965	C	A	Missense_Mutation	99	0.292929293	No	1	Clonal	Disease_causing	Passenger	Neutral	passenger	No	No	No	No	Yes
MP7-2	Squamous	ATRN	p.L1291I	Shared	20	3614965	C	A	Missense_Mutation	98	0.408163265	No	1	Clonal	Disease_causing	Passenger	Neutral	passenger	No	No	No	No	Yes
MP7-1	Spindle	AUTS2	p.R1119Q	Shared	7	70255558	G	A	Missense_Mutation	350	0.351428571	No	1	Clonal	Disease_causing	Passenger	Neutral	passenger	No	No	No	No	Yes
MP7-2	Squamous	AUTS2	p.R1119Q	Shared	7	70255558	G	A	Missense_Mutation	336	0.401785714	No	1	Clonal	Disease_causing	Passenger	Neutral	passenger	No	No	No	No	Yes
MP7-1	Spindle	C7orf60	p.P229S	Shared	7	112462332	G	A	Missense_Mutation	95	0.263157895	No	1	Clonal	Disease_causing	Passenger	.	passenger	No	No	No	No	Yes
MP7-2	Squamous	C7orf60	p.P229S	Shared	7	112462332	G	A	Missense_Mutation	109	0.394495413	No	1	Clonal	Disease_causing	Passenger	.	passenger	No	No	No	No	Yes
MP7-1	Spindle	CASR	p.N468S	Shared	3	121994684	A	G	Missense_Mutation	142	0.309859155	No	1	Clonal	Disease_causing	Driver	Disease_causing	likely_pathogenic	Yes	No	No	No	Yes
MP7-2	Squamous	CASR	p.N468S	Shared	3	121994684	A	G	Missense_Mutation	168	0.541666667	No	1	Clonal	Disease_causing	Driver	Disease_causing	likely_pathogenic	Yes	No	No	No	Yes
MP7-2	Squamous	CEP250	p.V313Gfs*14	Private	20	34057798	CAG	C	Frame_Shift_Del	306	0.12745098	No	0.884	Subclonal	.	.	.	.	No	No	No	No	Not tested
MP7-1	Spindle	CLINT1	p.Q335*	Shared	5	157230667	G	A	Nonsense_Mutation	152	0.171052632	No	0.756	Clonal	Neutral	.	.	passenger	No	No	No	No	Not tested
MP7-2	Squamous	CLINT1	p.Q335*	Shared	5	157230667	G	A	Nonsense_Mutation	192	0.239583333	No	1	Clonal	Neutral	.	.	passenger	No	No	No	No	Not tested
MP7-2	Squamous	CLK2	p.R347*	Private	1	155234457	G	A	Nonsense_Mutation	178	0.039325843	No	0.233	Subclonal	Neutral	.	.	passenger	No	No	No	No	Not tested
MP7-2	Squamous	CPSF2	p.R306C	Private	14	92609414	C	T	Missense_Mutation	198	0.035353535	No	0.21	Subclonal	Disease_causing	Passenger	Neutral	passenger	No	No	No	No	Not tested
MP7-1	Spindle	DBX1	p.G12R	Shared	11	20181837	C	T	Missense_Mutation	174	0.172413793	No	0.935	Clonal	Neutral	Passenger	Neutral	passenger	No	No	No	No	Not tested
MP7-2	Squamous	DBX1	p.G12R	Shared	11	20181837	C	T	Missense_Mutation	203	0.216748768	No	1	Clonal	Neutral	Passenger	Neutral	passenger	No	No	No	No	Not tested
MP7-1	Spindle	DGCR14	p.Y43C	Shared	22	19132026	T	C	Missense_Mutation	106	0.132075472	No	0.584	Subclonal	Disease_causing	Driver	Disease_causing	likely_pathogenic	No	No	No	No	Not tested
MP7-2	Squamous	DGCR14	p.Y43C	Shared	22	19132026	T	C	Missense_Mutation	106	0.226415094	No	1	Subclonal	Disease_causing	Driver	Disease_causing	likely_pathogenic	No	No	No	No	Not tested
MP7-1	Spindle	DOK5	p.E206V	Shared	20	53226944	A	T	Missense_Mutation	174	0.25862069	No	1	Clonal	Disease_causing	Passenger	Disease_causing	likely_pathogenic	No	No	No	No	Yes
MP7-2	Squamous	DOK5	p.E206V	Shared	20	53226944	A	T	Missense_Mutation	179	0.346368715	No	1	Clonal	Disease_causing	Passenger	Disease_causing	likely_pathogenic	No	No	No	No	Yes
MP7-1	Spindle	ERN2	p.F772L	Shared	16	23703581	A	C	Missense_Mutation	163	0.067484663	No	0.231	Subclonal	Disease_causing	Driver	Neutral	likely_pathogenic	Yes	No	No	No	Not tested
MP7-2	Squamous	ERN2	p.F772L	Shared	16	23703581	A	C	Missense_Mutation	219	0.146118721	No	1	Subclonal	Disease_causing	Driver	Neutral	likely_pathogenic	No	No	No	No	Not tested
MP7-1	Spindle	FAM177A1	p.T114I	Shared	14	35546357	C	T	Missense_Mutation	292	0.092465753	No	0.409	Subclonal	Disease_causing	Passenger	.	passenger	No	No	No	No	Not tested
MP7-2	Squamous	FAM177A1	p.T114I	Shared	14	35546357	C	T	Missense_Mutation	233	0.188841202	No	1	Subclonal	Disease_causing	Passenger	.	passenger	No	No	No	No	Not tested
MP7-1	Spindle	FAM184A	p.S415T	Shared	6	119341232	A	T	Missense_Mutation	97	0.18556701	No	0.635	Subclonal	Neutral	Passenger	Neutral	passenger	Yes	No	No	No	Not tested
MP7-2	Squamous	FAM184A	p.S415T	Shared	6	119341232	A	T	Missense_Mutation	65	0.353846154	No	1	Subclonal	Neutral	Passenger	Neutral	passenger	Yes	No	No	No	Not tested

MP7-1	Spindle	FOXRED1	p.G114V	Shared	11	126142898	G	T	Missense_Mutation	[illegible]	[illegible]	No	1	Clonal	Disease_causing	Passenger	Neutral	passenger	No	No	No	No	Not tested
MP7-2	Squamous	FOXRED1	p.G114V	Shared	11	126142898	G	T	Missense_Mutation	247	[illegible]	No	1	Clonal	Disease_causing	Passenger	Neutral	passenger	No	No	No	No	Not tested
MP7-2	Squamous	GALNTL6	p.R197Q	Private	4	173730548	G	A	Missense_Mutation	137	0.04379562	No	0.216	Subclonal	Disease_causing	Passenger	Neutral	passenger	No	No	No	No	Yes
MP7-1	Spindle	GLB1L3	p.I158V	Private	11	134151959	A	G	Missense_Mutation	78	0.102564103	No	0.351	Subclonal	Disease_causing	Passenger	Disease_causing	likely_pathogenic	No	No	No	No	Not tested
MP7-1	Spindle	GLE1	p.K157R	Shared	9	131284984	A	G	Missense_Mutation	80	0.15	No	0.663	Subclonal	Disease_causing	Passenger	Neutral	passenger	No	No	No	No	Yes
MP7-2	Squamous	GLE1	p.K157R	Shared	9	131284984	A	G	Missense_Mutation	82	0.207317073	No	1	Subclonal	Disease_causing	Passenger	Neutral	passenger	No	No	No	No	Yes
MP7-1	Spindle	GOLGA1	p.A46D	Shared	9	127693684	G	T	Missense_Mutation	97	0.18556701	No	0.82	Clonal	Disease_causing	Driver	Neutral	likely_pathogenic	No	No	No	No	Yes
MP7-2	Squamous	GOLGA1	p.A46D	Shared	9	127693684	G	T	Missense_Mutation	83	0.21686747	No	1	Clonal	Disease_causing	Driver	Neutral	likely_pathogenic	No	No	No	No	Yes
MP7-1	Spindle	HAPLN1	p.L145*	Private	5	82948310	A	C	Nonsense_Mutation	159	0.056603774	No	0.25	Subclonal	Disease_causing	.	.	passenger	No	No	No	No	Yes
MP7-1	Spindle	IQSEC3	p.R349W	Private	12	247574	C	T	Missense_Mutation	235	0.059574468	No	0.204	Subclonal	Disease_causing	Passenger	Neutral	passenger	No	No	No	No	Not tested
MP7-1	Spindle	IRX1	p.T141M	Shared	5	3599484	C	T	Missense_Mutation	245	0.346938776	No	1	Clonal	Disease_causing	Passenger	Disease_causing	likely_pathogenic	No	No	No	No	Not tested
MP7-2	Squamous	IRX1	p.T141M	Shared	5	3599484	C	T	Missense_Mutation	224	0.5	No	1	Clonal	Disease_causing	Passenger	Disease_causing	likely_pathogenic	No	No	No	No	Not tested
MP7-1	Spindle	KCNAB3	p.R244Sfs*5	Shared	17	7827562	GT	G	Frame_Shift_Del	214	0.373831776	No	1	Clonal	.	.	.	.	Yes	No	No	No	Not tested
MP7-2	Squamous	KCNAB3	p.R244Sfs*5	Shared	17	7827562	GT	G	Frame_Shift_Del	171	0.608187135	No	1	Clonal	.	.	.	.	Yes	No	No	No	Not tested
MP7-1	Spindle	KIAA0556	p.E499K	Shared	16	27720131	G	A	Missense_Mutation	264	0.090909091	No	0.311	Subclonal	Neutral	Passenger	Neutral	passenger	No	No	No	No	Not tested
MP7-2	Squamous	KIAA0556	p.E499K	Shared	16	27720131	G	A	Missense_Mutation	344	0.325581395	No	1	Subclonal	Neutral	Passenger	Neutral	passenger	No	No	No	No	Not tested
MP7-1	Spindle	KIF4B	p.E576D	Shared	5	154395147	A	C	Missense_Mutation	185	0.135135135	No	0.597	Subclonal	Disease_causing	Passenger	Neutral	passenger	No	No	No	No	Yes
MP7-2	Squamous	KIF4B	p.E576D	Shared	5	154395147	A	C	Missense_Mutation	167	0.203592814	No	1	Subclonal	Disease_causing	Passenger	Neutral	passenger	No	No	No	No	Yes
MP7-1	Spindle	KIF5A	p.M300Vfs*10	Shared	12	57963115	CTA	C	Frame_Shift_Del	319	0.269592476	No	1	Clonal	.	.	.	.	No	No	No	No	Not tested
MP7-2	Squamous	KIF5A	p.M300Vfs*10	Shared	12	57963115	CTA	C	Frame_Shift_Del	316	0.389240506	No	1	Clonal	.	.	.	.	No	No	No	No	Not tested
MP7-1	Spindle	L1TD1	p.K588T	Shared	1	62676209	A	C	Missense_Mutation	193	0.14507772	No	0.496	Subclonal	Neutral	Passenger	Neutral	passenger	No	No	No	No	Not tested
MP7-2	Squamous	L1TD1	p.K588T	Shared	1	62676209	A	C	Missense_Mutation	201	0.268656716	No	1	Subclonal	Neutral	Passenger	Neutral	passenger	No	No	No	No	Not tested
MP7-1	Spindle	LDLRAD3	p.A269V	Private	11	36250715	C	T	Missense_Mutation	187	0.032085561	No	0.174	Subclonal	Neutral	Passenger	Disease_causing	passenger	No	No	No	No	Not tested
MP7-2	Squamous	LILRA4	p.G191C	Private	19	54849291	C	A	Missense_Mutation	103	0.174757282	No	1	Subclonal	Neutral	Passenger	Neutral	passenger	No	No	No	No	Yes
MP7-2	Squamous	LRP1B	p.T1714K	Private	2	141597628	G	T	Missense_Mutation	97	0.164948454	No	0.979	Subclonal	Disease_causing	Passenger	Disease_causing	likely_pathogenic	Yes	Yes	No	No	Yes
MP7-1	Spindle	LYST	p.A3799S	Private	1	235826251	C	A	Missense_Mutation	119	0.092436975	No	0.316	Subclonal	Disease_causing	Passenger	Neutral	passenger	Yes	No	No	No	Yes
MP7-2	Squamous	MED23	p.Y472S	Private	6	131926578	T	G	Missense_Mutation	62	0.193548387	No	0.956	Subclonal	Disease_causing	Driver	Neutral	likely_pathogenic	Yes	No	No	Yes	Yes
MP7-1	Spindle	METTL21A	p.Q115E	Shared	2	208478084	G	C	Missense_Mutation	209	0.133971292	No	0.726	Subclonal	Disease_causing	Passenger	Neutral	passenger	Yes	No	No	No	Yes
MP7-2	Squamous	METTL21A	p.Q115E	Shared	2	208478084	G	C	Missense_Mutation	147	0.292517007	No	1	Subclonal	Disease_causing	Passenger	Neutral	passenger	Yes	No	No	No	Yes
MP7-1	Spindle	MRPL17	p.Q160*	Shared	11	6703399	G	A	Nonsense_Mutation	140	0.257142857	No	1	Clonal	Disease_causing	.	.	passenger	No	No	No	No	Not tested
MP7-2	Squamous	MRPL17	p.Q160*	Shared	11	6703399	G	A	Nonsense_Mutation	141	0.375886525	No	1	Clonal	Disease_causing	.	.	passenger	No	No	No	No	Not tested
MP7-1	Spindle	NCOR1	p.S1855R	Shared	17	15965031	G	T	Missense_Mutation	183	0.426229508	No	1	Clonal	Disease_causing	Passenger	Neutral	passenger	Yes	Yes	Yes	Yes	Not tested
MP7-2	Squamous	NCOR1	p.S1855R	Shared	17	15965031	G	T	Missense_Mutation	135	0.755555556	No	1	Clonal	Disease_causing	Passenger	Neutral	passenger	Yes	Yes	Yes	Yes	Not tested
MP7-1	Spindle	NDUFA11	p.I135T	Shared	19	5893211	A	G	Missense_Mutation	235	0.05106383	No	0.277	Subclonal	Neutral	Passenger	Neutral	passenger	Yes	No	No	No	Not tested
MP7-2	Squamous	NDUFA11	p.I135T	Shared	19	5893211	A	G	Missense_Mutation	253	0.035573123	No	0.282	Subclonal	Neutral	Passenger	Neutral	passenger	Yes	No	No	No	Not tested
MP7-1	Spindle	NEB	p.R6723L	Shared	2	152404242	C	A	Missense_Mutation	122	0.139344262	No	0.755	Clonal	Disease_causing	Driver	Neutral	likely_pathogenic	Yes	No	No	No	Not tested
MP7-2	Squamous	NEB	p.R6723L	Shared	2	152404242	C	A	Missense_Mutation	121	0.090909091	No	0.54	Clonal	Disease_causing	Driver	Neutral	likely_pathogenic	Yes	No	No	No	Not tested
MP7-1	Spindle	NPFFR1	p.L399I	Shared	10	72014811	G	T	Missense_Mutation	143	0.251748252	No	0.861	Clonal	Neutral	Passenger	Neutral	passenger	Yes	No	No	No	Not tested
MP7-2	Squamous	NPFFR1	p.L399I	Shared	10	72014811	G	T	Missense_Mutation	126	0.357142857	No	1	Clonal	Neutral	Passenger	Neutral	passenger	Yes	No	No	No	Not tested
MP7-1	Spindle	NUP160	p.W778G	Private	11	47829991	A	C	Missense_Mutation	313	0.03514377	No	0.191	Subclonal	Disease_causing	Passenger	Neutral	passenger	No	No	No	No	Yes
MP7-1	Spindle	NUP210	p.R1871H	Shared	3	13359233	C	T	Missense_Mutation	153	0.209150327	No	1	Clonal	Disease_causing	Passenger	Neutral	passenger	Yes	No	No	No	Not tested
MP7-2	Squamous	NUP210	p.R1871H	Shared	3	13359233	C	T	Missense_Mutation	121	0.363636364	No	1	Clonal	Disease_causing	Passenger	Neutral	passenger	Yes	No	No	No	Not tested
MP7-1	Spindle	OR8D4	p.R53C	Shared	11	123777295	C	T	Missense_Mutation	181	0.26519337	No	1	Clonal	Neutral	Passenger	Neutral	passenger	No	No	No	No	Not tested
MP7-2	Squamous	OR8D4	p.R53C	Shared	11	123777295	C	T	Missense_Mutation	177	0.418079096	No	1	Clonal	Neutral	Passenger	Neutral	passenger	No	No	No	No	Not tested
MP7-1	Spindle	PARD3	p.R1088W	Shared	10	34558751	G	A	Missense_Mutation	217	0.428571429	No	1	Clonal	Disease_causing	Passenger	Neutral	passenger	Yes	No	No	No	Not tested
MP7-2	Squamous	PARD3	p.R1088W	Shared	10	34558751	G	A	Missense_Mutation	171	0.619883041	No	1	Clonal	Disease_causing	Passenger	Neutral	passenger	Yes	No	No	No	Not tested
MP7-1	Spindle	PIK3CA	p.H1047R	Shared	3	178952085	A	G	Missense_Mutation	180	0.466666667	Yes	1	Clonal	Disease_causing	Driver	Neutral	likely_pathogenic	Yes	Yes	Yes	Yes	Not tested
MP7-2	Squamous	PIK3CA	p.H1047R	Shared	3	178952085	A	G	Missense_Mutation	180	0.6	Yes	1	Clonal	Disease_causing	Driver	Neutral	likely_pathogenic	Yes	Yes	Yes	Yes	Not tested
MP7-1	Spindle	PKDREJ	p.V1137I	Shared	22	46655811	C	T	Missense_Mutation	162	0.228395062	No	1	Clonal	Neutral	Passenger	Neutral	passenger	No	No	No	No	Not tested
MP7-2	Squamous	PKDREJ	p.V1137I	Shared	22	46655811	C	T	Missense_Mutation	149	0.255033557	No	1	Clonal	Neutral	Passenger	Neutral	passenger	No	No	No	No	Not tested
MP7-2	Squamous	POLR3A	p.P532L	Private	10	79770276	G	A	Missense_Mutation	135	0.111111111	No	0.549	Subclonal	Disease_causing	Passenger	Disease_causing	likely_pathogenic	Yes	No	No	No	Not tested
MP7-2	Squamous	POMT1	p.E143K	Private	9	134382901	G	A	Missense_Mutation	90	0.055555556	No	0.33	Subclonal	Disease_causing	Passenger	Disease_causing	likely_pathogenic	No	No	No	No	Yes
MP7-2	Squamous	PRLH	p.A43T	Private	2	238475681	G	A	Missense_Mutation	95	0.052631579	No	0.312	Subclonal	Neutral	Passenger	.	passenger	Yes	No	No	No	Not tested
MP7-1	Spindle	PSPC1	p.E368Tfs*12	Shared	13	20304433	TTC	T	Frame_Shift_Del	122	0.360655738	No	1	Clonal	.	.	.	.	No	No	No	No	Not tested
MP7-2	Squamous	PSPC1	p.E368Tfs*12	Shared	13	20304433	TTC	T	Frame_Shift_Del	103	0.475728155	No	1	Clonal	.	.	.	.	No	No	No	No	Not tested
MP7-1	Spindle	PYCR1	p.R112Q	Private	17	79893007	C	T	Missense_Mutation	87	0.057471264	No	0.254	Subclonal	Neutral	Passenger	Neutral	passenger	Yes	No	No	No	Not tested
MP7-1	Spindle	RAB40C	p.A38T	Shared	16	640403	G	A	Missense_Mutation	165	0.072727273	No	0.249	Subclonal	Neutral	Passenger	Neutral	passenger	Yes	No	No	No	Not tested
MP7-2	Squamous	RAB40C	p.A38T	Shared	16	640403	G	A	Missense_Mutation	173	0.086705202	No	0.515	Subclonal	Neutral	Passenger	Neutral	passenger	No	No	No	No	Not tested
MP7-1	Spindle	SGK223	p.S743R	Shared	8	8197079	G	T	Missense_Mutation	162	0.154320988	No	0.682	Subclonal	Disease_causing	Passenger	Neutral	passenger	No	No	No	No	Not tested
MP7-2	Squamous	SGK223	p.S743R	Shared	8	8197079	G	T	Missense_Mutation	157	0.203821656	No	1	Subclonal	Disease_causing	Passenger	Neutral	passenger	No	No	No	No	Not tested
MP7-1	Spindle	SH3RF3	p.G863E	Shared	2	110259187	G	A	Missense_Mutation	456	0.307017544	No	1	Clonal	Disease_causing	Passenger	Neutral	passenger	Yes	No	No	No	Not tested
MP7-2	Squamous	SH3RF3	p.G863E	Shared	2	110259187	G	A	Missense_Mutation	343	0.565597668	No	1	Clonal	Disease_causing	Passenger	Neutral	passenger	Yes	No	No	No	Not tested
MP7-2	Squamous	SIN3A	p.R1134W	Private	15	75668197	G	A	Missense_Mutation	96	0.052083333	No	0.309	Subclonal	Disease_causing	Passenger	Neutral	passenger	No	No	Yes	No	Yes
MP7-1	Spindle	SLCO4C1	p.P694A	Shared	5	101572657	G	C	Missense_Mutation	108	0.083333333	No	0.368	Subclonal	Neutral	Passenger	Neutral	passenger	No	No	No	No	Not tested
MP7-2	Squamous	SLCO4C1	p.P694A	Shared	5	101572657	G	C	Missense_Mutation	89	0.202247191	No	1	Subclonal	Neutral	Passenger	Neutral	passenger	No	No	No	No	Not tested
MP7-1	Spindle	SPAST	p.K290N	Shared	2	32339894	G	T	Missense_Mutation	140	0.085714286	No	0.55	Subclonal	Disease_causing	Passenger	Neutral	passenger	Yes	No	No	No	Not tested
MP7-2	Squamous	SPAST	p.K290N	Shared	2	32339894	G	T	Missense_Mutation	114	0.219298246	No	1	Subclonal	Disease_causing	Passenger	Neutral	passenger	Yes	No	No	No	Not tested
MP7-1	Spindle	SPRED3	p.R296C	Shared	19	38886438	C	T	Missense_Mutation	104	0.5	No	1	Clonal	Disease_causing	Passenger	Neutral	passenger	No	No	No	No	Not tested
MP7-2	Squamous	SPRED3	p.R296C	Shared	19	38886438	C	T	Missense_Mutation	147	0.646258503	No	1	Clonal	Disease_causing	Passenger	Neutral	passenger	No	No	No	No	Not tested
MP7-1	Spindle	SRA1	p.V53A	Shared	5	139936761	A	G	Missense_Mutation	301	0.355481728	No	1	Clonal	Disease_causing	Passenger	Neutral	passenger	No	No	No	No	Not tested
MP7-2	Squamous	SRA1	p.V53A	Shared	5	139936761	A	G	Missense_Mutation	249	0.530120482	No	1	Clonal	Disease_causing	Passenger	Neutral	passenger	No	No	No	No	Not tested
MP7-1	Spindle	STAB1	p.G1393E	Shared	3	52550405	G	A	Missense_Mutation	224	0.151785714	No	0.823	Clonal	Disease_causing	Passenger	Disease_causing	likely_pathogenic	Yes	No	No	No	Not tested
MP7-2	Squamous	STAB1	p.G1393E	Shared	3	52550405	G	A	Missense_Mutation	170	0.311764706	No	1	Clonal	Disease_causing	Passenger	Disease_causing	likely_pathogenic	Yes	No	No	No	Not tested
MP7-1	Spindle	TLE4	p.C53Wfs*25	Shared	9	82189802	GT	G	Frame_Shift_Del	129	0.108527132	No	0.48	Subclonal	.	.	.	.	No	No	No	No	Not tested
MP7-2	Squamous	TLE4	p.C53Wfs*25	Shared	9	82189802	GT	G	Frame_Shift_Del	98	0.306122449	No	1	Subclonal	.	.	.	.	No	No	No	No	Not tested
MP7-1	Spindle	TMCO3	p.L481V	Shared	13	114188457	C	G	Missense_Mutation	171	0.099415205	No	0.34	Subclonal	Disease_causing	Passenger	Neutral	passenger	Yes	No	No	No	Not tested
MP7-2	Squamous	TMCO3	p.L481V	Shared	13	114188457	C	G	Missense_Mutation	156	0.147435897	No	0.728	Subclonal	Disease_causing	Passenger	Neutral	passenger	Yes	No	No	No	Not tested
MP7-1	Spindle	TMEM132B	p.Q977K	Shared	12	126138948	C	A	Missense_Mutation	104	0.307692308	No	1	Clonal	Disease_causing	Passenger	Neutral	passenger	No	No	No	No	Not tested
MP7-2	Squamous	TMEM132B	p.Q977K	Shared	12	126138948	C	A	Missense_Mutation	115	0.408695652	No	1	Clonal	Disease_causing	Passenger	Neutral	passenger	No	No	No	No	Not tested
MP7-1	Spindle	TMEM180	p.L143M	Shared	10	104230597	C	A	Missense_Mutation	459	0.407407407	No	1	Clonal	Disease_causing	Passenger	Disease_causing	likely_pathogenic	Yes	No	No	No	Not tested
MP7-2	Squamous	TMEM180	p.L143M	Shared	10	104230597	C	A	Missense_Mutation	364	0.664835165	No	1	Clonal	Disease_causing	Passenger	Disease_causing	likely_pathogenic	Yes	No	No	No	Not tested
MP7-1	Spindle	TOPBP1	p.S1274del	Shared	3	133335705	AGAG	A	In_Frame_Del	53	0.245283019	No	1	Clonal	.	.	.	.	Yes	No	No	No	Not tested
MP7-2	Squamous	TOPBP1	p.S1274del	Shared	3	133335705	AGAG	A	In_Frame_Del	53	0.226415094	No	1	Clonal	.	.	.	.	Yes	No	No	No	Not tested
MP7-1	Spindle	TP53	p.C135Afs*35	Shared	17	7578526	CA	C	Frame_Shift_Del	239	0.384937238	No	1	Clonal	.	.	.	.	Yes	Yes	Yes	Yes	Yes
MP7-2	Squamous	TP53	p.C135Afs*35	Shared	17	7578526	CA	C	Frame_Shift_Del	188	0.632978723	No	1	Clonal	.	.	.	.	Yes	Yes	Yes	Yes	Yes
MP7-1	Spindle	TTC7A	p.K227N	Shared	2	47205963	A	T	Missense_Mutation	367	0.155313351	No	0.842	Clonal	Disease_causing	Passenger	Neutral	passenger	Yes	No	No	No	Not tested
MP7-2	Squamous	TTC7A	p.K227N	Shared	2	47205963	A	T	Missense_Mutation	261	0.279693487	No	1	Clonal	Disease_causing	Passenger	Neutral	passenger	Yes	No	No	No	Not tested
MP7-1	Spindle	TUBGCP2	p.K219E	Shared	10	135110870	T	C	Missense_Mutation	85	0.117647059	No	0.402	Subclonal	Neutral	Passenger	Neutral	passenger	Yes	No	No	No	Not tested
MP7-2	Squamous	TUBGCP2	p.K219E	Shared	10	135110870	T	C	Missense_Mutation	86	0.104651163	No	0.517	Subclonal	Neutral	Passenger	Neutral	passenger	Yes	No	No	No	Not tested
MP7-1	Spindle	ZNF579	p.E237K	Shared	19	56090297	C	T	Missense_Mutation	445	0.229213483	No	1	Clonal	Neutral	Passenger	Neutral	passenger	No	No	No	No	Not tested
MP7-2	Squamous	ZNF579	p.E237K	Shared	19	56090297	C	T	Missense_Mutation	400	0.29	No	1	Clonal	Neutral	Passenger	Neutral	passenger	No	No	No	No	Not tested
MP7-1	Spindle	ZNF587B	p.S191I	Shared	19	58352614	G	T	Missense_Mutation	78	0.230769231	No	1	Clonal	.	Passenger	Neutral	passenger	No	No	No	No	Not tested
MP7-2	Squamous	ZNF587B	p.S191I	Shared	19	58352614	G	T	Missense_Mutation	71	0.436619718	No	1	Clonal	.	Passenger	Neutral	passenger	No	No	No	No	Not tested
MP7-1	Spindle	ZNF629	p.D601H	Shared	16	30793848	C	G	Missense_Mutation	387	0.03875969	No	0.133	Subclonal	Neutral	Passenger	Neutral	passenger	Yes	No	No	No	Not tested
MP7-2	Squamous	ZNF629	p.E648D	Private	16	30793705	C	G	Missense_Mutation	269	0.148698885	No	1	Subclonal	Neutral	Passenger	Neutral	passenger	No	No	No	No	Not tested
MP7-2	Squamous	ZNF629	p.D601H	Shared	16	30793848	C	G	Missense_Mutation	397	0.153652393	No	1	Subclonal	Neutral	Passenger	Neutral	passenger	No	No	No	No	Not tested
MP7-1	Spindle	ZNF865	p.R272H	Shared	19	56125799	G	A	Missense_Mutation	87	0.252873563	No	1	Clonal	.	Passenger	.	passenger	No	No	No	No	Not tested
MP7-2	Squamous	ZNF865	p.R272H	Shared	19	56125799	G	A	Missense_Mutation	76	0.368421053	No	1	Clonal	.	Passenger	.	passenger	No	No	No	No	Not tested
MP8-2	Ductal	ABCA4	p.T867S	Private	1	94517243	T	A	Missense_Mutation	76	0.131578947	No	0.838	Subclonal	Neutral	Passenger	Disease_causing	passenger	No	No	No	No	Yes
MP8-2	Ductal	ABCA8	p.S203L	Private	17	66928618	G	A	Missense_Mutation	78	0.064102564	No	0.408	Subclonal	Disease_causing	Passenger	Disease_causing	likely_pathogenic	Yes	No	No	No	Yes
MP8-1	Spindle	ABL1	p.G678S	Shared	9	133759709	G	A	Missense_Mutation	179	0.441340782	No	1	Clonal	Neutral	Passenger	Neutral	passenger	Yes	Yes	No	No	Not tested
MP8-2	Ductal	ABL1	p.G678S	Shared	9	133759709	G	A	Missense_Mutation	251	0.163346614	No	1	Clonal	Neutral	Passenger	Neutral	passenger	Yes	Yes	No	No	Not tested
MP8-1	Spindle	ACTA1	p.V154M	Shared	1	229568173	C	T	Missense_Mutation	257	0.361867704	No	1	Clonal	Disease_causing	Passenger	Disease_causing	likely_pathogenic	Yes	No	No	No	Not tested
MP8-2	Ductal	ACTA1	p.V154M	Shared	1	229568173	C	T	Missense_Mutation	339	0.398230088	No	1	Clonal	Disease_causing	Passenger	Disease_causing	likely_pathogenic	Yes	No	No	No	Not tested
MP8-2	Ductal	ADCYAP1R1	p.T119M	Private	7	31123783	C	T	Missense_Mutation	142	0.063380282	No	0.34	Subclonal	Disease_causing	Passenger	Neutral	passenger	No	No	No	No	Not tested
MP8-1	Spindle	AGBL3	p.S863P	Shared	7	134819837	T	C	Missense_Mutation	88	0.227272727	No	1	Clonal	Neutral	Passenger	Neutral	passenger	No	No	No	No	Not tested
MP8-2	Ductal	AGBL3	p.S863P	Shared	7	134819837	T	C	Missense_Mutation	79	0.227848101	No	1	Clonal	Neutral	Passenger	Neutral	passenger	No	No	No	No	Not tested
MP8-1	Spindle	ARHGAP10	p.X84_splice	Private	4	148743974	G	C	Splice_Site	254	0.153543307	No	0.904	Clonal	Disease_causing	.	.	passenger	No	No	No	No	Not tested
MP8-2	Ductal	ARNTL2	p.D625N	Private	12	27573427	G	A	Missense_Mutation	112	0.107142857	No	0.575	Subclonal	Disease_causing	Passenger	Neutral	passenger	No	No	No	No	Yes
MP8-2	Ductal	ASXL1	p.K523N	Private	20	31021570	G	C	Missense_Mutation	203	0.064039409	No	0.408	Subclonal	Disease_causing	Passenger	Neutral	passenger	No	Yes	Yes	Yes	Yes
MP8-1	Spindle	ATP11B	p.S788*	Shared	3	182597394	C	G	Nonsense_Mutation	61	0.360655738	No	1	Clonal	Disease_causing	.	.	passenger	Yes	No	No	No	Not tested
MP8-2	Ductal	ATP11B	p.S788*	Shared	3	182597394	C	G	Nonsense_Mutation	74	0.216216216	No	1	Clonal	Disease_causing	.	.	passenger	Yes	No	No	No	Not tested
MP8-1	Spindle	ATP6AP1	p.A386S	Private	X	153663804	G	T	Missense_Mutation	241	0.082987552	No	0.488	Subclonal	Neutral	Passenger	.	passenger	No	No	No	No	Yes
MP8-2	Ductal	BAHD1	p.E660K	Private	15	40756672	G	A	Missense_Mutation	102	0.176470588	No	0.948	Subclonal	Disease_causing	Passenger	Disease_causing	likely_pathogenic	Yes	No	No	No	Yes
MP8-1	Spindle	BBX	p.L58F	Shared	3	107435463	C	T	Missense_Mutation	74	0.22972973	No	1	Clonal	Disease_causing	Passenger	Disease_causing	likely_pathogenic	Yes	No	No	No	Not tested
MP8-2	Ductal	BBX	p.L58F	Shared	3	107435463	C	T	Missense_Mutation	118	0.093220339	No	0.687	Clonal	Disease_causing	Passenger	Disease_causing	likely_pathogenic	Yes	No	No	No	Not tested
MP8-2	Ductal	C1RL	p.G465R	Private	12	7249058	C	G	Missense_Mutation	517	0.040618956	No	0.381	Subclonal	Disease_causing	Passenger	Disease_causing	likely_pathogenic	No	No	No	No	Yes
MP8-1	Spindle	C22orf26	p.N132K	Private	22	46447728	A	T	Missense_Mutation	71	0.070422535	No	0.414	Subclonal	Neutral	Passenger	Neutral	passenger	No	No	No	No	Not tested
MP8-2	Ductal	CAD	p.G132A	Private	2	27445104	G	C	Missense_Mutation	257	0.06614786	No	0.554	Subclonal	Disease_causing	Passenger	Disease_causing	likely_pathogenic	Yes	No	No	No	Yes
MP8-1	Spindle	CAMSAP3	p.L424F	Shared	19	7676468	C	T	Missense_Mutation	213	0.248826291	No	1	Clonal	Disease_causing	Passenger	Neutral	passenger	No	No	No	No	Yes
MP8-2	Ductal	CAMSAP3	p.L424F	Shared	19	7676468	C	T	Missense_Mutation	250	0.224	No	1	Clonal	Disease_causing	Passenger	Neutral	passenger	No	No	No	No	Yes
MP8-1	Spindle	CATSPER1	p.A90T	Shared	11	65793583	C	T	Missense_Mutation	248	0.314516129	No	1	Clonal	Neutral	Passenger	Disease_causing	passenger	No	No	No	No	Not tested
MP8-2	Ductal	CATSPER1	p.A90T	Shared	11	65793583	C	T	Missense_Mutation	297	0.208754209	No	1	Clonal	Neutral	Passenger	Disease_causing	passenger	No	No	No	No	Not tested
MP8-1	Spindle	CDC14A	p.A415V	Private	1	100960480	C	T	Missense_Mutation	125	0.104	No	0.612	Subclonal	Disease_causing	Passenger	Neutral	passenger	No	No	No	No	Yes
MP8-1	Spindle	CDC14A	p.A415P	Private	1	100960479	G	C	Missense_Mutation	126	0.103174603	No	0.607	Subclonal	Disease_causing	Passenger	Neutral	passenger	No	No	No	No	Yes
MP8-1	Spindle	CNKSR2	p.A798T	Shared	X	21627435	G	A	Missense_Mutation	205	0.243902439	No	1	Clonal	Neutral	Passenger	Neutral	passenger	Yes	No	No	No	Not tested
MP8-2	Ductal	CNKSR2	p.A798T	Shared	X	21627435	G	A	Missense_Mutation	289	0.200692042	No	0.877	Clonal	Neutral	Passenger	Neutral	passenger	Yes	No	No	No	Not tested
MP8-1	Spindle	COPZ1	p.D74H	Private	12	54737059	G	C	Missense_Mutation	181	0.215469613	No	1	Clonal	Disease_causing	Passenger	.	passenger	Yes	No	No	No	Yes
MP8-2	Ductal	CRAMP1L	p.A500T	Private	16	1706256	G	A	Missense_Mutation	230	0.173913043	No	0.934	Subclonal	Neutral	Passenger	.	passenger	Yes	No	No	No	Not tested
MP8-1	Spindle	CRTC2	p.P341L	Private	1	153924118	G	A	Missense_Mutation	76	0.065789474	No	0.453	Subclonal	Disease_causing	Passenger	Neutral	passenger	No	No	No	No	Yes
MP8-1	Spindle	CSMD2	p.P1217R	Private	1	34164508	G	C	Missense_Mutation	113	0.088495575	No	0.521	Subclonal	Disease_causing	Passenger	Neutral	passenger	No	No	No	No	Yes
MP8-2	Ductal	CTBP1	p.E326K	Private	4	1207311	C	T	Missense_Mutation	193	0.03626943	No	0.158	Subclonal	Disease_causing	Passenger	Disease_causing	likely_pathogenic	Yes	No	No	No	Yes
MP8-1	Spindle	CTCF	p.P696Qfs*24	Private	16	67671675	AGCCC	A	Frame_Shift_Del	185	0.243243243	No	1	Clonal	.	.	.	.	No	Yes	Yes	Yes	Yes
MP8-1	Spindle	CYLC1	p.T152S	Private	X	83128170	A	T	Missense_Mutation	58	0.172413793	No	1	Clonal	Neutral	Passenger	Neutral	passenger	No	No	No	No	Yes
MP8-1	Spindle	DAND5	p.V158M	Shared	19	13084350	G	A	Missense_Mutation	462	0.279220779	No	1	Clonal	Neutral	Passenger	Neutral	passenger	No	No	No	No	Not tested
MP8-2	Ductal	DAND5	p.V158M	Shared	19	13084350	G	A	Missense_Mutation	480	0.179166667	No	1	Clonal	Neutral	Passenger	Neutral	passenger	No	No	No	No	Not tested
MP8-1	Spindle	DDX20	p.D772H	Shared	1	112309360	G	C	Missense_Mutation	96	0.28125	No	1	Clonal	Disease_causing	Passenger	Neutral	passenger	No	No	No	No	Not tested
MP8-2	Ductal	DDX20	p.D772H	Shared	1	112309360	G	C	Missense_Mutation	126	0.126984127	No	0.809	Clonal	Disease_causing	Passenger	Neutral	passenger	No	No	No	No	Not tested
MP8-1	Spindle	DGKB	p.C339W	Private	7	14722196	A	C	Missense_Mutation	85	0.152941176	No	0.9	Clonal	Disease_causing	Driver	Disease_causing	likely_pathogenic	No	No	No	No	Yes
MP8-1	Spindle	DNASE1L1	p.L29R	Private	X	153633824	A	C	Missense_Mutation	264	0.181818182	No	1	Clonal	Neutral	Passenger	Neutral	passenger	No	No	No	No	Yes
MP8-1	Spindle	DOCK11	p.R1348Q	Private	X	117773439	G	A	Missense_Mutation	93	0.053763441	No	0.37	Subclonal	Disease_causing	Passenger	Neutral	passenger	No	No	No	No	Not tested
MP8-2	Ductal	DUSP27	p.A439T	Private	1	167095683	G	A	Missense_Mutation	311	0.144694534	No	0.777	Subclonal	Neutral	Passenger	Neutral	passenger	Yes	No	No	No	Not tested
MP8-2	Ductal	EEF1D	p.K646N	Private	8	144661968	C	G	Missense_Mutation	539	0.031539889	No	0.327	Subclonal	Disease_causing	Passenger	.	passenger	No	No	No	No	Not tested
MP8-2	Ductal	EEF2	p.C369*	Private	19	3980882	G	T	Nonsense_Mutation	188	0.122340426	No	0.902	Subclonal	Neutral	.	.	passenger	No	No	No	No	Not tested
MP8-1	Spindle	ELN	p.G57R	Shared	7	73452042	G	C	Missense_Mutation	124	0.258064516	No	1	Clonal	Neutral	Passenger	Neutral	passenger	No	No	No	No	Not tested
MP8-2	Ductal	ELN	p.G57R	Shared	7	73452042	G	C	Missense_Mutation	126	0.253968254	No	1	Clonal	Neutral	Passenger	Neutral	passenger	No	No	No	No	Not tested
MP8-2	Ductal	FAM220A	p.G6R	Private	7	6370770	C	G	Missense_Mutation	199	0.050251256	No	0.27	Subclonal	Neutral	Passenger	Neutral	passenger	No	No	No	No	Yes
MP8-1	Spindle	FBRS	p.H610L	Shared	16	30678265	A	T	Missense_Mutation	111	0.333333333	No	1	Clonal	Disease_causing	Passenger	Neutral	passenger	No	No	No	No	Not tested
MP8-2	Ductal	FBRS	p.H610L	Shared	16	30678265	A	T	Missense_Mutation	95	0.157894737	No	1	Clonal	Disease_causing	Passenger	Neutral	passenger	No	No	No	No	Not tested
MP8-1	Spindle	FBXL16	p.C115G	Shared	16	747063	A	C	Missense_Mutation	158	0.512658228	No	1	Clonal	Disease_causing	Passenger	Neutral	passenger	Yes	No	No	No	Yes
MP8-2	Ductal	FBXL16	p.C115G	Shared	16	747063	A	C	Missense_Mutation	180	0.266666667	No	1	Clonal	Disease_causing	Passenger	Neutral	passenger	Yes	No	No	No	Yes
MP8-1	Spindle	FRMPD3	p.Q1764_Q1765ins*	Private	X	106846459	A	ACAGTAG	Nonsense_Mutation	152	0.197368421	No	1	Clonal	.	.	.	.	No	No	No	No	Not tested
MP8-1	Spindle	GLDN	p.P252T	Shared	15	51689732	C	A	Missense_Mutation	91	0.384615385	No	1	Clonal	Neutral	Passenger	Disease_causing	passenger	Yes	No	No	No	Not tested
MP8-2	Ductal	GLDN	p.P252T	Shared	15	51689732	C	A	Missense_Mutation	97	0.134020619	No	0.72	Clonal	Neutral	Passenger	Disease_causing	passenger	Yes	No	No	No	Not tested
MP8-2	Ductal	GLI2	p.S863L	Private	2	121746078	C	T	Missense_Mutation	95	0.052631579	No	0.388	Subclonal	Disease_causing	Passenger	Disease_causing	likely_pathogenic	Yes	No	No	No	Yes
MP8-1	Spindle	GRIN2C	p.E134K	Shared	17	72848750	C	T	Missense_Mutation	82	0.243902439	No	1	Clonal	Disease_causing	Passenger	Disease_causing	likely_pathogenic	Yes	No	No	No	Not tested
MP8-2	Ductal	GRIN2C	p.E134K	Shared	17	72848750	C	T	Missense_Mutation	124	0.177419355	No	1	Clonal	Disease_causing	Passenger	Disease_causing	likely_pathogenic	Yes	No	No	No	Not tested
MP8-1	Spindle	HDAC7	p.D306E	Shared	12	48189539	G	T	Missense_Mutation	98	0.244897959	No	1	Clonal	Neutral	Passenger	Neutral	passenger	No	No	No	No	Not tested
MP8-2	Ductal	HDAC7	p.D306E	Shared	12	48189539	G	T	Missense_Mutation	122	0.245901639	No	1	Clonal	Neutral	Passenger	Neutral	passenger	No	No	No	No	Not tested
MP8-2	Ductal	HDC	p.Y322C	Private	15	50544703	T	C	Missense_Mutation	231	0.177489177	No	0.953	Subclonal	Disease_causing	Passenger	Neutral	passenger	Yes	No	No	No	Not tested
MP8-1	Spindle	HHLA3	p.K97*	Private	1	70833334	A	T	Nonsense_Mutation	67	0.074626866	No	0.439	Subclonal	Disease_causing	.	.	passenger	No	No	No	No	Not tested
MP8-1	Spindle	HMCN1	p.Q4802K	Private	1	186114672	C	A	Missense_Mutation	102	0.225490196	No	1	Clonal	Disease_causing	Passenger	Neutral	passenger	Yes	No	No	No	Not tested
MP8-1	Spindle	HPN	p.L171F	Shared	19	35551309	G	C	Missense_Mutation	274	0.437956204	No	1	Clonal	Disease_causing	Passenger	Disease_causing	likely_pathogenic	Yes	No	No	No	Yes
MP8-2	Ductal	HPN	p.L171F	Shared	19	35551309	G	C	Missense_Mutation	376	0.295212766	No	1	Clonal	Disease_causing	Passenger	Disease_causing	likely_pathogenic	Yes	No	No	No	Yes
MP8-1	Spindle	IL6	p.I151M	Shared	7	22769261	C	G	Missense_Mutation	151	0.357615894	No	1	Clonal	Neutral	Passenger	Neutral	passenger	No	No	No	No	Not tested
MP8-2	Ductal	IL6	p.I151M	Shared	7	22769261	C	G	Missense_Mutation	187	0.144385027	No	0.775	Clonal	Neutral	Passenger	Neutral	passenger	No	No	No	No	Not tested
MP8-1	Spindle	INSRR	p.E1030V	Private	1	156812833	T	A	Missense_Mutation	196	0.091836735	No	0.632	Subclonal	Disease_causing	Driver	Disease_causing	likely_pathogenic	No	No	No	No	Yes
MP8-1	Spindle	ITIH5	p.P49H	Shared	10	7684043	G	T	Missense_Mutation	62	0.096774194	No	0.666	Subclonal	Disease_causing	Passenger	Neutral	passenger	No	No	No	No	Not tested
MP8-2	Ductal	ITIH5	p.P49H	Shared	10	7684043	G	T	Missense_Mutation	68	0.147058824	No	0.937	Subclonal	Disease_causing	Passenger	Neutral	passenger	No	No	No	No	Not tested
MP8-1	Spindle	KDM2A	p.K310*	Shared	11	66986845	A	T	Nonsense_Mutation	87	0.275862069	No	1	Clonal	Disease_causing	.	.	passenger	No	No	No	No	Not tested
MP8-2	Ductal	KDM2A	p.K310*	Shared	11	66986845	A	T	Nonsense_Mutation	109	0.165137615	No	1	Clonal	Disease_causing	.	.	passenger	No	No	No	No	Not tested
MP8-2	Ductal	KIAA1551	p.C1172W	Private	12	32137405	C	G	Missense_Mutation	127	0.070866142	No	0.381	Subclonal	Disease_causing	Passenger	Neutral	passenger	No	No	No	No	Yes
MP8-1	Spindle	LAMA5	p.R3550P	Shared	20	60885319	C	G	Missense_Mutation	136	0.301470588	No	1	Clonal	Disease_causing	Passenger	Disease_causing	likely_pathogenic	No	No	No	No	Not tested
MP8-2	Ductal	LAMA5	p.R3550P	Shared	20	60885319	C	G	Missense_Mutation	167	0.143712575	No	0.915	Clonal	Disease_causing	Passenger	Disease_causing	likely_pathogenic	No	No	No	No	Not tested
MP8-1	Spindle	LLGL1	p.P178A	Shared	17	18137231	C	G	Missense_Mutation	202	0.103960396	No	0.508	Subclonal	Disease_causing	Passenger	Neutral	passenger	Yes	No	No	No	Not tested
MP8-2	Ductal	LLGL1	p.P178A	Shared	17	18137231	C	G	Missense_Mutation	287	0.202090592	No	1	Subclonal	Disease_causing	Passenger	Neutral	passenger	Yes	No	No	No	Not tested
MP8-1	Spindle	MAB21L1	p.E67D	Shared	13	36050075	C	G	Missense_Mutation	124	0.362903226	No	1	Clonal	Disease_causing	Passenger	Neutral	passenger	Yes	No	No	No	Not tested
MP8-2	Ductal	MAB21L1	p.E67D	Shared	13	36050075	C	G	Missense_Mutation	170	0.176470588	No	0.948	Clonal	Disease_causing	Passenger	Neutral	passenger	Yes	No	No	No	Not tested
MP8-1	Spindle	MAMDC2	p.X141_splice	Shared	9	72724635	G	C	Splice_Site	112	0.285714286	No	1	Clonal	Disease_causing	.	.	likely_pathogenic	Yes	No	No	No	Not tested
MP8-2	Ductal	MAMDC2	p.X141_splice	Shared	9	72724635	G	C	Splice_Site	156	0.25	No	1	Clonal	Disease_causing	.	.	likely_pathogenic	Yes	No	No	No	Not tested
MP8-1	Spindle	MAP1S	p.G251R	Shared	19	17836944	G	A	Missense_Mutation	275	0.276363636	No	1	Clonal	Disease_causing	Passenger	Neutral	passenger	No	No	No	No	Yes
MP8-2	Ductal	MAP1S	p.G251R	Shared	19	17836944	G	A	Missense_Mutation	373	0.18230563	No	1	Clonal	Disease_causing	Passenger	Neutral	passenger	No	No	No	No	Yes
MP8-1	Spindle	MFSD10	p.C9Y	Private	4	2935625	C	T	Missense_Mutation	95	0.052631579	No	0.257	Subclonal	Neutral	Passenger	Neutral	passenger	Yes	No	No	No	Yes
MP8-1	Spindle	MTUS2	p.G850E	Shared	13	29674982	G	A	Missense_Mutation	101	0.455445545	No	1	Clonal	Disease_causing	Passenger	Neutral	passenger	Yes	No	No	No	Not tested
MP8-2	Ductal	MTUS2	p.G850E	Shared	13	29674982	G	A	Missense_Mutation	117	0.230769231	No	1	Clonal	Disease_causing	Passenger	Neutral	passenger	Yes	No	No	No	Not tested
MP8-2	Ductal	MUC16	p.S13209N	Private	19	9006392	C	T	Missense_Mutation	236	0.072033898	No	0.531	Subclonal	.	Passenger	Neutral	passenger	Yes	No	No	No	Not tested
MP8-1	Spindle	MUM1	p.G323_S327del	Private	19	1360881	GCAGGGGCCGCACCAT	G	In_Frame_Del	138	0.123188406	No	0.848	Clonal	.	.	.	.	No	No	No	No	Not tested
MP8-2	Ductal	MYBL1	p.Q732E	Private	8	67476997	G	C	Missense_Mutation	272	0.080882353	No	0.596	Subclonal	Disease_causing	Passenger	Neutral	passenger	No	No	No	No	Not tested
MP8-1	Spindle	MYO18B	p.S71P	Shared	22	26164094	T	C	Missense_Mutation	238	0.243697479	No	1	Clonal	Neutral	Passenger	Disease_causing	passenger	No	No	No	No	Not tested
MP8-2	Ductal	MYO18B	p.S71P	Shared	22	26164094	T	C	Missense_Mutation	286	0.143356643	No	0.913	Clonal	Neutral	Passenger	Disease_causing	passenger	No	No	No	No	Not tested
MP8-2	Ductal	MYO5C	p.S630C	Private	15	52539205	T	A	Missense_Mutation	60	0.2	No	1	Subclonal	Disease_causing	Passenger	Neutral	passenger	Yes	No	No	No	Yes
MP8-1	Spindle	NLRP1	p.A438S	Shared	17	5462704	C	A	Missense_Mutation	144	0.416666667	No	1	Clonal	Neutral	Passenger	Neutral	passenger	Yes	No	No	No	Not tested
MP8-2	Ductal	NLRP1	p.A438S	Shared	17	5462704	C	A	Missense_Mutation	190	0.289473684	No	1	Clonal	Neutral	Passenger	Neutral	passenger	Yes	No	No	No	Not tested
MP8-1	Spindle	NPC1L1	p.Q161R	Private	7	44579514	T	C	Missense_Mutation	130	0.046153846	No	0.272	Subclonal	Neutral	Passenger	Disease_causing	passenger	No	No	No	No	Yes
MP8-2	Ductal	NR2F6	p.E329D	Private	19	17343389	C	G	Missense_Mutation	90	0.2	No	1	Subclonal	Disease_causing	Passenger	Disease_causing	likely_pathogenic	No	No	No	No	Yes
MP8-1	Spindle	OR10S1	p.A238_A245del	Shared	11	123847664	GGCTGTGCGGATGCGCAACACAGCT	G	In_Frame_Del	89	0.146067416	No	0.714	Subclonal	.	.	.	.	Yes	No	No	No	Not tested
MP8-2	Ductal	OR10S1	p.A238_A245del	Shared	11	123847664	GGCTGTGCGGATGCGCAACACAGCT	G	In_Frame_Del	140	0.092857143	No	0.406	Subclonal	.	.	.	.	Yes	No	No	No	Not tested
MP8-1	Spindle	OR3A3	p.F286S	Shared	17	3324718	T	C	Missense_Mutation	89	0.359550562	No	1	Clonal	Neutral	Passenger	Neutral	passenger	Yes	No	No	No	Not tested
MP8-2	Ductal	OR3A3	p.F286S	Shared	17	3324718	T	C	Missense_Mutation	121	0.148760331	No	0.799	Clonal	Neutral	Passenger	Neutral	passenger	Yes	No	No	No	Not tested
MP8-1	Spindle	OR5M11	p.Y126C	Shared	11	56310357	T	C	Missense_Mutation	126	0.222222222	No	1	Clonal	Neutral	Passenger	Neutral	passenger	No	No	No	No	Not tested
MP8-2	Ductal	OR5M11	p.Y126C	Shared	11	56310357	T	C	Missense_Mutation	150	0.206666667	No	1	Clonal	Neutral	Passenger	Neutral	passenger	No	No	No	No	Not tested
MP8-2	Ductal	OSBPL2	p.A438P	Private	20	60866821	G	C	Missense_Mutation	113	0.150442478	No	0.958	Subclonal	Disease_causing	Passenger	Neutral	passenger	No	No	No	No	Not tested
MP8-2	Ductal	PDE4DIP	p.Q646E	Private	1	144915489	G	C	Missense_Mutation	379	0.031662269	No	0.233	Subclonal	Disease_causing	Passenger	Neutral	passenger	No	Yes	No	No	Yes
MP8-1	Spindle	PDGFRL	p.P188R	Shared	8	17486053	C	G	Missense_Mutation	201	0.328358209	No	1	Clonal	Disease_causing	Passenger	Neutral	passenger	Yes	No	No	No	Not tested
MP8-2	Ductal	PDGFRL	p.P188R	Shared	8	17486053	C	G	Missense_Mutation	275	0.232727273	No	1	Clonal	Disease_causing	Passenger	Neutral	passenger	Yes	No	No	No	Not tested
MP8-1	Spindle	PEX11B	p.E117G	Shared	1	145518248	A	G	Missense_Mutation	74	0.175675676	No	1	Clonal	Disease_causing	Passenger	Neutral	passenger	No	No	No	No	Not tested
MP8-2	Ductal	PEX11B	p.E117G	Shared	1	145518248	A	G	Missense_Mutation	90	0.133333333	No	0.983	Clonal	Disease_causing	Passenger	Neutral	passenger	No	No	No	No	Not tested
MP8-1	Spindle	PIGA	p.S330T	Shared	X	15342986	C	G	Missense_Mutation	94	0.29787234	No	1	Clonal	Disease_causing	Passenger	Neutral	passenger	Yes	No	No	No	Yes
MP8-2	Ductal	PIGA	p.S330T	Shared	X	15342986	C	G	Missense_Mutation	107	0.196261682	No	0.858	Clonal	Disease_causing	Passenger	Neutral	passenger	Yes	No	No	No	Yes
MP8-2	Ductal	PKHD1	p.C2965R	Private	6	51618056	A	G	Missense_Mutation	160	0.05625	No	0.358	Subclonal	Disease_causing	Passenger	Disease_causing	likely_pathogenic	No	No	No	No	Not tested
MP8-1	Spindle	PLA2G4B	p.V188M	Shared	15	42128378	G	A	Missense_Mutation	60	0.416666667	No	1	Clonal	Disease_causing	Passenger	Neutral	passenger	Yes	No	No	No	Not tested
MP8-2	Ductal	PLA2G4B	p.V188M	Shared	15	42128378	G	A	Missense_Mutation	69	0.188405797	No	1	Clonal	Disease_causing	Passenger	Neutral	passenger	Yes	No	No	No	Not tested
MP8-1	Spindle	PLA2G4F	p.F145L	Private	15	42446305	G	C	Missense_Mutation	64	0.375	No	1	Clonal	Neutral	Passenger	Neutral	passenger	Yes	No	No	No	Yes
MP8-2	Ductal	PTDSS1	p.L113V	Private	8	97299270	C	G	Missense_Mutation	211	0.037914692	No	0.279	Subclonal	Disease_causing	Passenger	Neutral	passenger	No	No	No	No	Yes
MP8-2	Ductal	PTOV1	p.K210N	Private	19	50360303	G	C	Missense_Mutation	178	0.050561798	No	0.373	Subclonal	Disease_causing	Passenger	.	passenger	Yes	No	No	No	Yes
MP8-1	Spindle	R3HDM1	p.G64A	Shared	2	136373741	G	C	Missense_Mutation	85	0.2	No	1	Clonal	Disease_causing	Passenger	Neutral	passenger	Yes	No	No	No	Not tested
MP8-2	Ductal	R3HDM1	p.G64A	Shared	2	136373741	G	C	Missense_Mutation	114	0.192982456	No	1	Clonal	Disease_causing	Passenger	Neutral	passenger	Yes	No	No	No	Not tested
MP8-2	Ductal	RGS22	p.R405T	Private	8	101075782	C	G	Missense_Mutation	227	0.04845815	No	0.357	Subclonal	Neutral	Passenger	Neutral	passenger	No	No	No	No	Yes
MP8-1	Spindle	RIMS1	p.R1316C	Private	6	73017056	C	T	Missense_Mutation	81	0.320987654	No	1	Clonal	Disease_causing	Passenger	Neutral	passenger	No	No	No	No	Yes
MP8-1	Spindle	RNF39	p.A377T	Shared	6	30039022	C	T	Missense_Mutation	154	0.292207792	No	1	Clonal	Neutral	Passenger	Neutral	passenger	No	No	No	No	Yes
MP8-2	Ductal	RNF39	p.A377T	Shared	6	30039022	C	T	Missense_Mutation	190	0.157894737	No	0.848	Clonal	Neutral	Passenger	Neutral	passenger	No	No	No	No	Yes
MP8-1	Spindle	ROBO3	p.T623N	Shared	11	124744049	C	A	Missense_Mutation	96	0.447916667	No	1	Clonal	Disease_causing	Passenger	Neutral	passenger	Yes	No	No	No	Not tested
MP8-2	Ductal	ROBO3	p.T623N	Shared	11	124744049	C	A	Missense_Mutation	132	0.204545455	No	0.894	Clonal	Disease_causing	Passenger	Neutral	passenger	Yes	No	No	No	Not tested
MP8-1	Spindle	SCRIB	p.G1191S	Shared	8	144885585	C	T	Missense_Mutation	278	0.151079137	No	1	Clonal	Disease_causing	Passenger	Neutral	passenger	No	No	No	No	Yes
MP8-2	Ductal	SCRIB	p.G1191S	Shared	8	144885585	C	T	Missense_Mutation	261	0.183908046	No	1	Clonal	Disease_causing	Passenger	Neutral	passenger	No	No	No	No	Yes
MP8-1	Spindle	SH3TC1	p.W705*	Shared	4	8229535	G	A	Nonsense_Mutation	185	0.367567568	No	1	Clonal	Neutral	.	.	likely_pathogenic	Yes	No	No	No	Not tested
MP8-2	Ductal	SH3TC1	p.W705*	Shared	4	8229535	G	A	Nonsense_Mutation	295	0.240677966	No	1	Clonal	Neutral	.	.	likely_pathogenic	Yes	No	No	No	Not tested
MP8-2	Ductal	SLC22A6	p.V428L	Private	11	62747039	C	G	Missense_Mutation	273	0.036630037	No	0.233	Subclonal	Disease_causing	Passenger	Neutral	passenger	No	No	No	No	Yes
MP8-1	Spindle	SLC25A32	p.E24Q	Shared	8	104427096	C	G	Missense_Mutation	147	0.272108844	No	1	Clonal	Disease_causing	Passenger	Neutral	passenger	No	No	No	No	Not tested
MP8-2	Ductal	SLC25A32	p.E24Q	Shared	8	104427096	C	G	Missense_Mutation	263	0.136882129	No	1	Clonal	Disease_causing	Passenger	Neutral	passenger	No	No	No	No	Not tested
MP8-1	Spindle	SPTBN4	p.V1951I	Shared	19	41066245	G	A	Missense_Mutation	289	0.397923875	No	1	Clonal	Disease_causing	Passenger	Neutral	passenger	Yes	No	No	No	Yes
MP8-2	Ductal	SPTBN4	p.V1951I	Shared	19	41066245	G	A	Missense_Mutation	477	0.255765199	No	1	Clonal	Disease_causing	Passenger	Neutral	passenger	Yes	No	No	No	Yes
MP8-1	Spindle	SRC	p.H25Y	Shared	20	36012629	C	T	Missense_Mutation	394	0.30964467	No	1	Clonal	Neutral	Passenger	Neutral	passenger	No	Yes	No	No	Not tested
MP8-2	Ductal	SRC	p.H25Y	Shared	20	36012629	C	T	Missense_Mutation	364	0.211538462	No	1	Clonal	Neutral	Passenger	Neutral	passenger	No	Yes	No	No	Not tested
MP8-1	Spindle	STK31	p.T766K	Shared	7	23826149	C	A	Missense_Mutation	100	0.31	No	1	Clonal	Disease_causing	Passenger	Neutral	passenger	No	No	No	No	Not tested
MP8-2	Ductal	STK31	p.T766K	Shared	7	23826149	C	A	Missense_Mutation	132	0.174242424	No	0.936	Clonal	Disease_causing	Passenger	Neutral	passenger	No	No	No	No	Not tested
MP8-2	Ductal	SVIL	p.D219Y	Private	10	29839698	C	A	Missense_Mutation	301	0.122923588	No	0.783	Subclonal	Disease_causing	Passenger	Neutral	passenger	Yes	No	No	No	Not tested
MP8-1	Spindle	SYNE1	p.S4438R	Private	6	152652506	A	T	Missense_Mutation	216	0.222222222	No	1	Clonal	Disease_causing	Passenger	Neutral	passenger	No	No	No	No	Yes
MP8-1	Spindle	SYNE1	p.L1268Q	Private	6	152765580	A	T	Missense_Mutation	55	0.290909091	No	1	Clonal	Disease_causing	Passenger	Neutral	passenger	No	No	No	No	Yes
MP8-1	Spindle	TAF6L	p.P227L	Private	11	62549658	C	T	Missense_Mutation	249	0.200803213	No	1	Clonal	Disease_causing	Passenger	Neutral	passenger	No	No	No	No	Not tested
MP8-1	Spindle	TENM1	p.W248C	Shared	X	123870839	C	G	Missense_Mutation	166	0.385542169	No	1	Clonal	Disease_causing	Passenger	Neutral	passenger	Yes	No	No	No	Yes
MP8-2	Ductal	TENM1	p.W248C	Shared	X	123870839	C	G	Missense_Mutation	113	0.283185841	No	1	Clonal	Disease_causing	Passenger	Neutral	passenger	Yes	No	No	No	Yes
MP8-1	Spindle	THSD7B	p.I1408L	Private	2	138414473	A	C	Missense_Mutation	120	0.05	No	0.344	Subclonal	Disease_causing	Passenger	Neutral	passenger	Yes	No	No	No	Not tested
MP8-2	Ductal	TNFRSF21	p.H485Q	Private	6	47221046	G	T	Missense_Mutation	127	0.062992126	No	0.401	Subclonal	Disease_causing	Passenger	Disease_causing	likely_pathogenic	No	No	No	No	Yes
MP8-1	Spindle	TP53	p.R248Q	Shared	17	7577538	C	T	Missense_Mutation	85	0.4	No	1	Clonal	Neutral	Passenger	Disease_causing	likely_pathogenic	Yes	Yes	Yes	Yes	Yes
MP8-2	Ductal	TP53	p.R248Q	Shared	17	7577538	C	T	Missense_Mutation	120	0.216666667	No	1	Clonal	Neutral	Passenger	Disease_causing	likely_pathogenic	Yes	Yes	Yes	Yes	Yes
MP8-1	Spindle	TRAPPC10	p.A55T	Shared	21	45457686	G	A	Missense_Mutation	112	0.142857143	No	0.841	Clonal	Disease_causing	Passenger	Neutral	passenger	No	No	No	No	Not tested
MP8-2	Ductal	TRAPPC10	p.A55T	Shared	21	45457686	G	A	Missense_Mutation	151	0.152317881	No	0.818	Clonal	Disease_causing	Passenger	Neutral	passenger	No	No	No	No	Not tested
MP8-1	Spindle	TRAPPC8	p.K447N	Shared	18	29487471	C	G	Missense_Mutation	84	0.30952381	No	1	Clonal	Disease_causing	Passenger	Neutral	passenger	No	No	No	No	Not tested
MP8-2	Ductal	TRAPPC8	p.K447N	Shared	18	29487471	C	G	Missense_Mutation	155	0.077419355	No	0.493	Clonal	Disease_causing	Passenger	Neutral	passenger	No	No	No	No	Not tested
MP8-1	Spindle	ZNF227	p.E222K	Shared	19	44739247	G	A	Missense_Mutation	97	0.556701031	No	1	Clonal	Neutral	Passenger	Neutral	passenger	Yes	No	No	No	Not tested
MP8-2	Ductal	ZNF227	p.E222K	Shared	19	44739247	G	A	Missense_Mutation	138	0.391304348	No	1	Clonal	Neutral	Passenger	Neutral	passenger	Yes	No	No	No	Not tested
MP8-1	Spindle	ZNF423	p.E823*	Shared	16	49670596	C	A	Nonsense_Mutation	130	0.253846154	No	1	Clonal	Neutral	.	.	passenger	No	No	No	No	Not tested
MP8-2	Ductal	ZNF423	p.E823*	Shared	16	49670596	C	A	Nonsense_Mutation	155	0.122580645	No	0.781	Clonal	Neutral	.	.	passenger	No	No	No	No	Not tested
MP8-2	Ductal	ZNF451	p.Q369K	Private	6	57011988	C	A	Missense_Mutation	233	0.141630901	No	0.902	Subclonal	Disease_causing	Passenger	Neutral	passenger	No	No	No	No	Not tested
MP8-1	Spindle	ZNF740	p.X125_splice	Shared	12	53580174	G	C	Splice_Site	71	0.535211268	No	1	Clonal	Disease_causing	.	.	passenger	Yes	No	No	No	Not tested
MP8-2	Ductal	ZNF740	p.X125_splice	Shared	12	53580174	G	C	Splice_Site	83	0.265060241	No	1	Clonal	Disease_causing	.	.	passenger	Yes	No	No	No	Not tested
MP8-2	Ductal	ZNF827	p.E23*	Private	4	146824344	C	A	Nonsense_Mutation	149	0.060402685	No	0.264	Subclonal	Neutral	.	.	passenger	Yes	No	No	No	Yes
MP11-B1	Chondroid	5-Sep	p.K373Q	Private	X	118763444	T	G	Missense_Mutation	89	0.101123596	No	0.43	Subclonal	Disease_causing	Passenger	Disease_causing	likely_pathogenic	No	No	No	No	Not tested
MP11-A1	Squamous	ABCA13	p.L3667H	Shared	7	48411961	T	A	Missense_Mutation	236	0.241525424	No	1	Clonal	Disease_causing	Passenger	Disease_causing	likely_pathogenic	Yes	No	No	No	Not tested
MP11-A2	Squamous	ABCA13	p.L3667H	Shared	7	48411961	T	A	Missense_Mutation	213	0.17370892	No	1	Clonal	Disease_causing	Passenger	Disease_causing	likely_pathogenic	Yes	No	No	No	Not tested
MP11-B1	Chondroid	ABCA13	p.L3667H	Shared	7	48411961	T	A	Missense_Mutation	172	0.319767442	No	1	Clonal	Disease_causing	Passenger	Disease_causing	likely_pathogenic	No	No	No	No	Not tested

MP11-A2	Squamous	ABCA9	p.E175Q	Private	17	67040688	C	G	Missense_Mutation	[illegible]	[illegible]	No	0.782	Subclonal	Neutral	Passenger	Disease_causing	passenger	No	No	No	No	Yes
MP11-B1	Chondroid	ABCB4	p.V439A	Private	7	87072675	A	G	Missense_Mutation	166	[illegible]	No	0.655	Subclonal	Disease_causing	Passenger	Disease_causing	likely_pathogenic	Yes	No	No	No	Yes
MP11-B1	Chondroid	ABCC11	p.R135H	Private	16	48258332	C	T	Missense_Mutation	237	0.143459916	No	0.753	Subclonal	Neutral	Passenger	Disease_causing	passenger	Yes	No	No	No	Not tested
MP11-B1	Chondroid	ABHD2	p.P38R	Private	15	89659671	C	G	Missense_Mutation	266	0.236842105	No	1	Subclonal	Disease_causing	Passenger	Neutral	passenger	No	No	No	No	Yes
MP11-B1	Chondroid	ABL2	p.E551D	Private	1	179079589	C	G	Missense_Mutation	264	0.102272727	No	0.742	Subclonal	Disease_causing	Passenger	Neutral	passenger	Yes	Yes	No	No	Not tested
MP11-B1	Chondroid	ADAMTS5	p.A202G	Private	21	28338106	G	C	Missense_Mutation	234	0.123931624	No	0.775	Subclonal	Disease_causing	Passenger	Neutral	passenger	Yes	No	No	No	Yes
MP11-B1	Chondroid	ADARB1	p.L67Pfs*11	Private	21	46595810	ACCGCCTGAAGAAAAGGAGGAAAACACCAGGGC	A	Frame_Shift_Del	174	0.109195402	No	0.464	Subclonal	.	.	.	.	Yes	No	No	No	Yes
MP11-B1	Chondroid	ADCY10	p.R983Q	Private	1	167814860	C	T	Missense_Mutation	269	0.100371747	No	0.728	Subclonal	Neutral	Passenger	Neutral	passenger	Yes	No	No	No	Not tested
MP11-A1	Squamous	ADCY2	p.D222G	Shared	5	7626374	A	G	Missense_Mutation	268	0.145522388	No	1	Clonal	Disease_causing	Passenger	Neutral	passenger	Yes	No	No	No	Not tested
MP11-A2	Squamous	ADCY2	p.D222G	Shared	5	7626374	A	G	Missense_Mutation	212	0.183962264	No	1	Clonal	Disease_causing	Passenger	Neutral	passenger	Yes	No	No	No	Not tested
MP11-B1	Chondroid	ADNP	NA	Private	20	49520539	C	G	Splice_Site	286	0.083916084	No	0.692	Subclonal	.	.	.	likely_pathogenic	No	No	No	Yes	Yes
MP11-A1	Squamous	AHSG	p.R340C	Shared	3	186338633	C	T	Missense_Mutation	542	0.077490775	No	0.843	Clonal	Neutral	Passenger	Neutral	passenger	No	No	No	No	Not tested
MP11-A2	Squamous	AHSG	p.R340C	Shared	3	186338633	C	T	Missense_Mutation	393	0.081424936	No	1	Clonal	Neutral	Passenger	Neutral	passenger	No	No	No	No	Not tested
MP11-A1	Squamous	AKNA	p.P75R	Shared	9	117143390	G	C	Missense_Mutation	254	0.220472441	No	1	Clonal	Neutral	Passenger	Neutral	passenger	Yes	No	No	No	Not tested
MP11-A2	Squamous	AKNA	p.P75R	Shared	9	117143390	G	C	Missense_Mutation	191	0.167539267	No	1	Clonal	Neutral	Passenger	Neutral	passenger	Yes	No	No	No	Not tested
MP11-B1	Chondroid	AKNA	p.P75R	Shared	9	117143390	G	C	Missense_Mutation	204	0.269607843	No	1	Clonal	Neutral	Passenger	Neutral	passenger	Yes	No	No	No	Not tested
MP11-B1	Chondroid	ALS2CR11	p.C231Y	Private	2	202438993	C	T	Missense_Mutation	212	0.127358491	No	0.924	Subclonal	Disease_causing	Driver	Neutral	likely_pathogenic	No	No	No	No	Yes
MP11-A1	Squamous	ANKRD1	p.P288S	Shared	10	92672721	G	A	Missense_Mutation	297	0.262626263	No	1	Clonal	Disease_causing	Passenger	Neutral	passenger	Yes	No	No	No	Not tested
MP11-A2	Squamous	ANKRD1	p.P288S	Shared	10	92672721	G	A	Missense_Mutation	295	0.186440678	No	1	Clonal	Disease_causing	Passenger	Neutral	passenger	Yes	No	No	No	Not tested
MP11-A2	Squamous	AR	p.S697P	Private	X	66931447	T	C	Missense_Mutation	136	0.051470588	No	0.664	Subclonal	Disease_causing	Passenger	Disease_causing	likely_pathogenic	No	Yes	Yes	No	Yes
MP11-B1	Chondroid	ARCN1	p.S244A	Private	11	118455271	T	G	Missense_Mutation	178	0.393258427	No	1	Subclonal	Disease_causing	Passenger	Neutral	passenger	Yes	No	No	No	Yes
MP11-A2	Squamous	ASB2	p.Q354H	Private	14	94406009	C	G	Missense_Mutation	65	0.076923077	No	1	Subclonal	Neutral	Passenger	Neutral	passenger	No	No	No	No	Yes
MP11-A2	Squamous	ATF6B	p.R588G	Private	6	32084516	G	C	Missense_Mutation	185	0.037837838	No	0.564	Subclonal	Disease_causing	Passenger	Neutral	passenger	Yes	No	No	No	Not tested
MP11-A1	Squamous	ATP10A	p.D1486E	Shared	15	25924530	G	T	Missense_Mutation	260	0.265384615	No	1	Clonal	Neutral	Passenger	Neutral	passenger	Yes	No	No	No	Not tested
MP11-A2	Squamous	ATP10A	p.D1486E	Shared	15	25924530	G	T	Missense_Mutation	189	0.174603175	No	1	Clonal	Neutral	Passenger	Neutral	passenger	Yes	No	No	No	Not tested
MP11-B1	Chondroid	ATP1A4	p.M822V	Private	1	160146034	A	G	Missense_Mutation	431	0.11136891	No	0.808	Subclonal	Disease_causing	Passenger	Disease_causing	likely_pathogenic	Yes	No	No	No	Yes
MP11-B1	Chondroid	AUH	p.R226G	Private	9	93983254	G	C	Missense_Mutation	82	0.146341463	No	1	Subclonal	Disease_causing	Passenger	Neutral	passenger	Yes	No	No	No	Yes
MP11-A1	Squamous	AXIN1	p.S359R	Shared	16	360012	G	C	Missense_Mutation	101	0.217821782	No	1	Clonal	Disease_causing	Passenger	Disease_causing	likely_pathogenic	No	Yes	No	No	Yes
MP11-A2	Squamous	AXIN1	p.S359R	Shared	16	360012	G	C	Missense_Mutation	72	0.180555556	No	1	Clonal	Disease_causing	Passenger	Disease_causing	likely_pathogenic	No	Yes	No	No	Yes
MP11-B1	Chondroid	AXIN1	p.S359R	Shared	16	360012	G	C	Missense_Mutation	82	0.158536585	No	0.516	Clonal	Disease_causing	Passenger	Disease_causing	likely_pathogenic	No	Yes	No	No	Yes
MP11-B1	Chondroid	BANK1	p.T619A	Private	4	102951377	A	G	Missense_Mutation	242	0.037190083	No	0.232	Subclonal	Neutral	Passenger	Neutral	passenger	No	No	No	No	Yes
MP11-B1	Chondroid	BIRC6	p.M3824L	Private	2	32743441	A	C	Missense_Mutation	322	0.052795031	No	0.436	Subclonal	Disease_causing	Passenger	Neutral	passenger	No	No	No	No	Yes
MP11-B1	Chondroid	C17orf103	p.K70Rfs*105	Private	17	21147434	G	GGCGA	Frame_Shift_Ins	135	0.148148148	No	0.778	Subclonal	.	.	.	.	No	No	No	No	Not tested
MP11-A1	Squamous	C1orf177	p.R370Q	Shared	1	55282720	G	A	Missense_Mutation	271	0.147601476	No	1	Clonal	Neutral	Passenger	Neutral	passenger	Yes	No	No	No	Not tested
MP11-A2	Squamous	C1orf177	p.R370Q	Shared	1	55282720	G	A	Missense_Mutation	188	0.095744681	No	1	Clonal	Neutral	Passenger	Neutral	passenger	No	No	No	No	Not tested
MP11-A1	Squamous	C1orf228	p.S410T	Shared	1	45190981	G	C	Missense_Mutation	463	0.239740821	No	1	Clonal	Neutral	Passenger	.	passenger	Yes	No	No	No	Not tested
MP11-A2	Squamous	C1orf228	p.S410T	Shared	1	45190981	G	C	Missense_Mutation	283	0.144876325	No	1	Clonal	Neutral	Passenger	.	passenger	No	No	No	No	Not tested
MP11-B1	Chondroid	C1orf228	p.S410T	Shared	1	45190981	G	C	Missense_Mutation	329	0.246200608	No	1	Clonal	Neutral	Passenger	.	passenger	Yes	No	No	No	Not tested
MP11-A2	Squamous	C2orf68	p.D128Y	Private	2	85836187	C	A	Missense_Mutation	198	0.03030303	No	0.512	Subclonal	Disease_causing	Passenger	.	passenger	No	No	No	No	Not tested
MP11-B1	Chondroid	C5orf34	p.E523A	Private	5	43492329	T	G	Missense_Mutation	307	0.09771987	No	0.611	Subclonal	Neutral	Passenger	Neutral	passenger	No	No	No	No	Yes
MP11-A1	Squamous	C6orf62	NA	Shared	6	24706360	GTACTC	G	Missense_Mutation	301	0.215946844	No	1	Clonal	.	.	.	.	Yes	No	No	No	Not tested
MP11-A2	Squamous	C6orf62	NA	Shared	6	24706360	GTACTC	G	Missense_Mutation	313	0.095846645	No	1	Clonal	.	.	.	.	Yes	No	No	No	Not tested
MP11-B1	Chondroid	CACNA1E	p.E880G	Private	1	181701861	A	G	Missense_Mutation	336	0.119047619	No	0.863	Subclonal	Disease_causing	Passenger	Disease_causing	likely_pathogenic	Yes	No	No	No	Not tested
MP11-B1	Chondroid	CAMTA1	p.Y548D	Private	1	7724249	T	G	Missense_Mutation	231	0.064935065	No	0.341	Subclonal	Neutral	Passenger	Neutral	passenger	Yes	Yes	No	No	Yes
MP11-A1	Squamous	CAPN12	p.T378S	Shared	19	39228025	G	C	Missense_Mutation	296	0.290540541	No	1	Clonal	Disease_causing	Passenger	Disease_causing	likely_pathogenic	Yes	No	No	No	Not tested
MP11-A2	Squamous	CAPN12	p.T378S	Shared	19	39228025	G	C	Missense_Mutation	188	0.143617021	No	1	Clonal	Disease_causing	Passenger	Disease_causing	likely_pathogenic	Yes	No	No	No	Not tested
MP11-A1	Squamous	CARD11	p.N781K	Shared	7	2959173	G	T	Missense_Mutation	243	0.333333333	No	1	Clonal	Disease_causing	Passenger	Neutral	passenger	No	Yes	No	Yes	Yes
MP11-A2	Squamous	CARD11	p.N781K	Shared	7	2959173	G	T	Missense_Mutation	249	0.248995984	No	1	Clonal	Disease_causing	Passenger	Neutral	passenger	No	Yes	No	Yes	Yes
MP11-B1	Chondroid	CARD11	p.N781K	Shared	7	2959173	G	T	Missense_Mutation	234	0.230769231	No	0.75	Clonal	Disease_causing	Passenger	Neutral	passenger	No	Yes	No	Yes	Yes
MP11-B1	Chondroid	CCDC27	p.E516G	Private	1	3683813	A	G	Missense_Mutation	155	0.122580645	No	0.644	Subclonal	Neutral	Passenger	Neutral	passenger	Yes	No	No	No	Yes
MP11-B1	Chondroid	CCT8	p.D548N	Private	21	30428801	C	T	Missense_Mutation	238	0.088235294	No	0.552	Subclonal	Disease_causing	Passenger	Neutral	passenger	Yes	No	No	No	Yes
MP11-B1	Chondroid	CD6	p.I406L	Private	11	60780960	A	C	Missense_Mutation	184	0.10326087	No	0.852	Subclonal	Neutral	Passenger	Neutral	passenger	Yes	No	No	No	Yes
MP11-A2	Squamous	CDH10	p.D652H	Private	5	24488185	C	G	Missense_Mutation	280	0.032142857	No	0.511	Subclonal	Disease_causing	Passenger	Neutral	passenger	Yes	No	No	No	Yes
MP11-A1	Squamous	CDH13	p.V539M	Shared	16	83712002	G	A	Missense_Mutation	292	0.095890411	No	0.755	Clonal	Disease_causing	Passenger	Neutral	passenger	Yes	No	No	No	Not tested
MP11-A2	Squamous	CDH13	p.V539M	Shared	16	83712002	G	A	Missense_Mutation	195	0.061538462	No	0.855	Clonal	Disease_causing	Passenger	Neutral	passenger	No	No	No	No	Not tested
MP11-A1	Squamous	CEP57L1	p.M275V	Shared	6	109480472	A	G	Missense_Mutation	212	0.127358491	No	1	Clonal	Neutral	Passenger	Neutral	passenger	Yes	No	No	No	Not tested
MP11-A2	Squamous	CEP57L1	p.M275V	Shared	6	109480472	A	G	Missense_Mutation	282	0.106382979	No	1	Clonal	Neutral	Passenger	Neutral	passenger	Yes	No	No	No	Not tested
MP11-B1	Chondroid	CEP57L1	p.M275V	Shared	6	109480472	A	G	Missense_Mutation	270	0.192592593	No	1	Clonal	Neutral	Passenger	Neutral	passenger	Yes	No	No	No	Not tested
MP11-A1	Squamous	CES1	p.S12A	Private	16	55866934	A	C	Missense_Mutation	89	0.078651685	No	0.541	Subclonal	Neutral	Passenger	Neutral	passenger	Yes	No	No	No	Not tested
MP11-A2	Squamous	CKAP5	p.A1311P	Private	11	46784273	C	G	Missense_Mutation	302	0.046357616	No	0.691	Subclonal	Disease_causing	Passenger	Neutral	passenger	Yes	No	No	No	Not tested
MP11-A1	Squamous	CLEC6A	p.S17T	Shared	12	8610511	T	A	Missense_Mutation	95	0.052631579	No	0.362	Subclonal	Neutral	Passenger	Neutral	passenger	No	No	No	No	Not tested
MP11-A2	Squamous	CLEC6A	p.S17T	Shared	12	8610511	T	A	Missense_Mutation	92	0.054347826	No	0.701	Subclonal	Neutral	Passenger	Neutral	passenger	No	No	No	No	Not tested
MP11-A1	Squamous	CLPB	p.R417*	Shared	11	72013356	G	A	Nonsense_Mutation	168	0.125	No	0.86	Clonal	Neutral	.	.	passenger	Yes	No	No	No	Not tested
MP11-A2	Squamous	CLPB	p.R417*	Shared	11	72013356	G	A	Nonsense_Mutation	171	0.14619883	No	1	Clonal	Neutral	.	.	passenger	No	No	No	No	Not tested
MP11-B1	Chondroid	CLPB	p.R417*	Shared	11	72013356	G	A	Nonsense_Mutation	266	0.214285714	No	1	Clonal	Neutral	.	.	passenger	Yes	No	No	No	Not tested
MP11-B1	Chondroid	CNNM4	p.R670P	Private	2	97474358	G	C	Missense_Mutation	175	0.108571429	No	0.896	Subclonal	Disease_causing	Passenger	Neutral	passenger	No	No	No	No	Yes
MP11-A2	Squamous	COBLL1	p.X333_splice	Private	2	165561617	T	A	Splice_Site	124	0.080645161	No	1	Subclonal	Disease_causing	.	.	passenger	No	No	No	No	Yes
MP11-B1	Chondroid	COL1A2	p.S801C	Private	7	94051263	C	G	Missense_Mutation	270	0.059259259	No	0.43	Subclonal	Neutral	Passenger	Disease_causing	passenger	Yes	No	No	No	Yes
MP11-A1	Squamous	COL6A5	p.E927A	Shared	3	130110385	A	C	Missense_Mutation	245	0.13877551	No	1	Clonal	Neutral	Passenger	Disease_causing	passenger	No	No	No	No	Not tested
MP11-A2	Squamous	COL6A5	p.E927A	Shared	3	130110385	A	C	Missense_Mutation	253	0.090909091	No	1	Clonal	Neutral	Passenger	Disease_causing	passenger	No	No	No	No	Not tested
MP11-B1	Chondroid	COL7A1	p.G2304R	Private	3	48609962	C	T	Missense_Mutation	99	0.161616162	No	1	Subclonal	Disease_causing	Passenger	Disease_causing	likely_pathogenic	No	No	No	No	Yes
MP11-A1	Squamous	CORO7	p.T207S	Shared	16	4438629	T	A	Missense_Mutation	97	0.164948454	No	1	Clonal	Neutral	Passenger	Neutral	passenger	Yes	No	No	No	Not tested
MP11-B1	Chondroid	CORO7	p.T207S	Shared	16	4438629	T	A	Missense_Mutation	126	0.142857143	No	0.75	Clonal	Neutral	Passenger	Neutral	passenger	Yes	No	No	No	Not tested
MP11-B1	Chondroid	CPS1	p.G1254R	Private	2	211523398	G	C	Missense_Mutation	182	0.093406593	No	0.771	Subclonal	Disease_causing	Passenger	Disease_causing	likely_pathogenic	No	No	No	No	Yes
MP11-B1	Chondroid	CSMD3	p.D2335E	Private	8	113348895	A	T	Missense_Mutation	346	0.086705202	No	0.976	Subclonal	Disease_causing	Passenger	Neutral	passenger	Yes	No	No	No	Yes
MP11-B1	Chondroid	CST6	p.L89Hfs*37	Private	11	65780321	CTGACG	C	Frame_Shift_Del	214	0.08411215	No	0.442	Subclonal	.	.	.	.	Yes	No	No	No	Yes
MP11-A2	Squamous	CTNNA1	p.P677A	Private	5	138266180	C	G	Missense_Mutation	74	0.067567568	No	0.939	Subclonal	Disease_causing	Passenger	Neutral	passenger	Yes	No	No	No	Yes
MP11-B1	Chondroid	CTTN	p.C497_K498del	Private	11	70281213	TGTGCAA	T	In_Frame_Del	496	0.102822581	No	0.848	Subclonal	.	.	.	.	Yes	No	No	No	Yes
MP11-A2	Squamous	CYP4X1	p.H490Y	Private	1	47515784	C	T	Missense_Mutation	259	0.030888031	No	0.429	Subclonal	Neutral	Passenger	Neutral	passenger	No	No	No	No	Not tested
MP11-B1	Chondroid	DENND3	p.N972D	Private	8	142199154	A	G	Missense_Mutation	304	0.121710526	No	0.883	Subclonal	Disease_causing	Passenger	Neutral	passenger	Yes	No	No	No	Not tested
MP11-A2	Squamous	DENND6A	p.Q247E	Private	3	57644868	G	C	Missense_Mutation	227	0.088105727	No	1	Subclonal	Disease_causing	Passenger	.	passenger	No	No	No	No	Not tested
MP11-B1	Chondroid	DHX30	p.G859R	Private	3	47890040	G	C	Missense_Mutation	145	0.04137931	No	0.259	Subclonal	Disease_causing	Passenger	Neutral	passenger	No	No	No	No	Yes
MP11-A1	Squamous	DISP2	p.P745H	Shared	15	40660547	C	A	Missense_Mutation	115	0.417391304	No	1	Clonal	Disease_causing	Passenger	Neutral	passenger	No	No	No	No	Not tested
MP11-A2	Squamous	DISP2	p.P745H	Shared	15	40660547	C	A	Missense_Mutation	82	0.304878049	No	1	Clonal	Disease_causing	Passenger	Neutral	passenger	No	No	No	No	Not tested
MP11-A1	Squamous	DMRTB1	p.R31C	Shared	1	53925217	C	T	Missense_Mutation	229	0.231441048	No	1	Clonal	Disease_causing	Passenger	.	passenger	Yes	No	No	No	Yes
MP11-A2	Squamous	DMRTB1	p.R31C	Shared	1	53925217	C	T	Missense_Mutation	196	0.163265306	No	1	Clonal	Disease_causing	Passenger	.	passenger	No	No	No	No	Yes
MP11-B1	Chondroid	DMRTB1	p.R31C	Shared	1	53925217	C	T	Missense_Mutation	203	0.300492611	No	1	Clonal	Disease_causing	Passenger	.	passenger	Yes	No	No	No	Yes
MP11-B1	Chondroid	DNAH11	p.V127Lfs*12	Private	7	21584648	CTTGT	C	Frame_Shift_Del	102	0.205882353	No	1	Subclonal	.	.	.	.	No	No	No	No	Yes
MP11-A2	Squamous	DNAH8	p.I1298L	Private	6	38810220	A	T	Missense_Mutation	213	0.037558685	No	0.56	Subclonal	Disease_causing	Passenger	Neutral	passenger	Yes	No	No	No	Not tested
MP11-B1	Chondroid	DNAH9	p.I2857T	Private	17	11711198	T	C	Missense_Mutation	63	0.142857143	No	0.75	Subclonal	Disease_causing	Passenger	Neutral	passenger	No	No	No	No	Yes
MP11-A1	Squamous	DROSHA	p.S1101Y	Shared	5	31423011	G	T	Missense_Mutation	160	0.09375	No	0.738	Subclonal	Disease_causing	Passenger	Neutral	passenger	Yes	Yes	No	No	Not tested
MP11-A2	Squamous	DROSHA	p.S1101Y	Shared	5	31423011	G	T	Missense_Mutation	161	0.111801242	No	1	Subclonal	Disease_causing	Passenger	Neutral	passenger	Yes	Yes	No	No	Not tested
MP11-B1	Chondroid	DSCAML1	p.S84R	Private	11	117651500	G	C	Missense_Mutation	140	0.078571429	No	0.413	Subclonal	Disease_causing	Passenger	Neutral	passenger	Yes	No	No	No	Yes
MP11-A1	Squamous	DST	p.A456V	Shared	6	56501437	G	A	Missense_Mutation	267	0.269662921	No	1	Clonal	Disease_causing	Passenger	Disease_causing	likely_pathogenic	Yes	No	No	No	Not tested
MP11-A2	Squamous	DST	p.A456V	Shared	6	56501437	G	A	Missense_Mutation	338	0.198224852	No	1	Clonal	Disease_causing	Passenger	Disease_causing	likely_pathogenic	Yes	No	No	No	Not tested
MP11-B1	Chondroid	DST	p.A456V	Shared	6	56501437	G	A	Missense_Mutation	295	0.138983051	No	0.869	Clonal	Disease_causing	Passenger	Disease_causing	likely_pathogenic	Yes	No	No	No	Not tested
MP11-A1	Squamous	EEA1	p.C1361S	Shared	12	93170651	C	G	Missense_Mutation	215	0.106976744	No	0.629	Subclonal	Disease_causing	Passenger	Disease_causing	likely_pathogenic	No	No	No	No	Not tested
MP11-A2	Squamous	EEA1	p.C1361S	Shared	12	93170651	C	G	Missense_Mutation	263	0.102661597	No	1	Subclonal	Disease_causing	Passenger	Disease_causing	likely_pathogenic	No	No	No	No	Not tested
MP11-A1	Squamous	EGFLAM	p.F641_P643del	Shared	5	38427218	ACATTTCGGC	A	In_Frame_Del	260	0.130769231	No	1	Clonal	.	.	.	.	Yes	No	No	No	Not tested
MP11-A2	Squamous	EGFLAM	p.F641_P643del	Shared	5	38427218	ACATTTCGGC	A	In_Frame_Del	278	0.097122302	No	1	Clonal	.	.	.	.	Yes	No	No	No	Not tested
MP11-B1	Chondroid	EGFLAM	p.F641_P643del	Shared	5	38427218	ACATTTCGGC	A	In_Frame_Del	301	0.069767442	No	0.436	Clonal	.	.	.	.	No	No	No	No	Not tested
MP11-B1	Chondroid	EME1	p.S411T	Private	17	48456548	G	C	Missense_Mutation	311	0.109324759	No	0.793	Subclonal	Neutral	Passenger	Neutral	passenger	No	No	No	No	Not tested
MP11-B1	Chondroid	ENO1	p.D266Y	Private	1	8925413	C	A	Missense_Mutation	224	0.299107143	No	1	Subclonal	Disease_causing	Passenger	Neutral	passenger	Yes	No	No	No	Yes
MP11-B1	Chondroid	EPHX1	p.W227S	Private	1	226027005	G	C	Missense_Mutation	172	0.127906977	No	1	Subclonal	Disease_causing	Passenger	Neutral	passenger	Yes	No	No	No	Not tested
MP11-A2	Squamous	EZR	p.I260V	Private	6	159197457	T	C	Missense_Mutation	119	0.109243697	No	1	Subclonal	Disease_causing	Passenger	Disease_causing	likely_pathogenic	No	Yes	No	No	Yes
MP11-B1	Chondroid	F8	p.L548V	Private	X	154185342	G	C	Missense_Mutation	316	0.126582278	No	0.538	Subclonal	Disease_causing	Passenger	Disease_causing	likely_pathogenic	Yes	No	No	No	Yes
MP11-B1	Chondroid	FAM131B	p.A244S	Private	7	143053996	C	A	Missense_Mutation	270	0.155555556	No	1	Subclonal	Disease_causing	Passenger	Neutral	passenger	Yes	No	No	No	Yes
MP11-B1	Chondroid	FAM189A2	p.L407V	Private	9	72006586	C	G	Missense_Mutation	209	0.172248804	No	1	Subclonal	Disease_causing	Passenger	Neutral	passenger	Yes	No	No	No	Yes
MP11-A2	Squamous	FANCI	p.I175L	Private	15	89806669	A	C	Missense_Mutation	231	0.056277056	No	0.782	Subclonal	Disease_causing	Passenger	Neutral	passenger	No	No	No	No	Not tested
MP11-A1	Squamous	FANCM	p.E167K	Shared	14	45605733	G	A	Missense_Mutation	264	0.412878788	No	1	Clonal	Disease_causing	Passenger	Neutral	passenger	No	No	No	No	Yes
MP11-A2	Squamous	FANCM	p.E167K	Shared	14	45605733	G	A	Missense_Mutation	340	0.288235294	No	1	Clonal	Disease_causing	Passenger	Neutral	passenger	No	No	No	No	Yes
MP11-B1	Chondroid	FANCM	p.E167K	Shared	14	45605733	G	A	Missense_Mutation	344	0.177325581	No	0.931	Clonal	Disease_causing	Passenger	Neutral	passenger	Yes	No	No	No	Yes
MP11-B1	Chondroid	FAT3	p.I2723V	Private	11	92534346	A	G	Missense_Mutation	426	0.159624413	No	1	Subclonal	Disease_causing	Passenger	Neutral	passenger	Yes	No	No	No	Yes
MP11-A1	Squamous	FBXO27	p.Q207L	Shared	19	39517598	T	A	Missense_Mutation	236	0.118644068	No	0.816	Clonal	Neutral	Passenger	Neutral	passenger	Yes	No	No	No	Not tested
MP11-A2	Squamous	FBXO27	p.Q207L	Shared	19	39517598	T	A	Missense_Mutation	175	0.08	No	1	Clonal	Neutral	Passenger	Neutral	passenger	Yes	No	No	No	Not tested
MP11-A1	Squamous	FDXACB1	p.K547N	Shared	11	111745880	C	G	Missense_Mutation	298	0.228187919	No	1	Clonal	Neutral	Passenger	Neutral	passenger	Yes	No	No	No	Not tested
MP11-A2	Squamous	FDXACB1	p.K547N	Shared	11	111745880	C	G	Missense_Mutation	246	0.142276423	No	1	Clonal	Neutral	Passenger	Neutral	passenger	No	No	No	No	Not tested
MP11-B1	Chondroid	FDXACB1	p.K547N	Shared	11	111745880	C	G	Missense_Mutation	278	0.287769784	No	1	Clonal	Neutral	Passenger	Neutral	passenger	Yes	No	No	No	Not tested
MP11-A1	Squamous	FER1L6	p.D1378H	Shared	8	125088478	G	C	Missense_Mutation	411	0.077858881	No	0.769	Clonal	Disease_causing	Driver	Disease_causing	likely_pathogenic	Yes	No	No	No	Yes
MP11-A2	Squamous	FER1L6	p.D1378H	Shared	8	125088478	G	C	Missense_Mutation	328	0.06402439	No	1	Clonal	Disease_causing	Driver	Disease_causing	likely_pathogenic	Yes	No	No	No	Yes
MP11-B1	Chondroid	FER1L6	p.D1378H	Shared	8	125088478	G	C	Missense_Mutation	535	0.239252336	No	1	Clonal	Disease_causing	Driver	Disease_causing	likely_pathogenic	Yes	No	No	No	Yes
MP11-A2	Squamous	FLT4	p.L624M	Private	5	180048692	G	T	Missense_Mutation	138	0.065217391	No	0.907	Subclonal	Disease_causing	Passenger	Neutral	passenger	No	Yes	No	No	Not tested
MP11-B1	Chondroid	FMN2	p.L711P	Private	1	240370244	T	C	Missense_Mutation	534	0.172284644	No	1	Subclonal	Neutral	Passenger	Neutral	passenger	Yes	No	No	No	Yes
MP11-B1	Chondroid	FMN2	p.S614P	Private	1	240341278	T	C	Missense_Mutation	337	0.06231454	No	0.763	Subclonal	Neutral	Passenger	Neutral	passenger	Yes	No	No	No	Yes
MP11-B1	Chondroid	FSIP1	p.L176Ffs*8	Private	15	40056053	T	TA	Frame_Shift_Ins	141	0.113475177	No	0.482	Subclonal	.	.	.	.	No	No	No	No	Yes
MP11-B1	Chondroid	GALNT13	p.K377Q	Private	2	155158075	A	C	Missense_Mutation	110	0.109090909	No	0.9	Subclonal	Disease_causing	Passenger	Neutral	passenger	No	No	No	No	Yes
MP11-A2	Squamous	GALNT3	p.P503A	Private	2	166611459	G	C	Missense_Mutation	376	0.053191489	No	0.899	Subclonal	Disease_causing	Passenger	Neutral	passenger	No	No	No	No	Not tested
MP11-B1	Chondroid	GAS2L1	p.V158M	Private	22	29704567	G	A	Missense_Mutation	264	0.196969697	No	1	Subclonal	Disease_causing	Passenger	Neutral	passenger	Yes	No	No	No	Yes
MP11-A1	Squamous	GDF2	p.C393S	Shared	10	48413690	C	G	Missense_Mutation	205	0.302439024	No	1	Clonal	Disease_causing	Passenger	Disease_causing	likely_pathogenic	No	No	No	No	Not tested
MP11-A2	Squamous	GDF2	p.C393S	Shared	10	48413690	C	G	Missense_Mutation	171	0.233918129	No	1	Clonal	Disease_causing	Passenger	Disease_causing	likely_pathogenic	No	No	No	No	Not tested
MP11-B1	Chondroid	GDF2	p.C393S	Shared	10	48413690	C	G	Missense_Mutation	310	0.319354839	No	1	Clonal	Disease_causing	Passenger	Disease_causing	likely_pathogenic	Yes	No	No	No	Not tested
MP11-A1	Squamous	GJA10	p.E127K	Shared	6	90604566	G	A	Missense_Mutation	157	0.242038217	No	1	Clonal	Disease_causing	Passenger	Disease_causing	likely_pathogenic	Yes	No	No	No	Not tested
MP11-A2	Squamous	GJA10	p.E127K	Shared	6	90604566	G	A	Missense_Mutation	152	0.269736842	No	1	Clonal	Disease_causing	Passenger	Disease_causing	likely_pathogenic	Yes	No	No	No	Not tested
MP11-B1	Chondroid	GJA10	p.E127K	Shared	6	90604566	G	A	Missense_Mutation	167	0.245508982	No	1	Clonal	Disease_causing	Passenger	Disease_causing	likely_pathogenic	Yes	No	No	No	Not tested
MP11-A1	Squamous	GPANK1	p.R277Q	Shared	6	31630284	C	T	Missense_Mutation	521	0.214971209	No	1	Clonal	Disease_causing	Passenger	Neutral	passenger	Yes	No	No	No	Not tested
MP11-A2	Squamous	GPANK1	p.R277Q	Shared	6	31630284	C	T	Missense_Mutation	288	0.128472222	No	1	Clonal	Disease_causing	Passenger	Neutral	passenger	Yes	No	No	No	Not tested
MP11-A1	Squamous	GPN1	p.A113P	Private	2	27854750	G	C	Missense_Mutation	280	0.128571429	No	1	Clonal	Disease_causing	Passenger	Neutral	passenger	No	No	No	No	Not tested
MP11-A2	Squamous	GPN2	p.L73V	Private	1	27216371	G	C	Missense_Mutation	248	0.064516129	No	0.897	Subclonal	Disease_causing	Passenger	Neutral	passenger	No	No	No	No	Not tested
MP11-B1	Chondroid	GPR141	p.I296M	Private	7	37780883	T	G	Missense_Mutation	138	0.152173913	No	0.799	Subclonal	Neutral	Passenger	Neutral	passenger	No	No	No	No	Yes
MP11-A1	Squamous	GTF3C1	p.E377K	Shared	16	27519974	C	T	Missense_Mutation	68	0.279411765	No	1	Clonal	Disease_causing	Passenger	Neutral	passenger	Yes	No	No	No	Yes
MP11-A2	Squamous	GTF3C1	p.E377K	Shared	16	27519974	C	T	Missense_Mutation	78	0.205128205	No	1	Clonal	Disease_causing	Passenger	Neutral	passenger	No	No	No	No	Yes
MP11-B1	Chondroid	GTF3C1	p.E377K	Shared	16	27519974	C	T	Missense_Mutation	63	0.26984127	No	1	Clonal	Disease_causing	Passenger	Neutral	passenger	Yes	No	No	No	Yes
MP11-B1	Chondroid	GUCY1A2	p.Q541H	Private	11	106680788	T	G	Missense_Mutation	138	0.137681159	No	0.723	Subclonal	Disease_causing	Passenger	Disease_causing	likely_pathogenic	Yes	No	No	No	Yes
MP11-A1	Squamous	GUK1	p.R169W	Shared	1	228335367	C	T	Missense_Mutation	76	0.210526316	No	1	Clonal	Disease_causing	Passenger	Neutral	passenger	Yes	No	No	No	Yes
MP11-A2	Squamous	GUK1	p.R169W	Shared	1	228335367	C	T	Missense_Mutation	61	0.147540984	No	1	Clonal	Disease_causing	Passenger	Neutral	passenger	Yes	No	No	No	Yes
MP11-B1	Chondroid	GUK1	p.R169W	Shared	1	228335367	C	T	Missense_Mutation	116	0.413793103	No	1	Clonal	Disease_causing	Passenger	Neutral	passenger	Yes	No	No	No	Yes
MP11-A1	Squamous	HTR1D	p.P325S	Shared	1	23519740	G	A	Missense_Mutation	401	0.274314214	No	1	Clonal	Disease_causing	Passenger	Neutral	passenger	Yes	No	No	No	Yes
MP11-A2	Squamous	HTR1D	p.P325S	Shared	1	23519740	G	A	Missense_Mutation	296	0.212837838	No	1	Clonal	Disease_causing	Passenger	Neutral	passenger	No	No	No	No	Yes
MP11-B1	Chondroid	HTR1D	p.P325S	Shared	1	23519740	G	A	Missense_Mutation	280	0.189285714	No	0.805	Clonal	Disease_causing	Passenger	Neutral	passenger	Yes	No	No	No	Yes
MP11-A1	Squamous	HTR1F	p.L112_L114del	Shared	3	88040230	ATCTTGCATC	A	In_Frame_Del	186	0.048387097	No	0.381	Subclonal	.	.	.	.	No	No	No	No	Not tested
MP11-A2	Squamous	HTR1F	p.L112_L114del	Shared	3	88040230	ATCTTGCATC	A	In_Frame_Del	266	0.033834586	No	0.538	Subclonal	.	.	.	.	No	No	No	No	Not tested
MP11-B1	Chondroid	HTRA1	p.E277Q	Private	10	124266258	G	C	Missense_Mutation	639	0.211267606	No	1	Subclonal	Disease_causing	Passenger	Disease_causing	likely_pathogenic	Yes	No	No	No	Not tested
MP11-A1	Squamous	HYDIN	p.Y5036N	Shared	16	70841743	A	T	Missense_Mutation	426	0.098591549	No	0.777	Clonal	Neutral	Passenger	Neutral	passenger	Yes	No	No	No	Not tested
MP11-A2	Squamous	HYDIN	p.Y5036N	Shared	16	70841743	A	T	Missense_Mutation	364	0.065934066	No	0.917	Clonal	Neutral	Passenger	Neutral	passenger	No	No	No	No	Not tested
MP11-B1	Chondroid	HYDIN	p.Y5036N	Shared	16	70841743	A	T	Missense_Mutation	289	0.114186851	No	0.6	Clonal	Neutral	Passenger	Neutral	passenger	Yes	No	No	No	Not tested
MP11-B1	Chondroid	IFT88	p.S525T	Private	13	21212630	T	A	Missense_Mutation	148	0.081081081	No	0.426	Subclonal	Disease_causing	Passenger	Neutral	passenger	Yes	No	No	No	Not tested
MP11-A1	Squamous	IGSF9	p.L113M	Shared	1	159907539	G	T	Missense_Mutation	243	0.115226337	No	0.908	Clonal	Disease_causing	Passenger	Neutral	passenger	Yes	No	No	No	Not tested
MP11-A2	Squamous	IGSF9	p.L113M	Shared	1	159907539	G	T	Missense_Mutation	178	0.08988764	No	1	Clonal	Disease_causing	Passenger	Neutral	passenger	Yes	No	No	No	Not tested
MP11-B1	Chondroid	IL2RG	p.N254S	Private	X	70328542	T	C	Missense_Mutation	179	0.100558659	No	0.428	Subclonal	Neutral	Passenger	Disease_causing	passenger	No	No	No	No	Yes
MP11-A2	Squamous	IL33	p.K180N	Private	9	6254481	G	C	Missense_Mutation	244	0.069672131	No	1	Subclonal	Neutral	Passenger	Neutral	passenger	No	No	No	No	Not tested
MP11-A2	Squamous	INADL	p.D575H	Private	1	62288656	G	C	Missense_Mutation	273	0.062271062	No	0.866	Subclonal	Disease_causing	Passenger	Neutral	passenger	No	No	No	No	Not tested
MP11-B1	Chondroid	INPPL1	p.T213A	Private	11	71940252	A	G	Missense_Mutation	348	0.068965517	No	0.569	Subclonal	Disease_causing	Passenger	Disease_causing	likely_pathogenic	Yes	No	No	No	Yes
MP11-A1	Squamous	IRF6	p.E264_E272del	Shared	1	209964083	GCTCCAGGCTGACGGGACCAAAGAGCTC	G	In_Frame_Del	333	0.069069069	No	0.544	Subclonal	.	.	.	.	Yes	No	No	No	Not tested
MP11-A2	Squamous	IRF6	p.E264_E272del	Shared	1	209964083	GCTCCAGGCTGACGGGACCAAAGAGCTC	G	In_Frame_Del	257	0.042801556	No	0.638	Subclonal	.	.	.	.	Yes	No	No	No	Not tested
MP11-B1	Chondroid	IRF6	p.E264_E272del	Shared	1	209964083	GCTCCAGGCTGACGGGACCAAAGAGCTC	G	In_Frame_Del	393	0.195928753	No	1	Subclonal	.	.	.	.	Yes	No	No	No	Not tested
MP11-A1	Squamous	ITCH	p.D774G	Shared	20	33077175	A	G	Missense_Mutation	198	0.212121212	No	1	Clonal	Disease_causing	Driver	Neutral	likely_pathogenic	Yes	No	No	No	Not tested
MP11-A2	Squamous	ITCH	p.D774G	Shared	20	33077175	A	G	Missense_Mutation	163	0.208588957	No	1	Clonal	Disease_causing	Driver	Neutral	likely_pathogenic	Yes	No	No	No	Not tested
MP11-B1	Chondroid	ITFG2	p.E362Q	Private	12	2932953	G	C	Missense_Mutation	113	0.230088496	No	1	Subclonal	Disease_causing	Passenger	Neutral	passenger	No	No	No	No	Yes
MP11-B1	Chondroid	ITGA4	p.R971G	Private	2	182399570	A	G	Missense_Mutation	90	0.077777778	No	0.564	Subclonal	Disease_causing	Passenger	Neutral	passenger	No	No	No	No	Yes
MP11-A1	Squamous	KCNE1L	p.C72S	Shared	X	108868036	A	T	Missense_Mutation	232	0.181034483	No	1	Clonal	Disease_causing	Passenger	Neutral	passenger	No	No	No	No	Not tested
MP11-A2	Squamous	KCNE1L	p.C72S	Shared	X	108868036	A	T	Missense_Mutation	152	0.144736842	No	1	Clonal	Disease_causing	Passenger	Neutral	passenger	No	No	No	No	Not tested
MP11-B1	Chondroid	KCNH1	p.Y526C	Private	1	210977394	T	C	Missense_Mutation	264	0.125	No	1	Subclonal	Disease_causing	Driver	Disease_causing	likely_pathogenic	Yes	No	No	No	Yes
MP11-A1	Squamous	KCNK12	p.L313V	Shared	2	47748402	G	C	Missense_Mutation	158	0.170886076	No	1	Clonal	Neutral	Passenger	Neutral	passenger	No	No	No	No	Not tested
MP11-A2	Squamous	KCNK12	p.L313V	Shared	2	47748402	G	C	Missense_Mutation	128	0.1796875	No	1	Clonal	Neutral	Passenger	Neutral	passenger	No	No	No	No	Not tested
MP11-B1	Chondroid	KCNK12	p.L313V	Shared	2	47748402	G	C	Missense_Mutation	199	0.090452261	No	0.746	Clonal	Neutral	Passenger	Neutral	passenger	No	No	No	No	Not tested
MP11-A1	Squamous	KCNK3	p.S286L	Shared	2	26951108	C	T	Missense_Mutation	151	0.119205298	No	1	Clonal	Neutral	Passenger	Neutral	passenger	No	No	No	No	Not tested
MP11-A2	Squamous	KCNK3	p.S286L	Shared	2	26951108	C	T	Missense_Mutation	86	0.069767442	No	1	Clonal	Neutral	Passenger	Neutral	passenger	No	No	No	No	Not tested
MP11-B1	Chondroid	KCNK3	p.S286L	Shared	2	26951108	C	T	Missense_Mutation	186	0.123655914	No	1	Clonal	Neutral	Passenger	Neutral	passenger	No	No	No	No	Not tested
MP11-B1	Chondroid	KCNK7	p.A52P	Private	11	65363090	C	G	Missense_Mutation	135	0.111111111	No	0.584	Subclonal	Neutral	Passenger	Neutral	passenger	Yes	No	No	No	Yes
MP11-B1	Chondroid	KCNN3	p.A357G	Private	1	154744829	G	C	Missense_Mutation	177	0.13559322	No	0.983	Subclonal	Disease_causing	Passenger	Disease_causing	likely_pathogenic	Yes	No	No	No	Yes
MP11-B1	Chondroid	KCTD10	p.K241M	Private	12	109893924	T	A	Missense_Mutation	229	0.061135371	No	0.382	Subclonal	Disease_causing	Passenger	Neutral	passenger	No	No	No	No	Yes
MP11-B1	Chondroid	KHK	p.I101M	Private	2	27317438	C	G	Missense_Mutation	316	0.189873418	No	1	Subclonal	Disease_causing	Passenger	Neutral	passenger	No	No	No	No	Yes
MP11-A1	Squamous	KIAA1551	p.K1365Lfs*9	Shared	12	32137980	TGAAACTCAAATCA	T	Frame_Shift_Del	179	0.195530726	No	1	Clonal	.	.	.	.	Yes	No	No	No	Not tested
MP11-A2	Squamous	KIAA1551	p.K1365Lfs*9	Shared	12	32137980	TGAAACTCAAATCA	T	Frame_Shift_Del	215	0.111627907	No	1	Clonal	.	.	.	.	Yes	No	No	No	Not tested
MP11-B1	Chondroid	KIAA1551	p.K1365Lfs*9	Shared	12	32137980	TGAAACTCAAATCA	T	Frame_Shift_Del	225	0.084444444	No	0.612	Clonal	.	.	.	.	Yes	No	No	No	Not tested
MP11-B1	Chondroid	KIF1C	p.P1073R	Private	17	4927352	C	G	Missense_Mutation	254	0.196850394	No	1	Subclonal	Neutral	Passenger	Neutral	passenger	No	No	No	No	Yes
MP11-A2	Squamous	KLRF2	p.Y32C	Private	12	10037557	A	G	Missense_Mutation	122	0.057377049	No	0.74	Subclonal	Neutral	Passenger	Neutral	passenger	No	No	No	No	Not tested
MP11-A2	Squamous	KLRF2	p.A111G	Private	12	10045733	C	G	Missense_Mutation	176	0.056818182	No	0.733	Subclonal	Neutral	Passenger	Neutral	passenger	No	No	No	No	Not tested
MP11-B1	Chondroid	LEPREL4	p.D263H	Private	17	39967115	C	G	Missense_Mutation	173	0.092485549	No	0.671	Subclonal	Disease_causing	Passenger	Neutral	passenger	No	No	No	No	Yes
MP11-B1	Chondroid	LGR5	p.K675N	Private	12	71977815	A	C	Missense_Mutation	199	0.190954774	No	0.812	Subclonal	Disease_causing	Passenger	Disease_causing	likely_pathogenic	No	No	No	No	Yes
MP11-A1	Squamous	LRP10	p.R51G	Shared	14	23342591	C	G	Missense_Mutation	338	0.340236686	No	1	Clonal	Disease_causing	Passenger	Neutral	passenger	No	No	No	No	Not tested
MP11-A2	Squamous	LRP10	p.R51G	Shared	14	23342591	C	G	Missense_Mutation	345	0.217391304	No	1	Clonal	Disease_causing	Passenger	Neutral	passenger	No	No	No	No	Not tested
MP11-B1	Chondroid	LRP10	p.R51G	Shared	14	23342591	C	G	Missense_Mutation	356	0.199438202	No	1	Clonal	Disease_causing	Passenger	Neutral	passenger	Yes	No	No	No	Not tested
MP11-A1	Squamous	LRRC2	p.L371I	Shared	3	46560509	G	T	Missense_Mutation	205	0.224390244	No	1	Clonal	Disease_causing	Passenger	Neutral	passenger	No	No	No	No	Not tested
MP11-A2	Squamous	LRRC2	p.L371I	Shared	3	46560509	G	T	Missense_Mutation	220	0.186363636	No	1	Clonal	Disease_causing	Passenger	Neutral	passenger	No	No	No	No	Not tested
MP11-B1	Chondroid	LRRC2	p.L371I	Shared	3	46560509	G	T	Missense_Mutation	236	0.322033898	No	1	Clonal	Disease_causing	Passenger	Neutral	passenger	No	No	No	No	Not tested
MP11-B1	Chondroid	LUM	p.L329V	Private	12	91497974	G	C	Missense_Mutation	204	0.161764706	No	1	Subclonal	Disease_causing	Passenger	Neutral	passenger	No	No	No	No	Yes
MP11-B1	Chondroid	LXN	p.G206R	Private	3	158384488	C	G	Missense_Mutation	170	0.058823529	No	0.427	Subclonal	Disease_causing	Passenger	Neutral	passenger	No	No	No	No	Yes
MP11-A1	Squamous	LYN	p.K40E	Private	8	56854536	A	G	Missense_Mutation	287	0.038327526	No	0.302	Subclonal	Disease_causing	Passenger	Neutral	passenger	Yes	No	No	No	Not tested
MP11-B1	Chondroid	LZTS3	p.D539H	Private	20	3145507	C	G	Missense_Mutation	105	0.180952381	No	0.769	Subclonal	Disease_causing	Passenger	Neutral	passenger	Yes	No	No	No	Yes
MP11-B1	Chondroid	LZTS3	p.S538T	Private	20	3145509	C	G	Missense_Mutation	103	0.184466019	No	0.784	Subclonal	Disease_causing	Passenger	Neutral	passenger	Yes	No	No	No	Yes
MP11-B1	Chondroid	MAGED1	p.N631S	Private	X	51641275	A	G	Missense_Mutation	247	0.165991903	No	0.872	Subclonal	Disease_causing	Passenger	Neutral	passenger	Yes	No	No	No	Yes
MP11-B1	Chondroid	MBTPS2	p.T97M	Private	X	21863354	C	T	Missense_Mutation	202	0.138613861	No	0.728	Subclonal	Disease_causing	Passenger	Disease_causing	likely_pathogenic	Yes	No	No	No	Yes
MP11-B1	Chondroid	MED24	p.R629P	Private	17	38182508	C	G	Missense_Mutation	270	0.059259259	No	0.43	Subclonal	Disease_causing	Passenger	Neutral	passenger	No	No	No	No	Yes
MP11-A1	Squamous	MEGF6	p.L1511Rfs*5	Shared	1	3407502	TCGGGGAG	T	Frame_Shift_Del	232	0.168103448	No	1	Clonal	.	.	.	.	Yes	No	No	No	Not tested
MP11-A2	Squamous	MEGF6	p.L1511Rfs*5	Shared	1	3407502	TCGGGGAG	T	Frame_Shift_Del	166	0.168674699	No	1	Clonal	.	.	.	.	No	No	No	No	Not tested
MP11-B1	Chondroid	MEGF6	p.L1511Rfs*5	Shared	1	3407502	TCGGGGAG	T	Frame_Shift_Del	138	0.260869565	No	1	Clonal	.	.	.	.	Yes	No	No	No	Not tested
MP11-A1	Squamous	MFSD11	p.X51_splice	Shared	17	74735077	T	C	Splice_Site	283	0.197879859	No	1	Clonal	Disease_causing	.	.	likely_pathogenic	No	No	No	No	Not tested
MP11-A2	Squamous	MFSD11	p.X51_splice	Shared	17	74735077	T	C	Splice_Site	277	0.133574007	No	1	Clonal	Disease_causing	.	.	likely_pathogenic	No	No	No	No	Not tested
MP11-B1	Chondroid	MICALCL	p.R162G	Private	11	12315462	A	G	Missense_Mutation	270	0.092592593	No	0.671	Subclonal	Neutral	Passenger	Neutral	passenger	Yes	No	No	No	Yes
MP11-B1	Chondroid	MIS18BP1	p.S175*	Private	14	45715966	G	C	Nonsense_Mutation	68	0.191176471	No	1	Subclonal	Neutral	.	.	passenger	Yes	No	No	No	Not tested
MP11-A1	Squamous	MKNK1	p.P386L	Shared	1	47025921	G	A	Missense_Mutation	139	0.273381295	No	1	Clonal	Disease_causing	Passenger	Neutral	passenger	Yes	No	No	No	Not tested
MP11-A2	Squamous	MKNK1	p.P386L	Shared	1	47025921	G	A	Missense_Mutation	136	0.154411765	No	1	Clonal	Disease_causing	Passenger	Neutral	passenger	No	No	No	No	Not tested
MP11-B1	Chondroid	MKNK1	p.P386L	Shared	1	47025921	G	A	Missense_Mutation	165	0.163636364	No	0.859	Clonal	Disease_causing	Passenger	Neutral	passenger	Yes	No	No	No	Not tested
MP11-B1	Chondroid	MMP28	p.R122G	Private	17	34105907	G	C	Missense_Mutation	477	0.085953878	No	0.623	Subclonal	Disease_causing	Passenger	Neutral	passenger	Yes	No	No	No	Yes
MP11-B1	Chondroid	MOGS	p.A10Sfs*29	Private	2	74692345	TGC	T	Frame_Shift_Del	255	0.117647059	No	0.853	Subclonal	.	.	.	.	No	No	No	No	Yes
MP11-B1	Chondroid	MRC2	p.N69S	Private	17	60741996	A	G	Missense_Mutation	470	0.10212766	No	0.741	Subclonal	Disease_causing	Passenger	Neutral	passenger	No	No	No	No	Yes
MP11-B1	Chondroid	MROH2A	p.E1448K	Private	2	234737614	G	A	Missense_Mutation	247	0.109311741	No	0.902	Subclonal	Disease_causing	Passenger	Neutral	passenger	No	No	No	No	Yes
MP11-A1	Squamous	MRVI1	p.P230S	Shared	11	10649537	G	A	Missense_Mutation	95	0.2	No	1	Clonal	Disease_causing	Passenger	Neutral	passenger	Yes	No	No	No	Not tested
MP11-A2	Squamous	MRVI1	p.P230S	Shared	11	10649537	G	A	Missense_Mutation	62	0.177419355	No	1	Clonal	Disease_causing	Passenger	Neutral	passenger	Yes	No	No	No	Not tested
MP11-B1	Chondroid	MRVI1	p.P230S	Shared	11	10649537	G	A	Missense_Mutation	79	0.316455696	No	1	Clonal	Disease_causing	Passenger	Neutral	passenger	Yes	No	No	No	Not tested
MP11-B1	Chondroid	MTBP	p.L172V	Private	8	121467704	C	G	Missense_Mutation	233	0.068669528	No	0.773	Subclonal	Neutral	Passenger	.	passenger	Yes	No	No	No	Yes
MP11-A1	Squamous	MYH3	p.C793R	Shared	17	10543699	A	G	Missense_Mutation	389	0.29562982	No	1	Clonal	Disease_causing	Passenger	Neutral	passenger	No	No	No	No	Not tested
MP11-A2	Squamous	MYH3	p.C793R	Shared	17	10543699	A	G	Missense_Mutation	347	0.207492795	No	1	Clonal	Disease_causing	Passenger	Neutral	passenger	No	No	No	No	Not tested
MP11-B1	Chondroid	MYO15A	p.Y117_R123del	Private	17	18022460	GGCTACGGCCGCCTGCGGCCGC	G	In_Frame_Del	152	0.078947368	No	0.415	Subclonal	.	.	.	.	No	No	No	No	Not tested
MP11-A1	Squamous	MYO7A	p.D1960E	Shared	11	76919498	C	A	Missense_Mutation	191	0.09947644	No	0.684	Subclonal	Disease_causing	Passenger	Neutral	passenger	Yes	No	No	No	Not tested
MP11-A2	Squamous	MYO7A	p.D1960E	Shared	11	76919498	C	A	Missense_Mutation	150	0.04	No	0.516	Subclonal	Disease_causing	Passenger	Neutral	passenger	No	No	No	No	Not tested
MP11-B1	Chondroid	NCDN	p.Y39S	Private	1	36024790	A	C	Missense_Mutation	189	0.137566138	No	0.722	Subclonal	Disease_causing	Passenger	Neutral	passenger	Yes	No	No	No	Yes
MP11-A1	Squamous	NELFCD	p.R526*	Shared	20	57568760	C	T	Nonsense_Mutation	438	0.034246575	No	0.27	Subclonal	Neutral	.	.	passenger	Yes	No	No	No	Not tested
MP11-A2	Squamous	NELFCD	p.R526*	Shared	20	57568760	C	T	Nonsense_Mutation	295	0.030508475	No	0.455	Subclonal	Neutral	.	.	passenger	Yes	No	No	No	Not tested
MP11-B1	Chondroid	NEU4	p.R255G	Private	2	242757643	C	G	Missense_Mutation	142	0.154929577	No	0.969	Subclonal	Disease_causing	Passenger	Disease_causing	likely_pathogenic	No	No	No	No	Yes
MP11-B1	Chondroid	NFX1	p.L124V	Private	9	33294762	C	G	Missense_Mutation	372	0.075268817	No	0.32	Subclonal	Neutral	Passenger	Neutral	passenger	No	No	No	No	Not tested
MP11-A1	Squamous	NSD1	p.N1933H	Shared	5	176707740	A	C	Missense_Mutation	259	0.44015444	No	1	Clonal	Disease_causing	Passenger	Disease_causing	likely_pathogenic	No	Yes	Yes	Yes	Not tested
MP11-A2	Squamous	NSD1	p.N1933H	Shared	5	176707740	A	C	Missense_Mutation	271	0.339483395	No	1	Clonal	Disease_causing	Passenger	Disease_causing	likely_pathogenic	No	Yes	Yes	Yes	Not tested
MP11-B1	Chondroid	OBSCN	p.Y8270*	Private	1	228560418	C	G	Nonsense_Mutation	309	0.142394822	No	1	Subclonal	Disease_causing	.	.	passenger	Yes	No	No	No	Yes
MP11-A1	Squamous	OLFM4	p.I212S	Shared	13	53617304	T	G	Missense_Mutation	180	0.077777778	No	0.69	Subclonal	Disease_causing	Passenger	Disease_causing	likely_pathogenic	Yes	No	No	No	Not tested
MP11-A2	Squamous	OLFM4	p.I212S	Shared	13	53617304	T	G	Missense_Mutation	167	0.071856287	No	1	Subclonal	Disease_causing	Passenger	Disease_causing	likely_pathogenic	Yes	No	No	No	Not tested
MP11-A1	Squamous	OR10J3	p.R316Afs*7	Shared	1	159283491	CCTTCACAACCTCT	C	Frame_Shift_Del	329	0.072948328	No	0.575	Subclonal	.	.	.	.	Yes	No	No	No	Not tested
MP11-A2	Squamous	OR10J3	p.R316Afs*7	Shared	1	159283491	CCTTCACAACCTCT	C	Frame_Shift_Del	298	0.043624161	No	0.65	Subclonal	.	.	.	.	Yes	No	No	No	Not tested
MP11-B1	Chondroid	OR10K2	p.I65T	Private	1	158390463	A	G	Missense_Mutation	583	0.053173242	No	0.386	Subclonal	Neutral	Passenger	Neutral	passenger	Yes	No	No	No	Yes
MP11-A1	Squamous	OR11G2	p.G49A	Shared	14	20665640	G	C	Missense_Mutation	197	0.304568528	No	1	Clonal	Neutral	Passenger	Neutral	passenger	No	No	No	No	Not tested
MP11-A2	Squamous	OR11G2	p.G49A	Shared	14	20665640	G	C	Missense_Mutation	212	0.183962264	No	1	Clonal	Neutral	Passenger	Neutral	passenger	No	No	No	No	Not tested
MP11-B1	Chondroid	OR11G2	p.G49A	Shared	14	20665640	G	C	Missense_Mutation	234	0.333333333	No	1	Clonal	Neutral	Passenger	Neutral	passenger	Yes	No	No	No	Not tested
MP11-B1	Chondroid	OR6F1	p.R261L	Private	1	247875276	C	A	Missense_Mutation	630	0.082539683	No	1	Subclonal	Neutral	Passenger	Neutral	passenger	Yes	No	No	No	Not tested
MP11-B1	Chondroid	OSBP	p.E125Q	Private	11	59378052	C	G	Missense_Mutation	487	0.112936345	No	0.932	Subclonal	Disease_causing	Passenger	Neutral	passenger	Yes	No	No	No	Yes
MP11-A1	Squamous	OSBPL3	p.V547L	Shared	7	24874212	C	G	Missense_Mutation	572	0.251748252	No	1	Clonal	Disease_causing	Passenger	Neutral	passenger	No	No	No	No	Not tested
MP11-A2	Squamous	OSBPL3	p.V547L	Shared	7	24874212	C	G	Missense_Mutation	490	0.16122449	No	1	Clonal	Disease_causing	Passenger	Neutral	passenger	Yes	No	No	No	Not tested
MP11-B1	Chondroid	OSBPL3	p.V547L	Shared	7	24874212	C	G	Missense_Mutation	555	0.254054054	No	1	Clonal	Disease_causing	Passenger	Neutral	passenger	No	No	No	No	Not tested
MP11-B1	Chondroid	OTOG	p.T417P	Private	11	17580708	A	C	Missense_Mutation	119	0.12605042	No	0.914	Subclonal	Disease_causing	Passenger	Neutral	passenger	Yes	No	No	No	Yes
MP11-B1	Chondroid	PACRG	p.M203V	Private	6	163510434	A	G	Missense_Mutation	185	0.194594595	No	1	Subclonal	Disease_causing	Passenger	Neutral	passenger	No	No	No	No	Yes
MP11-B1	Chondroid	PARP2	p.G429A	Private	14	20824766	G	C	Missense_Mutation	212	0.136792453	No	0.992	Subclonal	Disease_causing	Passenger	Neutral	passenger	Yes	No	No	No	Yes
MP11-A2	Squamous	PCNT	p.A1889T	Private	21	47831652	G	A	Missense_Mutation	122	0.049180328	No	0.634	Subclonal	Neutral	Passenger	Neutral	passenger	No	No	No	No	Not tested
MP11-B1	Chondroid	PCSK9	p.H683N	Private	1	55529225	C	A	Missense_Mutation	134	0.164179104	No	0.862	Subclonal	Neutral	Passenger	Neutral	passenger	Yes	No	No	No	Not tested
MP11-B1	Chondroid	PDE2A	p.N674K	Private	11	72292424	G	C	Missense_Mutation	93	0.086021505	No	0.71	Subclonal	Disease_causing	Driver	Neutral	likely_pathogenic	No	No	No	No	Yes
MP11-A1	Squamous	PDYN	p.R212Q	Shared	20	1961099	C	T	Missense_Mutation	384	0.1015625	No	0.698	Subclonal	Disease_causing	Passenger	Disease_causing	likely_pathogenic	Yes	No	No	No	Not tested
MP11-A2	Squamous	PDYN	p.R212Q	Shared	20	1961099	C	T	Missense_Mutation	276	0.101449275	No	1	Subclonal	Disease_causing	Passenger	Disease_causing	likely_pathogenic	No	No	No	No	Not tested
MP11-B1	Chondroid	PDYN	p.R212Q	Shared	20	1961099	C	T	Missense_Mutation	285	0.122807018	No	0.522	Subclonal	Disease_causing	Passenger	Disease_causing	likely_pathogenic	Yes	No	No	No	Not tested
MP11-A1	Squamous	PDZD3	p.G456E	Shared	11	119059793	G	A	Missense_Mutation	274	0.167883212	No	1	Clonal	Neutral	Passenger	Neutral	passenger	Yes	No	No	No	Not tested
MP11-A2	Squamous	PDZD3	p.G456E	Shared	11	119059793	G	A	Missense_Mutation	219	0.118721461	No	1	Clonal	Neutral	Passenger	Neutral	passenger	No	No	No	No	Not tested
MP11-B1	Chondroid	PDZD3	p.G456E	Shared	11	119059793	G	A	Missense_Mutation	321	0.127725857	No	0.671	Clonal	Neutral	Passenger	Neutral	passenger	Yes	No	No	No	Not tested
MP11-A1	Squamous	PER3	p.Q590H	Shared	1	7880537	A	T	Missense_Mutation	400	0.465	No	1	Clonal	Neutral	Passenger	Neutral	passenger	Yes	No	No	No	Not tested
MP11-A1	Squamous	PER3	p.K158N	Shared	1	7848188	G	C	Missense_Mutation	458	0.150655022	No	1	Clonal	Disease_causing	Passenger	Neutral	passenger	Yes	No	No	No	Not tested

Supplementary Table S2
Page 4 of 16

MP11-A2	Squamous	PER3	p.Q590H	Shared	1	7880537	A	T	Missense_Mutation	[illegible]	[illegible]	No	1	Clonal	Neutral	Passenger	Neutral	passenger	No	No	No	No	Not tested
MP11-A2	Squamous	PER3	p.K158N	Shared	1	7848188	G	C	Missense_Mutation	399	[illegible]	No	1	Clonal	Disease_causing	Passenger	Neutral	passenger	No	No	No	No	Not tested
MP11-B1	Chondroid	PER3	p.Q590H	Shared	1	7880537	A	T	Missense_Mutation	240	0.125	No	0.656	Clonal	Neutral	Passenger	Neutral	passenger	Yes	No	No	No	Not tested
MP11-A1	Squamous	PEX13	p.Q352E	Shared	2	61275747	C	G	Missense_Mutation	397	0.186397985	No	1	Clonal	Disease_causing	Passenger	Neutral	passenger	No	No	No	No	Not tested
MP11-A2	Squamous	PEX13	p.Q352E	Shared	2	61275747	C	G	Missense_Mutation	431	0.106728538	No	1	Clonal	Disease_causing	Passenger	Neutral	passenger	No	No	No	No	Not tested
MP11-B1	Chondroid	PEX13	p.Q352E	Shared	2	61275747	C	G	Missense_Mutation	418	0.16507177	No	1	Clonal	Disease_causing	Passenger	Neutral	passenger	No	No	No	No	Not tested
MP11-A1	Squamous	PICALM	p.Y44*	Shared	11	85742652	G	C	Nonsense_Mutation	114	0.157894737	No	1	Clonal	Neutral	.	.	likely_pathogenic	Yes	Yes	No	No	Not tested
MP11-A2	Squamous	PICALM	p.Y44*	Shared	11	85742652	G	C	Nonsense_Mutation	125	0.2	No	1	Clonal	Neutral	.	.	likely_pathogenic	No	Yes	No	No	Not tested
MP11-B1	Chondroid	PICALM	p.Y44*	Shared	11	85742652	G	C	Nonsense_Mutation	171	0.257309942	No	1	Clonal	Neutral	.	.	likely_pathogenic	Yes	Yes	No	No	Not tested
MP11-B1	Chondroid	PIGG	p.A567T	Private	4	517332	G	A	Missense_Mutation	230	0.339130435	No	1	Subclonal	Disease_causing	Passenger	Neutral	passenger	Yes	No	No	No	Yes
MP11-B1	Chondroid	PLEKHM2	p.L723V	Private	1	16056383	C	G	Missense_Mutation	85	0.141176471	No	0.6	Subclonal	Disease_causing	Passenger	Neutral	passenger	Yes	No	No	No	Yes
MP11-A1	Squamous	PPOX	p.R113S	Shared	1	161137785	G	T	Missense_Mutation	153	0.261437908	No	1	Clonal	Disease_causing	Passenger	Disease_causing	likely_pathogenic	Yes	No	No	No	Not tested
MP11-A2	Squamous	PPOX	p.R113S	Shared	1	161137785	G	T	Missense_Mutation	80	0.1625	No	1	Clonal	Disease_causing	Passenger	Disease_causing	likely_pathogenic	Yes	No	No	No	Not tested
MP11-A1	Squamous	PPP1R27	p.V72M	Shared	17	79792506	C	T	Missense_Mutation	219	0.173515982	No	1	Clonal	Disease_causing	Passenger	Neutral	passenger	No	No	No	No	Not tested
MP11-A2	Squamous	PPP1R27	p.V72M	Shared	17	79792506	C	T	Missense_Mutation	128	0.1484375	No	1	Clonal	Disease_causing	Passenger	Neutral	passenger	No	No	No	No	Not tested
MP11-A1	Squamous	PPP1R3F	p.R498C	Shared	X	49142644	C	T	Missense_Mutation	139	0.100719424	No	0.793	Clonal	Disease_causing	Passenger	Neutral	passenger	Yes	No	No	No	Not tested
MP11-A2	Squamous	PPP1R3F	p.R498C	Shared	X	49142644	C	T	Missense_Mutation	99	0.121212121	No	1	Clonal	Disease_causing	Passenger	Neutral	passenger	Yes	No	No	No	Not tested
MP11-B1	Chondroid	PPP1R3F	p.R498C	Shared	X	49142644	C	T	Missense_Mutation	139	0.258992806	No	1	Clonal	Disease_causing	Passenger	Neutral	passenger	Yes	No	No	No	Not tested
MP11-B1	Chondroid	PRELP	p.A338V	Private	1	203455873	C	T	Missense_Mutation	813	0.056580566	No	0.467	Subclonal	Neutral	Passenger	Neutral	passenger	Yes	No	No	No	Not tested
MP11-A1	Squamous	PRKDC	p.L1796F	Shared	8	48777299	G	A	Missense_Mutation	110	0.281818182	No	1	Clonal	Disease_causing	Passenger	Neutral	passenger	Yes	No	No	No	Not tested
MP11-A2	Squamous	PRKDC	p.L1796F	Shared	8	48777299	G	A	Missense_Mutation	109	0.19266055	No	1	Clonal	Disease_causing	Passenger	Neutral	passenger	Yes	No	No	No	Not tested
MP11-B1	Chondroid	PRR12	p.E1712Q	Private	19	50119113	G	C	Missense_Mutation	278	0.115107914	No	0.605	Subclonal	Disease_causing	Passenger	.	passenger	Yes	No	No	No	Yes
MP11-B1	Chondroid	PRSS21	p.D182E	Private	16	2868966	T	G	Missense_Mutation	81	0.172839506	No	0.908	Subclonal	Neutral	Passenger	Disease_causing	passenger	Yes	No	No	No	Yes
MP11-A1	Squamous	QPRT	p.L6Q	Shared	16	29705988	T	A	Missense_Mutation	322	0.263975155	No	1	Clonal	Neutral	Passenger	Neutral	passenger	Yes	No	No	No	Not tested
MP11-A2	Squamous	QPRT	p.L6Q	Shared	16	29705988	T	A	Missense_Mutation	211	0.132701422	No	1	Clonal	Neutral	Passenger	Neutral	passenger	No	No	No	No	Not tested
MP11-B1	Chondroid	QPRT	p.L6Q	Shared	16	29705988	T	A	Missense_Mutation	270	0.292592593	No	1	Clonal	Neutral	Passenger	Neutral	passenger	Yes	No	No	No	Not tested
MP11-A1	Squamous	RASL11B	p.V26G	Shared	4	53728751	T	G	Missense_Mutation	220	0.159090909	No	1	Clonal	Neutral	Passenger	Neutral	passenger	Yes	No	No	No	Not tested
MP11-A2	Squamous	RASL11B	p.V26G	Shared	4	53728751	T	G	Missense_Mutation	121	0.074380165	No	1	Clonal	Neutral	Passenger	Neutral	passenger	No	No	No	No	Not tested
MP11-B1	Chondroid	RBPMS2	p.A181G	Private	15	65040643	G	C	Missense_Mutation	58	0.120689655	No	0.634	Subclonal	Disease_causing	Passenger	Neutral	passenger	No	No	No	No	Yes
MP11-B1	Chondroid	RCBTB2	p.N232K	Private	13	49085993	G	C	Missense_Mutation	847	0.063754427	No	0.526	Subclonal	Disease_causing	Passenger	Disease_causing	likely_pathogenic	Yes	No	No	No	Not tested
MP11-A2	Squamous	RFC2	p.K99R	Private	7	73663378	T	C	Missense_Mutation	146	0.034246575	No	0.442	Subclonal	Disease_causing	Passenger	Neutral	passenger	No	No	No	No	Not tested
MP11-A2	Squamous	RFC4	p.A353G	Private	3	186507792	G	C	Missense_Mutation	833	0.050420168	No	0.953	Subclonal	Disease_causing	Passenger	Neutral	passenger	No	No	No	No	Not tested
MP11-A1	Squamous	RFX1	p.Q263L	Shared	19	14090305	T	A	Missense_Mutation	83	0.337349398	No	1	Clonal	Neutral	Passenger	Neutral	passenger	Yes	No	No	No	Not tested
MP11-A2	Squamous	RFX1	p.Q263L	Shared	19	14090305	T	A	Missense_Mutation	60	0.216666667	No	1	Clonal	Neutral	Passenger	Neutral	passenger	Yes	No	No	No	Not tested
MP11-B1	Chondroid	RGS22	p.E414Q	Private	8	101075756	C	G	Missense_Mutation	354	0.110169492	No	1	Subclonal	Neutral	Passenger	Neutral	passenger	Yes	No	No	No	Yes
MP11-B1	Chondroid	RIBC1	p.Q43R	Private	X	53454961	A	G	Missense_Mutation	197	0.137055838	No	0.72	Subclonal	Neutral	Passenger	Neutral	passenger	Yes	No	No	No	Yes
MP11-B1	Chondroid	RPRD2	p.Y1167C	Private	1	150444924	A	G	Missense_Mutation	420	0.095238095	No	0.691	Subclonal	Disease_causing	Passenger	Neutral	passenger	Yes	No	No	No	Yes
MP11-B1	Chondroid	RPS6KA2	p.L531P	Private	6	166843954	A	G	Missense_Mutation	102	0.147058824	No	0.772	Subclonal	Disease_causing	Passenger	Neutral	passenger	Yes	No	No	No	Not tested
MP11-A1	Squamous	RTP2	p.D146H	Shared	3	187416528	C	G	Missense_Mutation	753	0.13811421	No	1	Clonal	Neutral	Passenger	Neutral	passenger	No	No	No	No	Not tested
MP11-A2	Squamous	RTP2	p.D146H	Shared	3	187416528	C	G	Missense_Mutation	434	0.115207373	No	1	Clonal	Neutral	Passenger	Neutral	passenger	No	No	No	No	Not tested
MP11-B1	Chondroid	RTP2	p.D146H	Shared	3	187416528	C	G	Missense_Mutation	518	0.096525097	No	1	Clonal	Neutral	Passenger	Neutral	passenger	No	No	No	No	Not tested
MP11-B1	Chondroid	RYR2	p.X187_splice	Private	1	237540714	CGTGTCCTCTGAAAGGTACTTGGTAA	C	Splice_Site	333	0.051051051	No	0.625	Subclonal	.	.	.	.	Yes	No	No	No	Yes
MP11-B1	Chondroid	SAAL1	p.H180R	Private	11	18111772	T	C	Missense_Mutation	202	0.094059406	No	0.682	Subclonal	Neutral	Passenger	Neutral	passenger	Yes	No	No	No	Yes
MP11-A1	Squamous	SACS	p.R3875H	Private	13	23906391	C	T	Missense_Mutation	316	0.117088608	No	0.922	Clonal	Disease_causing	Passenger	Disease_causing	likely_pathogenic	Yes	No	No	No	Not tested
MP11-A1	Squamous	SALL3	p.V1272F	Shared	18	76757233	G	T	Missense_Mutation	195	0.205128205	No	1	Clonal	Neutral	Passenger	Neutral	passenger	Yes	No	No	No	Not tested
MP11-A2	Squamous	SALL3	p.V1272F	Shared	18	76757233	G	T	Missense_Mutation	170	0.129411765	No	1	Clonal	Neutral	Passenger	Neutral	passenger	No	No	No	No	Not tested
MP11-B1	Chondroid	SALL3	p.V1272F	Shared	18	76757233	G	T	Missense_Mutation	173	0.202312139	No	0.86	Clonal	Neutral	Passenger	Neutral	passenger	No	No	No	No	Not tested
MP11-B1	Chondroid	SDR9C7	p.S312C	Private	12	57317625	T	A	Missense_Mutation	62	0.129032258	No	0.549	Subclonal	Disease_causing	Passenger	Disease_causing	likely_pathogenic	No	No	No	No	Yes
MP11-B1	Chondroid	SEL1L2	p.A519P	Private	20	13830890	C	G	Missense_Mutation	171	0.122807018	No	0.522	Subclonal	Disease_causing	Passenger	Neutral	passenger	Yes	No	No	No	Yes
MP11-A2	Squamous	SETDB1	p.P447L	Private	1	150921670	C	T	Missense_Mutation	211	0.056872038	No	0.847	Subclonal	Disease_causing	Passenger	Neutral	passenger	Yes	No	No	No	Not tested
MP11-A1	Squamous	SGPP2	p.G286A	Shared	2	223423274	G	C	Missense_Mutation	550	0.232727273	No	1	Clonal	Disease_causing	Passenger	.	passenger	No	No	No	No	Not tested
MP11-A2	Squamous	SGPP2	p.G286A	Shared	2	223423274	G	C	Missense_Mutation	357	0.196078431	No	1	Clonal	Disease_causing	Passenger	.	passenger	No	No	No	No	Not tested
MP11-B1	Chondroid	SH2B2	p.Y553C	Private	7	101960943	A	G	Missense_Mutation	244	0.094262295	No	0.684	Subclonal	Disease_causing	Passenger	Disease_causing	likely_pathogenic	Yes	No	No	No	Yes
MP11-B1	Chondroid	SLC12A4	p.T351R	Private	16	67985812	G	C	Missense_Mutation	164	0.091463415	No	0.48	Subclonal	Disease_causing	Passenger	Neutral	passenger	Yes	No	No	No	Not tested
MP11-A1	Squamous	SLC16A4	p.L160V	Shared	1	110923652	G	C	Missense_Mutation	173	0.046242775	No	0.272	Subclonal	Neutral	Passenger	Neutral	passenger	No	No	No	No	Not tested
MP11-A2	Squamous	SLC16A4	p.L160V	Shared	1	110923652	G	C	Missense_Mutation	226	0.044247788	No	0.571	Subclonal	Neutral	Passenger	Neutral	passenger	No	No	No	No	Not tested
MP11-A1	Squamous	SLC38A8	p.V284I	Shared	16	84050848	C	T	Missense_Mutation	220	0.168181818	No	1	Clonal	Neutral	Passenger	Neutral	passenger	Yes	No	No	No	Not tested
MP11-A2	Squamous	SLC38A8	p.V284I	Shared	16	84050848	C	T	Missense_Mutation	178	0.112359551	No	1	Clonal	Neutral	Passenger	Neutral	passenger	No	No	No	No	Not tested
MP11-B1	Chondroid	SLC38A8	p.V284I	Shared	16	84050848	C	T	Missense_Mutation	200	0.105	No	0.551	Clonal	Neutral	Passenger	Neutral	passenger	No	No	No	No	Not tested
MP11-B1	Chondroid	SLC41A1	p.E52Q	Private	1	205779416	C	G	Missense_Mutation	687	0.061135371	No	0.504	Subclonal	Disease_causing	Passenger	Neutral	passenger	Yes	No	No	No	Yes
MP11-B1	Chondroid	SLC7A11	p.K105*	Private	4	139157570	T	A	Nonsense_Mutation	235	0.131914894	No	0.693	Subclonal	Neutral	.	.	passenger	No	No	No	No	Not tested
MP11-A2	Squamous	SLC9C2	p.F339S	Private	1	173542351	A	G	Missense_Mutation	85	0.058823529	No	0.877	Subclonal	Neutral	Passenger	Neutral	passenger	Yes	No	No	No	Yes
MP11-B1	Chondroid	SLITRK3	p.E325K	Private	3	164907646	C	T	Missense_Mutation	231	0.164502165	No	1	Subclonal	Disease_causing	Passenger	Neutral	passenger	No	No	No	No	Not tested
MP11-B1	Chondroid	SLITRK5	p.T148S	Private	13	88328086	C	G	Missense_Mutation	322	0.170807453	No	1	Subclonal	Disease_causing	Passenger	Neutral	passenger	Yes	No	No	No	Not tested
MP11-A1	Squamous	SLTM	p.V558G	Shared	15	59185539	A	C	Missense_Mutation	221	0.357466063	No	1	Clonal	Disease_causing	Driver	Neutral	likely_pathogenic	No	No	No	No	Not tested
MP11-A2	Squamous	SLTM	p.V558G	Shared	15	59185539	A	C	Missense_Mutation	259	0.196911197	No	1	Clonal	Disease_causing	Driver	Neutral	likely_pathogenic	No	No	No	No	Not tested
MP11-B1	Chondroid	SLTM	p.V558G	Shared	15	59185539	A	C	Missense_Mutation	235	0.387234043	No	1	Clonal	Disease_causing	Driver	Neutral	likely_pathogenic	No	No	No	No	Not tested
MP11-B1	Chondroid	SLX4IP	p.Q379E	Private	20	10603935	C	G	Missense_Mutation	230	0.147826087	No	0.629	Subclonal	Neutral	Passenger	Neutral	passenger	Yes	No	No	No	Yes
MP11-A1	Squamous	SMC4	p.X869_splice	Shared	3	160146539	A	G	Splice_Site	190	0.231578947	No	1	Clonal	Disease_causing	.	.	passenger	No	No	No	No	Not tested
MP11-A2	Squamous	SMC4	p.X869_splice	Shared	3	160146539	A	G	Splice_Site	174	0.16091954	No	1	Clonal	Disease_causing	.	.	passenger	No	No	No	No	Not tested
MP11-B1	Chondroid	SOCS7	p.Q271E	Private	17	36517616	C	G	Missense_Mutation	316	0.148734177	No	1	Subclonal	Disease_causing	Driver	Neutral	likely_pathogenic	No	No	No	No	Yes
MP11-A1	Squamous	SPC24	p.R28P	Shared	19	11266370	C	G	Missense_Mutation	234	0.243589744	No	1	Clonal	Neutral	Passenger	.	passenger	Yes	No	No	No	Not tested
MP11-A2	Squamous	SPC24	p.R28P	Shared	19	11266370	C	G	Missense_Mutation	140	0.2	No	1	Clonal	Neutral	Passenger	.	passenger	Yes	No	No	No	Not tested
MP11-B1	Chondroid	SPTA1	p.Q2195E	Private	1	158587344	G	C	Missense_Mutation	177	0.101694915	No	0.737	Subclonal	Disease_causing	Passenger	Neutral	passenger	Yes	No	No	No	Yes
MP11-B1	Chondroid	STAC2	p.G335A	Private	17	37369375	C	G	Missense_Mutation	216	0.152777778	No	1	Subclonal	Disease_causing	Passenger	Disease_causing	likely_pathogenic	No	No	No	No	Yes
MP11-B1	Chondroid	STARD3NL	p.A127S	Private	7	38254704	G	T	Missense_Mutation	141	0.070921986	No	0.372	Subclonal	Disease_causing	Passenger	Neutral	passenger	No	No	No	No	Yes
MP11-B1	Chondroid	STK10	p.R636Q	Private	5	171509412	C	T	Missense_Mutation	165	0.163636364	No	0.859	Subclonal	Neutral	Passenger	Neutral	passenger	Yes	No	No	No	Not tested
MP11-B1	Chondroid	SV2B	p.R229G	Private	15	91795651	C	G	Missense_Mutation	158	0.17721519	No	0.931	Subclonal	Disease_causing	Passenger	Neutral	passenger	No	No	No	No	Yes
MP11-B1	Chondroid	SYBU	p.D361E	Private	8	110588044	G	C	Missense_Mutation	585	0.034188034	No	0.385	Subclonal	Disease_causing	Passenger	.	passenger	Yes	No	No	No	Yes
MP11-B1	Chondroid	SYNE1	p.M8374V	Private	6	152462464	T	C	Missense_Mutation	81	0.12345679	No	0.648	Subclonal	Disease_causing	Passenger	Neutral	passenger	No	No	No	No	Yes
MP11-B1	Chondroid	TAS2R13	p.N21K	Private	12	11061835	A	C	Missense_Mutation	154	0.058441558	No	0.307	Subclonal	Neutral	Passenger	Neutral	passenger	No	No	No	No	Yes
MP11-B1	Chondroid	TBC1D17	p.G149C	Private	19	50384650	G	T	Missense_Mutation	181	0.220994475	No	1	Subclonal	Neutral	Passenger	Neutral	passenger	Yes	No	No	No	Not tested
MP11-B1	Chondroid	TCTA	NA	Private	3	49449851	GGGCCAGTCATGGCGGAGTCCTGGTCTGGGCA	G	Missense_Mutation	203	0.054187192	No	0.339	Subclonal	.	.	.	.	No	No	No	Yes	Not tested
MP11-A1	Squamous	TENM3	p.Q234_V240del	Shared	4	183522261	CGTCCAGCTGCAGGACAGCTGG	C	In_Frame_Del	216	0.074074074	No	0.509	Subclonal	.	.	.	.	No	No	No	No	Not tested
MP11-A2	Squamous	TENM3	p.Q234_V240del	Shared	4	183522261	CGTCCAGCTGCAGGACAGCTGG	C	In_Frame_Del	194	0.077319588	No	0.997	Subclonal	.	.	.	.	No	No	No	No	Not tested
MP11-B1	Chondroid	TENM3	p.Q234_V240del	Shared	4	183522261	CGTCCAGCTGCAGGACAGCTGG	C	In_Frame_Del	172	0.139534884	No	0.733	Subclonal	.	.	.	.	No	No	No	No	Not tested
MP11-B1	Chondroid	TENM4	p.T1324A	Private	11	78423611	T	C	Missense_Mutation	351	0.0997151	No	0.823	Subclonal	Disease_causing	Passenger	Disease_causing	likely_pathogenic	Yes	No	No	No	Yes
MP11-A1	Squamous	TESC	p.L28Afs*48	Shared	12	117513105	CTTAAATCTCCGATGGAG	C	Frame_Shift_Del	75	0.293333333	No	1	Clonal	.	.	.	.	No	No	No	No	Not tested
MP11-A2	Squamous	TESC	p.L28Afs*48	Shared	12	117513105	CTTAAATCTCCGATGGAG	C	Frame_Shift_Del	83	0.144578313	No	1	Clonal	.	.	.	.	No	No	No	No	Not tested
MP11-B1	Chondroid	TESC	p.L28Afs*48	Shared	12	117513105	CTTAAATCTCCGATGGAG	C	Frame_Shift_Del	98	0.397959184	No	1	Clonal	.	.	.	.	No	No	No	No	Not tested
MP11-B1	Chondroid	TEX36	p.F18I	Private	10	127350546	A	T	Missense_Mutation	182	0.164835165	No	0.701	Subclonal	Disease_causing	Passenger	Neutral	passenger	No	No	No	No	Yes
MP11-B1	Chondroid	THAP11	p.E272V	Private	16	67877272	A	T	Missense_Mutation	632	0.120253165	No	0.632	Subclonal	Disease_causing	Passenger	Neutral	passenger	Yes	No	No	No	Yes
MP11-A1	Squamous	TIE1	p.R382Q	Shared	1	43774759	G	A	Missense_Mutation	173	0.225433526	No	1	Clonal	Disease_causing	Passenger	Neutral	passenger	Yes	No	No	No	Not tested
MP11-A2	Squamous	TIE1	p.R382Q	Shared	1	43774759	G	A	Missense_Mutation	110	0.145454545	No	1	Clonal	Disease_causing	Passenger	Neutral	passenger	No	No	No	No	Not tested
MP11-B1	Chondroid	TIE1	p.R382Q	Shared	1	43774759	G	A	Missense_Mutation	158	0.17721519	No	0.931	Clonal	Disease_causing	Passenger	Neutral	passenger	Yes	No	No	No	Not tested
MP11-B1	Chondroid	TMC8	p.L170I	Private	17	76128928	C	A	Missense_Mutation	230	0.186956522	No	0.982	Subclonal	Neutral	Passenger	Neutral	passenger	No	No	No	No	Yes
MP11-A1	Squamous	TMEM132D	p.V179L	Shared	12	130184788	C	A	Missense_Mutation	305	0.11147541	No	0.767	Clonal	Disease_causing	Passenger	Neutral	passenger	No	No	No	No	Not tested
MP11-A2	Squamous	TMEM132D	p.V179L	Shared	12	130184788	C	A	Missense_Mutation	233	0.094420601	No	1	Clonal	Disease_causing	Passenger	Neutral	passenger	No	No	No	No	Not tested
MP11-A1	Squamous	TNFRSF21	p.T161A	Shared	6	47253947	T	C	Missense_Mutation	729	0.207133059	No	1	Clonal	Disease_causing	Passenger	Neutral	passenger	Yes	No	No	No	Not tested
MP11-A2	Squamous	TNFRSF21	p.T161A	Shared	6	47253947	T	C	Missense_Mutation	541	0.199630314	No	1	Clonal	Disease_causing	Passenger	Neutral	passenger	Yes	No	No	No	Not tested
MP11-A1	Squamous	TP53	p.C229Yfs*10	Shared	17	7577593	TAC	T	Frame_Shift_Del	99	0.404040404	No	1	Clonal	.	.	.	.	No	Yes	Yes	Yes	Not tested
MP11-A2	Squamous	TP53	p.C229Yfs*10	Shared	17	7577593	TAC	T	Frame_Shift_Del	102	0.18627451	No	1	Clonal	.	.	.	.	No	Yes	Yes	Yes	Not tested
MP11-B1	Chondroid	TP53	p.C229Yfs*10	Shared	17	7577593	TAC	T	Frame_Shift_Del	134	0.432835821	No	1	Clonal	.	.	.	.	No	Yes	Yes	Yes	Not tested
MP11-A1	Squamous	TRMT2A	p.A553P	Shared	22	20100307	C	G	Missense_Mutation	169	0.106508876	No	0.839	Clonal	Disease_causing	Passenger	Neutral	passenger	Yes	No	No	No	Not tested
MP11-A2	Squamous	TRMT2A	p.A553P	Shared	22	20100307	C	G	Missense_Mutation	165	0.060606061	No	0.903	Clonal	Disease_causing	Passenger	Neutral	passenger	Yes	No	No	No	Not tested
MP11-A1	Squamous	UBN1	p.K447M	Shared	16	4920545	A	T	Missense_Mutation	240	0.254166667	No	1	Clonal	Disease_causing	Passenger	Neutral	passenger	Yes	No	No	No	Not tested
MP11-A2	Squamous	UBN1	p.K447M	Shared	16	4920545	A	T	Missense_Mutation	153	0.222222222	No	1	Clonal	Disease_causing	Passenger	Neutral	passenger	Yes	No	No	No	Not tested
MP11-B1	Chondroid	USH2A	p.G1301R	Private	1	216371837	C	G	Missense_Mutation	211	0.142180095	No	1	Subclonal	Disease_causing	Passenger	Neutral	passenger	Yes	No	No	No	Yes
MP11-B1	Chondroid	USP9X	p.G1923A	Private	X	41075588	G	C	Missense_Mutation	382	0.123036649	No	0.646	Subclonal	Disease_causing	Passenger	Neutral	passenger	Yes	No	Yes	No	Not tested
MP11-B1	Chondroid	UTP6	p.D269H	Private	17	30207754	C	G	Missense_Mutation	206	0.072815534	No	0.528	Subclonal	Disease_causing	Passenger	Neutral	passenger	No	No	No	No	Yes
MP11-B1	Chondroid	WDR7	p.T1076R	Private	18	54483298	C	G	Missense_Mutation	95	0.2	No	0.85	Subclonal	Neutral	Passenger	Neutral	passenger	No	No	No	No	Yes
MP11-A1	Squamous	WDR87	p.Q233E	Shared	19	38385529	G	C	Missense_Mutation	227	0.22907489	No	1	Clonal	Neutral	Passenger	Neutral	passenger	Yes	No	No	No	Not tested
MP11-A2	Squamous	WDR87	p.Q233E	Shared	19	38385529	G	C	Missense_Mutation	165	0.175757576	No	1	Clonal	Neutral	Passenger	Neutral	passenger	Yes	No	No	No	Not tested
MP11-A2	Squamous	ZBTB4	p.T8M	Private	17	7370098	G	A	Missense_Mutation	112	0.044642857	No	0.576	Subclonal	Disease_causing	Passenger	Neutral	passenger	Yes	No	No	No	Not tested
MP11-B1	Chondroid	ZC3HAV1	p.Q278*	Private	7	138764855	G	A	Nonsense_Mutation	529	0.376181474	No	1	Subclonal	Neutral	.	.	passenger	Yes	No	No	No	Not tested
MP11-A1	Squamous	ZFP91	p.C507W	Private	11	58384987	C	G	Missense_Mutation	360	0.038888889	No	0.267	Subclonal	Disease_causing	Passenger	Neutral	passenger	Yes	No	No	No	Not tested
MP11-A1	Squamous	ZMIZ2	p.P714A	Shared	7	44805076	C	G	Missense_Mutation	175	0.331428571	No	1	Clonal	Disease_causing	Passenger	Neutral	passenger	Yes	No	No	No	Not tested
MP11-A2	Squamous	ZMIZ2	p.P714A	Shared	7	44805076	C	G	Missense_Mutation	118	0.144067797	No	1	Clonal	Disease_causing	Passenger	Neutral	passenger	Yes	No	No	No	Not tested
MP11-B1	Chondroid	ZMIZ2	p.P714A	Shared	7	44805076	C	G	Missense_Mutation	113	0.194690265	No	1	Clonal	Disease_causing	Passenger	Neutral	passenger	No	No	No	No	Not tested
MP11-A1	Squamous	ZMYM6	p.L823V	Shared	1	35454216	G	C	Missense_Mutation	155	0.161290323	No	1	Clonal	Disease_causing	Passenger	Neutral	passenger	Yes	No	No	No	Not tested
MP11-A2	Squamous	ZMYM6	p.L823V	Shared	1	35454216	G	C	Missense_Mutation	172	0.151162791	No	1	Clonal	Disease_causing	Passenger	Neutral	passenger	No	No	No	No	Not tested
MP11-B1	Chondroid	ZMYM6	p.L823V	Shared	1	35454216	G	C	Missense_Mutation	168	0.130952381	No	0.688	Clonal	Disease_causing	Passenger	Neutral	passenger	Yes	No	No	No	Not tested
MP11-A1	Squamous	ZNF391	p.N168K	Private	6	27368653	T	A	Missense_Mutation	188	0.058510638	No	0.461	Subclonal	Neutral	Passenger	Neutral	passenger	Yes	No	No	No	Not tested
MP11-B1	Chondroid	ZNF469	p.P447Lfs*55	Private	16	88495214	GC	G	Frame_Shift_Del	114	0.157894737	No	0.829	Subclonal	.	.	.	.	Yes	No	No	No	Not tested
MP11-A1	Squamous	ZNF513	p.H434D	Shared	2	27600738	G	C	Missense_Mutation	396	0.113636364	No	1	Clonal	Disease_causing	Passenger	Disease_causing	likely_pathogenic	No	No	No	No	Not tested
MP11-A2	Squamous	ZNF513	p.H434D	Shared	2	27600738	G	C	Missense_Mutation	286	0.041958042	No	0.667	Clonal	Disease_causing	Passenger	Disease_causing	likely_pathogenic	No	No	No	No	Not tested
MP11-B1	Chondroid	ZNF513	p.H434D	Shared	2	27600738	G	C	Missense_Mutation	561	0.135472371	No	1	Clonal	Disease_causing	Passenger	Disease_causing	likely_pathogenic	No	No	No	No	Not tested
MP11-B1	Chondroid	ZNF536	p.K404N	Private	19	30935681	G	T	Missense_Mutation	167	0.107784431	No	0.566	Subclonal	Disease_causing	Passenger	Neutral	passenger	Yes	No	No	No	Yes
MP11-B1	Chondroid	ZNF572	p.H346L	Private	8	125989547	A	T	Missense_Mutation	554	0.055956679	No	0.63	Subclonal	Disease_causing	Passenger	Neutral	passenger	Yes	No	No	No	Yes
MP11-A2	Squamous	ZNF804B	p.D481H	Private	7	88963737	G	C	Missense_Mutation	369	0.035230352	No	0.454	Subclonal	Disease_causing	Passenger	Neutral	passenger	No	No	No	No	Yes
MP11-B1	Chondroid	ZNF837	p.R257Q	Private	19	58879930	C	T	Missense_Mutation	171	0.152046784	No	0.798	Subclonal	Neutral	Passenger	Neutral	passenger	Yes	No	No	No	Yes
MP15-1	Chondroid	AAMP	p.R391H	Shared	2	219129800	C	T	Missense_Mutation	198	0.267676768	No	1	Clonal	Neutral	Passenger	Neutral	passenger	Yes	No	No	No	Not tested
MP15-2	Ductal	AAMP	p.R391H	Shared	2	219129800	C	T	Missense_Mutation	191	0.387434555	No	1	Clonal	Neutral	Passenger	Neutral	passenger	Yes	No	No	No	Not tested
MP15-1	Chondroid	ABCA7	p.T748P	Shared	19	1047626	A	C	Missense_Mutation	223	0.31838565	No	1	Clonal	Neutral	Passenger	Neutral	passenger	No	No	No	No	Not tested
MP15-2	Ductal	ABCA7	p.T748P	Shared	19	1047626	A	C	Missense_Mutation	182	0.197802198	No	1	Clonal	Neutral	Passenger	Neutral	passenger	No	No	No	No	Not tested
MP15-1	Chondroid	ABCF3	p.I298L	Private	3	183906604	A	C	Missense_Mutation	612	0.116013072	No	1	Clonal	Disease_causing	Passenger	Disease_causing	likely_pathogenic	Yes	No	No	No	Not tested
MP15-1	Chondroid	ACOT8	p.E276K	Shared	20	44472181	C	T	Missense_Mutation	458	0.262008734	No	1	Clonal	Disease_causing	Passenger	.	passenger	No	No	No	No	Yes
MP15-2	Ductal	ACOT8	p.E276K	Shared	20	44472181	C	T	Missense_Mutation	480	0.29375	No	1	Clonal	Disease_causing	Passenger	.	passenger	No	No	No	No	Yes
MP15-2	Ductal	ADAM15	p.V144G	Private	1	155026801	T	G	Missense_Mutation	519	0.05973025	No	0.411	Subclonal	Disease_causing	Passenger	Neutral	passenger	No	No	No	No	Not tested
MP15-1	Chondroid	AP5M1	p.W198*	Shared	14	57741481	G	A	Nonsense_Mutation	224	0.401785714	No	1	Clonal	Neutral	.	.	passenger	Yes	No	No	No	Not tested
MP15-2	Ductal	AP5M1	p.W198*	Shared	14	57741481	G	A	Nonsense_Mutation	308	0.331168831	No	1	Clonal	Neutral	.	.	passenger	No	No	No	No	Not tested
MP15-2	Ductal	AP5Z1	p.H105D	Private	7	4821332	C	G	Missense_Mutation	710	0.150704225	No	1	Clonal	Neutral	Passenger	Neutral	passenger	No	No	No	No	Not tested
MP15-1	Chondroid	ARHGAP20	p.X472_splice	Shared	11	110454462	C	A	Splice_Site	58	0.206896552	No	1	Clonal	Disease_causing	.	.	passenger	No	No	No	No	Not tested
MP15-2	Ductal	ARHGAP20	p.X472_splice	Shared	11	110454462	C	A	Splice_Site	126	0.063492063	No	0.374	Subclonal	Disease_causing	.	.	passenger	No	No	No	No	Not tested
MP15-1	Chondroid	ARHGAP29	p.Y868H	Shared	1	94643602	A	G	Missense_Mutation	275	0.156363636	No	1	Clonal	Disease_causing	Passenger	Neutral	passenger	No	No	No	No	Not tested
MP15-2	Ductal	ARHGAP29	p.Y868H	Shared	1	94643602	A	G	Missense_Mutation	360	0.063888889	No	0.376	Subclonal	Disease_causing	Passenger	Neutral	passenger	No	No	No	No	Not tested
MP15-1	Chondroid	ATP13A3	p.R634H	Shared	3	194158138	C	T	Missense_Mutation	515	0.572815534	No	1	Clonal	Disease_causing	Passenger	Neutral	passenger	Yes	No	No	No	Yes
MP15-2	Ductal	ATP13A3	p.R634H	Shared	3	194158138	C	T	Missense_Mutation	888	0.593468468	No	1	Clonal	Disease_causing	Passenger	Neutral	passenger	Yes	No	No	No	Yes
MP15-1	Chondroid	ATP1A3	p.P532T	Private	19	42482833	G	T	Missense_Mutation	415	0.055421687	No	0.437	Subclonal	Disease_causing	Passenger	Neutral	passenger	Yes	No	No	No	Yes
MP15-2	Ductal	ATP9B	p.V987M	Private	18	77119409	G	A	Missense_Mutation	288	0.03125	No	0.184	Subclonal	Disease_causing	Passenger	Neutral	passenger	No	No	No	No	Not tested
MP15-1	Chondroid	BCLAF1	p.R69P	Shared	6	136599813	C	G	Missense_Mutation	633	0.085308057	No	0.928	Clonal	Disease_causing	Driver	Neutral	likely_pathogenic	No	No	No	Yes	Not tested
MP15-2	Ductal	BCLAF1	p.R69P	Shared	6	136599813	C	G	Missense_Mutation	1071	0.118580766	No	1	Clonal	Disease_causing	Driver	Neutral	likely_pathogenic	No	No	No	Yes	Not tested
MP15-1	Chondroid	BEND3	p.G637V	Private	6	107390485	C	A	Missense_Mutation	708	0.103107345	No	0.915	Clonal	Disease_causing	Passenger	Neutral	passenger	No	No	No	No	Not tested
MP15-1	Chondroid	C16orf11	p.P262A	Shared	16	614078	C	G	Missense_Mutation	248	0.213709677	No	1	Clonal	Disease_causing	Passenger	Neutral	passenger	No	No	No	No	Not tested
MP15-2	Ductal	C16orf11	p.P262A	Shared	16	614078	C	G	Missense_Mutation	222	0.036036036	No	0.212	Subclonal	Disease_causing	Passenger	Neutral	passenger	No	No	No	No	Not tested
MP15-2	Ductal	CACNA1S	p.Y299*	Private	1	201058389	G	C	Nonsense_Mutation	346	0.101156069	No	0.697	Subclonal	Neutral	.	.	passenger	No	No	No	No	Not tested
MP15-2	Ductal	CCAR1	p.Q97H	Private	10	70497646	G	C	Missense_Mutation	250	0.1	No	0.689	Subclonal	Disease_causing	Passenger	Neutral	passenger	No	No	No	No	Not tested
MP15-1	Chondroid	CCDC129	p.K480Rfs*24	Shared	7	31682343	AAAGCCAAG	A	Frame_Shift_Del	522	0.291187739	No	1	Clonal	.	.	.	.	No	No	No	No	Not tested
MP15-2	Ductal	CCDC129	p.K480Rfs*24	Shared	7	31682343	AAAGCCAAG	A	Frame_Shift_Del	810	0.35308642	No	1	Clonal	.	.	.	.	No	No	No	No	Not tested
MP15-1	Chondroid	CDKL5	p.H32P	Shared	X	18528970	A	C	Missense_Mutation	169	0.236686391	No	1	Clonal	Disease_causing	Driver	Neutral	likely_pathogenic	No	No	No	No	Yes
MP15-2	Ductal	CDKL5	p.H32P	Shared	X	18528970	A	C	Missense_Mutation	304	0.358552632	No	1	Clonal	Disease_causing	Driver	Neutral	likely_pathogenic	No	No	No	No	Yes
MP15-1	Chondroid	CHD7	p.Q555*	Shared	8	61655654	C	T	Nonsense_Mutation	63	0.285714286	No	1	Clonal	Neutral	.	.	likely_pathogenic	Yes	No	No	No	Not tested
MP15-2	Ductal	CHD7	p.Q555*	Shared	8	61655654	C	T	Nonsense_Mutation	97	0.24742268	No	1	Clonal	Neutral	.	.	likely_pathogenic	No	No	No	No	Not tested
MP15-1	Chondroid	CHRNA3	p.M455I	Shared	15	78893619	C	G	Missense_Mutation	229	0.231441048	No	1	Clonal	Disease_causing	Passenger	Disease_causing	likely_pathogenic	No	No	No	No	Not tested
MP15-2	Ductal	CHRNA3	p.M455I	Shared	15	78893619	C	G	Missense_Mutation	306	0.055555556	No	0.327	Subclonal	Disease_causing	Passenger	Disease_causing	likely_pathogenic	No	No	No	No	Not tested
MP15-2	Ductal	CHST2	p.D427Y	Private	3	142840937	G	T	Missense_Mutation	455	0.048351648	No	0.381	Subclonal	Disease_causing	Passenger	Disease_causing	likely_pathogenic	Yes	No	No	No	Not tested
MP15-1	Chondroid	COL13A1	p.L75Q	Shared	10	71562403	T	A	Missense_Mutation	279	0.222222222	No	1	Clonal	Disease_causing	Passenger	Disease_causing	likely_pathogenic	No	No	No	No	Not tested
MP15-2	Ductal	COL13A1	p.L75Q	Shared	10	71562403	T	A	Missense_Mutation	251	0.227091633	No	1	Clonal	Disease_causing	Passenger	Disease_causing	likely_pathogenic	No	No	No	No	Not tested
MP15-2	Ductal	COL18A1	p.G1205R	Private	21	46925331	G	C	Missense_Mutation	718	0.135097493	No	0.795	Subclonal	Disease_causing	Passenger	Disease_causing	likely_pathogenic	No	No	No	No	Not tested
MP15-1	Chondroid	COL6A1	p.H181Q	Shared	21	47406554	C	G	Missense_Mutation	239	0.267782427	No	1	Clonal	Disease_causing	Passenger	Disease_causing	likely_pathogenic	No	No	No	No	Yes
MP15-2	Ductal	COL6A1	p.H181Q	Shared	21	47406554	C	G	Missense_Mutation	252	0.341269841	No	1	Clonal	Disease_causing	Passenger	Disease_causing	likely_pathogenic	No	No	No	No	Yes
MP15-2	Ductal	COPS7A	p.E4A	Private	12	6833833	A	C	Missense_Mutation	105	0.238095238	No	1	Clonal	Disease_causing	Passenger	.	passenger	No	No	No	No	Not tested
MP15-2	Ductal	CPEB1	p.X64_splice	Private	15	83240283	C	A	Splice_Site	278	0.151079137	No	0.889	Clonal	Disease_causing	.	.	passenger	No	No	No	No	Not tested
MP15-1	Chondroid	CROT	p.W142_P143delinsC	Shared	7	86990806	GGCC	G	In_Frame_Del	307	0.260586319	No	1	Clonal	.	.	.	.	No	No	No	No	Not tested
MP15-2	Ductal	CROT	p.W142_P143delinsC	Shared	7	86990806	GGCC	G	In_Frame_Del	426	0.23943662	No	1	Clonal	.	.	.	.	No	No	No	No	Not tested
MP15-1	Chondroid	DAAM2	p.D659V	Shared	6	39855284	A	T	Missense_Mutation	389	0.151670951	No	1	Clonal	Disease_causing	Passenger	Neutral	passenger	No	No	No	No	Not tested
MP15-2	Ductal	DAAM2	p.D659V	Shared	6	39855284	A	T	Missense_Mutation	419	0.045346062	No	0.312	Subclonal	Disease_causing	Passenger	Neutral	passenger	No	No	No	No	Not tested
MP15-1	Chondroid	DHX9	p.Y554*	Shared	1	182841576	T	G	Nonsense_Mutation	161	0.217391304	No	1	Clonal	Neutral	.	.	passenger	No	No	No	No	Not tested
MP15-2	Ductal	DHX9	p.Y554*	Shared	1	182841576	T	G	Nonsense_Mutation	296	0.273648649	No	1	Clonal	Neutral	.	.	passenger	No	No	No	No	Not tested
MP15-1	Chondroid	DNAH1	p.S1490L	Shared	3	52393993	C	T	Missense_Mutation	126	0.126984127	No	0.873	Clonal	Disease_causing	Driver	Neutral	likely_pathogenic	Yes	No	No	No	Not tested
MP15-1	Chondroid	DNAH1	p.H3044Y	Shared	3	52422309	C	T	Missense_Mutation	371	0.210242588	No	1	Clonal	Disease_causing	Passenger	Neutral	passenger	Yes	No	No	No	Not tested
MP15-2	Ductal	DNAH1	p.S1490L	Shared	3	52393993	C	T	Missense_Mutation	123	0.06504065	No	0.383	Subclonal	Disease_causing	Driver	Neutral	likely_pathogenic	Yes	No	No	No	Not tested
MP15-2	Ductal	DNAH1	p.H3044Y	Shared	3	52422309	C	T	Missense_Mutation	354	0.042372881	No	0.249	Subclonal	Disease_causing	Passenger	Neutral	passenger	Yes	No	No	No	Not tested
MP15-2	Ductal	DNAH14	p.R60T	Private	1	225142762	G	C	Missense_Mutation	397	0.070528967	No	0.556	Subclonal	Disease_causing	Passenger	Neutral	passenger	No	No	No	No	Yes
MP15-1	Chondroid	DNAJC6	p.*971Yext*13	Shared	1	65878708	A	T	Nonstop_Mutation	200	0.235	No	1	Clonal	Neutral	.	.	passenger	No	No	No	No	Not tested
MP15-2	Ductal	DNAJC6	p.*971Yext*13	Shared	1	65878708	A	T	Nonstop_Mutation	351	0.276353276	No	1	Clonal	Neutral	.	.	passenger	No	No	No	No	Not tested
MP15-2	Ductal	ELTD1	p.S262A	Private	1	79402073	A	C	Missense_Mutation	272	0.080882353	No	0.476	Subclonal	Neutral	Passenger	Neutral	passenger	No	No	No	No	Not tested
MP15-1	Chondroid	EPHB1	p.Q715H	Shared	3	134920330	G	C	Missense_Mutation	357	0.176470588	No	1	Clonal	Disease_causing	Passenger	Disease_causing	likely_pathogenic	Yes	No	No	No	Not tested
MP15-2	Ductal	EPHB1	p.Q715H	Shared	3	134920330	G	C	Missense_Mutation	369	0.159891599	No	1	Clonal	Disease_causing	Passenger	Disease_causing	likely_pathogenic	Yes	No	No	No	Not tested
MP15-1	Chondroid	EXOSC5	p.F223Lfs*47	Shared	19	41892579	A	AACGGAAG	Frame_Shift_Ins	487	0.156057495	No	1	Clonal	.	.	.	.	No	No	No	No	Not tested
MP15-2	Ductal	EXOSC5	p.F223Lfs*47	Shared	19	41892579	A	AACGGAAG	Frame_Shift_Ins	412	0.225728155	No	1	Clonal	.	.	.	.	No	No	No	No	Not tested
MP15-2	Ductal	FAT1	p.S1566F	Private	4	187549421	G	A	Missense_Mutation	360	0.077777778	No	0.458	Subclonal	Disease_causing	Passenger	Neutral	passenger	Yes	Yes	No	Yes	Not tested
MP15-1	Chondroid	FAT3	p.I2233T	Shared	11	92532877	T	C	Missense_Mutation	211	0.251184834	No	1	Clonal	Disease_causing	Passenger	Neutral	passenger	No	No	No	No	Not tested
MP15-2	Ductal	FAT3	p.I2233T	Shared	11	92532877	T	C	Missense_Mutation	326	0.27607362	No	1	Clonal	Disease_causing	Passenger	Neutral	passenger	No	No	No	No	Not tested
MP15-2	Ductal	FDXACB1	p.Q601H	Private	11	111745718	C	G	Missense_Mutation	480	0.085416667	No	0.503	Subclonal	Neutral	Passenger	Neutral	passenger	No	No	No	No	Not tested
MP15-2	Ductal	GABRG2	p.Q438E	Private	5	161580138	C	G	Missense_Mutation	136	0.147058824	No	0.866	Clonal	Disease_causing	Passenger	Disease_causing	likely_pathogenic	Yes	No	No	No	Not tested
MP15-1	Chondroid	GANC	p.L272P	Shared	15	42602573	T	C	Missense_Mutation	161	0.204968944	No	1	Clonal	Disease_causing	Passenger	Disease_causing	likely_pathogenic	No	No	No	No	Not tested
MP15-2	Ductal	GANC	p.L272P	Shared	15	42602573	T	C	Missense_Mutation	212	0.056603774	No	0.333	Subclonal	Disease_causing	Passenger	Disease_causing	likely_pathogenic	No	No	No	No	Not tested
MP15-2	Ductal	GPR98	p.E3088D	Private	5	90012363	G	C	Missense_Mutation	347	0.031700288	No	0.187	Subclonal	Disease_causing	Passenger	Neutral	passenger	No	No	No	No	Not tested
MP15-1	Chondroid	GSK3A	p.A51P	Shared	19	42746467	C	G	Missense_Mutation	57	0.210526316	No	1	Clonal	Disease_causing	Passenger	Disease_causing	likely_pathogenic	No	No	No	No	Not tested
MP15-2	Ductal	GSK3A	p.A51P	Shared	19	42746467	C	G	Missense_Mutation	55	0.236363636	No	1	Clonal	Disease_causing	Passenger	Disease_causing	likely_pathogenic	No	No	No	No	Not tested
MP15-2	Ductal	HCK	p.S509T	Private	20	30689267	G	C	Missense_Mutation	392	0.181122449	No	1	Clonal	Disease_causing	Passenger	Disease_causing	likely_pathogenic	No	No	No	No	Not tested
MP15-1	Chondroid	HIST2H2AB	p.S17W	Shared	1	149859417	G	C	Missense_Mutation	441	0.170068027	No	1	Clonal	Disease_causing	Passenger	Neutral	passenger	No	No	No	No	Not tested
MP15-2	Ductal	HIST2H2AB	p.S17W	Shared	1	149859417	G	C	Missense_Mutation	629	0.248012719	No	1	Clonal	Disease_causing	Passenger	Neutral	passenger	No	No	No	No	Not tested
MP15-1	Chondroid	HNRNPU	p.K489E	Shared	1	245021342	T	C	Missense_Mutation	163	0.116564417	No	1	Clonal	Disease_causing	Passenger	Neutral	passenger	No	No	No	No	Not tested
MP15-2	Ductal	HNRNPU	p.K489E	Shared	1	245021342	T	C	Missense_Mutation	295	0.142372881	No	1	Clonal	Disease_causing	Passenger	Neutral	passenger	No	No	No	No	Not tested
MP15-1	Chondroid	HSP90AA1	p.Q526*	Shared	14	102550258	G	A	Nonsense_Mutation	83	0.530120482	No	1	Clonal	Neutral	.	.	likely_pathogenic	Yes	Yes	No	No	Not tested
MP15-2	Ductal	HSP90AA1	p.Q526*	Shared	14	102550258	G	A	Nonsense_Mutation	102	0.656862745	No	1	Clonal	Neutral	.	.	likely_pathogenic	No	Yes	No	No	Not tested
MP15-1	Chondroid	KCNJ13	p.H297Lfs*3	Shared	2	233633081	AGATGCAAAACAGT	A	Frame_Shift_Del	324	0.194444444	No	1	Clonal	.	.	.	.	Yes	No	No	No	Not tested
MP15-2	Ductal	KCNJ13	p.H297Lfs*3	Shared	2	233633081	AGATGCAAAACAGT	A	Frame_Shift_Del	560	0.201785714	No	1	Clonal	.	.	.	.	Yes	No	No	No	Not tested
MP15-1	Chondroid	KDR	p.D1052N	Private	4	55956161	C	T	Missense_Mutation	142	0.218309859	No	1	Clonal	Disease_causing	Passenger	Disease_causing	likely_pathogenic	No	Yes	No	No	Not tested
MP15-1	Chondroid	KIAA0947	p.S422*	Shared	5	5460712	C	G	Nonsense_Mutation	185	0.264864865	No	1	Clonal	Neutral	.	.	passenger	No	No	No	No	Not tested
MP15-2	Ductal	KIAA0947	p.S422*	Shared	5	5460712	C	G	Nonsense_Mutation	322	0.335403727	No	1	Clonal	Neutral	.	.	passenger	No	No	No	No	Not tested
MP15-1	Chondroid	KIF14	p.C1312R	Shared	1	200534334	A	G	Missense_Mutation	221	0.190045249	No	1	Clonal	Disease_causing	Passenger	Neutral	passenger	No	No	No	No	Not tested
MP15-2	Ductal	KIF14	p.C1312R	Shared	1	200534334	A	G	Missense_Mutation	385	0.174025974	No	1	Clonal	Disease_causing	Passenger	Neutral	passenger	No	No	No	No	Not tested
MP15-1	Chondroid	KLHL15	p.G418A	Shared	X	24006600	C	G	Missense_Mutation	292	0.287671233	No	1	Clonal	Disease_causing	Passenger	Neutral	passenger	No	No	No	No	Yes
MP15-2	Ductal	KLHL15	p.G418A	Shared	X	24006600	C	G	Missense_Mutation	484	0.33677686	No	1	Clonal	Disease_causing	Passenger	Neutral	passenger	No	No	No	No	Yes
MP15-1	Chondroid	LCOR	p.L33R	Shared	10	98708912	T	G	Missense_Mutation	279	0.204301075	No	1	Clonal	Disease_causing	Passenger	.	passenger	Yes	No	No	No	Not tested
MP15-2	Ductal	LCOR	p.L33R	Shared	10	98708912	T	G	Missense_Mutation	321	0.065420561	No	0.385	Subclonal	Disease_causing	Passenger	.	passenger	Yes	No	No	No	Not tested
MP15-1	Chondroid	LEMD1	p.A118T	Shared	1	205350980	C	T	Missense_Mutation	200	0.235	No	1	Clonal	Neutral	Passenger	Neutral	passenger	No	No	No	No	Not tested
MP15-2	Ductal	LEMD1	p.A118T	Shared	1	205350980	C	T	Missense_Mutation	239	0.242677824	No	1	Clonal	Neutral	Passenger	Neutral	passenger	No	No	No	No	Not tested
MP15-2	Ductal	LPAR4	p.T79A	Private	X	78010601	A	G	Missense_Mutation	473	0.114164905	No	0.672	Subclonal	Disease_causing	Passenger	Neutral	passenger	No	No	No	No	Not tested
MP15-2	Ductal	LRRC55	p.R88G	Private	11	56949629	C	G	Missense_Mutation	553	0.141048825	No	0.83	Clonal	Neutral	Passenger	Disease_causing	passenger	No	No	No	No	Not tested
MP15-1	Chondroid	LRRC66	p.M591I	Shared	4	52861415	C	A	Missense_Mutation	242	0.070247934	No	0.413	Subclonal	Neutral	Passenger	Neutral	passenger	Yes	No	No	No	Not tested
MP15-2	Ductal	LRRC66	p.M591I	Shared	4	52861415	C	A	Missense_Mutation	364	0.302197802	No	1	Clonal	Neutral	Passenger	Neutral	passenger	Yes	No	No	No	Not tested
MP15-1	Chondroid	MAMLD1	p.I161Ffs*9	Shared	X	149638397	CA	C	Frame_Shift_Del	222	0.306306306	No	1	Clonal	.	.	.	.	No	No	No	No	Not tested
MP15-2	Ductal	MAMLD1	p.I161Ffs*9	Shared	X	149638397	CA	C	Frame_Shift_Del	297	0.316498316	No	1	Clonal	.	.	.	.	No	No	No	No	Not tested
MP15-2	Ductal	MAP3K9	p.M307K	Private	14	71227800	A	T	Missense_Mutation	146	0.226027397	No	1	Clonal	Disease_causing	Driver	Neutral	likely_pathogenic	Yes	No	No	No	Not tested
MP15-1	Chondroid	MAP4K2	p.R677C	Shared	11	64559444	G	A	Missense_Mutation	312	0.073717949	No	0.507	Subclonal	Disease_causing	Passenger	Neutral	passenger	No	No	No	No	Not tested
MP15-2	Ductal	MAP4K2	p.R677C	Shared	11	64559444	G	A	Missense_Mutation	253	0.260869565	No	1	Clonal	Disease_causing	Passenger	Neutral	passenger	No	No	No	No	Not tested
MP15-1	Chondroid	MEGF10	p.C615Y	Shared	5	126770382	G	A	Missense_Mutation	398	0.233668342	No	1	Clonal	Disease_causing	Passenger	Disease_causing	likely_pathogenic	No	No	No	No	Not tested
MP15-2	Ductal	MEGF10	p.C615Y	Shared	5	126770382	G	A	Missense_Mutation	448	0.341517857	No	1	Clonal	Disease_causing	Passenger	Disease_causing	likely_pathogenic	No	No	No	No	Not tested
MP15-1	Chondroid	MRO	p.V48I	Shared	18	48333220	C	T	Missense_Mutation	132	0.363636364	No	1	Clonal	Neutral	Passenger	Neutral	passenger	No	No	No	No	Not tested
MP15-2	Ductal	MRO	p.V48I	Shared	18	48333220	C	T	Missense_Mutation	214	0.341121495	No	1	Clonal	Neutral	Passenger	Neutral	passenger	No	No	No	No	Not tested
MP15-1	Chondroid	MTMR12	p.V203I	Shared	5	32263325	C	T	Missense_Mutation	172	0.209302326	No	1	Clonal	Neutral	Passenger	Disease_causing	passenger	No	No	No	No	Not tested
MP15-2	Ductal	MTMR12	p.V203I	Shared	5	32263325	C	T	Missense_Mutation	257	0.042801556	No	0.252	Subclonal	Neutral	Passenger	Disease_causing	passenger	No	No	No	No	Not tested
MP15-1	Chondroid	MUC6	p.S1842P	Shared	11	1017277	A	G	Missense_Mutation	237	0.101265823	No	0.696	Subclonal	Neutral	Passenger	Neutral	passenger	No	No	No	No	Not tested
MP15-2	Ductal	MUC6	p.S1842P	Shared	11	1017277	A	G	Missense_Mutation	275	0.112727273	No	0.663	Subclonal	Neutral	Passenger	Neutral	passenger	No	No	No	No	Not tested
MP15-2	Ductal	MVB12B	p.S210*	Private	9	129157943	C	G	Nonsense_Mutation	157	0.038216561	No	0.225	Subclonal	Disease_causing	.	.	likely_pathogenic	Yes	No	No	No	Yes
MP15-2	Ductal	NSUN4	p.L167V	Private	1	46812654	C	G	Missense_Mutation	582	0.036082474	No	0.212	Subclonal	Disease_causing	Passenger	Neutral	passenger	No	No	No	No	Yes
MP15-1	Chondroid	NTSR2	p.L311V	Shared	2	11800227	G	C	Missense_Mutation	421	0.225653207	No	1	Clonal	Neutral	Passenger	Neutral	passenger	Yes	No	No	No	Not tested
MP15-2	Ductal	NTSR2	p.L311V	Shared	2	11800227	G	C	Missense_Mutation	438	0.155251142	No	1	Clonal	Neutral	Passenger	Neutral	passenger	Yes	No	No	No	Not tested
MP15-2	Ductal	NUF2	p.L331V	Private	1	163317595	C	G	Missense_Mutation	155	0.04516129	No	0.311	Subclonal	Disease_causing	Passenger	Neutral	passenger	No	No	No	No	Yes
MP15-1	Chondroid	OR10A7	p.I289V	Shared	12	55615673	A	G	Missense_Mutation	257	0.3307393	No	1	Clonal	Disease_causing	Passenger	Neutral	passenger	No	No	No	No	Not tested
MP15-2	Ductal	OR10A7	p.I289V	Shared	12	55615673	A	G	Missense_Mutation	456	0.302631579	No	1	Clonal	Disease_causing	Passenger	Neutral	passenger	No	No	No	No	Not tested
MP15-2	Ductal	PAK6	p.D544H	Private	15	40565764	G	C	Missense_Mutation	435	0.133333333	No	0.785	Subclonal	Disease_causing	Passenger	Disease_causing	likely_pathogenic	No	No	No	No	Yes
MP15-1	Chondroid	PCM1	p.E1579D	Shared	8	17851038	A	C	Missense_Mutation	205	0.117073171	No	0.805	Clonal	Neutral	Passenger	Neutral	passenger	No	Yes	No	No	Not tested
MP15-2	Ductal	PCM1	p.E1579D	Shared	8	17851038	A	C	Missense_Mutation	329	0.045592705	No	0.268	Subclonal	Neutral	Passenger	Neutral	passenger	No	Yes	No	No	Not tested
MP15-1	Chondroid	PCSK9	p.G263S	Shared	1	55518452	G	A	Missense_Mutation	69	0.217391304	No	1	Clonal	Disease_causing	Passenger	Disease_causing	likely_pathogenic	No	No	No	No	Yes
MP15-2	Ductal	PCSK9	p.G263S	Shared	1	55518452	G	A	Missense_Mutation	76	0.078947368	No	0.465	Subclonal	Disease_causing	Passenger	Disease_causing	likely_pathogenic	No	No	No	No	Yes
MP15-1	Chondroid	PDCD11	p.E788D	Shared	10	105181191	G	C	Missense_Mutation	275	0.487272727	No	1	Clonal	Disease_causing	Passenger	Neutral	passenger	Yes	No	No	No	Yes
MP15-2	Ductal	PDCD11	p.E788D	Shared	10	105181191	G	C	Missense_Mutation	202	0.49009901	No	1	Clonal	Disease_causing	Passenger	Neutral	passenger	Yes	No	No	No	Yes
MP15-2	Ductal	PEX26	p.L171Q	Private	22	18566343	T	A	Missense_Mutation	246	0.036585366	No	0.215	Subclonal	Neutral	Passenger	Disease_causing	passenger	No	No	No	No	Yes
MP15-2	Ductal	PIK3R1	p.X582_splice	Private	5	67591246	A	C	Splice_Site	307	0.221498371	No	1	Clonal	Disease_causing	.	.	likely_pathogenic	Yes	Yes	Yes	Yes	Yes
MP15-2	Ductal	PKHD1L1	p.N2948S	Private	8	110488822	A	G	Missense_Mutation	568	0.047535211	No	0.517	Subclonal	Neutral	Passenger	Disease_causing	passenger	No	No	No	No	Not tested
MP15-1	Chondroid	PLEC	p.S3350T	Shared	8	144994352	A	T	Missense_Mutation	526	0.384030418	No	1	Clonal	Disease_causing	Passenger	Neutral	passenger	Yes	No	No	No	Yes
MP15-2	Ductal	PLEC	p.S3350T	Shared	8	144994352	A	T	Missense_Mutation	539	0.447124304	No	1	Clonal	Disease_causing	Passenger	Neutral	passenger	Yes	No	No	No	Yes
MP15-2	Ductal	PRMT10	p.E433K	Private	4	148578976	C	T	Missense_Mutation	200	0.105	No	0.618	Subclonal	Disease_causing	Passenger	Neutral	passenger	Yes	No	No	No	Not tested
MP15-1	Chondroid	PRSS57	p.S218C	Private	19	685915	G	C	Missense_Mutation	178	0.078651685	No	0.541	Subclonal	Disease_causing	Passenger	Disease_causing	likely_pathogenic	No	No	No	No	Yes
MP15-1	Chondroid	PTPRS	p.E415D	Shared	19	5244237	C	G	Missense_Mutation	611	0.181669394	No	1	Clonal	Neutral	Passenger	Neutral	passenger	No	No	No	No	Not tested
MP15-2	Ductal	PTPRS	p.E415D	Shared	19	5244237	C	G	Missense_Mutation	480	0.04375	No	0.258	Subclonal	Neutral	Passenger	Neutral	passenger	No	No	No	No	Not tested
MP15-2	Ductal	PXDN	p.D1085Y	Private	2	1652299	C	A	Missense_Mutation	613	0.168026101	No	1	Clonal	Disease_causing	Passenger	Neutral	passenger	Yes	No	No	No	Not tested

MP15-1	Chondroid	RELN	p.C2491F	Shared	7	103163856	C	A	Missense_Mutation	[illegible]	[illegible]	No	1	Clonal	Disease_causing	Driver	Neutral	likely_pathogenic	No	No	No	No	Not tested
MP15-2	Ductal	RELN	p.C2491F	Shared	7	103163856	C	A	Missense_Mutation	79	[illegible]	No	1	Clonal	Disease_causing	Driver	Neutral	likely_pathogenic	No	No	No	No	Not tested
MP15-1	Chondroid	REM1	p.L108P	Shared	20	30064571	T	C	Missense_Mutation	90	0.211111111	No	1	Clonal	Disease_causing	Passenger	Neutral	passenger	No	No	No	No	Not tested
MP15-2	Ductal	REM1	p.L108P	Shared	20	30064571	T	C	Missense_Mutation	105	0.066666667	No	0.392	Subclonal	Disease_causing	Passenger	Neutral	passenger	No	No	No	No	Not tested
MP15-2	Ductal	RESP18	p.A2P	Private	2	220197896	C	G	Missense_Mutation	260	0.080769231	No	0.556	Subclonal	Neutral	Passenger	.	passenger	Yes	No	No	No	Not tested
MP15-2	Ductal	RNPS1	p.D33N	Private	16	2314307	C	T	Missense_Mutation	412	0.097087379	No	0.571	Subclonal	Disease_causing	Passenger	Neutral	passenger	No	No	No	No	Not tested
MP15-2	Ductal	RPTOR	p.R485G	Private	17	78831644	C	G	Missense_Mutation	402	0.104477612	No	0.719	Subclonal	Disease_causing	Driver	Neutral	likely_pathogenic	No	No	No	No	Yes
MP15-2	Ductal	RSPH6A	p.A161D	Private	19	46317953	G	T	Missense_Mutation	362	0.038674033	No	0.228	Subclonal	Neutral	Passenger	Neutral	passenger	No	No	No	No	Not tested
MP15-1	Chondroid	RYR1	p.X2222_splice	Private	19	38987048	G	C	Splice_Site	329	0.136778116	No	1	Clonal	Disease_causing	.	.	likely_pathogenic	No	No	No	No	Not tested
MP15-1	Chondroid	SCOC	p.V5I	Shared	4	141294703	G	A	Missense_Mutation	242	0.090909091	No	0.534	Subclonal	Neutral	Passenger	.	passenger	Yes	No	No	No	Not tested
MP15-2	Ductal	SCOC	p.V5I	Shared	4	141294703	G	A	Missense_Mutation	399	0.260651629	No	1	Clonal	Neutral	Passenger	.	passenger	Yes	No	No	No	Not tested
MP15-2	Ductal	SCUBE3	p.E662K	Private	6	35211445	G	A	Missense_Mutation	330	0.112121212	No	0.772	Subclonal	Disease_causing	Passenger	Neutral	passenger	No	No	No	No	Not tested
MP15-1	Chondroid	SHROOM3	p.Q1919R	Shared	4	77700095	A	G	Missense_Mutation	177	0.271186441	No	1	Clonal	Neutral	Passenger	Neutral	passenger	Yes	No	No	No	Not tested
MP15-2	Ductal	SHROOM3	p.Q1919R	Shared	4	77700095	A	G	Missense_Mutation	187	0.304812834	No	1	Clonal	Neutral	Passenger	Neutral	passenger	Yes	No	No	No	Not tested
MP15-1	Chondroid	SIK3	p.M1200I	Shared	11	116718226	C	A	Missense_Mutation	141	0.177304965	No	1	Clonal	Disease_causing	Passenger	Neutral	passenger	No	No	No	No	Not tested
MP15-2	Ductal	SIK3	p.M1200I	Shared	11	116718226	C	A	Missense_Mutation	167	0.053892216	No	0.317	Subclonal	Disease_causing	Passenger	Neutral	passenger	No	No	No	No	Not tested
MP15-2	Ductal	SLC17A4	p.I268Sfs*15	Private	6	25773596	ACATTGTGTGTTCATTGGCT	A	Frame_Shift_Del	342	0.067251462	No	0.463	Subclonal	.	.	.	.	No	No	No	No	Not tested
MP15-1	Chondroid	SLC22A11	p.F511V	Shared	11	64337272	T	G	Missense_Mutation	280	0.285714286	No	1	Clonal	Neutral	Passenger	Neutral	passenger	No	No	No	No	Not tested
MP15-2	Ductal	SLC22A11	p.F511V	Shared	11	64337272	T	G	Missense_Mutation	292	0.280821918	No	1	Clonal	Neutral	Passenger	Neutral	passenger	No	No	No	No	Not tested
MP15-1	Chondroid	SLC45A2	p.C428_L430del	Shared	5	33947344	AACAGGCTGC	A	In_Frame_Del	234	0.205128205	No	1	Clonal	.	.	.	.	No	No	No	No	Not tested
MP15-2	Ductal	SLC45A2	p.C428_L430del	Shared	5	33947344	AACAGGCTGC	A	In_Frame_Del	349	0.206303725	No	1	Clonal	.	.	.	.	No	No	No	No	Not tested
MP15-1	Chondroid	SLC9C2	p.S61Yfs*5	Shared	1	173569300	GAGAA	G	Frame_Shift_Del	192	0.171875	No	1	Clonal	.	.	.	.	No	No	No	No	Not tested
MP15-2	Ductal	SLC9C2	p.S61Yfs*5	Shared	1	173569300	GAGAA	G	Frame_Shift_Del	296	0.209459459	No	1	Clonal	.	.	.	.	No	No	No	No	Not tested
MP15-2	Ductal	SMTN	p.G778A	Private	22	31494826	G	C	Missense_Mutation	150	0.1	No	0.689	Subclonal	Disease_causing	Passenger	Neutral	passenger	No	No	No	No	Not tested
MP15-2	Ductal	TANGO2	p.D38E	Private	22	20030935	C	G	Missense_Mutation	398	0.057788945	No	0.34	Subclonal	Disease_causing	Passenger	Neutral	passenger	No	No	No	No	Not tested
MP15-1	Chondroid	TAS2R8	p.V52F	Shared	12	10959426	C	A	Missense_Mutation	124	0.290322581	No	1	Clonal	Neutral	Passenger	Neutral	passenger	No	No	No	No	Not tested
MP15-2	Ductal	TAS2R8	p.V52F	Shared	12	10959426	C	A	Missense_Mutation	269	0.289962825	No	1	Clonal	Neutral	Passenger	Neutral	passenger	No	No	No	No	Not tested
MP15-1	Chondroid	TBC1D32	p.X411_splice	Private	6	121615715	C	T	Splice_Site	117	0.085470085	No	0.759	Clonal	Disease_causing	.	.	passenger	No	No	No	No	Not tested
MP15-2	Ductal	TCHH	p.E1561K	Private	1	152081012	C	T	Missense_Mutation	751	0.061251664	No	0.422	Subclonal	Neutral	Passenger	Neutral	passenger	No	No	No	No	Not tested
MP15-2	Ductal	TCHH	p.E1568*	Private	1	152080991	C	A	Nonsense_Mutation	776	0.051546392	No	0.355	Subclonal	Disease_causing	.	.	passenger	No	No	No	No	Not tested
MP15-1	Chondroid	TDRD6	p.E1542Q	Shared	6	46660489	G	C	Missense_Mutation	298	0.194630872	No	1	Clonal	Neutral	Passenger	Neutral	passenger	No	No	No	No	Not tested
MP15-2	Ductal	TDRD6	p.E1542Q	Shared	6	46660489	G	C	Missense_Mutation	510	0.392156863	No	1	Clonal	Neutral	Passenger	Neutral	passenger	No	No	No	No	Not tested
MP15-2	Ductal	TECPR1	p.Q32*	Private	7	97875365	G	A	Nonsense_Mutation	462	0.03030303	No	0.178	Subclonal	Disease_causing	.	.	passenger	No	No	No	No	Not tested
MP15-1	Chondroid	TP53	p.G112Afs*11	Shared	17	7579351	GC	G	Frame_Shift_Del	300	0.313333333	No	1	Clonal	.	.	.	.	Yes	Yes	Yes	Yes	Yes
MP15-2	Ductal	TP53	p.G112Afs*11	Shared	17	7579351	GC	G	Frame_Shift_Del	290	0.55862069	No	1	Clonal	.	.	.	.	Yes	Yes	Yes	Yes	Yes
MP15-1	Chondroid	TRIM10	p.V178M	Shared	6	30126400	C	T	Missense_Mutation	270	0.22962963	No	1	Clonal	Disease_causing	Passenger	Disease_causing	likely_pathogenic	No	No	No	No	Not tested
MP15-2	Ductal	TRIM10	p.V178M	Shared	6	30126400	C	T	Missense_Mutation	340	0.402941176	No	1	Clonal	Disease_causing	Passenger	Disease_causing	likely_pathogenic	No	No	No	No	Not tested
MP15-2	Ductal	TRIM47	p.Q383H	Private	17	73872034	C	G	Missense_Mutation	647	0.080370943	No	0.553	Subclonal	Disease_causing	Passenger	Neutral	passenger	No	No	No	No	Not tested
MP15-1	Chondroid	UNC5C	p.C594F	Private	4	96127900	C	A	Missense_Mutation	125	0.16	No	1	Clonal	Disease_causing	Passenger	Neutral	passenger	Yes	No	No	No	Not tested
MP15-1	Chondroid	VGLL3	p.P37R	Private	3	87039784	G	C	Missense_Mutation	92	0.173913043	No	1	Clonal	Disease_causing	Passenger	Neutral	passenger	Yes	No	No	No	Not tested
MP15-2	Ductal	VWA8	p.D1831H	Private	13	42144722	C	G	Missense_Mutation	322	0.049689441	No	0.243	Subclonal	Disease_causing	Passenger	Neutral	passenger	Yes	No	No	No	Yes
MP15-1	Chondroid	XPNPEP3	p.S159N	Shared	22	41278068	G	A	Missense_Mutation	477	0.132075472	No	1	Clonal	Neutral	Passenger	Neutral	passenger	No	No	No	No	Not tested
MP15-2	Ductal	XPNPEP3	p.S159N	Shared	22	41278068	G	A	Missense_Mutation	542	0.031365314	No	0.216	Subclonal	Neutral	Passenger	Neutral	passenger	No	No	No	No	Not tested
MP15-2	Ductal	ZDHHC22	p.A59V	Private	14	77605906	G	A	Missense_Mutation	235	0.042553191	No	0.25	Subclonal	Disease_causing	Passenger	Neutral	passenger	Yes	No	No	No	Not tested
MP15-2	Ductal	ZNF438	p.R617G	Private	10	31137485	G	C	Missense_Mutation	98	0.051020408	No	0.249	Subclonal	Neutral	Passenger	Neutral	passenger	Yes	No	No	No	Not tested
MP15-1	Chondroid	ZNF544	p.G582V	Shared	19	58773717	G	T	Missense_Mutation	170	0.152941176	No	1	Clonal	Disease_causing	Passenger	Neutral	passenger	No	No	No	No	Not tested
MP15-2	Ductal	ZNF544	p.G582V	Shared	19	58773717	G	T	Missense_Mutation	223	0.044843049	No	0.264	Subclonal	Disease_causing	Passenger	Neutral	passenger	No	No	No	No	Not tested
MP17-A1	Spindle	CDH8	p.S767*	Shared	16	61687612	G	T	Nonsense_Mutation	250	0.248	No	1	Clonal	Disease_causing	.	.	passenger	No	No	No	No	Yes
MP17-A2	Spindle	CDH8	p.S767*	Shared	16	61687612	G	T	Nonsense_Mutation	331	0.247734139	No	1	Clonal	Disease_causing	.	.	passenger	No	No	No	No	Yes
MP17-B1	Squamous	CDH8	p.S767*	Shared	16	61687612	G	T	Nonsense_Mutation	273	0.230769231	No	1	Clonal	Disease_causing	.	.	passenger	No	No	No	No	Yes
MP17-A2	Spindle	CDKL5	p.R840C	Private	X	18646512	C	T	Missense_Mutation	418	0.107655502	No	0.492	Subclonal	Disease_causing	Passenger	Neutral	passenger	No	No	No	No	Yes
MP17-A1	Spindle	CORO2A	p.D95Y	Private	9	100899889	C	A	Missense_Mutation	123	0.105691057	No	0.664	Subclonal	Disease_causing	Passenger	Neutral	passenger	Yes	No	No	No	Not tested
MP17-A1	Spindle	EDC4	p.G1209D	Shared	16	67916765	G	A	Missense_Mutation	179	0.340782123	No	1	Clonal	Disease_causing	Passenger	.	passenger	No	No	No	No	Not tested
MP17-A2	Spindle	EDC4	p.G1209D	Shared	16	67916765	G	A	Missense_Mutation	196	0.285714286	No	1	Clonal	Disease_causing	Passenger	.	passenger	No	No	No	No	Not tested
MP17-B1	Squamous	EDC4	p.G1209D	Shared	16	67916765	G	A	Missense_Mutation	213	0.201877934	No	1	Clonal	Disease_causing	Passenger	.	passenger	No	No	No	No	Not tested
MP17-A2	Spindle	ELMOD1	p.G111V	Private	11	107506403	G	T	Missense_Mutation	200	0.225	No	1	Clonal	Disease_causing	Passenger	.	passenger	No	No	No	No	Not tested
MP17-A1	Spindle	EPHB6	p.Q771K	Private	7	142566754	C	A	Missense_Mutation	52	0.134615385	No	0.981	Clonal	Disease_causing	Driver	Disease_causing	likely_pathogenic	Yes	No	Yes	No	Not tested
MP17-A1	Spindle	ESYT2	p.R256*	Shared	7	158581071	G	A	Nonsense_Mutation	86	0.476744186	No	1	Clonal	Disease_causing	.	.	likely_pathogenic	Yes	No	No	No	Not tested
MP17-A2	Spindle	ESYT2	p.R256*	Shared	7	158581071	G	A	Nonsense_Mutation	106	0.396226415	No	1	Clonal	Disease_causing	.	.	likely_pathogenic	No	No	No	No	Not tested
MP17-B1	Squamous	ESYT2	p.R256*	Shared	7	158581071	G	A	Nonsense_Mutation	71	0.422535211	No	1	Clonal	Disease_causing	.	.	likely_pathogenic	Yes	No	No	No	Not tested
MP17-A1	Spindle	FAT4	p.D4021N	Shared	4	126389828	G	A	Missense_Mutation	292	0.260273973	No	1	Clonal	Disease_causing	Passenger	Neutral	passenger	No	Yes	No	No	Yes
MP17-A2	Spindle	FAT4	p.D4021N	Shared	4	126389828	G	A	Missense_Mutation	345	0.327536232	No	1	Clonal	Disease_causing	Passenger	Neutral	passenger	No	Yes	No	No	Yes
MP17-B1	Squamous	FAT4	p.D4021N	Shared	4	126389828	G	A	Missense_Mutation	268	0.179104478	No	1	Clonal	Disease_causing	Passenger	Neutral	passenger	No	No	No	No	Yes
MP17-A1	Spindle	FMN1	p.L615Ffs*5	Private	15	33261377	GAGAGCTGGCTT	G	Frame_Shift_Del	309	0.071197411	No	0.59	Subclonal	.	.	.	.	No	No	No	No	Not tested
MP17-A1	Spindle	GNAS	p.R201C	Shared	20	57484420	C	T	Missense_Mutation	330	0.260606061	Yes	1	Clonal	Disease_causing	Passenger	Disease_causing	likely_pathogenic	No	Yes	No	No	Yes
MP17-A2	Spindle	GNAS	p.R201C	Shared	20	57484420	C	T	Missense_Mutation	335	0.298507463	Yes	1	Clonal	Disease_causing	Passenger	Disease_causing	likely_pathogenic	No	Yes	No	No	Yes
MP17-B1	Squamous	GNAS	p.R201C	Shared	20	57484420	C	T	Missense_Mutation	334	0.254491018	Yes	1	Clonal	Disease_causing	Passenger	Disease_causing	likely_pathogenic	No	No	No	Yes	Yes
MP17-A1	Spindle	HMCN1	p.T5501I	Shared	1	186157102	C	T	Missense_Mutation	432	0.293981481	No	1	Clonal	Disease_causing	Passenger	Neutral	passenger	No	No	No	No	Yes
MP17-A2	Spindle	HMCN1	p.T5501I	Shared	1	186157102	C	T	Missense_Mutation	476	0.344537815	No	1	Clonal	Disease_causing	Passenger	Neutral	passenger	No	No	No	No	Yes
MP17-B1	Squamous	HMCN1	p.T5501I	Shared	1	186157102	C	T	Missense_Mutation	343	0.253644315	No	1	Clonal	Disease_causing	Passenger	Neutral	passenger	No	No	No	No	Yes
MP17-A1	Spindle	KMT2D	p.Q809Rfs*121	Shared	12	49445040	TG	T	Frame_Shift_Del	362	0.309392265	No	1	Clonal	.	.	.	.	Yes	Yes	Yes	Yes	Not tested
MP17-A2	Spindle	KMT2D	p.Q809Rfs*121	Shared	12	49445040	TG	T	Frame_Shift_Del	426	0.323943662	No	1	Clonal	.	.	.	.	No	Yes	Yes	Yes	Not tested
MP17-B1	Squamous	KMT2D	p.Q809Rfs*121	Shared	12	49445040	TG	T	Frame_Shift_Del	406	0.295566502	No	1	Clonal	.	.	.	.	Yes	Yes	Yes	No	Not tested
MP17-A1	Spindle	MIPEP	p.Q439Sfs*50	Shared	13	24413811	G	GA	Frame_Shift_Ins	140	0.207142857	No	1	Clonal	.	.	.	.	No	No	No	No	Not tested
MP17-A2	Spindle	MIPEP	p.Q439Sfs*50	Shared	13	24413811	G	GA	Frame_Shift_Ins	180	0.35	No	1	Clonal	.	.	.	.	No	No	No	No	Not tested
MP17-B1	Squamous	MIPEP	p.Q439Sfs*50	Shared	13	24413811	G	GA	Frame_Shift_Ins	144	0.138888889	No	1	Clonal	.	.	.	.	No	No	No	No	Not tested
MP17-A2	Spindle	MRPS15	p.P192R	Private	1	36921849	G	C	Missense_Mutation	261	0.275862069	No	1	Clonal	Disease_causing	Passenger	.	passenger	No	No	No	No	Not tested
MP17-A1	Spindle	NCAM1	p.R405Q	Shared	11	113085211	G	A	Missense_Mutation	74	0.135135135	No	1	Clonal	Disease_causing	Passenger	Neutral	passenger	No	No	No	No	Not tested
MP17-B1	Squamous	NCAM1	p.R405Q	Shared	11	113085211	G	A	Missense_Mutation	83	0.156626506	No	1	Clonal	Disease_causing	Passenger	Neutral	passenger	No	No	No	No	Not tested
MP17-A1	Spindle	NCAM2	p.P622S	Shared	21	22841072	C	T	Missense_Mutation	118	0.211864407	No	1	Clonal	Disease_causing	Driver	Neutral	likely_pathogenic	No	No	No	No	Yes
MP17-A2	Spindle	NCAM2	p.P622S	Shared	21	22841072	C	T	Missense_Mutation	136	0.316176471	No	1	Clonal	Disease_causing	Driver	Neutral	likely_pathogenic	No	No	No	No	Yes
MP17-B1	Squamous	NCAM2	p.P622S	Shared	21	22841072	C	T	Missense_Mutation	107	0.130841121	No	1	Clonal	Disease_causing	Driver	Neutral	likely_pathogenic	No	No	No	No	Yes
MP17-A1	Spindle	NLRP8	p.V571M	Shared	19	56467135	G	A	Missense_Mutation	182	0.285714286	No	1	Clonal	Neutral	Passenger	Disease_causing	passenger	No	No	No	No	Not tested
MP17-A2	Spindle	NLRP8	p.V571M	Shared	19	56467135	G	A	Missense_Mutation	258	0.282945736	No	1	Clonal	Neutral	Passenger	Disease_causing	passenger	No	No	No	No	Not tested
MP17-B1	Squamous	NLRP8	p.V571M	Shared	19	56467135	G	A	Missense_Mutation	228	0.311403509	No	1	Clonal	Neutral	Passenger	Disease_causing	passenger	No	No	No	No	Not tested
MP17-A1	Spindle	NOTCH3	p.Y2244Tfs*2	Shared	19	15271708	TA	T	Frame_Shift_Del	125	0.32	No	1	Clonal	.	.	.	.	No	No	No	No	Not tested
MP17-A2	Spindle	NOTCH3	p.Y2244Tfs*2	Shared	19	15271708	TA	T	Frame_Shift_Del	188	0.340425532	No	1	Clonal	.	.	.	.	No	No	No	No	Not tested
MP17-B1	Squamous	NOTCH3	p.Y2244Tfs*2	Shared	19	15271708	TA	T	Frame_Shift_Del	148	0.256756757	No	1	Clonal	.	.	.	.	No	No	No	No	Not tested
MP17-A2	Spindle	PCMTD1	p.I259T	Private	8	52733209	A	G	Missense_Mutation	190	0.063157895	No	0.289	Subclonal	Disease_causing	Passenger	Neutral	passenger	No	No	No	No	Not tested
MP17-A2	Spindle	PCMTD1	p.N257K	Private	8	52733214	A	C	Missense_Mutation	171	0.070175439	No	0.321	Subclonal	Disease_causing	Passenger	Neutral	passenger	No	No	No	No	Not tested
MP17-A1	Spindle	PHLDB1	p.T616N	Shared	11	118501943	C	A	Missense_Mutation	177	0.389830508	No	1	Clonal	Disease_causing	Passenger	Neutral	passenger	No	No	No	No	Yes
MP17-A2	Spindle	PHLDB1	p.T616N	Shared	11	118501943	C	A	Missense_Mutation	228	0.377192982	No	1	Clonal	Disease_causing	Passenger	Neutral	passenger	No	No	No	No	Yes
MP17-B1	Squamous	PHLDB1	p.T616N	Shared	11	118501943	C	A	Missense_Mutation	253	0.197628458	No	1	Clonal	Disease_causing	Passenger	Neutral	passenger	No	No	No	No	Yes
MP17-A1	Spindle	PIEZO2	p.E2283D	Shared	18	10696074	C	G	Missense_Mutation	122	0.221311475	No	1	Clonal	Disease_causing	Passenger	Disease_causing	likely_pathogenic	No	No	No	No	Yes
MP17-B1	Squamous	PIEZO2	p.E2283D	Shared	18	10696074	C	G	Missense_Mutation	160	0.24375	No	1	Clonal	Disease_causing	Passenger	Disease_causing	likely_pathogenic	No	No	No	No	Yes
MP17-A1	Spindle	PIK3CA	p.H1047R	Shared	3	178952085	A	G	Missense_Mutation	287	0.25087108	Yes	1	Clonal	Disease_causing	Driver	Neutral	likely_pathogenic	Yes	Yes	Yes	Yes	Yes
MP17-A2	Spindle	PIK3CA	p.H1047R	Shared	3	178952085	A	G	Missense_Mutation	346	0.309248555	Yes	1	Clonal	Disease_causing	Driver	Neutral	likely_pathogenic	Yes	Yes	Yes	Yes	Yes
MP17-B1	Squamous	PIK3CA	p.H1047R	Shared	3	178952085	A	G	Missense_Mutation	294	0.316326531	Yes	1	Clonal	Disease_causing	Driver	Neutral	likely_pathogenic	Yes	Yes	Yes	No	Yes
MP17-A2	Spindle	PRKCE	p.V73Rfs*24	Private	2	45879452	CGATGTGTGCAACGGACGCAA	C	Frame_Shift_Del	236	0.173728814	No	0.967	Clonal	.	.	.	.	Yes	No	No	No	Not tested
MP17-A1	Spindle	PRKD3	p.E703*	Private	2	37486764	C	A	Nonsense_Mutation	241	0.066390041	No	0.55	Subclonal	Neutral	.	.	passenger	Yes	No	No	No	Not tested
MP17-A1	Spindle	RSPH9	p.T219K	Shared	6	43634191	C	A	Missense_Mutation	154	0.227272727	No	1	Clonal	Neutral	Passenger	.	passenger	No	No	No	No	Not tested
MP17-A2	Spindle	RSPH9	p.T219K	Shared	6	43634191	C	A	Missense_Mutation	240	0.4125	No	1	Clonal	Neutral	Passenger	.	passenger	No	No	No	No	Not tested
MP17-B1	Squamous	RSPH9	p.T219K	Shared	6	43634191	C	A	Missense_Mutation	183	0.207650273	No	1	Clonal	Neutral	Passenger	.	passenger	No	No	No	No	Not tested
MP17-A1	Spindle	SLC1A4	p.V282L	Shared	2	65243617	G	T	Missense_Mutation	165	0.109090909	No	0.904	Clonal	Disease_causing	Passenger	Neutral	passenger	Yes	No	No	No	Not tested
MP17-A2	Spindle	SLC1A4	p.V282L	Shared	2	65243617	G	T	Missense_Mutation	226	0.274336283	No	1	Clonal	Disease_causing	Passenger	Neutral	passenger	Yes	No	No	No	Not tested
MP17-B1	Squamous	SLC1A4	p.V282L	Shared	2	65243617	G	T	Missense_Mutation	144	0.111111111	No	0.972	Clonal	Disease_causing	Passenger	Neutral	passenger	Yes	No	No	No	Not tested
MP17-A1	Spindle	SOX30	p.V462A	Shared	5	157073647	A	G	Missense_Mutation	78	0.282051282	No	1	Clonal	Disease_causing	Passenger	Disease_causing	likely_pathogenic	No	No	No	No	Yes
MP17-A2	Spindle	SOX30	p.V462A	Shared	5	157073647	A	G	Missense_Mutation	89	0.303370787	No	1	Clonal	Disease_causing	Passenger	Disease_causing	likely_pathogenic	No	No	No	No	Yes
MP17-B1	Squamous	SOX30	p.V462A	Shared	5	157073647	A	G	Missense_Mutation	82	0.182926829	No	1	Clonal	Disease_causing	Passenger	Disease_causing	likely_pathogenic	No	No	No	No	Yes
MP17-A1	Spindle	TP53	p.V173L	Shared	17	7578413	C	A	Missense_Mutation	243	0.263374486	Yes	1	Clonal	Disease_causing	Passenger	Disease_causing	likely_pathogenic	No	Yes	Yes	Yes	Not tested
MP17-A1	Spindle	TP53	p.X225_splice	Shared	17	7577609	C	T	Splice_Site	125	0.24	No	1	Clonal	Disease_causing	.	.	likely_pathogenic	No	Yes	Yes	Yes	Not tested
MP17-A2	Spindle	TP53	p.V173L	Shared	17	7578413	C	A	Missense_Mutation	294	0.285714286	Yes	1	Clonal	Disease_causing	Passenger	Disease_causing	likely_pathogenic	No	Yes	Yes	Yes	Not tested
MP17-B1	Squamous	TP53	p.V173L	Shared	17	7578413	C	A	Missense_Mutation	327	0.23853211	Yes	1	Clonal	Disease_causing	Passenger	Disease_causing	likely_pathogenic	No	Yes	Yes	Yes	Not tested
MP17-B1	Squamous	TP53	p.X225_splice	Shared	17	7577609	C	T	Splice_Site	129	0.170542636	No	1	Clonal	Disease_causing	.	.	likely_pathogenic	No	Yes	Yes	Yes	Not tested
MP18-1	Chondroid	ABCA13	p.T4150I	Shared	7	48452170	C	T	Missense_Mutation	96	0.270833333	No	1	Clonal	Disease_causing	Passenger	Disease_causing	likely_pathogenic	Yes	No	No	No	Yes
MP18-2	Ductal	ABCA13	p.T4150I	Shared	7	48452170	C	T	Missense_Mutation	83	0.397590361	No	1	Clonal	Disease_causing	Passenger	Disease_causing	likely_pathogenic	Yes	No	No	No	Yes
MP18-1	Chondroid	ACSS2	p.I93T	Private	20	33470696	T	C	Missense_Mutation	209	0.062200957	No	0.428	Subclonal	Disease_causing	Passenger	Neutral	passenger	Yes	No	No	No	Not tested
MP18-2	Ductal	AGBL2	p.P867T	Private	11	47681835	G	T	Missense_Mutation	206	0.072815534	No	0.391	Subclonal	Neutral	Passenger	Neutral	passenger	Yes	No	No	No	Yes
MP18-2	Ductal	AKIP1	p.V182I	Private	11	8940938	G	A	Missense_Mutation	152	0.401315789	No	1	Clonal	Neutral	Passenger	Neutral	passenger	Yes	No	No	No	Yes
MP18-1	Chondroid	ALPK3	p.D1439V	Private	15	85401679	A	T	Missense_Mutation	68	0.117647059	No	0.691	Subclonal	Disease_causing	Passenger	Neutral	passenger	No	No	No	No	Yes
MP18-1	Chondroid	ALS2	p.T387R	Shared	2	202622436	G	C	Missense_Mutation	115	0.130434783	No	1	Clonal	Disease_causing	Passenger	Neutral	passenger	No	No	No	No	Not tested
MP18-2	Ductal	ALS2	p.T387R	Shared	2	202622436	G	C	Missense_Mutation	89	0.359550562	No	1	Clonal	Disease_causing	Passenger	Neutral	passenger	No	No	No	No	Not tested
MP18-2	Ductal	ANKHD1	p.P1870A	Private	5	139908139	C	G	Missense_Mutation	278	0.413669065	No	1	Clonal	Disease_causing	Passenger	Neutral	passenger	No	No	No	No	Yes
MP18-1	Chondroid	ANKRD55	p.E601Q	Private	5	55396054	C	G	Missense_Mutation	124	0.185483871	No	1	Clonal	Neutral	Passenger	Neutral	passenger	Yes	No	No	No	Yes
MP18-1	Chondroid	BCL9L	p.F1499L	Shared	11	118769127	G	T	Missense_Mutation	168	0.303571429	No	1	Clonal	Disease_causing	Passenger	Neutral	passenger	Yes	Yes	No	No	Yes
MP18-2	Ductal	BCL9L	p.F1499L	Shared	11	118769127	G	T	Missense_Mutation	176	0.261363636	No	1	Clonal	Disease_causing	Passenger	Neutral	passenger	Yes	Yes	No	No	Yes
MP18-2	Ductal	C17orf80	p.A319P	Private	17	71232576	G	C	Missense_Mutation	275	0.214545455	No	1	Clonal	Neutral	Passenger	Neutral	passenger	No	No	No	No	Yes
MP18-2	Ductal	C9orf152	p.S238*	Private	9	112963235	G	C	Nonsense_Mutation	146	0.095890411	No	0.515	Subclonal	Neutral	.	.	likely_pathogenic	No	No	No	No	Yes
MP18-1	Chondroid	CASKIN2	p.E423G	Private	17	73500699	T	C	Missense_Mutation	441	0.038548753	No	0.342	Subclonal	Disease_causing	Passenger	Neutral	passenger	No	No	No	No	Yes
MP18-1	Chondroid	CCAR2	p.G51V	Private	8	22464121	G	T	Missense_Mutation	154	0.175324675	No	1	Clonal	Disease_causing	Passenger	Neutral	passenger	No	No	No	No	Yes
MP18-2	Ductal	CCT3	p.M59I	Private	1	156304534	C	T	Missense_Mutation	252	0.186507937	No	1	Clonal	Disease_causing	Passenger	Neutral	passenger	Yes	No	No	No	Not tested
MP18-1	Chondroid	CD3E	p.S55P	Shared	11	118183392	T	C	Missense_Mutation	327	0.259938838	No	1	Clonal	Neutral	Passenger	Neutral	passenger	Yes	No	No	No	Not tested
MP18-2	Ductal	CD3E	p.S55P	Shared	11	118183392	T	C	Missense_Mutation	285	0.38245614	No	1	Clonal	Neutral	Passenger	Neutral	passenger	Yes	No	No	No	Not tested
MP18-2	Ductal	CFHR5	p.G346R	Private	1	196967323	G	A	Missense_Mutation	50	0.14	No	0.892	Clonal	Neutral	Passenger	Neutral	passenger	Yes	No	No	No	Not tested
MP18-1	Chondroid	CLASRP	p.L12I	Private	19	45543504	C	A	Missense_Mutation	180	0.088888889	No	0.7	Subclonal	Disease_causing	Passenger	Neutral	passenger	Yes	No	No	No	Yes
MP18-2	Ductal	CRNN	p.T313I	Private	1	152382620	G	A	Missense_Mutation	462	0.212121212	No	1	Clonal	Neutral	Passenger	Neutral	passenger	Yes	No	No	No	Yes
MP18-1	Chondroid	CYFIP1	p.A823P	Shared	15	22969241	G	C	Missense_Mutation	279	0.129032258	No	0.758	Subclonal	Disease_causing	Passenger	Neutral	passenger	No	No	No	No	Not tested
MP18-2	Ductal	CYFIP1	p.A823P	Shared	15	22969241	G	C	Missense_Mutation	345	0.313043478	No	1	Clonal	Disease_causing	Passenger	Neutral	passenger	Yes	No	No	No	Not tested
MP18-2	Ductal	DCST1	p.A651V	Private	1	155023175	C	T	Missense_Mutation	153	0.267973856	No	1	Clonal	Disease_causing	Passenger	Neutral	passenger	Yes	No	No	No	Not tested
MP18-2	Ductal	DGAT1	p.V192A	Private	8	145542025	A	G	Missense_Mutation	269	0.126394052	No	1	Clonal	Disease_causing	Passenger	Neutral	passenger	Yes	No	No	No	Yes
MP18-1	Chondroid	DLC1	p.K1179E	Shared	8	12950326	T	C	Missense_Mutation	52	0.461538462	No	1	Clonal	Disease_causing	Passenger	Neutral	passenger	No	No	No	No	Not tested
MP18-2	Ductal	DLC1	p.K1179E	Shared	8	12950326	T	C	Missense_Mutation	57	0.526315789	No	1	Clonal	Disease_causing	Passenger	Neutral	passenger	No	No	No	No	Not tested
MP18-1	Chondroid	DLGAP5	p.N236K	Private	14	55646413	G	C	Missense_Mutation	95	0.105263158	No	0.619	Subclonal	Neutral	Passenger	Neutral	passenger	No	No	No	No	Yes
MP18-2	Ductal	DNAH10	p.R2487C	Private	12	124358132	C	T	Missense_Mutation	253	0.260869565	No	1	Clonal	Disease_causing	Passenger	Neutral	passenger	No	No	No	No	Not tested
MP18-1	Chondroid	DOPEY1	p.G2274V	Shared	6	83869538	G	T	Missense_Mutation	175	0.188571429	No	1	Clonal	Disease_causing	Passenger	Neutral	passenger	Yes	No	No	No	Yes
MP18-2	Ductal	DOPEY1	p.G2274V	Shared	6	83869538	G	T	Missense_Mutation	137	0.270072993	No	1	Clonal	Disease_causing	Passenger	Neutral	passenger	Yes	No	No	No	Yes
MP18-2	Ductal	DSCAML1	p.G2082C	Private	11	117299142	C	A	Missense_Mutation	355	0.261971831	No	1	Clonal	Disease_causing	Driver	Neutral	likely_pathogenic	Yes	No	No	No	Not tested
MP18-1	Chondroid	EPB42	p.S671L	Private	15	43489654	G	A	Missense_Mutation	190	0.2	No	1	Clonal	Neutral	Passenger	Neutral	passenger	No	No	No	No	Yes
MP18-1	Chondroid	GRIK3	p.I67F	Private	1	37356614	T	A	Missense_Mutation	409	0.058679707	No	0.404	Subclonal	Disease_causing	Passenger	Disease_causing	likely_pathogenic	Yes	No	No	No	Yes
MP18-1	Chondroid	GSPT2	p.D90N	Shared	X	51486990	G	A	Missense_Mutation	206	0.087378641	No	0.514	Subclonal	Neutral	Passenger	Neutral	passenger	No	No	No	No	Not tested
MP18-2	Ductal	GSPT2	p.D90N	Shared	X	51486990	G	A	Missense_Mutation	191	0.031413613	No	0.137	Subclonal	Neutral	Passenger	Neutral	passenger	No	No	No	No	Not tested
MP18-1	Chondroid	HP1BP3	p.M153L	Shared	1	21099997	T	A	Missense_Mutation	169	0.201183432	No	1	Clonal	Disease_causing	Passenger	Neutral	passenger	Yes	No	No	No	Yes
MP18-2	Ductal	HP1BP3	p.M153L	Shared	1	21099997	T	A	Missense_Mutation	174	0.281609195	No	1	Clonal	Disease_causing	Passenger	Neutral	passenger	Yes	No	No	No	Yes
MP18-2	Ductal	IFT74	p.Q440H	Private	9	27048259	G	C	Missense_Mutation	179	0.229050279	No	1	Clonal	Neutral	Passenger	Neutral	passenger	Yes	No	No	No	Yes
MP18-1	Chondroid	JPH3	p.G715_S721del	Private	16	87724107	ATGGGGATGAGCTCAAGTCCAG	A	In_Frame_Del	280	0.071428571	No	0.491	Subclonal	.	.	.	.	No	No	No	No	Not tested
MP18-1	Chondroid	KDM6A	p.I970Nfs*9	Shared	X	44937717	G	GT	Frame_Shift_Ins	358	0.346368715	No	1	Clonal	.	.	.	.	No	Yes	Yes	Yes	Yes
MP18-2	Ductal	KDM6A	p.I970Nfs*9	Shared	X	44937717	G	GT	Frame_Shift_Ins	241	0.452282158	No	1	Clonal	.	.	.	.	No	Yes	Yes	Yes	Yes
MP18-1	Chondroid	KIAA1244	p.V1889I	Private	6	138655648	G	A	Missense_Mutation	303	0.141914191	No	0.976	Clonal	Disease_causing	Passenger	Neutral	passenger	Yes	No	No	No	Yes
MP18-1	Chondroid	KIAA1244	p.G1449S	Private	6	138635076	G	A	Missense_Mutation	212	0.037735849	No	0.26	Subclonal	Disease_causing	Passenger	Neutral	passenger	Yes	No	No	No	Yes
MP18-1	Chondroid	KLF16	p.H201Q	Shared	19	1854614	G	T	Missense_Mutation	139	0.302158273	No	1	Clonal	Disease_causing	Passenger	Neutral	passenger	No	No	No	No	Yes
MP18-2	Ductal	KLF16	p.H201Q	Shared	19	1854614	G	T	Missense_Mutation	188	0.595744681	No	1	Clonal	Disease_causing	Passenger	Neutral	passenger	No	No	No	No	Yes
MP18-1	Chondroid	KLHL17	p.A258V	Private	1	897796	C	T	Missense_Mutation	477	0.062893082	No	0.433	Subclonal	Disease_causing	Passenger	Neutral	passenger	Yes	No	No	No	Not tested
MP18-2	Ductal	LAMA5	p.N327H	Private	20	60922062	T	G	Missense_Mutation	100	0.16	No	0.859	Clonal	Disease_causing	Passenger	Neutral	passenger	Yes	No	No	No	Yes
MP18-1	Chondroid	LRRC4C	p.N158K	Private	11	40137369	G	C	Missense_Mutation	219	0.050228311	No	0.345	Subclonal	Disease_causing	Passenger	Neutral	passenger	Yes	No	No	No	Yes
MP18-1	Chondroid	MAP1A	p.A728D	Shared	15	43815854	C	A	Missense_Mutation	137	0.167883212	No	0.987	Clonal	Disease_causing	Passenger	Neutral	passenger	No	No	No	No	Not tested
MP18-2	Ductal	MAP1A	p.A728D	Shared	15	43815854	C	A	Missense_Mutation	124	0.419354839	No	1	Clonal	Disease_causing	Passenger	Neutral	passenger	No	No	No	No	Not tested
MP18-1	Chondroid	MDN1	p.Q4932E	Shared	6	90368556	G	C	Missense_Mutation	236	0.296610169	No	1	Clonal	Neutral	Passenger	Neutral	passenger	Yes	No	No	No	Not tested
MP18-2	Ductal	MDN1	p.Q4932E	Shared	6	90368556	G	C	Missense_Mutation	225	0.284444444	No	1	Clonal	Neutral	Passenger	Neutral	passenger	Yes	No	No	No	Not tested
MP18-1	Chondroid	MMP16	p.P314L	Private	8	89128878	G	A	Missense_Mutation	117	0.102564103	No	0.91	Clonal	Disease_causing	Passenger	Neutral	passenger	Yes	No	No	No	Yes
MP18-2	Ductal	MRPS7	p.L110H	Private	17	73258938	T	A	Missense_Mutation	266	0.289473684	No	1	Clonal	Disease_causing	Passenger	Neutral	passenger	No	No	No	No	Yes
MP18-1	Chondroid	MSL2	p.P57R	Private	3	135871553	G	C	Missense_Mutation	240	0.104166667	No	0.821	Clonal	Disease_causing	Passenger	.	passenger	No	No	No	No	Yes
MP18-1	Chondroid	NIPAL1	p.C321F	Private	4	48037918	G	T	Missense_Mutation	202	0.064356436	No	0.443	Subclonal	Disease_causing	Passenger	Disease_causing	likely_pathogenic	Yes	No	No	No	Not tested
MP18-2	Ductal	NTSR2	p.L356V	Private	2	11798772	G	C	Missense_Mutation	97	0.381443299	No	1	Clonal	Neutral	Passenger	Neutral	passenger	No	No	No	No	Yes
MP18-1	Chondroid	OBSCN	p.R7879C	Shared	1	228559243	C	T	Missense_Mutation	250	0.164	No	1	Clonal	Neutral	Passenger	Neutral	passenger	Yes	No	No	No	Not tested
MP18-2	Ductal	OBSCN	p.R7879C	Shared	1	228559243	C	T	Missense_Mutation	131	0.374045802	No	1	Clonal	Neutral	Passenger	Neutral	passenger	Yes	No	No	No	Not tested
MP18-2	Ductal	OR13G1	p.L247F	Private	1	247835605	G	A	Missense_Mutation	104	0.211538462	No	1	Clonal	Neutral	Passenger	Neutral	passenger	Yes	No	No	No	Not tested
MP18-1	Chondroid	PARP1	p.V818F	Private	1	226553708	C	A	Missense_Mutation	259	0.057915058	No	0.572	Subclonal	Disease_causing	Passenger	Neutral	passenger	Yes	No	No	No	Yes
MP18-1	Chondroid	PDE9A	p.I83M	Private	21	44119108	C	G	Missense_Mutation	184	0.070652174	No	0.486	Subclonal	Disease_causing	Passenger	Neutral	passenger	Yes	No	No	No	Not tested
MP18-1	Chondroid	PIK3R1	p.N410Ifs*6	Private	5	67589239	GAATGA	G	Frame_Shift_Del	151	0.052980132	No	0.311	Subclonal	.	.	.	.	No	Yes	Yes	Yes	Not tested
MP18-2	Ductal	PITPNB	p.M74I	Private	22	28293856	C	G	Missense_Mutation	214	0.275700935	No	1	Clonal	Disease_causing	Passenger	Neutral	passenger	Yes	No	No	No	Yes
MP18-1	Chondroid	PRR14L	p.S458C	Private	22	32112452	G	C	Missense_Mutation	380	0.142105263	No	1	Clonal	Neutral	Passenger	Neutral	passenger	Yes	No	No	No	Not tested
MP18-1	Chondroid	PTCH1	p.D293A	Private	9	98242739	T	G	Missense_Mutation	97	0.082474227	No	0.485	Subclonal	Disease_causing	Passenger	Neutral	passenger	No	Yes	No	No	Yes
MP18-1	Chondroid	PTCHD2	p.A493Hfs*85	Private	1	11575448	TGCCAG	T	Frame_Shift_Del	158	0.164556962	No	1	Clonal	.	.	.	.	Yes	No	No	No	Yes
MP18-1	Chondroid	PVRIG	p.V181F	Private	7	99818241	G	T	Missense_Mutation	395	0.197468354	No	1	Clonal	Neutral	Passenger	Neutral	passenger	Yes	No	No	No	Yes
MP18-1	Chondroid	RBM44	p.P215Q	Private	2	238726203	C	A	Missense_Mutation	113	0.238938053	No	1	Clonal	Neutral	Passenger	Neutral	passenger	No	No	No	No	Yes
MP18-1	Chondroid	RGL3	p.V295M	Private	19	11517208	C	T	Missense_Mutation	195	0.102564103	No	0.808	Clonal	Disease_causing	Passenger	Neutral	passenger	Yes	No	No	No	Not tested
MP18-1	Chondroid	RIMBP2	p.G827R	Private	12	130898843	C	T	Missense_Mutation	139	0.158273381	No	1	Clonal	Disease_causing	Passenger	Neutral	passenger	Yes	No	No	No	Not tested
MP18-1	Chondroid	RITA1	p.Q13H	Private	12	113624590	G	C	Missense_Mutation	241	0.037344398	No	0.257	Subclonal	Neutral	Passenger	Neutral	passenger	Yes	No	No	No	Not tested
MP18-2	Ductal	ROBO4	p.R285K	Private	11	124765535	C	T	Missense_Mutation	267	0.277153558	No	1	Clonal	Disease_causing	Passenger	Neutral	passenger	Yes	No	No	No	Yes
MP18-2	Ductal	SDCBP2	p.H230Q	Private	20	1293023	G	C	Missense_Mutation	455	0.065934066	No	0.288	Subclonal	Disease_causing	Passenger	Neutral	passenger	No	No	No	No	Not tested
MP18-1	Chondroid	SERPINI2	p.D142Y	Private	3	167184897	C	A	Missense_Mutation	174	0.103448276	No	0.815	Clonal	Disease_causing	Passenger	Neutral	passenger	No	No	No	No	Yes
MP18-1	Chondroid	SHMT2	p.H504Y	Shared	12	57628139	C	T	Missense_Mutation	206	0.36407767	No	1	Clonal	Disease_causing	Passenger	Neutral	passenger	No	No	No	No	Yes
MP18-2	Ductal	SHMT2	p.H504Y	Shared	12	57628139	C	T	Missense_Mutation	190	0.410526316	No	1	Clonal	Disease_causing	Passenger	Neutral	passenger	No	No	No	No	Yes
MP18-1	Chondroid	SLC4A1	p.G609A	Private	17	42332639	C	G	Missense_Mutation	129	0.178294574	No	1	Clonal	Disease_causing	Passenger	Neutral	passenger	No	No	No	No	Not tested
MP18-1	Chondroid	SOBP	p.P133A	Private	6	107827607	C	G	Missense_Mutation	120	0.041666667	No	0.287	Subclonal	Disease_causing	Passenger	Neutral	passenger	Yes	No	No	No	Yes
MP18-1	Chondroid	SOCS3	p.C193*	Private	17	76354598	A	T	Nonsense_Mutation	282	0.088652482	No	0.787	Subclonal	Disease_causing	.	.	likely_pathogenic	No	No	No	No	Not tested
MP18-1	Chondroid	SP110	p.K432E	Private	2	231048342	T	C	Missense_Mutation	249	0.108433735	No	0.854	Clonal	Neutral	Passenger	Neutral	passenger	No	No	No	No	Not tested
MP18-2	Ductal	SPSB3	p.V222M	Private	16	1827805	C	T	Missense_Mutation	208	0.302884615	No	1	Clonal	Disease_causing	Passenger	Neutral	passenger	Yes	No	No	No	Yes
MP18-1	Chondroid	SUN1	p.V338A	Shared	7	892549	T	C	Missense_Mutation	63	0.079365079	No	0.466	Subclonal	Neutral	Passenger	Neutral	passenger	No	No	No	No	Not tested
MP18-2	Ductal	SUN1	p.V338A	Shared	7	892549	T	C	Missense_Mutation	51	0.31372549	No	1	Clonal	Neutral	Passenger	Neutral	passenger	Yes	No	No	No	Not tested
MP18-1	Chondroid	SYNE1	p.T4044S	Private	6	152665310	G	C	Missense_Mutation	126	0.047619048	No	0.327	Subclonal	Disease_causing	Passenger	Neutral	passenger	Yes	No	No	No	Yes
MP18-2	Ductal	SYT17	p.L61M	Private	16	19184899	C	A	Missense_Mutation	191	0.261780105	No	1	Clonal	Disease_causing	Passenger	Neutral	passenger	Yes	No	No	No	Not tested
MP18-1	Chondroid	TJP1	p.R1030Tfs*6	Shared	15	30011250	GTCTGGCC	G	Frame_Shift_Del	291	0.113402062	No	0.666	Subclonal	.	.	.	.	No	No	No	No	Not tested
MP18-2	Ductal	TJP1	p.R1030Tfs*6	Shared	15	30011250	GTCTGGCC	G	Frame_Shift_Del	185	0.464864865	No	1	Clonal	.	.	.	.	No	No	No	No	Not tested
MP18-2	Ductal	TMCC2	p.K162Q	Private	1	205210909	A	C	Missense_Mutation	307	0.179153094	No	1	Clonal	Disease_causing	Passenger	Neutral	passenger	Yes	No	No	No	Yes
MP18-1	Chondroid	TOP3B	p.R414I	Private	22	22317229	C	A	Missense_Mutation	197	0.096446701	No	0.663	Subclonal	Disease_causing	Passenger	Neutral	passenger	Yes	No	No	No	Yes
MP18-1	Chondroid	TP53	p.I195F	Shared	17	7578266	T	A	Missense_Mutation	160	0.3625	Yes	1	Clonal	Disease_causing	Passenger	Disease_causing	likely_pathogenic	No	Yes	Yes	Yes	Yes
MP18-2	Ductal	TP53	p.I195F	Shared	17	7578266	T	A	Missense_Mutation	181	0.408839779	Yes	1	Clonal	Disease_causing	Passenger	Disease_causing	likely_pathogenic	No	Yes	Yes	Yes	Yes
MP18-1	Chondroid	TRA2B	p.R47S	Private	3	185644418	T	A	Missense_Mutation	68	0.176470588	No	1	Clonal	Disease_causing	Passenger	Neutral	passenger	No	No	No	No	Yes
MP18-1	Chondroid	TRIM46	p.G621R	Private	1	155154600	G	C	Missense_Mutation	390	0.053846154	No	0.424	Subclonal	Disease_causing	Passenger	Neutral	passenger	Yes	No	No	No	Yes
MP18-1	Chondroid	TUBGCP6	p.P278L	Private	22	50678705	G	A	Missense_Mutation	154	0.116883117	No	0.921	Clonal	Disease_causing	Passenger	Neutral	passenger	Yes	No	No	No	Yes
MP18-1	Chondroid	UBR5	p.I374L	Private	8	103341606	T	G	Missense_Mutation	361	0.047091413	No	0.418	Subclonal	Disease_causing	Driver	Disease_causing	likely_pathogenic	Yes	Yes	No	No	Yes
MP18-1	Chondroid	UHRF1BP1L	p.A194G	Shared	12	100491231	G	C	Missense_Mutation	102	0.215686275	No	1	Clonal	Disease_causing	Passenger	Neutral	passenger	Yes	No	No	No	Not tested
MP18-2	Ductal	UHRF1BP1L	p.A194G	Shared	12	100491231	G	C	Missense_Mutation	86	0.244186047	No	1	Clonal	Disease_causing	Passenger	Neutral	passenger	Yes	No	No	No	Not tested
MP18-1	Chondroid	UPF2	p.D76H	Shared	10	12077197	C	G	Missense_Mutation	388	0.072164948	No	0.568	Subclonal	Disease_causing	Passenger	Neutral	passenger	Yes	No	No	No	Not tested
MP18-2	Ductal	UPF2	p.D76H	Shared	10	12077197	C	G	Missense_Mutation	266	0.345864662	No	1	Clonal	Disease_causing	Passenger	Neutral	passenger	Yes	No	No	No	Not tested
MP18-1	Chondroid	VPS37C	p.P183L	Private	11	60899812	G	A	Missense_Mutation	161	0.068322981	No	0.47	Subclonal	Disease_causing	Passenger	Neutral	passenger	Yes	No	No	No	Yes
MP18-1	Chondroid	WDR63	p.S383N	Private	1	85560213	G	A	Missense_Mutation	240	0.070833333	No	0.487	Subclonal	Disease_causing	Passenger	Neutral	passenger	Yes	No	No	No	Yes
MP18-2	Ductal	WDR74	p.R374P	Private	11	62600461	C	G	Missense_Mutation	205	0.292682927	No	1	Clonal	Neutral	Passenger	Neutral	passenger	Yes	No	No	No	Yes
MP18-1	Chondroid	WDR87	p.S2521R	Private	19	38376631	G	T	Missense_Mutation	311	0.051446945	No	0.405	Subclonal	Neutral	Passenger	Neutral	passenger	Yes	No	No	No	Yes
MP18-1	Chondroid	ZAP70	p.P91A	Private	2	98340770	C	G	Missense_Mutation	396	0.070707071	No	0.557	Subclonal	Neutral	Passenger	Neutral	passenger	No	No	No	No	Yes
MP18-1	Chondroid	ZFYVE28	p.E193D	Private	4	2339166	C	G	Missense_Mutation	185	0.108108108	No	0.743	Subclonal	Disease_causing	Passenger	Neutral	passenger	Yes	No	No	No	Yes
MP18-1	Chondroid	ZNF436	p.E88G	Private	1	23689612	T	C	Missense_Mutation	426	0.169014085	No	1	Clonal	Disease_causing	Passenger	Neutral	passenger	Yes	No	No	No	Yes
MP18-1	Chondroid	ZNF814	p.L815V	Private	19	58384315	G	C	Missense_Mutation	164	0.06097561	No	0.48	Subclonal	Neutral	Passenger	Neutral	passenger	Yes	No	No	No	Yes
MP18-1	Chondroid	ZNF83	p.F108V	Private	19	53117496	A	C	Missense_Mutation	178	0.050561798	No	0.398	Subclonal	Neutral	Passenger	Neutral	passenger	Yes	No	No	No	Yes
MP18-1	Chondroid	ZSCAN23	p.E184D	Private	6	28403241	C	A	Missense_Mutation	235	0.072340426	No	0.497	Subclonal	Neutral	Passenger	Neutral	passenger	Yes	No	No	No	Yes
MP18-1	Chondroid	ZUFSP	p.G232S	Shared	6	116980064	C	T	Missense_Mutation	209	0.220095694	No	1	Clonal	Neutral	Passenger	Neutral	passenger	Yes	No	No	No	Yes
MP18-2	Ductal	ZUFSP	p.G232S	Shared	6	116980064	C	T	Missense_Mutation	154	0.285714286	No	1	Clonal	Neutral	Passenger	Neutral	passenger	Yes	No	No	No	Yes
MP19-1	Chondroid	ACAD11	p.P346T	Shared	3	132347218	G	T	Missense_Mutation	81	0.419753086	No	1	Clonal	Disease_causing	Passenger	Neutral	passenger	No	No	No	No	Yes
MP19-2	Ductal	ACAD11	p.P346T	Shared	3	132347218	G	T	Missense_Mutation	83	0.325301205	No	1	Clonal	Disease_causing	Passenger	Neutral	passenger	No	No	No	No	Yes
MP19-1	Chondroid	ACBD3	p.A114S	Shared	1	226353648	C	A	Missense_Mutation	195	0.394871795	No	1	Clonal	Disease_causing	Passenger	Neutral	passenger	Yes	No	No	No	Yes
MP19-2	Ductal	ACBD3	p.A114S	Shared	1	226353648	C	A	Missense_Mutation	196	0.301020408	No	1	Clonal	Disease_causing	Passenger	Neutral	passenger	Yes	No	No	No	Yes
MP19-1	Chondroid	ADAM23	p.C573S	Shared	2	207437899	T	A	Missense_Mutation	132	0.439393939	No	1	Clonal	Disease_causing	Passenger	Neutral	passenger	No	No	No	No	Yes
MP19-2	Ductal	ADAM23	p.C573S	Shared	2	207437899	T	A	Missense_Mutation	136	0.382352941	No	1	Clonal	Disease_causing	Passenger	Neutral	passenger	No	No	No	No	Yes
MP19-1	Chondroid	ADCY2	p.V695L	Shared	5	7757688	G	T	Missense_Mutation	171	0.304093567	No	1	Clonal	Disease_causing	Passenger	Neutral	passenger	Yes	No	No	No	Yes
MP19-2	Ductal	ADCY2	p.V695L	Shared	5	7757688	G	T	Missense_Mutation	168	0.220238095	No	1	Clonal	Disease_causing	Passenger	Neutral	passenger	Yes	No	No	No	Yes
MP19-1	Chondroid	AFF1	p.S278C	Shared	4	87968520	C	G	Missense_Mutation	576	0.442708333	No	1	Clonal	Disease_causing	Passenger	Neutral	passenger	Yes	Yes	No	No	Yes
MP19-2	Ductal	AFF1	p.S278C	Shared	4	87968520	C	G	Missense_Mutation	509	0.332023576	No	1	Clonal	Disease_causing	Passenger	Neutral	passenger	No	Yes	No	No	Yes
MP19-1	Chondroid	AGPS	p.D217H	Private	2	178305704	G	C	Missense_Mutation	64	0.1875	No	1	Clonal	Disease_causing	Passenger	Disease_causing	likely_pathogenic	No	No	No	No	Not tested
MP19-1	Chondroid	ALS2CR11	p.T372M	Shared	2	202410313	G	A	Missense_Mutation	99	0.515151515	No	1	Clonal	Neutral	Passenger	Neutral	passenger	No	No	No	No	Not tested
MP19-2	Ductal	ALS2CR11	p.T372M	Shared	2	202410313	G	A	Missense_Mutation	87	0.505747126	No	1	Clonal	Neutral	Passenger	Neutral	passenger	No	No	No	No	Not tested
MP19-1	Chondroid	ARHGAP5	p.E555*	Shared	14	32561538	G	T	Nonsense_Mutation	99	0.212121212	No	1	Clonal	Neutral	.	.	passenger	Yes	No	No	No	Not tested
MP19-2	Ductal	ARHGAP5	p.E555*	Shared	14	32561538	G	T	Nonsense_Mutation	100	0.08	No	0.326	Subclonal	Neutral	.	.	passenger	Yes	No	No	No	Not tested
MP19-1	Chondroid	ARID1A	p.T2105I	Shared	1	27106703	C	T	Missense_Mutation	251	0.243027888	No	1	Clonal	Disease_causing	Passenger	Neutral	passenger	Yes	Yes	Yes	Yes	Yes
MP19-2	Ductal	ARID1A	p.T2105I	Shared	1	27106703	C	T	Missense_Mutation	243	0.189300412	No	0.772	Subclonal	Disease_causing	Passenger	Neutral	passenger	Yes	Yes	Yes	Yes	Yes
MP19-1	Chondroid	ASH1L	p.A7D	Shared	1	155491291	G	T	Missense_Mutation	200	0.035	No	0.286	Subclonal	Disease_causing	Passenger	Disease_causing	likely_pathogenic	Yes	No	No	No	Not tested
MP19-2	Ductal	ASH1L	p.A7D	Shared	1	155491291	G	T	Missense_Mutation	223	0.228699552	No	0.932	Clonal	Disease_causing	Passenger	Disease_causing	likely_pathogenic	Yes	No	No	No	Not tested
MP19-2	Ductal	BRIP1	p.K235R	Private	17	59886042	T	C	Missense_Mutation	250	0.044	No	0.223	Subclonal	Disease_causing	Passenger	Neutral	passenger	No	Yes	No	No	Not tested
MP19-1	Chondroid	C16orf74	p.A76V	Shared	16	85741618	G	A	Missense_Mutation	214	0.163551402	No	1	Clonal	Neutral	Passenger	.	passenger	Yes	No	No	No	Not tested
MP19-2	Ductal	C16orf74	p.A76V	Shared	16	85741618	G	A	Missense_Mutation	160	0.13125	No	0.798	Clonal	Neutral	Passenger	.	passenger	Yes	No	No	No	Not tested
MP19-1	Chondroid	C17orf82	p.N103K	Shared	17	59489645	C	G	Missense_Mutation	679	0.094256259	No	0.866	Clonal	Neutral	Passenger	Neutral	passenger	No	No	No	No	Not tested
MP19-2	Ductal	C17orf82	p.N103K	Shared	17	59489645	C	G	Missense_Mutation	537	0.046554935	No	0.236	Subclonal	Neutral	Passenger	Neutral	passenger	No	No	No	No	Not tested
MP19-2	Ductal	C22orf42	p.W229C	Private	22	32545596	C	G	Missense_Mutation	101	0.069306931	No	0.283	Subclonal	Neutral	Passenger	Neutral	passenger	Yes	No	No	No	Not tested
MP19-2	Ductal	C3orf20	p.W51S	Private	3	14724372	G	C	Missense_Mutation	329	0.09118541	No	0.372	Subclonal	Disease_causing	Passenger	Neutral	passenger	No	No	No	No	Not tested
MP19-2	Ductal	CCDC113	p.D73G	Private	16	58286747	A	G	Missense_Mutation	67	0.089552239	No	0.276	Subclonal	Neutral	Passenger	Neutral	passenger	No	No	No	No	Not tested
MP19-1	Chondroid	CCDC9	p.E419*	Shared	19	47774594	G	T	Nonsense_Mutation	235	0.417021277	No	1	Clonal	Disease_causing	.	.	passenger	Yes	No	No	No	Not tested
MP19-2	Ductal	CCDC9	p.E419*	Shared	19	47774594	G	T	Nonsense_Mutation	244	0.31557377	No	1	Clonal	Disease_causing	.	.	passenger	Yes	No	No	No	Not tested
MP19-1	Chondroid	CDC20B	p.G367V	Shared	5	54420746	C	A	Missense_Mutation	133	0.248120301	No	1	Clonal	Neutral	Passenger	Neutral	passenger	No	No	No	No	Not tested
MP19-2	Ductal	CDC20B	p.G367V	Shared	5	54420746	C	A	Missense_Mutation	122	0.319672131	No	0.984	Clonal	Neutral	Passenger	Neutral	passenger	No	No	No	No	Not tested
MP19-1	Chondroid	CDKN2A	p.H83Y	Shared	9	21971111	G	A	Missense_Mutation	213	0.596244131	Yes	1	Clonal	Disease_causing	Driver	Neutral	likely_pathogenic	No	Yes	Yes	Yes	Yes
MP19-2	Ductal	CDKN2A	p.H83Y	Shared	9	21971111	G	A	Missense_Mutation	186	0.61827957	Yes	1	Clonal	Disease_causing	Driver	Neutral	likely_pathogenic	No	Yes	Yes	Yes	Yes
MP19-1	Chondroid	CHDC2	p.K164T	Shared	X	36103505	A	C	Missense_Mutation	229	0.179039301	No	1	Clonal	Neutral	Passenger	.	passenger	Yes	No	No	No	Not tested

MP19-2	Ductal	CHDC2	p.K164T	Shared	X	36103505	A	C	Missense_Mutation	[illegible]	[illegible]	No	0.759	Clonal	Neutral	Passenger	.	passenger	No	No	No	No	Not tested
MP19-1	Chondroid	CHST15	p.L122F	Shared	10	125805363	C	G	Missense_Mutation	150	[illegible]	No	1	Clonal	Neutral	Passenger	.	passenger	No	No	No	No	Not tested
MP19-2	Ductal	CHST15	p.L122F	Shared	10	125805363	C	G	Missense_Mutation	185	0.205405405	No	0.837	Clonal	Neutral	Passenger	.	passenger	No	No	No	No	Not tested
MP19-1	Chondroid	DDX43	p.Q287H	Shared	6	74116140	G	C	Missense_Mutation	221	0.34841629	No	1	Clonal	Disease_causing	Passenger	Neutral	passenger	Yes	No	No	No	Yes
MP19-2	Ductal	DDX43	p.Q287H	Shared	6	74116140	G	C	Missense_Mutation	257	0.338521401	No	1	Clonal	Disease_causing	Passenger	Neutral	passenger	Yes	No	No	No	Yes
MP19-1	Chondroid	DEPDC5	p.T880S	Shared	22	32239663	C	G	Missense_Mutation	134	0.164179104	No	1	Clonal	Disease_causing	Passenger	Neutral	passenger	Yes	No	No	No	Not tested
MP19-2	Ductal	DEPDC5	p.T880S	Shared	22	32239663	C	G	Missense_Mutation	174	0.08045977	No	0.328	Subclonal	Disease_causing	Passenger	Neutral	passenger	Yes	No	No	No	Not tested
MP19-1	Chondroid	DNAJB5	p.R277Q	Shared	9	34996538	G	A	Missense_Mutation	393	0.55216285	No	1	Clonal	Disease_causing	Passenger	Neutral	passenger	No	No	No	No	Not tested
MP19-2	Ductal	DNAJB5	p.R277Q	Shared	9	34996538	G	A	Missense_Mutation	339	0.480825959	No	1	Clonal	Disease_causing	Passenger	Neutral	passenger	No	No	No	No	Not tested
MP19-1	Chondroid	DNAJC1	p.R340I	Shared	10	22094988	C	A	Missense_Mutation	92	0.217391304	No	1	Clonal	Disease_causing	Passenger	Neutral	passenger	Yes	No	No	No	Not tested
MP19-2	Ductal	DNAJC1	p.R340I	Shared	10	22094988	C	A	Missense_Mutation	109	0.128440367	No	0.524	Subclonal	Disease_causing	Passenger	Neutral	passenger	Yes	No	No	No	Not tested
MP19-1	Chondroid	DSG4	p.W3R	Shared	18	28956881	T	A	Missense_Mutation	334	0.074850299	No	0.987	Clonal	Disease_causing	Passenger	Neutral	passenger	Yes	No	No	No	Not tested
MP19-2	Ductal	DSG4	p.W3R	Shared	18	28956881	T	A	Missense_Mutation	271	0.033210332	No	0.268	Subclonal	Disease_causing	Passenger	Neutral	passenger	Yes	No	No	No	Not tested
MP19-1	Chondroid	DYNC1H1	p.D3725N	Shared	14	102505152	G	A	Missense_Mutation	307	0.381107492	No	1	Clonal	Disease_causing	Passenger	Neutral	passenger	Yes	No	No	No	Not tested
MP19-2	Ductal	DYNC1H1	p.D3725N	Shared	14	102505152	G	A	Missense_Mutation	350	0.288571429	No	1	Clonal	Disease_causing	Passenger	Neutral	passenger	Yes	No	No	No	Not tested
MP19-1	Chondroid	DYNC2H1	p.D1304N	Shared	11	103027282	G	A	Missense_Mutation	83	0.060240964	No	0.372	Subclonal	Disease_causing	Passenger	Neutral	passenger	Yes	No	No	No	Not tested
MP19-2	Ductal	DYNC2H1	p.D1304N	Shared	11	103027282	G	A	Missense_Mutation	117	0.094017094	No	0.383	Subclonal	Disease_causing	Passenger	Neutral	passenger	Yes	No	No	No	Not tested
MP19-1	Chondroid	ELF1	p.Y592D	Shared	13	41507647	A	C	Missense_Mutation	141	0.553191489	No	1	Clonal	Neutral	Passenger	Neutral	passenger	No	No	No	No	Not tested
MP19-2	Ductal	ELF1	p.Y592D	Shared	13	41507647	A	C	Missense_Mutation	172	0.377906977	No	1	Clonal	Neutral	Passenger	Neutral	passenger	No	No	No	No	Not tested
MP19-1	Chondroid	EML2	p.W734L	Shared	19	46117958	C	A	Missense_Mutation	159	0.188679245	No	1	Clonal	Disease_causing	Driver	Disease_causing	likely_pathogenic	Yes	No	No	No	Not tested
MP19-2	Ductal	EML2	p.W734L	Shared	19	46117958	C	A	Missense_Mutation	164	0.079268293	No	0.402	Subclonal	Disease_causing	Driver	Disease_causing	likely_pathogenic	Yes	No	No	No	Not tested
MP19-1	Chondroid	FAM159B	p.A80T	Shared	5	63991380	G	A	Missense_Mutation	98	0.071428571	No	0.442	Subclonal	Disease_causing	Passenger	Neutral	passenger	No	No	No	No	Not tested
MP19-2	Ductal	FAM159B	p.A80T	Shared	5	63991380	G	A	Missense_Mutation	100	0.13	No	0.4	Subclonal	Disease_causing	Passenger	Neutral	passenger	No	No	No	No	Not tested
MP19-1	Chondroid	FAM160A1	p.Q639K	Shared	4	152571108	C	A	Missense_Mutation	269	0.144981413	No	1	Clonal	Disease_causing	Passenger	Neutral	passenger	Yes	No	No	No	Not tested
MP19-2	Ductal	FAM160A1	p.Q639K	Shared	4	152571108	C	A	Missense_Mutation	288	0.201388889	No	0.62	Subclonal	Disease_causing	Passenger	Neutral	passenger	No	No	No	No	Not tested
MP19-1	Chondroid	FAM19A2	p.C69W	Shared	12	62148705	G	C	Missense_Mutation	67	0.253731343	No	1	Clonal	Disease_causing	Driver	.	likely_pathogenic	Yes	No	No	No	Not tested
MP19-2	Ductal	FAM19A2	p.C69W	Shared	12	62148705	G	C	Missense_Mutation	69	0.130434783	No	0.532	Subclonal	Disease_causing	Driver	.	likely_pathogenic	Yes	No	No	No	Not tested
MP19-2	Ductal	FOXF2	p.S341T	Private	6	1391204	G	C	Missense_Mutation	156	0.115384615	No	0.47	Subclonal	Neutral	Passenger	Disease_causing	passenger	No	No	No	No	Not tested
MP19-2	Ductal	FUT9	p.D78V	Private	6	96651264	A	T	Missense_Mutation	214	0.088785047	No	0.451	Subclonal	Disease_causing	Passenger	Neutral	passenger	Yes	No	No	No	Not tested
MP19-1	Chondroid	GAD1	p.N20S	Shared	2	171675160	A	G	Missense_Mutation	391	0.217391304	No	1	Clonal	Disease_causing	Passenger	Neutral	passenger	No	No	No	No	Yes
MP19-2	Ductal	GAD1	p.N20S	Shared	2	171675160	A	G	Missense_Mutation	366	0.177595628	No	0.902	Clonal	Disease_causing	Passenger	Neutral	passenger	No	No	No	No	Yes
MP19-1	Chondroid	GOLGB1	p.E2608G	Shared	3	121410388	T	C	Missense_Mutation	167	0.461077844	No	1	Clonal	Disease_causing	Passenger	Neutral	passenger	No	No	No	No	Yes
MP19-2	Ductal	GOLGB1	p.E2608G	Shared	3	121410388	T	C	Missense_Mutation	176	0.431818182	No	1	Clonal	Disease_causing	Passenger	Neutral	passenger	No	No	No	No	Yes
MP19-1	Chondroid	GOLIM4	p.E173D	Shared	3	167759261	C	G	Missense_Mutation	89	0.393258427	No	1	Clonal	Disease_causing	Passenger	.	passenger	No	No	No	No	Not tested
MP19-2	Ductal	GOLIM4	p.E173D	Shared	3	167759261	C	G	Missense_Mutation	89	0.449438202	No	1	Clonal	Disease_causing	Passenger	.	passenger	No	No	No	No	Not tested
MP19-1	Chondroid	GPR112	p.I2810T	Shared	X	135482129	T	C	Missense_Mutation	95	0.126315789	No	1	Clonal	Neutral	Passenger	Neutral	passenger	Yes	No	No	No	Not tested
MP19-2	Ductal	GPR112	p.I2810T	Shared	X	135482129	T	C	Missense_Mutation	88	0.193181818	No	0.788	Clonal	Neutral	Passenger	Neutral	passenger	Yes	No	No	No	Not tested
MP19-1	Chondroid	GPR158	p.P958T	Shared	10	25887427	C	A	Missense_Mutation	148	0.209459459	No	1	Clonal	Neutral	Passenger	Neutral	passenger	Yes	No	No	No	Not tested
MP19-2	Ductal	GPR158	p.P958T	Shared	10	25887427	C	A	Missense_Mutation	148	0.189189189	No	0.771	Subclonal	Neutral	Passenger	Neutral	passenger	Yes	No	No	No	Not tested
MP19-2	Ductal	GRIPAP1	p.E801*	Private	X	48831599	C	A	Nonsense_Mutation	160	0.15	No	0.612	Subclonal	Disease_causing	.	.	passenger	No	No	No	No	Not tested
MP19-2	Ductal	H1FOO	p.I332M	Private	3	129270138	C	G	Missense_Mutation	270	0.055555556	No	0.393	Subclonal	Neutral	Passenger	Neutral	passenger	No	No	No	No	Not tested
MP19-1	Chondroid	HOXC10	p.E248V	Shared	12	54379786	A	T	Missense_Mutation	187	0.427807487	No	1	Clonal	Disease_causing	Passenger	Disease_causing	likely_pathogenic	Yes	No	No	No	Yes
MP19-2	Ductal	HOXC10	p.E248V	Shared	12	54379786	A	T	Missense_Mutation	160	0.4	No	1	Clonal	Disease_causing	Passenger	Disease_causing	likely_pathogenic	Yes	No	No	No	Yes
MP19-1	Chondroid	IFT122	p.Y345C	Shared	3	129195222	A	G	Missense_Mutation	305	0.11147541	No	1	Clonal	Disease_causing	Driver	Neutral	likely_pathogenic	No	No	No	No	Yes
MP19-2	Ductal	IFT122	p.Y345C	Shared	3	129195222	A	G	Missense_Mutation	245	0.13877551	No	0.982	Clonal	Disease_causing	Driver	Neutral	likely_pathogenic	No	No	No	No	Yes
MP19-1	Chondroid	ITPRIPL2	p.A210D	Shared	16	19126412	C	A	Missense_Mutation	405	0.088888889	No	0.727	Subclonal	Disease_causing	Passenger	Neutral	passenger	Yes	No	No	No	Not tested
MP19-2	Ductal	ITPRIPL2	p.A210D	Shared	16	19126412	C	A	Missense_Mutation	307	0.048859935	No	0.199	Subclonal	Disease_causing	Passenger	Neutral	passenger	No	No	No	No	Not tested
MP19-1	Chondroid	KCNJ8	p.G333V	Shared	12	21918934	C	A	Missense_Mutation	125	0.192	No	1	Clonal	Disease_causing	Passenger	Disease_causing	likely_pathogenic	Yes	No	No	No	Not tested
MP19-2	Ductal	KCNJ8	p.G333V	Shared	12	21918934	C	A	Missense_Mutation	154	0.11038961	No	0.45	Subclonal	Disease_causing	Passenger	Disease_causing	likely_pathogenic	Yes	No	No	No	Not tested
MP19-1	Chondroid	KIAA1109	p.E4083K	Shared	4	123260458	G	A	Missense_Mutation	163	0.042944785	No	0.308	Subclonal	Disease_causing	Passenger	Neutral	passenger	Yes	No	No	No	Not tested
MP19-2	Ductal	KIAA1109	p.E4083K	Shared	4	123260458	G	A	Missense_Mutation	175	0.142857143	No	0.44	Subclonal	Disease_causing	Passenger	Neutral	passenger	No	No	No	No	Not tested
MP19-2	Ductal	LGI3	p.X222_splice	Private	8	22009343	C	T	Splice_Site	218	0.055045872	No	0.114	Subclonal	Disease_causing	.	.	likely_pathogenic	No	No	No	No	Not tested
MP19-1	Chondroid	LRRC39	p.E68V	Shared	1	100626038	T	A	Missense_Mutation	112	0.267857143	No	1	Clonal	Disease_causing	Passenger	Neutral	passenger	Yes	No	No	No	Yes
MP19-2	Ductal	LRRC39	p.E68V	Shared	1	100626038	T	A	Missense_Mutation	106	0.254716981	No	0.784	Clonal	Disease_causing	Passenger	Neutral	passenger	Yes	No	No	No	Yes
MP19-2	Ductal	MAGEA11	p.P284T	Private	X	148797996	C	A	Missense_Mutation	488	0.049180328	No	0.201	Subclonal	Neutral	Passenger	Neutral	passenger	Yes	No	No	No	Not tested
MP19-1	Chondroid	MAP7D1	p.S41L	Shared	1	36636647	C	T	Missense_Mutation	291	0.127147766	No	0.913	Clonal	Disease_causing	Passenger	Neutral	passenger	Yes	No	No	No	Not tested
MP19-2	Ductal	MAP7D1	p.S41L	Shared	1	36636647	C	T	Missense_Mutation	289	0.141868512	No	0.578	Subclonal	Disease_causing	Passenger	Neutral	passenger	Yes	No	No	No	Not tested
MP19-1	Chondroid	MEST	p.S45F	Shared	7	130135316	C	T	Missense_Mutation	379	0.110817942	No	1	Clonal	Neutral	Passenger	Neutral	passenger	Yes	No	No	No	Not tested
MP19-2	Ductal	MEST	p.S45F	Shared	7	130135316	C	T	Missense_Mutation	473	0.076109937	No	0.31	Subclonal	Neutral	Passenger	Neutral	passenger	Yes	No	No	No	Not tested
MP19-1	Chondroid	METTL3	p.E226D	Shared	14	21971361	C	G	Missense_Mutation	405	0.162962963	No	1	Clonal	Disease_causing	Passenger	Neutral	passenger	Yes	No	No	No	Yes
MP19-2	Ductal	METTL3	p.E226D	Shared	14	21971361	C	G	Missense_Mutation	398	0.168341709	No	0.686	Subclonal	Disease_causing	Passenger	Neutral	passenger	Yes	No	No	No	Yes
MP19-1	Chondroid	MRPL2	p.L6V	Shared	6	43027104	G	C	Missense_Mutation	623	0.332263242	No	1	Clonal	Neutral	Passenger	Neutral	passenger	Yes	No	No	No	Not tested
MP19-2	Ductal	MRPL2	p.L6V	Shared	6	43027104	G	C	Missense_Mutation	515	0.295145631	No	1	Clonal	Neutral	Passenger	Neutral	passenger	Yes	No	No	No	Not tested
MP19-1	Chondroid	MTF2	p.H547Y	Private	1	93602441	C	T	Missense_Mutation	180	0.038888889	No	0.279	Subclonal	Disease_causing	Passenger	Neutral	passenger	Yes	No	No	No	Yes
MP19-1	Chondroid	MYH8	p.D1624A	Shared	17	10298541	T	G	Missense_Mutation	92	0.576086957	No	1	Clonal	Disease_causing	Passenger	Neutral	passenger	No	No	No	No	Yes
MP19-2	Ductal	MYH8	p.D1624A	Shared	17	10298541	T	G	Missense_Mutation	76	0.368421053	No	1	Clonal	Disease_causing	Passenger	Neutral	passenger	No	No	No	No	Yes
MP19-1	Chondroid	MYT1	p.S50N	Shared	20	62836450	G	A	Missense_Mutation	272	0.113970588	No	0.705	Subclonal	Disease_causing	Driver	Neutral	likely_pathogenic	Yes	No	No	No	Not tested
MP19-2	Ductal	MYT1	p.S50N	Shared	20	62836450	G	A	Missense_Mutation	224	0.120535714	No	0.371	Subclonal	Disease_causing	Driver	Neutral	likely_pathogenic	Yes	No	No	No	Not tested
MP19-1	Chondroid	NEURL4	p.Y467_V470del	Shared	17	7228748	CCACCACACCATA	C	In_Frame_Del	315	0.234920635	No	1	Clonal	.	.	.	.	No	No	No	No	Not tested
MP19-2	Ductal	NEURL4	p.Y467_V470del	Shared	17	7228748	CCACCACACCATA	C	In_Frame_Del	318	0.097484277	No	0.3	Subclonal	.	.	.	.	No	No	No	No	Not tested
MP19-1	Chondroid	NHSL1	p.E844Q	Shared	6	138752964	C	G	Missense_Mutation	451	0.578713969	No	1	Clonal	Disease_causing	Passenger	Neutral	passenger	No	No	No	No	Not tested
MP19-2	Ductal	NHSL1	p.E844Q	Shared	6	138752964	C	G	Missense_Mutation	509	0.52848723	No	1	Clonal	Disease_causing	Passenger	Neutral	passenger	No	No	No	No	Not tested
MP19-2	Ductal	NOBOX	p.D154N	Private	7	144098523	C	T	Missense_Mutation	319	0.034482759	No	0.141	Subclonal	Neutral	Passenger	Disease_causing	passenger	Yes	No	No	No	Not tested
MP19-1	Chondroid	NOD1	p.L384I	Shared	7	30491883	G	T	Missense_Mutation	278	0.291366906	No	1	Clonal	Disease_causing	Passenger	Disease_causing	likely_pathogenic	Yes	No	No	No	Not tested
MP19-2	Ductal	NOD1	p.L384I	Shared	7	30491883	G	T	Missense_Mutation	271	0.295202952	No	1	Clonal	Disease_causing	Passenger	Disease_causing	likely_pathogenic	Yes	No	No	No	Not tested
MP19-1	Chondroid	OAS2	p.Q14H	Shared	12	113416455	G	C	Missense_Mutation	213	0.577464789	No	1	Clonal	Neutral	Passenger	Neutral	passenger	No	No	No	No	Not tested
MP19-2	Ductal	OAS2	p.Q14H	Shared	12	113416455	G	C	Missense_Mutation	208	0.5	No	1	Clonal	Neutral	Passenger	Neutral	passenger	No	No	No	No	Not tested
MP19-2	Ductal	OLFML2B	p.R91K	Private	1	161989875	C	T	Missense_Mutation	688	0.087209302	No	0.356	Subclonal	Disease_causing	Passenger	Neutral	passenger	Yes	No	No	No	Not tested
MP19-1	Chondroid	OR13H1	p.Y258*	Private	X	130678821	T	A	Nonsense_Mutation	194	0.149484536	No	1	Clonal	Disease_causing	.	.	passenger	Yes	No	No	No	Not tested
MP19-1	Chondroid	OR4F15	p.Y35*	Shared	15	102358494	C	A	Nonsense_Mutation	288	0.607638889	No	1	Clonal	Disease_causing	.	.	passenger	Yes	No	No	No	Not tested
MP19-2	Ductal	OR4F15	p.Y35*	Shared	15	102358494	C	A	Nonsense_Mutation	329	0.553191489	No	1	Clonal	Disease_causing	.	.	passenger	Yes	No	No	No	Not tested
MP19-1	Chondroid	OR51T1	p.P158T	Shared	11	4903520	C	A	Missense_Mutation	204	0.18627451	No	1	Clonal	Disease_causing	Passenger	Neutral	passenger	No	No	No	No	Not tested
MP19-2	Ductal	OR51T1	p.P158T	Shared	11	4903520	C	A	Missense_Mutation	216	0.069444444	No	0.283	Subclonal	Disease_causing	Passenger	Neutral	passenger	No	No	No	No	Not tested
MP19-1	Chondroid	PARP14	p.E604D	Shared	3	122419213	G	T	Missense_Mutation	186	0.209677419	No	1	Clonal	Disease_causing	Passenger	Neutral	passenger	No	No	No	No	Not tested
MP19-2	Ductal	PARP14	p.E604D	Shared	3	122419213	G	T	Missense_Mutation	166	0.222891566	No	1	Clonal	Disease_causing	Passenger	Neutral	passenger	No	No	No	No	Not tested
MP19-2	Ductal	PARP15	p.R56C	Private	3	122296680	C	T	Missense_Mutation	52	0.096153846	No	0.488	Subclonal	Neutral	Passenger	Neutral	passenger	No	No	No	No	Yes
MP19-1	Chondroid	PCDH18	p.C98Y	Shared	4	138452950	C	T	Missense_Mutation	183	0.382513661	No	1	Clonal	Disease_causing	Passenger	Neutral	passenger	Yes	No	No	No	Not tested
MP19-2	Ductal	PCDH18	p.C98Y	Shared	4	138452950	C	T	Missense_Mutation	181	0.342541436	No	1	Clonal	Disease_causing	Passenger	Neutral	passenger	No	No	No	No	Not tested
MP19-1	Chondroid	PCF11	p.E580K	Shared	11	82877677	G	A	Missense_Mutation	107	0.308411215	No	1	Clonal	Disease_causing	Passenger	Neutral	passenger	Yes	No	No	No	Not tested
MP19-2	Ductal	PCF11	p.E580K	Shared	11	82877677	G	A	Missense_Mutation	131	0.381679389	No	1	Clonal	Disease_causing	Passenger	Neutral	passenger	Yes	No	No	No	Not tested
MP19-2	Ductal	PCLO	p.T1669A	Private	7	82585264	T	C	Missense_Mutation	286	0.073426573	No	0.373	Subclonal	Disease_causing	Passenger	Neutral	passenger	Yes	No	No	No	Not tested
MP19-2	Ductal	PCSK6	p.A494P	Private	15	101922346	C	G	Missense_Mutation	314	0.089171975	No	0.542	Subclonal	Neutral	Passenger	Neutral	passenger	Yes	No	No	No	Not tested
MP19-1	Chondroid	PHRF1	p.A241T	Private	11	597397	G	A	Missense_Mutation	278	0.035971223	No	0.258	Subclonal	Neutral	Passenger	Neutral	passenger	No	No	No	No	Yes
MP19-1	Chondroid	PLCG1	p.D1165H	Shared	20	39802390	G	C	Missense_Mutation	612	0.419934641	No	1	Clonal	Disease_causing	Passenger	Neutral	passenger	Yes	Yes	No	No	Not tested
MP19-2	Ductal	PLCG1	p.D1165H	Shared	20	39802390	G	C	Missense_Mutation	608	0.325657895	No	1	Clonal	Disease_causing	Passenger	Neutral	passenger	Yes	Yes	No	No	Not tested
MP19-2	Ductal	PRDM10	p.Q908E	Private	11	129784730	G	C	Missense_Mutation	448	0.107142857	No	0.437	Subclonal	Disease_causing	Passenger	Neutral	passenger	Yes	No	No	No	Not tested
MP19-1	Chondroid	PRR25	p.T4N	Shared	16	855453	C	A	Missense_Mutation	134	0.119402985	No	0.977	Clonal	Neutral	Passenger	Neutral	passenger	Yes	No	No	No	Not tested
MP19-2	Ductal	PRR25	p.T4N	Shared	16	855453	C	A	Missense_Mutation	86	0.069767442	No	0.284	Subclonal	Neutral	Passenger	Neutral	passenger	No	No	No	No	Not tested
MP19-2	Ductal	PSD4	p.G522E	Private	2	113943769	G	A	Missense_Mutation	186	0.134408602	No	0.682	Subclonal	Neutral	Passenger	Neutral	passenger	No	No	No	No	Not tested
MP19-1	Chondroid	RELN	p.M1956L	Shared	7	103194210	T	A	Missense_Mutation	199	0.185929648	No	1	Clonal	Neutral	Passenger	Neutral	passenger	Yes	No	No	No	Not tested
MP19-2	Ductal	RELN	p.M1956L	Shared	7	103194210	T	A	Missense_Mutation	214	0.275700935	No	1	Clonal	Neutral	Passenger	Neutral	passenger	Yes	No	No	No	Not tested
MP19-1	Chondroid	RGP1	p.L182F	Shared	9	35751321	G	C	Missense_Mutation	156	0.564102564	No	1	Clonal	Disease_causing	Passenger	.	passenger	No	No	No	No	Not tested
MP19-2	Ductal	RGP1	p.L182F	Shared	9	35751321	G	C	Missense_Mutation	205	0.492682927	No	1	Clonal	Disease_causing	Passenger	.	passenger	No	No	No	No	Not tested
MP19-2	Ductal	SCAND1	p.A125P	Private	20	34541834	C	G	Missense_Mutation	339	0.041297935	No	0.251	Subclonal	Disease_causing	Passenger	Neutral	passenger	Yes	No	No	No	Not tested
MP19-1	Chondroid	SCAP	p.P813S	Shared	3	47459634	G	A	Missense_Mutation	183	0.267759563	No	1	Clonal	Disease_causing	Passenger	Neutral	passenger	No	No	No	No	Not tested
MP19-2	Ductal	SCAP	p.P813S	Shared	3	47459634	G	A	Missense_Mutation	115	0.113043478	No	0.461	Subclonal	Disease_causing	Passenger	Neutral	passenger	No	No	No	No	Not tested
MP19-2	Ductal	SCN4A	p.X367_splice	Private	17	62043605	T	C	Splice_Site	238	0.071428571	No	0.363	Subclonal	Disease_causing	.	.	likely_pathogenic	No	No	No	No	Not tested
MP19-1	Chondroid	SELP	p.R359S	Shared	1	169580800	T	A	Missense_Mutation	141	0.191489362	No	1	Clonal	Neutral	Passenger	Neutral	passenger	Yes	No	No	Yes	Not tested
MP19-2	Ductal	SELP	p.R359S	Shared	1	169580800	T	A	Missense_Mutation	113	0.07079646	No	0.289	Subclonal	Neutral	Passenger	Neutral	passenger	Yes	No	No	Yes	Not tested
MP19-1	Chondroid	SERPINA1	p.W218S	Shared	14	94847472	C	G	Missense_Mutation	58	0.224137931	No	1	Clonal	Disease_causing	Passenger	Disease_causing	likely_pathogenic	No	No	No	No	Not tested
MP19-2	Ductal	SERPINA1	p.W218S	Shared	14	94847472	C	G	Missense_Mutation	64	0.15625	No	0.481	Subclonal	Disease_causing	Passenger	Disease_causing	likely_pathogenic	No	No	No	No	Not tested
MP19-1	Chondroid	SGOL2	p.F1184L	Shared	2	201438619	T	C	Missense_Mutation	130	0.192307692	No	1	Clonal	Neutral	Passenger	Neutral	passenger	No	No	No	No	Not tested
MP19-2	Ductal	SGOL2	p.F1184L	Shared	2	201438619	T	C	Missense_Mutation	168	0.160714286	No	0.816	Clonal	Neutral	Passenger	Neutral	passenger	No	No	No	No	Not tested
MP19-1	Chondroid	SHMT1	p.V33F	Shared	17	18257131	C	A	Missense_Mutation	96	0.104166667	No	0.644	Subclonal	Disease_causing	Passenger	Neutral	passenger	No	No	No	No	Not tested
MP19-2	Ductal	SHMT1	p.V33F	Shared	17	18257131	C	A	Missense_Mutation	126	0.182539683	No	0.562	Subclonal	Disease_causing	Passenger	Neutral	passenger	No	No	No	No	Not tested
MP19-2	Ductal	SIGLEC8	p.E33K	Private	19	51961545	C	T	Missense_Mutation	281	0.085409253	No	0.519	Subclonal	Neutral	Passenger	Neutral	passenger	Yes	No	No	No	Not tested
MP19-2	Ductal	SIPA1L1	p.S992I	Private	14	72139210	G	T	Missense_Mutation	433	0.090069284	No	0.367	Subclonal	Disease_causing	Driver	Neutral	likely_pathogenic	Yes	No	No	No	Not tested
MP19-2	Ductal	SLC11A1	p.E123D	Private	2	219249965	G	C	Missense_Mutation	608	0.128289474	No	0.651	Subclonal	Disease_causing	Passenger	Neutral	passenger	No	No	No	No	Not tested
MP19-1	Chondroid	SLC29A1	p.A174T	Shared	6	44198149	G	A	Missense_Mutation	321	0.152647975	No	1	Clonal	Disease_causing	Passenger	Neutral	passenger	Yes	No	No	No	Not tested
MP19-2	Ductal	SLC29A1	p.A174T	Shared	6	44198149	G	A	Missense_Mutation	278	0.079136691	No	0.402	Subclonal	Disease_causing	Passenger	Neutral	passenger	Yes	No	No	No	Not tested
MP19-2	Ductal	SLC43A2	p.L36Sfs*32	Private	17	1531048	TGAGCATGATGAGCAGC	T	Frame_Shift_Del	259	0.054054054	No	0.166	Subclonal	.	.	.	.	No	No	No	No	Not tested
MP19-1	Chondroid	SLFN5	p.A46S	Shared	17	33585845	G	T	Missense_Mutation	199	0.165829146	No	1	Clonal	Neutral	Passenger	Neutral	passenger	No	No	No	No	Not tested
MP19-2	Ductal	SLFN5	p.A46S	Shared	17	33585845	G	T	Missense_Mutation	196	0.081632653	No	0.251	Subclonal	Neutral	Passenger	Neutral	passenger	No	No	No	No	Not tested
MP19-1	Chondroid	SLITRK2	p.S601L	Shared	X	144905745	C	T	Missense_Mutation	215	0.241860465	No	1	Clonal	Neutral	Passenger	Neutral	passenger	Yes	No	No	No	Not tested
MP19-2	Ductal	SLITRK2	p.S601L	Shared	X	144905745	C	T	Missense_Mutation	203	0.201970443	No	0.823	Clonal	Neutral	Passenger	Neutral	passenger	Yes	No	No	No	Not tested
MP19-1	Chondroid	SMCHD1	p.N373Y	Shared	18	2697106	A	T	Missense_Mutation	151	0.198675497	No	1	Clonal	Disease_causing	Passenger	Neutral	passenger	No	No	No	No	Not tested
MP19-2	Ductal	SMCHD1	p.N373Y	Shared	18	2697106	A	T	Missense_Mutation	194	0.097938144	No	0.301	Subclonal	Disease_causing	Passenger	Neutral	passenger	No	No	No	No	Not tested
MP19-1	Chondroid	SP140	p.E164Q	Shared	2	231106202	G	C	Missense_Mutation	86	0.476744186	No	1	Clonal	Neutral	Passenger	Neutral	passenger	No	No	No	No	Not tested
MP19-2	Ductal	SP140	p.E164Q	Shared	2	231106202	G	C	Missense_Mutation	91	0.406593407	No	1	Clonal	Neutral	Passenger	Neutral	passenger	No	No	No	No	Not tested
MP19-1	Chondroid	SPATA16	p.D392N	Shared	3	172643190	C	T	Missense_Mutation	138	0.326086957	No	1	Clonal	Neutral	Passenger	Neutral	passenger	No	No	No	No	Not tested
MP19-2	Ductal	SPATA16	p.D392N	Shared	3	172643190	C	T	Missense_Mutation	135	0.22962963	No	1	Clonal	Neutral	Passenger	Neutral	passenger	No	No	No	No	Not tested
MP19-1	Chondroid	STAT4	p.K590E	Shared	2	191898300	T	C	Missense_Mutation	226	0.238938053	No	1	Clonal	Disease_causing	Passenger	Disease_causing	likely_pathogenic	No	No	No	No	Not tested
MP19-2	Ductal	STAT4	p.K590E	Shared	2	191898300	T	C	Missense_Mutation	236	0.169491525	No	0.86	Clonal	Disease_causing	Passenger	Disease_causing	likely_pathogenic	No	No	No	No	Not tested
MP19-1	Chondroid	SVEP1	p.V1096L	Shared	9	113228181	C	G	Missense_Mutation	100	0.53	No	1	Clonal	Disease_causing	Passenger	Neutral	passenger	No	No	No	No	Not tested
MP19-2	Ductal	SVEP1	p.V1096L	Shared	9	113228181	C	G	Missense_Mutation	123	0.430894309	No	1	Clonal	Disease_causing	Passenger	Neutral	passenger	No	No	No	No	Not tested
MP19-2	Ductal	TAL1	p.T163N	Private	1	47689729	G	T	Missense_Mutation	64	0.09375	No	0.382	Subclonal	Neutral	Passenger	Disease_causing	passenger	Yes	Yes	No	No	Not tested
MP19-2	Ductal	TCERG1	p.H336Q	Private	5	145843229	C	A	Missense_Mutation	303	0.108910891	No	0.444	Subclonal	Neutral	Passenger	Neutral	passenger	No	No	No	No	Not tested
MP19-1	Chondroid	TCF20	p.W820C	Shared	22	42608852	C	A	Missense_Mutation	165	0.115151515	No	1	Clonal	Disease_causing	Passenger	Neutral	passenger	Yes	No	No	No	Not tested
MP19-1	Chondroid	TCF20	p.Q939H	Shared	22	42608495	T	A	Missense_Mutation	272	0.154411765	No	1	Clonal	Disease_causing	Passenger	Neutral	passenger	Yes	No	No	No	Not tested
MP19-2	Ductal	TCF20	p.W820C	Shared	22	42608852	C	A	Missense_Mutation	184	0.043478261	No	0.177	Subclonal	Disease_causing	Passenger	Neutral	passenger	Yes	No	No	No	Not tested
MP19-2	Ductal	TCF20	p.Q939H	Shared	22	42608495	T	A	Missense_Mutation	272	0.066176471	No	0.27	Subclonal	Disease_causing	Passenger	Neutral	passenger	Yes	No	No	No	Not tested
MP19-1	Chondroid	TGS1	p.E147K	Shared	8	56698896	G	A	Missense_Mutation	106	0.613207547	No	1	Clonal	Neutral	Passenger	Neutral	passenger	No	No	No	No	Not tested
MP19-2	Ductal	TGS1	p.E147K	Shared	8	56698896	G	A	Missense_Mutation	134	0.47761194	No	1	Clonal	Neutral	Passenger	Neutral	passenger	Yes	No	No	No	Not tested
MP19-2	Ductal	TMEM242	p.A69S	Private	6	157739936	C	A	Missense_Mutation	283	0.053003534	No	0.163	Subclonal	Neutral	Passenger	.	passenger	No	No	No	No	Not tested
MP19-1	Chondroid	TNRC6C	p.G301V	Shared	17	76046045	G	T	Missense_Mutation	330	0.16969697	No	1	Clonal	Disease_causing	Passenger	Neutral	passenger	No	No	No	No	Not tested
MP19-2	Ductal	TNRC6C	p.G301V	Shared	17	76046045	G	T	Missense_Mutation	385	0.12987013	No	0.659	Subclonal	Disease_causing	Passenger	Neutral	passenger	No	No	No	No	Not tested
MP19-1	Chondroid	TP53	p.T123Dfs*26	Shared	17	7579321	C	CA	Frame_Shift_Ins	190	0.531578947	No	1	Clonal	.	.	.	.	No	Yes	Yes	Yes	Yes
MP19-2	Ductal	TP53	p.T123Dfs*26	Shared	17	7579321	C	CA	Frame_Shift_Ins	186	0.478494624	No	1	Clonal	.	.	.	.	No	Yes	Yes	Yes	Yes
MP19-1	Chondroid	TRAPPC2	p.S9F	Shared	X	13752226	G	A	Missense_Mutation	235	0.30212766	No	1	Clonal	Neutral	Passenger	Disease_causing	passenger	Yes	No	No	No	Not tested
MP19-2	Ductal	TRAPPC2	p.S9F	Shared	X	13752226	G	A	Missense_Mutation	260	0.253846154	No	1	Clonal	Neutral	Passenger	Disease_causing	passenger	No	No	No	No	Not tested
MP19-2	Ductal	TRIOBP	p.V1652F	Private	22	38131297	G	T	Missense_Mutation	441	0.092970522	No	0.379	Subclonal	Neutral	Passenger	Neutral	passenger	Yes	No	No	No	Not tested
MP19-2	Ductal	TTC18	p.E1101D	Private	10	75013796	C	A	Missense_Mutation	70	0.1	No	0.208	Subclonal	Disease_causing	Passenger	Neutral	passenger	No	No	No	No	Not tested
MP19-1	Chondroid	TTC34	p.R149W	Shared	1	2702459	G	A	Missense_Mutation	95	0.242105263	No	1	Clonal	Neutral	Passenger	Neutral	passenger	Yes	No	No	No	Not tested
MP19-2	Ductal	TTC34	p.R149W	Shared	1	2702459	G	A	Missense_Mutation	79	0.303797468	No	0.935	Clonal	Neutral	Passenger	Neutral	passenger	Yes	No	No	No	Not tested
MP19-2	Ductal	VIPAS39	p.A382T	Private	14	77900220	C	T	Missense_Mutation	91	0.054945055	No	0.224	Subclonal	Disease_causing	Passenger	Neutral	passenger	Yes	No	No	No	Yes
MP19-1	Chondroid	WASF1	p.Q309*	Shared	6	110423388	G	A	Nonsense_Mutation	89	0.191011236	No	1	Clonal	Neutral	.	.	passenger	Yes	No	No	No	Not tested
MP19-2	Ductal	WASF1	p.Q309*	Shared	6	110423388	G	A	Nonsense_Mutation	83	0.060240964	No	0.306	Subclonal	Neutral	.	.	passenger	Yes	No	No	No	Not tested
MP19-1	Chondroid	WDR64	p.G52C	Shared	1	241823840	G	T	Missense_Mutation	153	0.091503268	No	0.932	Clonal	Neutral	Passenger	Neutral	passenger	Yes	No	No	No	Not tested
MP19-2	Ductal	WDR64	p.G52C	Shared	1	241823840	G	T	Missense_Mutation	127	0.125984252	No	0.64	Subclonal	Neutral	Passenger	Neutral	passenger	Yes	No	No	No	Not tested
MP19-2	Ductal	WWC2	p.L446V	Private	4	184182112	C	G	Missense_Mutation	145	0.137931034	No	0.424	Subclonal	Disease_causing	Passenger	Neutral	passenger	No	No	No	No	Not tested
MP19-2	Ductal	ZC3H13	p.K1058E	Private	13	46543507	T	C	Missense_Mutation	228	0.092105263	No	0.283	Subclonal	Disease_causing	Passenger	Neutral	passenger	No	No	No	No	Not tested
MP19-2	Ductal	ZFYVE9	p.S314N	Private	1	52704030	G	A	Missense_Mutation	117	0.102564103	No	0.418	Subclonal	Neutral	Passenger	Neutral	passenger	Yes	No	No	No	Not tested
MP19-2	Ductal	ZNF14	p.N528S	Private	19	19822507	T	C	Missense_Mutation	99	0.121212121	No	0.373	Subclonal	Neutral	Passenger	Neutral	passenger	No	No	No	No	Not tested
MP19-1	Chondroid	ZNF143	p.V482F	Shared	11	9534063	G	T	Missense_Mutation	75	0.68	No	1	Clonal	Disease_causing	Passenger	Neutral	passenger	No	No	No	No	Not tested
MP19-2	Ductal	ZNF143	p.V482F	Shared	11	9534063	G	T	Missense_Mutation	76	0.565789474	No	1	Clonal	Disease_causing	Passenger	Neutral	passenger	No	No	No	No	Not tested
MP19-1	Chondroid	ZNF226	p.R30G	Shared	19	44676313	A	G	Missense_Mutation	214	0.144859813	No	1	Clonal	Neutral	Passenger	Neutral	passenger	Yes	No	No	No	Not tested
MP19-2	Ductal	ZNF226	p.R30G	Shared	19	44676313	A	G	Missense_Mutation	220	0.081818182	No	0.415	Subclonal	Neutral	Passenger	Neutral	passenger	Yes	No	No	No	Not tested
MP19-1	Chondroid	ZNF318	p.A666T	Shared	6	43323076	C	T	Missense_Mutation	318	0.339622642	No	1	Clonal	Neutral	Passenger	Neutral	passenger	Yes	No	No	No	Not tested
MP19-2	Ductal	ZNF318	p.A666T	Shared	6	43323076	C	T	Missense_Mutation	292	0.287671233	No	1	Clonal	Neutral	Passenger	Neutral	passenger	Yes	No	No	No	Not tested
MP19-2	Ductal	ZNF383	p.P52T	Private	19	37726898	C	A	Missense_Mutation	107	0.08411215	No	0.427	Subclonal	Neutral	Passenger	Neutral	passenger	Yes	No	No	No	Yes
MP19-1	Chondroid	ZNF564	p.K312N	Shared	19	12637986	T	G	Missense_Mutation	114	0.228070175	No	1	Clonal	Neutral	Passenger	Neutral	passenger	Yes	No	No	No	Not tested
MP19-2	Ductal	ZNF564	p.K312N	Shared	19	12637986	T	G	Missense_Mutation	124	0.201612903	No	0.822	Clonal	Neutral	Passenger	Neutral	passenger	Yes	No	No	No	Not tested
MP19-2	Ductal	ZNF630	p.E223K	Private	X	47919164	C	T	Missense_Mutation	152	0.059210526	No	0.241	Subclonal	Neutral	Passenger	Neutral	passenger	No	No	No	No	Not tested
MP19-1	Chondroid	ZYG11A	p.C123W	Shared	1	53322782	C	G	Missense_Mutation	74	0.094594595	No	0.679	Subclonal	Disease_causing	Passenger	Neutral	passenger	Yes	No	No	No	Not tested
MP19-2	Ductal	ZYG11A	p.C123W	Shared	1	53322782	C	G	Missense_Mutation	81	0.160493827	No	0.654	Subclonal	Disease_causing	Passenger	Neutral	passenger	Yes	No	No	No	Not tested
MP19-2	Ductal	ZZEF1	p.D1802N	Private	17	3957381	C	T	Missense_Mutation	72	0.069444444	No	0.214	Subclonal	Disease_causing	Passenger	Disease_causing	likely_pathogenic	No	No	No	No	Yes
MP21-2	Ductal	5-Mar	p.Q166*	Private	5	10390532	C	T	Nonsense_Mutation	155	0.180645161	No	0.718	Subclonal	Disease_causing	.	.	passenger	Yes	No	No	No	Not tested
MP21-2	Ductal	ABCA12	p.M1642I	Shared	2	215843579	C	A	Missense_Mutation	113	0.646017699	No	1	Clonal	Disease_causing	Passenger	Disease_causing	likely_pathogenic	Yes	No	No	No	Not tested
MP21-R1	Spindle	ABCA12	p.M1642I	Shared	2	215843579	C	A	Missense_Mutation	73	0.780821918	No	1	Clonal	Disease_causing	Passenger	Disease_causing	likely_pathogenic	Yes	No	No	No	Not tested
MP21-R2	Spindle	ABCA12	p.M1642I	Shared	2	215843579	C	A	Missense_Mutation	54	0.666666667	No	1	Clonal	Disease_causing	Passenger	Disease_causing	likely_pathogenic	Yes	No	No	No	Not tested
MP21-2	Ductal	ABCC12	p.F174S	Private	16	48174734	A	G	Missense_Mutation	206	0.286407767	No	1	Clonal	Disease_causing	Passenger	Disease_causing	likely_pathogenic	No	No	No	No	Yes
MP21-2	Ductal	ADAMTS2	p.R1047H	Shared	5	178548700	C	T	Missense_Mutation	296	0.662162162	No	1	Clonal	Disease_causing	Passenger	Neutral	passenger	Yes	No	No	No	Yes
MP21-R1	Spindle	ADAMTS2	p.R1047H	Shared	5	178548700	C	T	Missense_Mutation	924	0.873376623	No	1	Clonal	Disease_causing	Passenger	Neutral	passenger	Yes	No	No	No	Yes
MP21-R2	Spindle	ADAMTS2	p.R1047H	Shared	5	178548700	C	T	Missense_Mutation	559	0.819320215	No	1	Clonal	Disease_causing	Passenger	Neutral	passenger	Yes	No	No	No	Yes
MP21-2	Ductal	AKAP6	p.G205R	Private	14	33014472	G	C	Missense_Mutation	232	0.043103448	No	0.171	Subclonal	Disease_causing	Passenger	Neutral	passenger	No	No	No	No	Yes
MP21-2	Ductal	ARHGEF15	p.F552L	Private	17	8219418	C	A	Missense_Mutation	317	0.063091483	No	0.188	Subclonal	Disease_causing	Passenger	Neutral	passenger	Yes	No	No	No	Not tested
MP21-2	Ductal	ARRDC2	p.E334Q	Shared	19	18121155	G	C	Missense_Mutation	164	0.317073171	No	1	Clonal	Disease_causing	Passenger	Neutral	passenger	No	No	No	No	Yes
MP21-R1	Spindle	ARRDC2	p.E334Q	Shared	19	18121155	G	C	Missense_Mutation	371	0.447439353	No	1	Clonal	Disease_causing	Passenger	Neutral	passenger	No	No	No	No	Yes
MP21-R2	Spindle	ARRDC2	p.E334Q	Shared	19	18121155	G	C	Missense_Mutation	222	0.373873874	No	1	Clonal	Disease_causing	Passenger	Neutral	passenger	No	No	No	No	Yes
MP21-2	Ductal	BARHL1	p.T186S	Private	9	135462806	C	G	Missense_Mutation	221	0.045248869	No	0.135	Subclonal	Disease_causing	Passenger	Disease_causing	likely_pathogenic	Yes	No	No	No	Not tested
MP21-R1	Spindle	BFSP2	p.V8M	Shared	3	133118949	G	A	Missense_Mutation	780	0.406410256	No	1	Clonal	Neutral	Passenger	Disease_causing	passenger	Yes	No	No	No	Not tested
MP21-R2	Spindle	BFSP2	p.V8M	Shared	3	133118949	G	A	Missense_Mutation	507	0.353057199	No	1	Clonal	Neutral	Passenger	Disease_causing	passenger	Yes	No	No	No	Not tested
MP21-2	Ductal	C1QTNF1	p.K213*	Shared	17	77043961	A	T	Nonsense_Mutation	562	0.734875445	No	1	Clonal	Disease_causing	.	.	likely_pathogenic	Yes	No	No	No	Yes
MP21-R1	Spindle	C1QTNF1	p.K213*	Shared	17	77043961	A	T	Nonsense_Mutation	902	0.851441242	No	1	Clonal	Disease_causing	.	.	likely_pathogenic	Yes	No	No	No	Yes
MP21-R2	Spindle	C1QTNF1	p.K213*	Shared	17	77043961	A	T	Nonsense_Mutation	527	0.857685009	No	1	Clonal	Disease_causing	.	.	likely_pathogenic	Yes	No	No	No	Yes
MP21-2	Ductal	CCDC120	p.S290L	Private	X	48923289	C	T	Missense_Mutation	389	0.249357326	No	0.991	Clonal	Disease_causing	Passenger	.	passenger	No	No	No	Yes	Not tested
MP21-2	Ductal	CDC42BPG	p.V1060L	Shared	11	64599103	C	G	Missense_Mutation	469	0.326226013	No	1	Clonal	Neutral	Passenger	Neutral	passenger	No	No	No	No	Not tested
MP21-R1	Spindle	CDC42BPG	p.V1060L	Shared	11	64599103	C	G	Missense_Mutation	1069	0.376987839	No	1	Clonal	Neutral	Passenger	Neutral	passenger	No	No	No	No	Not tested
MP21-R2	Spindle	CDC42BPG	p.V1060L	Shared	11	64599103	C	G	Missense_Mutation	681	0.400881057	No	1	Clonal	Neutral	Passenger	Neutral	passenger	No	No	No	No	Not tested
MP21-2	Ductal	CDCP2	p.P51H	Private	1	54610414	G	T	Missense_Mutation	308	0.042207792	No	0.168	Subclonal	Disease_causing	Passenger	Neutral	passenger	Yes	No	No	No	Yes
MP21-R1	Spindle	COL20A1	p.I550M	Shared	20	61943087	C	G	Missense_Mutation	940	0.494680851	No	1	Clonal	Neutral	Passenger	Disease_causing	passenger	No	No	No	No	Not tested
MP21-R2	Spindle	COL20A1	p.I550M	Shared	20	61943087	C	G	Missense_Mutation	553	0.481012658	No	1	Clonal	Neutral	Passenger	Disease_causing	passenger	No	No	No	No	Not tested
MP21-2	Ductal	COL4A6	p.X840_splice	Shared	X	107420242	C	T	Splice_Site	185	0.572972973	No	1	Clonal	Disease_causing	.	.	likely_pathogenic	No	No	No	No	Yes
MP21-R1	Spindle	COL4A6	p.X840_splice	Shared	X	107420242	C	T	Splice_Site	127	0.763779528	No	1	Clonal	Disease_causing	.	.	likely_pathogenic	No	No	No	No	Yes
MP21-R2	Spindle	COL4A6	p.X840_splice	Shared	X	107420242	C	T	Splice_Site	103	0.699029126	No	1	Clonal	Disease_causing	.	.	likely_pathogenic	No	No	No	No	Yes
MP21-2	Ductal	CRYBB1	p.G111W	Shared	22	27003954	C	A	Missense_Mutation	295	0.4	No	1	Clonal	Disease_causing	Passenger	Disease_causing	likely_pathogenic	Yes	No	No	No	Not tested
MP21-R1	Spindle	CRYBB1	p.G111W	Shared	22	27003954	C	A	Missense_Mutation	311	0.736334405	No	1	Clonal	Disease_causing	Passenger	Disease_causing	likely_pathogenic	Yes	No	No	No	Not tested
MP21-R2	Spindle	CRYBB1	p.G111W	Shared	22	27003954	C	A	Missense_Mutation	267	0.647940075	No	1	Clonal	Disease_causing	Passenger	Disease_causing	likely_pathogenic	Yes	No	No	No	Not tested
MP21-2	Ductal	CUL7	p.E163*	Shared	6	43020196	C	A	Nonsense_Mutation	570	0.275438596	No	1	Clonal	Disease_causing	.	.	passenger	No	No	No	No	Not tested
MP21-R1	Spindle	CUL7	p.E163*	Shared	6	43020196	C	A	Nonsense_Mutation	715	0.413986014	No	1	Clonal	Disease_causing	.	.	passenger	No	No	No	No	Not tested
MP21-R2	Spindle	CUL7	p.E163*	Shared	6	43020196	C	A	Nonsense_Mutation	486	0.440329218	No	1	Clonal	Disease_causing	.	.	passenger	No	No	No	No	Not tested
MP21-2	Ductal	CYB5RL	p.E49D	Shared	1	54661143	C	G	Missense_Mutation	94	0.606382979	No	1	Clonal	Disease_causing	Passenger	Neutral	passenger	Yes	No	No	No	Yes
MP21-R1	Spindle	CYB5RL	p.E49D	Shared	1	54661143	C	G	Missense_Mutation	170	0.794117647	No	1	Clonal	Disease_causing	Passenger	Neutral	passenger	Yes	No	No	No	Yes
MP21-R2	Spindle	CYB5RL	p.E49D	Shared	1	54661143	C	G	Missense_Mutation	99	0.797979798	No	1	Clonal	Disease_causing	Passenger	Neutral	passenger	Yes	No	No	No	Yes
MP21-2	Ductal	CYP2E1	p.D190*	Private	10	135345703	ATTTTGACTACAATGATGAGAAG	A	Frame_Shift_Del	187	0.213903743	No	0.85	Clonal	.	.	.	.	No	No	No	No	Yes
MP21-R1	Spindle	DEPDC5	p.R165C	Shared	22	32179902	C	T	Missense_Mutation	102	0.774509804	No	1	Clonal	Disease_causing	Passenger	Neutral	passenger	Yes	No	No	No	Not tested
MP21-R2	Spindle	DEPDC5	p.R165C	Shared	22	32179902	C	T	Missense_Mutation	69	0.68115942	No	1	Clonal	Disease_causing	Passenger	Neutral	passenger	Yes	No	No	No	Not tested
MP21-2	Ductal	DNAH1	p.R4250*	Private	3	52434412	C	T	Nonsense_Mutation	161	0.080745342	No	0.24	Subclonal	Disease_causing	.	.	likely_pathogenic	Yes	No	No	No	Yes
MP21-2	Ductal	FADS2	p.R114W	Shared	11	61607827	C	T	Missense_Mutation	182	0.357142857	No	1	Clonal	Disease_causing	Passenger	Neutral	passenger	No	No	No	No	Yes
MP21-R1	Spindle	FADS2	p.R114W	Shared	11	61607827	C	T	Missense_Mutation	273	0.457875458	No	1	Clonal	Disease_causing	Passenger	Neutral	passenger	No	No	No	No	Yes
MP21-R2	Spindle	FADS2	p.R114W	Shared	11	61607827	C	T	Missense_Mutation	194	0.381443299	No	1	Clonal	Disease_causing	Passenger	Neutral	passenger	No	No	No	No	Yes
MP21-2	Ductal	FOXA3	p.R180P	Private	19	46375802	G	C	Missense_Mutation	287	0.097560976	No	0.29	Subclonal	Disease_causing	Passenger	Disease_causing	likely_pathogenic	Yes	No	No	No	Yes
MP21-2	Ductal	GABRA6	p.A410T	Shared	5	161128645	G	A	Missense_Mutation	253	0.624505929	No	1	Clonal	Neutral	Passenger	Disease_causing	passenger	Yes	No	No	No	Not tested
MP21-R1	Spindle	GABRA6	p.A410T	Shared	5	161128645	G	A	Missense_Mutation	276	0.873188406	No	1	Clonal	Neutral	Passenger	Disease_causing	passenger	Yes	No	No	No	Not tested
MP21-R2	Spindle	GABRA6	p.A410T	Shared	5	161128645	G	A	Missense_Mutation	160	0.8375	No	1	Clonal	Neutral	Passenger	Disease_causing	passenger	Yes	No	No	No	Not tested
MP21-2	Ductal	GCNT1	p.L203R	Shared	9	79117905	T	G	Missense_Mutation	278	0.374100719	No	1	Clonal	Disease_causing	Driver	Neutral	likely_pathogenic	No	No	No	No	Yes
MP21-R1	Spindle	GCNT1	p.L203R	Shared	9	79117905	T	G	Missense_Mutation	166	0.439759036	No	1	Clonal	Disease_causing	Driver	Neutral	likely_pathogenic	No	No	No	No	Yes
MP21-R2	Spindle	GCNT1	p.L203R	Shared	9	79117905	T	G	Missense_Mutation	109	0.449541284	No	1	Clonal	Disease_causing	Driver	Neutral	likely_pathogenic	No	No	No	No	Yes
MP21-2	Ductal	GFPT1	p.Q404H	Private	2	69565689	T	A	Missense_Mutation	313	0.067092652	No	0.334	Subclonal	Disease_causing	Passenger	Neutral	passenger	Yes	No	No	No	Yes
MP21-R1	Spindle	HES5	p.K73R	Shared	1	2461287	T	C	Missense_Mutation	258	0.798449612	No	1	Clonal	Neutral	Passenger	Neutral	passenger	Yes	No	No	No	Not tested
MP21-R2	Spindle	HES5	p.K73R	Shared	1	2461287	T	C	Missense_Mutation	140	0.707142857	No	1	Clonal	Neutral	Passenger	Neutral	passenger	Yes	No	No	No	Not tested
MP21-R1	Spindle	HGS	p.A756T	Shared	17	79668580	G	A	Missense_Mutation	333	0.42042042	No	1	Clonal	Disease_causing	Passenger	Neutral	passenger	Yes	No	No	No	Not tested
MP21-R2	Spindle	HGS	p.A756T	Shared	17	79668580	G	A	Missense_Mutation	209	0.368421053	No	1	Clonal	Disease_causing	Passenger	Neutral	passenger	Yes	No	No	No	Not tested
MP21-2	Ductal	IMMT	p.M475L	Private	2	86374935	T	G	Missense_Mutation	267	0.052434457	No	0.261	Subclonal	Disease_causing	Passenger	Neutral	passenger	Yes	No	No	No	Yes
MP21-2	Ductal	KCNK4	p.A326V	Shared	11	64066993	C	T	Missense_Mutation	483	0.370600414	No	1	Clonal	Neutral	Passenger	Neutral	passenger	No	No	No	No	Not tested
MP21-R1	Spindle	KCNK4	p.A326V	Shared	11	64066993	C	T	Missense_Mutation	992	0.4375	No	1	Clonal	Neutral	Passenger	Neutral	passenger	No	No	No	No	Not tested
MP21-R2	Spindle	KCNK4	p.A326V	Shared	11	64066993	C	T	Missense_Mutation	674	0.402077151	No	1	Clonal	Neutral	Passenger	Neutral	passenger	No	No	No	No	Not tested
MP21-2	Ductal	KCNS1	p.R60W	Private	20	43727235	G	A	Missense_Mutation	134	0.044776119	No	0.178	Subclonal	Disease_causing	Passenger	Neutral	passenger	No	No	No	No	Not tested
MP21-2	Ductal	KIAA0319	p.W72C	Private	6	24596686	C	A	Missense_Mutation	390	0.112820513	No	0.449	Subclonal	Disease_causing	Passenger	Neutral	passenger	No	No	No	No	Not tested
MP21-2	Ductal	KIAA2026	p.E946Q	Private	9	5923160	C	G	Missense_Mutation	264	0.087121212	No	0.259	Subclonal	Neutral	Passenger	.	passenger	Yes	No	No	No	Yes
MP21-R1	Spindle	LDB3	p.K243R	Shared	10	88446864	A	G	Missense_Mutation	593	0.401349073	No	1	Clonal	Disease_causing	Passenger	Disease_causing	likely_pathogenic	Yes	No	No	No	Not tested
MP21-R2	Spindle	LDB3	p.K243R	Shared	10	88446864	A	G	Missense_Mutation	355	0.402816901	No	1	Clonal	Disease_causing	Passenger	Disease_causing	likely_pathogenic	Yes	No	No	No	Not tested
MP21-2	Ductal	MAP3K9	p.D389Y	Shared	14	71215707	C	A	Missense_Mutation	101	0.376237624	No	1	Clonal	Disease_causing	Driver	Disease_causing	likely_pathogenic	Yes	No	No	No	Yes
MP21-R1	Spindle	MAP3K9	p.D389Y	Shared	14	71215707	C	A	Missense_Mutation	79	0.430379747	No	0.964	Clonal	Disease_causing	Driver	Disease_causing	likely_pathogenic	Yes	No	No	No	Yes
MP21-R2	Spindle	MAP3K9	p.D389Y	Shared	14	71215707	C	A	Missense_Mutation	50	0.54	No	1	Clonal	Disease_causing	Driver	Disease_causing	likely_pathogenic	Yes	No	No	No	Yes
MP21-2	Ductal	MOCOS	p.M655L	Private	18	33828887	A	T	Missense_Mutation	250	0.112	No	0.445	Subclonal	Neutral	Passenger	Neutral	passenger	No	No	No	No	Yes
MP21-R1	Spindle	MS4A4A	p.T213I	Shared	11	60073664	C	T	Missense_Mutation	228	0.429824561	No	1	Clonal	Neutral	Passenger	Neutral	passenger	No	No	No	No	Not tested
MP21-R2	Spindle	MS4A4A	p.T213I	Shared	11	60073664	C	T	Missense_Mutation	137	0.328467153	No	1	Clonal	Neutral	Passenger	Neutral	passenger	No	No	No	No	Not tested
MP21-2	Ductal	MYEF2	p.G384R	Private	15	48444128	C	G	Missense_Mutation	213	0.0657277	No	0.196	Subclonal	Disease_causing	Passenger	Neutral	passenger	Yes	No	No	No	Not tested
MP21-2	Ductal	MYH6	p.S1414C	Private	14	23857482	G	C	Missense_Mutation	375	0.08	No	0.318	Subclonal	Disease_causing	Passenger	Neutral	passenger	No	No	No	No	Yes
MP21-2	Ductal	MYO3B	p.E1152K	Shared	2	171371514	G	A	Missense_Mutation	156	0.58974359	No	1	Clonal	Neutral	Passenger	Neutral	passenger	Yes	No	No	No	Not tested
MP21-R1	Spindle	MYO3B	p.E1152K	Shared	2	171371514	G	A	Missense_Mutation	110	0.754545455	No	1	Clonal	Neutral	Passenger	Neutral	passenger	Yes	No	No	No	Not tested
MP21-R2	Spindle	MYO3B	p.E1152K	Shared	2	171371514	G	A	Missense_Mutation	109	0.71559633	No	1	Clonal	Neutral	Passenger	Neutral	passenger	Yes	No	No	No	Not tested
MP21-R1	Spindle	NLGN1	p.P309S	Shared	3	173996716	C	T	Missense_Mutation	223	0.201793722	No	1	Clonal	Disease_causing	Passenger	Neutral	passenger	Yes	No	No	No	Not tested
MP21-R2	Spindle	NLGN1	p.P309S	Shared	3	173996716	C	T	Missense_Mutation	125	0.184	No	0.997	Clonal	Disease_causing	Passenger	Neutral	passenger	Yes	No	No	No	Not tested
MP21-2	Ductal	OLFML2B	p.R277Q	Shared	1	161970022	C	T	Missense_Mutation	210	0.338095238	No	1	Clonal	Neutral	Passenger	Disease_causing	passenger	No	No	No	No	Not tested
MP21-R1	Spindle	OLFML2B	p.R277Q	Shared	1	161970022	C	T	Missense_Mutation	776	0.213917526	No	1	Clonal	Neutral	Passenger	Disease_causing	passenger	No	No	No	No	Not tested
MP21-R2	Spindle	OLFML2B	p.R277Q	Shared	1	161970022	C	T	Missense_Mutation	428	0.21728972	No	1	Clonal	Neutral	Passenger	Disease_causing	passenger	No	No	No	No	Not tested
MP21-2	Ductal	PCIF1	p.I456V	Shared	20	44574692	A	G	Missense_Mutation	500	0.346	No	1	Clonal	Neutral	Passenger	.	passenger	No	No	No	No	Not tested
MP21-R1	Spindle	PCIF1	p.I456V	Shared	20	44574692	A	G	Missense_Mutation	997	0.416248746	No	1	Clonal	Neutral	Passenger	.	passenger	No	No	No	No	Not tested
MP21-R2	Spindle	PCIF1	p.I456V	Shared	20	44574692	A	G	Missense_Mutation	590	0.427118644	No	1	Clonal	Neutral	Passenger	.	passenger	No	No	No	No	Not tested
MP21-2	Ductal	PKHD1L1	p.W2625R	Private	8	110476934	T	C	Missense_Mutation	514	0.056420233	No	0.281	Subclonal	Disease_causing	Passenger	Neutral	passenger	No	No	No	No	Yes

MP21-2	Ductal	PNPLA6	p.R442Pfs*33	Private	19	7607490	G	CCGACTTCC	Frame_Shift_Ins	[illegible]	[illegible]	No	0.634	Subclonal	.	.	.	.	No	No	No	No	Yes
MP21-2	Ductal	PPP1R13B	p.G932S	Private	14	104205086	C	T	Missense_Mutation	378	[illegible]	No	0.276	Subclonal	Disease_causing	Passenger	Neutral	passenger	Yes	No	No	No	Not tested
MP21-2	Ductal	RNF213	p.G1885R	Private	17	78313820	G	C	Missense_Mutation	366	0.076502732	No	0.381	Subclonal	Disease_causing	Passenger	Neutral	passenger	Yes	No	No	No	Not tested
MP21-2	Ductal	RUNX1	p.S77G	Shared	21	36259262	T	C	Missense_Mutation	117	0.538461538	No	1	Clonal	Disease_causing	Passenger	Disease_causing	likely_pathogenic	Yes	Yes	Yes	Yes	Yes
MP21-R1	Spindle	RUNX1	p.S77G	Shared	21	36259262	T	C	Missense_Mutation	233	0.802575107	No	1	Clonal	Disease_causing	Passenger	Disease_causing	likely_pathogenic	Yes	Yes	Yes	Yes	Yes
MP21-R2	Spindle	RUNX1	p.S77G	Shared	21	36259262	T	C	Missense_Mutation	117	0.683760684	No	1	Clonal	Disease_causing	Passenger	Disease_causing	likely_pathogenic	Yes	Yes	Yes	Yes	Yes
MP21-R1	Spindle	SCNN1A	p.R198S	Shared	12	6472876	C	A	Missense_Mutation	504	0.347222222	No	1	Clonal	Disease_causing	Passenger	Neutral	passenger	No	No	No	No	Not tested
MP21-R2	Spindle	SCNN1A	p.R198S	Shared	12	6472876	C	A	Missense_Mutation	298	0.32885906	No	1	Clonal	Disease_causing	Passenger	Neutral	passenger	No	No	No	No	Not tested
MP21-2	Ductal	SCNN1D	p.P157T	Shared	1	1220955	C	A	Missense_Mutation	97	0.319587629	No	1	Clonal	Disease_causing	Passenger	Neutral	passenger	Yes	No	No	No	Not tested
MP21-R1	Spindle	SCNN1D	p.P157T	Shared	1	1220955	C	A	Missense_Mutation	221	0.366515837	No	1	Clonal	Disease_causing	Passenger	Neutral	passenger	Yes	No	No	No	Not tested
MP21-R2	Spindle	SCNN1D	p.P157T	Shared	1	1220955	C	A	Missense_Mutation	109	0.431192661	No	1	Clonal	Disease_causing	Passenger	Neutral	passenger	Yes	No	No	No	Not tested
MP21-2	Ductal	SEC23B	p.T381S	Private	20	18511356	C	G	Missense_Mutation	183	0.103825137	No	0.309	Subclonal	Disease_causing	Passenger	Neutral	passenger	Yes	No	No	No	Yes
MP21-R1	Spindle	SELENBP1	p.I390M	Shared	1	151337488	G	C	Missense_Mutation	1484	0.132075472	No	0.692	Subclonal	Disease_causing	Passenger	Neutral	passenger	No	No	No	No	Not tested
MP21-R2	Spindle	SELENBP1	p.I390M	Shared	1	151337488	G	C	Missense_Mutation	1019	0.153091266	No	0.983	Clonal	Disease_causing	Passenger	Neutral	passenger	No	No	No	No	Not tested
MP21-2	Ductal	SETBP1	p.X162_splice	Private	18	42281799	T	C	Splice_Site	178	0.08988764	No	0.357	Subclonal	Disease_causing	.	.	likely_pathogenic	No	Yes	Yes	No	Yes
MP21-2	Ductal	SMARCC2	p.V132M	Private	12	56578819	C	T	Missense_Mutation	90	0.077777778	No	0.231	Subclonal	Disease_causing	Passenger	Neutral	passenger	Yes	No	No	No	Yes
MP21-2	Ductal	SRSF4	p.R125C	Shared	1	29481413	G	A	Missense_Mutation	218	0.73853211	No	1	Clonal	Disease_causing	Passenger	Neutral	passenger	Yes	No	No	No	Not tested
MP21-R1	Spindle	SRSF4	p.R125C	Shared	1	29481413	G	A	Missense_Mutation	111	0.846846847	No	1	Clonal	Disease_causing	Passenger	Neutral	passenger	Yes	No	No	No	Not tested
MP21-R2	Spindle	SRSF4	p.R125C	Shared	1	29481413	G	A	Missense_Mutation	82	0.756097561	No	1	Clonal	Disease_causing	Passenger	Neutral	passenger	Yes	No	No	No	Not tested
MP21-2	Ductal	STAB2	p.V1621M	Shared	12	104121054	G	A	Missense_Mutation	336	0.580357143	No	1	Clonal	Neutral	Passenger	Disease_causing	passenger	Yes	No	No	No	Not tested
MP21-R1	Spindle	STAB2	p.V1621M	Shared	12	104121054	G	A	Missense_Mutation	312	0.78525641	No	1	Clonal	Neutral	Passenger	Disease_causing	passenger	Yes	No	No	No	Not tested
MP21-R2	Spindle	STAB2	p.V1621M	Shared	12	104121054	G	A	Missense_Mutation	237	0.708860759	No	1	Clonal	Neutral	Passenger	Disease_causing	passenger	Yes	No	No	No	Not tested
MP21-2	Ductal	STIP1	p.R470H	Private	11	63970944	G	A	Missense_Mutation	177	0.04519774	No	0.18	Subclonal	Disease_causing	Passenger	Neutral	passenger	No	No	No	No	Yes
MP21-2	Ductal	TAF1	p.K258T	Private	X	70597040	A	C	Missense_Mutation	237	0.054852321	No	0.218	Subclonal	Disease_causing	Driver	Neutral	likely_pathogenic	No	No	Yes	No	Yes
MP21-R2	Spindle	TAF5L	p.E236D	Private	1	229738206	C	G	Missense_Mutation	584	0.104452055	No	0.462	Subclonal	Disease_causing	Passenger	Neutral	passenger	No	No	No	No	Not tested
MP21-R1	Spindle	THBS3	p.G656A	Shared	1	155168307	C	G	Missense_Mutation	713	0.217391304	No	1	Clonal	Disease_causing	Passenger	Disease_causing	likely_pathogenic	No	No	No	No	Not tested
MP21-R2	Spindle	THBS3	p.G656A	Shared	1	155168307	C	G	Missense_Mutation	548	0.140510949	No	0.902	Clonal	Disease_causing	Passenger	Disease_causing	likely_pathogenic	No	No	No	No	Not tested
MP21-R1	Spindle	TMEM161A	p.M203Tfs*2	Shared	19	19232616	A	ACTAAG	Nonsense_Mutation	485	0.156701031	No	0.508	Subclonal	.	.	.	.	No	No	No	No	Not tested
MP21-R2	Spindle	TMEM161A	p.M203Tfs*2	Shared	19	19232616	A	ACTAAG	Nonsense_Mutation	334	0.116766467	No	0.399	Subclonal	.	.	.	.	No	No	No	No	Not tested
MP21-2	Ductal	TP53	p.V173G	Shared	17	7578412	A	C	Missense_Mutation	302	0.552980132	Yes	1	Clonal	Disease_causing	Passenger	Disease_causing	likely_pathogenic	Yes	Yes	Yes	Yes	Not tested
MP21-R1	Spindle	TP53	p.V173G	Shared	17	7578412	A	C	Missense_Mutation	662	0.773413897	Yes	1	Clonal	Disease_causing	Passenger	Disease_causing	likely_pathogenic	Yes	Yes	Yes	Yes	Not tested
MP21-R2	Spindle	TP53	p.V173G	Shared	17	7578412	A	C	Missense_Mutation	472	0.752118644	Yes	1	Clonal	Disease_causing	Passenger	Disease_causing	likely_pathogenic	Yes	Yes	Yes	Yes	Not tested
MP21-2	Ductal	TSR2	p.R9*	Private	X	54466879	C	T	Nonsense_Mutation	243	0.251028807	No	0.998	Clonal	Neutral	.	.	likely_pathogenic	No	No	No	No	Not tested
MP21-2	Ductal	TUT1	p.R573H	Private	11	62343587	C	T	Missense_Mutation	419	0.088305489	No	0.351	Subclonal	Disease_causing	Passenger	Neutral	passenger	No	No	No	No	Yes
MP21-2	Ductal	USP28	p.D265Y	Private	11	113702682	C	A	Missense_Mutation	98	0.683673469	No	1	Clonal	Disease_causing	Passenger	Neutral	passenger	Yes	No	No	No	Yes
MP21-2	Ductal	VILL	p.S233R	Shared	3	38039111	C	G	Missense_Mutation	137	0.343065693	No	1	Clonal	Neutral	Passenger	Neutral	passenger	Yes	No	No	No	Not tested
MP21-R1	Spindle	VILL	p.S233R	Shared	3	38039111	C	G	Missense_Mutation	396	0.464646465	No	1	Clonal	Neutral	Passenger	Neutral	passenger	Yes	No	No	No	Not tested
MP21-R2	Spindle	VILL	p.S233R	Shared	3	38039111	C	G	Missense_Mutation	242	0.433884298	No	1	Clonal	Neutral	Passenger	Neutral	passenger	Yes	No	No	No	Not tested
MP27-1	Chondroid	ABCA13	p.R3784Q	Private	7	48427434	G	A	Missense_Mutation	100	0.13	No	0.594	Subclonal	Neutral	Passenger	Disease_causing	passenger	No	No	No	No	Not tested
MP27-1	Chondroid	ABCB1	p.R157G	Private	7	87196162	G	C	Missense_Mutation	115	0.086956522	No	0.397	Subclonal	Disease_causing	Passenger	Neutral	passenger	No	No	No	No	Not tested
MP27-3	Ductal	ADAM9	p.R202L	Private	8	38874932	G	T	Missense_Mutation	149	0.268456376	No	1	Clonal	Disease_causing	Passenger	Neutral	passenger	No	No	No	No	Yes
MP27-1	Chondroid	ADORA2B	p.R215L	Private	17	15878301	G	T	Missense_Mutation	136	0.198529412	No	0.709	Subclonal	Disease_causing	Passenger	Neutral	passenger	Yes	No	No	No	Not tested
MP27-3	Ductal	ALCAM	NA	Private	3	105290809	G	C	Splice_Site	174	0.229885057	No	1	Clonal	.	.	.	passenger	No	No	No	No	Not tested
MP27-3	Ductal	ALDH4A1	p.V329M	Private	1	19204062	C	T	Missense_Mutation	95	0.052631579	No	0.15	Subclonal	Disease_causing	Passenger	Neutral	passenger	Yes	No	No	No	Not tested
MP27-1	Chondroid	AMICA1	p.X304_splice	Private	11	118068807	C	T	Splice_Site	241	0.066390041	No	0.303	Subclonal	Disease_causing	.	.	likely_pathogenic	No	No	No	No	Yes
MP27-3	Ductal	ANAPC4	NA	Shared	4	25378967	G	C	Splice_Site	181	0.044198895	No	0.17	Subclonal	.	.	.	passenger	No	No	No	No	Not tested
MP27-1	Chondroid	ANAPC4	NA	Shared	4	25378967	G	C	Splice_Site	226	0.123893805	No	0.566	Subclonal	.	.	.	passenger	No	No	No	No	Not tested
MP27-3	Ductal	AOC1	p.R428W	Private	7	150554840	C	T	Missense_Mutation	136	0.051470588	No	0.147	Subclonal	Disease_causing	Passenger	Neutral	passenger	Yes	No	No	No	Yes
MP27-1	Chondroid	AP2M1	p.I222V	Private	3	183898971	A	G	Missense_Mutation	380	0.110526316	No	0.726	Subclonal	Disease_causing	Passenger	Neutral	passenger	Yes	No	No	No	Not tested
MP27-3	Ductal	ARHGAP21	p.Q763E	Shared	10	24908537	G	C	Missense_Mutation	216	0.060185185	No	0.232	Subclonal	Neutral	Passenger	Neutral	passenger	No	No	No	No	Not tested
MP27-1	Chondroid	ARHGAP21	p.Q763E	Shared	10	24908537	G	C	Missense_Mutation	222	0.166666667	No	0.761	Subclonal	Neutral	Passenger	Neutral	passenger	No	No	No	No	Not tested
MP27-3	Ductal	ASXL1	p.N57S	Private	20	30956844	A	G	Missense_Mutation	166	0.114457831	No	0.441	Subclonal	Disease_causing	Passenger	Neutral	passenger	No	Yes	Yes	Yes	Yes
MP27-1	Chondroid	ATF6B	p.N175H	Private	6	32089081	T	G	Missense_Mutation	141	0.04964539	No	0.227	Subclonal	Neutral	Passenger	Neutral	passenger	No	No	No	No	Yes
MP27-3	Ductal	BMP4	p.S133I	Private	14	54417579	C	A	Missense_Mutation	186	0.064516129	No	0.184	Subclonal	Disease_causing	Passenger	Neutral	passenger	Yes	No	No	No	Yes
MP27-3	Ductal	C14orf164	p.K57N	Shared	14	23713548	G	C	Missense_Mutation	187	0.262032086	No	1	Clonal	Disease_causing	Passenger	.	passenger	No	No	No	No	Not tested
MP27-1	Chondroid	C14orf164	p.K57N	Shared	14	23713548	G	C	Missense_Mutation	110	0.281818182	No	1	Clonal	Disease_causing	Passenger	.	passenger	No	No	No	No	Not tested
MP27-1	Chondroid	CALB1	p.L218P	Private	8	91072876	A	G	Missense_Mutation	90	0.144444444	No	0.804	Subclonal	Disease_causing	Passenger	Disease_causing	likely_pathogenic	No	No	No	No	Not tested
MP27-3	Ductal	CAND2	p.E861V	Shared	3	12859013	A	T	Missense_Mutation	120	0.141666667	No	0.546	Subclonal	Disease_causing	Passenger	Neutral	passenger	Yes	No	No	No	Not tested
MP27-1	Chondroid	CAND2	p.E861V	Shared	3	12859013	A	T	Missense_Mutation	102	0.274509804	No	1	Subclonal	Disease_causing	Passenger	Neutral	passenger	Yes	No	No	No	Not tested
MP27-1	Chondroid	CCDC149	p.Q156*	Private	4	24839801	G	A	Nonsense_Mutation	101	0.158415842	No	0.724	Subclonal	Disease_causing	.	.	passenger	No	No	No	No	Not tested
MP27-1	Chondroid	CDHR2	p.N922S	Private	5	176012985	A	G	Missense_Mutation	195	0.041025641	No	0.146	Subclonal	Disease_causing	Passenger	Neutral	passenger	Yes	No	No	No	Yes
MP27-3	Ductal	CHD7	p.T335R	Private	8	61654995	C	G	Missense_Mutation	364	0.035714286	No	0.173	Subclonal	Disease_causing	Passenger	Neutral	passenger	No	No	No	No	Not tested
MP27-1	Chondroid	CHERP	p.W472C	Private	19	16636378	C	G	Missense_Mutation	58	0.275862069	No	0.984	Subclonal	Disease_causing	Passenger	Neutral	passenger	Yes	No	No	No	Yes
MP27-3	Ductal	CHI3L1	p.T82R	Shared	1	203154324	G	C	Missense_Mutation	87	0.413793103	No	1	Clonal	Neutral	Passenger	Neutral	passenger	No	No	No	No	Not tested
MP27-1	Chondroid	CHI3L1	p.T82R	Shared	1	203154324	G	C	Missense_Mutation	104	0.269230769	No	1	Clonal	Neutral	Passenger	Neutral	passenger	No	No	No	No	Not tested
MP27-3	Ductal	CNOT4	p.L240P	Shared	7	135095367	A	G	Missense_Mutation	199	0.351758794	No	1	Clonal	Disease_causing	Driver	Neutral	likely_pathogenic	No	No	No	No	Not tested
MP27-1	Chondroid	CNOT4	p.L240P	Shared	7	135095367	A	G	Missense_Mutation	143	0.167832168	No	0.767	Clonal	Disease_causing	Driver	Neutral	likely_pathogenic	No	No	No	No	Not tested
MP27-3	Ductal	COL6A2	p.I759F	Shared	21	47546004	A	T	Missense_Mutation	355	0.056338028	No	0.217	Subclonal	Disease_causing	Passenger	Disease_causing	likely_pathogenic	Yes	No	No	No	Not tested
MP27-1	Chondroid	COL6A2	p.I759F	Shared	21	47546004	A	T	Missense_Mutation	249	0.212851406	No	0.972	Subclonal	Disease_causing	Passenger	Disease_causing	likely_pathogenic	Yes	No	No	No	Not tested
MP27-1	Chondroid	CORO7	p.T207M	Private	16	4438628	G	A	Missense_Mutation	170	0.047058824	No	0.215	Subclonal	Neutral	Passenger	Neutral	passenger	No	No	No	No	Not tested
MP27-3	Ductal	CP	p.S474R	Private	3	148917580	T	G	Missense_Mutation	235	0.280851064	No	1	Clonal	Disease_causing	Passenger	Disease_causing	likely_pathogenic	Yes	No	No	No	Yes
MP27-3	Ductal	CPSF2	p.E475A	Shared	14	92621649	A	C	Missense_Mutation	93	0.688172043	No	1	Clonal	Disease_causing	Passenger	Neutral	passenger	Yes	No	No	No	Yes
MP27-1	Chondroid	CPSF2	p.E475A	Shared	14	92621649	A	C	Missense_Mutation	86	0.476744186	No	1	Clonal	Disease_causing	Passenger	Neutral	passenger	Yes	No	No	No	Yes
MP27-3	Ductal	CSMD2	p.R2988K	Shared	1	34006792	C	T	Missense_Mutation	187	0.385026738	No	1	Clonal	Disease_causing	Passenger	Neutral	passenger	No	No	No	No	Yes
MP27-1	Chondroid	CSMD2	p.R2988K	Shared	1	34006792	C	T	Missense_Mutation	91	0.373626374	No	1	Clonal	Disease_causing	Passenger	Neutral	passenger	No	No	No	No	Yes
MP27-1	Chondroid	CSRP2BP	p.E467K	Private	20	18143317	G	A	Missense_Mutation	239	0.033472803	No	0.153	Subclonal	Disease_causing	Passenger	Neutral	passenger	No	No	No	No	Not tested
MP27-1	Chondroid	DNAH14	p.A3107P	Private	1	225512026	G	C	Missense_Mutation	157	0.165605096	No	0.757	Subclonal	Disease_causing	Passenger	Neutral	passenger	No	No	No	No	Not tested
MP27-3	Ductal	DNAH3	p.A3421P	Shared	16	20974945	C	G	Missense_Mutation	347	0.057636888	No	0.28	Subclonal	Disease_causing	Passenger	Neutral	passenger	No	No	No	No	Not tested
MP27-1	Chondroid	DNAH3	p.A3421P	Shared	16	20974945	C	G	Missense_Mutation	273	0.183150183	No	1	Subclonal	Disease_causing	Passenger	Neutral	passenger	No	No	No	No	Not tested
MP27-3	Ductal	DNAH6	p.V1752I	Shared	2	84880618	G	A	Missense_Mutation	179	0.497206704	No	1	Clonal	Disease_causing	Passenger	Neutral	passenger	Yes	No	No	No	Yes
MP27-1	Chondroid	DNAH6	p.V1752I	Shared	2	84880618	G	A	Missense_Mutation	156	0.314102564	No	1	Clonal	Disease_causing	Passenger	Neutral	passenger	Yes	No	No	No	Yes
MP27-3	Ductal	DOCK8	p.N642S	Shared	9	371484	A	G	Missense_Mutation	152	0.085526316	No	0.329	Subclonal	Neutral	Passenger	Neutral	passenger	No	No	No	No	Not tested
MP27-1	Chondroid	DOCK8	p.N642S	Shared	9	371484	A	G	Missense_Mutation	111	0.216216216	No	0.988	Subclonal	Neutral	Passenger	Neutral	passenger	No	No	No	No	Not tested
MP27-3	Ductal	E4F1	p.H457Y	Shared	16	2283985	C	T	Missense_Mutation	149	0.093959732	No	0.362	Subclonal	Disease_causing	Passenger	Neutral	passenger	No	No	No	No	Not tested
MP27-1	Chondroid	E4F1	p.H457Y	Shared	16	2283985	C	T	Missense_Mutation	126	0.174603175	No	0.798	Subclonal	Disease_causing	Passenger	Neutral	passenger	No	No	No	No	Not tested
MP27-1	Chondroid	EBLN1	p.S36Tfs*17	Private	10	22498801	GTCTGC	G	Frame_Shift_Del	187	0.058823529	No	0.269	Subclonal	.	.	.	.	No	No	No	No	Not tested
MP27-3	Ductal	EEF1D	p.R276_R281del	Shared	8	144671407	TTGCGGCCCCGCCGGTCTC	T	In_Frame_Del	383	0.133159269	No	0.646	Subclonal	.	.	.	.	No	No	No	No	Not tested
MP27-1	Chondroid	EEF1D	p.R276_R281del	Shared	8	144671407	TTGCGGCCCCGCCGGTCTC	T	In_Frame_Del	290	0.103448276	No	0.576	Subclonal	.	.	.	.	No	No	No	No	Not tested
MP27-3	Ductal	EEF1G	p.G318C	Private	11	62327832	C	A	Missense_Mutation	113	0.283185841	No	1	Clonal	Disease_causing	Passenger	Neutral	passenger	No	No	No	No	Yes
MP27-3	Ductal	EFS	p.R505S	Private	14	23826606	C	G	Missense_Mutation	241	0.078838174	No	0.304	Subclonal	Neutral	Passenger	Neutral	passenger	No	No	No	No	Not tested
MP27-3	Ductal	EID1	p.A49P	Shared	15	49170518	G	C	Missense_Mutation	120	0.65	No	1	Clonal	Neutral	Passenger	Neutral	passenger	Yes	No	No	No	Not tested
MP27-1	Chondroid	EID1	p.A49P	Shared	15	49170518	G	C	Missense_Mutation	146	0.452054795	No	1	Clonal	Neutral	Passenger	Neutral	passenger	Yes	No	No	No	Not tested
MP27-1	Chondroid	EPS8	p.A263Qfs*11	Private	12	15814982	GC	G	Frame_Shift_Del	201	0.124378109	No	0.568	Subclonal	.	.	.	.	No	No	No	No	Yes
MP27-3	Ductal	ETV6	p.P25R	Shared	12	11905424	C	G	Missense_Mutation	184	0.358695652	No	1	Clonal	Neutral	Passenger	Neutral	passenger	No	Yes	No	No	Not tested
MP27-1	Chondroid	ETV6	p.P25R	Shared	12	11905424	C	G	Missense_Mutation	194	0.319587629	No	1	Clonal	Neutral	Passenger	Neutral	passenger	No	Yes	No	No	Not tested
MP27-3	Ductal	FAM13A	p.D391Y	Shared	4	89709004	C	A	Missense_Mutation	107	0.177570093	No	0.506	Subclonal	Disease_causing	Passenger	Neutral	passenger	Yes	No	No	No	Not tested
MP27-1	Chondroid	FAM13A	p.D391Y	Shared	4	89709004	C	A	Missense_Mutation	83	0.530120482	No	1	Subclonal	Disease_causing	Passenger	Neutral	passenger	Yes	No	No	No	Not tested
MP27-3	Ductal	FAM154A	p.S321P	Private	9	18928514	A	G	Missense_Mutation	110	0.336363636	No	1	Clonal	Neutral	Passenger	Neutral	passenger	No	No	No	No	Yes
MP27-3	Ductal	FAM47A	p.K663T	Shared	X	34148408	T	G	Missense_Mutation	232	0.362068966	No	1	Clonal	Neutral	Passenger	Neutral	passenger	No	No	No	No	Not tested
MP27-1	Chondroid	FAM47A	p.K663T	Shared	X	34148408	T	G	Missense_Mutation	167	0.395209581	No	1	Clonal	Neutral	Passenger	Neutral	passenger	No	No	No	No	Not tested
MP27-3	Ductal	FAM71E2	p.E45*	Private	19	55874302	C	A	Nonsense_Mutation	341	0.53372434	No	1	Clonal	Neutral	.	.	likely_pathogenic	Yes	No	No	No	Yes
MP27-1	Chondroid	FKBP11	p.X106_splice	Private	12	49317636	C	T	Splice_Site	140	0.071428571	No	0.255	Subclonal	Disease_causing	.	.	likely_pathogenic	No	No	No	No	Not tested
MP27-3	Ductal	GABRP	p.I355del	Shared	5	170238998	TATC	T	In_Frame_Del	143	0.615384615	No	1	Clonal	.	.	.	.	Yes	No	No	No	Not tested
MP27-1	Chondroid	GABRP	p.I355del	Shared	5	170238998	TATC	T	In_Frame_Del	123	0.504065041	No	1	Clonal	.	.	.	.	Yes	No	No	No	Not tested
MP27-3	Ductal	GDF9	p.Q33*	Private	5	132200129	G	A	Nonsense_Mutation	229	0.056768559	No	0.162	Subclonal	Neutral	.	.	likely_pathogenic	Yes	No	No	No	Not tested
MP27-3	Ductal	GPKOW	p.R25L	Shared	X	48979999	C	A	Missense_Mutation	417	0.388489209	No	1	Clonal	Disease_causing	Passenger	.	passenger	No	No	No	No	Yes
MP27-1	Chondroid	GPKOW	p.R25L	Shared	X	48979999	C	A	Missense_Mutation	418	0.332535885	No	1	Clonal	Disease_causing	Passenger	.	passenger	No	No	No	No	Yes
MP27-3	Ductal	GPR98	p.K5789E	Private	5	90149261	A	G	Missense_Mutation	91	0.076923077	No	0.219	Subclonal	Neutral	Passenger	Neutral	passenger	Yes	No	No	No	Yes
MP27-3	Ductal	GRIN3A	p.I587V	Private	9	104432935	T	C	Missense_Mutation	138	0.050724638	No	0.145	Subclonal	Neutral	Passenger	Neutral	passenger	Yes	No	No	No	Yes
MP27-3	Ductal	HSDL2	p.I72V	Shared	9	115167936	A	G	Missense_Mutation	204	0.691176471	No	1	Clonal	Disease_causing	Passenger	Disease_causing	likely_pathogenic	Yes	No	No	No	Yes
MP27-1	Chondroid	HSDL2	p.I72V	Shared	9	115167936	A	G	Missense_Mutation	188	0.670212766	No	1	Clonal	Disease_causing	Passenger	Disease_causing	likely_pathogenic	Yes	No	No	No	Yes
MP27-3	Ductal	IFFO1	p.K90E	Private	12	6664928	T	C	Missense_Mutation	543	0.215469613	No	1	Clonal	Disease_causing	Passenger	Disease_causing	likely_pathogenic	No	No	No	No	Yes
MP27-1	Chondroid	IGFN1	p.G895V	Private	1	201176705	G	T	Missense_Mutation	84	0.095238095	No	0.435	Subclonal	Neutral	Passenger	Neutral	passenger	No	No	No	No	Not tested
MP27-3	Ductal	IKBKB	p.X613_splice	Private	8	42179864	G	C	Splice_Site	256	0.0390625	No	0.15	Subclonal	Disease_causing	.	.	likely_pathogenic	No	Yes	No	No	Yes
MP27-1	Chondroid	JADE2	p.M182I	Private	5	133896509	G	A	Missense_Mutation	97	0.06185567	No	0.221	Subclonal	Disease_causing	Passenger	Neutral	passenger	Yes	No	No	No	Yes
MP27-3	Ductal	KIF7	p.P543R	Shared	15	90190221	G	C	Missense_Mutation	241	0.871369295	No	1	Clonal	Disease_causing	Passenger	Neutral	passenger	Yes	No	No	No	Yes
MP27-1	Chondroid	KIF7	p.P543R	Shared	15	90190221	G	C	Missense_Mutation	171	0.725146199	No	1	Clonal	Disease_causing	Passenger	Neutral	passenger	Yes	No	No	No	Yes
MP27-1	Chondroid	KMT2C	p.D4530H	Private	7	151845424	C	G	Missense_Mutation	251	0.03187251	No	0.114	Subclonal	Disease_causing	Passenger	Disease_causing	likely_pathogenic	Yes	Yes	Yes	Yes	Not tested
MP27-3	Ductal	KPNA6	p.E350D	Shared	1	32631765	G	T	Missense_Mutation	287	0.358885017	No	1	Clonal	Disease_causing	Passenger	Neutral	passenger	No	No	No	No	Not tested
MP27-1	Chondroid	KPNA6	p.E350D	Shared	1	32631765	G	T	Missense_Mutation	248	0.241935484	No	1	Clonal	Disease_causing	Passenger	Neutral	passenger	No	No	No	No	Not tested
MP27-3	Ductal	LOXL4	p.E663Q	Shared	10	100011424	C	G	Missense_Mutation	88	0.659090909	No	1	Clonal	Disease_causing	Passenger	Neutral	passenger	Yes	No	No	No	Yes
MP27-1	Chondroid	LOXL4	p.E663Q	Shared	10	100011424	C	G	Missense_Mutation	82	0.585365854	No	1	Clonal	Disease_causing	Passenger	Neutral	passenger	Yes	No	No	No	Yes
MP27-3	Ductal	LRRIQ1	p.W752*	Private	12	85450826	G	A	Nonsense_Mutation	168	0.279761905	No	1	Clonal	Neutral	.	.	likely_pathogenic	Yes	No	No	No	Yes
MP27-3	Ductal	LYZL2	p.S19Y	Shared	10	30918579	G	T	Missense_Mutation	143	0.482517483	No	1	Clonal	Neutral	Passenger	Neutral	passenger	No	No	No	No	Not tested
MP27-1	Chondroid	LYZL2	p.S19Y	Shared	10	30918579	G	T	Missense_Mutation	130	0.415384615	No	1	Clonal	Neutral	Passenger	Neutral	passenger	No	No	No	No	Not tested
MP27-1	Chondroid	MAPK14	p.V319L	Private	6	36075345	G	C	Missense_Mutation	332	0.138554217	No	0.633	Subclonal	Disease_causing	Passenger	Neutral	passenger	No	No	No	No	Not tested
MP27-1	Chondroid	MRPL38	p.R58L	Private	17	73900695	C	A	Missense_Mutation	231	0.116883117	No	0.534	Subclonal	Disease_causing	Passenger	Neutral	passenger	Yes	No	No	No	Not tested
MP27-1	Chondroid	NBL1	p.M49K	Private	1	19981564	T	A	Missense_Mutation	80	0.2625	No	0.937	Subclonal	Neutral	Passenger	Neutral	passenger	Yes	No	No	No	Not tested
MP27-3	Ductal	NID2	p.D1044N	Private	14	52481892	C	T	Missense_Mutation	150	0.04	No	0.114	Subclonal	Disease_causing	Passenger	Neutral	passenger	Yes	No	No	No	Yes
MP27-3	Ductal	NNT	p.N3I	Shared	5	43609305	A	T	Missense_Mutation	176	0.375	No	1	Clonal	Disease_causing	Passenger	Disease_causing	likely_pathogenic	No	No	No	No	Yes
MP27-1	Chondroid	NNT	p.N3I	Shared	5	43609305	A	T	Missense_Mutation	140	0.321428571	No	1	Clonal	Disease_causing	Passenger	Disease_causing	likely_pathogenic	No	No	No	No	Yes
MP27-1	Chondroid	NOTCH1	p.R1683W	Private	9	139397754	G	A	Missense_Mutation	98	0.051020408	No	0.284	Subclonal	Disease_causing	Passenger	Neutral	passenger	No	Yes	Yes	Yes	Not tested
MP27-3	Ductal	OR5AR1	p.F104Lfs*2	Shared	11	56431466	CT	C	Frame_Shift_Del	246	0.321138211	No	1	Clonal	.	.	.	.	No	No	No	No	Not tested
MP27-1	Chondroid	OR5AR1	p.F104Lfs*2	Shared	11	56431466	CT	C	Frame_Shift_Del	177	0.305084746	No	1	Clonal	.	.	.	.	No	No	No	No	Not tested
MP27-3	Ductal	ORC2	p.R482Q	Private	2	201784966	C	T	Missense_Mutation	137	0.04379562	No	0.212	Subclonal	Disease_causing	Passenger	Neutral	passenger	Yes	No	No	No	Yes
MP27-3	Ductal	OXR1	p.S270L	Shared	8	107715264	C	T	Missense_Mutation	164	0.329268293	No	1	Clonal	Disease_causing	Passenger	Neutral	passenger	No	No	No	No	Not tested
MP27-1	Chondroid	OXR1	p.S270L	Shared	8	107715264	C	T	Missense_Mutation	139	0.194244604	No	1	Clonal	Disease_causing	Passenger	Neutral	passenger	No	No	No	No	Not tested
MP27-1	Chondroid	PALM	p.R33L	Private	19	727048	G	T	Missense_Mutation	59	0.169491525	No	0.605	Subclonal	Disease_causing	Passenger	Neutral	passenger	Yes	No	No	No	Not tested
MP27-3	Ductal	PCDH18	p.S782F	Shared	4	138450898	G	A	Missense_Mutation	220	0.168181818	No	0.479	Subclonal	Disease_causing	Passenger	Neutral	passenger	No	No	No	No	Not tested
MP27-1	Chondroid	PCDH18	p.S782F	Shared	4	138450898	G	A	Missense_Mutation	207	0.45410628	No	1	Subclonal	Disease_causing	Passenger	Neutral	passenger	Yes	No	No	No	Not tested
MP27-3	Ductal	PHLDB1	p.R288C	Private	11	118498401	C	T	Missense_Mutation	98	0.051020408	No	0.145	Subclonal	Disease_causing	Passenger	Neutral	passenger	Yes	No	No	No	Not tested
MP27-1	Chondroid	PHLDB3	p.R545C	Private	19	43983598	G	A	Missense_Mutation	237	0.156118143	No	0.713	Subclonal	Disease_causing	Passenger	Neutral	passenger	Yes	No	No	No	Not tested
MP27-3	Ductal	PKDREJ	p.A237E	Shared	22	46658510	G	T	Missense_Mutation	521	0.591170825	No	1	Clonal	Neutral	Passenger	Neutral	passenger	No	No	No	No	Not tested
MP27-1	Chondroid	PKDREJ	p.A237E	Shared	22	46658510	G	T	Missense_Mutation	307	0.299674267	No	1	Clonal	Neutral	Passenger	Neutral	passenger	No	No	No	No	Not tested
MP27-3	Ductal	PLSCR4	p.V81Afs*39	Private	3	145924396	GCATAGGATATTTGCCAGGCTGATACCGGA	G	Frame_Shift_Del	165	0.090909091	No	0.441	Subclonal	.	.	.	.	No	No	No	No	Not tested
MP27-3	Ductal	PPIG	p.H133N	Private	2	170470993	C	A	Missense_Mutation	347	0.236311239	No	1	Clonal	Disease_causing	Passenger	Neutral	passenger	Yes	No	No	No	Yes
MP27-3	Ductal	PPM1F	p.K8N	Shared	22	22300397	C	A	Missense_Mutation	141	0.397163121	No	1	Clonal	Neutral	Passenger	Neutral	passenger	No	No	No	No	Not tested
MP27-1	Chondroid	PPM1F	p.K8N	Shared	22	22300397	C	A	Missense_Mutation	123	0.268292683	No	1	Clonal	Neutral	Passenger	Neutral	passenger	No	No	No	No	Not tested
MP27-1	Chondroid	PROZ	p.L119V	Private	13	113817352	C	G	Missense_Mutation	316	0.060126582	No	0.335	Subclonal	Neutral	Passenger	Disease_causing	passenger	Yes	No	No	No	Not tested
MP27-1	Chondroid	PRRC2B	p.Q2043H	Private	9	134363387	G	T	Missense_Mutation	163	0.110429448	No	0.505	Subclonal	Disease_causing	Passenger	Neutral	passenger	Yes	No	No	No	Yes
MP27-1	Chondroid	PRRT4	p.S851Y	Private	7	127991058	G	T	Missense_Mutation	82	0.12195122	No	0.557	Subclonal	Neutral	Passenger	.	passenger	No	No	No	No	Not tested
MP27-3	Ductal	PTAFR	p.L203V	Private	1	28476926	G	C	Missense_Mutation	285	0.031578947	No	0.122	Subclonal	Neutral	Passenger	Neutral	passenger	No	No	No	No	Yes
MP27-3	Ductal	RASAL3	p.E924K	Private	19	15563527	C	T	Missense_Mutation	118	0.43220339	No	1	Clonal	Neutral	Passenger	Disease_causing	passenger	Yes	No	No	No	Yes
MP27-3	Ductal	RBM5	p.R764Q	Private	3	50154781	G	A	Missense_Mutation	81	0.061728395	No	0.176	Subclonal	Disease_causing	Passenger	Neutral	passenger	Yes	No	No	No	Not tested
MP27-3	Ductal	RCOR1	p.M164I	Private	14	103167661	G	A	Missense_Mutation	71	0.577464789	No	1	Clonal	Disease_causing	Passenger	.	passenger	Yes	No	No	No	Yes
MP27-1	Chondroid	RNF20	p.R142H	Private	9	104302899	G	A	Missense_Mutation	241	0.033195021	No	0.152	Subclonal	Disease_causing	Passenger	Neutral	passenger	No	No	No	No	Not tested
MP27-3	Ductal	RP9	p.R110C	Shared	7	33136960	G	A	Missense_Mutation	85	0.447058824	No	1	Clonal	Disease_causing	Passenger	Disease_causing	likely_pathogenic	No	No	No	No	Yes
MP27-1	Chondroid	RP9	p.R110C	Shared	7	33136960	G	A	Missense_Mutation	72	0.347222222	No	1	Clonal	Disease_causing	Passenger	Disease_causing	likely_pathogenic	No	No	No	No	Yes
MP27-1	Chondroid	SAC3D1	p.P211L	Private	11	64811754	C	T	Missense_Mutation	271	0.062730627	No	0.287	Subclonal	Disease_causing	Passenger	Neutral	passenger	No	No	No	No	Not tested
MP27-3	Ductal	SAP30BP	p.K202N	Shared	17	73700840	G	C	Missense_Mutation	218	0.357798165	No	1	Clonal	Disease_causing	Passenger	.	passenger	Yes	No	No	No	Not tested
MP27-1	Chondroid	SAP30BP	p.K202N	Shared	17	73700840	G	C	Missense_Mutation	218	0.357798165	No	1	Clonal	Disease_causing	Passenger	.	passenger	Yes	No	No	No	Not tested
MP27-3	Ductal	SCARF2	p.R390C	Shared	22	20784750	G	A	Missense_Mutation	106	0.386792453	No	1	Clonal	Disease_causing	Passenger	Neutral	passenger	No	No	No	No	Yes
MP27-1	Chondroid	SCARF2	p.R390C	Shared	22	20784750	G	A	Missense_Mutation	96	0.3125	No	1	Clonal	Disease_causing	Passenger	Neutral	passenger	No	No	No	No	Yes
MP27-3	Ductal	SCN2A	p.G1937A	Private	2	166246126	G	C	Missense_Mutation	176	0.102272727	No	0.496	Subclonal	Neutral	Passenger	Disease_causing	passenger	Yes	No	No	No	Not tested
MP27-1	Chondroid	SECISBP2L	p.S439T	Private	15	49309149	A	T	Missense_Mutation	158	0.044303797	No	0.158	Subclonal	Disease_causing	Passenger	Disease_causing	likely_pathogenic	Yes	No	No	No	Not tested
MP27-3	Ductal	SIPA1L2	p.K524Q	Shared	1	232629320	T	G	Missense_Mutation	208	0.466346154	No	1	Clonal	Disease_causing	Passenger	Neutral	passenger	No	No	No	No	Not tested
MP27-1	Chondroid	SIPA1L2	p.K524Q	Shared	1	232629320	T	G	Missense_Mutation	142	0.345070423	No	1	Clonal	Disease_causing	Passenger	Neutral	passenger	No	No	No	No	Not tested
MP27-3	Ductal	SLC11A1	p.E79D	Private	2	219249052	G	T	Missense_Mutation	263	0.239543726	No	1	Clonal	Disease_causing	Passenger	Neutral	passenger	Yes	No	No	No	Yes
MP27-3	Ductal	SLC52A3	p.V415M	Private	20	741837	C	T	Missense_Mutation	130	0.253846154	No	0.977	Clonal	Disease_causing	Passenger	Neutral	passenger	No	No	No	No	Not tested
MP27-3	Ductal	SLIT3	p.P1269A	Private	5	168100218	G	C	Missense_Mutation	112	0.044642857	No	0.127	Subclonal	Disease_causing	Passenger	Neutral	passenger	Yes	No	No	No	Yes
MP27-3	Ductal	SLITRK4	p.V724M	Private	X	142716755	C	T	Missense_Mutation	308	0.146103896	No	0.709	Subclonal	Neutral	Passenger	Neutral	passenger	No	No	No	No	Yes
MP27-3	Ductal	SLX4	p.A1358G	Shared	16	3639566	G	C	Missense_Mutation	114	0.061403509	No	0.236	Subclonal	Neutral	Passenger	Neutral	passenger	No	No	No	No	Not tested
MP27-1	Chondroid	SLX4	p.A1358G	Shared	16	3639566	G	C	Missense_Mutation	106	0.179245283	No	0.819	Subclonal	Neutral	Passenger	Neutral	passenger	No	No	No	No	Not tested
MP27-3	Ductal	SST	p.A9P	Private	3	187388055	C	G	Missense_Mutation	171	0.467836257	No	1	Clonal	Disease_causing	Passenger	Neutral	passenger	Yes	No	No	No	Yes
MP27-3	Ductal	SUOX	p.G54Sfs*6	Shared	12	56396433	CAGGGATGGAG	C	Frame_Shift_Del	146	0.438356164	No	1	Clonal	.	.	.	.	Yes	No	No	No	Not tested
MP27-1	Chondroid	SUOX	p.G54Sfs*6	Shared	12	56396433	CAGGGATGGAG	C	Frame_Shift_Del	152	0.322368421	No	1	Clonal	.	.	.	.	Yes	No	No	No	Not tested
MP27-1	Chondroid	SV2A	p.G254C	Private	1	149883395	C	A	Missense_Mutation	267	0.037453184	No	0.209	Subclonal	Disease_causing	Passenger	Neutral	passenger	No	No	No	No	Yes
MP27-3	Ductal	TAF1L	p.R823*	Shared	9	32633111	G	A	Nonsense_Mutation	234	0.337606838	No	1	Clonal	Disease_causing	.	.	passenger	No	No	No	No	Not tested
MP27-1	Chondroid	TAF1L	p.R823*	Shared	9	32633111	G	A	Nonsense_Mutation	220	0.395454545	No	1	Clonal	Disease_causing	.	.	passenger	No	No	No	No	Not tested
MP27-1	Chondroid	TAF1L	p.S576C	Private	9	32633851	G	C	Missense_Mutation	376	0.130319149	No	0.595	Subclonal	Disease_causing	Passenger	Neutral	passenger	No	No	No	No	Not tested
MP27-3	Ductal	TANGO2	p.R67G	Shared	22	20040041	C	G	Missense_Mutation	101	0.435643564	No	1	Clonal	Neutral	Passenger	Neutral	passenger	No	No	No	No	Not tested
MP27-1	Chondroid	TANGO2	p.R67G	Shared	22	20040041	C	G	Missense_Mutation	87	0.310344828	No	1	Clonal	Neutral	Passenger	Neutral	passenger	No	No	No	No	Not tested
MP27-1	Chondroid	TCF4	p.V697M	Private	18	52896174	C	T	Missense_Mutation	264	0.034090909	No	0.122	Subclonal	Disease_causing	Passenger	Disease_causing	likely_pathogenic	Yes	No	No	No	Not tested
MP27-3	Ductal	TJP2	p.K869*	Shared	9	71855009	A	T	Nonsense_Mutation	202	0.366336634	No	1	Clonal	Neutral	.	.	passenger	No	No	No	No	Not tested
MP27-1	Chondroid	TJP2	p.K869*	Shared	9	71855009	A	T	Nonsense_Mutation	183	0.349726776	No	1	Clonal	Neutral	.	.	passenger	No	No	No	No	Not tested
MP27-3	Ductal	TP53	p.C176F	Shared	17	7578403	C	A	Missense_Mutation	185	0.627027027	Yes	1	Clonal	Disease_causing	Passenger	Disease_causing	likely_pathogenic	Yes	Yes	Yes	Yes	Yes
MP27-1	Chondroid	TP53	p.C176F	Shared	17	7578403	C	A	Missense_Mutation	189	0.423280423	Yes	1	Clonal	Disease_causing	Passenger	Disease_causing	likely_pathogenic	Yes	Yes	Yes	Yes	Yes
MP27-3	Ductal	TTC28	p.V1638I	Private	22	28394735	C	T	Missense_Mutation	343	0.206997085	No	0.797	Subclonal	Disease_causing	Passenger	Disease_causing	likely_pathogenic	No	No	No	No	Yes
MP27-3	Ductal	UBQLN4	p.R407H	Private	1	156012611	C	T	Missense_Mutation	82	0.219512195	No	1	Clonal	Disease_causing	Passenger	Neutral	passenger	No	No	No	No	Yes
MP27-3	Ductal	VPS52	p.R239*	Private	6	33235970	G	A	Nonsense_Mutation	73	0.068493151	No	0.264	Subclonal	Neutral	.	.	passenger	No	No	No	No	Yes
MP27-1	Chondroid	VPS53	p.P797L	Private	17	422477	G	A	Missense_Mutation	89	0.056179775	No	0.2	Subclonal	Disease_causing	Passenger	Neutral	passenger	Yes	No	No	No	Yes
MP27-1	Chondroid	XPO5	p.V19F	Private	6	43543547	C	A	Missense_Mutation	479	0.03131524	No	0.143	Subclonal	Disease_causing	Passenger	Neutral	passenger	No	No	No	No	Yes
MP27-3	Ductal	ZFHX4	p.R2282G	Private	8	77766001	C	G	Missense_Mutation	214	0.037383178	No	0.144	Subclonal	Disease_causing	Driver	Disease_causing	likely_pathogenic	No	No	No	No	Yes
MP27-3	Ductal	ZNF586	p.R310Q	Private	19	58290884	G	A	Missense_Mutation	210	0.047619048	No	0.183	Subclonal	Neutral	Passenger	Neutral	passenger	Yes	No	No	No	Not tested
MP35-1	Chondroid	ARHGEF12	p.R923C	Private	11	120337930	C	T	Missense_Mutation	97	0.051546392	No	0.16	Subclonal	Disease_causing	Passenger	Neutral	passenger	Yes	Yes	No	No	Yes
MP35-1	Chondroid	ARSB	p.T204K	Shared	5	78260318	G	T	Missense_Mutation	80	0.525	No	1	Clonal	Neutral	Passenger	Disease_causing	passenger	Yes	No	No	No	Not tested
MP35-2	Ductal	ARSB	p.T204K	Shared	5	78260318	G	T	Missense_Mutation	76	0.486842105	No	1	Clonal	Neutral	Passenger	Disease_causing	passenger	Yes	No	No	No	Not tested
MP35-2	Ductal	ATXN1	p.E725K	Private	6	16306835	C	T	Missense_Mutation	261	0.210727969	No	0.866	Subclonal	Disease_causing	Passenger	Neutral	passenger	No	No	No	No	Not tested
MP35-1	Chondroid	BRD4	p.A3S	Private	19	15383904	C	A	Missense_Mutation	275	0.065454545	No	0.334	Subclonal	Neutral	Passenger	Neutral	passenger	No	Yes	No	No	Yes
MP35-1	Chondroid	C8orf44	p.M42T	Shared	8	67590068	T	C	Missense_Mutation	138	0.086956522	No	0.444	Subclonal	Neutral	Passenger	Neutral	passenger	No	No	No	No	Not tested
MP35-2	Ductal	C8orf44	p.M42T	Shared	8	67590068	T	C	Missense_Mutation	133	0.045112782	No	0.23	Subclonal	Neutral	Passenger	Neutral	passenger	No	No	No	No	Not tested
MP35-1	Chondroid	CAMK2B	p.R312Q	Private	7	44280317	C	T	Missense_Mutation	122	0.040983607	No	0.168	Subclonal	Disease_causing	Passenger	Neutral	passenger	No	No	No	No	Yes
MP35-1	Chondroid	CCDC173	p.L65*	Private	2	170537617	A	T	Nonsense_Mutation	138	0.108695652	No	0.555	Subclonal	Neutral	.	.	passenger	Yes	No	No	No	Yes
MP35-1	Chondroid	CHRM2	p.K264R	Shared	7	136700403	A	G	Missense_Mutation	102	0.401960784	No	1	Clonal	Disease_causing	Passenger	Neutral	passenger	Yes	No	No	No	Not tested
MP35-2	Ductal	CHRM2	p.K264R	Shared	7	136700403	A	G	Missense_Mutation	164	0.31097561	No	1	Clonal	Disease_causing	Passenger	Neutral	passenger	No	No	No	No	Not tested
MP35-1	Chondroid	CLEC11A	p.G35V	Private	19	51226886	G	T	Missense_Mutation	126	0.222222222	No	1	Clonal	Disease_causing	Passenger	Neutral	passenger	No	No	No	No	Yes
MP35-1	Chondroid	CUL9	p.R321Q	Private	6	43153904	G	A	Missense_Mutation	233	0.030042918	No	0.123	Subclonal	Neutral	Passenger	Neutral	passenger	No	No	No	No	Yes
MP35-1	Chondroid	DEPDC5	p.D46N	Private	22	32154609	G	A	Missense_Mutation	120	0.066666667	No	0.274	Subclonal	Disease_causing	Passenger	Neutral	passenger	No	No	No	No	Not tested
MP35-2	Ductal	DNAH11	p.F2992Y	Private	7	21805059	T	A	Missense_Mutation	141	0.127659574	No	0.397	Subclonal	Disease_causing	Passenger	Neutral	passenger	Yes	No	No	No	Yes
MP35-1	Chondroid	DNAH8	p.R526P	Private	6	38749118	G	C	Missense_Mutation	186	0.048387097	No	0.199	Subclonal	Disease_causing	Passenger	Neutral	passenger	No	No	No	No	Yes
MP35-1	Chondroid	EHF	p.V150F	Shared	11	34670604	G	T	Missense_Mutation	260	0.238461538	No	1	Clonal	Disease_causing	Passenger	Neutral	passenger	No	No	No	No	Yes
MP35-2	Ductal	EHF	p.V150F	Shared	11	34670604	G	T	Missense_Mutation	208	0.245192308	No	1	Clonal	Disease_causing	Passenger	Neutral	passenger	No	No	No	No	Yes
MP35-1	Chondroid	EPHA3	p.W345*	Shared	3	89390969	G	A	Nonsense_Mutation	144	0.3125	No	1	Clonal	Neutral	.	.	likely_pathogenic	Yes	No	Yes	No	Not tested
MP35-2	Ductal	EPHA3	p.W345*	Shared	3	89390969	G	A	Nonsense_Mutation	158	0.265822785	No	1	Clonal	Neutral	.	.	likely_pathogenic	Yes	No	Yes	No	Not tested
MP35-2	Ductal	EXTL3	p.T664M	Private	8	28575567	C	T	Missense_Mutation	142	0.049295775	No	0.153	Subclonal	Disease_causing	Passenger	Disease_causing	likely_pathogenic	Yes	No	No	No	Yes
MP35-2	Ductal	FGF11	p.P87H	Private	17	7344856	C	A	Missense_Mutation	64	0.3125	No	0.972	Subclonal	Disease_causing	Passenger	Disease_causing	likely_pathogenic	Yes	No	No	No	Yes
MP35-1	Chondroid	FGFBP2	p.S149P	Shared	4	15964308	A	G	Missense_Mutation	94	0.436170213	No	1	Clonal	Neutral	Passenger	Neutral	passenger	Yes	No	No	No	Not tested
MP35-2	Ductal	FGFBP2	p.S149P	Shared	4	15964308	A	G	Missense_Mutation	117	0.52991453	No	1	Clonal	Neutral	Passenger	Neutral	passenger	Yes	No	No	No	Not tested
MP35-1	Chondroid	GCGR	p.A77D	Private	17	79768767	C	A	Missense_Mutation	162	0.216049383	No	1	Clonal	Disease_causing	Passenger	Neutral	passenger	Yes	No	No	No	Yes
MP35-1	Chondroid	GLI2	p.R1470W	Private	2	121747898	C	T	Missense_Mutation	171	0.046783626	No	0.239	Subclonal	Neutral	Passenger	Neutral	passenger	Yes	No	No	No	Yes
MP35-1	Chondroid	GPATCH11	p.R59H	Private	2	37316911	G	A	Missense_Mutation	98	0.071428571	No	0.294	Subclonal	Disease_causing	Passenger	Neutral	passenger	Yes	No	No	No	Yes
MP35-2	Ductal	GPR142	p.C153*	Private	17	72366760	T	A	Nonsense_Mutation	177	0.271186441	No	1	Subclonal	Neutral	.	.	likely_pathogenic	No	No	No	No	Not tested
MP35-1	Chondroid	GPR65	p.G34V	Shared	14	88477292	G	T	Missense_Mutation	181	0.386740331	No	1	Clonal	Disease_causing	Passenger	Neutral	passenger	Yes	No	No	No	Yes
MP35-2	Ductal	GPR65	p.G34V	Shared	14	88477292	G	T	Missense_Mutation	160	0.33125	No	1	Clonal	Disease_causing	Passenger	Neutral	passenger	Yes	No	No	No	Yes
MP35-1	Chondroid	ID1	p.G119E	Shared	20	30193546	G	A	Missense_Mutation	231	0.333333333	No	1	Clonal	Disease_causing	Passenger	Neutral	passenger	No	No	No	No	Yes
MP35-2	Ductal	ID1	p.G119E	Shared	20	30193546	G	A	Missense_Mutation	224	0.388392857	No	1	Clonal	Disease_causing	Passenger	Neutral	passenger	No	No	No	No	Yes
MP35-1	Chondroid	IKBKB	p.L123F	Shared	8	42151009	C	T	Missense_Mutation	251	0.362549801	No	1	Clonal	Disease_causing	Driver	Neutral	likely_pathogenic	No	Yes	No	No	Yes
MP35-2	Ductal	IKBKB	p.L123F	Shared	8	42151009	C	T	Missense_Mutation	203	0.399014778	No	1	Clonal	Disease_causing	Driver	Neutral	likely_pathogenic	No	Yes	No	No	Yes
MP35-1	Chondroid	ILVBL	p.L405S	Shared	19	15227306	A	G	Missense_Mutation	74	0.243243243	No	1	Clonal	Disease_causing	Passenger	Neutral	passenger	No	No	No	No	Not tested
MP35-2	Ductal	ILVBL	p.L405S	Shared	19	15227306	A	G	Missense_Mutation	88	0.193181818	No	0.794	Clonal	Disease_causing	Passenger	Neutral	passenger	No	No	No	No	Not tested
MP35-1	Chondroid	INTS3	p.V79L	Shared	1	153713761	G	T	Missense_Mutation	261	0.333333333	No	1	Clonal	Disease_causing	Passenger	.	passenger	No	No	No	No	Not tested
MP35-2	Ductal	INTS3	p.V79L	Shared	1	153713761	G	T	Missense_Mutation	268	0.347014925	No	1	Clonal	Disease_causing	Passenger	.	passenger	No	No	No	No	Not tested
MP35-1	Chondroid	JADE1	p.P396L	Shared	4	129783064	C	T	Missense_Mutation	133	0.278195489	No	1	Clonal	Neutral	Passenger	Neutral	passenger	No	No	No	No	Not tested
MP35-2	Ductal	JADE1	p.P396L	Shared	4	129783064	C	T	Missense_Mutation	111	0.378378378	No	1	Clonal	Neutral	Passenger	Neutral	passenger	No	No	No	No	Not tested
MP35-1	Chondroid	KIAA1217	p.E286A	Shared	10	24762167	A	C	Missense_Mutation	200	0.285	No	0.886	Clonal	Disease_causing	Passenger	Neutral	passenger	Yes	No	No	No	Yes
MP35-2	Ductal	KIAA1217	p.E286A	Shared	10	24762167	A	C	Missense_Mutation	231	0.372294372	No	1	Clonal	Disease_causing	Passenger	Neutral	passenger	Yes	No	No	No	Yes
MP35-2	Ductal	MARCKS	p.E190K	Private	6	114181324	G	A	Missense_Mutation	54	0.092592593	No	0.473	Subclonal	Disease_causing	Passenger	Neutral	passenger	No	No	No	No	Yes
MP35-1	Chondroid	MDH1	p.R175G	Shared	2	63826397	C	G	Missense_Mutation	105	0.371428571	No	1	Clonal	Disease_causing	Passenger	Neutral	passenger	Yes	No	No	No	Yes
MP35-2	Ductal	MDH1	p.R175G	Shared	2	63826397	C	G	Missense_Mutation	93	0.322580645	No	1	Clonal	Disease_causing	Passenger	Neutral	passenger	Yes	No	No	No	Yes
MP35-1	Chondroid	MUS81	p.H68Q	Shared	11	65628512	C	A	Missense_Mutation	230	0.386956522	No	1	Clonal	Disease_causing	Passenger	Neutral	passenger	Yes	No	No	No	Not tested
MP35-2	Ductal	MUS81	p.H68Q	Shared	11	65628512	C	A	Missense_Mutation	194	0.355670103	No	1	Clonal	Disease_causing	Passenger	Neutral	passenger	Yes	No	No	No	Not tested
MP35-1	Chondroid	NAV2	p.S1821N	Shared	11	20101724	G	A	Missense_Mutation	108	0.592592593	No	1	Clonal	Disease_causing	Passenger	Disease_causing	likely_pathogenic	Yes	No	No	No	Yes
MP35-2	Ductal	NAV2	p.S1821N	Shared	11	20101724	G	A	Missense_Mutation	99	0.636363636	No	1	Clonal	Disease_causing	Passenger	Disease_causing	likely_pathogenic	Yes	No	No	No	Yes
MP35-2	Ductal	NBEA	p.S1572L	Private	13	35756549	C	T	Missense_Mutation	139	0.043165468	No	0.134	Subclonal	Disease_causing	Passenger	Neutral	passenger	Yes	No	No	No	Yes
MP35-1	Chondroid	NFE2	p.E171K	Private	12	54686769	C	T	Missense_Mutation	232	0.034482759	No	0.142	Subclonal	Disease_causing	Passenger	.	passenger	No	No	No	No	Not tested
MP35-2	Ductal	NLRC3	p.T909S	Private	16	3599158	T	A	Missense_Mutation	114	0.105263158	No	0.433	Subclonal	Neutral	Passenger	Neutral	passenger	No	No	No	No	Yes
MP35-2	Ductal	NLRP14	p.K24E	Private	11	7059887	A	G	Missense_Mutation	68	0.147058824	No	0.457	Subclonal	Neutral	Passenger	Neutral	passenger	Yes	No	No	No	Yes
MP35-1	Chondroid	NOL12	p.R37Q	Private	22	38083943	G	A	Missense_Mutation	77	0.064935065	No	0.267	Subclonal	Disease_causing	Passenger	Disease_causing	likely_pathogenic	No	No	No	No	Yes
MP35-1	Chondroid	OR14A16	p.A36D	Shared	1	247978925	G	T	Missense_Mutation	68	0.338235294	No	1	Clonal	Neutral	Passenger	Neutral	passenger	No	No	No	No	Not tested
MP35-2	Ductal	OR14A16	p.A36D	Shared	1	247978925	G	T	Missense_Mutation	81	0.234567901	No	1	Clonal	Neutral	Passenger	Neutral	passenger	No	No	No	No	Not tested
MP35-1	Chondroid	OR2B2	p.I256T	Shared	6	27879331	A	G	Missense_Mutation	197	0.284263959	No	1	Clonal	Neutral	Passenger	Neutral	passenger	No	No	No	No	Not tested
MP35-2	Ductal	OR2B2	p.I256T	Shared	6	27879331	A	G	Missense_Mutation	193	0.352331606	No	1	Clonal	Neutral	Passenger	Neutral	passenger	No	No	No	No	Not tested
MP35-1	Chondroid	OR2G2	p.S107C	Shared	1	247751981	C	G	Missense_Mutation	243	0.382716049	No	1	Clonal	Neutral	Passenger	Neutral	passenger	No	No	No	No	Not tested
MP35-2	Ductal	OR2G2	p.S107C	Shared	1	247751981	C	G	Missense_Mutation	235	0.336170213	No	1	Clonal	Neutral	Passenger	Neutral	passenger	No	No	No	No	Not tested
MP35-1	Chondroid	OR6S1	p.V15I	Shared	14	21109808	C	T	Missense_Mutation	218	0.362385321	No	1	Clonal	Neutral	Passenger	Neutral	passenger	No	No	No	No	Not tested
MP35-2	Ductal	OR6S1	p.V15I	Shared	14	21109808	C	T	Missense_Mutation	192	0.34375	No	1	Clonal	Neutral	Passenger	Neutral	passenger	No	No	No	No	Not tested
MP35-1	Chondroid	PCDH8	p.V636Pfs*73	Shared	13	53420660	CCTGCACA	C	Frame_Shift_Del	81	0.543209877	No	1	Clonal	.	.	.	.	Yes	No	No	No	Not tested
MP35-2	Ductal	PCDH8	p.V636Pfs*73	Shared	13	53420660	CCTGCACA	C	Frame_Shift_Del	94	0.361702128	No	1	Clonal	.	.	.	.	Yes	No	No	No	Not tested
MP35-1	Chondroid	PLXNA2	p.E716Gfs*7	Shared	1	208257876	TCC	T	Frame_Shift_Del	281	0.277580071	No	1	Clonal	.	.	.	.	No	No	No	No	Not tested
MP35-2	Ductal	PLXNA2	p.E716Gfs*7	Shared	1	208257876	TCC	T	Frame_Shift_Del	275	0.243636364	No	1	Clonal	.	.	.	.	No	No	No	No	Not tested
MP35-1	Chondroid	PPP6R3	p.W496*	Shared	11	68343453	G	A	Nonsense_Mutation	90	0.4	No	1	Clonal	Neutral	.	.	likely_pathogenic	Yes	No	No	No	Not tested
MP35-2	Ductal	PPP6R3	p.W496*	Shared	11	68343453	G	A	Nonsense_Mutation	87	0.229885057	No	0.945	Clonal	Neutral	.	.	likely_pathogenic	Yes	No	No	No	Not tested

MP35-1	Chondroid	PRRC2A	p.V1462I	Shared	6	31601220	G	A	Missense_Mutation	[illegible]	[illegible]	No	1	Clonal	Disease_causing	Passenger	Neutral	passenger	No	No	No	No	Not tested
MP35-2	Ductal	PRRC2A	p.R4G	Private	6	31590576	C	G	Missense_Mutation	149	[illegible]	No	1	Subclonal	Disease_causing	Passenger	Neutral	passenger	No	No	No	No	Not tested
MP35-2	Ductal	PRRC2A	p.V1462I	Shared	6	31601220	G	A	Missense_Mutation	140	0.378571429	No	1	Clonal	Disease_causing	Passenger	Neutral	passenger	No	No	No	No	Not tested
MP35-2	Ductal	RASGEF1A	p.R229S	Private	10	43695699	C	A	Missense_Mutation	75	0.4	No	1	Subclonal	Disease_causing	Passenger	Neutral	passenger	Yes	No	No	No	Yes
MP35-1	Chondroid	RBL1	p.S615C	Shared	20	35668615	G	C	Missense_Mutation	203	0.39408867	No	1	Clonal	Disease_causing	Passenger	Disease_causing	likely_pathogenic	No	No	No	No	Not tested
MP35-2	Ductal	RBL1	p.S615C	Shared	20	35668615	G	C	Missense_Mutation	188	0.292553191	No	1	Clonal	Disease_causing	Passenger	Disease_causing	likely_pathogenic	No	No	No	No	Not tested
MP35-1	Chondroid	RHBDL1	p.F93S	Shared	16	726379	T	C	Missense_Mutation	298	0.348993289	No	1	Clonal	Disease_causing	Passenger	Neutral	passenger	No	No	No	No	Yes
MP35-2	Ductal	RHBDL1	p.F93S	Shared	16	726379	T	C	Missense_Mutation	251	0.338645418	No	1	Clonal	Disease_causing	Passenger	Neutral	passenger	No	No	No	No	Yes
MP35-1	Chondroid	SERAC1	p.G254V	Shared	6	158551496	C	A	Missense_Mutation	237	0.291139241	No	1	Clonal	Disease_causing	Passenger	Neutral	passenger	No	No	No	No	Not tested
MP35-2	Ductal	SERAC1	p.G254V	Shared	6	158551496	C	A	Missense_Mutation	249	0.309236948	No	1	Clonal	Disease_causing	Passenger	Neutral	passenger	No	No	No	No	Not tested
MP35-1	Chondroid	SLC4A1AP	p.G40W	Shared	2	27886737	G	T	Missense_Mutation	247	0.344129555	No	1	Clonal	Neutral	Passenger	Neutral	passenger	Yes	No	No	No	Not tested
MP35-2	Ductal	SLC4A1AP	p.G40W	Shared	2	27886737	G	T	Missense_Mutation	266	0.458646617	No	1	Clonal	Neutral	Passenger	Neutral	passenger	Yes	No	No	No	Not tested
MP35-1	Chondroid	SMC4	p.R705P	Shared	3	160141307	G	C	Missense_Mutation	103	0.233009709	No	1	Clonal	Disease_causing	Passenger	Disease_causing	likely_pathogenic	Yes	No	No	No	Not tested
MP35-2	Ductal	SMC4	p.R705P	Shared	3	160141307	G	C	Missense_Mutation	105	0.219047619	No	1	Clonal	Disease_causing	Passenger	Disease_causing	likely_pathogenic	Yes	No	No	No	Not tested
MP35-1	Chondroid	SRGAP1	p.A656D	Shared	12	64505589	C	A	Missense_Mutation	215	0.36744186	No	1	Clonal	Disease_causing	Passenger	Neutral	passenger	No	No	No	No	Yes
MP35-2	Ductal	SRGAP1	p.A656D	Shared	12	64505589	C	A	Missense_Mutation	173	0.404624277	No	1	Clonal	Disease_causing	Passenger	Neutral	passenger	No	No	No	No	Yes
MP35-2	Ductal	SSC5D	p.L455Tfs*26	Private	19	56006125	CCTTTGGGAGCCTGG	C	Frame_Shift_Del	110	0.218181818	No	0.897	Subclonal	.	.	.	.	No	No	No	No	Yes
MP35-2	Ductal	STAB2	p.G2118R	Private	12	104142848	G	A	Missense_Mutation	97	0.051546392	No	0.16	Subclonal	Disease_causing	Passenger	Disease_causing	likely_pathogenic	Yes	No	No	No	Not tested
MP35-1	Chondroid	TCF25	p.P473Rfs*12	Private	16	89970559	GGCCCGACGCCA	G	Frame_Shift_Del	303	0.062706271	No	0.258	Subclonal	.	.	.	.	Yes	No	No	No	Yes
MP35-2	Ductal	TMED8	p.R194Q	Private	14	77809700	C	T	Missense_Mutation	185	0.037837838	No	0.118	Subclonal	Disease_causing	Passenger	Neutral	passenger	Yes	No	No	No	Yes
MP35-1	Chondroid	TMEM131	p.P209T	Shared	2	98458376	G	T	Missense_Mutation	279	0.164874552	No	1	Clonal	Disease_causing	Passenger	Neutral	passenger	Yes	No	No	No	Not tested
MP35-2	Ductal	TMEM131	p.P209T	Shared	2	98458376	G	T	Missense_Mutation	314	0.203821656	No	1	Clonal	Disease_causing	Passenger	Neutral	passenger	Yes	No	No	No	Not tested
MP35-2	Ductal	TMEM132B	p.S988N	Private	12	126138982	G	A	Missense_Mutation	64	0.078125	No	0.243	Subclonal	Disease_causing	Passenger	Neutral	passenger	Yes	No	No	No	Yes
MP35-1	Chondroid	TP53	p.C238Y	Shared	17	7577568	C	T	Missense_Mutation	120	0.616666667	Yes	1	Clonal	Disease_causing	Passenger	Disease_causing	likely_pathogenic	No	Yes	Yes	Yes	Yes
MP35-2	Ductal	TP53	p.C238Y	Shared	17	7577568	C	T	Missense_Mutation	102	0.549019608	Yes	1	Clonal	Disease_causing	Passenger	Disease_causing	likely_pathogenic	No	Yes	Yes	Yes	Yes
MP35-1	Chondroid	TTYH2	p.M322I	Shared	17	72246110	G	A	Missense_Mutation	702	0.300569801	No	1	Clonal	Disease_causing	Passenger	Neutral	passenger	Yes	No	No	No	Not tested
MP35-2	Ductal	TTYH2	p.M322I	Shared	17	72246110	G	A	Missense_Mutation	543	0.309392265	No	1	Clonal	Disease_causing	Passenger	Neutral	passenger	Yes	No	No	No	Not tested
MP35-2	Ductal	UGGT2	p.D1152H	Private	13	96529552	C	G	Missense_Mutation	185	0.32972973	No	1	Subclonal	Disease_causing	Passenger	Neutral	passenger	Yes	No	No	No	Yes
MP35-2	Ductal	URB2	p.S14G	Private	1	229763420	A	G	Missense_Mutation	199	0.331658291	No	1	Subclonal	Disease_causing	Passenger	Neutral	passenger	No	No	No	No	Not tested
MP35-2	Ductal	USP19	p.R620W	Private	3	49152901	G	A	Missense_Mutation	133	0.045112782	No	0.185	Subclonal	Disease_causing	Passenger	Neutral	passenger	Yes	No	No	No	Yes
MP35-2	Ductal	WNT3	p.A121V	Private	17	44847375	G	A	Missense_Mutation	137	0.03649635	No	0.186	Subclonal	Disease_causing	Passenger	Disease_causing	likely_pathogenic	Yes	No	No	No	Yes
MP35-1	Chondroid	ZFHX2	p.A430P	Shared	14	24003247	C	G	Missense_Mutation	69	0.260869565	No	1	Clonal	Neutral	Passenger	Neutral	passenger	No	No	No	No	Not tested
MP35-2	Ductal	ZFHX2	p.A430P	Shared	14	24003247	C	G	Missense_Mutation	82	0.341463415	No	1	Clonal	Neutral	Passenger	Neutral	passenger	No	No	No	No	Not tested

UTERINE CARCINOSARCOMAS - MULTIPLE COMPONENTS

Sample ID	Histologic component	Gene symbol	Amino acid change	Shared/Private Mutations	Chromosome	Genomic position	Reference allele	Alternate allele	Type of mutation	Depth at mutation (x)	Mutant allele fraction	HOTSPOT	Cancer Cell Fraction (Pyclone)	Clonal Status	Mutation Taster	CHASM	FATHMM	Pathogenecity	Loss of heterozygosity (LOH)	Cancer Gene Census	Kandoth	Cancer5000-S genes (Lawrence et al)	Validated by AmpliSeq
CS1-S	Sarcoma	SEPT5	p.E339Rfs*31	Private	X	118767397	C	CT	Frame_Shift_Ins	423	0.122931442	No	0.433	Subclonal	.	.	.	.	No	No	No	No	Not tested
CS1-C	Carcinoma	A1BG	p.R244W	Private	19	58862937	G	A	Missense_Mutation	292	0.123287671	No	1	Clonal	Neutral	Passenger	Neutral	passenger	No	No	No	No	Not tested
CS1-C	Carcinoma	AACS	p.A143T	Private	12	125570944	G	A	Missense_Mutation	394	0.053299492	No	0.51	Subclonal	Disease_causing	Passenger	Neutral	passenger	No	No	No	No	Not tested
CS1-S	Sarcoma	AAED1	p.I94M	Private	9	99413974	T	C	Missense_Mutation	281	0.24911032	No	0.877	Subclonal	Disease_causing	Passenger	Neutral	passenger	No	No	No	No	Not tested
CS1-C	Carcinoma	AAK1	p.V162I	Private	2	69769705	C	T	Missense_Mutation	191	0.183246073	No	1	Clonal	Disease_causing	Passenger	Neutral	passenger	No	No	No	No	Not tested
CS1-C	Carcinoma	AAMP	p.P214H	Private	2	219131204	G	T	Missense_Mutation	159	0.062893082	No	0.601	Subclonal	Disease_causing	Passenger	Neutral	passenger	No	No	No	No	Not tested
CS1-S	Sarcoma	AARS	p.R198W	Private	16	70305763	G	A	Missense_Mutation	128	0.390625	No	1	Clonal	Disease_causing	Passenger	Neutral	passenger	No	No	No	No	Not tested
CS1-S	Sarcoma	AARS	p.A734T	Private	16	70289717	C	T	Missense_Mutation	392	0.262755102	No	0.925	Clonal	Disease_causing	Passenger	Neutral	passenger	No	No	No	No	Not tested
CS1-S	Sarcoma	AARS2	p.V380del	Private	6	44274667	TCCA	T	In_Frame_Del	274	0.350364964	No	1	Clonal	.	.	.	.	No	No	No	No	Not tested
CS1-S	Sarcoma	AASDH	p.Q945Tfs*54	Private	4	57209076	T	TG	Frame_Shift_Ins	338	0.281065089	No	0.989	Clonal	.	.	.	.	No	No	No	No	Not tested
CS1-C	Carcinoma	ABCA2	p.H1659Y	Private	9	139907270	G	A	Missense_Mutation	88	0.056818182	No	0.543	Subclonal	Disease_causing	Passenger	Disease_causing	likely_pathogenic	No	No	No	No	Not tested
CS1-C	Carcinoma	ABCB4	p.R1116G	Private	7	87035765	T	C	Missense_Mutation	241	0.041493776	No	0.397	Subclonal	Disease_causing	Passenger	Disease_causing	likely_pathogenic	No	No	No	No	Not tested
CS1-C	Carcinoma	ABCC1	p.N898S	Private	16	16196532	A	G	Missense_Mutation	236	0.233050847	No	1	Clonal	Disease_causing	Passenger	Disease_causing	likely_pathogenic	No	No	No	No	Not tested
CS1-C	Carcinoma	ABCC12	p.L1135F	Private	16	48122528	G	A	Missense_Mutation	329	0.185410334	No	1	Clonal	Disease_causing	Passenger	Disease_causing	likely_pathogenic	No	No	No	No	Not tested
CS1-C	Carcinoma	ABCC9	p.R824*	Private	12	22012555	G	A	Nonsense_Mutation	378	0.034391534	No	0.329	Subclonal	Neutral	.	.	passenger	No	No	No	No	Not tested
CS1-S	Sarcoma	ABCF2	p.R113C	Private	7	150921892	G	A	Missense_Mutation	343	0.134110787	No	0.472	Subclonal	Disease_causing	Passenger	Neutral	passenger	No	No	No	No	Not tested
CS1-C	Carcinoma	ABHD10	p.D268V	Private	3	111710450	A	T	Missense_Mutation	439	0.079726651	No	0.762	Clonal	Disease_causing	Passenger	Neutral	passenger	No	No	No	No	Not tested
CS1-C	Carcinoma	ABHD6	p.N333del	Shared	3	58279469	GACA	G	In_Frame_Del	200	0.31	No	1	Clonal	.	.	.	.	No	No	No	No	Not tested
CS1-S	Sarcoma	ABHD6	p.N333del	Shared	3	58279469	GACA	G	In_Frame_Del	300	0.406666667	No	1	Clonal	.	.	.	.	No	No	No	No	Not tested
CS1-C	Carcinoma	ABLIM2	p.D120Tfs*3	Private	4	8089995	CG	C	Frame_Shift_Del	244	0.155737705	No	1	Clonal	.	.	.	.	No	No	No	No	Not tested
CS1-S	Sarcoma	ACACB	p.E1972D	Private	12	109690834	G	T	Missense_Mutation	152	0.453947368	No	1	Clonal	Disease_causing	Passenger	Disease_causing	likely_pathogenic	No	No	No	No	Not tested
CS1-S	Sarcoma	ACACB	p.L1398Sfs*26	Private	12	109671599	AC	A	Frame_Shift_Del	475	0.210526316	No	0.741	Subclonal	.	.	.	.	No	No	No	No	Not tested
CS1-S	Sarcoma	ACLY	p.M474I	Private	17	40054009	C	T	Missense_Mutation	86	0.5	No	1	Clonal	Neutral	Passenger	Neutral	passenger	No	No	No	No	Not tested
CS1-S	Sarcoma	ACPT	p.Q117R	Private	19	51294959	A	G	Missense_Mutation	168	0.035714286	No	0.126	Subclonal	Disease_causing	Passenger	Neutral	passenger	No	No	No	No	Not tested
CS1-C	Carcinoma	ACSL5	p.P69Q	Private	10	114154742	C	A	Missense_Mutation	264	0.071969697	No	0.688	Subclonal	Disease_causing	Passenger	Neutral	passenger	No	No	No	No	Not tested
CS1-C	Carcinoma	ADAM28	p.N75Kfs*15	Private	8	24167472	G	GA	Frame_Shift_Ins	257	0.105058366	No	1	Clonal	.	.	.	.	No	No	No	No	Not tested
CS1-S	Sarcoma	ADAMTS18	p.E677G	Private	16	77359765	T	C	Missense_Mutation	278	0.143884892	No	0.506	Subclonal	Disease_causing	Passenger	Neutral	passenger	No	No	No	No	Not tested
CS1-S	Sarcoma	ADAMTSL4	p.Q643R	Private	1	150529692	A	G	Missense_Mutation	393	0.264631043	No	1	Clonal	Disease_causing	Passenger	Neutral	passenger	No	No	No	No	Not tested
CS1-C	Carcinoma	ADCY10	p.E7del	Private	1	167874357	ATTC	A	In_Frame_Del	308	0.12987013	No	1	Clonal	.	.	.	.	No	No	No	No	Not tested
CS1-S	Sarcoma	ADCY10	p.P1022Lfs*9	Private	1	167806500	GA	G	Frame_Shift_Del	525	0.283809524	No	1	Clonal	.	.	.	.	No	No	No	No	Not tested
CS1-S	Sarcoma	ADCY5	p.L1151P	Private	3	123008677	A	G	Missense_Mutation	242	0.05785124	No	0.204	Subclonal	Disease_causing	Passenger	Neutral	passenger	No	No	No	No	Not tested
CS1-C	Carcinoma	ADCY6	p.R668H	Private	12	49168815	C	T	Missense_Mutation	106	0.132075472	No	1	Clonal	Disease_causing	Passenger	Neutral	passenger	No	No	No	No	Not tested
CS1-C	Carcinoma	ADCY7	p.N346D	Private	16	50332902	A	G	Missense_Mutation	228	0.057017544	No	0.545	Subclonal	Disease_causing	Passenger	Disease_causing	likely_pathogenic	No	No	No	No	Not tested
CS1-C	Carcinoma	ADCY8	p.R179H	Private	8	132052044	C	T	Missense_Mutation	397	0.214105793	No	1	Clonal	Disease_causing	Passenger	Neutral	passenger	No	No	No	No	Not tested
CS1-C	Carcinoma	ADCY8	p.R32Q	Private	8	132052485	C	T	Missense_Mutation	158	0.037974684	No	0.363	Subclonal	Disease_causing	Passenger	Neutral	passenger	No	No	No	No	Not tested
CS1-C	Carcinoma	ADD3	p.K701Rfs*8	Shared	10	111893349	CA	C	Frame_Shift_Del	228	0.188596491	No	1	Clonal	.	.	.	.	No	No	No	No	Not tested
CS1-S	Sarcoma	ADD3	p.K701Rfs*8	Shared	10	111893349	CA	C	Frame_Shift_Del	289	0.522491349	No	1	Clonal	.	.	.	.	No	No	No	No	Not tested
CS1-C	Carcinoma	ADGB	p.T1547I	Private	6	147122969	C	T	Missense_Mutation	106	0.283018868	No	1	Clonal	Neutral	Passenger	Neutral	passenger	No	No	No	No	Not tested
CS1-S	Sarcoma	ADNP2	p.G512C	Private	18	77894830	G	T	Missense_Mutation	358	0.270949721	No	0.953	Clonal	Disease_causing	Passenger	.	passenger	No	No	No	No	Not tested
CS1-C	Carcinoma	ADO	p.L54P	Private	10	64564980	T	C	Missense_Mutation	397	0.04534005	No	0.433	Subclonal	Disease_causing	Passenger	Neutral	passenger	No	No	No	No	Not tested
CS1-C	Carcinoma	AFTPH	p.G720S	Private	2	64796296	G	A	Missense_Mutation	263	0.060836502	No	0.642	Subclonal	Disease_causing	Passenger	Neutral	passenger	No	No	No	No	Not tested
CS1-C	Carcinoma	AGBL2	p.K337E	Private	11	47712250	T	C	Missense_Mutation	336	0.232142857	No	1	Clonal	Disease_causing	Passenger	Neutral	passenger	No	No	No	No	Not tested
CS1-C	Carcinoma	AGRN	p.G1002Afs*38	Private	1	981860	GC	G	Frame_Shift_Del	276	0.192028986	No	1	Clonal	.	.	.	.	No	No	No	No	Not tested
CS1-C	Carcinoma	AHNAK	p.P883S	Private	11	62299242	G	A	Missense_Mutation	257	0.163424125	No	1	Clonal	Neutral	Passenger	Neutral	passenger	No	No	No	No	Not tested
CS1-C	Carcinoma	AHSG	p.A302T	Private	3	186338519	G	A	Missense_Mutation	375	0.08	No	0.765	Clonal	Neutral	Passenger	Neutral	passenger	No	No	No	No	Not tested
CS1-C	Carcinoma	AIRE	p.A482T	Private	21	45714327	G	A	Missense_Mutation	193	0.191709845	No	1	Clonal	Neutral	Passenger	Neutral	passenger	No	No	No	No	Not tested
CS1-C	Carcinoma	AK8	p.L474P	Private	9	135601094	A	G	Missense_Mutation	147	0.040816327	No	0.39	Subclonal	Disease_causing	Passenger	Neutral	passenger	No	No	No	No	Not tested
CS1-S	Sarcoma	AKAP6	p.T88I	Private	14	32902962	C	T	Missense_Mutation	251	0.09561753	No	0.336	Subclonal	Disease_causing	Passenger	Neutral	passenger	No	No	No	No	Not tested
CS1-S	Sarcoma	ALYREF	p.M14T	Private	17	79849416	A	G	Missense_Mutation	558	0.053763441	No	0.189	Subclonal	Disease_causing	Passenger	Neutral	passenger	No	No	No	No	Not tested
CS1-C	Carcinoma	AMER2	p.A153T	Private	13	25745301	C	T	Missense_Mutation	233	0.068669528	No	0.657	Subclonal	Neutral	Passenger	Neutral	passenger	No	No	No	No	Not tested
CS1-C	Carcinoma	AMER3	p.T517M	Private	2	131521195	C	T	Missense_Mutation	142	0.063380282	No	0.606	Subclonal	Neutral	Passenger	Neutral	passenger	No	No	No	No	Not tested
CS1-S	Sarcoma	AMHR2	p.W470R	Private	12	53824049	T	C	Missense_Mutation	92	0.489130435	No	1	Clonal	Disease_causing	Passenger	Disease_causing	likely_pathogenic	No	No	No	No	Not tested
CS1-C	Carcinoma	AMOTL2	p.G198Afs*21	Private	3	134089682	GC	G	Frame_Shift_Del	184	0.201086957	No	1	Clonal	.	.	.	.	No	No	No	No	Not tested
CS1-S	Sarcoma	AMPD1	p.M50I	Private	1	115231346	C	T	Missense_Mutation	546	0.256410256	No	0.902	Clonal	Neutral	Passenger	Neutral	passenger	No	No	No	No	Not tested
CS1-S	Sarcoma	AMPD2	p.R324H	Private	1	110169887	G	A	Missense_Mutation	132	0.325757576	No	1	Clonal	Neutral	Passenger	Disease_causing	passenger	No	No	No	No	Not tested
CS1-S	Sarcoma	ANAPC7	p.A143D	Private	12	110832988	G	T	Missense_Mutation	97	0.12371134	No	0.435	Subclonal	Disease_causing	Passenger	Neutral	passenger	No	No	No	No	Not tested
CS1-C	Carcinoma	ANGPT4	p.Q186R	Private	20	868991	T	C	Missense_Mutation	146	0.219178082	No	1	Clonal	Neutral	Passenger	Neutral	passenger	No	No	No	No	Not tested
CS1-C	Carcinoma	ANKRD12	p.S984Ifs*7	Private	18	9256206	G	GA	Frame_Shift_Ins	235	0.208510638	No	1	Clonal	.	.	.	.	No	No	No	No	Not tested
CS1-S	Sarcoma	ANKRD12	p.N935Ifs*24	Private	18	9256061	GA	G	Frame_Shift_Del	429	0.130536131	No	0.459	Subclonal	.	.	.	.	No	No	No	No	Not tested
CS1-C	Carcinoma	ANKRD17	p.A4T	Private	4	74124376	C	T	Missense_Mutation	157	0.21656051	No	1	Clonal	Neutral	Passenger	Neutral	passenger	No	No	No	No	Not tested
CS1-S	Sarcoma	ANKRD32	p.R735W	Private	5	94024292	C	T	Missense_Mutation	235	0.417021277	No	1	Clonal	Neutral	Passenger	Neutral	passenger	No	No	No	No	Not tested
CS1-C	Carcinoma	ANKRD53	p.X206_splice	Private	2	71209065	G	A	Splice_Site	83	0.240963855	No	1	Clonal	Disease_causing	.	.	passenger	No	No	No	No	Not tested
CS1-C	Carcinoma	ANKS1A	p.T312A	Private	6	34951124	A	G	Missense_Mutation	227	0.061674009	No	0.59	Subclonal	Disease_causing	Passenger	Neutral	passenger	No	No	No	No	Not tested
CS1-C	Carcinoma	ANKS1B	p.Q840*	Private	12	99548075	G	A	Nonsense_Mutation	287	0.038327526	No	0.366	Subclonal	Neutral	.	.	passenger	No	No	No	No	Not tested
CS1-C	Carcinoma	ANKS6	p.S152F	Private	9	101552793	G	A	Missense_Mutation	206	0.266990291	No	1	Clonal	Disease_causing	Passenger	Neutral	passenger	No	No	No	No	Not tested
CS1-C	Carcinoma	ANKZF1	p.A35V	Private	2	220095083	C	T	Missense_Mutation	270	0.248148148	No	1	Clonal	Neutral	Passenger	Neutral	passenger	No	No	No	No	Not tested
CS1-S	Sarcoma	ANLN	p.R1053H	Private	7	36489353	G	A	Missense_Mutation	378	0.402116402	No	1	Clonal	Disease_causing	Passenger	Neutral	passenger	No	No	No	No	Not tested
CS1-S	Sarcoma	ANO5	p.N64Ifs*27	Private	11	22242646	CA	C	Frame_Shift_Del	182	0.340659341	No	1	Clonal	.	.	.	.	No	No	No	No	Not tested
CS1-S	Sarcoma	ANXA2	p.G225*	Private	15	60644645	C	A	Nonsense_Mutation	115	0.373913043	No	1	Clonal	Neutral	.	.	passenger	No	No	No	No	Not tested
CS1-C	Carcinoma	AP1G2	p.L587P	Private	14	24030818	A	G	Missense_Mutation	166	0.15060241	No	1	Clonal	Disease_causing	Passenger	Neutral	passenger	No	No	No	No	Not tested
CS1-C	Carcinoma	AP1G2	p.A449Pfs*84	Private	14	24032653	GC	G	Frame_Shift_Del	158	0.14556962	No	1	Clonal	.	.	.	.	No	No	No	No	Not tested
CS1-C	Carcinoma	AP1S1	p.D122Mfs*11	Private	7	100802404	TG	T	Frame_Shift_Del	231	0.225108225	No	1	Clonal	.	.	.	.	No	No	No	No	Not tested
CS1-S	Sarcoma	AP1S1	p.D122Gfs*18	Private	7	100802404	T	TG	Frame_Shift_Ins	323	0.3374613	No	1	Clonal	.	.	.	.	No	No	No	No	Not tested
CS1-C	Carcinoma	AP3B1	p.L446S	Private	5	77458669	A	G	Missense_Mutation	255	0.08627451	No	0.825	Clonal	Disease_causing	Passenger	Neutral	passenger	No	No	No	No	Not tested
CS1-C	Carcinoma	AP3D1	p.K745Rfs*66	Private	19	2115332	CT	C	Frame_Shift_Del	324	0.212962963	No	1	Clonal	.	.	.	.	No	No	No	No	Not tested
CS1-S	Sarcoma	AP5Z1	p.V388I	Private	7	4825910	G	A	Missense_Mutation	318	0.13836478	No	0.487	Subclonal	Neutral	Passenger	Neutral	passenger	No	No	No	No	Not tested
CS1-C	Carcinoma	APBA2	p.S144L	Private	15	29346518	C	T	Missense_Mutation	218	0.032110092	No	0.307	Subclonal	Neutral	Passenger	Neutral	passenger	No	No	No	No	Not tested
CS1-C	Carcinoma	APBB1IP	p.Q91*	Private	10	26789858	C	T	Nonsense_Mutation	342	0.064327485	No	0.615	Subclonal	Disease_causing	.	.	likely_pathogenic	No	No	No	No	Not tested
CS1-S	Sarcoma	APCDD1L	p.A172V	Private	20	57042388	G	A	Missense_Mutation	153	0.098039216	No	0.345	Subclonal	Neutral	Passenger	Neutral	passenger	No	No	No	No	Not tested
CS1-S	Sarcoma	APLNR	p.A138T	Private	11	57004067	C	T	Missense_Mutation	279	0.111111111	No	0.391	Subclonal	Disease_causing	Passenger	Neutral	passenger	No	No	No	No	Not tested
CS1-C	Carcinoma	APOL2	p.L52F	Private	22	36624310	G	A	Missense_Mutation	391	0.056265985	No	0.538	Subclonal	Neutral	Passenger	Neutral	passenger	No	No	No	Yes	Not tested
CS1-S	Sarcoma	AQPEP	p.W913R	Private	5	115351443	T	A	Missense_Mutation	233	0.369098712	No	1	Clonal	Disease_causing	Passenger	Neutral	passenger	No	No	No	No	Not tested
CS1-S	Sarcoma	AR	p.A766T	Private	X	66937442	G	A	Missense_Mutation	279	0.415770609	No	1	Clonal	Disease_causing	Passenger	Disease_causing	likely_pathogenic	No	Yes	Yes	No	Not tested
CS1-C	Carcinoma	ARAF	p.R255Gfs*37	Private	X	47426414	AC	A	Frame_Shift_Del	229	0.183406114	No	1	Clonal	.	.	.	.	No	No	No	No	Not tested
CS1-C	Carcinoma	ARF3	p.L34P	Private	12	49334778	A	G	Missense_Mutation	164	0.201219512	No	1	Clonal	Disease_causing	Passenger	Neutral	passenger	No	No	No	No	Not tested
CS1-C	Carcinoma	ARFGAP2	p.F14*	Private	11	47198362	TAA	T	Frame_Shift_Del	273	0.212454212	No	1	Clonal	.	.	.	.	No	No	No	No	Not tested
CS1-C	Carcinoma	ARHGAP11A	p.F352L	Private	15	32921912	T	C	Missense_Mutation	158	0.075949367	No	0.726	Subclonal	Disease_causing	Passenger	Disease_causing	likely_pathogenic	No	No	No	No	Not tested
CS1-C	Carcinoma	ARHGAP15	p.C301Y	Private	2	144276910	G	A	Missense_Mutation	131	0.091603053	No	0.876	Clonal	Disease_causing	Passenger	Neutral	passenger	No	No	No	No	Not tested
CS1-S	Sarcoma	ARHGAP23	p.R377W	Private	17	36623053	C	T	Missense_Mutation	373	0.423592493	No	1	Clonal	Neutral	Passenger	Neutral	passenger	No	No	No	No	Not tested
CS1-S	Sarcoma	ARHGAP23	p.R91H	Private	17	36619105	G	A	Missense_Mutation	125	0.488	No	1	Clonal	Disease_causing	Passenger	Neutral	passenger	No	No	No	No	Not tested
CS1-C	Carcinoma	ARHGAP31	p.F525L	Private	3	119121174	T	A	Missense_Mutation	221	0.031674208	No	0.303	Subclonal	Neutral	Passenger	Neutral	passenger	No	No	No	No	Not tested
CS1-S	Sarcoma	ARHGAP35	p.K1148N	Private	19	47425376	G	T	Missense_Mutation	612	0.433006536	No	1	Clonal	Disease_causing	Passenger	Neutral	passenger	No	No	Yes	Yes	Not tested
CS1-C	Carcinoma	ARHGAP5	p.R1122*	Private	14	32563239	C	T	Nonsense_Mutation	255	0.082352941	No	0.787	Clonal	Neutral	.	.	passenger	No	No	No	No	Not tested
CS1-S	Sarcoma	ARHGEF10L	p.A216T	Private	1	17939589	G	A	Missense_Mutation	239	0.041841004	No	0.147	Subclonal	Disease_causing	Passenger	Neutral	passenger	No	No	No	No	Not tested
CS1-S	Sarcoma	ARHGEF12	p.F1382Lfs*35	Private	11	120351042	CT	C	Frame_Shift_Del	481	0.355509356	No	1	Clonal	.	.	.	.	No	Yes	No	No	Not tested
CS1-C	Carcinoma	ARID1A	p.Q566*	Shared	1	27057988	C	T	Nonsense_Mutation	196	0.346938776	No	1	Clonal	Disease_causing	.	.	likely_pathogenic	No	Yes	Yes	Yes	Not tested
CS1-S	Sarcoma	ARID1A	p.P1407Qfs*74	Private	1	27100933	GC	G	Frame_Shift_Del	308	0.13961039	No	0.491	Subclonal	.	.	.	.	No	Yes	Yes	Yes	Not tested
CS1-S	Sarcoma	ARID1A	p.P1898Hfs*25	Private	1	27106077	GC	G	Frame_Shift_Del	484	0.262396694	No	0.923	Clonal	.	.	.	.	No	Yes	Yes	Yes	Not tested
CS1-S	Sarcoma	ARID1A	p.Q566*	Shared	1	27057988	C	T	Nonsense_Mutation	283	0.431095406	No	1	Clonal	Disease_causing	.	.	likely_pathogenic	No	Yes	Yes	Yes	Not tested
CS1-S	Sarcoma	ARID1B	p.T695A	Private	6	157405841	A	G	Missense_Mutation	492	0.093495935	No	0.329	Subclonal	Neutral	Passenger	Neutral	passenger	No	Yes	No	No	Not tested
CS1-S	Sarcoma	ARID2	p.D196Ifs*19	Private	12	46211614	GA	G	Frame_Shift_Del	176	0.363636364	No	1	Clonal	.	.	.	.	No	Yes	No	Yes	Not tested
CS1-S	Sarcoma	ARL13B	p.N277Tfs*13	Private	3	93761882	GA	G	Frame_Shift_Del	211	0.402843602	No	1	Clonal	.	.	.	.	No	No	No	No	Not tested
CS1-S	Sarcoma	ARL3	p.V65A	Private	10	104459200	A	G	Missense_Mutation	256	0.4453125	No	1	Clonal	Disease_causing	Driver	Disease_causing	likely_pathogenic	No	No	No	No	Not tested
CS1-S	Sarcoma	ARMCX3	p.G50E	Private	X	100880118	G	A	Missense_Mutation	445	0.164044944	No	0.577	Subclonal	Disease_causing	Passenger	Neutral	passenger	No	No	No	No	Not tested
CS1-S	Sarcoma	ARMCX4	p.W1426C	Private	X	100747854	G	T	Missense_Mutation	298	0.426174497	No	1	Clonal	Neutral	Passenger	.	passenger	No	No	No	No	Not tested
CS1-S	Sarcoma	ARNT2	p.G284*	Private	15	80806719	G	T	Nonsense_Mutation	232	0.275862069	No	0.971	Clonal	Neutral	.	.	passenger	No	No	No	No	Not tested
CS1-C	Carcinoma	ARNTL	p.Y132H	Private	11	13381906	T	C	Missense_Mutation	314	0.219745223	No	1	Clonal	Disease_causing	Passenger	Neutral	passenger	No	No	No	No	Not tested
CS1-C	Carcinoma	ARPP21	p.G407D	Private	3	35770891	G	A	Missense_Mutation	210	0.061904762	No	0.592	Subclonal	Neutral	Passenger	Neutral	passenger	No	No	No	No	Not tested
CS1-C	Carcinoma	ARPP21	p.R476Q	Private	3	35778739	G	A	Missense_Mutation	270	0.040740741	No	0.39	Subclonal	Disease_causing	Passenger	Neutral	passenger	No	No	No	No	Not tested
CS1-C	Carcinoma	ARSH	p.T110M	Private	X	2931202	C	T	Missense_Mutation	120	0.175	No	1	Clonal	Disease_causing	Passenger	Disease_causing	likely_pathogenic	No	No	No	No	Not tested
CS1-C	Carcinoma	ASB13	p.A65V	Private	10	5694863	G	A	Missense_Mutation	80	0.075	No	0.717	Subclonal	Neutral	Passenger	Neutral	passenger	No	No	No	No	Not tested
CS1-C	Carcinoma	ASB8	p.K222E	Private	12	48543352	T	C	Missense_Mutation	448	0.073660714	No	0.704	Subclonal	Disease_causing	Passenger	Neutral	passenger	No	No	No	No	Not tested
CS1-C	Carcinoma	ASCC3	p.T2062I	Private	6	100960685	G	A	Missense_Mutation	195	0.148717949	No	1	Clonal	Disease_causing	Passenger	Neutral	passenger	No	No	No	No	Not tested
CS1-S	Sarcoma	ASCC3	p.F1364Sfs*18	Private	6	101086507	GA	G	Frame_Shift_Del	196	0.469387755	No	1	Clonal	.	.	.	.	No	No	No	No	Not tested
CS1-C	Carcinoma	ASPH	p.R636K	Private	8	62430676	C	T	Missense_Mutation	114	0.087719298	No	0.839	Clonal	Disease_causing	Passenger	Neutral	passenger	No	No	No	No	Not tested
CS1-C	Carcinoma	ASPN	p.I304Sfs*8	Private	9	95221948	AT	A	Frame_Shift_Del	97	0.257731959	No	1	Clonal	.	.	.	.	No	No	No	No	Not tested
CS1-C	Carcinoma	ASTN1	p.P64H	Private	1	177133622	G	T	Missense_Mutation	712	0.040730337	No	0.43	Subclonal	Disease_causing	Passenger	Neutral	passenger	No	No	No	No	Not tested
CS1-S	Sarcoma	ATAD2B	p.A663T	Private	2	24046272	C	T	Missense_Mutation	386	0.437823834	No	1	Clonal	Disease_causing	Passenger	Disease_causing	likely_pathogenic	No	No	No	No	Not tested
CS1-S	Sarcoma	ATE1	p.L167R	Private	10	123670504	A	C	Missense_Mutation	394	0.218274112	No	0.768	Subclonal	Neutral	Passenger	.	passenger	No	No	No	No	Not tested
CS1-C	Carcinoma	ATF7IP	p.X549_splice	Private	12	14587358	G	A	Splice_Site	179	0.061452514	No	0.588	Subclonal	Disease_causing	.	.	passenger	No	No	No	No	Not tested
CS1-C	Carcinoma	ATG2A	p.A1835T	Private	11	64662839	C	T	Missense_Mutation	113	0.212389381	No	1	Clonal	Neutral	Passenger	Neutral	passenger	No	No	No	No	Not tested
CS1-C	Carcinoma	ATG2A	p.M1557V	Private	11	64666110	T	C	Missense_Mutation	268	0.067164179	No	0.642	Subclonal	Disease_causing	Passenger	Neutral	passenger	No	No	No	No	Not tested
CS1-S	Sarcoma	ATG2A	p.R665W	Private	11	64677267	G	A	Missense_Mutation	267	0.033707865	No	0.119	Subclonal	Disease_causing	Passenger	Neutral	passenger	No	No	No	No	Not tested
CS1-S	Sarcoma	ATG9B	p.A71T	Private	7	150721300	C	T	Missense_Mutation	177	0.418079096	No	1	Clonal	.	Passenger	.	passenger	No	No	No	No	Not tested
CS1-C	Carcinoma	ATM	p.R2060Afs*22	Private	11	108186819	AC	A	Frame_Shift_Del	143	0.104895105	No	1	Clonal	.	.	.	.	No	Yes	Yes	Yes	Not tested
CS1-S	Sarcoma	ATP13A5	p.L461P	Private	3	193048991	A	G	Missense_Mutation	190	0.2	No	0.704	Subclonal	Disease_causing	Driver	Disease_causing	likely_pathogenic	No	No	No	No	Not tested
CS1-C	Carcinoma	ATP2A1	p.R822Pfs*39	Private	16	28913639	G	GC	Frame_Shift_Ins	325	0.166153846	No	1	Clonal	.	.	.	.	No	No	No	No	Not tested
CS1-C	Carcinoma	ATP2B3	p.A312Pfs*23	Private	X	152811559	TG	T	Frame_Shift_Del	303	0.191419142	No	1	Clonal	.	.	.	.	No	Yes	No	No	Not tested
CS1-S	Sarcoma	ATP6V0D1	p.F193del	Private	16	67473235	TAGA	T	In_Frame_Del	163	0.380368098	No	1	Clonal	.	.	.	.	No	No	No	No	Not tested
CS1-C	Carcinoma	ATP6V1B1	p.I183N	Private	2	71187171	T	A	Missense_Mutation	164	0.042682927	No	0.451	Subclonal	Disease_causing	Passenger	Neutral	passenger	No	No	No	No	Not tested
CS1-C	Carcinoma	ATP7B	p.D1222Tfs*108	Private	13	52513221	TC	T	Frame_Shift_Del	248	0.209677419	No	1	Clonal	.	.	.	.	No	No	No	No	Not tested
CS1-C	Carcinoma	ATP8B4	p.L535Wfs*13	Private	15	50223353	CA	C	Frame_Shift_Del	151	0.218543046	No	1	Clonal	.	.	.	.	No	No	No	No	Not tested
CS1-C	Carcinoma	ATP9A	p.T634M	Private	20	50241846	G	A	Missense_Mutation	294	0.18707483	No	1	Clonal	Disease_causing	Passenger	Neutral	passenger	No	No	No	No	Not tested
CS1-S	Sarcoma	ATRNL1	p.P110T	Private	10	116879983	C	A	Missense_Mutation	180	0.094444444	No	0.332	Subclonal	Disease_causing	Passenger	Neutral	passenger	No	No	No	No	Not tested
CS1-S	Sarcoma	ATXN2	p.A445V	Private	12	111957815	G	A	Missense_Mutation	342	0.269005848	No	0.947	Clonal	Disease_causing	Driver	Disease_causing	likely_pathogenic	No	No	No	No	Not tested
CS1-C	Carcinoma	AVPR2	p.V88M	Private	X	153171222	G	A	Missense_Mutation	331	0.202416918	No	1	Clonal	Disease_causing	Passenger	Neutral	passenger	No	No	No	No	Not tested
CS1-C	Carcinoma	AVPR2	p.A295T	Private	X	153171843	G	A	Missense_Mutation	191	0.246073298	No	1	Clonal	Disease_causing	Passenger	Neutral	passenger	No	No	No	No	Not tested
CS1-S	Sarcoma	B4GALT2	p.R327H	Private	1	44451218	G	A	Missense_Mutation	433	0.032332564	No	0.114	Subclonal	Disease_causing	Passenger	Disease_causing	likely_pathogenic	No	No	No	No	Not tested
CS1-C	Carcinoma	BACE2	p.P102Rfs*25	Private	21	42540488	AC	A	Frame_Shift_Del	188	0.228723404	No	1	Clonal	.	.	.	.	No	No	No	No	Not tested
CS1-S	Sarcoma	BAIAP2L1	p.V254Cfs*10	Private	7	97941456	CG	C	Frame_Shift_Del	160	0.24375	No	0.858	Subclonal	.	.	.	.	No	No	No	No	Not tested
CS1-S	Sarcoma	BAIAP3	p.R674H	Private	16	1395076	G	A	Missense_Mutation	361	0.423822715	No	1	Clonal	Disease_causing	Passenger	Neutral	passenger	No	No	No	No	Not tested
CS1-C	Carcinoma	BAZ2A	p.R1761C	Private	12	56993040	G	A	Missense_Mutation	294	0.030612245	No	0.293	Subclonal	Disease_causing	Driver	Neutral	likely_pathogenic	No	No	No	No	Not tested
CS1-S	Sarcoma	BCAN	p.R107C	Private	1	156616820	C	T	Missense_Mutation	462	0.207792208	No	0.939	Clonal	Disease_causing	Passenger	Neutral	passenger	No	No	No	No	Not tested
CS1-C	Carcinoma	BCL6	p.R270Q	Private	3	187447384	C	T	Missense_Mutation	325	0.046153846	No	0.441	Subclonal	Disease_causing	Passenger	Neutral	passenger	No	Yes	No	No	Not tested
CS1-C	Carcinoma	BCL9	p.P516Lfs*5	Shared	1	147091500	AC	A	Frame_Shift_Del	381	0.194225722	No	1	Clonal	.	.	.	.	No	Yes	No	No	Not tested
CS1-S	Sarcoma	BCL9	p.P516Lfs*5	Shared	1	147091500	AC	A	Frame_Shift_Del	598	0.301003344	No	1	Clonal	.	.	.	.	No	Yes	No	No	Not tested
CS1-C	Carcinoma	BCOR	p.A1013V	Private	X	39930903	G	A	Missense_Mutation	334	0.203592814	No	1	Clonal	Disease_causing	Passenger	Neutral	passenger	No	Yes	No	Yes	Not tested
CS1-C	Carcinoma	BCOR	p.N1459S	Shared	X	39921444	T	C	Missense_Mutation	118	0.483050847	No	1	Clonal	Disease_causing	Driver	Neutral	likely_pathogenic	No	Yes	No	Yes	Not tested
CS1-S	Sarcoma	BCOR	p.N1459S	Shared	X	39921444	T	C	Missense_Mutation	160	0.35	No	1	Clonal	Disease_causing	Driver	Neutral	likely_pathogenic	No	Yes	No	Yes	Not tested
CS1-C	Carcinoma	BCORL1	p.P1681Qfs*20	Shared	X	129190010	TC	T	Frame_Shift_Del	204	0.049019608	No	0.469	Subclonal	.	.	.	.	No	Yes	No	No	Not tested
CS1-S	Sarcoma	BCORL1	p.P1681Qfs*20	Shared	X	129190010	TC	T	Frame_Shift_Del	282	0.397163121	No	1	Clonal	.	.	.	.	No	Yes	No	No	Not tested
CS1-S	Sarcoma	BDP1	p.G1574D	Private	5	70811959	G	A	Missense_Mutation	280	0.117857143	No	0.415	Subclonal	Disease_causing	Passenger	Neutral	passenger	No	No	No	No	Not tested
CS1-S	Sarcoma	BHLHB9	p.K384Rfs*3	Private	X	102005068	GA	G	Frame_Shift_Del	263	0.357414449	No	1	Clonal	.	.	.	.	No	No	No	No	Not tested
CS1-S	Sarcoma	BICD1	p.K340Rfs*22	Private	12	32480404	GA	G	Frame_Shift_Del	288	0.350694444	No	1	Clonal	.	.	.	.	No	No	No	No	Not tested
CS1-S	Sarcoma	BIN2	p.A394T	Private	12	51685710	C	T	Missense_Mutation	312	0.051282051	No	0.18	Subclonal	Neutral	Passenger	Neutral	passenger	No	No	No	No	Not tested
CS1-C	Carcinoma	BIRC6	p.C3773R	Private	2	32740805	T	C	Missense_Mutation	147	0.040816327	No	0.431	Subclonal	Disease_causing	Passenger	Disease_causing	likely_pathogenic	No	No	No	No	Not tested
CS1-S	Sarcoma	BMP5	p.Y443Ifs*2	Private	6	55620369	A	AT	Frame_Shift_Ins	409	0.141809291	No	0.499	Subclonal	.	.	.	.	No	No	No	No	Not tested
CS1-S	Sarcoma	BPIFC	p.X199_splice	Private	22	32838719	C	T	Splice_Site	365	0.123287671	No	0.434	Subclonal	Disease_causing	.	.	passenger	No	No	No	No	Not tested
CS1-C	Carcinoma	BRD3	p.P288L	Shared	9	136913428	G	A	Missense_Mutation	177	0.389830508	No	1	Clonal	Disease_causing	Passenger	Neutral	passenger	No	Yes	No	No	Not tested
CS1-S	Sarcoma	BRD3	p.P288L	Shared	9	136913428	G	A	Missense_Mutation	233	0.394849785	No	1	Clonal	Disease_causing	Passenger	Neutral	passenger	No	Yes	No	No	Not tested
CS1-C	Carcinoma	BRD4	p.G1158R	Private	19	15350307	C	T	Missense_Mutation	255	0.066666667	No	0.637	Subclonal	Disease_causing	Passenger	Neutral	passenger	No	No	No	No	Not tested
CS1-C	Carcinoma	BRD4	p.E505D	Private	19	15367811	C	A	Missense_Mutation	222	0.054054054	No	0.517	Subclonal	Disease_causing	Passenger	Neutral	passenger	No	Yes	No	No	Not tested
CS1-S	Sarcoma	BRDT	p.D311G	Private	1	92442913	A	G	Missense_Mutation	414	0.425120773	No	1	Clonal	Disease_causing	Passenger	Neutral	passenger	No	No	No	No	Not tested
CS1-C	Carcinoma	BRINP3	p.R159Q	Private	1	190234137	C	T	Missense_Mutation	243	0.160493827	No	1	Clonal	Neutral	Passenger	Neutral	passenger	No	No	No	No	Not tested
CS1-S	Sarcoma	BRSK2	p.R401Gfs*4	Private	11	1466879	GC	G	Frame_Shift_Del	275	0.265454545	No	0.934	Clonal	.	.	.	.	No	No	No	No	Not tested
CS1-S	Sarcoma	BRWD1	p.R313H	Private	21	40650734	C	T	Missense_Mutation	189	0.17989418	No	0.633	Subclonal	Disease_causing	Passenger	Neutral	passenger	No	No	No	No	Not tested
CS1-S	Sarcoma	BRWD3	p.N1480Mfs*130	Private	X	79937551	AT	A	Frame_Shift_Del	289	0.24567474	No	0.864	Subclonal	.	.	.	.	No	No	No	No	Not tested
CS1-S	Sarcoma	BYSL	p.P415S	Private	6	41900373	C	T	Missense_Mutation	303	0.03960396	No	0.139	Subclonal	Neutral	Passenger	Neutral	passenger	No	No	No	No	Not tested
CS1-S	Sarcoma	BZW1	p.V107A	Private	2	201680223	T	C	Missense_Mutation	557	0.064631957	No	0.227	Subclonal	Disease_causing	Passenger	Neutral	passenger	No	No	No	No	Not tested
CS1-C	Carcinoma	C10orf118	p.N867Ifs*4	Private	10	115885657	AT	A	Frame_Shift_Del	186	0.252688172	No	1	Clonal	.	.	.	.	No	No	No	No	Not tested
CS1-C	Carcinoma	C10orf12	p.A421T	Private	10	98742408	G	A	Missense_Mutation	335	0.191044776	No	1	Clonal	Neutral	Passenger	Neutral	passenger	No	No	No	No	Not tested
CS1-C	Carcinoma	C10orf71	p.R27S	Private	10	50530671	G	T	Missense_Mutation	428	0.200934579	No	1	Clonal	Disease_causing	Passenger	Neutral	passenger	No	No	No	No	Not tested
CS1-C	Carcinoma	C10orf90	p.A698V	Private	10	128114440	G	A	Missense_Mutation	286	0.073426573	No	0.702	Subclonal	Disease_causing	Passenger	Neutral	passenger	No	No	No	No	Not tested
CS1-S	Sarcoma	C12orf55	p.E1419Gfs*9	Private	12	97024358	T	TA	Frame_Shift_Ins	205	0.307317073	No	1	Clonal	.	.	.	.	No	No	No	No	Not tested
CS1-S	Sarcoma	C12orf77	p.T7A	Private	12	25149258	T	C	Missense_Mutation	362	0.262430939	No	0.923	Clonal	Disease_causing	Passenger	Neutral	passenger	No	No	No	No	Not tested
CS1-C	Carcinoma	C16orf11	p.S289F	Private	16	614160	C	T	Missense_Mutation	141	0.226950355	No	1	Clonal	Neutral	Passenger	Neutral	passenger	No	No	No	No	Not tested
CS1-C	Carcinoma	C17orf99	p.R240H	Private	17	76162048	G	A	Missense_Mutation	226	0.283185841	No	1	Clonal	Neutral	Passenger	Neutral	passenger	No	No	No	No	Not tested
CS1-S	Sarcoma	C19orf40	p.D7G	Private	19	33464122	A	G	Missense_Mutation	274	0.065693431	No	0.231	Subclonal	Neutral	Passenger	.	passenger	No	No	No	No	Not tested
CS1-S	Sarcoma	C19orf54	p.P282Rfs*31	Private	19	41248548	CG	C	Frame_Shift_Del	333	0.252252252	No	0.888	Subclonal	.	.	.	.	No	No	No	No	Not tested
CS1-S	Sarcoma	C19orf66	p.P189Rfs*67	Private	19	10202223	TC	T	Frame_Shift_Del	227	0.149779736	No	0.527	Subclonal	.	.	.	.	No	No	No	No	Not tested
CS1-S	Sarcoma	C1orf100	p.D28Efs*28	Private	1	244538695	A	AAG	Frame_Shift_Ins	642	0.200934579	No	0.908	Clonal	.	.	.	.	No	No	No	No	Not tested
CS1-C	Carcinoma	C1orf110	p.P265Lfs*43	Private	1	162824669	AG	A	Frame_Shift_Del	471	0.135881104	No	1	Clonal	.	.	.	.	No	No	No	No	Not tested
CS1-S	Sarcoma	C1orf43	p.E61*	Private	1	154187038	C	A	Nonsense_Mutation	226	0.296460177	No	1	Clonal	Neutral	.	.	passenger	No	No	No	No	Not tested
CS1-S	Sarcoma	C1QL2	p.A21T	Private	2	119915785	C	T	Missense_Mutation	301	0.365448505	No	1	Clonal	Disease_causing	Passenger	Neutral	passenger	No	No	No	No	Not tested
CS1-S	Sarcoma	C1QL3	p.G73D	Private	10	16562847	C	T	Missense_Mutation	91	0.098901099	No	0.348	Subclonal	Disease_causing	Passenger	Disease_causing	likely_pathogenic	No	No	No	No	Not tested
CS1-S	Sarcoma	C1R	p.Q496H	Private	12	7188310	C	A	Missense_Mutation	328	0.167682927	No	0.59	Subclonal	Neutral	Passenger	Disease_causing	passenger	No	No	No	No	Not tested
CS1-S	Sarcoma	C22orf42	p.L170I	Private	22	32546452	G	T	Missense_Mutation	332	0.394578313	No	1	Clonal	Neutral	Passenger	Neutral	passenger	No	No	No	No	Not tested
CS1-C	Carcinoma	C2CD4C	p.K55Qfs*73	Private	19	408199	T	TG	Frame_Shift_Ins	304	0.190789474	No	1	Clonal	.	.	.	.	No	No	No	No	Not tested
CS1-C	Carcinoma	C2orf16	p.Q759H	Private	2	27801716	G	T	Missense_Mutation	338	0.275147929	No	1	Clonal	Neutral	Passenger	Neutral	passenger	No	No	No	No	Not tested
CS1-S	Sarcoma	C2orf47	p.C43Y	Private	2	200820649	G	A	Missense_Mutation	446	0.156950673	No	0.552	Subclonal	Neutral	Passenger	Neutral	passenger	No	No	No	No	Not tested
CS1-S	Sarcoma	C2orf71	p.P1188Lfs*126	Private	2	29293564	AG	A	Frame_Shift_Del	226	0.340707965	No	1	Clonal	.	.	.	.	No	No	No	No	Not tested
CS1-C	Carcinoma	C3	p.Q1152*	Private	19	6690675	G	A	Nonsense_Mutation	278	0.04676259	No	0.447	Subclonal	Neutral	.	.	passenger	No	No	No	No	Not tested
CS1-C	Carcinoma	C3orf18	p.E33K	Private	3	50603034	C	T	Missense_Mutation	230	0.039130435	No	0.374	Subclonal	Disease_causing	Passenger	Neutral	passenger	No	No	No	No	Not tested
CS1-C	Carcinoma	C4BPB	p.X20_splice	Private	1	207262935	G	C	Splice_Site	140	0.064285714	No	0.679	Subclonal	Disease_causing	.	.	passenger	No	No	No	No	Not tested
CS1-S	Sarcoma	C6ORF165	p.N477Ifs*4	Private	6	88144699	CA	C	Frame_Shift_Del	341	0.108504399	No	0.382	Subclonal	.	.	.	.	No	No	No	No	Not tested
CS1-S	Sarcoma	C8orf47	p.S234N	Private	8	99101946	G	A	Missense_Mutation	530	0.194339623	No	0.878	Subclonal	Neutral	Passenger	Neutral	passenger	No	No	No	No	Not tested
CS1-S	Sarcoma	CACFD1	p.P8S	Private	9	136325210	C	T	Missense_Mutation	145	0.268965517	No	0.946	Clonal	Neutral	Passenger	Neutral	passenger	No	No	No	No	Not tested
CS1-S	Sarcoma	CACHD1	p.D1170G	Private	1	65157081	A	G	Missense_Mutation	233	0.484978541	No	1	Clonal	Disease_causing	Passenger	Neutral	passenger	No	No	No	No	Not tested
CS1-C	Carcinoma	CACNA1A	p.G1863C	Private	19	13338283	C	A	Missense_Mutation	146	0.054794521	No	0.524	Subclonal	Disease_causing	Passenger	Neutral	passenger	No	No	No	No	Not tested
CS1-C	Carcinoma	CACNA1B	p.L37Cfs*64	Shared	9	140772487	CG	C	Frame_Shift_Del	195	0.030769231	No	0.294	Subclonal	.	.	.	.	No	No	No	No	Not tested
CS1-C	Carcinoma	CACNA1B	p.V1593I	Private	9	140968042	G	A	Missense_Mutation	101	0.168316832	No	1	Clonal	Disease_causing	Passenger	Disease_causing	likely_pathogenic	No	No	No	No	Not tested
CS1-S	Sarcoma	CACNA1B	p.L37Cfs*64	Shared	9	140772487	CG	C	Frame_Shift_Del	281	0.15658363	No	0.551	Subclonal	.	.	.	.	No	No	No	No	Not tested
CS1-S	Sarcoma	CACNA1E	p.G901Efs*6	Private	1	181701919	AG	A	Frame_Shift_Del	861	0.269454123	No	1	Clonal	.	.	.	.	No	No	No	No	Not tested
CS1-S	Sarcoma	CACNA2D2	p.X542_splice	Private	3	50414896	A	G	Splice_Site	351	0.136752137	No	0.481	Subclonal	Disease_causing	.	.	passenger	No	No	No	No	Not tested
CS1-S	Sarcoma	CACNA2D2	p.R239C	Private	3	50418495	G	A	Missense_Mutation	401	0.391521197	No	1	Clonal	Disease_causing	Passenger	Neutral	passenger	No	No	No	No	Not tested
CS1-C	Carcinoma	CACNB2	p.R646Q	Private	10	18828607	G	A	Missense_Mutation	249	0.044176707	No	0.422	Subclonal	Disease_causing	Passenger	Disease_causing	likely_pathogenic	No	No	No	No	Not tested
CS1-C	Carcinoma	CACNB4	p.R468Q	Private	2	152695793	C	T	Missense_Mutation	269	0.037174721	No	0.355	Subclonal	Disease_causing	Passenger	Neutral	passenger	No	No	No	No	Not tested
CS1-C	Carcinoma	CACNG7	p.I262V	Private	19	54445503	A	G	Missense_Mutation	425	0.223529412	No	1	Clonal	Disease_causing	Passenger	Neutral	passenger	No	No	No	No	Not tested
CS1-C	Carcinoma	CAD	p.R1667C	Private	2	27461437	C	T	Missense_Mutation	199	0.070351759	No	0.743	Subclonal	Disease_causing	Passenger	Neutral	passenger	No	No	No	No	Not tested
CS1-S	Sarcoma	CAD	p.P1171Hfs*40	Private	2	27456981	AC	A	Frame_Shift_Del	323	0.278637771	No	0.98	Clonal	.	.	.	.	No	No	No	No	Not tested
CS1-C	Carcinoma	CAMTA1	p.D129N	Private	1	7309633	G	A	Missense_Mutation	198	0.166666667	No	1	Clonal	Disease_causing	Passenger	Neutral	passenger	No	Yes	No	No	Not tested
CS1-C	Carcinoma	CAMTA2	p.S864L	Private	17	4875813	G	A	Missense_Mutation	70	0.128571429	No	1	Clonal	Disease_causing	Driver	Neutral	likely_pathogenic	No	No	No	No	Not tested
CS1-S	Sarcoma	CAPN13	p.K493E	Private	2	30965194	T	C	Missense_Mutation	342	0.073099415	No	0.257	Subclonal	Neutral	Passenger	Disease_causing	passenger	No	No	No	No	Not tested
CS1-S	Sarcoma	CASC3	p.Q575R	Private	17	38324175	A	G	Missense_Mutation	181	0.127071823	No	0.447	Subclonal	Disease_causing	Driver	.	likely_pathogenic	No	No	No	No	Not tested
CS1-S	Sarcoma	CASQ1	p.S356N	Private	1	160171042	G	A	Missense_Mutation	162	0.308641975	No	1	Clonal	Disease_causing	Passenger	Neutral	passenger	No	No	No	No	Not tested
CS1-S	Sarcoma	CAST	p.E516Kfs*10	Private	5	96090398	GA	G	Frame_Shift_Del	512	0.111328125	No	0.392	Subclonal	.	.	.	.	No	No	No	No	Not tested
CS1-S	Sarcoma	CASZ1	p.T196M	Private	1	10720512	G	A	Missense_Mutation	448	0.245535714	No	0.864	Subclonal	Disease_causing	Passenger	.	passenger	No	No	No	No	Not tested
CS1-S	Sarcoma	CATSPERG	p.G786D	Private	19	38853354	G	A	Missense_Mutation	466	0.429184549	No	1	Clonal	Neutral	Passenger	Neutral	passenger	No	No	No	No	Not tested
CS1-C	Carcinoma	CAV2	p.A93T	Private	7	116140440	G	A	Missense_Mutation	348	0.244252874	No	1	Clonal	Disease_causing	Passenger	Disease_causing	likely_pathogenic	No	No	No	No	Not tested
CS1-C	Carcinoma	CBR3	p.F116Lfs*13	Private	21	37510174	AT	A	Frame_Shift_Del	367	0.198910082	No	1	Clonal	.	.	.	.	No	No	No	No	Not tested
CS1-S	Sarcoma	CBX2	p.D99N	Private	17	77757537	G	A	Missense_Mutation	354	0.378531073	No	1	Clonal	Disease_causing	Passenger	.	passenger	No	No	No	No	Not tested
CS1-S	Sarcoma	CBX4	p.V545I	Private	17	77807808	C	T	Missense_Mutation	654	0.267584098	No	0.942	Clonal	Disease_causing	Passenger	.	passenger	No	No	No	No	Not tested
CS1-C	Carcinoma	CC2D1A	p.P466T	Private	19	14031409	C	A	Missense_Mutation	159	0.050314465	No	0.481	Subclonal	Disease_causing	Passenger	Neutral	passenger	No	No	No	No	Not tested
CS1-C	Carcinoma	CCDC105	p.A130T	Private	19	15122025	G	A	Missense_Mutation	73	0.205479452	No	1	Clonal	Neutral	Passenger	Neutral	passenger	No	No	No	No	Not tested

CS1-C	Carcinoma	CCDC135	p.E9V	Private	16	57731887	A	T	Missense_Mutation	[illegible]	[illegible]	No	0.582	Subclonal	Disease_causing	Passenger	Neutral	passenger	No	No	No	No	Not tested
CS1-C	Carcinoma	CCDC137	p.K179R	Private	17	79638812	A	G	Missense_Mutation	153	[illegible]	No	0.875	Clonal	Neutral	Passenger	Disease_causing	passenger	No	No	No	No	Not tested
CS1-C	Carcinoma	CCDC154	p.H53R	Private	16	1493863	T	C	Missense_Mutation	284	0.221830986	No	1	Clonal	Neutral	Passenger	.	passenger	No	No	No	No	Not tested
CS1-C	Carcinoma	CCDC157	p.S226N	Private	22	30766571	G	A	Missense_Mutation	150	0.26	No	1	Clonal	Neutral	Passenger	Neutral	passenger	No	No	No	No	Not tested
CS1-S	Sarcoma	CCDC158	p.D321N	Private	4	77300511	C	T	Missense_Mutation	399	0.105263158	No	0.37	Subclonal	Disease_causing	Passenger	Neutral	passenger	No	No	No	No	Not tested
CS1-S	Sarcoma	CCDC22	p.G502C	Private	X	49105350	G	T	Missense_Mutation	459	0.39869281	No	1	Clonal	Disease_causing	Passenger	.	passenger	No	No	No	No	Not tested
CS1-S	Sarcoma	CCDC33	p.A356T	Private	15	74574161	G	A	Missense_Mutation	203	0.403940887	No	1	Clonal	Neutral	Passenger	Neutral	passenger	No	No	No	No	Not tested
CS1-C	Carcinoma	CCDC34	p.N244Mfs*28	Private	11	27362972	AT	A	Frame_Shift_Del	123	0.203252033	No	1	Clonal	.	.	.	.	No	No	No	No	Not tested
CS1-S	Sarcoma	CCDC38	p.A150Qfs*24	Private	12	96292431	CT	C	Frame_Shift_Del	344	0.156976744	No	0.552	Subclonal	.	.	.	.	No	No	No	No	Not tested
CS1-S	Sarcoma	CCDC64B	p.E205Gfs*21	Private	16	3080698	T	TC	Frame_Shift_Ins	509	0.10805501	No	0.38	Subclonal	.	.	.	.	No	No	No	No	Not tested
CS1-S	Sarcoma	CCDC78	p.A383V	Private	16	773147	G	A	Missense_Mutation	187	0.411764706	No	1	Clonal	Neutral	Passenger	Neutral	passenger	No	No	No	No	Not tested
CS1-S	Sarcoma	CCDC96	p.A458V	Private	4	7043293	G	A	Missense_Mutation	658	0.139817629	No	0.492	Subclonal	Neutral	Passenger	Neutral	passenger	No	No	No	No	Not tested
CS1-S	Sarcoma	CCNL2	p.R434Afs*93	Private	1	1322873	CG	C	Frame_Shift_Del	301	0.388704319	No	1	Clonal	.	.	.	.	No	No	No	No	Not tested
CS1-S	Sarcoma	CCR9	p.V320Cfs*3	Private	3	45943232	G	GT	Frame_Shift_Ins	645	0.120930233	No	0.426	Subclonal	.	.	.	.	No	No	No	No	Not tested
CS1-C	Carcinoma	CD101	p.R594*	Private	1	117560945	C	T	Nonsense_Mutation	369	0.046070461	No	0.44	Subclonal	Neutral	.	.	passenger	No	No	No	No	Not tested
CS1-S	Sarcoma	CD209	p.G363D	Private	19	7808052	C	T	Missense_Mutation	508	0.409448819	No	1	Clonal	Disease_causing	Passenger	Neutral	passenger	No	No	No	No	Not tested
CS1-C	Carcinoma	CD248	p.P432Rfs*37	Private	11	66083203	CG	C	Frame_Shift_Del	196	0.173469388	No	1	Clonal	.	.	.	.	No	No	No	No	Not tested
CS1-C	Carcinoma	CDC42BPG	p.X84_splice	Private	11	64609283	A	G	Splice_Site	180	0.116666667	No	1	Clonal	Disease_causing	.	.	passenger	No	No	No	No	Not tested
CS1-S	Sarcoma	CDH16	p.K563Sfs*22	Private	16	66945905	TG	T	Frame_Shift_Del	492	0.386178862	No	1	Clonal	.	.	.	.	No	No	No	No	Not tested
CS1-C	Carcinoma	CDH23	p.R1582C	Private	10	73501562	C	T	Missense_Mutation	466	0.24248927	No	1	Clonal	Disease_causing	Passenger	.	passenger	No	No	No	No	Not tested
CS1-S	Sarcoma	CDH23	p.L944Pfs*31	Private	10	73464745	C	CT	Frame_Shift_Ins	537	0.240223464	No	0.845	Subclonal	.	.	.	.	No	No	No	No	Not tested
CS1-C	Carcinoma	CDHR2	p.P930S	Private	5	176013008	C	T	Missense_Mutation	483	0.223602484	No	1	Clonal	Neutral	Passenger	Neutral	passenger	No	No	No	No	Not tested
CS1-C	Carcinoma	CDK13	p.A162Pfs*174	Private	7	39990716	TG	T	Frame_Shift_Del	136	0.125	No	1	Clonal	.	.	.	.	No	No	No	No	Not tested
CS1-C	Carcinoma	CDR2	p.A394T	Private	16	22358471	C	T	Missense_Mutation	234	0.055555556	No	0.531	Subclonal	Neutral	Passenger	Neutral	passenger	No	No	No	No	Not tested
CS1-C	Carcinoma	CDS2	p.P306S	Private	20	5167420	C	T	Missense_Mutation	268	0.03358209	No	0.321	Subclonal	Disease_causing	Passenger	Neutral	passenger	No	No	No	No	Not tested
CS1-S	Sarcoma	CDT1	p.L111M	Private	16	88871055	C	A	Missense_Mutation	356	0.407303371	No	1	Clonal	Neutral	Passenger	Neutral	passenger	No	No	No	No	Not tested
CS1-S	Sarcoma	CEBPB	p.L121I	Private	20	48807931	C	A	Missense_Mutation	154	0.253246753	No	0.891	Subclonal	Disease_causing	Passenger	Neutral	passenger	No	No	No	No	Not tested
CS1-C	Carcinoma	CENPB	p.E449V	Private	20	3765785	T	A	Missense_Mutation	181	0.08839779	No	0.845	Clonal	Neutral	Passenger	Neutral	passenger	No	No	No	No	Not tested
CS1-C	Carcinoma	CENPI	p.X626_splice	Private	X	100402930	A	G	Splice_Site	310	0.2	No	1	Clonal	Disease_causing	.	.	passenger	No	No	No	No	Not tested
CS1-S	Sarcoma	CENPQ	p.N42Ifs*5	Private	6	49438701	GA	G	Frame_Shift_Del	156	0.134615385	No	0.474	Subclonal	.	.	.	.	No	No	No	No	Not tested
CS1-C	Carcinoma	CEP152	p.I1277Lfs*20	Private	15	49036442	AT	A	Frame_Shift_Del	154	0.227272727	No	1	Clonal	.	.	.	.	No	No	No	No	Not tested
CS1-S	Sarcoma	CEP170	p.A757D	Private	1	243328992	G	T	Missense_Mutation	397	0.113350126	No	0.512	Subclonal	Disease_causing	Passenger	Neutral	passenger	No	No	No	No	Not tested
CS1-C	Carcinoma	CEP170B	p.A999T	Private	14	105353571	G	A	Missense_Mutation	321	0.034267913	No	0.328	Subclonal	Neutral	Passenger	Neutral	passenger	No	No	No	No	Not tested
CS1-S	Sarcoma	CERS1	p.R173H	Private	19	18994968	C	T	Missense_Mutation	316	0.452531646	No	1	Clonal	Disease_causing	Passenger	Disease_causing	likely_pathogenic	No	No	No	No	Not tested
CS1-C	Carcinoma	CFD	p.Q167*	Private	19	861840	C	T	Nonsense_Mutation	139	0.050359712	No	0.481	Subclonal	Neutral	.	.	passenger	No	No	No	No	Not tested
CS1-C	Carcinoma	CFDP1	p.K109del	Private	16	75446507	CCTT	C	In_Frame_Del	240	0.175	No	1	Clonal	.	.	.	.	No	No	No	No	Not tested
CS1-C	Carcinoma	CFL1	p.E50A	Private	11	65623568	T	G	Missense_Mutation	183	0.076502732	No	0.731	Subclonal	Disease_causing	Passenger	Neutral	passenger	No	No	No	No	Not tested
CS1-S	Sarcoma	CFLAR	p.S285G	Private	2	202025214	A	G	Missense_Mutation	694	0.116714697	No	0.411	Subclonal	Neutral	Passenger	Neutral	passenger	No	No	No	No	Not tested
CS1-S	Sarcoma	CGNL1	p.X1125_splice	Private	15	57835979	T	C	Splice_Site	265	0.226415094	No	0.797	Subclonal	Disease_causing	.	.	passenger	No	No	No	No	Not tested
CS1-S	Sarcoma	CHD3	p.R599Vfs*16	Private	17	7798764	AC	A	Frame_Shift_Del	306	0.290849673	No	1	Clonal	.	.	.	.	No	No	No	No	Not tested
CS1-C	Carcinoma	CHDH	p.R124H	Private	3	53857665	C	T	Missense_Mutation	139	0.064748201	No	0.619	Subclonal	Disease_causing	Passenger	Neutral	passenger	No	No	No	No	Not tested
CS1-C	Carcinoma	CHGA	p.R391*	Private	14	93399077	C	T	Nonsense_Mutation	118	0.042372881	No	0.405	Subclonal	Neutral	.	.	passenger	No	No	No	No	Not tested
CS1-C	Carcinoma	CHMP1B	p.A77D	Private	18	11851740	C	A	Missense_Mutation	278	0.032374101	No	0.31	Subclonal	Disease_causing	Passenger	Neutral	passenger	No	No	No	No	Not tested
CS1-C	Carcinoma	CHMP7	p.A152P	Private	8	23106877	G	C	Missense_Mutation	70	0.171428571	No	1	Clonal	Neutral	Passenger	Neutral	passenger	No	No	No	No	Not tested
CS1-S	Sarcoma	CHPF	p.A540V	Private	2	220404814	G	A	Missense_Mutation	176	0.335227273	No	1	Clonal	Disease_causing	Passenger	Neutral	passenger	No	No	No	No	Not tested
CS1-S	Sarcoma	CHRD	p.X819_splice	Private	3	184105717	AG	A	Splice_Site	178	0.117977528	No	0.415	Subclonal	.	.	.	.	No	No	No	No	Not tested
CS1-S	Sarcoma	CHRNA7	p.W31C	Private	15	32322890	G	T	Missense_Mutation	407	0.228501229	No	0.804	Subclonal	Disease_causing	Passenger	Neutral	passenger	No	No	No	No	Not tested
CS1-C	Carcinoma	CHRNE	p.V285I	Private	17	4804152	C	T	Missense_Mutation	358	0.055865922	No	0.534	Subclonal	Disease_causing	Passenger	Disease_causing	likely_pathogenic	No	No	No	No	Not tested
CS1-S	Sarcoma	CHST13	p.A80T	Private	3	126260633	G	A	Missense_Mutation	206	0.427184466	No	1	Clonal	Disease_causing	Passenger	Neutral	passenger	No	No	No	No	Not tested
CS1-C	Carcinoma	CHST4	p.W305C	Private	16	71571495	G	T	Missense_Mutation	324	0.037037037	No	0.354	Subclonal	Disease_causing	Passenger	Disease_causing	likely_pathogenic	No	No	No	No	Not tested
CS1-C	Carcinoma	CHST7	p.R72Q	Private	X	46433581	G	A	Missense_Mutation	105	0.085714286	No	0.82	Clonal	Neutral	Passenger	Disease_causing	passenger	No	No	No	No	Not tested
CS1-C	Carcinoma	CHST8	p.R179H	Private	19	34263229	G	A	Missense_Mutation	165	0.206060606	No	1	Clonal	Neutral	Passenger	Neutral	passenger	No	No	No	No	Not tested
CS1-C	Carcinoma	CIC	p.R440H	Private	19	42793517	G	A	Missense_Mutation	231	0.073593074	No	0.704	Subclonal	Disease_causing	Passenger	.	passenger	No	Yes	No	No	Not tested
CS1-C	Carcinoma	CIITA	p.G655Rfs*94	Private	16	11001304	G	GC	Frame_Shift_Ins	156	0.153846154	No	1	Clonal	.	.	.	.	No	Yes	No	No	Not tested
CS1-C	Carcinoma	CLASP2	p.M965Wfs*33	Private	3	33602360	AT	A	Frame_Shift_Del	251	0.211155378	No	1	Clonal	.	.	.	.	No	No	No	No	Not tested
CS1-C	Carcinoma	CLDN23	p.A178T	Private	8	8560440	G	A	Missense_Mutation	154	0.064935065	No	0.621	Subclonal	Neutral	Passenger	Neutral	passenger	No	No	No	No	Not tested
CS1-S	Sarcoma	CLDN4	p.M4Ifs*108	Private	7	73245541	A	AT	Frame_Shift_Ins	307	0.25732899	No	0.906	Clonal	.	.	.	.	No	No	No	No	Not tested
CS1-C	Carcinoma	CLEC17A	p.V301M	Private	19	14717811	G	A	Missense_Mutation	170	0.076470588	No	0.731	Subclonal	Disease_causing	Passenger	Neutral	passenger	No	No	No	No	Not tested
CS1-S	Sarcoma	CLIP1	p.A612T	Private	12	122825917	C	T	Missense_Mutation	201	0.308457711	No	1	Clonal	Disease_causing	Driver	Neutral	likely_pathogenic	No	Yes	No	No	Not tested
CS1-S	Sarcoma	CLRN3	p.S100G	Private	10	129682071	T	C	Missense_Mutation	305	0.354098361	No	1	Clonal	Neutral	Passenger	Neutral	passenger	No	No	No	No	Not tested
CS1-S	Sarcoma	CLTC	p.V1647I	Private	17	57771124	G	A	Missense_Mutation	180	0.438888889	No	1	Clonal	Disease_causing	Passenger	Neutral	passenger	No	Yes	No	No	Not tested
CS1-C	Carcinoma	CLVS1	p.H230R	Private	8	62366758	A	G	Missense_Mutation	196	0.035714286	No	0.341	Subclonal	Disease_causing	Passenger	Neutral	passenger	No	No	No	No	Not tested
CS1-S	Sarcoma	CMPK2	p.V329I	Private	2	7001322	C	T	Missense_Mutation	261	0.398467433	No	1	Clonal	Disease_causing	Passenger	Neutral	passenger	No	No	No	No	Not tested
CS1-C	Carcinoma	CNGA3	p.M406V	Private	2	99012849	A	G	Missense_Mutation	392	0.06122449	No	0.585	Subclonal	Disease_causing	Passenger	Disease_causing	likely_pathogenic	No	No	No	No	Not tested
CS1-S	Sarcoma	CNKSR2	p.R632H	Private	X	21613156	G	A	Missense_Mutation	271	0.306273063	No	1	Clonal	Disease_causing	Passenger	Neutral	passenger	No	No	No	No	Not tested
CS1-C	Carcinoma	CNN2	p.A176T	Private	19	1036433	G	A	Missense_Mutation	313	0.063897764	No	0.611	Subclonal	Disease_causing	Passenger	Neutral	passenger	No	No	No	No	Not tested
CS1-C	Carcinoma	CNOT3	p.A38V	Private	19	54647197	C	T	Missense_Mutation	231	0.038961039	No	0.373	Subclonal	Disease_causing	Passenger	Neutral	passenger	No	Yes	No	No	Not tested
CS1-C	Carcinoma	CNR1	p.R405*	Private	6	88853781	G	A	Nonsense_Mutation	281	0.074733096	No	0.715	Subclonal	Disease_causing	.	.	passenger	No	No	No	No	Not tested
CS1-C	Carcinoma	CNTLN	p.I173T	Private	9	17226269	T	C	Missense_Mutation	139	0.086330935	No	0.825	Clonal	Disease_causing	Passenger	Neutral	passenger	No	No	No	No	Not tested
CS1-C	Carcinoma	CNTN6	p.G137C	Private	3	1320147	G	T	Missense_Mutation	277	0.0433213	No	0.414	Subclonal	Disease_causing	Passenger	Disease_causing	likely_pathogenic	No	No	No	No	Not tested
CS1-C	Carcinoma	CNTNAP5	p.E997Rfs*7	Private	2	125547711	C	CA	Frame_Shift_Ins	120	0.116666667	No	1	Clonal	.	.	.	.	No	No	No	No	Not tested
CS1-C	Carcinoma	COG1	p.K322Nfs*26	Shared	17	71196053	TA	T	Frame_Shift_Del	267	0.352059925	No	1	Clonal	.	.	.	.	No	No	No	No	Not tested
CS1-S	Sarcoma	COG1	p.K322Nfs*26	Shared	17	71196053	TA	T	Frame_Shift_Del	349	0.398280802	No	1	Clonal	.	.	.	.	No	No	No	No	Not tested
CS1-C	Carcinoma	COL15A1	p.P789Lfs*17	Private	9	101798630	AC	A	Frame_Shift_Del	184	0.25	No	1	Clonal	.	.	.	.	No	No	No	No	Not tested
CS1-S	Sarcoma	COL1A1	p.A283V	Private	17	48273988	G	A	Missense_Mutation	548	0.118613139	No	0.417	Subclonal	Disease_causing	Passenger	Disease_causing	likely_pathogenic	No	Yes	No	No	Not tested
CS1-C	Carcinoma	COL22A1	p.A180T	Private	8	139890113	C	T	Missense_Mutation	282	0.244680851	No	1	Clonal	Disease_causing	Passenger	Neutral	passenger	No	No	No	No	Not tested
CS1-S	Sarcoma	COL3A1	p.A1213Lfs*23	Private	2	189873755	GA	G	Frame_Shift_Del	228	0.263157895	No	0.926	Clonal	.	.	.	.	No	No	No	No	Not tested
CS1-C	Carcinoma	COL4A1	p.G371R	Private	13	110853210	C	T	Missense_Mutation	282	0.039007092	No	0.373	Subclonal	Disease_causing	Passenger	Disease_causing	likely_pathogenic	No	No	No	No	Not tested
CS1-C	Carcinoma	COL6A6	p.Q1012K	Shared	3	130292856	C	A	Missense_Mutation	191	0.403141361	No	1	Clonal	Neutral	Passenger	Neutral	passenger	No	No	No	No	Not tested
CS1-S	Sarcoma	COL6A6	p.Q1012K	Shared	3	130292856	C	A	Missense_Mutation	209	0.38277512	No	1	Clonal	Neutral	Passenger	Neutral	passenger	No	No	No	No	Not tested
CS1-S	Sarcoma	COL8A2	p.G223Vfs*14	Private	1	36564613	AC	A	Frame_Shift_Del	386	0.344559585	No	1	Clonal	.	.	.	.	No	No	No	No	Not tested
CS1-S	Sarcoma	COL8A2	p.G229Afs*8	Private	1	36564597	CG	C	Frame_Shift_Del	404	0.301980198	No	1	Clonal	.	.	.	.	No	No	No	No	Not tested
CS1-C	Carcinoma	COL9A2	p.X111_splice	Shared	1	40778242	TCACCGGAAGGC	T	Splice_Site	287	0.229965157	No	1	Clonal	.	.	.	.	No	No	No	No	Not tested
CS1-S	Sarcoma	COL9A2	p.X111_splice	Shared	1	40778242	TCACCGGAAGGC	T	Splice_Site	374	0.235294118	No	0.828	Clonal	.	.	.	.	No	No	No	No	Not tested
CS1-C	Carcinoma	COLGALT2	p.H591Y	Private	1	183908005	G	A	Missense_Mutation	449	0.158129176	No	1	Clonal	Disease_causing	Passenger	Neutral	passenger	No	No	No	No	Not tested
CS1-C	Carcinoma	COPS2	p.N442D	Private	15	49420176	T	C	Missense_Mutation	219	0.228310502	No	1	Clonal	Disease_causing	Passenger	.	passenger	No	No	No	No	Not tested
CS1-C	Carcinoma	CPAMD8	p.R718H	Private	19	17085965	C	T	Missense_Mutation	85	0.058823529	No	0.562	Subclonal	Disease_causing	Passenger	Neutral	passenger	No	No	No	No	Not tested
CS1-S	Sarcoma	CPEB1	p.L77F	Private	15	83240244	G	A	Missense_Mutation	343	0.131195335	No	0.462	Subclonal	Disease_causing	Passenger	.	passenger	No	No	No	No	Not tested
CS1-S	Sarcoma	CPED1	p.M482I	Private	7	120770217	G	A	Missense_Mutation	208	0.096153846	No	0.338	Subclonal	Disease_causing	Passenger	Neutral	passenger	No	No	No	No	Not tested
CS1-C	Carcinoma	CPNE6	p.R480W	Private	14	24546501	C	T	Missense_Mutation	432	0.037037037	No	0.354	Subclonal	Disease_causing	Passenger	Neutral	passenger	No	No	No	No	Not tested
CS1-S	Sarcoma	CPT1C	p.L641P	Private	19	50215121	T	C	Missense_Mutation	285	0.470175439	No	1	Clonal	Disease_causing	Passenger	Disease_causing	likely_pathogenic	No	No	No	No	Not tested
CS1-S	Sarcoma	CRB2	p.Q437H	Private	9	126132643	G	T	Missense_Mutation	648	0.416666667	No	1	Clonal	Neutral	Passenger	Neutral	passenger	No	No	No	No	Not tested
CS1-C	Carcinoma	CREB3L2	p.E439D	Private	7	137567328	C	A	Missense_Mutation	282	0.042553191	No	0.407	Subclonal	Disease_causing	Passenger	Neutral	passenger	No	Yes	No	No	Not tested
CS1-C	Carcinoma	CREBRF	p.H471Y	Private	5	172535815	C	T	Missense_Mutation	134	0.179104478	No	1	Clonal	Disease_causing	Driver	Neutral	likely_pathogenic	No	No	No	No	Not tested
CS1-C	Carcinoma	CRTAM	p.V305F	Private	11	122738212	G	T	Missense_Mutation	91	0.087912088	No	0.841	Clonal	Disease_causing	Passenger	Neutral	passenger	No	No	No	No	Not tested
CS1-C	Carcinoma	CRTAP	p.Y337H	Private	3	33174133	T	C	Missense_Mutation	177	0.04519774	No	0.432	Subclonal	Disease_causing	Passenger	Neutral	passenger	No	No	No	No	Not tested
CS1-C	Carcinoma	CRTC1	p.S588Afs*6	Shared	19	18887992	TC	T	Frame_Shift_Del	178	0.061797753	No	0.591	Subclonal	.	.	.	.	No	Yes	No	No	Not tested
CS1-S	Sarcoma	CRTC1	p.S588Afs*6	Shared	19	18887992	TC	T	Frame_Shift_Del	269	0.219330855	No	0.772	Subclonal	.	.	.	.	No	Yes	No	No	Not tested
CS1-C	Carcinoma	CRYBG3	p.W694*	Private	3	97618062	G	A	Nonsense_Mutation	334	0.203592814	No	1	Clonal	Disease_causing	.	.	passenger	No	No	No	No	Not tested
CS1-S	Sarcoma	CRYBG3	p.Q1337H	Private	3	97594049	G	T	Missense_Mutation	370	0.427027027	No	1	Clonal	.	Passenger	.	passenger	No	No	No	No	Not tested
CS1-S	Sarcoma	CRYGS	p.R158Q	Private	3	186256549	C	T	Missense_Mutation	498	0.259036145	No	0.912	Clonal	Disease_causing	Passenger	Neutral	passenger	No	No	No	No	Not tested
CS1-C	Carcinoma	CSF2RB	p.R799H	Shared	22	37334246	G	A	Missense_Mutation	366	0.393442623	No	1	Clonal	Neutral	Passenger	Disease_causing	passenger	No	No	No	No	Not tested
CS1-S	Sarcoma	CSF2RB	p.R799H	Shared	22	37334246	G	A	Missense_Mutation	473	0.433403805	No	1	Clonal	Neutral	Passenger	Disease_causing	passenger	No	No	No	No	Not tested
CS1-C	Carcinoma	CSMD3	p.A2917V	Private	8	113304804	G	A	Missense_Mutation	113	0.230088496	No	1	Clonal	Disease_causing	Passenger	Neutral	passenger	No	No	No	No	Not tested
CS1-S	Sarcoma	CSN2	p.K2T	Private	4	70826709	T	G	Missense_Mutation	292	0.04109589	No	0.145	Subclonal	Neutral	Passenger	.	passenger	No	No	No	No	Not tested
CS1-C	Carcinoma	CSNK1E	p.A180V	Private	22	38696755	G	A	Missense_Mutation	153	0.222222222	No	1	Clonal	Disease_causing	Driver	Neutral	likely_pathogenic	No	No	No	No	Not tested
CS1-S	Sarcoma	CSPG4	p.E1216D	Private	15	75979758	T	G	Missense_Mutation	126	0.277777778	No	0.977	Clonal	Neutral	Passenger	Neutral	passenger	No	No	No	No	Not tested
CS1-C	Carcinoma	CT83	p.S32L	Private	X	115593155	G	A	Missense_Mutation	320	0.059375	No	0.568	Subclonal	Neutral	Passenger	.	passenger	No	No	No	No	Not tested
CS1-C	Carcinoma	CTNND2	p.T340I	Private	5	11384935	G	A	Missense_Mutation	215	0.218604651	No	1	Clonal	Disease_causing	Passenger	Neutral	passenger	No	No	No	No	Not tested
CS1-S	Sarcoma	CTRC	p.G193C	Private	1	15771184	G	T	Missense_Mutation	241	0.253112033	No	0.891	Subclonal	Disease_causing	Passenger	Disease_causing	likely_pathogenic	No	No	No	No	Not tested
CS1-C	Carcinoma	CTSD	p.E69K	Private	11	1782562	C	T	Missense_Mutation	271	0.081180812	No	0.776	Clonal	Disease_causing	Passenger	Neutral	passenger	No	No	No	No	Not tested
CS1-S	Sarcoma	CWF19L2	p.N326Ifs*19	Private	11	107299980	AT	A	Frame_Shift_Del	319	0.122257053	No	0.43	Subclonal	.	.	.	.	No	No	No	No	Not tested
CS1-S	Sarcoma	CXXC1	p.R574C	Private	18	47809340	G	A	Missense_Mutation	130	0.138461538	No	0.487	Subclonal	Disease_causing	Passenger	Neutral	passenger	No	No	No	No	Not tested
CS1-S	Sarcoma	CYP11B1	p.R412C	Private	8	143956537	G	A	Missense_Mutation	486	0.590534979	No	1	Clonal	Disease_causing	Passenger	Neutral	passenger	No	No	No	No	Not tested
CS1-S	Sarcoma	CYP11B2	p.L496Sfs*178	Private	8	143993421	AG	A	Frame_Shift_Del	320	0.50625	No	1	Clonal	.	.	.	.	No	No	No	No	Not tested
CS1-S	Sarcoma	CYP1B1	p.R158H	Private	2	38302059	C	T	Missense_Mutation	323	0.269349845	No	0.948	Clonal	Disease_causing	Passenger	Neutral	passenger	No	No	No	No	Not tested
CS1-C	Carcinoma	CYP2C18	p.R442C	Private	10	96495052	C	T	Missense_Mutation	139	0.035971223	No	0.344	Subclonal	Disease_causing	Passenger	Neutral	passenger	No	No	No	No	Not tested
CS1-C	Carcinoma	CYP4F12	p.T152M	Private	19	15791259	C	T	Missense_Mutation	200	0.305	No	1	Clonal	Disease_causing	Passenger	Neutral	passenger	No	No	No	No	Not tested
CS1-C	Carcinoma	CYTH1	p.K91E	Private	17	76698289	T	C	Missense_Mutation	183	0.06557377	No	0.627	Subclonal	Disease_causing	Passenger	Neutral	passenger	No	No	No	No	Not tested
CS1-S	Sarcoma	CYTH1	p.N241Mfs*28	Private	17	76694439	AT	A	Frame_Shift_Del	172	0.244186047	No	0.859	Subclonal	.	.	.	.	No	No	No	No	Not tested
CS1-C	Carcinoma	CYTIP	p.A46T	Private	2	158300397	C	T	Missense_Mutation	116	0.077586207	No	0.742	Subclonal	Disease_causing	Passenger	Neutral	passenger	No	No	No	No	Not tested
CS1-C	Carcinoma	DAB2IP	p.H997Pfs*4	Shared	9	124536636	A	AC	Frame_Shift_Ins	135	0.177777778	No	1	Clonal	.	.	.	.	No	No	No	No	Not tested
CS1-S	Sarcoma	DAB2IP	p.H997Pfs*4	Shared	9	124536636	A	AC	Frame_Shift_Ins	172	0.412790698	No	1	Clonal	.	.	.	.	No	No	No	No	Not tested
CS1-C	Carcinoma	DACT2	p.S178L	Private	6	168710973	G	A	Missense_Mutation	116	0.051724138	No	0.495	Subclonal	Disease_causing	Passenger	Neutral	passenger	No	No	No	No	Not tested
CS1-C	Carcinoma	DAPK2	p.A50T	Private	15	64275898	C	T	Missense_Mutation	439	0.10022779	No	0.958	Clonal	Disease_causing	Passenger	Neutral	passenger	No	No	No	No	Not tested
CS1-C	Carcinoma	DCBLD1	p.Q397Rfs*7	Private	6	117861913	TC	T	Frame_Shift_Del	328	0.167682927	No	1	Clonal	.	.	.	.	No	No	No	No	Not tested
CS1-S	Sarcoma	DCBLD2	p.A678T	Private	3	98518512	C	T	Missense_Mutation	369	0.111111111	No	0.391	Subclonal	Disease_causing	Passenger	Neutral	passenger	No	No	No	No	Not tested
CS1-S	Sarcoma	DCC	p.D1152*	Private	18	50985656	A	AC	Frame_Shift_Ins	333	0.252252252	No	0.888	Subclonal	.	.	.	.	No	No	No	No	Not tested
CS1-C	Carcinoma	DCDC2	p.C139Y	Private	6	24302205	C	T	Missense_Mutation	198	0.191919192	No	1	Clonal	Disease_causing	Passenger	Disease_causing	likely_pathogenic	No	No	No	No	Not tested
CS1-C	Carcinoma	DCHS1	p.R2173Q	Private	11	6647458	C	T	Missense_Mutation	434	0.267281106	No	1	Clonal	Neutral	Passenger	Neutral	passenger	No	No	No	No	Not tested
CS1-C	Carcinoma	DCHS1	p.Y1068F	Private	11	6653540	T	A	Missense_Mutation	316	0.050632911	No	0.484	Subclonal	Disease_causing	Passenger	Neutral	passenger	No	No	No	No	Not tested
CS1-C	Carcinoma	DCLK1	p.Y612H	Private	13	36382390	A	G	Missense_Mutation	181	0.071823204	No	0.687	Subclonal	Disease_causing	Passenger	Neutral	passenger	No	No	No	No	Not tested
CS1-C	Carcinoma	DCP1A	p.A14V	Private	3	53381504	G	A	Missense_Mutation	250	0.048	No	0.459	Subclonal	.	Passenger	.	passenger	No	No	No	No	Not tested
CS1-C	Carcinoma	DDB2	p.A118V	Private	11	47238497	C	T	Missense_Mutation	113	0.053097345	No	0.508	Subclonal	Disease_causing	Passenger	Neutral	passenger	No	Yes	No	No	Not tested
CS1-C	Carcinoma	DDC	p.D92N	Private	7	50607654	C	T	Missense_Mutation	161	0.043478261	No	0.416	Subclonal	Disease_causing	Passenger	Neutral	passenger	No	No	No	No	Not tested
CS1-S	Sarcoma	DDN	p.R197W	Private	12	49392070	G	A	Missense_Mutation	330	0.4	No	1	Clonal	Neutral	Passenger	Neutral	passenger	No	No	No	No	Not tested
CS1-C	Carcinoma	DDR1	p.R248W	Private	6	30859855	C	T	Missense_Mutation	179	0.167597765	No	1	Clonal	Disease_causing	Passenger	Neutral	passenger	No	No	No	No	Not tested
CS1-S	Sarcoma	DDX10	p.X62_splice	Private	11	108536067	G	A	Splice_Site	254	0.307086614	No	1	Clonal	Disease_causing	.	.	likely_pathogenic	No	Yes	No	No	Not tested
CS1-S	Sarcoma	DDX23	p.R217H	Private	12	49231410	C	T	Missense_Mutation	177	0.15819209	No	0.557	Subclonal	Disease_causing	Passenger	Neutral	passenger	No	No	No	No	Not tested
CS1-C	Carcinoma	DDX42	p.A359V	Private	17	61886232	C	T	Missense_Mutation	142	0.077464789	No	0.741	Subclonal	Disease_causing	Passenger	Neutral	passenger	No	No	No	No	Not tested
CS1-S	Sarcoma	DDX42	p.A67T	Private	17	61864608	G	A	Missense_Mutation	398	0.135678392	No	0.477	Subclonal	Disease_causing	Passenger	Neutral	passenger	No	No	No	No	Not tested
CS1-C	Carcinoma	DDX50	p.K339Nfs*3	Private	10	70673881	CA	C	Frame_Shift_Del	124	0.241935484	No	1	Clonal	.	.	.	.	No	No	No	No	Not tested
CS1-C	Carcinoma	DENND1A	p.X613_splice	Private	9	126144905	C	A	Splice_Site	50	0.22	No	1	Clonal	Disease_causing	.	.	passenger	No	No	No	No	Not tested
CS1-S	Sarcoma	DENND1C	p.I31Sfs*81	Private	19	6479905	TG	T	Frame_Shift_Del	261	0.187739464	No	0.661	Subclonal	.	.	.	.	No	No	No	No	Not tested
CS1-S	Sarcoma	DENND4A	p.W1716Gfs*19	Private	15	65959878	CA	C	Frame_Shift_Del	369	0.371273713	No	1	Clonal	.	.	.	.	No	No	No	No	Not tested
CS1-S	Sarcoma	DENND4A	p.M800Cfs*75	Private	15	65994674	AT	A	Frame_Shift_Del	184	0.22826087	No	0.803	Subclonal	.	.	.	.	No	No	No	No	Not tested
CS1-C	Carcinoma	DENND6B	p.Q453Rfs*55	Private	22	50751527	TG	T	Frame_Shift_Del	56	0.285714286	No	1	Clonal	.	.	.	.	No	No	No	No	Not tested
CS1-S	Sarcoma	DGKG	p.H352Y	Private	3	185986652	G	A	Missense_Mutation	372	0.14516129	No	0.511	Subclonal	Disease_causing	Passenger	Disease_causing	likely_pathogenic	No	No	No	No	Not tested
CS1-S	Sarcoma	DGKH	p.S327L	Private	13	42740672	C	T	Missense_Mutation	158	0.316455696	No	1	Clonal	Disease_causing	Passenger	Neutral	passenger	No	No	No	No	Not tested
CS1-S	Sarcoma	DGKQ	p.R139C	Private	4	962635	G	A	Missense_Mutation	298	0.298657718	No	1	Clonal	Disease_causing	Passenger	Disease_causing	likely_pathogenic	No	No	No	No	Not tested
CS1-C	Carcinoma	DHRS13	p.A300V	Private	17	27225694	G	A	Missense_Mutation	360	0.052777778	No	0.505	Subclonal	Disease_causing	Passenger	Neutral	passenger	No	No	No	No	Not tested
CS1-C	Carcinoma	DHRS7C	p.Y213C	Private	17	9676176	T	C	Missense_Mutation	274	0.189781022	No	1	Clonal	Disease_causing	Passenger	Neutral	passenger	No	No	No	No	Not tested
CS1-S	Sarcoma	DHTKD1	p.L658P	Private	10	12148321	T	C	Missense_Mutation	434	0.087557604	No	0.308	Subclonal	Disease_causing	Passenger	Disease_causing	likely_pathogenic	No	No	No	No	Not tested
CS1-S	Sarcoma	DHX37	p.E26Sfs*15	Private	12	125473493	CG	C	Frame_Shift_Del	343	0.239067055	No	0.841	Subclonal	.	.	.	.	No	No	No	No	Not tested
CS1-C	Carcinoma	DICER1	p.A1816D	Private	14	95557620	G	T	Missense_Mutation	290	0.196551724	No	1	Clonal	Disease_causing	Passenger	Neutral	passenger	No	Yes	No	No	Not tested
CS1-C	Carcinoma	DICER1	p.D1709E	Shared	14	95560462	A	C	Missense_Mutation	82	0.475609756	No	1	Clonal	Disease_causing	Passenger	Disease_causing	likely_pathogenic	No	Yes	No	No	Not tested
CS1-S	Sarcoma	DICER1	p.V1378Cfs*19	Private	14	95566192	A	AG	Frame_Shift_Ins	204	0.382352941	No	1	Clonal	.	.	.	.	No	Yes	No	No	Not tested
CS1-S	Sarcoma	DICER1	p.D1709E	Shared	14	95560462	A	C	Missense_Mutation	92	0.434782609	No	1	Clonal	Disease_causing	Passenger	Disease_causing	likely_pathogenic	No	Yes	No	No	Not tested
CS1-C	Carcinoma	DLAT	p.A298T	Private	11	111908101	G	A	Missense_Mutation	273	0.271062271	No	1	Clonal	Neutral	Passenger	Neutral	passenger	No	No	No	No	Not tested
CS1-S	Sarcoma	DLGAP3	p.S86*	Private	1	35370730	A	AC	Frame_Shift_Ins	255	0.270588235	No	0.952	Clonal	.	.	.	.	No	No	No	No	Not tested
CS1-S	Sarcoma	DLGAP3	p.D403Mfs*65	Private	1	35365774	TC	T	Frame_Shift_Del	570	0.121052632	No	0.426	Subclonal	.	.	.	.	No	No	No	No	Not tested
CS1-C	Carcinoma	DLST	p.K409N	Private	14	75367936	G	T	Missense_Mutation	157	0.038216561	No	0.365	Subclonal	Disease_causing	Passenger	Neutral	passenger	No	No	No	No	Not tested
CS1-C	Carcinoma	DMKN	p.C15Y	Private	19	36004334	C	T	Missense_Mutation	265	0.105660377	No	1	Clonal	Neutral	Passenger	Neutral	passenger	No	No	No	No	Not tested
CS1-S	Sarcoma	DMRT3	p.A124V	Private	9	977372	C	T	Missense_Mutation	153	0.124183007	No	0.437	Subclonal	Neutral	Passenger	Neutral	passenger	No	No	No	No	Not tested
CS1-C	Carcinoma	DNAH17	p.A4363T	Private	17	76421550	C	T	Missense_Mutation	263	0.053231939	No	0.509	Subclonal	Disease_causing	Passenger	Neutral	passenger	No	No	No	No	Not tested
CS1-C	Carcinoma	DNAH5	p.R2502H	Shared	5	13810272	C	T	Missense_Mutation	132	0.340909091	No	1	Clonal	Neutral	Passenger	Neutral	passenger	No	No	No	No	Not tested
CS1-S	Sarcoma	DNAH5	p.R2502H	Shared	5	13810272	C	T	Missense_Mutation	183	0.459016393	No	1	Clonal	Neutral	Passenger	Neutral	passenger	No	No	No	No	Not tested
CS1-C	Carcinoma	DNAH7	p.L1074Ffs*4	Private	2	196801373	C	CA	Frame_Shift_Ins	175	0.137142857	No	1	Clonal	.	.	.	.	No	No	No	No	Not tested
CS1-C	Carcinoma	DNAH8	p.C1936Y	Private	6	38831796	G	A	Missense_Mutation	89	0.247191011	No	1	Clonal	Disease_causing	Passenger	Neutral	passenger	No	No	No	No	Not tested
CS1-C	Carcinoma	DNAI2	p.L251R	Private	17	72295884	T	G	Missense_Mutation	144	0.034722222	No	0.332	Subclonal	Disease_causing	Passenger	Neutral	passenger	No	No	No	No	Not tested
CS1-S	Sarcoma	DNAI2	p.X450_splice	Private	17	72306155	G	T	Splice_Site	193	0.487046632	No	1	Clonal	Disease_causing	.	.	passenger	No	No	No	No	Not tested
CS1-C	Carcinoma	DNAJA2	p.K215E	Private	16	46998654	T	C	Missense_Mutation	149	0.040268456	No	0.385	Subclonal	Disease_causing	Passenger	Neutral	passenger	No	No	No	No	Not tested
CS1-S	Sarcoma	DNAJA3	p.P378Lfs*39	Private	16	4498735	TC	T	Frame_Shift_Del	257	0.412451362	No	1	Clonal	.	.	.	.	No	No	No	No	Not tested
CS1-C	Carcinoma	DNAJB2	p.T299Pfs*54	Private	2	220149621	TG	T	Frame_Shift_Del	213	0.211267606	No	1	Clonal	.	.	.	.	No	No	No	No	Not tested
CS1-S	Sarcoma	DNAJC5G	p.G98D	Private	2	27500801	G	A	Missense_Mutation	449	0.38752784	No	1	Clonal	Disease_causing	Passenger	Neutral	passenger	No	No	No	No	Not tested
CS1-S	Sarcoma	DNM3	p.P119S	Private	1	171956915	C	T	Missense_Mutation	732	0.094262295	No	0.426	Subclonal	Disease_causing	Passenger	Disease_causing	likely_pathogenic	No	No	No	No	Not tested
CS1-S	Sarcoma	DNMBP	p.L879Q	Private	10	101659742	A	T	Missense_Mutation	327	0.418960245	No	1	Clonal	Disease_causing	Passenger	Neutral	passenger	No	No	No	No	Not tested
CS1-C	Carcinoma	DOCK3	p.S330T	Private	3	51198084	T	A	Missense_Mutation	185	0.086486486	No	0.827	Clonal	Disease_causing	Passenger	Neutral	passenger	No	No	No	No	Not tested
CS1-C	Carcinoma	DOCK3	p.P1852Qfs*45	Shared	3	51417603	AC	A	Frame_Shift_Del	209	0.043062201	No	0.412	Subclonal	.	.	.	.	No	No	No	No	Not tested
CS1-S	Sarcoma	DOCK3	p.P1852Qfs*45	Shared	3	51417603	AC	A	Frame_Shift_Del	256	0.3359375	No	1	Clonal	.	.	.	.	No	No	No	No	Not tested
CS1-S	Sarcoma	DOCK3	p.R421C	Private	3	51263088	C	T	Missense_Mutation	160	0.29375	No	1	Clonal	Disease_causing	Passenger	Neutral	passenger	No	No	No	No	Not tested
CS1-S	Sarcoma	DOCK8	p.F1113Lfs*2	Private	9	405014	CT	C	Frame_Shift_Del	312	0.423076923	No	1	Clonal	.	.	.	.	No	No	No	No	Not tested
CS1-C	Carcinoma	DOCK9	p.S1666G	Private	13	99478153	T	C	Missense_Mutation	88	0.056818182	No	0.543	Subclonal	Disease_causing	Passenger	Neutral	passenger	No	No	No	No	Not tested
CS1-S	Sarcoma	DOCK9	p.Q720H	Private	13	99538062	C	A	Missense_Mutation	200	0.455	No	1	Clonal	Disease_causing	Passenger	Neutral	passenger	No	No	No	No	Not tested
CS1-C	Carcinoma	DPEP2	p.R147H	Private	16	68026047	C	T	Missense_Mutation	90	0.111111111	No	1	Clonal	Disease_causing	Passenger	Neutral	passenger	No	No	No	No	Not tested
CS1-C	Carcinoma	DPH3	p.P27T	Private	3	16306305	G	T	Missense_Mutation	246	0.040650407	No	0.389	Subclonal	Disease_causing	Passenger	.	passenger	No	No	No	No	Not tested
CS1-S	Sarcoma	DPP9	p.G761R	Private	19	4683539	C	T	Missense_Mutation	267	0.037453184	No	0.132	Subclonal	Disease_causing	Passenger	Disease_causing	likely_pathogenic	No	No	No	No	Not tested
CS1-C	Carcinoma	DPY19L3	p.N359D	Private	19	32945932	A	G	Missense_Mutation	110	0.227272727	No	1	Clonal	Disease_causing	Driver	Neutral	likely_pathogenic	No	No	No	No	Not tested
CS1-C	Carcinoma	DPYSL3	p.A496T	Private	5	146778782	C	T	Missense_Mutation	296	0.037162162	No	0.355	Subclonal	Disease_causing	Passenger	Disease_causing	likely_pathogenic	No	No	No	No	Not tested
CS1-C	Carcinoma	DROSHA	p.L612Pfs*8	Private	5	31493321	A	AG	Frame_Shift_Ins	256	0.21875	No	1	Clonal	.	.	.	.	No	Yes	No	No	Not tested
CS1-C	Carcinoma	DSCAML1	p.G534C	Private	11	117389271	C	A	Missense_Mutation	309	0.200647249	No	1	Clonal	Disease_causing	Passenger	Neutral	passenger	No	No	No	No	Not tested
CS1-C	Carcinoma	DSE	p.A54T	Private	6	116720573	G	A	Missense_Mutation	399	0.042606516	No	0.407	Subclonal	Neutral	Passenger	Neutral	passenger	No	No	No	No	Not tested
CS1-C	Carcinoma	DSEL	p.N739D	Private	18	65179661	T	C	Missense_Mutation	272	0.183823529	No	1	Clonal	Neutral	Passenger	Neutral	passenger	No	No	No	No	Not tested
CS1-S	Sarcoma	DSEL	p.I210Yfs*30	Private	18	65181247	AT	A	Frame_Shift_Del	370	0.251351351	No	0.884	Subclonal	.	.	.	.	No	No	No	No	Not tested
CS1-C	Carcinoma	DTX1	p.V508I	Private	12	113532982	G	A	Missense_Mutation	359	0.077994429	No	0.746	Subclonal	Disease_causing	Passenger	Neutral	passenger	No	No	No	No	Not tested
CS1-S	Sarcoma	DVL1	p.P646Rfs*3	Private	1	1271597	CG	C	Frame_Shift_Del	484	0.115702479	No	0.407	Subclonal	.	.	.	.	No	No	No	No	Not tested
CS1-C	Carcinoma	DVL3	p.N253D	Private	3	183883273	A	G	Missense_Mutation	198	0.181818182	No	1	Clonal	Disease_causing	Passenger	Neutral	passenger	No	No	No	No	Not tested
CS1-C	Carcinoma	EBF1	p.V471A	Private	5	158139299	A	G	Missense_Mutation	307	0.052117264	No	0.498	Subclonal	Disease_causing	Passenger	Neutral	passenger	No	Yes	No	No	Not tested
CS1-C	Carcinoma	EBF3	p.A396V	Private	10	131640538	G	A	Missense_Mutation	201	0.2039801	No	1	Clonal	Disease_causing	Passenger	Neutral	passenger	No	No	No	No	Not tested
CS1-C	Carcinoma	ECEL1	p.L7P	Private	2	233351344	A	G	Missense_Mutation	95	0.105263158	No	1	Clonal	Disease_causing	Passenger	Disease_causing	likely_pathogenic	No	No	No	No	Not tested
CS1-C	Carcinoma	ECHS1	p.I37V	Private	10	135184241	T	C	Missense_Mutation	231	0.090909091	No	0.869	Clonal	Neutral	Passenger	Neutral	passenger	No	No	No	No	Not tested
CS1-S	Sarcoma	EDA	p.K175Rfs*105	Private	X	69243084	GA	G	Frame_Shift_Del	124	0.483870968	No	1	Clonal	.	.	.	.	No	No	No	No	Not tested
CS1-C	Carcinoma	EDEM2	p.A193T	Private	20	33722666	C	T	Missense_Mutation	158	0.17721519	No	1	Clonal	Disease_causing	Passenger	Neutral	passenger	No	No	No	No	Not tested
CS1-C	Carcinoma	EDEM3	p.A74V	Private	1	184706829	G	A	Missense_Mutation	253	0.043478261	No	0.459	Subclonal	Disease_causing	Passenger	Neutral	passenger	No	No	No	No	Not tested
CS1-S	Sarcoma	EEPD1	p.L461P	Private	7	36336668	T	C	Missense_Mutation	575	0.44	No	1	Clonal	Disease_causing	Driver	Disease_causing	likely_pathogenic	No	No	No	No	Not tested
CS1-C	Carcinoma	EFEMP1	p.R148H	Private	2	56144874	C	T	Missense_Mutation	119	0.050420168	No	0.532	Subclonal	Disease_causing	Passenger	Neutral	passenger	No	No	No	No	Not tested
CS1-S	Sarcoma	EFEMP2	p.E126K	Private	11	65638121	C	T	Missense_Mutation	302	0.380794702	No	1	Clonal	Disease_causing	Passenger	Disease_causing	likely_pathogenic	No	No	No	No	Not tested
CS1-S	Sarcoma	EFHC1	p.D408Tfs*7	Private	6	52334207	CA	C	Frame_Shift_Del	437	0.148741419	No	0.523	Subclonal	.	.	.	.	No	No	No	No	Not tested
CS1-S	Sarcoma	EFHC2	p.A439Cfs*10	Private	X	44094659	C	CA	Frame_Shift_Ins	302	0.119205298	No	0.419	Subclonal	.	.	.	.	No	No	No	No	Not tested
CS1-S	Sarcoma	EFNB3	p.H5Ifs*54	Private	17	7608922	GC	G	Frame_Shift_Del	194	0.31443299	No	1	Clonal	.	.	.	.	No	No	No	No	Not tested
CS1-C	Carcinoma	EFTUD1	p.A1093V	Private	15	82422799	G	A	Missense_Mutation	370	0.075675676	No	0.724	Subclonal	Disease_causing	Passenger	Neutral	passenger	No	No	No	No	Not tested
CS1-S	Sarcoma	EFTUD2	p.K359Rfs*27	Private	17	42945247	CT	C	Frame_Shift_Del	106	0.141509434	No	0.498	Subclonal	.	.	.	.	No	No	No	No	Not tested
CS1-C	Carcinoma	EGFR	p.A647T	Private	7	55240695	G	A	Missense_Mutation	302	0.056291391	No	0.538	Subclonal	Disease_causing	Passenger	Neutral	passenger	No	Yes	Yes	Yes	Not tested
CS1-S	Sarcoma	EGR2	p.Q371Pfs*13	Private	10	64573288	G	GA	Frame_Shift_Ins	597	0.262981575	No	0.925	Clonal	.	.	.	.	No	No	No	No	Not tested
CS1-S	Sarcoma	EI24	p.G188*	Private	11	125449989	G	T	Nonsense_Mutation	106	0.103773585	No	0.365	Subclonal	Neutral	.	.	passenger	No	No	No	No	Not tested
CS1-C	Carcinoma	EIF2D	p.P470S	Private	1	206769168	G	A	Missense_Mutation	289	0.076124567	No	0.804	Clonal	Disease_causing	Passenger	Neutral	passenger	No	No	No	No	Not tested
CS1-S	Sarcoma	EIF3E	p.R191Q	Private	8	109241324	C	T	Missense_Mutation	147	0.149659864	No	0.676	Subclonal	Disease_causing	Passenger	Neutral	passenger	No	Yes	No	No	Not tested
CS1-C	Carcinoma	ELAVL3	p.N322K	Private	19	11565479	G	T	Missense_Mutation	328	0.067073171	No	0.641	Subclonal	Disease_causing	Passenger	Neutral	passenger	No	No	No	No	Not tested
CS1-S	Sarcoma	ELMO1	p.A641T	Private	7	36901326	C	T	Missense_Mutation	555	0.162162162	No	0.571	Subclonal	Disease_causing	Passenger	Neutral	passenger	No	No	No	No	Not tested
CS1-C	Carcinoma	ELMO3	p.D191N	Private	16	67234263	G	A	Missense_Mutation	334	0.25748503	No	1	Clonal	Disease_causing	Passenger	Neutral	passenger	No	No	No	No	Not tested
CS1-S	Sarcoma	ELMOD3	p.V298I	Private	2	85617337	G	A	Missense_Mutation	308	0.438311688	No	1	Clonal	Disease_causing	Passenger	Neutral	passenger	No	No	No	No	Not tested
CS1-S	Sarcoma	EMC2	p.A216V	Private	8	109489066	C	T	Missense_Mutation	347	0.265129683	No	1	Clonal	Disease_causing	Passenger	Neutral	passenger	No	No	No	No	Not tested
CS1-C	Carcinoma	EML4	p.T600A	Private	2	42530485	A	G	Missense_Mutation	297	0.097643098	No	1	Clonal	Disease_causing	Passenger	Neutral	passenger	No	Yes	No	No	Not tested
CS1-C	Carcinoma	EML6	p.A1772T	Private	2	55191278	G	A	Missense_Mutation	413	0.033898305	No	0.358	Subclonal	Disease_causing	Passenger	Neutral	passenger	No	No	No	No	Not tested
CS1-C	Carcinoma	EMX2	p.S190R	Private	10	119305306	C	A	Missense_Mutation	120	0.083333333	No	0.797	Clonal	Disease_causing	Passenger	.	passenger	No	No	No	No	Not tested
CS1-C	Carcinoma	ENPEP	p.L134P	Private	4	111397971	T	C	Missense_Mutation	276	0.032608696	No	0.312	Subclonal	Disease_causing	Driver	Neutral	likely_pathogenic	No	No	No	No	Not tested
CS1-C	Carcinoma	ENPP5	p.R166S	Private	6	46135502	T	A	Missense_Mutation	274	0.197080292	No	1	Clonal	Disease_causing	Passenger	Neutral	passenger	No	No	No	No	Not tested
CS1-S	Sarcoma	EPB41	p.K690Rfs*43	Private	1	29391550	GA	G	Frame_Shift_Del	312	0.352564103	No	1	Clonal	.	.	.	.	No	No	No	No	Not tested
CS1-C	Carcinoma	EPG5	p.A1862T	Private	18	43460123	C	T	Missense_Mutation	174	0.074712644	No	0.714	Subclonal	Neutral	Passenger	Neutral	passenger	No	No	No	No	Not tested
CS1-S	Sarcoma	EPHA5	p.S459F	Private	4	66356121	G	A	Missense_Mutation	433	0.071593533	No	0.252	Subclonal	Disease_causing	Passenger	Neutral	passenger	No	No	No	No	Not tested
CS1-S	Sarcoma	EPHB1	p.P420Hfs*28	Private	3	134851847	TC	T	Frame_Shift_Del	186	0.38172043	No	1	Clonal	.	.	.	.	No	No	No	No	Not tested
CS1-S	Sarcoma	EPHB4	p.X707_splice	Private	7	100405204	T	C	Splice_Site	186	0.155913978	No	0.549	Subclonal	Disease_causing	.	.	passenger	No	No	No	No	Not tested
CS1-C	Carcinoma	EPM2AIP1	p.E51K	Private	3	37034418	C	T	Missense_Mutation	227	0.273127753	No	1	Clonal	Disease_causing	Passenger	Neutral	passenger	No	No	No	No	Not tested
CS1-C	Carcinoma	EPSTI1	p.R19H	Private	13	43566246	C	T	Missense_Mutation	243	0.218106996	No	1	Clonal	Neutral	Passenger	.	passenger	No	No	No	No	Not tested
CS1-S	Sarcoma	EPSTI1	p.E263G	Private	13	43474506	T	C	Missense_Mutation	359	0.155988858	No	0.549	Subclonal	Neutral	Passenger	Neutral	passenger	No	No	No	No	Not tested
CS1-S	Sarcoma	ERGIC3	p.V229I	Private	20	34136618	G	A	Missense_Mutation	160	0.075	No	0.264	Subclonal	Disease_causing	Passenger	Neutral	passenger	No	No	No	No	Not tested
CS1-C	Carcinoma	ERLIN2	p.I116V	Private	8	37602136	A	G	Missense_Mutation	471	0.180467091	No	1	Clonal	Disease_causing	Driver	Disease_causing	likely_pathogenic	No	No	No	No	Not tested
CS1-S	Sarcoma	ESPL1	p.E1811G	Private	12	53684692	A	G	Missense_Mutation	128	0.0625	No	0.22	Subclonal	Neutral	Passenger	Neutral	passenger	No	No	No	No	Not tested
CS1-C	Carcinoma	ETV3	p.G16W	Private	1	157106099	C	A	Missense_Mutation	178	0.056179775	No	0.593	Subclonal	Disease_causing	Passenger	Neutral	passenger	No	No	No	No	Not tested
CS1-S	Sarcoma	EXOC3	p.R277H	Private	5	453950	G	A	Missense_Mutation	422	0.431279621	No	1	Clonal	Disease_causing	Passenger	Neutral	passenger	No	No	No	No	Not tested
CS1-S	Sarcoma	EXOSC4	p.R237H	Private	8	145135476	G	A	Missense_Mutation	268	0.171641791	No	0.776	Subclonal	Disease_causing	Passenger	Neutral	passenger	No	No	No	No	Not tested
CS1-C	Carcinoma	EYA2	p.A189T	Private	20	45702878	G	A	Missense_Mutation	380	0.039473684	No	0.377	Subclonal	Disease_causing	Passenger	Neutral	passenger	No	No	No	No	Not tested
CS1-S	Sarcoma	F3	p.S117N	Private	1	95001583	C	T	Missense_Mutation	167	0.053892216	No	0.19	Subclonal	Neutral	Passenger	Neutral	passenger	No	No	No	No	Not tested
CS1-C	Carcinoma	FAH	p.D233N	Shared	15	80464581	G	A	Missense_Mutation	267	0.34082397	No	1	Clonal	Disease_causing	Passenger	Disease_causing	likely_pathogenic	No	No	No	No	Not tested
CS1-S	Sarcoma	FAH	p.D233N	Shared	15	80464581	G	A	Missense_Mutation	320	0.421875	No	1	Clonal	Disease_causing	Passenger	Disease_causing	likely_pathogenic	No	No	No	No	Not tested
CS1-C	Carcinoma	FAM120C	p.T1089A	Private	X	54099492	T	C	Missense_Mutation	256	0.04296875	No	0.411	Subclonal	Disease_causing	Passenger	Neutral	passenger	No	No	No	No	Not tested
CS1-C	Carcinoma	FAM127C	p.D36N	Private	X	134156384	C	T	Missense_Mutation	316	0.041139241	No	0.393	Subclonal	Neutral	Passenger	Neutral	passenger	No	No	No	No	Not tested
CS1-C	Carcinoma	FAM186B	p.P432S	Private	12	49994129	G	A	Missense_Mutation	401	0.216957606	No	1	Clonal	Neutral	Passenger	Neutral	passenger	No	No	No	No	Not tested
CS1-S	Sarcoma	FAM193A	p.V231Sfs*81	Private	4	2661592	AC	A	Frame_Shift_Del	598	0.37458194	No	1	Clonal	.	.	.	.	No	No	No	No	Not tested
CS1-C	Carcinoma	FAM19A5	p.R63Q	Private	22	49042484	G	A	Missense_Mutation	200	0.225	No	1	Clonal	Disease_causing	Passenger	.	passenger	No	No	No	No	Not tested
CS1-C	Carcinoma	FAM20A	p.H438Y	Private	17	66535527	G	A	Missense_Mutation	273	0.058608059	No	0.56	Subclonal	Disease_causing	Passenger	.	passenger	No	No	No	No	Not tested
CS1-C	Carcinoma	FAM214B	p.V444M	Private	9	35105812	C	T	Missense_Mutation	169	0.059171598	No	0.566	Subclonal	Disease_causing	Passenger	.	passenger	No	No	No	No	Not tested
CS1-S	Sarcoma	FAM219A	p.K165Sfs*3	Private	9	34401027	TG	T	Frame_Shift_Del	633	0.134281201	No	0.473	Subclonal	.	.	.	.	No	No	No	No	Not tested
CS1-C	Carcinoma	FAM47A	p.R236H	Private	X	34149689	C	T	Missense_Mutation	302	0.215231788	No	1	Clonal	Neutral	Passenger	Neutral	passenger	No	No	No	No	Not tested
CS1-S	Sarcoma	FAM71E2	p.R66W	Private	19	55874055	G	A	Missense_Mutation	428	0.051401869	No	0.181	Subclonal	Neutral	Passenger	Neutral	passenger	No	No	No	No	Not tested
CS1-S	Sarcoma	FAM71E2	p.P588L	Private	19	55870473	G	A	Missense_Mutation	457	0.266958425	No	0.939	Clonal	Neutral	Passenger	Neutral	passenger	No	No	No	No	Not tested
CS1-C	Carcinoma	FAM83E	p.E420Kfs*121	Private	19	49104544	TC	T	Frame_Shift_Del	199	0.095477387	No	0.913	Clonal	.	.	.	.	No	No	No	No	Not tested
CS1-C	Carcinoma	FANCB	p.T553A	Private	X	14863248	T	C	Missense_Mutation	198	0.04040404	No	0.386	Subclonal	Neutral	Passenger	.	passenger	No	No	No	No	Not tested
CS1-S	Sarcoma	FANCM	p.V1336Lfs*2	Private	14	45645954	CA	C	Frame_Shift_Del	364	0.285714286	No	1	Clonal	.	.	.	.	No	No	No	No	Not tested
CS1-C	Carcinoma	FASN	p.T1480A	Private	17	80042801	T	C	Missense_Mutation	217	0.046082949	No	0.441	Subclonal	Neutral	Passenger	Neutral	passenger	No	No	No	No	Not tested
CS1-C	Carcinoma	FASTKD2	p.R652Efs*6	Private	2	207655346	AC	A	Frame_Shift_Del	232	0.193965517	No	1	Clonal	.	.	.	.	No	No	No	No	Not tested
CS1-C	Carcinoma	FAT2	p.P2377Qfs*41	Private	5	150923557	TG	T	Frame_Shift_Del	288	0.201388889	No	1	Clonal	.	.	.	.	No	No	No	No	Not tested

CS1-C	Carcinoma	FBF1	p.V542Sfs*60	Private	17	73917329	AC	A	Frame_Shift_Del	131	0.229007634	No	1	Clonal	.	.	.	.	No	No	No	No	Not tested
CS1-S	Sarcoma	FBLN2	p.G227A	Private	3	13612535	G	C	Missense_Mutation	104	0.221153846	No	0.778	Subclonal	Neutral	Passenger	Neutral	passenger	No	No	No	No	Not tested
CS1-C	Carcinoma	FBN1	p.D1363E	Private	15	48766573	A	C	Missense_Mutation	207	0.101449275	No	0.97	Clonal	Disease_causing	Passenger	Disease_causing	likely_pathogenic	No	No	No	No	Not tested
CS1-S	Sarcoma	FBXL13	p.A38V	Private	7	102669151	G	A	Missense_Mutation	258	0.360465116	No	1	Clonal	Neutral	Passenger	Neutral	passenger	No	No	No	No	Not tested
CS1-S	Sarcoma	FBXL15	p.L200P	Private	10	104181935	T	C	Missense_Mutation	297	0.134680135	No	0.474	Subclonal	Disease_causing	Passenger	Neutral	passenger	No	No	No	No	Not tested
CS1-C	Carcinoma	FBXL19	p.L381Yfs*86	Private	16	30941675	TG	T	Frame_Shift_Del	161	0.211180124	No	1	Clonal	.	.	.	.	No	No	No	No	Not tested
CS1-S	Sarcoma	FCHSD1	p.P568H	Private	5	141023945	G	T	Missense_Mutation	449	0.158129176	No	0.556	Subclonal	Disease_causing	Passenger	Neutral	passenger	No	No	No	No	Not tested
CS1-S	Sarcoma	FCRL1	p.X11_splice	Private	1	157776913	C	T	Splice_Site	461	0.284164859	No	1	Clonal	Disease_causing	.	.	passenger	No	No	No	No	Not tested
CS1-C	Carcinoma	FGD6	p.A197T	Private	12	95604471	C	T	Missense_Mutation	245	0.220408163	No	1	Clonal	Neutral	Passenger	Neutral	passenger	No	No	No	No	Not tested
CS1-S	Sarcoma	FGF18	p.T41M	Private	5	170863149	C	T	Missense_Mutation	122	0.073770492	No	0.26	Subclonal	Disease_causing	Passenger	Disease_causing	likely_pathogenic	No	No	No	No	Not tested
CS1-C	Carcinoma	FHDC1	p.A326T	Private	4	153884229	G	A	Missense_Mutation	267	0.037453184	No	0.358	Subclonal	Disease_causing	Passenger	Neutral	passenger	No	No	No	No	Not tested
CS1-C	Carcinoma	FHOD1	p.A269V	Private	16	67271421	G	A	Missense_Mutation	258	0.034883721	No	0.334	Subclonal	Neutral	Passenger	Neutral	passenger	No	No	No	No	Not tested
CS1-S	Sarcoma	FHOD3	p.L211P	Private	18	34174775	T	C	Missense_Mutation	123	0.422764228	No	1	Clonal	Disease_causing	Driver	Neutral	likely_pathogenic	No	No	No	No	Not tested
CS1-S	Sarcoma	FIBP	p.R349H	Private	11	65651494	C	T	Missense_Mutation	153	0.37254902	No	1	Clonal	Disease_causing	Passenger	Neutral	passenger	No	No	No	No	Not tested
CS1-C	Carcinoma	FIGN	p.S603G	Private	2	164466535	T	C	Missense_Mutation	230	0.043478261	No	0.416	Subclonal	Disease_causing	Passenger	Disease_causing	likely_pathogenic	No	No	No	No	Not tested
CS1-S	Sarcoma	FKBP10	p.G278Afs*20	Private	17	39975558	TC	T	Frame_Shift_Del	268	0.257462687	No	0.906	Clonal	.	.	.	.	No	No	No	No	Not tested
CS1-C	Carcinoma	FKRP	p.R203C	Private	19	47259314	C	T	Missense_Mutation	243	0.041152263	No	0.393	Subclonal	Disease_causing	Passenger	Disease_causing	likely_pathogenic	No	No	No	No	Not tested
CS1-S	Sarcoma	FLNC	p.R526L	Private	7	128480629	G	T	Missense_Mutation	467	0.391862955	No	1	Clonal	Neutral	Passenger	Disease_causing	passenger	No	No	No	No	Not tested
CS1-C	Carcinoma	FLT1	p.W186C	Private	13	29008313	C	A	Missense_Mutation	320	0.03125	No	0.299	Subclonal	Disease_causing	Passenger	Neutral	passenger	No	No	No	No	Not tested
CS1-C	Carcinoma	FMR1	p.N412D	Private	X	147022140	A	G	Missense_Mutation	135	0.037037037	No	0.354	Subclonal	Disease_causing	Passenger	Neutral	passenger	No	No	No	No	Not tested
CS1-C	Carcinoma	FNDC1	p.R856*	Shared	6	159654110	C	T	Nonsense_Mutation	329	0.376899696	No	1	Clonal	Neutral	.	.	passenger	No	No	No	No	Not tested
CS1-C	Carcinoma	FNDC1	p.R609S	Private	6	159653369	C	A	Missense_Mutation	282	0.085106383	No	0.814	Clonal	Neutral	Passenger	Neutral	passenger	No	No	No	No	Not tested
CS1-S	Sarcoma	FNDC1	p.R856*	Shared	6	159654110	C	T	Nonsense_Mutation	509	0.406679764	No	1	Clonal	Neutral	.	.	passenger	No	No	No	No	Not tested
CS1-S	Sarcoma	FOXA2	p.Q122Afs*123	Private	20	22563516	G	GC	Frame_Shift_Ins	215	0.130232558	No	0.458	Subclonal	.	.	.	.	No	No	Yes	No	Not tested
CS1-S	Sarcoma	FOXG1	p.V364M	Private	14	29237575	G	A	Missense_Mutation	337	0.347181009	No	1	Clonal	Disease_causing	Passenger	Disease_causing	likely_pathogenic	No	No	No	No	Not tested
CS1-C	Carcinoma	FOXL2	p.G247V	Private	3	138664825	C	A	Missense_Mutation	223	0.094170404	No	0.9	Clonal	Disease_causing	Passenger	Disease_causing	likely_pathogenic	No	Yes	No	No	Not tested
CS1-C	Carcinoma	FOXM1	p.A403T	Private	12	2973545	C	T	Missense_Mutation	203	0.177339901	No	1	Clonal	Neutral	Passenger	Disease_causing	passenger	No	No	No	No	Not tested
CS1-C	Carcinoma	FOXN2	p.A18V	Private	2	48573406	C	T	Missense_Mutation	406	0.0591133	No	0.624	Subclonal	Disease_causing	Passenger	Disease_causing	likely_pathogenic	No	No	No	No	Not tested
CS1-C	Carcinoma	FRAS1	p.E2419Rfs*19	Private	4	79387577	A	AG	Frame_Shift_Ins	235	0.208510638	No	1	Clonal	.	.	.	.	No	No	No	No	Not tested
CS1-C	Carcinoma	FSIP2	p.S6308*	Shared	2	186672689	C	A	Nonsense_Mutation	278	0.417266187	No	1	Clonal	Neutral	.	.	passenger	No	No	No	No	Not tested
CS1-S	Sarcoma	FSIP2	p.S6308*	Shared	2	186672689	C	A	Nonsense_Mutation	471	0.380042463	No	1	Clonal	Neutral	.	.	passenger	No	No	No	No	Not tested
CS1-C	Carcinoma	FUBP1	p.X643_splice	Private	1	78414461	T	C	Splice_Site	299	0.043478261	No	0.416	Subclonal	Disease_causing	.	.	passenger	No	No	No	No	Not tested
CS1-S	Sarcoma	FUBP1	p.S11Lfs*43	Private	1	78444658	AG	A	Frame_Shift_Del	290	0.213793103	No	0.752	Subclonal	.	.	.	.	No	Yes	No	No	Not tested
CS1-S	Sarcoma	FUBP3	p.Q527Tfs*91	Private	9	133510115	C	CA	Frame_Shift_Ins	216	0.148148148	No	0.521	Subclonal	.	.	.	.	No	No	No	No	Not tested
CS1-C	Carcinoma	FUT4	p.A469T	Private	11	94278704	G	A	Missense_Mutation	560	0.030357143	No	0.29	Subclonal	Disease_causing	Passenger	Neutral	passenger	No	No	No	No	Not tested
CS1-C	Carcinoma	FXR2	p.A541Sfs*14	Private	17	7496121	T	TG	Frame_Shift_Ins	268	0.126865672	No	1	Clonal	.	.	.	.	No	No	No	No	Not tested
CS1-C	Carcinoma	FZD10	p.G554S	Shared	12	130649147	G	A	Missense_Mutation	296	0.398648649	No	1	Clonal	Neutral	Passenger	Neutral	passenger	No	No	No	No	Not tested
CS1-S	Sarcoma	FZD10	p.G554S	Shared	12	130649147	G	A	Missense_Mutation	430	0.448837209	No	1	Clonal	Neutral	Passenger	Neutral	passenger	No	No	No	No	Not tested
CS1-S	Sarcoma	FZD9	p.R428P	Private	7	72849620	G	C	Missense_Mutation	508	0.141732283	No	0.499	Subclonal	Disease_causing	Passenger	Disease_causing	likely_pathogenic	No	No	No	No	Not tested
CS1-S	Sarcoma	FZR1	p.W57G	Private	19	3525965	T	G	Missense_Mutation	400	0.1075	No	0.378	Subclonal	Disease_causing	Passenger	Neutral	passenger	No	No	No	No	Not tested
CS1-C	Carcinoma	GABRA4	p.Y79C	Private	4	46981085	T	C	Missense_Mutation	220	0.031818182	No	0.304	Subclonal	Disease_causing	Passenger	Neutral	passenger	No	No	No	No	Not tested
CS1-S	Sarcoma	GABRA4	p.M174V	Private	4	46979135	T	C	Missense_Mutation	293	0.102389078	No	0.36	Subclonal	Disease_causing	Passenger	Neutral	passenger	No	No	No	No	Not tested
CS1-C	Carcinoma	GABRR3	p.I443M	Private	3	97705602	A	C	Missense_Mutation	217	0.036866359	No	0.352	Subclonal	.	Passenger	.	passenger	No	No	No	No	Not tested
CS1-C	Carcinoma	GALNS	p.P420T	Private	16	88889103	G	T	Missense_Mutation	216	0.236111111	No	1	Clonal	Disease_causing	Passenger	Disease_causing	likely_pathogenic	No	No	No	No	Not tested
CS1-C	Carcinoma	GAS6	p.I403Nfs*28	Private	13	114531620	A	AT	Frame_Shift_Ins	323	0.19504644	No	1	Clonal	.	.	.	.	No	No	No	No	Not tested
CS1-S	Sarcoma	GBP5	p.K232Efs*6	Private	1	89732204	C	CT	Frame_Shift_Ins	478	0.135983264	No	0.479	Subclonal	.	.	.	.	No	No	No	No	Not tested
CS1-S	Sarcoma	GCM1	p.P319Lfs*2	Private	6	52993360	GA	G	Frame_Shift_Del	497	0.273641851	No	0.963	Clonal	.	.	.	.	No	No	No	No	Not tested
CS1-C	Carcinoma	GFRA1	p.P231S	Private	10	117884811	G	A	Missense_Mutation	215	0.065116279	No	0.623	Subclonal	Disease_causing	Passenger	Neutral	passenger	No	No	No	No	Not tested
CS1-S	Sarcoma	GGACT	p.Y68C	Private	13	101184643	T	C	Missense_Mutation	706	0.281869688	No	0.992	Clonal	Disease_causing	Passenger	Neutral	passenger	No	No	No	No	Not tested
CS1-C	Carcinoma	GGN	p.P195L	Private	19	38877318	G	A	Missense_Mutation	190	0.105263158	No	1	Clonal	Neutral	Passenger	.	passenger	No	No	No	No	Not tested
CS1-C	Carcinoma	GIPC3	p.A229Gfs*10	Private	19	3589526	T	TG	Frame_Shift_Ins	148	0.162162162	No	1	Clonal	.	.	.	.	No	No	No	No	Not tested
CS1-S	Sarcoma	GIPC3	p.A229Lfs*55	Private	19	3589526	TG	T	Frame_Shift_Del	196	0.219387755	No	0.772	Subclonal	.	.	.	.	No	No	No	No	Not tested
CS1-C	Carcinoma	GIT1	p.A342T	Private	17	27904250	C	T	Missense_Mutation	193	0.046632124	No	0.446	Subclonal	Disease_causing	Passenger	Neutral	passenger	No	No	No	No	Not tested
CS1-C	Carcinoma	GJA5	p.V323M	Private	1	147230380	C	T	Missense_Mutation	451	0.181818182	No	1	Clonal	Neutral	Passenger	Disease_causing	passenger	No	No	No	No	Not tested
CS1-S	Sarcoma	GJC2	p.P305Rfs*166	Private	1	228346367	GC	G	Frame_Shift_Del	315	0.142857143	No	0.646	Subclonal	.	.	.	.	No	No	No	No	Not tested
CS1-S	Sarcoma	GK	p.F175Lfs*12	Private	X	30712622	CT	C	Frame_Shift_Del	170	0.270588235	No	0.952	Clonal	.	.	.	.	No	No	No	No	Not tested
CS1-C	Carcinoma	GLI3	p.R667*	Private	7	42012040	G	A	Nonsense_Mutation	291	0.034364261	No	0.329	Subclonal	Disease_causing	.	.	likely_pathogenic	No	No	No	No	Not tested
CS1-S	Sarcoma	GLI3	p.A697G	Private	7	42011949	G	C	Missense_Mutation	185	0.389189189	No	1	Clonal	Disease_causing	Passenger	Neutral	passenger	No	No	No	No	Not tested
CS1-C	Carcinoma	GLIS3	p.P166H	Private	9	4125833	G	T	Missense_Mutation	182	0.252747253	No	1	Clonal	Disease_causing	Passenger	Neutral	passenger	No	No	No	No	Not tested
CS1-S	Sarcoma	GLRA1	p.A134G	Private	5	151239421	G	C	Missense_Mutation	257	0.412451362	No	1	Clonal	Disease_causing	Passenger	Neutral	passenger	No	No	No	No	Not tested
CS1-C	Carcinoma	GLTSCR1	p.P937Hfs*10	Shared	19	48197890	AC	A	Frame_Shift_Del	149	0.127516779	No	1	Clonal	.	.	.	.	No	No	No	No	Not tested
CS1-S	Sarcoma	GLTSCR1	p.P937Hfs*10	Shared	19	48197890	AC	A	Frame_Shift_Del	244	0.18852459	No	0.663	Clonal	.	.	.	.	No	No	No	No	Not tested
CS1-C	Carcinoma	GLUD1	p.D427N	Private	10	88819030	C	T	Missense_Mutation	238	0.088235294	No	0.844	Clonal	Disease_causing	Passenger	Disease_causing	likely_pathogenic	No	No	No	No	Not tested
CS1-S	Sarcoma	GLYR1	p.R312S	Private	16	4864621	G	T	Missense_Mutation	144	0.25	No	0.88	Subclonal	Disease_causing	Passenger	Neutral	passenger	No	No	No	No	Not tested
CS1-S	Sarcoma	GMEB1	p.G503D	Private	1	29041071	G	A	Missense_Mutation	418	0.174641148	No	0.615	Subclonal	Neutral	Passenger	Neutral	passenger	No	No	No	No	Not tested
CS1-C	Carcinoma	GNAI2	p.R314H	Private	3	50294995	G	A	Missense_Mutation	117	0.196581197	No	1	Clonal	Disease_causing	Passenger	Disease_causing	likely_pathogenic	No	No	No	No	Not tested
CS1-S	Sarcoma	GNB3	p.N119del	Private	12	6952611	GACA	G	In_Frame_Del	424	0.113207547	No	0.398	Subclonal	.	.	.	.	No	No	No	No	Not tested
CS1-C	Carcinoma	GNL3L	p.R320H	Shared	X	54578096	G	A	Missense_Mutation	265	0.343396226	No	1	Clonal	Disease_causing	Passenger	Neutral	passenger	No	No	No	No	Not tested
CS1-S	Sarcoma	GNL3L	p.R320H	Shared	X	54578096	G	A	Missense_Mutation	356	0.415730337	No	1	Clonal	Disease_causing	Passenger	Neutral	passenger	No	No	No	No	Not tested
CS1-S	Sarcoma	GOLGA5	p.M486I	Private	14	93282733	G	A	Missense_Mutation	257	0.190661479	No	0.671	Subclonal	Disease_causing	Passenger	Neutral	passenger	No	Yes	No	No	Not tested
CS1-S	Sarcoma	GOSR1	p.L104P	Private	17	28811751	T	C	Missense_Mutation	383	0.279373368	No	0.983	Clonal	Disease_causing	Passenger	.	passenger	No	No	No	No	Not tested
CS1-S	Sarcoma	GP1BA	p.T494Nfs*4	Private	17	4837372	C	CA	Frame_Shift_Ins	571	0.248686515	No	0.875	Subclonal	.	.	.	.	No	No	No	No	Not tested
CS1-S	Sarcoma	GPATCH2L	p.F471L	Private	14	76668158	T	A	Missense_Mutation	188	0.127659574	No	0.449	Subclonal	Disease_causing	Passenger	Neutral	passenger	No	No	No	No	Not tested
CS1-C	Carcinoma	GPR149	p.A283T	Private	3	154146558	C	T	Missense_Mutation	168	0.202380952	No	1	Clonal	Neutral	Passenger	Neutral	passenger	No	No	No	No	Not tested
CS1-C	Carcinoma	GPR153	p.R205H	Private	1	6313950	C	T	Missense_Mutation	94	0.063829787	No	0.61	Subclonal	Neutral	Passenger	Neutral	passenger	No	No	No	No	Not tested
CS1-S	Sarcoma	GPR156	p.C391R	Private	3	119887153	A	G	Missense_Mutation	295	0.247457627	No	0.871	Subclonal	Disease_causing	Passenger	Neutral	passenger	No	No	No	No	Not tested
CS1-C	Carcinoma	GPR26	p.R115Q	Private	10	125426267	G	A	Missense_Mutation	240	0.033333333	No	0.319	Subclonal	Disease_causing	Passenger	Neutral	passenger	No	No	No	No	Not tested
CS1-C	Carcinoma	GPR4	p.S270N	Private	19	46094316	C	T	Missense_Mutation	213	0.154929577	No	1	Clonal	Neutral	Passenger	Neutral	passenger	No	No	No	No	Not tested
CS1-S	Sarcoma	GPR4	p.A62T	Private	19	46094941	C	T	Missense_Mutation	261	0.38697318	No	1	Clonal	Disease_causing	Passenger	Neutral	passenger	No	No	No	No	Not tested
CS1-S	Sarcoma	GPT	p.A71T	Private	8	145730035	G	A	Missense_Mutation	755	0.568211921	No	1	Clonal	Disease_causing	Passenger	Neutral	passenger	No	No	No	No	Not tested
CS1-S	Sarcoma	GREB1L	p.Y1829H	Private	18	19100661	T	C	Missense_Mutation	183	0.453551913	No	1	Clonal	Disease_causing	Driver	Neutral	likely_pathogenic	No	No	No	No	Not tested
CS1-C	Carcinoma	GRIK2	p.N849Tfs*14	Private	6	102503431	CA	C	Frame_Shift_Del	141	0.141843972	No	1	Clonal	.	.	.	.	No	No	No	No	Not tested
CS1-S	Sarcoma	GRIN1	p.S159N	Private	9	140040260	G	A	Missense_Mutation	377	0.193633952	No	0.681	Subclonal	Neutral	Passenger	Neutral	passenger	No	No	No	No	Not tested
CS1-C	Carcinoma	GRIN2A	p.A33Rfs*105	Private	16	10274172	C	CG	Frame_Shift_Ins	342	0.192982456	No	1	Clonal	.	.	.	.	No	Yes	No	No	Not tested
CS1-C	Carcinoma	GRIN2B	p.N697D	Private	12	13724820	T	C	Missense_Mutation	253	0.166007905	No	1	Clonal	Disease_causing	Passenger	Neutral	passenger	No	No	No	No	Not tested
CS1-S	Sarcoma	GRIN2D	p.G989R	Private	19	48946148	G	A	Missense_Mutation	74	0.27027027	No	0.951	Clonal	Neutral	Passenger	Neutral	passenger	No	No	No	No	Not tested
CS1-C	Carcinoma	GRM8	p.D45N	Private	7	126883126	C	T	Missense_Mutation	377	0.045092838	No	0.431	Subclonal	Disease_causing	Passenger	Neutral	passenger	No	No	No	No	Not tested
CS1-S	Sarcoma	GRM8	p.G803Wfs*73	Private	7	126173029	C	CA	Frame_Shift_Ins	185	0.378378378	No	1	Clonal	.	.	.	.	No	No	No	No	Not tested
CS1-S	Sarcoma	GSE1	p.S47N	Private	16	85667652	G	A	Missense_Mutation	312	0.217948718	No	0.767	Subclonal	Disease_causing	Passenger	Neutral	passenger	No	No	No	No	Not tested
CS1-S	Sarcoma	GTF3C2	p.G395Dfs*29	Private	2	27559235	TC	T	Frame_Shift_Del	169	0.136094675	No	0.479	Subclonal	.	.	.	.	No	No	No	No	Not tested
CS1-C	Carcinoma	GUCY2C	p.W187R	Private	12	14836028	A	G	Missense_Mutation	213	0.220657277	No	1	Clonal	Disease_causing	Passenger	Disease_causing	likely_pathogenic	No	No	No	No	Not tested
CS1-S	Sarcoma	GXYLT1	p.L223Yfs*11	Private	12	42499815	TA	T	Frame_Shift_Del	223	0.192825112	No	0.679	Subclonal	.	.	.	.	No	No	No	No	Not tested
CS1-S	Sarcoma	HAMP	p.C81W	Private	19	35775933	C	G	Missense_Mutation	426	0.363849765	No	1	Clonal	Disease_causing	Passenger	Disease_causing	likely_pathogenic	No	No	No	No	Not tested
CS1-C	Carcinoma	HAP1	p.R202Gfs*49	Private	17	39888590	CCT	C	Frame_Shift_Del	128	0.1796875	No	1	Clonal	.	.	.	.	No	No	No	No	Not tested
CS1-S	Sarcoma	HAP1	p.E500Kfs*27	Private	17	39881314	TC	T	Frame_Shift_Del	531	0.237288136	No	0.835	Subclonal	.	.	.	.	No	No	No	No	Not tested
CS1-C	Carcinoma	HAPLN4	p.Y221H	Private	19	19369488	A	G	Missense_Mutation	202	0.03960396	No	0.379	Subclonal	Disease_causing	Driver	Neutral	likely_pathogenic	No	No	No	No	Not tested
CS1-S	Sarcoma	HARS	p.R73C	Private	5	140062768	G	A	Missense_Mutation	220	0.472727273	No	1	Clonal	Disease_causing	Passenger	Neutral	passenger	No	No	No	No	Not tested
CS1-S	Sarcoma	HARS2	p.R389*	Private	5	140076959	C	T	Nonsense_Mutation	349	0.23495702	No	0.827	Subclonal	Neutral	.	.	passenger	No	No	No	No	Not tested
CS1-S	Sarcoma	HBP1	p.Y103H	Private	7	106822955	T	C	Missense_Mutation	282	0.241134752	No	0.849	Subclonal	Disease_causing	Passenger	Disease_causing	likely_pathogenic	No	No	No	No	Not tested
CS1-S	Sarcoma	HCFC1	p.Q1402Pfs*22	Private	X	153219645	T	TG	Frame_Shift_Ins	224	0.138392857	No	0.487	Subclonal	.	.	.	.	No	No	No	No	Not tested
CS1-S	Sarcoma	HDAC11	p.W133L	Private	3	13540041	G	T	Missense_Mutation	178	0.04494382	No	0.158	Subclonal	Disease_causing	Passenger	Neutral	passenger	No	No	No	No	Not tested
CS1-C	Carcinoma	HDAC2	p.A133T	Private	6	114277277	C	T	Missense_Mutation	329	0.170212766	No	1	Clonal	Disease_causing	Passenger	Neutral	passenger	No	No	No	No	Not tested
CS1-S	Sarcoma	HDAC4	p.V1064M	Private	2	239975181	C	T	Missense_Mutation	98	0.244897959	No	0.862	Subclonal	Disease_causing	Passenger	Neutral	passenger	No	No	No	No	Not tested
CS1-C	Carcinoma	HDAC6	p.A48V	Private	X	48661327	C	T	Missense_Mutation	298	0.067114094	No	0.642	Subclonal	Neutral	Passenger	Neutral	passenger	No	No	No	No	Not tested
CS1-S	Sarcoma	HDAC7	p.M808Wfs*12	Private	12	48181526	TG	T	Frame_Shift_Del	81	0.197530864	No	0.695	Subclonal	.	.	.	.	No	No	No	No	Not tested
CS1-S	Sarcoma	HDGFRP2	p.R356W	Private	19	4494314	C	T	Missense_Mutation	137	0.189781022	No	0.668	Subclonal	Neutral	Passenger	Neutral	passenger	No	No	No	No	Not tested
CS1-C	Carcinoma	HECA	p.R152C	Private	6	139487603	C	T	Missense_Mutation	342	0.032163743	No	0.308	Subclonal	Disease_causing	Passenger	.	passenger	No	No	No	No	Not tested
CS1-C	Carcinoma	HECA	p.G256C	Private	6	139487915	G	T	Missense_Mutation	268	0.097014925	No	0.928	Clonal	Neutral	Passenger	.	passenger	No	No	No	No	Not tested
CS1-C	Carcinoma	HECTD4	p.C1280Y	Private	12	112685326	C	T	Missense_Mutation	217	0.041474654	No	0.397	Subclonal	Disease_causing	Driver	Neutral	likely_pathogenic	No	No	No	No	Not tested
CS1-S	Sarcoma	HECTD4	p.S343F	Private	12	112720982	G	A	Missense_Mutation	130	0.046153846	No	0.162	Subclonal	Disease_causing	Driver	Neutral	likely_pathogenic	No	No	No	No	Not tested
CS1-C	Carcinoma	HECW2	p.I463N	Private	2	197184226	A	T	Missense_Mutation	424	0.051886792	No	0.496	Subclonal	Disease_causing	Passenger	Neutral	passenger	No	No	No	No	Not tested
CS1-S	Sarcoma	HELLS	p.E45Afs*13	Private	10	96306227	TAG	T	Frame_Shift_Del	390	0.223076923	No	0.785	Subclonal	.	.	.	.	No	No	No	No	Not tested
CS1-S	Sarcoma	HELZ2	p.R2206Pfs*42	Private	20	62193250	C	CG	Frame_Shift_Ins	211	0.355450237	No	1	Clonal	.	.	.	.	No	No	No	No	Not tested
CS1-C	Carcinoma	HERC1	p.V1879Lfs*26	Private	15	63986202	CT	C	Frame_Shift_Del	163	0.18404908	No	1	Clonal	.	.	.	.	No	No	No	No	Not tested
CS1-C	Carcinoma	HGS	p.R543W	Private	17	79663697	C	T	Missense_Mutation	209	0.04784689	No	0.457	Subclonal	Disease_causing	Passenger	Neutral	passenger	No	No	No	No	Not tested
CS1-C	Carcinoma	HIC1	p.A114T	Shared	17	1960267	G	A	Missense_Mutation	169	0.414201183	No	1	Clonal	Neutral	Passenger	Neutral	passenger	No	No	No	No	Not tested
CS1-S	Sarcoma	HIC1	p.A114T	Shared	17	1960267	G	A	Missense_Mutation	276	0.43115942	No	1	Clonal	Neutral	Passenger	Neutral	passenger	No	No	No	No	Not tested
CS1-S	Sarcoma	HID1	p.V58M	Private	17	72960622	C	T	Missense_Mutation	135	0.377777778	No	1	Clonal	Disease_causing	Passenger	.	passenger	No	No	No	No	Not tested
CS1-C	Carcinoma	HINFP	p.R324*	Private	11	119003668	C	T	Nonsense_Mutation	280	0.039285714	No	0.376	Subclonal	Neutral	.	.	passenger	No	No	No	No	Not tested
CS1-C	Carcinoma	HIPK1	p.A732T	Private	1	114506099	G	A	Missense_Mutation	166	0.253012048	No	1	Clonal	Disease_causing	Passenger	Neutral	passenger	No	No	No	No	Not tested
CS1-C	Carcinoma	HIST1H2AK	p.S2L	Shared	6	27806113	G	A	Missense_Mutation	215	0.311627907	No	1	Clonal	Neutral	Passenger	Disease_causing	passenger	No	No	No	No	Not tested
CS1-S	Sarcoma	HIST1H2AK	p.S2L	Shared	6	27806113	G	A	Missense_Mutation	284	0.383802817	No	1	Clonal	Neutral	Passenger	Disease_causing	passenger	No	No	No	No	Not tested
CS1-C	Carcinoma	HIST1H2BG	p.S65P	Private	6	26216679	A	G	Missense_Mutation	194	0.231958763	No	1	Clonal	Disease_causing	Passenger	Neutral	passenger	No	No	No	No	Not tested
CS1-S	Sarcoma	HIVEP1	p.S2533N	Private	6	12164135	G	A	Missense_Mutation	467	0.248394004	No	0.874	Subclonal	Disease_causing	Passenger	Neutral	passenger	No	No	No	No	Not tested
CS1-C	Carcinoma	HIVEP2	p.A2289T	Private	6	143074720	C	T	Missense_Mutation	235	0.238297872	No	1	Clonal	Neutral	Passenger	Neutral	passenger	No	No	No	No	Not tested
CS1-C	Carcinoma	HK1	p.F131Lfs*37	Private	10	71124539	CT	C	Frame_Shift_Del	245	0.204081633	No	1	Clonal	.	.	.	.	No	No	No	No	Not tested
CS1-C	Carcinoma	HK3	p.E13Kfs*4	Private	5	176323123	TC	T	Frame_Shift_Del	169	0.124260355	No	1	Clonal	.	.	.	.	No	No	No	No	Not tested
CS1-S	Sarcoma	HKDC1	p.M554K	Private	10	71010136	T	A	Missense_Mutation	256	0.45703125	No	1	Clonal	Disease_causing	Passenger	Disease_causing	likely_pathogenic	No	No	No	No	Not tested
CS1-C	Carcinoma	HMCN1	p.P5342S	Private	1	186147628	C	T	Missense_Mutation	325	0.030769231	No	0.325	Subclonal	Disease_causing	Passenger	Disease_causing	likely_pathogenic	No	No	No	No	Not tested
CS1-S	Sarcoma	HMX2	p.I84Sfs*87	Private	10	124908137	TC	T	Frame_Shift_Del	501	0.139720559	No	0.492	Subclonal	.	.	.	.	No	No	No	No	Not tested
CS1-S	Sarcoma	HNF4A	p.P21S	Private	20	43030073	C	T	Missense_Mutation	226	0.159292035	No	0.561	Subclonal	Disease_causing	Passenger	Disease_causing	likely_pathogenic	No	No	No	No	Not tested
CS1-C	Carcinoma	HNRNPAB	p.X126_splice	Private	5	177633013	T	C	Splice_Site	268	0.063432836	No	0.606	Subclonal	Disease_causing	.	.	passenger	No	No	No	No	Not tested
CS1-C	Carcinoma	HOOK1	p.G84*	Private	1	60297862	G	T	Nonsense_Mutation	239	0.221757322	No	1	Clonal	Neutral	.	.	passenger	No	No	No	No	Not tested
CS1-S	Sarcoma	HOXA2	p.F362Lfs*13	Private	7	27140389	TA	T	Frame_Shift_Del	612	0.117647059	No	0.414	Subclonal	.	.	.	.	No	No	No	No	Not tested
CS1-S	Sarcoma	HOXB1	p.P143Lfs*41	Private	17	46607838	AG	A	Frame_Shift_Del	348	0.373563218	No	1	Clonal	.	.	.	.	No	No	No	No	Not tested
CS1-S	Sarcoma	HOXD10	p.L14*	Private	2	176981596	CT	C	Frame_Shift_Del	530	0.375471698	No	1	Clonal	.	.	.	.	No	No	No	No	Not tested
CS1-S	Sarcoma	HPS1	p.M325Wfs*6	Private	10	100186986	TG	T	Frame_Shift_Del	197	0.116751269	No	0.411	Subclonal	.	.	.	.	No	No	No	No	Not tested
CS1-S	Sarcoma	HPS4	p.A238T	Private	22	26861512	C	T	Missense_Mutation	328	0.164634146	No	0.579	Subclonal	Neutral	Passenger	Neutral	passenger	No	No	No	No	Not tested
CS1-C	Carcinoma	HRG	p.H281R	Private	3	186394936	A	G	Missense_Mutation	422	0.232227488	No	1	Clonal	Neutral	Passenger	Neutral	passenger	No	No	No	No	Not tested
CS1-C	Carcinoma	HRG	p.H431Tfs*8	Private	3	186395383	GC	G	Frame_Shift_Del	326	0.214723926	No	1	Clonal	.	.	.	.	No	No	No	No	Not tested
CS1-S	Sarcoma	HS3ST1	p.V160A	Private	4	11401151	A	G	Missense_Mutation	546	0.042124542	No	0.148	Subclonal	Disease_causing	Passenger	Disease_causing	likely_pathogenic	No	No	No	No	Not tested
CS1-C	Carcinoma	HS3ST4	p.P22L	Private	16	25703803	C	T	Missense_Mutation	180	0.038888889	No	0.372	Subclonal	Neutral	Passenger	Neutral	passenger	No	No	No	No	Not tested
CS1-C	Carcinoma	HS6ST2	p.R51C	Private	X	132092480	G	A	Missense_Mutation	151	0.059602649	No	0.57	Subclonal	Disease_causing	Passenger	Neutral	passenger	No	No	No	No	Not tested
CS1-S	Sarcoma	HS6ST3	p.P78A	Private	13	96743348	C	G	Missense_Mutation	160	0.375	No	1	Clonal	Neutral	Passenger	Disease_causing	passenger	No	No	No	No	Not tested
CS1-C	Carcinoma	HSF1	p.L374F	Private	8	145535908	C	T	Missense_Mutation	66	0.136363636	No	1	Clonal	Disease_causing	Driver	.	likely_pathogenic	No	No	No	No	Not tested
CS1-S	Sarcoma	HSF1	p.R79Q	Private	8	145533150	G	A	Missense_Mutation	386	0.163212435	No	0.738	Subclonal	Disease_causing	Passenger	Disease_causing	likely_pathogenic	No	No	No	No	Not tested
CS1-S	Sarcoma	HSF4	p.G435V	Private	16	67203231	G	T	Missense_Mutation	347	0.268011527	No	0.943	Clonal	Disease_causing	Passenger	.	passenger	No	No	No	No	Not tested
CS1-S	Sarcoma	HSPA1L	p.R38C	Private	6	31779638	G	A	Missense_Mutation	100	0.19	No	0.669	Subclonal	Disease_causing	Passenger	Neutral	passenger	No	No	No	No	Not tested
CS1-S	Sarcoma	HSPA1L	p.Q24R	Private	6	31779679	T	C	Missense_Mutation	97	0.216494845	No	0.762	Subclonal	Disease_causing	Passenger	Neutral	passenger	No	No	No	No	Not tested
CS1-S	Sarcoma	HSPA4L	p.C38R	Private	4	128715236	T	C	Missense_Mutation	456	0.153508772	No	0.54	Subclonal	Disease_causing	Passenger	Neutral	passenger	No	No	No	No	Not tested
CS1-C	Carcinoma	HSPG2	p.H1559L	Private	1	22190657	T	A	Missense_Mutation	76	0.065789474	No	0.629	Subclonal	Disease_causing	Passenger	Neutral	passenger	No	No	No	No	Not tested
CS1-C	Carcinoma	HTR1D	p.R225W	Private	1	23520040	G	A	Missense_Mutation	161	0.055900621	No	0.534	Subclonal	Disease_causing	Passenger	Neutral	passenger	No	No	No	No	Not tested
CS1-C	Carcinoma	HTR1E	p.V146I	Private	6	87725488	G	A	Missense_Mutation	450	0.182222222	No	1	Clonal	Disease_causing	Passenger	Neutral	passenger	No	No	No	No	Not tested
CS1-C	Carcinoma	HUWE1	p.R2133L	Private	X	53596702	C	A	Missense_Mutation	151	0.052980132	No	0.507	Subclonal	Disease_causing	Driver	Neutral	likely_pathogenic	No	No	No	No	Not tested
CS1-S	Sarcoma	IARS2	p.M313I	Private	1	220276108	G	A	Missense_Mutation	203	0.113300493	No	0.512	Subclonal	Disease_causing	Passenger	Neutral	passenger	No	No	No	No	Not tested
CS1-C	Carcinoma	IBA57	p.V102M	Private	1	228353821	G	A	Missense_Mutation	321	0.046728972	No	0.494	Subclonal	Disease_causing	Passenger	Neutral	passenger	No	No	No	No	Not tested
CS1-C	Carcinoma	IBSP	p.Q317K	Private	4	88733057	C	A	Missense_Mutation	157	0.031847134	No	0.304	Subclonal	Disease_causing	Passenger	Neutral	passenger	No	No	No	No	Not tested
CS1-S	Sarcoma	IBTK	p.C764Mfs*6	Private	6	82922425	A	AT	Frame_Shift_Ins	286	0.377622378	No	1	Clonal	.	.	.	.	No	No	No	No	Not tested
CS1-C	Carcinoma	IDS	p.E27G	Private	X	148586588	T	C	Missense_Mutation	309	0.064724919	No	0.619	Subclonal	Neutral	Passenger	Disease_causing	passenger	No	No	No	No	Not tested
CS1-S	Sarcoma	IFFO1	p.R367Gfs*47	Private	12	6657973	GC	G	Frame_Shift_Del	476	0.12605042	No	0.444	Subclonal	.	.	.	.	No	No	No	No	Not tested
CS1-S	Sarcoma	IFLTD1	p.G379D	Private	12	25671782	C	T	Missense_Mutation	368	0.065217391	No	0.229	Subclonal	Neutral	Passenger	Neutral	passenger	No	No	No	No	Not tested
CS1-C	Carcinoma	IFNAR1	p.M411T	Private	21	34725152	T	C	Missense_Mutation	346	0.196531792	No	1	Clonal	Neutral	Passenger	Neutral	passenger	No	No	No	No	Not tested
CS1-C	Carcinoma	IFNGR1	p.I352T	Private	6	137519583	A	G	Missense_Mutation	343	0.049562682	No	0.474	Subclonal	Neutral	Passenger	Neutral	passenger	No	No	No	No	Not tested
CS1-C	Carcinoma	IFNGR2	p.Y69D	Private	21	34787326	T	G	Missense_Mutation	134	0.253731343	No	1	Clonal	Disease_causing	Passenger	Neutral	passenger	No	No	No	No	Not tested
CS1-S	Sarcoma	IFT81	p.K216R	Private	12	110573193	A	G	Missense_Mutation	323	0.250773994	No	0.882	Subclonal	Disease_causing	Passenger	Neutral	passenger	No	No	No	No	Not tested
CS1-S	Sarcoma	IGDCC4	p.V309I	Private	15	65689244	C	T	Missense_Mutation	189	0.386243386	No	1	Clonal	Disease_causing	Passenger	Neutral	passenger	No	No	No	No	Not tested
CS1-S	Sarcoma	IGF1R	p.P941S	Private	15	99472825	C	T	Missense_Mutation	304	0.309210526	No	1	Clonal	Disease_causing	Passenger	Neutral	passenger	No	No	No	No	Not tested
CS1-S	Sarcoma	IKBKE	p.V145I	Private	1	206649598	G	A	Missense_Mutation	423	0.040189125	No	0.182	Subclonal	Neutral	Passenger	Neutral	passenger	No	No	No	No	Not tested
CS1-C	Carcinoma	IL17RA	p.A598V	Private	22	17589902	C	T	Missense_Mutation	189	0.084656085	No	0.809	Clonal	Neutral	Passenger	Neutral	passenger	No	No	No	No	Not tested
CS1-C	Carcinoma	IL17RC	p.R751Sfs*14	Private	3	9975147	AAG	A	Frame_Shift_Del	264	0.170454545	No	1	Clonal	.	.	.	.	No	No	No	No	Not tested
CS1-C	Carcinoma	IL2RG	p.P361Hfs*5	Private	X	70327613	TG	T	Frame_Shift_Del	235	0.153191489	No	1	Clonal	.	.	.	.	No	No	No	No	Not tested
CS1-S	Sarcoma	IL3	p.T112M	Private	5	131398259	C	T	Missense_Mutation	662	0.401812689	No	1	Clonal	Neutral	Passenger	Neutral	passenger	No	No	No	No	Not tested
CS1-C	Carcinoma	IL4I1	p.R521C	Private	19	50393136	G	A	Missense_Mutation	266	0.033834586	No	0.323	Subclonal	Disease_causing	Passenger	Disease_causing	likely_pathogenic	No	No	No	No	Not tested
CS1-S	Sarcoma	IL4R	p.A82V	Private	16	27356225	C	T	Missense_Mutation	238	0.138655462	No	0.488	Subclonal	Neutral	Passenger	Neutral	passenger	No	No	No	No	Not tested
CS1-S	Sarcoma	ILDR2	p.L488*	Private	1	166890365	AG	A	Frame_Shift_Del	311	0.282958199	No	1	Clonal	.	.	.	.	No	No	No	No	Not tested
CS1-S	Sarcoma	INA	p.G331V	Private	10	105037960	G	T	Missense_Mutation	160	0.26875	No	0.946	Clonal	Disease_causing	Passenger	Disease_causing	likely_pathogenic	No	No	No	No	Not tested
CS1-S	Sarcoma	INADL	p.P959S	Private	1	62340954	C	T	Missense_Mutation	448	0.383928571	No	1	Clonal	Neutral	Passenger	Neutral	passenger	No	No	No	No	Not tested
CS1-C	Carcinoma	INHBA	p.A252T	Shared	7	41729775	C	T	Missense_Mutation	419	0.398568019	No	1	Clonal	Disease_causing	Passenger	Neutral	passenger	No	No	No	No	Not tested
CS1-S	Sarcoma	INHBA	p.A252T	Shared	7	41729775	C	T	Missense_Mutation	616	0.431818182	No	1	Clonal	Disease_causing	Passenger	Neutral	passenger	No	No	No	No	Not tested
CS1-C	Carcinoma	INPP5J	p.R506C	Private	22	31530004	C	T	Missense_Mutation	277	0.054151625	No	0.518	Subclonal	Disease_causing	Passenger	Disease_causing	likely_pathogenic	No	No	No	No	Not tested
CS1-C	Carcinoma	INPPL1	p.R1156Gfs*46	Shared	11	71948747	GC	G	Frame_Shift_Del	86	0.360465116	No	1	Clonal	.	.	.	.	No	No	No	No	Not tested
CS1-C	Carcinoma	INPPL1	p.S656del	Private	11	71944131	TCTC	T	In_Frame_Del	146	0.232876712	No	1	Clonal	.	.	.	.	No	No	No	No	Not tested
CS1-S	Sarcoma	INPPL1	p.R1156Gfs*46	Shared	11	71948747	GC	G	Frame_Shift_Del	97	0.649484536	No	1	Clonal	.	.	.	.	No	No	No	No	Not tested
CS1-S	Sarcoma	INTS1	p.G2022S	Private	7	1512714	C	T	Missense_Mutation	167	0.383233533	No	1	Clonal	Neutral	Passenger	Neutral	passenger	No	No	No	No	Not tested
CS1-S	Sarcoma	INTS3	p.R931W	Private	1	153744886	C	T	Missense_Mutation	859	0.086146682	No	0.389	Subclonal	Disease_causing	Passenger	.	passenger	No	No	No	No	Not tested
CS1-C	Carcinoma	IPO5	p.K544I	Private	13	98658463	A	T	Missense_Mutation	399	0.037593985	No	0.359	Subclonal	Disease_causing	Passenger	Neutral	passenger	No	No	No	No	Not tested
CS1-C	Carcinoma	IPO8	p.K503R	Private	12	30816509	T	C	Missense_Mutation	343	0.069970845	No	0.669	Subclonal	Disease_causing	Passenger	Neutral	passenger	No	No	No	No	Not tested
CS1-C	Carcinoma	IQGAP1	p.Y1530C	Private	15	91035904	A	G	Missense_Mutation	143	0.216783217	No	1	Clonal	Disease_causing	Driver	Neutral	likely_pathogenic	No	No	No	No	Not tested
CS1-C	Carcinoma	IRAK1	p.L493_G494del	Private	X	153279548	TGGCCCA	T	In_Frame_Del	144	0.138888889	No	1	Clonal	.	.	.	.	No	No	No	No	Not tested
CS1-S	Sarcoma	IRS4	p.G362C	Private	X	107978491	C	A	Missense_Mutation	591	0.395939086	No	1	Clonal	Neutral	Passenger	Neutral	passenger	No	No	No	No	Not tested
CS1-S	Sarcoma	ITGA2B	p.Q624H	Private	17	42457063	C	A	Missense_Mutation	218	0.036697248	No	0.129	Subclonal	Disease_causing	Passenger	Neutral	passenger	No	No	No	No	Not tested
CS1-C	Carcinoma	ITGA9	p.Y579H	Private	3	37670723	T	C	Missense_Mutation	99	0.141414141	No	1	Clonal	Disease_causing	Driver	Neutral	likely_pathogenic	No	No	No	No	Not tested
CS1-C	Carcinoma	ITGAX	p.G64D	Private	16	31367993	G	A	Missense_Mutation	108	0.138888889	No	1	Clonal	Disease_causing	Passenger	Neutral	passenger	No	No	No	No	Not tested
CS1-C	Carcinoma	ITGAX	p.V924I	Shared	16	31388567	G	A	Missense_Mutation	243	0.407407407	No	1	Clonal	Neutral	Passenger	Neutral	passenger	No	No	No	No	Not tested
CS1-S	Sarcoma	ITGAX	p.V924I	Shared	16	31388567	G	A	Missense_Mutation	310	0.432258065	No	1	Clonal	Neutral	Passenger	Neutral	passenger	No	No	No	No	Not tested
CS1-S	Sarcoma	ITGAX	p.L1108Sfs*3	Private	16	31392257	AC	A	Frame_Shift_Del	263	0.125475285	No	0.442	Subclonal	.	.	.	.	No	No	No	No	Not tested
CS1-S	Sarcoma	ITGB2	p.D80Y	Private	21	46326920	C	A	Missense_Mutation	227	0.052863436	No	0.186	Subclonal	Disease_causing	Passenger	Disease_causing	likely_pathogenic	No	No	No	No	Not tested
CS1-S	Sarcoma	ITIH4	p.*931Wext*2	Private	3	52847438	T	C	Nonstop_Mutation	116	0.431034483	No	1	Clonal	Disease_causing	.	.	passenger	No	No	No	No	Not tested
CS1-S	Sarcoma	ITLN2	p.X5_splice	Private	1	160924515	A	G	Splice_Site	496	0.245967742	No	1	Clonal	Neutral	.	.	passenger	No	No	No	No	Not tested
CS1-C	Carcinoma	ITPR3	p.A2373T	Private	6	33658778	G	A	Missense_Mutation	126	0.079365079	No	0.759	Clonal	Disease_causing	Passenger	Disease_causing	likely_pathogenic	No	No	No	No	Not tested
CS1-S	Sarcoma	JAG1	p.E553Rfs*11	Private	20	10628671	CG	C	Frame_Shift_Del	242	0.23553719	No	0.829	Subclonal	.	.	.	.	No	No	No	No	Not tested
CS1-S	Sarcoma	JPH4	p.A153V	Private	14	24045587	G	A	Missense_Mutation	132	0.348484848	No	1	Clonal	Disease_causing	Passenger	Neutral	passenger	No	No	No	No	Not tested
CS1-C	Carcinoma	JSRP1	p.A71V	Private	19	2254236	G	A	Missense_Mutation	418	0.102870813	No	0.984	Clonal	Neutral	Passenger	Neutral	passenger	No	No	No	No	Not tested
CS1-S	Sarcoma	KALRN	p.M1022Nfs*27	Private	3	124431854	C	CA	Frame_Shift_Ins	217	0.216589862	No	0.762	Subclonal	.	.	.	.	No	No	No	No	Not tested
CS1-S	Sarcoma	KALRN	p.Y1307F	Private	3	124181375	A	T	Missense_Mutation	318	0.374213836	No	1	Clonal	Disease_causing	Passenger	Neutral	passenger	No	No	No	No	Not tested
CS1-S	Sarcoma	KCNA2	p.R43Q	Private	1	111147277	C	T	Missense_Mutation	486	0.144032922	No	0.507	Subclonal	Disease_causing	Passenger	Neutral	passenger	No	No	No	No	Not tested
CS1-S	Sarcoma	KCNAB1	p.S45N	Private	3	155838534	G	A	Missense_Mutation	468	0.239316239	No	0.842	Subclonal	Neutral	Passenger	Neutral	passenger	No	No	No	No	Not tested
CS1-C	Carcinoma	KCNB1	p.R736Gfs*9	Private	20	47989890	CG	C	Frame_Shift_Del	276	0.173913043	No	1	Clonal	.	.	.	.	No	No	No	No	Not tested
CS1-S	Sarcoma	KCND1	p.W8*	Private	X	48826655	C	T	Nonsense_Mutation	254	0.05511811	No	0.194	Subclonal	Neutral	.	.	passenger	No	No	No	No	Not tested
CS1-C	Carcinoma	KCNE1	p.Q23R	Private	21	35821865	T	C	Missense_Mutation	278	0.035971223	No	0.344	Subclonal	Neutral	Passenger	Disease_causing	passenger	No	No	No	No	Not tested
CS1-C	Carcinoma	KCNG2	p.A41T	Private	18	77623788	G	A	Missense_Mutation	62	0.338709677	No	1	Clonal	Neutral	Passenger	Neutral	passenger	No	No	No	No	Not tested
CS1-C	Carcinoma	KCNG4	p.E95K	Private	16	84270809	C	T	Missense_Mutation	378	0.227513228	No	1	Clonal	Disease_causing	Passenger	Disease_causing	likely_pathogenic	No	No	No	No	Not tested
CS1-S	Sarcoma	KCNH5	p.V58I	Private	14	63483574	C	T	Missense_Mutation	372	0.26344086	No	0.927	Clonal	Disease_causing	Passenger	Disease_causing	likely_pathogenic	No	No	No	No	Not tested
CS1-S	Sarcoma	KCNH6	p.R379W	Private	17	61613063	C	T	Missense_Mutation	347	0.126801153	No	0.446	Subclonal	Disease_causing	Driver	Disease_causing	likely_pathogenic	No	No	No	No	Not tested
CS1-C	Carcinoma	KCNJ12	p.A158V	Private	17	21319127	C	T	Missense_Mutation	781	0.053777209	No	0.514	Subclonal	Disease_causing	Passenger	Disease_causing	likely_pathogenic	No	No	No	No	Not tested
CS1-C	Carcinoma	KCNJ4	p.G74D	Private	22	38823917	C	T	Missense_Mutation	268	0.223880597	No	1	Clonal	Disease_causing	Passenger	Disease_causing	likely_pathogenic	No	No	No	No	Not tested
CS1-S	Sarcoma	KCNJ5	p.I166M	Private	11	128781666	A	G	Missense_Mutation	537	0.277467412	No	0.976	Clonal	Neutral	Passenger	Disease_causing	passenger	No	Yes	No	No	Not tested
CS1-C	Carcinoma	KCNK13	p.V391Wfs*7	Private	14	90651284	AG	A	Frame_Shift_Del	132	0.234848485	No	1	Clonal	.	.	.	.	No	No	No	No	Not tested
CS1-S	Sarcoma	KCNK13	p.A234V	Private	14	90650821	C	T	Missense_Mutation	487	0.26899384	No	0.947	Clonal	Disease_causing	Passenger	Neutral	passenger	No	No	No	No	Not tested
CS1-S	Sarcoma	KCNK15	p.G97D	Private	20	43378776	G	A	Missense_Mutation	151	0.086092715	No	0.303	Subclonal	Disease_causing	Passenger	Neutral	passenger	No	No	No	No	Not tested
CS1-S	Sarcoma	KCNK17	p.X80_splice	Private	6	39278784	C	A	Splice_Site	80	0.1625	No	0.572	Subclonal	Disease_causing	.	.	passenger	No	No	No	No	Not tested
CS1-C	Carcinoma	KCNMA1	p.V907I	Private	10	78704663	C	T	Missense_Mutation	147	0.034013605	No	0.325	Subclonal	Disease_causing	Passenger	Neutral	passenger	No	No	No	No	Not tested
CS1-S	Sarcoma	KCNMA1	p.P256S	Private	10	78943221	G	A	Missense_Mutation	139	0.100719424	No	0.354	Subclonal	Disease_causing	Passenger	Neutral	passenger	No	No	No	No	Not tested
CS1-S	Sarcoma	KCTD16	p.F55V	Private	5	143586440	T	G	Missense_Mutation	672	0.12202381	No	0.429	Subclonal	Disease_causing	Passenger	Neutral	passenger	No	No	No	No	Not tested
CS1-S	Sarcoma	KCTD19	p.V768Rfs*12	Private	16	67325657	C	CG	Frame_Shift_Ins	323	0.325077399	No	1	Clonal	.	.	.	.	No	No	No	No	Not tested
CS1-S	Sarcoma	KCTD21	p.A110T	Private	11	77885273	C	T	Missense_Mutation	206	0.257281553	No	0.905	Clonal	Disease_causing	Passenger	Neutral	passenger	No	No	No	No	Not tested
CS1-C	Carcinoma	KDM3B	p.F1039del	Private	5	137735614	CTCT	C	In_Frame_Del	243	0.148148148	No	1	Clonal	.	.	.	.	No	No	No	No	Not tested
CS1-C	Carcinoma	KDM6B	p.Q897K	Private	17	7752295	C	A	Missense_Mutation	126	0.047619048	No	0.455	Subclonal	Neutral	Passenger	Neutral	passenger	No	No	No	No	Not tested
CS1-S	Sarcoma	KDM8	p.Q200R	Private	16	27221929	A	G	Missense_Mutation	190	0.389473684	No	1	Clonal	Neutral	Passenger	Neutral	passenger	No	No	No	No	Not tested
CS1-C	Carcinoma	KHNYN	p.R346Q	Shared	14	24901504	G	A	Missense_Mutation	356	0.404494382	No	1	Clonal	Disease_causing	Passenger	Neutral	passenger	No	No	No	No	Not tested
CS1-S	Sarcoma	KHNYN	p.R346Q	Shared	14	24901504	G	A	Missense_Mutation	509	0.438113949	No	1	Clonal	Disease_causing	Passenger	Neutral	passenger	No	No	No	No	Not tested
CS1-C	Carcinoma	KIAA0195	p.S895Pfs*13	Private	17	73491062	TC	T	Frame_Shift_Del	141	0.184397163	No	1	Clonal	.	.	.	.	No	No	No	No	Not tested
CS1-S	Sarcoma	KIAA0319L	p.P503Lfs*10	Private	1	35921761	AG	A	Frame_Shift_Del	277	0.368231047	No	1	Clonal	.	.	.	.	No	No	No	No	Not tested
CS1-C	Carcinoma	KIAA0513	p.P299S	Private	16	85112602	C	T	Missense_Mutation	150	0.046666667	No	0.446	Subclonal	Disease_causing	Passenger	Neutral	passenger	No	No	No	No	Not tested
CS1-C	Carcinoma	KIAA0922	p.C143R	Private	4	154477057	T	C	Missense_Mutation	204	0.225490196	No	1	Clonal	Disease_causing	Passenger	Neutral	passenger	No	No	No	No	Not tested
CS1-S	Sarcoma	KIAA0947	p.A1724T	Private	5	5464617	G	A	Missense_Mutation	474	0.265822785	No	0.935	Clonal	Disease_causing	Passenger	Neutral	passenger	No	No	No	No	Not tested
CS1-C	Carcinoma	KIAA1024	p.S702Afs*11	Shared	15	79750585	TA	T	Frame_Shift_Del	267	0.228464419	No	1	Clonal	.	.	.	.	No	No	No	No	Not tested
CS1-S	Sarcoma	KIAA1024	p.S702Afs*11	Shared	15	79750585	TA	T	Frame_Shift_Del	436	0.121559633	No	0.428	Clonal	.	.	.	.	No	No	No	No	Not tested
CS1-C	Carcinoma	KIAA1109	p.N610Ifs*7	Private	4	123130384	TA	T	Frame_Shift_Del	247	0.226720648	No	1	Clonal	.	.	.	.	No	No	No	No	Not tested
CS1-C	Carcinoma	KIAA1109	p.K3591Nfs*12	Private	4	123239398	TA	T	Frame_Shift_Del	312	0.192307692	No	1	Clonal	.	.	.	.	No	No	No	No	Not tested
CS1-S	Sarcoma	KIAA1109	p.K1590Nfs*11	Private	4	123165028	GA	G	Frame_Shift_Del	191	0.193717277	No	0.682	Subclonal	.	.	.	.	No	No	No	No	Not tested
CS1-S	Sarcoma	KIAA1109	p.A2011T	Private	4	123175458	G	A	Missense_Mutation	295	0.179661017	No	0.632	Subclonal	Disease_causing	Passenger	Neutral	passenger	No	No	No	No	Not tested
CS1-S	Sarcoma	KIAA1257	p.T391I	Private	3	128690126	G	A	Missense_Mutation	174	0.333333333	No	1	Clonal	Disease_causing	Passenger	.	passenger	No	No	No	No	Not tested
CS1-S	Sarcoma	KIAA1377	p.E732Kfs*25	Private	11	101833952	CA	C	Frame_Shift_Del	383	0.399477807	No	1	Clonal	.	.	.	.	No	No	No	No	Not tested
CS1-C	Carcinoma	KIF15	p.D274Y	Shared	3	44835889	G	T	Missense_Mutation	141	0.411347518	No	1	Clonal	Disease_causing	Passenger	Neutral	passenger	No	No	No	No	Not tested
CS1-S	Sarcoma	KIF15	p.D274Y	Shared	3	44835889	G	T	Missense_Mutation	163	0.404907975	No	1	Clonal	Disease_causing	Passenger	Neutral	passenger	No	No	No	No	Not tested
CS1-C	Carcinoma	KIF24	p.K661Sfs*34	Private	9	34257622	CT	C	Frame_Shift_Del	332	0.189759036	No	1	Clonal	.	.	.	.	No	No	No	No	Not tested
CS1-S	Sarcoma	KIF26B	p.R2002W	Private	1	245861587	C	T	Missense_Mutation	231	0.32034632	No	1	Clonal	Disease_causing	Passenger	Disease_causing	likely_pathogenic	No	No	No	No	Not tested
CS1-C	Carcinoma	KIF2B	p.Q577R	Private	17	51902124	A	G	Missense_Mutation	282	0.031914894	No	0.305	Subclonal	Neutral	Passenger	Neutral	passenger	No	No	No	No	Not tested
CS1-C	Carcinoma	KIF4B	p.Y51H	Private	5	154393570	T	C	Missense_Mutation	355	0.081690141	No	0.781	Clonal	Disease_causing	Passenger	Neutral	passenger	No	No	No	No	Not tested
CS1-C	Carcinoma	KIF7	p.V120I	Private	15	90193143	C	T	Missense_Mutation	307	0.084690554	No	0.81	Clonal	Neutral	Passenger	Neutral	passenger	No	No	No	No	Not tested
CS1-C	Carcinoma	KLF4	p.R205L	Private	9	110250061	C	A	Missense_Mutation	200	0.045	No	0.43	Subclonal	Disease_causing	Passenger	Neutral	passenger	No	Yes	No	No	Not tested
CS1-C	Carcinoma	KLHL22	p.H474R	Private	22	20800848	T	C	Missense_Mutation	216	0.125	No	1	Clonal	Neutral	Passenger	Neutral	passenger	No	No	No	No	Not tested
CS1-C	Carcinoma	KLHL33	p.L387P	Private	14	20897450	A	G	Missense_Mutation	308	0.25974026	No	1	Clonal	Neutral	Passenger	Neutral	passenger	No	No	No	No	Not tested
CS1-C	Carcinoma	KLHL38	p.H566Y	Shared	8	124658029	G	A	Missense_Mutation	207	0.376811594	No	1	Clonal	Disease_causing	Passenger	Neutral	passenger	No	No	No	No	Not tested
CS1-S	Sarcoma	KLHL38	p.H566Y	Shared	8	124658029	G	A	Missense_Mutation	418	0.277511962	No	1	Clonal	Disease_causing	Passenger	Neutral	passenger	No	No	No	No	Not tested
CS1-C	Carcinoma	KMT2C	p.C139R	Private	7	152012398	A	G	Missense_Mutation	196	0.030612245	No	0.293	Subclonal	Disease_causing	Passenger	Disease_causing	likely_pathogenic	No	Yes	Yes	Yes	Not tested
CS1-C	Carcinoma	KMT2C	p.F4496Lfs*21	Private	7	151845523	TA	T	Frame_Shift_Del	268	0.182835821	No	1	Clonal	.	.	.	.	No	Yes	Yes	Yes	Not tested
CS1-S	Sarcoma	KMT2C	p.Q4877*	Private	7	151835895	G	A	Nonsense_Mutation	538	0.130111524	No	0.458	Subclonal	Disease_causing	.	.	likely_pathogenic	No	Yes	Yes	Yes	Not tested
CS1-C	Carcinoma	KMT2D	p.A21T	Private	12	49448798	C	T	Missense_Mutation	299	0.076923077	No	0.735	Subclonal	Neutral	Passenger	Neutral	passenger	No	Yes	Yes	Yes	Not tested
CS1-S	Sarcoma	KMT2D	p.P2392Lfs*34	Private	12	49434377	AG	A	Frame_Shift_Del	126	0.404761905	No	1	Clonal	.	.	.	.	No	Yes	Yes	Yes	Not tested
CS1-C	Carcinoma	KMT2E	p.R1167L	Private	7	104749420	G	T	Missense_Mutation	175	0.062857143	No	0.601	Subclonal	Disease_causing	Passenger	Neutral	passenger	No	No	No	No	Not tested

CS1-C	Carcinoma	KMT2E	p.T1041M	Private	7	104748026	C	T	Missense_Mutation	[illegible]	[illegible]	No	0.668	Subclonal	Neutral	Passenger	Neutral	passenger	No	No	No	No	Not tested
CS1-C	Carcinoma	KNTC1	p.Q1602H	Private	12	123087268	G	T	Missense_Mutation	[illegible]	[illegible]	No	1	Clonal	Disease_causing	Passenger	Neutral	passenger	No	No	No	No	Not tested
CS1-C	Carcinoma	KRT222	p.R113C	Private	17	38816348	G	A	Missense_Mutation	308	0.051948052	No	0.497	Subclonal	Disease_causing	Passenger	Disease_causing	likely_pathogenic	No	No	No	No	Not tested
CS1-C	Carcinoma	KRT24	p.E331Gfs*2	Private	17	38856497	CCT	C	Frame_Shift_Del	330	0.187878788	No	1	Clonal	.	.	.	.	No	No	No	No	Not tested
CS1-C	Carcinoma	KRT76	p.S532C	Shared	12	53162820	T	A	Missense_Mutation	227	0.392070485	No	1	Clonal	Neutral	Passenger	Disease_causing	passenger	No	No	No	No	Not tested
CS1-S	Sarcoma	KRT76	p.S532C	Shared	12	53162820	T	A	Missense_Mutation	316	0.462025316	No	1	Clonal	Neutral	Passenger	Disease_causing	passenger	No	No	No	No	Not tested
CS1-C	Carcinoma	KRTDAP	p.P78Lfs*11	Private	19	35978641	AG	A	Frame_Shift_Del	220	0.25	No	1	Clonal	.	.	.	.	No	No	No	No	Not tested
CS1-C	Carcinoma	KY	p.D423N	Private	3	134323140	C	T	Missense_Mutation	250	0.048	No	0.459	Subclonal	Disease_causing	Passenger	.	passenger	No	No	No	No	Not tested
CS1-C	Carcinoma	L3MBTL1	p.G173R	Private	20	42143729	G	A	Missense_Mutation	214	0.042056075	No	0.402	Subclonal	Neutral	Passenger	Disease_causing	passenger	No	No	No	No	Not tested
CS1-C	Carcinoma	LAMA2	p.C1557*	Private	6	129674456	T	A	Nonsense_Mutation	155	0.051612903	No	0.493	Subclonal	Neutral	.	.	passenger	No	No	No	No	Not tested
CS1-S	Sarcoma	LAMA3	p.H1594Y	Private	18	21451407	C	T	Missense_Mutation	364	0.274725275	No	0.967	Clonal	Disease_causing	Passenger	Neutral	passenger	No	No	No	No	Not tested
CS1-C	Carcinoma	LAMA4	p.V1059M	Private	6	112460429	C	T	Missense_Mutation	377	0.082228117	No	0.786	Clonal	Neutral	Passenger	Neutral	passenger	No	No	No	No	Not tested
CS1-S	Sarcoma	LAMA4	p.N1605Mfs*33	Private	6	112440365	AT	A	Frame_Shift_Del	130	0.461538462	No	1	Clonal	.	.	.	.	No	No	No	No	Not tested
CS1-S	Sarcoma	LAMP3	p.T268M	Private	3	182870248	G	A	Missense_Mutation	281	0.409252669	No	1	Clonal	Disease_causing	Passenger	Neutral	passenger	No	No	No	No	Not tested
CS1-C	Carcinoma	LARP1	p.F919del	Private	5	154191103	CCTT	C	In_Frame_Del	253	0.177865613	No	1	Clonal	.	.	.	.	No	No	No	No	Not tested
CS1-C	Carcinoma	LARP4	p.C224Y	Private	12	50834253	G	A	Missense_Mutation	164	0.048780488	No	0.466	Subclonal	Disease_causing	Passenger	Neutral	passenger	No	No	No	No	Not tested
CS1-C	Carcinoma	LCE4A	p.P12S	Private	1	152681585	C	T	Missense_Mutation	326	0.17791411	No	1	Clonal	Neutral	Passenger	Neutral	passenger	No	No	No	No	Not tested
CS1-C	Carcinoma	LENG8	p.A699T	Private	19	54969555	G	A	Missense_Mutation	258	0.255813953	No	1	Clonal	Disease_causing	Passenger	Neutral	passenger	No	No	No	No	Not tested
CS1-S	Sarcoma	LGI3	p.N121S	Private	8	22011715	T	C	Missense_Mutation	443	0.083521445	No	0.377	Subclonal	Disease_causing	Passenger	Neutral	passenger	No	No	No	No	Not tested
CS1-C	Carcinoma	LGR5	p.S159G	Private	12	71946899	A	G	Missense_Mutation	219	0.073059361	No	0.699	Subclonal	Neutral	Passenger	Neutral	passenger	No	No	No	No	Not tested
CS1-S	Sarcoma	LIG3	p.A189T	Private	17	33313024	G	A	Missense_Mutation	387	0.095607235	No	0.336	Subclonal	Neutral	Passenger	Neutral	passenger	No	No	No	No	Not tested
CS1-S	Sarcoma	LIN28B	p.N168Mfs*65	Private	6	105526403	TA	T	Frame_Shift_Del	334	0.380239521	No	1	Clonal	.	.	.	.	No	No	No	No	Not tested
CS1-C	Carcinoma	LIN7B	p.R194H	Private	19	49621254	G	A	Missense_Mutation	157	0.057324841	No	0.548	Subclonal	Disease_causing	Passenger	Neutral	passenger	No	No	No	No	Not tested
CS1-S	Sarcoma	LINGO4	p.Y273C	Private	1	151774363	T	C	Missense_Mutation	552	0.307971014	No	1	Clonal	Neutral	Passenger	Neutral	passenger	No	No	No	No	Not tested
CS1-S	Sarcoma	LMTK2	p.I651Yfs*4	Private	7	97821722	CA	C	Frame_Shift_Del	368	0.266304348	No	0.937	Clonal	.	.	.	.	No	No	No	No	Not tested
CS1-C	Carcinoma	LNX2	p.R345Q	Shared	13	28136740	C	T	Missense_Mutation	434	0.366359447	No	1	Clonal	Disease_causing	Passenger	Neutral	passenger	No	No	No	No	Not tested
CS1-S	Sarcoma	LNX2	p.R345Q	Shared	13	28136740	C	T	Missense_Mutation	608	0.417763158	No	1	Clonal	Disease_causing	Passenger	Neutral	passenger	No	No	No	No	Not tested
CS1-C	Carcinoma	LOXHD1	p.A427T	Private	18	44104799	C	T	Missense_Mutation	397	0.236775819	No	1	Clonal	Disease_causing	Passenger	Neutral	passenger	No	No	No	No	Not tested
CS1-C	Carcinoma	LPA	p.S1867P	Private	6	160961211	A	G	Missense_Mutation	340	0.035294118	No	0.337	Subclonal	Neutral	Passenger	Disease_causing	passenger	No	No	No	No	Not tested
CS1-C	Carcinoma	LPGAT1	p.G185V	Private	1	211956744	C	A	Missense_Mutation	305	0.039344262	No	0.416	Subclonal	Disease_causing	Passenger	Disease_causing	likely_pathogenic	No	No	No	No	Not tested
CS1-C	Carcinoma	LPIN1	p.G636Rfs*19	Private	2	11932075	C	CA	Frame_Shift_Ins	287	0.216027875	No	1	Clonal	.	.	.	.	No	No	No	No	Not tested
CS1-C	Carcinoma	LRAT	p.D111N	Private	4	155665809	G	A	Missense_Mutation	376	0.058510638	No	0.559	Subclonal	Disease_causing	Passenger	Neutral	passenger	No	No	No	No	Not tested
CS1-S	Sarcoma	LRBA	p.W2616*	Private	4	151231415	C	T	Nonsense_Mutation	145	0.351724138	No	1	Clonal	Neutral	.	.	passenger	No	No	No	No	Not tested
CS1-S	Sarcoma	LRFN4	p.G524Vfs*100	Private	11	66627323	TG	T	Frame_Shift_Del	177	0.389830508	No	1	Clonal	.	.	.	.	No	No	No	No	Not tested
CS1-C	Carcinoma	LRIG2	p.R741*	Private	1	113657189	C	T	Nonsense_Mutation	296	0.168918919	No	1	Clonal	Neutral	.	.	passenger	No	No	No	No	Not tested
CS1-S	Sarcoma	LRIG2	p.D76Rfs*10	Private	1	113616247	G	GC	Frame_Shift_Ins	252	0.174603175	No	0.614	Subclonal	.	.	.	.	No	No	No	No	Not tested
CS1-S	Sarcoma	LRP1	p.R2206C	Private	12	57579466	C	T	Missense_Mutation	595	0.426890756	No	1	Clonal	Disease_causing	Passenger	Disease_causing	likely_pathogenic	No	No	No	No	Not tested
CS1-C	Carcinoma	LRP2	p.P4380Hfs*46	Shared	2	169997024	TG	T	Frame_Shift_Del	191	0.062827225	No	0.601	Subclonal	.	.	.	.	No	No	No	No	Not tested
CS1-S	Sarcoma	LRP2	p.P4380Hfs*46	Shared	2	169997024	TG	T	Frame_Shift_Del	295	0.355932203	No	1	Clonal	.	.	.	.	No	No	No	No	Not tested
CS1-C	Carcinoma	LRP3	p.P685S	Private	19	33698221	C	T	Missense_Mutation	163	0.208588957	No	1	Clonal	Neutral	Passenger	Disease_causing	passenger	No	No	No	No	Not tested
CS1-C	Carcinoma	LRRC15	p.Y521H	Private	3	194080230	A	G	Missense_Mutation	312	0.064102564	No	0.613	Subclonal	Neutral	Passenger	Neutral	passenger	No	No	No	No	Not tested
CS1-C	Carcinoma	LRRC66	p.H434Y	Private	4	52861888	G	A	Missense_Mutation	339	0.109144543	No	1	Clonal	Neutral	Passenger	Neutral	passenger	No	No	No	No	Not tested
CS1-C	Carcinoma	LRRIQ4	p.R306L	Private	3	169540626	G	T	Missense_Mutation	295	0.074576271	No	0.713	Subclonal	Neutral	Passenger	Neutral	passenger	No	No	No	No	Not tested
CS1-S	Sarcoma	LRRK1	p.P647Rfs*115	Private	15	101562670	TC	T	Frame_Shift_Del	287	0.243902439	No	0.858	Subclonal	.	.	.	.	No	No	No	No	Not tested
CS1-C	Carcinoma	LRRN2	p.Q353R	Private	1	204588063	T	C	Missense_Mutation	382	0.036649215	No	0.387	Subclonal	Disease_causing	Passenger	Neutral	passenger	No	No	No	No	Not tested
CS1-S	Sarcoma	LSP1	p.H15Y	Private	11	1887747	C	T	Missense_Mutation	298	0.395973154	No	1	Clonal	Neutral	Passenger	Neutral	passenger	No	No	No	No	Not tested
CS1-S	Sarcoma	LTB	p.G168Afs*18	Private	6	31548717	GC	G	Frame_Shift_Del	209	0.33492823	No	1	Clonal	.	.	.	.	No	No	No	No	Not tested
CS1-C	Carcinoma	LTBP1	p.M690I	Private	2	33477814	G	A	Missense_Mutation	300	0.09	No	0.95	Clonal	Disease_causing	Passenger	Disease_causing	likely_pathogenic	No	No	No	No	Not tested
CS1-C	Carcinoma	LTBR	p.L228P	Private	12	6497579	T	C	Missense_Mutation	134	0.171641791	No	1	Clonal	Disease_causing	Passenger	Disease_causing	likely_pathogenic	No	No	No	No	Not tested
CS1-S	Sarcoma	LUC7L2	p.L15M	Private	7	139026173	C	A	Missense_Mutation	412	0.451456311	No	1	Clonal	Disease_causing	Passenger	Neutral	passenger	No	No	No	No	Not tested
CS1-S	Sarcoma	LYRM7	p.I52L	Private	5	130517984	A	C	Missense_Mutation	72	0.486111111	No	1	Clonal	Disease_causing	Passenger	Neutral	passenger	No	No	No	No	Not tested
CS1-C	Carcinoma	LZTR1	p.R283W	Shared	22	21345972	C	T	Missense_Mutation	195	0.374358974	No	1	Clonal	Disease_causing	Passenger	Neutral	passenger	No	Yes	No	No	Not tested
CS1-S	Sarcoma	LZTR1	p.R283W	Shared	22	21345972	C	T	Missense_Mutation	275	0.472727273	No	1	Clonal	Disease_causing	Passenger	Neutral	passenger	No	Yes	No	No	Not tested
CS1-S	Sarcoma	MAFG	p.R69C	Private	17	79880765	G	A	Missense_Mutation	537	0.040968343	No	0.144	Subclonal	Disease_causing	Passenger	Disease_causing	likely_pathogenic	No	No	No	No	Not tested
CS1-S	Sarcoma	MAGEB5	p.R174G	Private	X	26235938	A	G	Missense_Mutation	363	0.38292011	No	1	Clonal	Neutral	Passenger	Neutral	passenger	No	No	No	No	Not tested
CS1-C	Carcinoma	MAMLD1	p.A96V	Shared	X	149638207	C	T	Missense_Mutation	356	0.359550562	No	1	Clonal	Neutral	Passenger	Neutral	passenger	No	No	No	No	Not tested
CS1-C	Carcinoma	MAMLD1	p.Q477dup	Private	X	149639324	A	ACAG	In_Frame_Ins	370	0.148648649	No	1	Clonal	.	.	.	.	No	No	No	No	Not tested
CS1-S	Sarcoma	MAMLD1	p.A96V	Shared	X	149638207	C	T	Missense_Mutation	464	0.372844828	No	1	Clonal	Neutral	Passenger	Neutral	passenger	No	No	No	No	Not tested
CS1-C	Carcinoma	MAN1C1	p.Q174*	Private	1	25944808	C	T	Nonsense_Mutation	250	0.032	No	0.306	Subclonal	Disease_causing	.	.	passenger	No	No	No	No	Not tested
CS1-S	Sarcoma	MAP2K7	p.R217H	Private	19	7975663	G	A	Missense_Mutation	563	0.131438721	No	0.463	Subclonal	Disease_causing	Passenger	Neutral	passenger	No	No	No	No	Not tested
CS1-S	Sarcoma	MAP3K13	p.R803Q	Private	3	185191527	G	A	Missense_Mutation	135	0.511111111	No	1	Clonal	Disease_causing	Passenger	Neutral	passenger	No	Yes	No	No	Not tested
CS1-C	Carcinoma	MAP3K6	p.A664T	Private	1	27687260	C	T	Missense_Mutation	128	0.0390625	No	0.373	Subclonal	Disease_causing	Passenger	Neutral	passenger	No	No	No	No	Not tested
CS1-S	Sarcoma	MAP3K9	p.R1010C	Private	14	71197426	G	A	Missense_Mutation	385	0.275324675	No	0.969	Clonal	Disease_causing	Driver	Disease_causing	likely_pathogenic	No	No	No	No	Not tested
CS1-S	Sarcoma	MAP6D1	p.P79S	Private	3	183543101	G	A	Missense_Mutation	141	0.177304965	No	0.624	Subclonal	Neutral	Passenger	Neutral	passenger	No	No	No	No	Not tested
CS1-C	Carcinoma	MAPK14	p.G181D	Private	6	36043671	G	A	Missense_Mutation	308	0.224025974	No	1	Clonal	Disease_causing	Passenger	Neutral	passenger	No	No	No	No	Not tested
CS1-C	Carcinoma	MAST2	p.F726Lfs*12	Shared	1	46494558	CT	C	Frame_Shift_Del	214	0.23364486	No	1	Clonal	.	.	.	.	No	No	No	No	Not tested
CS1-S	Sarcoma	MAST2	p.F726Lfs*12	Shared	1	46494558	CT	C	Frame_Shift_Del	287	0.090592334	No	0.319	Clonal	.	.	.	.	No	No	No	No	Not tested
CS1-C	Carcinoma	MAST4	p.P2572Qfs*11	Private	5	66462717	TC	T	Frame_Shift_Del	242	0.090909091	No	0.869	Clonal	.	.	.	.	No	No	No	No	Not tested
CS1-C	Carcinoma	MBD6	p.G782Efs*13	Shared	12	57921731	TG	T	Frame_Shift_Del	304	0.128289474	No	1	Clonal	.	.	.	.	No	No	No	No	Not tested
CS1-S	Sarcoma	MBD6	p.G782Efs*13	Shared	12	57921731	TG	T	Frame_Shift_Del	429	0.400932401	No	1	Clonal	.	.	.	.	No	No	No	No	Not tested
CS1-C	Carcinoma	MCAT	p.Y306C	Private	22	43529305	T	C	Missense_Mutation	395	0.050632911	No	0.484	Subclonal	Disease_causing	Passenger	Neutral	passenger	No	No	No	No	Not tested
CS1-C	Carcinoma	MCCC2	p.P224S	Private	5	70922512	C	T	Missense_Mutation	157	0.076433121	No	0.731	Subclonal	Disease_causing	Passenger	Disease_causing	likely_pathogenic	No	No	No	No	Not tested
CS1-S	Sarcoma	MCTP2	p.G137S	Private	15	94841903	G	A	Missense_Mutation	266	0.345864662	No	1	Clonal	Neutral	Passenger	Neutral	passenger	No	No	No	No	Not tested
CS1-C	Carcinoma	MDN1	p.A5068V	Private	6	90366501	G	A	Missense_Mutation	197	0.218274112	No	1	Clonal	Disease_causing	Passenger	Neutral	passenger	No	No	No	No	Not tested
CS1-C	Carcinoma	MEGF6	p.C900R	Private	1	3417206	A	G	Missense_Mutation	113	0.061946903	No	0.592	Subclonal	Disease_causing	Passenger	Neutral	passenger	No	No	No	No	Not tested
CS1-C	Carcinoma	MEGF9	p.A602V	Private	9	123367472	G	A	Missense_Mutation	270	0.055555556	No	0.531	Subclonal	Disease_causing	Passenger	Neutral	passenger	No	No	No	No	Not tested
CS1-C	Carcinoma	MEX3A	p.G67Afs*60	Private	1	156051589	GC	G	Frame_Shift_Del	164	0.158536585	No	1	Clonal	.	.	.	.	No	No	No	No	Not tested
CS1-S	Sarcoma	MEX3C	p.T395I	Private	18	48703517	G	A	Missense_Mutation	450	0.12	No	0.422	Subclonal	Disease_causing	Passenger	Neutral	passenger	No	No	No	No	Not tested
CS1-C	Carcinoma	MFAP1	p.S132N	Private	15	44107177	C	T	Missense_Mutation	310	0.248387097	No	1	Clonal	Disease_causing	Passenger	.	passenger	No	No	No	No	Not tested
CS1-C	Carcinoma	MFSD6	p.S444N	Private	2	191302086	G	A	Missense_Mutation	428	0.035046729	No	0.335	Subclonal	Disease_causing	Passenger	Neutral	passenger	No	No	No	No	Not tested
CS1-S	Sarcoma	MFSD6L	p.A536V	Private	17	8700832	G	A	Missense_Mutation	444	0.121621622	No	0.428	Subclonal	Neutral	Passenger	Neutral	passenger	No	No	No	No	Not tested
CS1-S	Sarcoma	MGAT1	p.A42T	Private	5	180219848	C	T	Missense_Mutation	72	0.180555556	No	0.635	Subclonal	Neutral	Passenger	Disease_causing	passenger	No	No	No	No	Not tested
CS1-C	Carcinoma	MID2	p.R408*	Private	X	107160756	C	T	Nonsense_Mutation	465	0.077419355	No	0.74	Subclonal	Neutral	.	.	passenger	No	No	No	No	Not tested
CS1-C	Carcinoma	MID2	p.A608T	Private	X	107169917	G	A	Missense_Mutation	230	0.030434783	No	0.291	Subclonal	Disease_causing	Passenger	Neutral	passenger	No	No	No	No	Not tested
CS1-C	Carcinoma	MINK1	p.E1233D	Private	17	4799807	G	T	Missense_Mutation	145	0.103448276	No	0.989	Clonal	Disease_causing	Passenger	Neutral	passenger	No	No	No	No	Not tested
CS1-S	Sarcoma	MISP	p.Q435Sfs*2	Private	19	758243	AC	A	Frame_Shift_Del	427	0.1264637	No	0.445	Subclonal	.	.	.	.	No	No	No	No	Not tested
CS1-C	Carcinoma	MKI67	p.S870F	Private	10	129907495	G	A	Missense_Mutation	257	0.038910506	No	0.372	Subclonal	Neutral	Passenger	Neutral	passenger	No	No	No	No	Not tested
CS1-C	Carcinoma	MKRN1	p.A391V	Private	7	140154959	G	A	Missense_Mutation	58	0.189655172	No	1	Clonal	Disease_causing	Passenger	Neutral	passenger	No	No	No	No	Not tested
CS1-S	Sarcoma	MLH3	p.K881T	Private	14	75513717	T	G	Missense_Mutation	508	0.149606299	No	0.526	Subclonal	Disease_causing	Passenger	Disease_causing	likely_pathogenic	No	No	No	No	Not tested
CS1-S	Sarcoma	MLLT4	p.G1139E	Private	6	168347465	G	A	Missense_Mutation	269	0.412639405	No	1	Clonal	Disease_causing	Passenger	Neutral	passenger	No	Yes	No	No	Not tested
CS1-S	Sarcoma	MLPH	p.E123A	Private	2	238419667	A	C	Missense_Mutation	525	0.064761905	No	0.228	Subclonal	Neutral	Passenger	Neutral	passenger	No	No	No	No	Not tested
CS1-S	Sarcoma	MMGT1	p.H87R	Private	X	135047319	T	C	Missense_Mutation	289	0.034602076	No	0.122	Subclonal	Disease_causing	Passenger	.	passenger	No	No	No	No	Not tested
CS1-C	Carcinoma	MMP16	p.D321Efs*21	Shared	8	89128848	TTTGGCCTG	T	Frame_Shift_Del	189	0.195767196	No	1	Clonal	.	.	.	.	No	No	No	No	Not tested
CS1-S	Sarcoma	MMP16	p.D321Efs*21	Shared	8	89128848	TTTGGCCTG	T	Frame_Shift_Del	391	0.135549872	No	0.613	Clonal	.	.	.	.	No	No	No	No	Not tested
CS1-C	Carcinoma	MMP26	p.A23Cfs*5	Private	11	5009501	G	GC	Frame_Shift_Ins	332	0.186746988	No	1	Clonal	.	.	.	.	No	No	No	No	Not tested
CS1-S	Sarcoma	MORC4	p.E673*	Private	X	106186104	C	A	Nonsense_Mutation	481	0.118503119	No	0.417	Subclonal	Neutral	.	.	passenger	No	No	No	No	Not tested
CS1-S	Sarcoma	MORN1	p.K53N	Private	1	2319766	C	A	Missense_Mutation	511	0.221135029	No	0.778	Subclonal	Neutral	Passenger	Neutral	passenger	No	No	No	No	Not tested
CS1-C	Carcinoma	MPPED1	p.R324Q	Private	22	43901486	G	A	Missense_Mutation	315	0.203174603	No	1	Clonal	Disease_causing	Passenger	Neutral	passenger	No	No	No	No	Not tested
CS1-C	Carcinoma	MRC2	p.W1229*	Private	17	60767368	G	A	Nonsense_Mutation	101	0.059405941	No	0.568	Subclonal	Neutral	.	.	passenger	No	No	No	No	Not tested
CS1-C	Carcinoma	MROH2B	p.F1426Lfs*5	Private	5	41000851	CA	C	Frame_Shift_Del	164	0.280487805	No	1	Clonal	.	.	.	.	No	No	No	No	Not tested
CS1-C	Carcinoma	MROH8	p.R50S	Private	20	35807816	G	T	Missense_Mutation	93	0.161290323	No	1	Clonal	Disease_causing	Passenger	Neutral	passenger	No	No	No	No	Not tested
CS1-S	Sarcoma	MROH8	p.S171I	Private	20	35800409	C	A	Missense_Mutation	268	0.253731343	No	0.893	Subclonal	Neutral	Passenger	Neutral	passenger	No	No	No	No	Not tested
CS1-S	Sarcoma	MSANTD2	p.A488T	Private	11	124637134	C	T	Missense_Mutation	373	0.056300268	No	0.198	Subclonal	Disease_causing	Passenger	.	passenger	No	No	No	No	Not tested
CS1-C	Carcinoma	MSANTD3	p.I84Lfs*10	Private	9	103204463	CA	C	Frame_Shift_Del	392	0.224489796	No	1	Clonal	.	.	.	.	No	No	No	No	Not tested
CS1-S	Sarcoma	MSH3	p.E751D	Private	5	80064822	G	T	Missense_Mutation	131	0.389312977	No	1	Clonal	Disease_causing	Passenger	Disease_causing	likely_pathogenic	No	No	No	No	Not tested
CS1-C	Carcinoma	MSH4	p.P345H	Private	1	76288138	C	A	Missense_Mutation	126	0.103174603	No	0.986	Clonal	Disease_causing	Passenger	Disease_causing	likely_pathogenic	No	No	No	No	Not tested
CS1-C	Carcinoma	MSRB3	p.A182T	Private	12	65857067	G	A	Missense_Mutation	133	0.105263158	No	1	Clonal	Neutral	Passenger	Neutral	passenger	No	No	No	No	Not tested
CS1-C	Carcinoma	MTMR10	p.F760I	Private	15	31233729	A	T	Missense_Mutation	345	0.255072464	No	1	Clonal	Disease_causing	Passenger	Disease_causing	likely_pathogenic	No	No	No	No	Not tested
CS1-C	Carcinoma	MTMR4	p.F340S	Private	17	56582917	A	G	Missense_Mutation	233	0.231759657	No	1	Clonal	Disease_causing	Driver	Disease_causing	likely_pathogenic	No	No	No	No	Not tested
CS1-C	Carcinoma	MTSS1	p.G572D	Private	8	125565786	C	T	Missense_Mutation	281	0.053380783	No	0.51	Subclonal	Disease_causing	Passenger	Neutral	passenger	No	No	No	No	Not tested
CS1-C	Carcinoma	MTUS2	p.E69G	Private	13	29599011	A	G	Missense_Mutation	403	0.034739454	No	0.332	Subclonal	Neutral	Passenger	Neutral	passenger	No	No	No	No	Not tested
CS1-C	Carcinoma	MUC21	p.S28N	Private	6	30954035	G	A	Missense_Mutation	343	0.186588921	No	1	Clonal	Neutral	Passenger	Neutral	passenger	No	No	No	No	Not tested
CS1-S	Sarcoma	MUC6	p.X2381_splice	Private	11	1013898	C	A	Splice_Site	325	0.381538462	No	1	Clonal	Disease_causing	Passenger	.	passenger	No	No	No	No	Not tested
CS1-C	Carcinoma	MXD4	p.A149V	Private	4	2252837	G	A	Missense_Mutation	88	0.113636364	No	1	Clonal	Neutral	Passenger	Neutral	passenger	No	No	No	No	Not tested
CS1-S	Sarcoma	MYBPC2	p.L743M	Private	19	50958790	C	A	Missense_Mutation	383	0.248041775	No	0.873	Subclonal	Disease_causing	Passenger	Neutral	passenger	No	No	No	No	Not tested
CS1-S	Sarcoma	MYH1	p.C796Y	Private	17	10408528	C	T	Missense_Mutation	386	0.235751295	No	0.83	Subclonal	Disease_causing	Passenger	Neutral	passenger	No	No	No	No	Not tested
CS1-C	Carcinoma	MYH10	p.K1376del	Private	17	8397129	GTTC	G	In_Frame_Del	206	0.199029126	No	1	Clonal	.	.	.	.	No	No	No	No	Not tested
CS1-C	Carcinoma	MYH4	p.A1491V	Private	17	10351994	G	A	Missense_Mutation	368	0.149456522	No	1	Clonal	Disease_causing	Passenger	Neutral	passenger	No	No	No	No	Not tested
CS1-S	Sarcoma	MYLK3	p.M564T	Private	16	46763017	A	G	Missense_Mutation	295	0.277966102	No	0.978	Clonal	Disease_causing	Driver	Neutral	likely_pathogenic	No	No	No	No	Not tested
CS1-S	Sarcoma	MYO15A	p.A2V	Private	17	18022119	C	T	Missense_Mutation	361	0.135734072	No	0.478	Subclonal	Disease_causing	Passenger	Disease_causing	likely_pathogenic	No	No	No	No	Not tested
CS1-C	Carcinoma	MYO18A	p.E1896K	Shared	17	27413980	C	T	Missense_Mutation	105	0.40952381	No	1	Clonal	Disease_causing	Passenger	Disease_causing	likely_pathogenic	No	No	No	No	Not tested
CS1-S	Sarcoma	MYO18A	p.E1896K	Shared	17	27413980	C	T	Missense_Mutation	139	0.474820144	No	1	Clonal	Disease_causing	Passenger	Disease_causing	likely_pathogenic	No	No	No	No	Not tested
CS1-C	Carcinoma	MYO18B	p.E2342K	Private	22	26422964	G	A	Missense_Mutation	327	0.03058104	No	0.292	Subclonal	Neutral	Passenger	Disease_causing	passenger	No	No	No	No	Not tested
CS1-C	Carcinoma	MYO1A	p.K1033Rfs*8	Shared	12	57422572	CT	C	Frame_Shift_Del	313	0.031948882	No	0.305	Subclonal	.	.	.	.	No	No	No	No	Not tested
CS1-S	Sarcoma	MYO1A	p.K1033Rfs*8	Shared	12	57422572	CT	C	Frame_Shift_Del	427	0.353629977	No	1	Clonal	.	.	.	.	No	No	No	No	Not tested
CS1-C	Carcinoma	MYO1B	p.I403V	Private	2	192246209	A	G	Missense_Mutation	206	0.184466019	No	1	Clonal	Disease_causing	Passenger	Neutral	passenger	No	No	No	No	Not tested
CS1-S	Sarcoma	MYO9A	p.G1297D	Private	15	72190954	C	T	Missense_Mutation	437	0.212814645	No	0.749	Subclonal	Disease_causing	Passenger	Disease_causing	likely_pathogenic	No	No	No	No	Not tested
CS1-C	Carcinoma	MYOF	p.T1120A	Private	10	95115430	T	C	Missense_Mutation	173	0.260115607	No	1	Clonal	Neutral	Passenger	Disease_causing	passenger	No	No	No	No	Not tested
CS1-S	Sarcoma	MYOG	p.R84W	Private	1	203054840	G	A	Missense_Mutation	782	0.167519182	No	0.757	Subclonal	Disease_causing	Passenger	Disease_causing	likely_pathogenic	No	No	No	No	Not tested
CS1-S	Sarcoma	MYOM2	p.V895G	Private	8	2050521	T	G	Missense_Mutation	257	0.085603113	No	0.387	Subclonal	Disease_causing	Passenger	Neutral	passenger	No	No	No	No	Not tested
CS1-S	Sarcoma	NAGPA	p.R46Pfs*53	Private	16	5083677	GGC	G	Frame_Shift_Del	159	0.257861635	No	0.907	Clonal	.	.	.	.	No	No	No	No	Not tested
CS1-C	Carcinoma	NALCN	p.R669H	Private	13	101795543	C	T	Missense_Mutation	295	0.240677966	No	1	Clonal	Disease_causing	Passenger	Disease_causing	likely_pathogenic	No	No	No	No	Not tested
CS1-S	Sarcoma	NAPSA	p.M203I	Private	19	50864257	C	A	Missense_Mutation	266	0.116541353	No	0.41	Subclonal	Neutral	Passenger	Neutral	passenger	No	No	No	No	Not tested
CS1-C	Carcinoma	NARG2	p.K633Nfs*17	Private	15	60741266	GT	G	Frame_Shift_Del	88	0.181818182	No	1	Clonal	.	.	.	.	No	No	No	No	Not tested
CS1-C	Carcinoma	NAV1	p.P442S	Private	1	201749646	C	T	Missense_Mutation	314	0.041401274	No	0.437	Subclonal	Disease_causing	Passenger	Neutral	passenger	No	No	No	No	Not tested
CS1-C	Carcinoma	NBEAL2	p.H193N	Private	3	47032729	C	A	Missense_Mutation	359	0.072423398	No	0.692	Subclonal	Disease_causing	Passenger	Neutral	passenger	No	No	No	No	Not tested
CS1-S	Sarcoma	NCAM1	p.N448D	Private	11	113102472	A	G	Missense_Mutation	90	0.4	No	1	Clonal	Disease_causing	Passenger	Disease_causing	likely_pathogenic	No	No	No	No	Not tested
CS1-C	Carcinoma	NCAPD2	p.R465*	Private	12	6626834	C	T	Nonsense_Mutation	292	0.034246575	No	0.327	Subclonal	Neutral	.	.	passenger	No	No	No	No	Not tested
CS1-C	Carcinoma	NCOA2	p.S801N	Private	8	71060711	C	T	Missense_Mutation	271	0.044280443	No	0.423	Subclonal	Disease_causing	Passenger	Neutral	passenger	No	Yes	No	No	Not tested
CS1-C	Carcinoma	NCOR2	p.I976Sfs*87	Private	12	124848227	TG	T	Frame_Shift_Del	150	0.206666667	No	1	Clonal	.	.	.	.	No	Yes	No	No	Not tested
CS1-C	Carcinoma	NCOR2	p.P154Rfs*19	Private	12	124957627	CG	C	Frame_Shift_Del	79	0.189873418	No	1	Clonal	.	.	.	.	No	Yes	No	No	Not tested
CS1-C	Carcinoma	NCSTN	p.R232W	Private	1	160321123	C	T	Missense_Mutation	268	0.156716418	No	1	Clonal	Disease_causing	Passenger	Disease_causing	likely_pathogenic	No	No	No	No	Not tested
CS1-S	Sarcoma	NCSTN	p.G6Vfs*22	Private	1	160313198	AG	A	Frame_Shift_Del	218	0.275229358	No	1	Clonal	.	.	.	.	No	No	No	No	Not tested
CS1-S	Sarcoma	NDUFA10	p.Q299H	Private	2	240929593	C	A	Missense_Mutation	380	0.273684211	No	0.963	Clonal	Disease_causing	Passenger	Neutral	passenger	No	No	No	No	Not tested
CS1-S	Sarcoma	NDUFA7	p.X84_splice	Private	19	8381379	C	T	Splice_Site	176	0.090909091	No	0.32	Subclonal	Disease_causing	.	.	passenger	No	No	No	No	Not tested
CS1-C	Carcinoma	NDUFS4	p.N141Kfs*6	Private	5	52954445	G	GA	Frame_Shift_Ins	209	0.177033493	No	1	Clonal	.	.	.	.	No	No	No	No	Not tested
CS1-S	Sarcoma	NEDD4L	p.N156Mfs*51	Private	18	55990514	CA	C	Frame_Shift_Del	331	0.441087613	No	1	Clonal	.	.	.	.	No	No	No	No	Not tested
CS1-C	Carcinoma	NEURL1	p.A528T	Private	10	105349986	G	A	Missense_Mutation	185	0.032432432	No	0.31	Subclonal	Disease_causing	Passenger	Neutral	passenger	No	No	No	No	Not tested
CS1-S	Sarcoma	NEURL1B	p.A361V	Private	5	172110926	C	T	Missense_Mutation	168	0.077380952	No	0.272	Subclonal	Neutral	Passenger	.	passenger	No	No	No	No	Not tested
CS1-S	Sarcoma	NEURL1B	p.A372V	Private	5	172110959	C	T	Missense_Mutation	138	0.485507246	No	1	Clonal	Neutral	Passenger	.	passenger	No	No	No	No	Not tested
CS1-S	Sarcoma	NEXN	p.E240Nfs*8	Private	1	78392423	CA	C	Frame_Shift_Del	242	0.107438017	No	0.378	Subclonal	.	.	.	.	No	No	No	No	Not tested
CS1-C	Carcinoma	NF1	p.N78Kfs*29	Private	17	29486049	G	GA	Frame_Shift_Ins	164	0.219512195	No	1	Clonal	.	.	.	.	No	Yes	Yes	Yes	Not tested
CS1-S	Sarcoma	NFATC4	p.G273D	Private	14	24839233	G	A	Missense_Mutation	545	0.411009174	No	1	Clonal	Disease_causing	Passenger	Neutral	passenger	No	No	No	No	Not tested
CS1-C	Carcinoma	NFIX	p.S380L	Shared	19	13192554	C	T	Missense_Mutation	187	0.406417112	No	1	Clonal	Disease_causing	Passenger	Neutral	passenger	No	No	No	No	Not tested
CS1-S	Sarcoma	NFIX	p.S380L	Shared	19	13192554	C	T	Missense_Mutation	234	0.423076923	No	1	Clonal	Disease_causing	Passenger	Neutral	passenger	No	No	No	No	Not tested
CS1-C	Carcinoma	NFRKB	p.E60K	Private	11	129758687	C	T	Missense_Mutation	193	0.03626943	No	0.347	Subclonal	Disease_causing	Passenger	.	passenger	No	No	No	No	Not tested
CS1-C	Carcinoma	NGFR	p.Q93*	Private	17	47583729	C	T	Nonsense_Mutation	399	0.245614035	No	1	Clonal	Disease_causing	.	.	passenger	No	No	No	No	Not tested
CS1-C	Carcinoma	NHS	p.P1161S	Private	X	17745770	C	T	Missense_Mutation	320	0.19375	No	1	Clonal	Disease_causing	Passenger	Neutral	passenger	No	No	No	No	Not tested
CS1-C	Carcinoma	NHS	p.V594I	Private	X	17744069	G	A	Missense_Mutation	305	0.239344262	No	1	Clonal	Disease_causing	Passenger	Neutral	passenger	No	No	No	No	Not tested
CS1-S	Sarcoma	NHSL2	p.R74C	Private	X	71131157	C	T	Missense_Mutation	79	0.430379747	No	1	Clonal	Neutral	Passenger	Neutral	passenger	No	No	No	No	Not tested
CS1-S	Sarcoma	NIPA2	p.X47_splice	Private	15	23019857	C	A	Splice_Site	271	0.151291513	No	0.532	Subclonal	Disease_causing	.	.	passenger	No	No	No	No	Not tested
CS1-C	Carcinoma	NIPBL	p.S2458Cfs*4	Private	5	37057396	TCA	T	Frame_Shift_Del	175	0.194285714	No	1	Clonal	.	.	.	.	No	No	No	No	Not tested
CS1-S	Sarcoma	NISCH	p.A229T	Private	3	52507765	G	A	Missense_Mutation	221	0.285067873	No	1	Clonal	Disease_causing	Passenger	Neutral	passenger	No	No	No	No	Not tested
CS1-C	Carcinoma	NKAPL	p.R127Dfs*8	Shared	6	28227518	GGA	G	Frame_Shift_Del	351	0.051282051	No	0.49	Subclonal	.	.	.	.	No	No	No	No	Not tested
CS1-S	Sarcoma	NKAPL	p.R127Dfs*8	Shared	6	28227518	GGA	G	Frame_Shift_Del	482	0.342323651	No	1	Clonal	.	.	.	.	No	No	No	No	Not tested
CS1-C	Carcinoma	NLGN4X	p.F798S	Private	X	5810916	A	G	Missense_Mutation	459	0.080610022	No	0.771	Clonal	Disease_causing	Passenger	Neutral	passenger	No	No	No	No	Not tested
CS1-C	Carcinoma	NLRP1	p.E23G	Private	17	5487210	T	C	Missense_Mutation	273	0.06959707	No	0.665	Subclonal	Neutral	Passenger	Neutral	passenger	No	No	No	No	Not tested
CS1-C	Carcinoma	NNAT	p.N81Tfs*51	Private	20	36151150	GC	G	Frame_Shift_Del	229	0.192139738	No	1	Clonal	.	.	.	.	No	No	No	No	Not tested
CS1-S	Sarcoma	NOD2	p.P528Qfs*235	Private	16	50745398	AC	A	Frame_Shift_Del	557	0.10951526	No	0.385	Subclonal	.	.	.	.	No	No	No	No	Not tested
CS1-C	Carcinoma	NOL9	p.V232A	Private	1	6609680	A	G	Missense_Mutation	194	0.067010309	No	0.641	Subclonal	Neutral	Passenger	Neutral	passenger	No	No	No	No	Not tested
CS1-S	Sarcoma	NOLC1	p.V243Tfs*2	Private	10	103919191	CTG	C	Frame_Shift_Del	360	0.383333333	No	1	Clonal	.	.	.	.	No	No	No	No	Not tested
CS1-S	Sarcoma	NOSTRIN	p.N191Tfs*9	Private	2	169699562	CA	C	Frame_Shift_Del	265	0.173584906	No	0.611	Subclonal	.	.	.	.	No	No	No	No	Not tested
CS1-C	Carcinoma	NOTCH3	p.G165D	Private	19	15302956	C	T	Missense_Mutation	215	0.079069767	No	0.756	Clonal	Disease_causing	Passenger	Disease_causing	likely_pathogenic	No	No	No	No	Not tested
CS1-S	Sarcoma	NOX5	p.H470Tfs*10	Private	15	69331225	CT	C	Frame_Shift_Del	613	0.375203915	No	1	Clonal	.	.	.	.	No	No	No	No	Not tested
CS1-C	Carcinoma	NPAS3	p.C30Y	Private	14	33525149	G	A	Missense_Mutation	176	0.25	No	1	Clonal	Disease_causing	Driver	Neutral	likely_pathogenic	No	No	No	No	Not tested
CS1-C	Carcinoma	NPFFR1	p.S282F	Private	10	72015161	G	A	Missense_Mutation	367	0.223433243	No	1	Clonal	Disease_causing	Passenger	Neutral	passenger	No	No	No	No	Not tested
CS1-S	Sarcoma	NPLOC4	p.A372T	Private	17	79563148	C	T	Missense_Mutation	198	0.388888889	No	1	Clonal	Disease_causing	Passenger	.	passenger	No	No	No	No	Not tested
CS1-S	Sarcoma	NR1D2	p.R516Q	Private	3	24018717	G	A	Missense_Mutation	227	0.40969163	No	1	Clonal	Disease_causing	Passenger	Disease_causing	likely_pathogenic	No	No	No	No	Not tested
CS1-C	Carcinoma	NR1H3	p.H48Pfs*178	Private	11	47281436	G	GC	Frame_Shift_Ins	82	0.304878049	No	1	Clonal	.	.	.	.	No	No	No	No	Not tested
CS1-S	Sarcoma	NR4A1	p.L39*	Private	12	52448183	AC	A	Frame_Shift_Del	551	0.255898367	No	0.9	Clonal	.	.	.	.	No	No	No	No	Not tested
CS1-C	Carcinoma	NR5A2	p.S246N	Private	1	200017573	G	A	Missense_Mutation	638	0.039184953	No	0.414	Subclonal	Disease_causing	Passenger	Disease_causing	likely_pathogenic	No	No	No	No	Not tested
CS1-S	Sarcoma	NRD1	p.W413Gfs*22	Private	1	52290961	CA	C	Frame_Shift_Del	237	0.388185654	No	1	Clonal	.	.	.	.	No	No	No	No	Not tested
CS1-C	Carcinoma	NRSN2	p.G14S	Private	20	330327	G	A	Missense_Mutation	204	0.18627451	No	1	Clonal	Neutral	Passenger	Neutral	passenger	No	No	No	No	Not tested
CS1-C	Carcinoma	NSD1	p.A2300T	Private	5	176721267	G	A	Missense_Mutation	276	0.039855072	No	0.381	Subclonal	Disease_causing	Passenger	Disease_causing	likely_pathogenic	No	Yes	Yes	Yes	Not tested
CS1-C	Carcinoma	NSL1	p.E178Kfs*3	Private	1	212912910	TC	T	Frame_Shift_Del	162	0.135802469	No	1	Clonal	.	.	.	.	No	No	No	No	Not tested
CS1-S	Sarcoma	NSRP1	p.S414Afs*8	Private	17	28512251	GA	G	Frame_Shift_Del	379	0.121372032	No	0.427	Subclonal	.	.	.	.	No	No	No	No	Not tested
CS1-S	Sarcoma	NSUN2	p.G495D	Private	5	6607337	C	T	Missense_Mutation	654	0.24617737	No	0.866	Subclonal	Neutral	Passenger	Neutral	passenger	No	No	No	No	Not tested
CS1-S	Sarcoma	NSUN2	p.W34*	Private	5	6632865	C	T	Nonsense_Mutation	276	0.137681159	No	0.484	Subclonal	Disease_causing	.	.	passenger	No	No	No	No	Not tested
CS1-C	Carcinoma	NSUN3	p.K339Nfs*7	Private	3	93845324	GA	G	Frame_Shift_Del	266	0.195488722	No	1	Clonal	.	.	.	.	No	No	No	No	Not tested
CS1-C	Carcinoma	NSUN7	p.I342*	Private	4	40778259	CA	C	Frame_Shift_Del	117	0.213675214	No	1	Clonal	.	.	.	.	No	No	No	No	Not tested
CS1-S	Sarcoma	NT5C1B	p.V117M	Private	2	18767609	C	T	Missense_Mutation	253	0.434782609	No	1	Clonal	Neutral	Passenger	Disease_causing	passenger	No	No	No	No	Not tested
CS1-S	Sarcoma	NTN1	p.R538C	Private	17	9143082	C	T	Missense_Mutation	425	0.371764706	No	1	Clonal	Disease_causing	Passenger	Neutral	passenger	No	No	No	No	Not tested
CS1-C	Carcinoma	NUDT10	p.A20T	Private	X	51075875	G	A	Missense_Mutation	222	0.036036036	No	0.345	Subclonal	Disease_causing	Passenger	Neutral	passenger	No	No	No	No	Not tested
CS1-C	Carcinoma	NUFIP1	p.C176Lfs*2	Private	13	45554924	C	CA	Frame_Shift_Ins	120	0.241666667	No	1	Clonal	.	.	.	.	No	No	No	No	Not tested
CS1-C	Carcinoma	NUP98	p.R1635*	Private	11	3704445	G	A	Nonsense_Mutation	268	0.055970149	No	0.535	Subclonal	Neutral	.	.	likely_pathogenic	No	Yes	No	No	Not tested
CS1-S	Sarcoma	NXF1	p.R459H	Private	11	62563623	C	T	Missense_Mutation	543	0.390423573	No	1	Clonal	Disease_causing	Driver	Neutral	likely_pathogenic	No	No	No	No	Not tested
CS1-C	Carcinoma	NXNL1	p.A41D	Private	19	17571557	G	T	Missense_Mutation	264	0.068181818	No	0.652	Subclonal	Neutral	Passenger	Disease_causing	passenger	No	No	No	No	Not tested
CS1-S	Sarcoma	NXNL2	p.L103P	Private	9	91159299	T	C	Missense_Mutation	180	0.183333333	No	0.645	Subclonal	Disease_causing	Passenger	Disease_causing	likely_pathogenic	No	No	No	No	Not tested
CS1-S	Sarcoma	OBSCN	p.C3863Wfs*25	Private	1	228476543	CTG	C	Frame_Shift_Del	680	0.135294118	No	0.611	Subclonal	.	.	.	.	No	No	No	No	Not tested
CS1-S	Sarcoma	OBSCN	p.L8028S	Private	1	228559691	T	C	Missense_Mutation	559	0.094812165	No	0.428	Subclonal	Neutral	Passenger	Neutral	passenger	No	No	No	No	Not tested
CS1-S	Sarcoma	OBSL1	p.K511Nfs*26	Private	2	220432440	AT	A	Frame_Shift_Del	319	0.122257053	No	0.43	Subclonal	.	.	.	.	No	No	No	No	Not tested
CS1-C	Carcinoma	ODC1	p.S177N	Private	2	10583882	C	T	Missense_Mutation	365	0.068493151	No	0.723	Subclonal	Disease_causing	Passenger	Neutral	passenger	No	No	No	No	Not tested
CS1-S	Sarcoma	OGDH	p.A660V	Private	7	44736591	C	T	Missense_Mutation	332	0.144578313	No	0.509	Subclonal	Disease_causing	Passenger	Disease_causing	likely_pathogenic	No	No	No	No	Not tested
CS1-C	Carcinoma	OGDHL	p.W640G	Private	10	50950968	A	C	Missense_Mutation	200	0.13	No	1	Clonal	Disease_causing	Passenger	Disease_causing	likely_pathogenic	No	No	No	No	Not tested
CS1-C	Carcinoma	OGT	p.A607V	Private	X	70779496	C	T	Missense_Mutation	165	0.072727273	No	0.695	Subclonal	Disease_causing	Passenger	Neutral	passenger	No	No	No	No	Not tested
CS1-C	Carcinoma	OLFM3	p.D430E	Private	1	102269881	A	T	Missense_Mutation	258	0.189922481	No	1	Clonal	Disease_causing	Passenger	Disease_causing	likely_pathogenic	No	No	No	No	Not tested
CS1-S	Sarcoma	OPCML	p.G177D	Private	11	132307250	C	T	Missense_Mutation	293	0.04778157	No	0.168	Subclonal	Disease_causing	Passenger	Neutral	passenger	No	No	No	No	Not tested
CS1-S	Sarcoma	OPRL1	p.R148H	Private	20	62729364	G	A	Missense_Mutation	635	0.240944882	No	0.848	Subclonal	Disease_causing	Passenger	Disease_causing	likely_pathogenic	No	No	No	No	Not tested
CS1-C	Carcinoma	OR11L1	p.S275Y	Private	1	248004375	G	T	Missense_Mutation	351	0.148148148	No	1	Clonal	Neutral	Passenger	Neutral	passenger	No	No	No	No	Not tested
CS1-C	Carcinoma	OR14C36	p.F105Lfs*28	Private	1	248512383	GT	G	Frame_Shift_Del	560	0.294642857	No	1	Clonal	.	.	.	.	No	No	No	No	Not tested
CS1-C	Carcinoma	OR1L3	p.N172D	Private	9	125437922	A	G	Missense_Mutation	325	0.178461538	No	1	Clonal	Neutral	Passenger	Neutral	passenger	No	No	No	No	Not tested
CS1-S	Sarcoma	OR1L3	p.C179Vfs*7	Private	9	125437935	AT	A	Frame_Shift_Del	462	0.121212121	No	0.427	Subclonal	.	.	.	.	No	No	No	No	Not tested
CS1-C	Carcinoma	OR2J3	p.A148T	Private	6	29080109	G	A	Missense_Mutation	438	0.066210046	No	0.633	Subclonal	Neutral	Passenger	Neutral	passenger	No	No	No	No	Not tested
CS1-S	Sarcoma	OR2T6	p.M143I	Private	1	248551338	G	A	Missense_Mutation	551	0.304900181	No	1	Clonal	Neutral	Passenger	Neutral	passenger	No	No	No	No	Not tested
CS1-C	Carcinoma	OR4D10	p.N95D	Private	11	59245185	A	G	Missense_Mutation	387	0.072351421	No	0.692	Subclonal	Neutral	Passenger	Neutral	passenger	No	No	No	No	Not tested
CS1-S	Sarcoma	OR4P4	p.F249Lfs*39	Private	11	55406574	GT	G	Frame_Shift_Del	181	0.309392265	No	1	Clonal	.	.	.	.	No	No	No	No	Not tested
CS1-S	Sarcoma	OR51E2	p.G151R	Private	11	4703491	C	T	Missense_Mutation	372	0.389784946	No	1	Clonal	Neutral	Passenger	Neutral	passenger	No	No	No	No	Not tested
CS1-C	Carcinoma	OR52E8	p.R268C	Shared	11	5878131	G	A	Missense_Mutation	236	0.368644068	No	1	Clonal	Neutral	Passenger	Neutral	passenger	No	No	No	No	Not tested
CS1-S	Sarcoma	OR52E8	p.P289T	Private	11	5878068	G	T	Missense_Mutation	352	0.130681818	No	0.46	Subclonal	Neutral	Passenger	Neutral	passenger	No	No	No	No	Not tested
CS1-S	Sarcoma	OR52E8	p.R268C	Shared	11	5878131	G	A	Missense_Mutation	314	0.461783439	No	1	Clonal	Neutral	Passenger	Neutral	passenger	No	No	No	No	Not tested
CS1-S	Sarcoma	OR52W1	p.V162del	Private	11	6220931	ATTG	A	In_Frame_Del	455	0.134065934	No	0.472	Subclonal	.	.	.	.	No	No	No	No	Not tested
CS1-S	Sarcoma	OR5AP2	p.Y66H	Private	11	56409720	A	G	Missense_Mutation	290	0.434482759	No	1	Clonal	Disease_causing	Passenger	Neutral	passenger	No	No	No	No	Not tested
CS1-S	Sarcoma	OR5B21	p.A200T	Private	11	58274981	C	T	Missense_Mutation	354	0.033898305	No	0.119	Subclonal	Neutral	Passenger	Neutral	passenger	No	No	No	No	Not tested
CS1-C	Carcinoma	OR5M9	p.G131D	Shared	11	56230486	C	T	Missense_Mutation	377	0.342175066	No	1	Clonal	Neutral	Passenger	Neutral	passenger	No	No	No	No	Not tested
CS1-S	Sarcoma	OR5M9	p.G131D	Shared	11	56230486	C	T	Missense_Mutation	554	0.389891697	No	1	Clonal	Neutral	Passenger	Neutral	passenger	No	No	No	No	Not tested
CS1-C	Carcinoma	OR5P2	p.R289M	Private	11	7817624	C	A	Missense_Mutation	297	0.070707071	No	0.676	Subclonal	Neutral	Passenger	Neutral	passenger	No	No	No	No	Not tested
CS1-S	Sarcoma	OR6B2	p.C291Y	Private	2	240968975	C	T	Missense_Mutation	547	0.12797075	No	0.45	Subclonal	Disease_causing	Passenger	Neutral	passenger	No	No	No	No	Not tested
CS1-S	Sarcoma	OR6C75	p.L200*	Private	12	55759485	AT	A	Frame_Shift_Del	574	0.196864111	No	0.693	Subclonal	.	.	.	.	No	No	No	No	Not tested
CS1-S	Sarcoma	OR6C76	p.A113V	Private	12	55820375	C	T	Missense_Mutation	279	0.032258065	No	0.114	Subclonal	Neutral	Passenger	Neutral	passenger	No	No	No	No	Not tested
CS1-S	Sarcoma	OR6Q1	p.A123T	Private	11	57798791	G	A	Missense_Mutation	466	0.13304721	No	0.468	Subclonal	Disease_causing	Passenger	Neutral	passenger	No	No	No	No	Not tested
CS1-C	Carcinoma	OR6T1	p.L187F	Private	11	123813987	G	A	Missense_Mutation	321	0.093457944	No	0.894	Clonal	Neutral	Passenger	Neutral	passenger	No	No	No	No	Not tested
CS1-S	Sarcoma	ORAI2	p.R249C	Private	7	102087479	C	T	Missense_Mutation	110	0.154545455	No	0.544	Subclonal	Disease_causing	Passenger	Neutral	passenger	No	No	No	No	Not tested
CS1-S	Sarcoma	ORC5	p.H344Tfs*25	Private	7	103805691	G	GT	Frame_Shift_Ins	127	0.37007874	No	1	Clonal	.	.	.	.	No	No	No	No	Not tested
CS1-C	Carcinoma	OSBP2	p.R512C	Private	22	31285534	C	T	Missense_Mutation	332	0.042168675	No	0.403	Subclonal	Disease_causing	Passenger	Neutral	passenger	No	No	No	No	Not tested
CS1-S	Sarcoma	OSBPL1A	p.H670R	Private	18	21758061	T	C	Missense_Mutation	318	0.238993711	No	0.841	Subclonal	Disease_causing	Passenger	Neutral	passenger	No	No	No	No	Not tested
CS1-S	Sarcoma	OSM	p.R233C	Private	22	30659934	G	A	Missense_Mutation	441	0.204081633	No	0.718	Subclonal	Neutral	Passenger	Neutral	passenger	No	No	No	No	Not tested
CS1-S	Sarcoma	OTOF	p.X1764_splice	Private	2	26684949	A	AC	Splice_Site	339	0.244837758	No	0.862	Subclonal	.	.	.	.	No	No	No	No	Not tested
CS1-S	Sarcoma	OTP	p.R24W	Private	5	76933023	G	A	Missense_Mutation	221	0.384615385	No	1	Clonal	Disease_causing	Passenger	Disease_causing	likely_pathogenic	No	No	No	No	Not tested
CS1-S	Sarcoma	OTUD6B	p.A208V	Private	8	92090801	C	T	Missense_Mutation	294	0.108843537	No	0.492	Subclonal	Neutral	Passenger	Neutral	passenger	No	No	No	No	Not tested
CS1-S	Sarcoma	OVCA2	p.E96*	Private	17	1946000	G	T	Nonsense_Mutation	368	0.402173913	No	1	Clonal	Disease_causing	.	.	passenger	No	No	No	No	Not tested
CS1-C	Carcinoma	OXER1	p.A113T	Private	2	42990983	C	T	Missense_Mutation	219	0.068493151	No	0.723	Subclonal	Disease_causing	Passenger	Neutral	passenger	No	No	No	No	Not tested
CS1-C	Carcinoma	P2RY4	p.A297T	Private	X	69478586	C	T	Missense_Mutation	266	0.041353383	No	0.395	Subclonal	Disease_causing	Passenger	Neutral	passenger	No	No	No	No	Not tested
CS1-S	Sarcoma	P4HB	p.N387Tfs*118	Private	17	79803763	GT	G	Frame_Shift_Del	253	0.395256917	No	1	Clonal	.	.	.	.	No	No	No	No	Not tested
CS1-C	Carcinoma	PABPC5	p.K214Nfs*2	Private	X	90691213	GA	G	Frame_Shift_Del	294	0.176870748	No	1	Clonal	.	.	.	.	No	No	No	No	Not tested
CS1-S	Sarcoma	PAF1	p.*532Wext*14	Private	19	39876631	T	C	Nonstop_Mutation	548	0.129562044	No	0.456	Subclonal	Disease_causing	Passenger	.	passenger	No	No	No	No	Not tested
CS1-C	Carcinoma	PAK7	p.Y272C	Private	20	9560967	T	C	Missense_Mutation	269	0.07063197	No	0.675	Subclonal	Disease_causing	Passenger	Neutral	passenger	No	No	No	No	Not tested
CS1-C	Carcinoma	PALB2	p.Q39R	Private	16	23649266	T	C	Missense_Mutation	228	0.052631579	No	0.503	Subclonal	Neutral	Passenger	Neutral	passenger	No	Yes	No	No	Not tested
CS1-S	Sarcoma	PANK4	p.R462C	Private	1	2445896	G	A	Missense_Mutation	193	0.227979275	No	0.802	Subclonal	Disease_causing	Passenger	Neutral	passenger	No	No	No	No	Not tested
CS1-C	Carcinoma	PAPPA	p.C1362Y	Private	9	119115105	G	A	Missense_Mutation	165	0.042424242	No	0.406	Subclonal	Disease_causing	Driver	Neutral	likely_pathogenic	No	No	No	No	Not tested
CS1-S	Sarcoma	PARP4	p.T802A	Private	13	25033202	T	C	Missense_Mutation	245	0.302040816	No	1	Clonal	Disease_causing	Passenger	Neutral	passenger	No	No	No	No	Not tested
CS1-C	Carcinoma	PASD1	p.A335T	Private	X	150832752	G	A	Missense_Mutation	216	0.055555556	No	0.531	Subclonal	Neutral	Passenger	Neutral	passenger	No	No	No	No	Not tested
CS1-S	Sarcoma	PAWR	p.P36H	Private	12	80083918	G	T	Missense_Mutation	399	0.303258145	No	1	Clonal	Neutral	Passenger	Neutral	passenger	No	No	No	No	Not tested
CS1-C	Carcinoma	PAX6	p.P389Hfs*7	Shared	11	31812316	TG	T	Frame_Shift_Del	201	0.054726368	No	0.523	Subclonal	.	.	.	.	No	No	No	No	Not tested
CS1-S	Sarcoma	PAX6	p.P389Hfs*7	Shared	11	31812316	TG	T	Frame_Shift_Del	261	0.180076628	No	0.634	Subclonal	.	.	.	.	No	No	No	No	Not tested
CS1-C	Carcinoma	PBRM1	p.V1139Lfs*16	Private	3	52613111	GAC	G	Frame_Shift_Del	384	0.1640625	No	1	Clonal	.	.	.	.	No	Yes	Yes	Yes	Not tested
CS1-S	Sarcoma	PCDH12	p.K483N	Private	5	141335968	C	A	Missense_Mutation	593	0.038785835	No	0.136	Subclonal	Disease_causing	Passenger	Neutral	passenger	No	No	No	No	Not tested
CS1-C	Carcinoma	PCDH18	p.I356T	Private	4	138452176	A	G	Missense_Mutation	269	0.059479554	No	0.569	Subclonal	Disease_causing	Passenger	Neutral	passenger	No	No	No	No	Not tested
CS1-S	Sarcoma	PCDHAC2	p.I141T	Private	5	140346773	T	C	Missense_Mutation	426	0.131455399	No	0.463	Subclonal	Disease_causing	Passenger	Neutral	passenger	No	No	No	No	Not tested
CS1-C	Carcinoma	PCDHGA10	p.Q528*	Private	5	140794324	C	T	Nonsense_Mutation	586	0.034129693	No	0.326	Subclonal	Neutral	.	.	passenger	No	No	No	No	Not tested
CS1-S	Sarcoma	PCDHGA9	p.G620W	Private	5	140784377	G	T	Missense_Mutation	418	0.282296651	No	0.993	Clonal	.	Passenger	.	passenger	No	No	No	No	Not tested
CS1-C	Carcinoma	PCDHGB1	p.K11R	Private	5	140729859	A	G	Missense_Mutation	387	0.031007752	No	0.296	Subclonal	Neutral	Passenger	Neutral	passenger	No	No	No	No	Not tested
CS1-S	Sarcoma	PCDHGB2	p.R647C	Private	5	140741641	C	T	Missense_Mutation	256	0.375	No	1	Clonal	Disease_causing	Passenger	Neutral	passenger	No	No	No	No	Not tested

CS1-C	Carcinoma	PCDHGB3	p.Y170C	Private	5	140750470	A	G	Missense_Mutation	[illegible]	[illegible]	No	1	Clonal	.	Passenger	.	passenger	No	No	No	No	Not tested
CS1-S	Sarcoma	PCLO	p.V3720Sfs*2	Private	7	82546144	C	CT	Frame_Shift_Ins	[illegible]	[illegible]	No	0.925	Clonal	.	.	.	.	No	No	No	No	Not tested
CS1-S	Sarcoma	PCNT	p.I785V	Private	21	47783593	A	G	Missense_Mutation	618	0.411003236	No	1	Clonal	Neutral	Passenger	Neutral	passenger	No	No	No	No	Not tested
CS1-C	Carcinoma	PCNX	p.V549I	Private	14	71444699	G	A	Missense_Mutation	517	0.044487427	No	0.425	Subclonal	Neutral	Passenger	Neutral	passenger	No	No	No	No	Not tested
CS1-S	Sarcoma	PCNXL2	p.R1409C	Private	1	233190140	G	A	Missense_Mutation	387	0.100775194	No	0.455	Subclonal	Disease_causing	Passenger	Neutral	passenger	No	No	No	No	Not tested
CS1-C	Carcinoma	PCNXL3	p.A106V	Private	11	65384458	C	T	Missense_Mutation	114	0.052631579	No	0.503	Subclonal	Neutral	Passenger	Neutral	passenger	No	No	No	No	Not tested
CS1-C	Carcinoma	PDE11A	p.I558V	Private	2	178681621	T	C	Missense_Mutation	247	0.064777328	No	0.619	Subclonal	Disease_causing	Passenger	Neutral	passenger	No	No	No	No	Not tested
CS1-S	Sarcoma	PDE11A	p.P187Lfs*10	Private	2	178936604	AG	A	Frame_Shift_Del	637	0.368916797	No	1	Clonal	.	.	.	.	No	No	No	No	Not tested
CS1-C	Carcinoma	PDGFB	p.M64T	Private	22	39629499	A	G	Missense_Mutation	166	0.06626506	No	0.634	Subclonal	Neutral	Passenger	Neutral	passenger	No	Yes	No	No	Not tested
CS1-S	Sarcoma	PDGFRB	p.S286L	Private	5	149513226	G	A	Missense_Mutation	333	0.387387387	No	1	Clonal	Neutral	Passenger	Neutral	passenger	No	Yes	No	No	Not tested
CS1-S	Sarcoma	PDHX	p.L28P	Private	11	34938285	T	C	Missense_Mutation	267	0.265917603	No	0.936	Clonal	Neutral	Passenger	Neutral	passenger	No	No	No	No	Not tested
CS1-C	Carcinoma	PDIA2	p.P103T	Shared	16	334494	C	A	Missense_Mutation	113	0.407079646	No	1	Clonal	Neutral	Passenger	Neutral	passenger	No	No	No	No	Not tested
CS1-S	Sarcoma	PDIA2	p.P103T	Shared	16	334494	C	A	Missense_Mutation	145	0.468965517	No	1	Clonal	Neutral	Passenger	Neutral	passenger	No	No	No	No	Not tested
CS1-C	Carcinoma	PDK1	p.V85I	Private	2	173423492	G	A	Missense_Mutation	127	0.267716535	No	1	Clonal	Disease_causing	Passenger	Neutral	passenger	No	No	No	No	Not tested
CS1-S	Sarcoma	PDP2	p.V152A	Private	16	66918642	T	C	Missense_Mutation	427	0.121779859	No	0.429	Subclonal	Disease_causing	Passenger	Neutral	passenger	No	No	No	No	Not tested
CS1-S	Sarcoma	PDZRN3	p.X270_splice	Private	3	73657747	A	G	Splice_Site	133	0.428571429	No	1	Clonal	Disease_causing	.	.	passenger	No	No	No	No	Not tested
CS1-C	Carcinoma	PFAS	p.R1149L	Private	17	8171914	G	T	Missense_Mutation	67	0.23880597	No	1	Clonal	Disease_causing	Driver	Neutral	likely_pathogenic	No	No	No	No	Not tested
CS1-S	Sarcoma	PGC	p.A291V	Private	6	41707580	G	A	Missense_Mutation	312	0.326923077	No	1	Clonal	Neutral	Passenger	Neutral	passenger	No	No	No	No	Not tested
CS1-C	Carcinoma	PGR	p.C347R	Private	11	100998763	A	G	Missense_Mutation	305	0.042622951	No	0.408	Subclonal	Neutral	Passenger	Neutral	passenger	No	No	No	No	Not tested
CS1-C	Carcinoma	PHEX	p.T615Qfs*4	Private	X	22239798	GA	G	Frame_Shift_Del	235	0.2	No	1	Clonal	.	.	.	.	No	No	No	No	Not tested
CS1-C	Carcinoma	PHF2	p.K492Rfs*6	Private	9	96422611	CA	C	Frame_Shift_Del	144	0.173611111	No	1	Clonal	.	.	.	.	No	No	No	No	Not tested
CS1-C	Carcinoma	PHF2	p.V921M	Private	9	96438000	G	A	Missense_Mutation	304	0.046052632	No	0.44	Subclonal	Disease_causing	Passenger	Neutral	passenger	No	No	No	No	Not tested
CS1-C	Carcinoma	PHF8	p.X69_splice	Private	X	54069063	C	T	Splice_Site	205	0.224390244	No	1	Clonal	Disease_causing	.	.	passenger	No	No	No	No	Not tested
CS1-C	Carcinoma	PHKB	p.S704G	Private	16	47694644	A	G	Missense_Mutation	297	0.050505051	No	0.483	Subclonal	Neutral	Passenger	Disease_causing	passenger	No	No	No	No	Not tested
CS1-C	Carcinoma	PHRF1	p.A394T	Shared	11	605146	G	A	Missense_Mutation	245	0.375510204	No	1	Clonal	Disease_causing	Passenger	Neutral	passenger	No	No	No	No	Not tested
CS1-S	Sarcoma	PHRF1	p.A394T	Shared	11	605146	G	A	Missense_Mutation	372	0.454301075	No	1	Clonal	Disease_causing	Passenger	Neutral	passenger	No	No	No	No	Not tested
CS1-S	Sarcoma	PIAS1	p.N239Mfs*47	Private	15	68438920	CA	C	Frame_Shift_Del	202	0.420792079	No	1	Clonal	.	.	.	.	No	No	No	No	Not tested
CS1-S	Sarcoma	PIDD	p.R718W	Private	11	800341	G	A	Missense_Mutation	466	0.040772532	No	0.143	Subclonal	Disease_causing	Passenger	Neutral	passenger	No	No	No	No	Not tested
CS1-C	Carcinoma	PIGT	p.L495M	Private	20	44053218	C	A	Missense_Mutation	264	0.049242424	No	0.471	Subclonal	Disease_causing	Passenger	Neutral	passenger	No	No	No	No	Not tested
CS1-S	Sarcoma	PIK3C2A	p.A153V	Private	11	17190831	G	A	Missense_Mutation	457	0.361050328	No	1	Clonal	Disease_causing	Passenger	Neutral	passenger	No	No	No	No	Not tested
CS1-C	Carcinoma	PIK3C2B	p.R287Pfs*38	Private	1	204438071	C	CG	Frame_Shift_Ins	235	0.136170213	No	1	Clonal	.	.	.	.	No	No	No	No	Not tested
CS1-C	Carcinoma	PIK3CA	p.R88Q	Shared	3	178916876	G	A	Missense_Mutation	392	0.405612245	Yes	1	Clonal	Disease_causing	Driver	Neutral	likely_pathogenic	No	Yes	Yes	Yes	Not tested
CS1-S	Sarcoma	PIK3CA	p.R88Q	Shared	3	178916876	G	A	Missense_Mutation	524	0.446564885	Yes	1	Clonal	Disease_causing	Driver	Neutral	likely_pathogenic	No	Yes	Yes	Yes	Not tested
CS1-S	Sarcoma	PIK3R5	p.T687M	Private	17	8789614	G	A	Missense_Mutation	274	0.310218978	No	1	Clonal	Neutral	Passenger	Neutral	passenger	No	No	No	No	Not tested
CS1-S	Sarcoma	PIM2	p.*312Qext*38	Private	X	48771410	A	G	Nonstop_Mutation	154	0.032467532	No	0.114	Subclonal	Neutral	.	.	passenger	No	No	No	No	Not tested
CS1-S	Sarcoma	PIP5KL1	p.L30Ffs*26	Private	9	130692107	GC	G	Frame_Shift_Del	157	0.337579618	No	1	Clonal	.	.	.	.	No	No	No	No	Not tested
CS1-S	Sarcoma	PISD	p.R332H	Private	22	32015731	C	T	Missense_Mutation	306	0.434640523	No	1	Clonal	Disease_causing	Passenger	.	passenger	No	No	No	No	Not tested
CS1-C	Carcinoma	PIWIL2	p.Q697H	Private	8	22171942	G	T	Missense_Mutation	113	0.044247788	No	0.423	Subclonal	Disease_causing	Passenger	Disease_causing	likely_pathogenic	No	No	No	No	Not tested
CS1-C	Carcinoma	PKDREJ	p.G387C	Shared	22	46658061	C	A	Missense_Mutation	197	0.350253807	No	1	Clonal	Neutral	Passenger	Neutral	passenger	No	No	No	No	Not tested
CS1-S	Sarcoma	PKDREJ	p.G387C	Shared	22	46658061	C	A	Missense_Mutation	297	0.356902357	No	1	Clonal	Neutral	Passenger	Neutral	passenger	No	No	No	No	Not tested
CS1-C	Carcinoma	PKHD1	p.G1233D	Private	6	51890910	C	T	Missense_Mutation	187	0.053475936	No	0.511	Subclonal	Neutral	Passenger	Disease_causing	passenger	No	No	No	No	Not tested
CS1-C	Carcinoma	PLA2G7	p.L123S	Private	6	46684129	A	G	Missense_Mutation	237	0.185654008	No	1	Clonal	Neutral	Passenger	Neutral	passenger	No	No	No	No	Not tested
CS1-C	Carcinoma	PLCB1	p.P800S	Private	20	8721080	C	T	Missense_Mutation	160	0.20625	No	1	Clonal	Disease_causing	Passenger	Neutral	passenger	No	No	No	No	Not tested
CS1-C	Carcinoma	PLCB3	p.R918Afs*95	Private	11	64032516	TC	T	Frame_Shift_Del	68	0.117647059	No	1	Clonal	.	.	.	.	No	No	No	No	Not tested
CS1-C	Carcinoma	PLCE1	p.G1001R	Private	10	96006283	G	A	Missense_Mutation	329	0.030395137	No	0.291	Subclonal	Disease_causing	Passenger	Neutral	passenger	No	No	No	No	Not tested
CS1-C	Carcinoma	PLEC	p.R2705H	Private	8	144996286	C	T	Missense_Mutation	128	0.09375	No	0.896	Clonal	Neutral	Passenger	Neutral	passenger	No	No	No	No	Not tested
CS1-C	Carcinoma	PLEK2	p.L160P	Private	14	67859869	A	G	Missense_Mutation	139	0.223021583	No	1	Clonal	Disease_causing	Passenger	Neutral	passenger	No	No	No	No	Not tested
CS1-C	Carcinoma	PLEKHA6	p.Q288H	Private	1	204228529	C	A	Missense_Mutation	441	0.145124717	No	1	Clonal	Neutral	Passenger	Neutral	passenger	No	No	No	No	Not tested
CS1-C	Carcinoma	PLEKHG4B	p.R805*	Private	5	169459	C	T	Nonsense_Mutation	271	0.036900369	No	0.353	Subclonal	Neutral	.	.	passenger	No	No	No	No	Not tested
CS1-C	Carcinoma	PLEKHG5	p.T1081A	Private	1	6527892	T	C	Missense_Mutation	129	0.062015504	No	0.593	Subclonal	Disease_causing	Passenger	Neutral	passenger	No	No	No	No	Not tested
CS1-C	Carcinoma	PLIN1	p.P477L	Private	15	90208953	G	A	Missense_Mutation	89	0.056179775	No	0.537	Subclonal	Neutral	Passenger	Neutral	passenger	No	No	No	No	Not tested
CS1-S	Sarcoma	PLXNA1	p.Y914C	Private	3	126733457	A	G	Missense_Mutation	561	0.11942959	No	0.42	Subclonal	Disease_causing	Passenger	Neutral	passenger	No	No	No	No	Not tested
CS1-S	Sarcoma	PLXNB1	p.N96K	Private	3	48465733	G	T	Missense_Mutation	251	0.163346614	No	0.575	Subclonal	Disease_causing	Passenger	Neutral	passenger	No	No	No	No	Not tested
CS1-C	Carcinoma	PLXNB3	p.V1678Cfs*7	Shared	X	153042690	AG	A	Frame_Shift_Del	153	0.235294118	No	1	Clonal	.	.	.	.	No	No	No	No	Not tested
CS1-S	Sarcoma	PLXNB3	p.V1678Cfs*7	Shared	X	153042690	AG	A	Frame_Shift_Del	228	0.302631579	No	1	Clonal	.	.	.	.	No	No	No	No	Not tested
CS1-S	Sarcoma	PLXND1	p.N675Tfs*5	Private	3	129303234	TG	T	Frame_Shift_Del	314	0.385350318	No	1	Clonal	.	.	.	.	No	No	No	No	Not tested
CS1-C	Carcinoma	PNPLA6	p.R662H	Private	19	7615414	G	A	Missense_Mutation	142	0.063380282	No	0.606	Subclonal	Disease_causing	Passenger	Disease_causing	likely_pathogenic	No	No	No	No	Not tested
CS1-C	Carcinoma	PNPLA7	p.A383T	Shared	9	140409909	C	T	Missense_Mutation	76	0.421052632	No	1	Clonal	Neutral	Passenger	Neutral	passenger	No	No	No	No	Not tested
CS1-S	Sarcoma	PNPLA7	p.A383T	Shared	9	140409909	C	T	Missense_Mutation	110	0.436363636	No	1	Clonal	Neutral	Passenger	Neutral	passenger	No	No	No	No	Not tested
CS1-C	Carcinoma	PODN	p.L433I	Private	1	53544335	C	A	Missense_Mutation	228	0.035087719	No	0.335	Subclonal	Disease_causing	Passenger	Neutral	passenger	No	No	No	No	Not tested
CS1-C	Carcinoma	POGZ	p.X857_splice	Private	1	151379081	A	G	Splice_Site	195	0.123076923	No	1	Clonal	Disease_causing	.	.	passenger	No	No	No	No	Not tested
CS1-C	Carcinoma	POLD1	p.D502N	Private	19	50910249	G	A	Missense_Mutation	116	0.24137931	No	1	Clonal	Disease_causing	Passenger	Neutral	passenger	No	No	No	No	Not tested
CS1-C	Carcinoma	POLE	p.E1190D	Private	12	133233734	C	A	Missense_Mutation	177	0.062146893	No	0.594	Subclonal	Disease_causing	Passenger	Neutral	passenger	No	Yes	No	Yes	Not tested
CS1-C	Carcinoma	POLR2A	p.L26M	Private	17	7388159	C	A	Missense_Mutation	418	0.208133971	No	1	Clonal	Disease_causing	Passenger	Neutral	passenger	No	No	No	No	Not tested
CS1-S	Sarcoma	POMGNT1	p.X404_splice	Private	1	46658263	CTG	C	Splice_Site	229	0.126637555	No	0.446	Subclonal	.	.	.	.	No	No	No	No	Not tested
CS1-C	Carcinoma	PORCN	p.V115A	Private	X	48370294	T	C	Missense_Mutation	248	0.072580645	No	0.694	Subclonal	Neutral	Passenger	Disease_causing	passenger	No	No	No	No	Not tested
CS1-S	Sarcoma	POU6F2	p.A158V	Private	7	39247181	C	T	Missense_Mutation	342	0.210526316	No	0.741	Subclonal	Disease_causing	Passenger	Disease_causing	likely_pathogenic	No	No	No	No	Not tested
CS1-S	Sarcoma	PPARG	p.R308H	Private	3	12458306	G	A	Missense_Mutation	223	0.246636771	No	0.868	Subclonal	Disease_causing	Passenger	Neutral	passenger	No	Yes	No	No	Not tested
CS1-S	Sarcoma	PPFIA1	p.D288G	Private	11	70172857	A	G	Missense_Mutation	339	0.032448378	No	0.114	Subclonal	Disease_causing	Passenger	Neutral	passenger	No	No	No	No	Not tested
CS1-S	Sarcoma	PPFIA1	p.K861Sfs*27	Private	11	70202353	CA	C	Frame_Shift_Del	207	0.178743961	No	0.629	Subclonal	.	.	.	.	No	No	No	No	Not tested
CS1-S	Sarcoma	PPFIA1	p.X861_splice	Private	11	70208200	G	T	Splice_Site	262	0.129770992	No	0.457	Subclonal	Disease_causing	.	.	passenger	No	No	No	No	Not tested
CS1-S	Sarcoma	PPFIA4	p.R665H	Private	1	203044823	G	A	Missense_Mutation	440	0.204545455	No	0.924	Clonal	Disease_causing	Passenger	Neutral	passenger	No	No	No	No	Not tested
CS1-C	Carcinoma	PPFIBP2	p.R774M	Private	11	7672960	G	T	Missense_Mutation	310	0.080645161	No	0.771	Clonal	Disease_causing	Passenger	Disease_causing	likely_pathogenic	No	No	No	No	Not tested
CS1-S	Sarcoma	PPFIBP2	p.R814M	Private	11	7674259	G	T	Missense_Mutation	117	0.170940171	No	0.602	Subclonal	Disease_causing	Passenger	Neutral	passenger	No	No	No	No	Not tested
CS1-S	Sarcoma	PPP1R26	p.V898Sfs*56	Private	9	138379042	AG	A	Frame_Shift_Del	93	0.397849462	No	1	Clonal	.	.	.	.	No	No	No	No	Not tested
CS1-C	Carcinoma	PPP1R3D	p.R214C	Private	20	58514347	G	A	Missense_Mutation	383	0.039164491	No	0.374	Subclonal	Disease_causing	Passenger	Neutral	passenger	No	No	No	No	Not tested
CS1-S	Sarcoma	PPP1R3D	p.T216A	Private	20	58514341	T	C	Missense_Mutation	611	0.111292962	No	0.392	Subclonal	Neutral	Passenger	Neutral	passenger	No	No	No	No	Not tested
CS1-S	Sarcoma	PPRC1	p.P1461Hfs*79	Private	10	103907124	TC	T	Frame_Shift_Del	510	0.11372549	No	0.4	Subclonal	.	.	.	.	No	No	No	No	Not tested
CS1-S	Sarcoma	PQLC3	p.V99I	Private	2	11300819	G	A	Missense_Mutation	352	0.298295455	No	1	Clonal	Neutral	Passenger	Neutral	passenger	No	No	No	No	Not tested
CS1-C	Carcinoma	PRAME	p.M220I	Private	22	22892441	C	T	Missense_Mutation	290	0.193103448	No	1	Clonal	Neutral	Passenger	Neutral	passenger	No	No	No	No	Not tested
CS1-S	Sarcoma	PRC1	p.Q457Tfs*16	Private	15	91517458	G	GT	Frame_Shift_Ins	137	0.445255474	No	1	Clonal	.	.	.	.	No	No	No	No	Not tested
CS1-S	Sarcoma	PRDM10	p.C677R	Private	11	129793160	A	G	Missense_Mutation	60	0.116666667	No	0.411	Subclonal	Disease_causing	Passenger	Disease_causing	likely_pathogenic	No	No	No	No	Not tested
CS1-C	Carcinoma	PRDM4	p.H173Y	Private	12	108145801	G	A	Missense_Mutation	485	0.037113402	No	0.355	Subclonal	Disease_causing	Passenger	Disease_causing	likely_pathogenic	No	No	No	No	Not tested
CS1-S	Sarcoma	PRG4	p.P139Tfs*8	Private	1	186273331	C	CA	Frame_Shift_Ins	438	0.205479452	No	0.929	Clonal	.	.	.	.	No	No	No	No	Not tested
CS1-C	Carcinoma	PRKAR1B	p.T214N	Private	7	635850	G	T	Missense_Mutation	249	0.060240964	No	0.576	Subclonal	Disease_causing	Passenger	Disease_causing	likely_pathogenic	No	No	No	No	Not tested
CS1-C	Carcinoma	PRKCE	p.P153Q	Private	2	46203613	C	A	Missense_Mutation	112	0.133928571	No	1	Clonal	Disease_causing	Driver	Disease_causing	likely_pathogenic	No	No	No	No	Not tested
CS1-C	Carcinoma	PRKDC	p.N3604Kfs*3	Private	8	48701554	G	GT	Frame_Shift_Ins	131	0.198473282	No	1	Clonal	.	.	.	.	No	No	No	No	Not tested
CS1-C	Carcinoma	PROKR2	p.R264C	Private	20	5283051	G	A	Missense_Mutation	432	0.081018519	No	0.775	Clonal	Disease_causing	Passenger	Neutral	passenger	No	No	No	No	Not tested
CS1-S	Sarcoma	PROSER1	p.G128Afs*8	Private	13	39600510	GC	G	Frame_Shift_Del	244	0.352459016	No	1	Clonal	.	.	.	.	No	No	No	No	Not tested
CS1-C	Carcinoma	PROX1	p.Q289H	Private	1	214170745	G	T	Missense_Mutation	743	0.055181696	No	0.583	Subclonal	Disease_causing	Passenger	Neutral	passenger	No	No	No	No	Not tested
CS1-S	Sarcoma	PRPF19	p.G494D	Private	11	60658672	C	T	Missense_Mutation	221	0.248868778	No	0.876	Subclonal	Disease_causing	Passenger	Neutral	passenger	No	No	No	No	Not tested
CS1-C	Carcinoma	PRPF4	p.G419C	Private	9	116053086	G	T	Missense_Mutation	284	0.038732394	No	0.37	Subclonal	Disease_causing	Passenger	Disease_causing	likely_pathogenic	No	No	No	No	Not tested
CS1-S	Sarcoma	PRPF6	p.R599M	Private	20	62655934	G	T	Missense_Mutation	272	0.238970588	No	0.841	Subclonal	Disease_causing	Driver	Neutral	likely_pathogenic	No	No	No	No	Not tested
CS1-C	Carcinoma	PRPF8	p.V668Wfs*5	Private	17	1580448	AC	A	Frame_Shift_Del	150	0.226666667	No	1	Clonal	.	.	.	.	No	No	No	No	Not tested
CS1-C	Carcinoma	PRR14L	p.N217Mfs*4	Private	22	32113174	AT	A	Frame_Shift_Del	279	0.204301075	No	1	Clonal	.	.	.	.	No	No	No	No	Not tested
CS1-C	Carcinoma	PRR9	p.C12Y	Private	1	153190655	G	A	Missense_Mutation	280	0.142857143	No	1	Clonal	Disease_causing	Passenger	.	passenger	No	No	No	No	Not tested
CS1-S	Sarcoma	PRSS23	p.C370Y	Private	11	86519794	G	A	Missense_Mutation	300	0.133333333	No	0.469	Subclonal	Disease_causing	Passenger	Neutral	passenger	No	No	No	No	Not tested
CS1-C	Carcinoma	PRSS37	p.A109V	Private	7	141537764	G	A	Missense_Mutation	368	0.065217391	No	0.624	Subclonal	Disease_causing	Passenger	Disease_causing	likely_pathogenic	No	No	No	No	Not tested
CS1-S	Sarcoma	PRUNE2	p.P879H	Private	9	79324554	G	T	Missense_Mutation	274	0.167883212	No	0.591	Subclonal	Disease_causing	Passenger	Neutral	passenger	No	No	No	No	Not tested
CS1-S	Sarcoma	PRUNE2	p.N2824D	Private	9	79267486	T	C	Missense_Mutation	443	0.408577878	No	1	Clonal	Disease_causing	Passenger	Neutral	passenger	No	No	No	No	Not tested
CS1-C	Carcinoma	PSAPL1	p.R373Q	Private	4	7435489	C	T	Missense_Mutation	397	0.062972292	No	0.602	Subclonal	Neutral	Passenger	Neutral	passenger	No	No	No	No	Not tested
CS1-C	Carcinoma	PSD3	p.N234H	Private	8	18729674	T	G	Missense_Mutation	282	0.095744681	No	0.915	Clonal	Neutral	Passenger	Neutral	passenger	No	No	No	No	Not tested
CS1-C	Carcinoma	PSMD1	p.G152D	Private	2	231931770	G	A	Missense_Mutation	202	0.064356436	No	0.615	Subclonal	Disease_causing	Passenger	Neutral	passenger	No	No	No	No	Not tested
CS1-S	Sarcoma	PSMD2	p.R478K	Private	3	184021844	G	A	Missense_Mutation	529	0.039697543	No	0.14	Subclonal	Disease_causing	Passenger	Neutral	passenger	No	No	No	No	Not tested
CS1-S	Sarcoma	PSMD6	p.F288Lfs*9	Private	3	63999245	CA	C	Frame_Shift_Del	304	0.213815789	No	0.752	Subclonal	.	.	.	.	No	No	No	No	Not tested
CS1-C	Carcinoma	PSTPIP1	p.E339K	Private	15	77328172	G	A	Missense_Mutation	96	0.229166667	No	1	Clonal	Neutral	Passenger	Neutral	passenger	No	No	No	No	Not tested
CS1-S	Sarcoma	PTBP3	p.G469S	Private	9	114989752	C	T	Missense_Mutation	505	0.061386139	No	0.216	Subclonal	Disease_causing	Passenger	Neutral	passenger	No	No	No	No	Not tested
CS1-C	Carcinoma	PTCD2	p.T365K	Private	5	71654181	C	A	Missense_Mutation	228	0.070175439	No	0.671	Subclonal	Neutral	Passenger	Neutral	passenger	No	No	No	No	Not tested
CS1-C	Carcinoma	PTCHD1	p.Q622Sfs*13	Private	X	23411493	G	GC	Frame_Shift_Ins	418	0.193779904	No	1	Clonal	.	.	.	.	No	No	No	No	Not tested
CS1-C	Carcinoma	PTEN	p.R130G	Shared	10	89692904	C	G	Missense_Mutation	385	0.768831169	Yes	1	Clonal	Disease_causing	Driver	Disease_causing	likely_pathogenic	No	Yes	Yes	Yes	Not tested
CS1-S	Sarcoma	PTEN	p.R130G	Shared	10	89692904	C	G	Missense_Mutation	549	0.821493625	Yes	1	Clonal	Disease_causing	Driver	Disease_causing	likely_pathogenic	No	Yes	Yes	Yes	Not tested
CS1-C	Carcinoma	PTGER3	p.G238W	Private	1	71512549	C	A	Missense_Mutation	376	0.031914894	No	0.305	Subclonal	Disease_causing	Passenger	Neutral	passenger	No	No	No	No	Not tested
CS1-S	Sarcoma	PTGR1	p.W109*	Private	9	114348328	C	T	Nonsense_Mutation	143	0.062937063	No	0.221	Subclonal	Disease_causing	.	.	passenger	No	No	No	No	Not tested
CS1-S	Sarcoma	PTK2B	p.R422H	Private	8	27293789	G	A	Missense_Mutation	365	0.189041096	No	0.854	Subclonal	Disease_causing	Driver	Neutral	likely_pathogenic	No	No	No	No	Not tested
CS1-C	Carcinoma	PTPDC1	p.A217V	Private	9	96857638	C	T	Missense_Mutation	263	0.247148289	No	1	Clonal	Disease_causing	Passenger	Neutral	passenger	No	No	No	No	Not tested
CS1-S	Sarcoma	PTPN23	p.K608Sfs*5	Private	3	47450927	GA	G	Frame_Shift_Del	202	0.371287129	No	1	Clonal	.	.	.	.	No	No	No	No	Not tested
CS1-C	Carcinoma	PTPN3	p.V40I	Private	9	112225597	C	T	Missense_Mutation	184	0.206521739	No	1	Clonal	Disease_causing	Passenger	Neutral	passenger	No	No	No	No	Not tested
CS1-S	Sarcoma	PTPRB	p.N1549D	Private	12	70953192	T	C	Missense_Mutation	476	0.134453782	No	0.473	Subclonal	Neutral	Passenger	Neutral	passenger	No	Yes	No	No	Not tested
CS1-C	Carcinoma	PTPRK	p.E815G	Private	6	128326279	T	C	Missense_Mutation	139	0.050359712	No	0.481	Subclonal	Disease_causing	Driver	Neutral	likely_pathogenic	No	Yes	No	No	Not tested
CS1-C	Carcinoma	PTPRM	p.R767M	Private	18	8143777	G	T	Missense_Mutation	198	0.176767677	No	1	Clonal	Disease_causing	Driver	Neutral	likely_pathogenic	No	No	No	No	Not tested
CS1-S	Sarcoma	PTPRS	p.V1025Sfs*18	Private	19	5222730	CG	C	Frame_Shift_Del	119	0.369747899	No	1	Clonal	.	.	.	.	No	No	No	No	Not tested
CS1-C	Carcinoma	PTPRT	p.R1046C	Private	20	40743859	G	A	Missense_Mutation	254	0.188976378	No	1	Clonal	Disease_causing	Passenger	Neutral	passenger	No	Yes	No	No	Not tested
CS1-C	Carcinoma	PXDN	p.C196Y	Private	2	1684108	C	T	Missense_Mutation	229	0.074235808	No	0.784	Clonal	Disease_causing	Passenger	Neutral	passenger	No	No	No	No	Not tested
CS1-S	Sarcoma	QPCTL	p.H290R	Private	19	46202141	A	G	Missense_Mutation	266	0.244360902	No	0.86	Subclonal	Neutral	Passenger	Neutral	passenger	No	No	No	No	Not tested
CS1-C	Carcinoma	QRFPR	p.V79I	Private	4	122301568	C	T	Missense_Mutation	364	0.076923077	No	0.735	Subclonal	Disease_causing	Passenger	Neutral	passenger	No	No	No	No	Not tested
CS1-C	Carcinoma	RAB11FIP4	p.X188_splice	Private	17	29844896	G	A	Splice_Site	303	0.052805281	No	0.505	Subclonal	Disease_causing	.	.	passenger	No	No	No	No	Not tested
CS1-S	Sarcoma	RAB12	p.A187V	Private	18	8636294	C	T	Missense_Mutation	456	0.243421053	No	0.857	Subclonal	Disease_causing	Passenger	Disease_causing	likely_pathogenic	No	No	No	No	Not tested
CS1-C	Carcinoma	RABGGTA	p.N496D	Private	14	24735705	T	C	Missense_Mutation	205	0.063414634	No	0.606	Subclonal	Disease_causing	Passenger	Disease_causing	likely_pathogenic	No	No	No	No	Not tested
CS1-C	Carcinoma	RAD52	p.A85V	Private	12	1039243	G	A	Missense_Mutation	219	0.068493151	No	0.655	Subclonal	Disease_causing	Passenger	Neutral	passenger	No	No	No	No	Not tested
CS1-S	Sarcoma	RAD9B	p.A321Sfs*9	Private	12	110960044	C	CA	Frame_Shift_Ins	303	0.118811881	No	0.418	Subclonal	.	.	.	.	No	No	No	No	Not tested
CS1-C	Carcinoma	RAE1	p.P248Rfs*29	Private	20	55948625	AC	A	Frame_Shift_Del	236	0.190677966	No	1	Clonal	.	.	.	.	No	No	No	No	Not tested
CS1-S	Sarcoma	RALGAPA2	p.Y1040Lfs*51	Private	20	20552139	T	TA	Frame_Shift_Ins	121	0.247933884	No	0.872	Subclonal	.	.	.	.	No	No	No	No	Not tested
CS1-S	Sarcoma	RANBP6	p.N973Mfs*12	Private	9	6012689	AT	A	Frame_Shift_Del	341	0.384164223	No	1	Clonal	.	.	.	.	No	No	No	No	Not tested
CS1-S	Sarcoma	RAP2B	p.D38G	Private	3	152880595	A	G	Missense_Mutation	493	0.261663286	No	0.921	Clonal	Disease_causing	Passenger	Disease_causing	likely_pathogenic	No	No	No	No	Not tested
CS1-S	Sarcoma	RAPGEF1	p.A284T	Private	9	134504535	C	T	Missense_Mutation	396	0.429292929	No	1	Clonal	Disease_causing	Passenger	Neutral	passenger	No	No	No	No	Not tested
CS1-S	Sarcoma	RAPGEF4	p.F847I	Private	2	173891968	T	A	Missense_Mutation	131	0.145038168	No	0.51	Subclonal	Disease_causing	Passenger	Neutral	passenger	No	No	No	No	Not tested
CS1-S	Sarcoma	RAPGEF5	p.K366Rfs*22	Private	7	22197473	CT	C	Frame_Shift_Del	249	0.148594378	No	0.523	Subclonal	.	.	.	.	No	No	No	No	Not tested
CS1-S	Sarcoma	RAPGEFL1	p.S126R	Private	17	38345507	A	C	Missense_Mutation	282	0.074468085	No	0.262	Subclonal	Disease_causing	Passenger	Neutral	passenger	No	No	No	No	Not tested
CS1-C	Carcinoma	RASIP1	p.R491H	Private	19	49232555	C	T	Missense_Mutation	150	0.22	No	1	Clonal	Disease_causing	Passenger	Neutral	passenger	No	No	No	No	Not tested
CS1-C	Carcinoma	RBM10	p.H791R	Private	X	47045131	A	G	Missense_Mutation	377	0.039787798	No	0.38	Subclonal	Disease_causing	Passenger	Neutral	passenger	No	Yes	No	Yes	Not tested
CS1-S	Sarcoma	RBM10	p.E119del	Private	X	47030560	GGGA	G	In_Frame_Del	210	0.238095238	No	0.838	Subclonal	.	.	.	.	No	Yes	No	Yes	Not tested
CS1-C	Carcinoma	RBM24	p.P227H	Private	6	17292319	C	A	Missense_Mutation	321	0.065420561	No	0.625	Subclonal	Disease_causing	Passenger	Neutral	passenger	No	No	No	No	Not tested
CS1-S	Sarcoma	RBM48	p.P196Q	Private	7	92163854	C	A	Missense_Mutation	334	0.032934132	No	0.116	Subclonal	Neutral	Passenger	.	passenger	No	No	No	No	Not tested
CS1-C	Carcinoma	RCOR3	p.R203H	Private	1	211451550	G	A	Missense_Mutation	431	0.139211137	No	1	Clonal	Disease_causing	Passenger	Neutral	passenger	No	No	No	No	Not tested
CS1-C	Carcinoma	RDH5	p.A240Gfs*19	Private	12	56117811	T	TG	Frame_Shift_Ins	169	0.177514793	No	1	Clonal	.	.	.	.	No	No	No	No	Not tested
CS1-S	Sarcoma	REC8	p.P275Rfs*142	Private	14	24646920	AC	A	Frame_Shift_Del	118	0.423728814	No	1	Clonal	.	.	.	.	No	No	No	No	Not tested
CS1-S	Sarcoma	REEP3	p.F45Lfs*6	Private	10	65354562	GT	G	Frame_Shift_Del	236	0.25	No	0.88	Subclonal	.	.	.	.	No	No	No	No	Not tested
CS1-S	Sarcoma	RERE	p.S1084Vfs*19	Private	1	8420317	A	AC	Frame_Shift_Ins	93	0.365591398	No	1	Clonal	.	.	.	.	No	No	No	No	Not tested
CS1-C	Carcinoma	REV1	p.K1075N	Private	2	100019511	C	G	Missense_Mutation	363	0.052341598	No	0.5	Subclonal	Neutral	Passenger	Neutral	passenger	No	No	No	No	Not tested
CS1-S	Sarcoma	REV1	p.M392Nfs*12	Private	2	100055101	A	AT	Frame_Shift_Ins	394	0.319796954	No	1	Clonal	.	.	.	.	No	No	No	No	Not tested
CS1-S	Sarcoma	REXO1	p.A317T	Private	19	1827839	C	T	Missense_Mutation	242	0.454545455	No	1	Clonal	Neutral	Passenger	Neutral	passenger	No	No	No	No	Not tested
CS1-C	Carcinoma	REXO4	p.K370R	Private	9	136272971	T	C	Missense_Mutation	184	0.157608696	No	1	Clonal	Neutral	Passenger	Neutral	passenger	No	No	No	No	Not tested
CS1-S	Sarcoma	RGL2	p.G17Efs*4	Private	6	33266342	CG	C	Frame_Shift_Del	295	0.118644068	No	0.417	Subclonal	.	.	.	.	No	No	No	No	Not tested
CS1-C	Carcinoma	RGS17	p.T58Yfs*28	Private	6	153347624	AGT	A	Frame_Shift_Del	120	0.208333333	No	1	Clonal	.	.	.	.	No	No	No	No	Not tested
CS1-S	Sarcoma	RHOXF1	p.P81S	Private	X	119249532	G	A	Missense_Mutation	390	0.320512821	No	1	Clonal	Neutral	Passenger	Disease_causing	passenger	No	No	No	No	Not tested
CS1-S	Sarcoma	RIMS1	p.D1611*	Private	6	73108761	G	GT	Frame_Shift_Ins	395	0.149367089	No	0.526	Subclonal	.	.	.	.	No	No	No	No	Not tested
CS1-C	Carcinoma	RMDN3	p.S44N	Private	15	41046851	C	T	Missense_Mutation	179	0.055865922	No	0.534	Subclonal	Neutral	Passenger	Neutral	passenger	No	No	No	No	Not tested
CS1-C	Carcinoma	RND3	p.R234*	Private	2	151326536	G	A	Nonsense_Mutation	133	0.052631579	No	0.503	Subclonal	Disease_causing	.	.	passenger	No	No	No	No	Not tested
CS1-C	Carcinoma	RNF167	p.A231Hfs*5	Shared	17	4847869	CTG	C	Frame_Shift_Del	80	0.5375	No	1	Clonal	.	.	.	.	No	No	No	No	Not tested
CS1-S	Sarcoma	RNF167	p.A231Hfs*5	Shared	17	4847869	CTG	C	Frame_Shift_Del	89	0.370786517	No	1	Clonal	.	.	.	.	No	No	No	No	Not tested
CS1-C	Carcinoma	RNF213	p.A4776T	Private	17	78358842	G	A	Missense_Mutation	261	0.210727969	No	1	Clonal	Neutral	Passenger	Neutral	passenger	No	Yes	No	No	Not tested
CS1-C	Carcinoma	RNF223	p.R232Gfs*15	Private	1	1007252	CG	C	Frame_Shift_Del	119	0.235294118	No	1	Clonal	.	.	.	.	No	No	No	No	Not tested
CS1-S	Sarcoma	RNF31	p.R577Q	Private	14	24620581	G	A	Missense_Mutation	571	0.152364273	No	0.536	Subclonal	Disease_causing	Passenger	Neutral	passenger	No	No	No	No	Not tested
CS1-C	Carcinoma	RNF40	p.R235S	Private	16	30776312	C	A	Missense_Mutation	60	0.283333333	No	1	Clonal	Neutral	Passenger	Neutral	passenger	No	No	No	No	Not tested
CS1-S	Sarcoma	RNF43	p.G659Vfs*41	Private	17	56435160	AC	A	Frame_Shift_Del	325	0.467692308	No	1	Clonal	.	.	.	.	No	Yes	No	No	Not tested
CS1-C	Carcinoma	ROBO2	p.L142V	Shared	3	77147479	C	G	Missense_Mutation	290	0.362068966	No	1	Clonal	Disease_causing	Passenger	Neutral	passenger	No	No	No	No	Not tested
CS1-S	Sarcoma	ROBO2	p.L142V	Shared	3	77147479	C	G	Missense_Mutation	410	0.436585366	No	1	Clonal	Disease_causing	Passenger	Neutral	passenger	No	No	No	No	Not tested
CS1-C	Carcinoma	ROS1	p.Y1262H	Private	6	117679037	A	G	Missense_Mutation	150	0.233333333	No	1	Clonal	Neutral	Passenger	Disease_causing	passenger	No	Yes	No	No	Not tested
CS1-S	Sarcoma	ROS1	p.A1596T	Private	6	117662679	C	T	Missense_Mutation	414	0.241545894	No	0.85	Subclonal	Disease_causing	Passenger	Neutral	passenger	No	Yes	No	No	Not tested
CS1-C	Carcinoma	RP1L1	p.R385Q	Private	8	10470454	C	T	Missense_Mutation	400	0.23	No	1	Clonal	Neutral	Passenger	Neutral	passenger	No	No	No	No	Not tested
CS1-C	Carcinoma	RPA1	p.R31H	Private	17	1747221	G	A	Missense_Mutation	92	0.108695652	No	1	Clonal	Disease_causing	Passenger	Neutral	passenger	No	No	No	No	Not tested
CS1-S	Sarcoma	RPF2	p.R176H	Private	6	111336990	G	A	Missense_Mutation	207	0.260869565	No	0.918	Clonal	Disease_causing	Passenger	Neutral	passenger	No	No	No	No	Not tested
CS1-S	Sarcoma	RPL7L1	p.V182I	Private	6	42854005	G	A	Missense_Mutation	221	0.095022624	No	0.334	Subclonal	Neutral	Passenger	.	passenger	No	No	No	No	Not tested
CS1-C	Carcinoma	RPTOR	p.A1125S	Private	17	78931426	G	T	Missense_Mutation	159	0.056603774	No	0.541	Subclonal	Disease_causing	Driver	Neutral	likely_pathogenic	No	No	No	No	Not tested
CS1-S	Sarcoma	RQCD1	p.L119Cfs*29	Private	2	219449363	CT	C	Frame_Shift_Del	112	0.232142857	No	0.817	Subclonal	.	.	.	.	No	No	No	No	Not tested
CS1-C	Carcinoma	RREB1	p.Q1009*	Private	6	7231357	C	T	Nonsense_Mutation	269	0.037174721	No	0.355	Subclonal	Neutral	.	.	passenger	No	No	No	No	Not tested
CS1-C	Carcinoma	RREB1	p.R727H	Private	6	7230512	G	A	Missense_Mutation	422	0.241706161	No	1	Clonal	Disease_causing	Passenger	Neutral	passenger	No	No	No	No	Not tested
CS1-C	Carcinoma	RSBN1	p.V460I	Private	1	114320430	C	T	Missense_Mutation	141	0.226950355	No	1	Clonal	Disease_causing	Passenger	.	passenger	No	No	No	No	Not tested
CS1-C	Carcinoma	RTCB	p.G365R	Private	22	32791099	C	T	Missense_Mutation	230	0.039130435	No	0.374	Subclonal	Disease_causing	Passenger	Neutral	passenger	No	No	No	No	Not tested
CS1-C	Carcinoma	RTKN	p.L483M	Private	2	74653615	G	T	Missense_Mutation	83	0.265060241	No	1	Clonal	Disease_causing	Passenger	Neutral	passenger	No	No	No	No	Not tested
CS1-S	Sarcoma	RTN1	p.A630T	Private	14	60074088	C	T	Missense_Mutation	240	0.283333333	No	0.997	Clonal	Disease_causing	Passenger	Neutral	passenger	No	No	No	No	Not tested
CS1-S	Sarcoma	RTN1	p.V753M	Private	14	60069814	C	T	Missense_Mutation	286	0.36013986	No	1	Clonal	Disease_causing	Passenger	Neutral	passenger	No	No	No	No	Not tested
CS1-C	Carcinoma	RTN4RL1	p.N63S	Private	17	1840928	T	C	Missense_Mutation	176	0.051136364	No	0.489	Subclonal	Disease_causing	Passenger	Neutral	passenger	No	No	No	No	Not tested
CS1-C	Carcinoma	RUNDC3A	p.*447Cext*88	Private	17	42395606	A	C	Nonstop_Mutation	91	0.153846154	No	1	Clonal	Disease_causing	.	.	passenger	No	No	No	No	Not tested
CS1-C	Carcinoma	RUNX1	p.T246M	Private	21	36206775	G	A	Missense_Mutation	92	0.076086957	No	0.727	Clonal	Disease_causing	Passenger	Neutral	passenger	No	Yes	Yes	Yes	Not tested
CS1-S	Sarcoma	RWDD2A	p.Q280*	Private	6	83905950	C	T	Nonsense_Mutation	325	0.4	No	1	Clonal	Disease_causing	.	.	passenger	No	No	No	No	Not tested
CS1-C	Carcinoma	RYR1	p.G2483E	Private	19	38991464	G	A	Missense_Mutation	158	0.03164557	No	0.303	Subclonal	Disease_causing	Passenger	Disease_causing	likely_pathogenic	No	No	No	No	Not tested
CS1-C	Carcinoma	RYR3	p.Q1009*	Private	15	33925307	C	T	Nonsense_Mutation	204	0.156862745	No	1	Clonal	Neutral	.	.	passenger	No	No	No	No	Not tested
CS1-C	Carcinoma	RYR3	p.Q2697R	Private	15	34040415	A	G	Missense_Mutation	123	0.040650407	No	0.389	Subclonal	Disease_causing	Passenger	Disease_causing	likely_pathogenic	No	No	No	No	Not tested
CS1-C	Carcinoma	SALL3	p.A541T	Private	18	76753612	G	A	Missense_Mutation	215	0.130232558	No	1	Clonal	Neutral	Passenger	Neutral	passenger	No	No	No	No	Not tested
CS1-C	Carcinoma	SASH3	p.R245*	Shared	X	128926744	C	T	Nonsense_Mutation	218	0.353211009	No	1	Clonal	Neutral	.	.	passenger	No	No	No	No	Not tested
CS1-S	Sarcoma	SASH3	p.R245*	Shared	X	128926744	C	T	Nonsense_Mutation	249	0.393574297	No	1	Clonal	Neutral	.	.	passenger	No	No	No	No	Not tested
CS1-S	Sarcoma	SBF1	p.R721H	Private	22	50900868	C	T	Missense_Mutation	398	0.379396985	No	1	Clonal	Neutral	Passenger	Neutral	passenger	No	No	No	No	Not tested
CS1-S	Sarcoma	SBK2	p.S129T	Private	19	56042581	A	T	Missense_Mutation	620	0.127419355	No	0.448	Subclonal	Disease_causing	Passenger	Neutral	passenger	No	No	No	No	Not tested
CS1-C	Carcinoma	SCAF4	p.R1059T	Private	21	33043980	C	G	Missense_Mutation	508	0.061023622	No	0.583	Subclonal	Disease_causing	Passenger	Neutral	passenger	No	No	No	No	Not tested
CS1-S	Sarcoma	SCAF4	p.P250Hfs*96	Private	21	33073335	TG	T	Frame_Shift_Del	351	0.356125356	No	1	Clonal	.	.	.	.	No	No	No	No	Not tested
CS1-C	Carcinoma	SCAI	p.A301T	Private	9	127781107	C	T	Missense_Mutation	181	0.077348066	No	0.74	Subclonal	Disease_causing	Passenger	Neutral	passenger	No	No	No	No	Not tested
CS1-C	Carcinoma	SCARA5	p.V479*	Private	8	27729503	AC	A	Frame_Shift_Del	194	0.134020619	No	1	Clonal	.	.	.	.	No	No	No	No	Not tested
CS1-C	Carcinoma	SCARF2	p.A489V	Private	22	20783601	G	A	Missense_Mutation	163	0.245398773	No	1	Clonal	Disease_causing	Passenger	Neutral	passenger	No	No	No	No	Not tested
CS1-C	Carcinoma	SCN4A	p.S326I	Shared	17	62045442	C	A	Missense_Mutation	393	0.409669211	No	1	Clonal	Neutral	Passenger	Disease_causing	passenger	No	No	No	No	Not tested
CS1-S	Sarcoma	SCN4A	p.S326I	Shared	17	62045442	C	A	Missense_Mutation	543	0.44014733	No	1	Clonal	Neutral	Passenger	Disease_causing	passenger	No	No	No	No	Not tested
CS1-C	Carcinoma	SCN5A	p.Y1009Tfs*136	Shared	3	38622625	AG	A	Frame_Shift_Del	234	0.055555556	No	0.531	Subclonal	.	.	.	.	No	No	No	No	Not tested
CS1-S	Sarcoma	SCN5A	p.Y1009Tfs*136	Shared	3	38622625	AG	A	Frame_Shift_Del	345	0.115942029	No	0.408	Subclonal	.	.	.	.	No	No	No	No	Not tested
CS1-S	Sarcoma	SCRIB	p.A110T	Private	8	144896016	C	T	Missense_Mutation	542	0.1900369	No	0.859	Subclonal	Disease_causing	Passenger	Neutral	passenger	No	No	No	No	Not tested
CS1-S	Sarcoma	SCYL2	p.S56Vfs*12	Private	12	100676906	C	CA	Frame_Shift_Ins	358	0.354748603	No	1	Clonal	.	.	.	.	No	No	No	No	Not tested
CS1-S	Sarcoma	SCYL3	p.A321T	Private	1	169831933	C	T	Missense_Mutation	396	0.305555556	No	1	Clonal	Neutral	Passenger	Neutral	passenger	No	No	No	No	Not tested
CS1-C	Carcinoma	SDK1	p.V1838M	Private	7	4249767	G	A	Missense_Mutation	157	0.044585987	No	0.426	Subclonal	Disease_causing	Passenger	Neutral	passenger	No	No	No	No	Not tested
CS1-S	Sarcoma	SEC14L2	p.K376del	Private	22	30818306	CAAG	C	In_Frame_Del	451	0.2172949	No	0.765	Subclonal	.	.	.	.	No	No	No	No	Not tested
CS1-S	Sarcoma	SEC16B	p.R1054H	Private	1	177899015	C	T	Missense_Mutation	292	0.109589041	No	0.495	Subclonal	Disease_causing	Passenger	Neutral	passenger	No	No	No	No	Not tested
CS1-C	Carcinoma	SEC24A	p.Q1089*	Private	5	134060767	C	T	Nonsense_Mutation	202	0.257425743	No	1	Clonal	Disease_causing	.	.	passenger	No	No	No	No	Not tested
CS1-S	Sarcoma	SEC24C	p.Q459Sfs*32	Private	10	75525559	TC	T	Frame_Shift_Del	799	0.133917397	No	0.471	Subclonal	.	.	.	.	No	No	No	No	Not tested
CS1-S	Sarcoma	SECISBP2L	p.K471Nfs*20	Private	15	49309050	AT	A	Frame_Shift_Del	350	0.237142857	No	0.834	Subclonal	.	.	.	.	No	No	No	No	Not tested
CS1-S	Sarcoma	SECISBP2L	p.C743*	Private	15	49293091	TCA	T	Nonsense_Mutation	307	0.19218241	No	0.676	Subclonal	.	.	.	.	No	No	No	No	Not tested
CS1-C	Carcinoma	SELP	p.A417V	Private	1	169578825	G	A	Missense_Mutation	536	0.156716418	No	1	Clonal	Neutral	Passenger	Neutral	passenger	No	No	No	Yes	Not tested
CS1-S	Sarcoma	SEMA4G	p.K162R	Private	10	102738101	A	G	Missense_Mutation	365	0.391780822	No	1	Clonal	Disease_causing	Passenger	Neutral	passenger	No	No	No	No	Not tested
CS1-S	Sarcoma	SEMA5B	p.L598P	Private	3	122640819	A	G	Missense_Mutation	92	0.163043478	No	0.574	Subclonal	Disease_causing	Driver	Neutral	likely_pathogenic	No	No	No	No	Not tested
CS1-S	Sarcoma	SENP1	p.G410Vfs*4	Private	12	48458895	CT	C	Frame_Shift_Del	127	0.409448819	No	1	Clonal	.	.	.	.	No	No	No	No	Not tested
CS1-S	Sarcoma	SERINC4	p.S360Pfs*142	Private	15	44088415	AG	A	Frame_Shift_Del	193	0.398963731	No	1	Clonal	.	.	.	.	No	No	No	No	Not tested
CS1-C	Carcinoma	SERPINB7	p.R315H	Private	18	61471670	G	A	Missense_Mutation	223	0.049327354	No	0.472	Subclonal	Disease_causing	Passenger	Neutral	passenger	No	No	No	No	Not tested
CS1-C	Carcinoma	SERPINC1	p.P429H	Private	1	173873136	G	T	Missense_Mutation	369	0.040650407	No	0.429	Subclonal	Neutral	Passenger	Disease_causing	passenger	No	No	No	No	Not tested
CS1-S	Sarcoma	SESN1	p.H150R	Private	6	109322588	T	C	Missense_Mutation	328	0.134146341	No	0.472	Subclonal	Disease_causing	Driver	Neutral	likely_pathogenic	No	No	No	No	Not tested
CS1-S	Sarcoma	SETD1A	p.R1132C	Private	16	30990501	C	T	Missense_Mutation	157	0.394904459	No	1	Clonal	Neutral	Passenger	Disease_causing	passenger	No	No	No	No	Not tested
CS1-C	Carcinoma	SETD1B	p.H8Tfs*27	Private	12	122242657	AC	A	Frame_Shift_Del	121	0.140495868	No	1	Clonal	.	.	.	.	No	No	No	No	Not tested
CS1-C	Carcinoma	SETD1B	p.R1022P	Private	12	122255363	G	C	Missense_Mutation	216	0.064814815	No	0.62	Subclonal	Disease_causing	Passenger	Neutral	passenger	No	No	No	No	Not tested
CS1-C	Carcinoma	SETDB2	p.R232Q	Private	13	50050965	G	A	Missense_Mutation	220	0.036363636	No	0.348	Subclonal	Disease_causing	Passenger	Disease_causing	likely_pathogenic	No	No	No	No	Not tested
CS1-C	Carcinoma	SETDB2	p.S402N	Private	13	50056885	G	A	Missense_Mutation	89	0.168539326	No	1	Clonal	Neutral	Passenger	Disease_causing	passenger	No	No	No	No	Not tested
CS1-C	Carcinoma	SFSWAP	p.A932T	Private	12	132281826	G	A	Missense_Mutation	197	0.050761421	No	0.485	Subclonal	Neutral	Passenger	Neutral	passenger	No	No	No	No	Not tested
CS1-C	Carcinoma	SGK223	p.V131I	Private	8	8235528	C	T	Missense_Mutation	306	0.068627451	No	0.656	Subclonal	Neutral	Passenger	Neutral	passenger	No	No	No	No	Not tested
CS1-C	Carcinoma	SGMS1	p.W182L	Private	10	52103330	C	A	Missense_Mutation	269	0.078066914	No	0.746	Subclonal	Disease_causing	Passenger	Neutral	passenger	No	No	No	No	Not tested
CS1-C	Carcinoma	SGSM2	p.Y155H	Private	17	2266136	T	C	Missense_Mutation	217	0.059907834	No	0.573	Subclonal	Disease_causing	Passenger	Neutral	passenger	No	No	No	No	Not tested
CS1-S	Sarcoma	SGSM3	p.X618_splice	Private	22	40804796	A	G	Splice_Site	376	0.446808511	No	1	Clonal	Disease_causing	.	.	passenger	No	No	No	No	Not tested
CS1-C	Carcinoma	SH2B1	p.E299G	Private	16	28878311	A	G	Missense_Mutation	216	0.041666667	No	0.398	Subclonal	Disease_causing	Passenger	Neutral	passenger	No	No	No	No	Not tested
CS1-C	Carcinoma	SH2B3	p.P4S	Private	12	111855959	C	T	Missense_Mutation	299	0.214046823	No	1	Clonal	Disease_causing	Passenger	Neutral	passenger	No	Yes	No	No	Not tested
CS1-C	Carcinoma	SH2D3A	p.V425A	Private	19	6754173	A	G	Missense_Mutation	296	0.030405405	No	0.291	Subclonal	Neutral	Passenger	Neutral	passenger	No	No	No	No	Not tested
CS1-S	Sarcoma	SH2D3C	p.P115Qfs*9	Private	9	130536439	TG	T	Frame_Shift_Del	343	0.151603499	No	0.533	Subclonal	.	.	.	.	No	No	No	No	Not tested
CS1-S	Sarcoma	SH2D5	p.R241H	Private	1	21050653	C	T	Missense_Mutation	268	0.089552239	No	0.315	Subclonal	Disease_causing	Passenger	.	passenger	No	No	No	No	Not tested
CS1-C	Carcinoma	SH3TC2	p.A1240T	Private	5	148384423	C	T	Missense_Mutation	235	0.259574468	No	1	Clonal	Disease_causing	Passenger	Neutral	passenger	No	No	No	No	Not tested
CS1-S	Sarcoma	SH3TC2	p.M454V	Private	5	148407935	T	C	Missense_Mutation	497	0.390342052	No	1	Clonal	Neutral	Passenger	Neutral	passenger	No	No	No	No	Not tested
CS1-C	Carcinoma	SHANK2	p.P890Lfs*56	Private	11	70333731	AG	A	Frame_Shift_Del	196	0.178571429	No	1	Clonal	.	.	.	.	No	No	No	No	Not tested
CS1-C	Carcinoma	SHANK3	p.R1590C	Private	22	51169264	C	T	Missense_Mutation	58	0.206896552	No	1	Clonal	Disease_causing	Passenger	Neutral	passenger	No	No	No	No	Not tested
CS1-S	Sarcoma	SHD	p.R35H	Private	19	4280164	G	A	Missense_Mutation	489	0.141104294	No	0.497	Subclonal	Disease_causing	Passenger	Neutral	passenger	No	No	No	No	Not tested
CS1-C	Carcinoma	SHISA6	p.P430L	Private	17	11461254	C	T	Missense_Mutation	454	0.055066079	No	0.526	Subclonal	Disease_causing	Passenger	.	passenger	No	No	No	No	Not tested
CS1-C	Carcinoma	SHROOM3	p.L1865I	Private	4	77692022	C	A	Missense_Mutation	409	0.034229829	No	0.327	Subclonal	Disease_causing	Passenger	Neutral	passenger	No	No	No	No	Not tested
CS1-S	Sarcoma	SIGLEC1	p.R1336H	Private	20	3673191	C	T	Missense_Mutation	120	0.075	No	0.264	Subclonal	Neutral	Passenger	Neutral	passenger	No	No	No	No	Not tested
CS1-S	Sarcoma	SIN3B	p.R919W	Private	19	16986932	C	T	Missense_Mutation	251	0.386454183	No	1	Clonal	Disease_causing	Passenger	Neutral	passenger	No	No	No	No	Not tested
CS1-C	Carcinoma	SIPA1L1	p.R173Q	Private	14	72055107	G	A	Missense_Mutation	386	0.183937824	No	1	Clonal	Disease_causing	Driver	Neutral	likely_pathogenic	No	No	No	No	Not tested
CS1-S	Sarcoma	SIRT5	p.R44*	Private	6	13588577	C	T	Nonsense_Mutation	133	0.07518797	No	0.265	Subclonal	Disease_causing	.	.	passenger	No	No	No	No	Not tested
CS1-C	Carcinoma	SIX5	p.A625Pfs*31	Private	19	46269106	CG	C	Frame_Shift_Del	102	0.156862745	No	1	Clonal	.	.	.	.	No	No	No	No	Not tested
CS1-S	Sarcoma	SKI	p.A534V	Private	1	2235858	C	T	Missense_Mutation	302	0.145695364	No	0.513	Subclonal	Neutral	Passenger	Disease_causing	passenger	No	No	No	No	Not tested
CS1-S	Sarcoma	SKIDA1	p.R325M	Private	10	21805778	C	A	Missense_Mutation	91	0.076923077	No	0.271	Subclonal	Neutral	Passenger	.	passenger	No	No	No	No	Not tested
CS1-C	Carcinoma	SLC12A1	p.F966I	Private	15	48583997	T	A	Missense_Mutation	294	0.030612245	No	0.293	Subclonal	Disease_causing	Passenger	Disease_causing	likely_pathogenic	No	No	No	No	Not tested
CS1-C	Carcinoma	SLC12A7	p.W501*	Private	5	1078074	C	T	Nonsense_Mutation	187	0.229946524	No	1	Clonal	Neutral	.	.	passenger	No	No	No	No	Not tested
CS1-S	Sarcoma	SLC13A1	p.T271A	Private	7	122787214	T	C	Missense_Mutation	117	0.076923077	No	0.271	Subclonal	Disease_causing	Passenger	Neutral	passenger	No	No	No	No	Not tested
CS1-C	Carcinoma	SLC13A4	p.I514V	Private	7	135370335	T	C	Missense_Mutation	198	0.262626263	No	1	Clonal	Disease_causing	Passenger	Neutral	passenger	No	No	No	No	Not tested
CS1-C	Carcinoma	SLC16A2	p.A432P	Private	X	73749171	G	C	Missense_Mutation	178	0.056179775	No	0.537	Subclonal	Disease_causing	Passenger	Neutral	passenger	No	No	No	No	Not tested
CS1-S	Sarcoma	SLC17A7	p.R552Gfs*30	Private	19	49933805	TG	T	Frame_Shift_Del	115	0.165217391	No	0.581	Subclonal	.	.	.	.	No	No	No	No	Not tested
CS1-S	Sarcoma	SLC18A2	p.F238Sfs*48	Private	10	119014791	GC	G	Frame_Shift_Del	237	0.240506329	No	0.846	Subclonal	.	.	.	.	No	No	No	No	Not tested
CS1-S	Sarcoma	SLC18A3	p.R500C	Private	10	50820284	C	T	Missense_Mutation	404	0.45049505	No	1	Clonal	Disease_causing	Passenger	Neutral	passenger	No	No	No	No	Not tested
CS1-S	Sarcoma	SLC20A1	p.R332Afs*14	Private	2	113416606	CAG	C	Frame_Shift_Del	487	0.184804928	No	0.65	Subclonal	.	.	.	.	No	No	No	No	Not tested
CS1-S	Sarcoma	SLC20A2	p.R381C	Private	8	42294889	G	A	Missense_Mutation	620	0.093548387	No	0.423	Subclonal	Disease_causing	Passenger	Disease_causing	likely_pathogenic	No	No	No	No	Not tested
CS1-C	Carcinoma	SLC22A17	p.P462L	Private	14	23816089	G	A	Missense_Mutation	116	0.094827586	No	0.907	Clonal	Disease_causing	Passenger	Neutral	passenger	No	No	No	No	Not tested
CS1-C	Carcinoma	SLC25A12	p.R353*	Private	2	172669963	G	A	Nonsense_Mutation	84	0.083333333	No	0.797	Clonal	Neutral	.	.	passenger	No	No	No	No	Not tested

CS1-C	Carcinoma	SLC25A22	p.M107I	Private	11	792961	C	T	Missense_Mutation	[illegible]	[illegible]	No	0.806	Clonal	Disease_causing	Passenger	Neutral	passenger	No	No	No	No	Not tested
CS1-C	Carcinoma	SLC25A37	p.V268M	Shared	8	23429153	G	A	Missense_Mutation	[illegible]	[illegible]	No	1	Clonal	Neutral	Passenger	Neutral	passenger	No	No	No	No	Not tested
CS1-S	Sarcoma	SLC25A37	p.V268M	Shared	8	23429153	G	A	Missense_Mutation	933	0.279742765	No	1	Clonal	Neutral	Passenger	Neutral	passenger	No	No	No	No	Not tested
CS1-C	Carcinoma	SLC2A13	p.A584V	Private	12	40154024	G	A	Missense_Mutation	215	0.065116279	No	0.623	Subclonal	Neutral	Passenger	Neutral	passenger	No	No	No	No	Not tested
CS1-S	Sarcoma	SLC36A4	p.Y326N	Private	11	92895933	A	T	Missense_Mutation	174	0.120689655	No	0.425	Subclonal	Disease_causing	Passenger	Neutral	passenger	No	No	No	No	Not tested
CS1-S	Sarcoma	SLC39A3	p.A62T	Private	19	2737072	C	T	Missense_Mutation	438	0.139269406	No	0.49	Subclonal	Disease_causing	Passenger	Neutral	passenger	No	No	No	No	Not tested
CS1-C	Carcinoma	SLC43A3	p.L446P	Private	11	57176682	A	G	Missense_Mutation	222	0.031531532	No	0.301	Subclonal	Disease_causing	Passenger	Disease_causing	likely_pathogenic	No	No	No	No	Not tested
CS1-C	Carcinoma	SLC44A1	p.I459V	Private	9	108127885	A	G	Missense_Mutation	194	0.139175258	No	1	Clonal	Disease_causing	Passenger	Neutral	passenger	No	No	No	No	Not tested
CS1-C	Carcinoma	SLC45A3	p.S451N	Private	1	205628672	C	T	Missense_Mutation	480	0.170833333	No	1	Clonal	Neutral	Passenger	Neutral	passenger	No	Yes	No	No	Not tested
CS1-C	Carcinoma	SLC45A4	p.R703W	Private	8	142222337	G	A	Missense_Mutation	153	0.052287582	No	0.5	Subclonal	Neutral	Passenger	Neutral	passenger	No	No	No	No	Not tested
CS1-C	Carcinoma	SLC47A1	p.A465Pfs*11	Private	17	19474867	GA	G	Frame_Shift_Del	141	0.212765957	No	1	Clonal	.	.	.	.	No	No	No	No	Not tested
CS1-S	Sarcoma	SLC4A3	p.A1234P	Private	2	220505682	G	C	Missense_Mutation	328	0.067073171	No	0.236	Subclonal	Disease_causing	Passenger	Neutral	passenger	No	No	No	No	Not tested
CS1-C	Carcinoma	SLC4A4	p.N616D	Private	4	72338630	A	G	Missense_Mutation	265	0.037735849	No	0.361	Subclonal	Neutral	Passenger	Neutral	passenger	No	No	No	Yes	Not tested
CS1-C	Carcinoma	SLC6A19	p.L540Cfs*4	Private	5	1221340	GC	G	Frame_Shift_Del	246	0.134146341	No	1	Clonal	.	.	.	.	No	No	No	No	Not tested
CS1-C	Carcinoma	SLC6A3	p.C319*	Private	5	1416287	G	T	Nonsense_Mutation	103	0.233009709	No	1	Clonal	Neutral	.	.	passenger	No	No	No	No	Not tested
CS1-C	Carcinoma	SLC6A8	p.G284V	Private	X	152958569	G	T	Missense_Mutation	60	0.1	No	0.956	Clonal	Disease_causing	Driver	Disease_causing	likely_pathogenic	No	No	No	No	Not tested
CS1-S	Sarcoma	SLC9A3R2	p.L112P	Private	16	2079704	T	C	Missense_Mutation	165	0.109090909	No	0.384	Subclonal	Disease_causing	Passenger	Neutral	passenger	No	No	No	No	Not tested
CS1-C	Carcinoma	SLC9C1	p.V209M	Private	3	111988913	C	T	Missense_Mutation	311	0.24437299	No	1	Clonal	Neutral	Passenger	Neutral	passenger	No	No	No	No	Not tested
CS1-C	Carcinoma	SLFN13	p.T728I	Private	17	33768125	G	A	Missense_Mutation	407	0.221130221	No	1	Clonal	Neutral	Passenger	Disease_causing	passenger	No	No	No	No	Not tested
CS1-S	Sarcoma	SLIT2	p.C510Vfs*4	Private	4	20530631	G	GA	Frame_Shift_Ins	251	0.25498008	No	0.897	Subclonal	.	.	.	.	No	No	No	No	Not tested
CS1-S	Sarcoma	SLPI	p.A19V	Private	20	43883129	G	A	Missense_Mutation	86	0.476744186	No	1	Clonal	Neutral	Passenger	Neutral	passenger	No	No	No	No	Not tested
CS1-C	Carcinoma	SMARCA4	p.V1016M	Private	19	11135079	G	A	Missense_Mutation	228	0.105263158	No	1	Clonal	Disease_causing	Passenger	Disease_causing	likely_pathogenic	No	Yes	No	Yes	Not tested
CS1-C	Carcinoma	SMARCAL1	p.G857R	Private	2	217342966	G	A	Missense_Mutation	218	0.224770642	No	1	Clonal	Disease_causing	Passenger	Disease_causing	likely_pathogenic	No	No	No	No	Not tested
CS1-C	Carcinoma	SMC1A	p.A301T	Private	X	53439157	C	T	Missense_Mutation	236	0.038135593	No	0.365	Subclonal	Disease_causing	Passenger	Neutral	passenger	No	No	Yes	Yes	Not tested
CS1-C	Carcinoma	SMG5	p.S182F	Private	1	156243241	G	A	Missense_Mutation	287	0.174216028	No	1	Clonal	Disease_causing	Passenger	Neutral	passenger	No	No	No	No	Not tested
CS1-C	Carcinoma	SMG6	p.R1095W	Shared	17	2076026	G	A	Missense_Mutation	137	0.335766423	No	1	Clonal	Disease_causing	Passenger	Neutral	passenger	No	No	No	No	Not tested
CS1-S	Sarcoma	SMG6	p.R1095W	Shared	17	2076026	G	A	Missense_Mutation	192	0.463541667	No	1	Clonal	Disease_causing	Passenger	Neutral	passenger	No	No	No	No	Not tested
CS1-C	Carcinoma	SMTN	p.T481I	Private	22	31487451	C	T	Missense_Mutation	172	0.058139535	No	0.556	Subclonal	Neutral	Passenger	Neutral	passenger	No	No	No	No	Not tested
CS1-C	Carcinoma	SNAP29	p.R256Q	Private	22	21242114	G	A	Missense_Mutation	165	0.121212121	No	1	Clonal	Disease_causing	Passenger	.	passenger	No	No	No	No	Not tested
CS1-C	Carcinoma	SNRNP200	p.R1152C	Private	2	96953207	G	A	Missense_Mutation	237	0.088607595	No	0.847	Clonal	Disease_causing	Passenger	Neutral	passenger	No	No	No	No	Not tested
CS1-S	Sarcoma	SNRNP35	p.G151E	Private	12	123950524	G	A	Missense_Mutation	412	0.25	No	0.88	Subclonal	Disease_causing	Passenger	Neutral	passenger	No	No	No	No	Not tested
CS1-S	Sarcoma	SNX25	p.N638Ifs*51	Private	4	186272694	GA	G	Frame_Shift_Del	321	0.115264798	No	0.406	Subclonal	.	.	.	.	No	No	No	Yes	Not tested
CS1-C	Carcinoma	SNX31	p.I150Lfs*27	Private	8	101625297	AT	A	Frame_Shift_Del	343	0.160349854	No	1	Clonal	.	.	.	.	No	No	No	No	Not tested
CS1-C	Carcinoma	SNX4	p.R105W	Private	3	125216989	G	A	Missense_Mutation	133	0.112781955	No	1	Clonal	Disease_causing	Passenger	Neutral	passenger	No	No	No	No	Not tested
CS1-S	Sarcoma	SOX1	p.Q255*	Private	13	112722735	C	T	Nonsense_Mutation	194	0.226804124	No	0.798	Subclonal	Disease_causing	.	.	passenger	No	No	No	No	Not tested
CS1-S	Sarcoma	SP5	p.K294del	Private	2	171573589	GGAA	G	In_Frame_Del	293	0.126279863	No	0.444	Subclonal	.	.	.	.	No	No	No	No	Not tested
CS1-C	Carcinoma	SP7	p.P292S	Private	12	53722352	G	A	Missense_Mutation	168	0.172619048	No	1	Clonal	Disease_causing	Passenger	Neutral	passenger	No	No	No	No	Not tested
CS1-S	Sarcoma	SPATA20	p.S72Y	Private	17	48625733	C	A	Missense_Mutation	508	0.267716535	No	0.942	Clonal	Neutral	Passenger	Neutral	passenger	No	No	No	No	Not tested
CS1-C	Carcinoma	SPG11	p.G1539Efs*6	Private	15	44887476	CCT	C	Frame_Shift_Del	221	0.199095023	No	1	Clonal	.	.	.	.	No	No	No	No	Not tested
CS1-S	Sarcoma	SPINT2	p.R48W	Private	19	38774302	C	T	Missense_Mutation	373	0.136729223	No	0.481	Subclonal	Disease_causing	Passenger	Neutral	passenger	No	No	No	No	Not tested
CS1-S	Sarcoma	SPIRE2	p.V531Wfs*6	Private	16	89929894	AC	A	Frame_Shift_Del	109	0.293577982	No	1	Clonal	.	.	.	.	No	No	No	No	Not tested
CS1-C	Carcinoma	SPOPL	p.A321V	Private	2	139322402	C	T	Missense_Mutation	218	0.169724771	No	1	Clonal	Disease_causing	Passenger	Neutral	passenger	No	No	No	No	Not tested
CS1-C	Carcinoma	SPRED3	p.A302T	Private	19	38886456	G	A	Missense_Mutation	57	0.263157895	No	1	Clonal	Neutral	Passenger	Neutral	passenger	No	No	No	No	Not tested
CS1-C	Carcinoma	SPTLC2	p.G497E	Private	14	77984460	C	T	Missense_Mutation	185	0.048648649	No	0.465	Subclonal	Disease_causing	Passenger	Disease_causing	likely_pathogenic	No	No	No	No	Not tested
CS1-C	Carcinoma	SPTY2D1	p.D33Tfs*23	Private	11	18638500	CT	C	Frame_Shift_Del	138	0.152173913	No	1	Clonal	.	.	.	.	No	No	No	No	Not tested
CS1-C	Carcinoma	SRCIN1	p.R996H	Shared	17	36705422	C	T	Missense_Mutation	50	0.38	No	1	Clonal	Disease_causing	Passenger	Neutral	passenger	No	No	No	No	Not tested
CS1-S	Sarcoma	SRCIN1	p.V361I	Private	17	36718318	C	T	Missense_Mutation	215	0.106976744	No	0.376	Subclonal	Neutral	Passenger	Neutral	passenger	No	No	No	No	Not tested
CS1-S	Sarcoma	SRCIN1	p.R996H	Shared	17	36705422	C	T	Missense_Mutation	59	0.457627119	No	1	Clonal	Disease_causing	Passenger	Neutral	passenger	No	No	No	No	Not tested
CS1-C	Carcinoma	SRP72	p.V9Gfs*9	Private	4	57333819	C	CG	Frame_Shift_Ins	323	0.179566563	No	1	Clonal	.	.	.	.	No	No	No	No	Not tested
CS1-C	Carcinoma	SRPR	p.K170Rfs*33	Private	11	126137086	CT	C	Frame_Shift_Del	134	0.223880597	No	1	Clonal	.	.	.	.	No	No	No	No	Not tested
CS1-C	Carcinoma	SRSF2	p.R154L	Private	17	74732448	C	A	Missense_Mutation	488	0.034836066	No	0.333	Subclonal	Disease_causing	Passenger	Neutral	passenger	No	Yes	No	Yes	Not tested
CS1-C	Carcinoma	STAB1	p.R1122Pfs*37	Private	3	52547907	G	GC	Frame_Shift_Ins	329	0.197568389	No	1	Clonal	.	.	.	.	No	No	No	No	Not tested
CS1-C	Carcinoma	STARD3NL	p.X185_splice	Private	7	38259165	G	A	Splice_Site	138	0.057971014	No	0.554	Subclonal	Disease_causing	.	.	passenger	No	No	No	No	Not tested
CS1-C	Carcinoma	STARD9	p.E4068G	Private	15	42985979	A	G	Missense_Mutation	295	0.033898305	No	0.324	Subclonal	Neutral	Passenger	Neutral	passenger	No	No	No	No	Not tested
CS1-S	Sarcoma	STIM2	p.S457A	Private	4	27010480	T	G	Missense_Mutation	399	0.426065163	No	1	Clonal	Disease_causing	Passenger	Neutral	passenger	No	No	No	No	Not tested
CS1-S	Sarcoma	STK10	p.T526Nfs*27	Private	5	171517344	G	GT	Frame_Shift_Ins	379	0.168865435	No	0.594	Subclonal	.	.	.	.	No	No	No	No	Not tested
CS1-C	Carcinoma	STK3	p.E53del	Private	8	99786997	ACTT	A	In_Frame_Del	199	0.105527638	No	1	Clonal	.	.	.	.	No	No	No	No	Not tested
CS1-S	Sarcoma	STK4	p.N371D	Private	20	43629958	A	G	Missense_Mutation	260	0.130769231	No	0.46	Subclonal	Disease_causing	Passenger	Neutral	passenger	No	No	No	No	Not tested
CS1-S	Sarcoma	STRIP2	p.P514L	Private	7	129102871	C	T	Missense_Mutation	220	0.281818182	No	0.992	Clonal	Disease_causing	Passenger	Neutral	passenger	No	No	No	No	Not tested
CS1-S	Sarcoma	STRN4	p.R495W	Private	19	47228599	G	A	Missense_Mutation	444	0.146396396	No	0.515	Subclonal	Disease_causing	Passenger	Neutral	passenger	No	No	No	No	Not tested
CS1-S	Sarcoma	STT3A	p.L18*	Private	11	125465859	CT	C	Frame_Shift_Del	257	0.256809339	No	0.904	Clonal	.	.	.	.	No	No	No	No	Not tested
CS1-C	Carcinoma	STT3B	p.F353Sfs*7	Private	3	31658498	CT	C	Frame_Shift_Del	270	0.225925926	No	1	Clonal	.	.	.	.	No	No	No	No	Not tested
CS1-C	Carcinoma	SUPT5H	p.R711C	Private	19	39963545	C	T	Missense_Mutation	148	0.054054054	No	0.517	Subclonal	Disease_causing	Passenger	.	passenger	No	No	No	No	Not tested
CS1-C	Carcinoma	SV2A	p.T500M	Private	1	149879639	G	A	Missense_Mutation	414	0.171497585	No	1	Clonal	Disease_causing	Passenger	Neutral	passenger	No	No	No	No	Not tested
CS1-S	Sarcoma	SVEP1	p.C305Y	Private	9	113308445	C	T	Missense_Mutation	470	0.108510638	No	0.382	Subclonal	Disease_causing	Passenger	Disease_causing	likely_pathogenic	No	No	No	No	Not tested
CS1-S	Sarcoma	SVIL	p.M1863Wfs*44	Private	10	29760115	TC	T	Frame_Shift_Del	366	0.363387978	No	1	Clonal	.	.	.	.	No	No	No	No	Not tested
CS1-S	Sarcoma	SYBU	p.Q37Tfs*10	Private	8	110655077	G	GT	Frame_Shift_Ins	601	0.18469218	No	0.835	Subclonal	.	.	.	.	No	No	No	No	Not tested
CS1-S	Sarcoma	SYCE1	p.K69Efs*4	Private	10	135372448	C	CT	Frame_Shift_Ins	403	0.094292804	No	0.332	Subclonal	.	.	.	.	No	No	No	No	Not tested
CS1-S	Sarcoma	SYMPK	p.R205Dfs*49	Private	19	46351072	CG	C	Frame_Shift_Del	118	0.415254237	No	1	Clonal	.	.	.	.	No	No	No	No	Not tested
CS1-C	Carcinoma	SYNDIG1	p.R52W	Shared	20	24523887	C	T	Missense_Mutation	250	0.4	No	1	Clonal	Neutral	Passenger	Disease_causing	passenger	No	No	No	No	Not tested
CS1-S	Sarcoma	SYNDIG1	p.R52W	Shared	20	24523887	C	T	Missense_Mutation	332	0.406626506	No	1	Clonal	Neutral	Passenger	Disease_causing	passenger	No	No	No	No	Not tested
CS1-C	Carcinoma	SYNE1	p.V8387M	Shared	6	152462425	C	T	Missense_Mutation	178	0.359550562	No	1	Clonal	Disease_causing	Passenger	Neutral	passenger	No	No	No	No	Not tested
CS1-C	Carcinoma	SYNE1	p.A5498V	Private	6	152639295	G	A	Missense_Mutation	290	0.034482759	No	0.33	Subclonal	Disease_causing	Passenger	Neutral	passenger	No	No	No	No	Not tested
CS1-S	Sarcoma	SYNE1	p.V8387M	Shared	6	152462425	C	T	Missense_Mutation	219	0.401826484	No	1	Clonal	Disease_causing	Passenger	Neutral	passenger	No	No	No	No	Not tested
CS1-S	Sarcoma	SYNE1	p.D4244G	Private	6	152655206	T	C	Missense_Mutation	408	0.154411765	No	0.543	Subclonal	Disease_causing	Passenger	Neutral	passenger	No	No	No	No	Not tested
CS1-S	Sarcoma	SYNE2	p.Q6862L	Private	14	64692105	A	T	Missense_Mutation	195	0.266666667	No	0.938	Clonal	Disease_causing	Passenger	Neutral	passenger	No	No	No	No	Not tested
CS1-C	Carcinoma	SYNGAP1	p.P939S	Private	6	33411144	C	T	Missense_Mutation	335	0.03880597	No	0.371	Subclonal	Disease_causing	Passenger	Neutral	passenger	No	No	No	No	Not tested
CS1-S	Sarcoma	SYNJ1	p.F949Lfs*5	Private	21	34029061	CA	C	Frame_Shift_Del	409	0.37408313	No	1	Clonal	.	.	.	.	No	No	No	No	Not tested
CS1-C	Carcinoma	SYNM	p.R843I	Private	15	99671096	G	T	Missense_Mutation	351	0.071225071	No	0.681	Subclonal	Neutral	Passenger	Disease_causing	passenger	No	No	No	No	Not tested
CS1-C	Carcinoma	SYT14	p.E406G	Private	1	210273724	A	G	Missense_Mutation	458	0.074235808	No	0.784	Clonal	Disease_causing	Passenger	Neutral	passenger	No	No	No	No	Not tested
CS1-S	Sarcoma	SYT17	p.T277I	Private	16	19195348	C	T	Missense_Mutation	353	0.385269122	No	1	Clonal	Disease_causing	Passenger	Neutral	passenger	No	No	No	No	Not tested
CS1-S	Sarcoma	SZT2	p.R1994C	Private	1	43902958	C	T	Missense_Mutation	628	0.404458599	No	1	Clonal	Disease_causing	Passenger	.	passenger	No	No	No	No	Not tested
CS1-C	Carcinoma	TAB3	p.A567V	Private	X	30870905	G	A	Missense_Mutation	290	0.034482759	No	0.33	Subclonal	Disease_causing	Passenger	Neutral	passenger	No	No	No	No	Not tested
CS1-C	Carcinoma	TACC1	p.P35S	Private	8	38645203	C	T	Missense_Mutation	189	0.17989418	No	1	Clonal	Neutral	Passenger	Neutral	passenger	No	No	No	No	Not tested
CS1-C	Carcinoma	TACC3	p.R801K	Private	4	1746510	G	A	Missense_Mutation	177	0.192090395	No	1	Clonal	Disease_causing	Passenger	Neutral	passenger	No	No	No	No	Not tested
CS1-S	Sarcoma	TAF1	p.D1748Y	Private	X	70679519	G	T	Missense_Mutation	490	0.13877551	No	0.488	Subclonal	Disease_causing	Passenger	Neutral	passenger	No	No	Yes	No	Not tested
CS1-C	Carcinoma	TARS	p.S314P	Private	5	33457365	T	C	Missense_Mutation	196	0.117346939	No	1	Clonal	Disease_causing	Driver	Neutral	likely_pathogenic	No	No	No	No	Not tested
CS1-S	Sarcoma	TARS	p.V405A	Private	5	33459831	T	C	Missense_Mutation	345	0.107246377	No	0.377	Subclonal	Disease_causing	Driver	Neutral	likely_pathogenic	No	No	No	No	Not tested
CS1-C	Carcinoma	TAS1R2	p.D519N	Private	1	19168259	C	T	Missense_Mutation	236	0.275423729	No	1	Clonal	Neutral	Passenger	Disease_causing	passenger	No	No	No	No	Not tested
CS1-S	Sarcoma	TAS2R39	p.M56V	Private	7	142880677	A	G	Missense_Mutation	340	0.117647059	No	0.414	Subclonal	Neutral	Passenger	Neutral	passenger	No	No	No	No	Not tested
CS1-S	Sarcoma	TAS2R50	p.I199F	Private	12	11138865	T	A	Missense_Mutation	288	0.256944444	No	0.904	Clonal	Neutral	Passenger	Neutral	passenger	No	No	No	No	Not tested
CS1-S	Sarcoma	TBC1D10B	p.S321Lfs*20	Private	16	30376909	GCT	G	Frame_Shift_Del	427	0.227166276	No	0.799	Subclonal	.	.	.	.	No	No	No	No	Not tested
CS1-C	Carcinoma	TBC1D30	p.A42V	Private	12	65218784	C	T	Missense_Mutation	99	0.050505051	No	0.483	Subclonal	Disease_causing	Passenger	Neutral	passenger	No	No	No	No	Not tested
CS1-C	Carcinoma	TBC1D4	p.R244H	Private	13	75936511	C	T	Missense_Mutation	280	0.235714286	No	1	Clonal	Disease_causing	Passenger	Neutral	passenger	No	No	No	No	Not tested
CS1-C	Carcinoma	TBC1D8	p.P529T	Private	2	101650194	G	T	Missense_Mutation	172	0.255813953	No	1	Clonal	Disease_causing	Passenger	Neutral	passenger	No	No	No	No	Not tested
CS1-C	Carcinoma	TBC1D8B	p.F650S	Private	X	106093366	T	C	Missense_Mutation	295	0.030508475	No	0.292	Subclonal	Disease_causing	Passenger	Neutral	passenger	No	No	No	No	Not tested
CS1-S	Sarcoma	TBR1	p.A489V	Private	2	162280155	C	T	Missense_Mutation	139	0.410071942	No	1	Clonal	Disease_causing	Passenger	Disease_causing	likely_pathogenic	No	No	No	No	Not tested
CS1-C	Carcinoma	TCEANC	p.E179D	Private	X	13681074	G	T	Missense_Mutation	308	0.194805195	No	1	Clonal	Neutral	Passenger	Neutral	passenger	No	No	No	No	Not tested
CS1-C	Carcinoma	TCERG1	p.K957Rfs*17	Private	5	145886722	CA	C	Frame_Shift_Del	100	0.17	No	1	Clonal	.	.	.	.	No	No	No	No	Not tested
CS1-S	Sarcoma	TCHH	p.Q907E	Private	1	152082974	G	C	Missense_Mutation	560	0.119642857	No	0.541	Subclonal	Neutral	Passenger	Neutral	passenger	No	No	No	No	Not tested
CS1-C	Carcinoma	TCHP	p.R405K	Private	12	110352326	G	A	Missense_Mutation	188	0.031914894	No	0.305	Subclonal	Disease_causing	Passenger	Neutral	passenger	No	No	No	No	Not tested
CS1-S	Sarcoma	TDGF1	p.F94L	Private	3	46621287	T	G	Missense_Mutation	228	0.421052632	No	1	Clonal	Disease_causing	Passenger	Neutral	passenger	No	No	No	No	Not tested
CS1-C	Carcinoma	TDRD15	p.L1609Ffs*8	Private	2	21365158	G	GT	Frame_Shift_Ins	243	0.267489712	No	1	Clonal	.	.	.	.	No	No	No	No	Not tested
CS1-C	Carcinoma	TDRD6	p.Q753*	Private	6	46658122	C	T	Nonsense_Mutation	227	0.070484581	No	0.674	Subclonal	Neutral	.	.	passenger	No	No	No	No	Not tested
CS1-S	Sarcoma	TDRD7	p.P1016S	Private	9	100249584	C	T	Missense_Mutation	140	0.15	No	0.528	Subclonal	Disease_causing	Passenger	Neutral	passenger	No	No	No	No	Not tested
CS1-C	Carcinoma	TEAD2	p.H299Mfs*12	Shared	19	49850472	TG	T	Frame_Shift_Del	390	0.235897436	No	1	Clonal	.	.	.	.	No	No	No	No	Not tested
CS1-S	Sarcoma	TEAD2	p.H299Mfs*12	Shared	19	49850472	TG	T	Frame_Shift_Del	483	0.293995859	No	1	Clonal	.	.	.	.	No	No	No	No	Not tested
CS1-S	Sarcoma	TECR	p.K16del	Private	19	14673364	GAGA	G	In_Frame_Del	336	0.383928571	No	1	Clonal	.	.	.	.	No	No	No	No	Not tested
CS1-C	Carcinoma	TENM4	p.Q2047*	Private	11	78381251	G	A	Nonsense_Mutation	382	0.07591623	No	0.726	Subclonal	Neutral	.	.	passenger	No	No	No	No	Not tested
CS1-C	Carcinoma	TEP1	p.R2242C	Private	14	20841519	G	A	Missense_Mutation	291	0.10652921	No	1	Clonal	Neutral	Passenger	Neutral	passenger	No	No	No	No	Not tested
CS1-S	Sarcoma	TERT	p.A817T	Private	5	1271253	C	T	Missense_Mutation	292	0.29109589	No	1	Clonal	Neutral	Passenger	Disease_causing	passenger	No	Yes	No	No	Not tested
CS1-C	Carcinoma	TET3	p.R721W	Private	2	74300747	C	T	Missense_Mutation	267	0.217228464	No	1	Clonal	Disease_causing	Passenger	Neutral	passenger	No	No	No	No	Not tested
CS1-S	Sarcoma	TEX264	p.D214A	Private	3	51733582	A	C	Missense_Mutation	162	0.135802469	No	0.478	Subclonal	Disease_causing	Passenger	Neutral	passenger	No	No	No	No	Not tested
CS1-S	Sarcoma	TFB2M	p.N139Ifs*8	Private	1	246720822	AT	A	Frame_Shift_Del	336	0.1875	No	0.847	Subclonal	.	.	.	.	No	No	No	No	Not tested
CS1-S	Sarcoma	TGDS	p.Y302C	Private	13	95228645	T	C	Missense_Mutation	205	0.165853659	No	0.584	Subclonal	Disease_causing	Passenger	Disease_causing	likely_pathogenic	No	No	No	No	Not tested
CS1-S	Sarcoma	THAP4	p.G111Vfs*2	Private	2	242573239	AC	A	Frame_Shift_Del	469	0.373134328	No	1	Clonal	.	.	.	.	No	No	No	No	Not tested
CS1-S	Sarcoma	THG1L	p.H10R	Private	5	157158477	A	G	Missense_Mutation	369	0.273712737	No	0.963	Clonal	Neutral	Passenger	Neutral	passenger	No	No	No	No	Not tested
CS1-S	Sarcoma	THYN1	p.N194Ifs*15	Private	11	134118752	AT	A	Frame_Shift_Del	103	0.174757282	No	0.615	Subclonal	.	.	.	.	No	No	No	No	Not tested
CS1-S	Sarcoma	TIAM2	p.G115D	Private	6	155450701	G	A	Missense_Mutation	425	0.428235294	No	1	Clonal	Disease_causing	Passenger	Neutral	passenger	No	No	No	No	Not tested
CS1-C	Carcinoma	TLR7	p.S490R	Private	X	12905095	A	C	Missense_Mutation	223	0.058295964	No	0.557	Subclonal	Neutral	Passenger	Neutral	passenger	No	No	No	No	Not tested
CS1-S	Sarcoma	TM7SF2	p.A125T	Private	11	64880760	G	A	Missense_Mutation	163	0.134969325	No	0.475	Subclonal	Neutral	Passenger	Disease_causing	passenger	No	No	No	No	Not tested
CS1-S	Sarcoma	TM9SF2	p.R357C	Private	13	100196168	C	T	Missense_Mutation	426	0.370892019	No	1	Clonal	Disease_causing	Passenger	Neutral	passenger	No	No	No	No	Not tested
CS1-C	Carcinoma	TMED4	p.L208I	Private	7	44619140	G	T	Missense_Mutation	234	0.179487179	No	1	Clonal	Disease_causing	Passenger	Neutral	passenger	No	No	No	No	Not tested
CS1-C	Carcinoma	TMEM132D	p.T28M	Shared	12	130185240	G	A	Missense_Mutation	154	0.37012987	No	1	Clonal	Neutral	Passenger	Neutral	passenger	No	No	No	No	Not tested
CS1-S	Sarcoma	TMEM132D	p.T28M	Shared	12	130185240	G	A	Missense_Mutation	208	0.413461538	No	1	Clonal	Neutral	Passenger	Neutral	passenger	No	No	No	No	Not tested
CS1-C	Carcinoma	TMEM143	p.G297S	Private	19	48845873	C	T	Missense_Mutation	160	0.0625	No	0.598	Subclonal	Neutral	Passenger	Neutral	passenger	No	No	No	No	Not tested
CS1-S	Sarcoma	TMEM151B	p.F92S	Private	6	44240942	T	C	Missense_Mutation	383	0.031331593	No	0.11	Subclonal	Disease_causing	Passenger	.	passenger	No	No	No	No	Not tested
CS1-S	Sarcoma	TMEM181	p.R448Q	Private	6	159044617	G	A	Missense_Mutation	246	0.394308943	No	1	Clonal	Disease_causing	Passenger	Neutral	passenger	No	No	No	No	Not tested
CS1-S	Sarcoma	TMEM186	p.W150*	Private	16	8890001	C	T	Nonsense_Mutation	400	0.2825	No	0.994	Clonal	Disease_causing	.	.	passenger	No	No	No	No	Not tested
CS1-S	Sarcoma	TMEM207	p.A68V	Private	3	190158134	G	A	Missense_Mutation	139	0.064748201	No	0.228	Subclonal	Neutral	Passenger	Neutral	passenger	No	No	No	No	Not tested
CS1-C	Carcinoma	TMEM215	p.W76C	Private	9	32784409	G	T	Missense_Mutation	253	0.193675889	No	1	Clonal	Disease_causing	Passenger	.	passenger	No	No	No	No	Not tested
CS1-S	Sarcoma	TMEM223	p.R84C	Private	11	62559217	G	A	Missense_Mutation	113	0.318584071	No	1	Clonal	Neutral	Passenger	Neutral	passenger	No	No	No	No	Not tested
CS1-C	Carcinoma	TMEM229A	p.G203Afs*112	Private	7	123672449	GC	G	Frame_Shift_Del	130	0.207692308	No	1	Clonal	.	.	.	.	No	No	No	No	Not tested
CS1-S	Sarcoma	TMEM232	p.V510F	Private	5	109864464	C	A	Missense_Mutation	358	0.033519553	No	0.118	Subclonal	Disease_causing	Passenger	.	passenger	No	No	No	No	Not tested
CS1-C	Carcinoma	TMEM251	p.E70K	Private	14	93652714	G	A	Missense_Mutation	373	0.048257373	No	0.461	Subclonal	Disease_causing	Passenger	Neutral	passenger	No	No	No	No	Not tested
CS1-C	Carcinoma	TMEM57	p.A317T	Private	1	25785178	G	A	Missense_Mutation	205	0.073170732	No	0.7	Subclonal	Disease_causing	Passenger	Neutral	passenger	No	No	No	No	Not tested
CS1-S	Sarcoma	TMPRSS11A	p.I57S	Private	4	68810319	A	C	Missense_Mutation	167	0.215568862	No	0.759	Subclonal	Disease_causing	Passenger	Neutral	passenger	No	No	No	No	Not tested
CS1-C	Carcinoma	TMPRSS11B	p.I77M	Private	4	69101878	A	C	Missense_Mutation	180	0.038888889	No	0.372	Subclonal	Neutral	Passenger	Neutral	passenger	No	No	No	No	Not tested
CS1-C	Carcinoma	TMPRSS15	p.V815I	Private	21	19666630	C	T	Missense_Mutation	140	0.235714286	No	1	Clonal	Neutral	Passenger	Disease_causing	passenger	No	No	No	No	Not tested
CS1-C	Carcinoma	TMPRSS9	p.P448H	Private	19	2413888	C	A	Missense_Mutation	170	0.064705882	No	0.619	Subclonal	Neutral	Passenger	Disease_causing	passenger	No	No	No	No	Not tested
CS1-C	Carcinoma	TNIK	p.N563Qfs*12	Private	3	170846589	T	TG	Frame_Shift_Ins	185	0.205405405	No	1	Clonal	.	.	.	.	No	No	No	No	Not tested
CS1-C	Carcinoma	TNKS	p.V179M	Private	8	9413984	G	A	Missense_Mutation	272	0.0625	No	0.598	Subclonal	Disease_causing	Passenger	Neutral	passenger	No	No	No	No	Not tested
CS1-S	Sarcoma	TNPO3	p.K196*	Private	7	128645180	T	A	Nonsense_Mutation	258	0.139534884	No	0.491	Subclonal	Neutral	.	.	passenger	No	No	No	No	Not tested
CS1-C	Carcinoma	TNRC18	p.K1629E	Private	7	5396856	T	C	Missense_Mutation	136	0.066176471	No	0.633	Subclonal	Disease_causing	Passenger	Neutral	passenger	No	No	No	No	Not tested
CS1-C	Carcinoma	TNRC18	p.R807C	Private	7	5417044	G	A	Missense_Mutation	62	0.080645161	No	0.771	Clonal	Disease_causing	Passenger	Neutral	passenger	No	No	No	No	Not tested
CS1-C	Carcinoma	TNRC6C	p.T760A	Private	17	76047421	A	G	Missense_Mutation	237	0.253164557	No	1	Clonal	Neutral	Passenger	Neutral	passenger	No	No	No	No	Not tested
CS1-S	Sarcoma	TNRC6C	p.G1675C	Private	17	76100768	G	T	Missense_Mutation	66	0.166666667	No	0.586	Subclonal	Disease_causing	Passenger	Neutral	passenger	No	No	No	No	Not tested
CS1-C	Carcinoma	TOE1	p.A237V	Private	1	45808273	C	T	Missense_Mutation	279	0.035842294	No	0.343	Subclonal	Disease_causing	Passenger	Neutral	passenger	No	No	No	No	Not tested
CS1-C	Carcinoma	TOM1L2	p.A205V	Private	17	17786065	G	A	Missense_Mutation	254	0.043307087	No	0.414	Subclonal	Disease_causing	Passenger	Neutral	passenger	No	No	No	No	Not tested
CS1-S	Sarcoma	TONSL	p.V1226M	Private	8	145657727	C	T	Missense_Mutation	285	0.280701754	No	1	Clonal	Neutral	Passenger	Neutral	passenger	No	No	No	No	Not tested
CS1-C	Carcinoma	TP53INP2	p.L5I	Private	20	33296556	C	A	Missense_Mutation	195	0.123076923	No	1	Clonal	Neutral	Passenger	Neutral	passenger	No	No	No	No	Not tested
CS1-C	Carcinoma	TPH2	p.V89A	Private	12	72338084	T	C	Missense_Mutation	365	0.219178082	No	1	Clonal	Disease_causing	Passenger	Disease_causing	likely_pathogenic	No	No	No	No	Not tested
CS1-C	Carcinoma	TPR	p.R1110C	Private	1	186313596	G	A	Missense_Mutation	554	0.048736462	No	0.515	Subclonal	Disease_causing	Passenger	Neutral	passenger	No	Yes	No	No	Not tested
CS1-C	Carcinoma	TPRN	p.A398Pfs*52	Private	9	140093971	GC	G	Frame_Shift_Del	349	0.240687679	No	1	Clonal	.	.	.	.	No	No	No	No	Not tested
CS1-C	Carcinoma	TPST2	p.L38M	Private	22	26937485	G	T	Missense_Mutation	209	0.19138756	No	1	Clonal	Neutral	Passenger	.	passenger	No	No	No	No	Not tested
CS1-C	Carcinoma	TRABD2B	p.X35_splice	Private	1	48460270	C	T	Splice_Site	230	0.056521739	No	0.54	Subclonal	.	.	.	passenger	No	No	No	No	Not tested
CS1-S	Sarcoma	TRANK1	p.E1095K	Private	3	36897798	C	T	Missense_Mutation	493	0.139959432	No	0.492	Subclonal	Neutral	Passenger	Neutral	passenger	No	No	No	No	Not tested
CS1-S	Sarcoma	TRAPPC12	p.V562Cfs*35	Private	2	3469373	TG	T	Frame_Shift_Del	189	0.46031746	No	1	Clonal	.	.	.	.	No	No	No	No	Not tested
CS1-S	Sarcoma	TREM1	p.V82A	Private	6	41250294	A	G	Missense_Mutation	534	0.056179775	No	0.198	Subclonal	Neutral	Passenger	Neutral	passenger	No	No	No	No	Not tested
CS1-C	Carcinoma	TRIM3	p.D678G	Private	11	6470585	T	C	Missense_Mutation	167	0.041916168	No	0.401	Subclonal	Disease_causing	Driver	Disease_causing	likely_pathogenic	No	No	No	No	Not tested
CS1-S	Sarcoma	TRIM37	p.R14Q	Private	17	57181736	C	T	Missense_Mutation	225	0.137777778	No	0.485	Subclonal	Disease_causing	Passenger	Neutral	passenger	No	No	No	No	Not tested
CS1-S	Sarcoma	TRIM39	p.Q109Nfs*19	Private	6	30297414	GC	G	Frame_Shift_Del	416	0.098557692	No	0.347	Subclonal	.	.	.	.	No	No	No	No	Not tested
CS1-C	Carcinoma	TRIM4	p.W484Gfs*2	Private	7	99489838	CA	C	Frame_Shift_Del	315	0.225396825	No	1	Clonal	.	.	.	.	No	No	No	No	Not tested
CS1-C	Carcinoma	TRIM46	p.H455Ifs*32	Private	1	155152178	GC	G	Frame_Shift_Del	407	0.154791155	No	1	Clonal	.	.	.	.	No	No	No	No	Not tested
CS1-S	Sarcoma	TRIM46	p.G733Vfs*81	Private	1	155156579	CG	C	Frame_Shift_Del	395	0.217721519	No	0.984	Clonal	.	.	.	.	No	No	No	No	Not tested
CS1-C	Carcinoma	TRIM64C	p.I248V	Shared	11	49077921	T	C	Missense_Mutation	173	0.098265896	No	0.94	Clonal	Neutral	Passenger	Neutral	passenger	No	No	No	No	Not tested
CS1-C	Carcinoma	TRIM64C	p.D244H	Shared	11	49077933	C	G	Missense_Mutation	146	0.054794521	No	0.524	Subclonal	Neutral	Passenger	Neutral	passenger	No	No	No	No	Not tested
CS1-S	Sarcoma	TRIM64C	p.I248V	Shared	11	49077921	T	C	Missense_Mutation	205	0.07804878	No	0.275	Clonal	Neutral	Passenger	Neutral	passenger	No	No	No	No	Not tested
CS1-S	Sarcoma	TRIM64C	p.D244H	Shared	11	49077933	C	G	Missense_Mutation	180	0.055555556	No	0.195	Subclonal	Neutral	Passenger	Neutral	passenger	No	No	No	No	Not tested
CS1-C	Carcinoma	TRIO	p.L2031*	Private	5	14477003	CT	C	Frame_Shift_Del	187	0.155080214	No	1	Clonal	.	.	.	.	No	No	No	No	Not tested
CS1-S	Sarcoma	TRIO	p.R2187H	Private	5	14482785	G	A	Missense_Mutation	268	0.208955224	No	0.735	Subclonal	Disease_causing	Passenger	Neutral	passenger	No	No	No	No	Not tested
CS1-C	Carcinoma	TRIP12	p.A1332V	Private	2	230656777	G	A	Missense_Mutation	414	0.033816425	No	0.323	Subclonal	Disease_causing	Passenger	Neutral	passenger	No	No	No	No	Not tested
CS1-S	Sarcoma	TRMT112	p.V16Wfs*30	Private	11	64084952	AC	A	Frame_Shift_Del	422	0.362559242	No	1	Clonal	.	.	.	.	No	No	No	No	Not tested
CS1-S	Sarcoma	TRPM2	p.E1383K	Private	21	45857030	G	A	Missense_Mutation	349	0.040114613	No	0.141	Subclonal	Disease_causing	Passenger	Neutral	passenger	No	No	No	No	Not tested
CS1-C	Carcinoma	TRPM6	p.R952W	Private	9	77400855	G	A	Missense_Mutation	282	0.24822695	No	1	Clonal	Disease_causing	Passenger	Disease_causing	likely_pathogenic	No	No	No	No	Not tested
CS1-C	Carcinoma	TRRAP	p.A2006V	Private	7	98553869	C	T	Missense_Mutation	67	0.074626866	No	0.714	Clonal	Disease_causing	Passenger	Neutral	passenger	No	Yes	No	No	Not tested
CS1-C	Carcinoma	TSGA10	p.H678N	Private	2	99634703	G	T	Missense_Mutation	186	0.032258065	No	0.308	Subclonal	Disease_causing	Passenger	Neutral	passenger	No	No	No	No	Not tested
CS1-S	Sarcoma	TSHZ1	p.S519*	Private	18	72999049	A	AT	Frame_Shift_Ins	510	0.423529412	No	1	Clonal	.	.	.	.	No	No	No	No	Not tested
CS1-S	Sarcoma	TSKS	p.R179W	Private	19	50251386	G	A	Missense_Mutation	163	0.208588957	No	0.734	Subclonal	Disease_causing	Passenger	Neutral	passenger	No	No	No	No	Not tested
CS1-S	Sarcoma	TSPAN7	p.S173Afs*4	Private	X	38535026	TC	T	Frame_Shift_Del	168	0.410714286	No	1	Clonal	.	.	.	.	No	No	No	No	Not tested
CS1-C	Carcinoma	TSPYL4	p.R236*	Private	6	116574466	G	A	Nonsense_Mutation	254	0.05511811	No	0.527	Subclonal	Disease_causing	.	.	passenger	No	No	No	No	Not tested
CS1-S	Sarcoma	TSPYL5	p.E46Kfs*118	Private	8	98289936	TC	T	Frame_Shift_Del	565	0.571681416	No	1	Clonal	.	.	.	.	No	No	No	No	Not tested
CS1-S	Sarcoma	TST	p.P285Qfs*16	Private	22	37407107	TG	T	Frame_Shift_Del	440	0.352272727	No	1	Clonal	.	.	.	.	No	No	No	No	Not tested
CS1-C	Carcinoma	TTC21B	p.C1289Y	Private	2	166732682	C	T	Missense_Mutation	96	0.052083333	No	0.498	Subclonal	Disease_causing	Passenger	Neutral	passenger	No	No	No	No	Not tested
CS1-C	Carcinoma	TTC24	p.E214G	Private	1	156551797	A	G	Missense_Mutation	235	0.140425532	No	1	Clonal	Neutral	Passenger	Neutral	passenger	No	No	No	No	Not tested
CS1-S	Sarcoma	TTC3	p.E779Rfs*18	Private	21	38524242	C	CA	Frame_Shift_Ins	178	0.325842697	No	1	Clonal	.	.	.	.	No	No	No	No	Not tested
CS1-C	Carcinoma	TTC38	p.A214T	Private	22	46677520	G	A	Missense_Mutation	214	0.224299065	No	1	Clonal	Disease_causing	Passenger	Neutral	passenger	No	No	No	No	Not tested
CS1-C	Carcinoma	TUBAL3	p.P366L	Private	10	5435724	G	A	Missense_Mutation	311	0.041800643	No	0.4	Subclonal	Disease_causing	Passenger	Disease_causing	likely_pathogenic	No	No	No	No	Not tested
CS1-S	Sarcoma	TUBAL3	p.H68Q	Private	10	5442850	A	T	Missense_Mutation	494	0.400809717	No	1	Clonal	Disease_causing	Passenger	Neutral	passenger	No	No	No	No	Not tested
CS1-S	Sarcoma	TXK	p.H219R	Private	4	48096147	T	C	Missense_Mutation	124	0.120967742	No	0.426	Subclonal	Disease_causing	Passenger	Disease_causing	likely_pathogenic	No	No	No	No	Not tested
CS1-C	Carcinoma	TXNRD1	p.K570R	Private	12	104728073	A	G	Missense_Mutation	140	0.071428571	No	0.683	Subclonal	Disease_causing	Passenger	Disease_causing	likely_pathogenic	No	No	No	No	Not tested
CS1-S	Sarcoma	TYK2	p.H198R	Private	19	10477129	T	C	Missense_Mutation	167	0.371257485	No	1	Clonal	Neutral	Passenger	Neutral	passenger	No	No	No	No	Not tested
CS1-C	Carcinoma	TYRP1	p.N353Vfs*31	Private	9	12702410	TACAA	T	Frame_Shift_Del	84	0.19047619	No	1	Clonal	.	.	.	.	No	No	No	No	Not tested
CS1-S	Sarcoma	UBA6	p.E300*	Private	4	68530906	C	CA	Frame_Shift_Ins	232	0.357758621	No	1	Clonal	.	.	.	.	No	No	No	No	Not tested
CS1-C	Carcinoma	UBAP1	p.P183H	Private	9	34241379	C	A	Missense_Mutation	419	0.054892601	No	0.525	Subclonal	Disease_causing	Passenger	Neutral	passenger	No	No	No	No	Not tested
CS1-S	Sarcoma	UBQLNL	p.G76D	Private	11	5537445	C	T	Missense_Mutation	401	0.039900249	No	0.14	Subclonal	Disease_causing	Driver	Disease_causing	likely_pathogenic	No	No	No	No	Not tested
CS1-S	Sarcoma	UBR1	p.V1664L	Private	15	43244492	C	G	Missense_Mutation	108	0.296296296	No	1	Clonal	Disease_causing	Passenger	Neutral	passenger	No	No	No	No	Not tested
CS1-S	Sarcoma	UBR3	p.H1589Y	Private	2	170912074	C	T	Missense_Mutation	357	0.378151261	No	1	Clonal	Neutral	Passenger	Neutral	passenger	No	No	No	No	Not tested
CS1-S	Sarcoma	UBR4	p.L1930P	Private	1	19483391	A	G	Missense_Mutation	259	0.409266409	No	1	Clonal	Disease_causing	Driver	Neutral	likely_pathogenic	No	No	No	No	Not tested
CS1-S	Sarcoma	UBTD1	p.*228Wext*42	Private	10	99330280	A	G	Nonstop_Mutation	229	0.436681223	No	1	Clonal	Neutral	.	.	likely_pathogenic	No	No	No	No	Not tested
CS1-S	Sarcoma	UBXN6	p.F408del	Private	19	4445595	GAGA	G	In_Frame_Del	105	0.2	No	0.704	Subclonal	.	.	.	.	No	No	No	No	Not tested
CS1-S	Sarcoma	UCHL5	p.S13C	Private	1	193028354	T	A	Missense_Mutation	538	0.304832714	No	1	Clonal	Disease_causing	Passenger	Neutral	passenger	No	No	No	No	Not tested
CS1-C	Carcinoma	UNC45B	p.R481C	Shared	17	33495369	C	T	Missense_Mutation	172	0.412790698	No	1	Clonal	Disease_causing	Passenger	Neutral	passenger	No	No	No	No	Not tested
CS1-S	Sarcoma	UNC45B	p.R481C	Shared	17	33495369	C	T	Missense_Mutation	245	0.371428571	No	1	Clonal	Disease_causing	Passenger	Neutral	passenger	No	No	No	No	Not tested
CS1-S	Sarcoma	UNKL	p.R276H	Private	16	1447204	C	T	Missense_Mutation	352	0.428977273	No	1	Clonal	Disease_causing	Passenger	Neutral	passenger	No	No	No	No	Not tested
CS1-S	Sarcoma	UPK1A	p.A2T	Private	19	36157718	G	A	Missense_Mutation	175	0.205714286	No	0.724	Subclonal	Neutral	Passenger	Neutral	passenger	No	No	No	No	Not tested
CS1-C	Carcinoma	URB2	p.S555T	Private	1	229772024	G	C	Missense_Mutation	270	0.033333333	No	0.352	Subclonal	Disease_causing	Passenger	Neutral	passenger	No	No	No	No	Not tested
CS1-S	Sarcoma	USHBP1	p.R658C	Private	19	17361174	G	A	Missense_Mutation	109	0.04587156	No	0.161	Subclonal	Disease_causing	Passenger	Neutral	passenger	No	No	No	No	Not tested
CS1-S	Sarcoma	USP25	p.P1010S	Private	21	17250654	C	T	Missense_Mutation	306	0.359477124	No	1	Clonal	Disease_causing	Passenger	Neutral	passenger	No	No	No	No	Not tested
CS1-S	Sarcoma	USP26	p.G613Afs*26	Private	X	132160412	CT	C	Frame_Shift_Del	466	0.354077253	No	1	Clonal	.	.	.	.	No	No	No	No	Not tested
CS1-S	Sarcoma	USP35	p.T655Pfs*23	Private	11	77920855	AC	A	Frame_Shift_Del	274	0.368613139	No	1	Clonal	.	.	.	.	No	No	No	No	Not tested
CS1-C	Carcinoma	USP43	p.R292Q	Private	17	9580104	G	A	Missense_Mutation	277	0.061371841	No	0.587	Subclonal	Disease_causing	Passenger	Neutral	passenger	No	No	No	No	Not tested
CS1-C	Carcinoma	UTRN	p.R1399C	Private	6	144811267	C	T	Missense_Mutation	357	0.030812325	No	0.295	Subclonal	Neutral	Passenger	Neutral	passenger	No	No	No	No	Not tested
CS1-C	Carcinoma	VGLL1	p.W80*	Private	X	135630773	G	A	Nonsense_Mutation	301	0.046511628	No	0.445	Subclonal	Neutral	.	.	passenger	No	No	No	No	Not tested
CS1-S	Sarcoma	VILL	p.Q342R	Private	3	38040485	A	G	Missense_Mutation	340	0.147058824	No	0.517	Subclonal	Disease_causing	Passenger	Neutral	passenger	No	No	No	No	Not tested
CS1-C	Carcinoma	VNN2	p.A202T	Private	6	133073822	C	T	Missense_Mutation	302	0.043046358	No	0.412	Subclonal	Neutral	Passenger	Disease_causing	passenger	No	No	No	No	Not tested
CS1-S	Sarcoma	VPS13A	p.K372Sfs*2	Private	9	79836218	TA	T	Frame_Shift_Del	287	0.386759582	No	1	Clonal	.	.	.	.	No	No	No	No	Not tested
CS1-C	Carcinoma	VPS13C	p.Q3143H	Private	15	62176381	C	G	Missense_Mutation	192	0.171875	No	1	Clonal	Disease_causing	Passenger	Neutral	passenger	No	No	No	No	Not tested
CS1-C	Carcinoma	VPS13D	p.T1099A	Private	1	12336940	A	G	Missense_Mutation	329	0.212765957	No	1	Clonal	Disease_causing	Passenger	Neutral	passenger	No	No	No	No	Not tested
CS1-C	Carcinoma	VPS13D	p.S2336P	Private	1	12374242	T	C	Missense_Mutation	188	0.031914894	No	0.305	Subclonal	Disease_causing	Passenger	Neutral	passenger	No	No	No	No	Not tested
CS1-C	Carcinoma	VPS18	p.Q969R	Private	15	41195523	A	G	Missense_Mutation	186	0.209677419	No	1	Clonal	Disease_causing	Passenger	Neutral	passenger	No	No	No	No	Not tested
CS1-S	Sarcoma	VPS51	p.Q323H	Private	11	64875912	G	T	Missense_Mutation	329	0.03343465	No	0.118	Subclonal	Disease_causing	Passenger	Neutral	passenger	No	No	No	No	Not tested
CS1-S	Sarcoma	VSX2	p.G74C	Private	14	74706484	G	T	Missense_Mutation	226	0.030973451	No	0.109	Subclonal	Disease_causing	Passenger	Disease_causing	likely_pathogenic	No	No	No	No	Not tested
CS1-C	Carcinoma	VWA8	p.X784_splice	Private	13	42335341	C	T	Splice_Site	208	0.033653846	No	0.322	Subclonal	Disease_causing	.	.	passenger	No	No	No	No	Not tested
CS1-C	Carcinoma	VWA8	p.I1889V	Private	13	42142386	T	C	Missense_Mutation	163	0.073619632	No	0.704	Subclonal	Disease_causing	Passenger	Neutral	passenger	No	No	No	No	Not tested
CS1-S	Sarcoma	VWA8	p.A457T	Private	13	42439928	C	T	Missense_Mutation	312	0.153846154	No	0.541	Subclonal	Disease_causing	Passenger	Neutral	passenger	No	No	No	No	Not tested
CS1-C	Carcinoma	VWCE	p.P869H	Private	11	61026409	G	T	Missense_Mutation	110	0.163636364	No	1	Clonal	Neutral	Passenger	Neutral	passenger	No	No	No	No	Not tested
CS1-S	Sarcoma	WASL	p.T23A	Private	7	123388722	T	C	Missense_Mutation	496	0.225806452	No	0.795	Subclonal	Disease_causing	Passenger	Disease_causing	likely_pathogenic	No	No	No	No	Not tested
CS1-S	Sarcoma	WBP1	p.P140Lfs*10	Private	2	74687409	AC	A	Frame_Shift_Del	385	0.532467532	No	1	Clonal	.	.	.	.	No	No	No	No	Not tested
CS1-C	Carcinoma	WBSCR22	p.E9K	Private	7	73097980	G	A	Missense_Mutation	135	0.125925926	No	1	Clonal	Disease_causing	Passenger	Neutral	passenger	No	No	No	No	Not tested
CS1-S	Sarcoma	WDFY3	p.L2656Wfs*3	Private	4	85634386	CA	C	Frame_Shift_Del	240	0.35	No	1	Clonal	.	.	.	.	No	No	No	No	Not tested
CS1-S	Sarcoma	WDFY3	p.L1060Cfs*41	Private	4	85716120	CA	C	Frame_Shift_Del	264	0.215909091	No	0.76	Subclonal	.	.	.	.	No	No	No	No	Not tested
CS1-C	Carcinoma	WDR11	p.V688A	Private	10	122646290	T	C	Missense_Mutation	222	0.058558559	No	0.56	Subclonal	Disease_causing	Driver	Neutral	likely_pathogenic	No	No	No	No	Not tested
CS1-S	Sarcoma	WDR37	p.L491P	Private	10	1175271	T	C	Missense_Mutation	604	0.258278146	No	0.909	Clonal	Disease_causing	Passenger	Neutral	passenger	No	No	No	No	Not tested
CS1-S	Sarcoma	WDR47	p.R81Gfs*7	Private	1	109560140	CT	C	Frame_Shift_Del	264	0.113636364	No	0.4	Subclonal	.	.	.	.	No	No	No	No	Not tested
CS1-C	Carcinoma	WDR60	p.N527Ifs*23	Private	7	158704352	GA	G	Frame_Shift_Del	161	0.173913043	No	1	Clonal	.	.	.	.	No	No	No	No	Not tested
CS1-S	Sarcoma	WDR87	p.R2063Gfs*11	Private	19	38378006	CT	C	Frame_Shift_Del	302	0.374172185	No	1	Clonal	.	.	.	.	No	No	No	No	Not tested
CS1-C	Carcinoma	WDR96	p.A432V	Private	10	105953771	G	A	Missense_Mutation	296	0.067567568	No	0.646	Subclonal	Neutral	Passenger	Neutral	passenger	No	No	No	No	Not tested
CS1-C	Carcinoma	WFDC5	p.C34Y	Private	20	43739401	C	T	Missense_Mutation	289	0.221453287	No	1	Clonal	Disease_causing	Passenger	Disease_causing	likely_pathogenic	No	No	No	No	Not tested
CS1-S	Sarcoma	WNT5B	p.R259C	Private	12	1755113	C	T	Missense_Mutation	353	0.254957507	No	0.897	Subclonal	Disease_causing	Passenger	Neutral	passenger	No	No	No	No	Not tested
CS1-S	Sarcoma	XIRP1	p.A1299V	Private	3	39227041	G	A	Missense_Mutation	151	0.337748344	No	1	Clonal	Disease_causing	Passenger	Neutral	passenger	No	No	No	No	Not tested
CS1-S	Sarcoma	XIRP1	p.W1135Gfs*2	Private	3	39227534	AC	A	Frame_Shift_Del	534	0.348314607	No	1	Clonal	.	.	.	.	No	No	No	No	Not tested
CS1-C	Carcinoma	XPO5	p.A217V	Shared	6	43535090	G	A	Missense_Mutation	132	0.151515152	No	1	Clonal	Disease_causing	Passenger	Neutral	passenger	No	No	No	No	Not tested
CS1-S	Sarcoma	XPO5	p.A217V	Shared	6	43535090	G	A	Missense_Mutation	164	0.420731707	No	1	Clonal	Disease_causing	Passenger	Neutral	passenger	No	No	No	No	Not tested
CS1-C	Carcinoma	XYLT1	p.D796G	Private	16	17211673	T	C	Missense_Mutation	351	0.031339031	No	0.3	Subclonal	Disease_causing	Passenger	Neutral	passenger	No	No	No	No	Not tested
CS1-S	Sarcoma	YDJC	p.Q292*	Private	22	21982805	G	A	Nonsense_Mutation	194	0.360824742	No	1	Clonal	Disease_causing	.	.	passenger	No	No	No	No	Not tested
CS1-S	Sarcoma	YIF1A	p.P165L	Private	11	66052999	G	A	Missense_Mutation	273	0.230769231	No	0.812	Subclonal	Disease_causing	Passenger	Neutral	passenger	No	No	No	No	Not tested
CS1-C	Carcinoma	YTHDF3	p.H517P	Shared	8	64100122	A	C	Missense_Mutation	378	0.338624339	No	1	Clonal	.	.	.	passenger	No	No	No	No	Not tested
CS1-S	Sarcoma	YTHDF3	p.H517P	Shared	8	64100122	A	C	Missense_Mutation	769	0.613784135	No	1	Clonal	.	.	.	passenger	No	No	No	No	Not tested
CS1-S	Sarcoma	YWHAQ	p.K11E	Private	2	9770551	T	C	Missense_Mutation	342	0.447368421	No	1	Clonal	Disease_causing	Passenger	Neutral	passenger	No	No	No	No	Not tested
CS1-S	Sarcoma	ZAR1	p.C20Afs*50	Private	4	48492358	GC	G	Frame_Shift_Del	410	0.124390244	No	0.438	Subclonal	.	.	.	.	No	No	No	No	Not tested
CS1-S	Sarcoma	ZBED3	p.A32T	Private	5	76373610	C	T	Missense_Mutation	106	0.216981132	No	0.764	Subclonal	Neutral	Passenger	.	passenger	No	No	No	No	Not tested
CS1-S	Sarcoma	ZBTB1	p.S383Vfs*6	Private	14	64989361	TA	T	Frame_Shift_Del	382	0.240837696	No	0.847	Subclonal	.	.	.	.	No	No	No	No	Not tested
CS1-S	Sarcoma	ZBTB1	p.E187G	Private	14	64988782	A	G	Missense_Mutation	470	0.419148936	No	1	Clonal	Disease_causing	Driver	Neutral	likely_pathogenic	No	No	No	No	Not tested
CS1-C	Carcinoma	ZBTB18	p.R195*	Private	1	244217659	C	T	Nonsense_Mutation	358	0.189944134	No	1	Clonal	Disease_causing	.	.	passenger	No	No	No	No	Not tested
CS1-S	Sarcoma	ZBTB20	p.E574G	Private	3	114069204	T	C	Missense_Mutation	346	0.355491329	No	1	Clonal	Disease_causing	Passenger	Neutral	passenger	No	No	No	No	Not tested
CS1-S	Sarcoma	ZBTB45	p.A252V	Private	19	59028286	G	A	Missense_Mutation	307	0.071661238	No	0.252	Subclonal	Neutral	Passenger	Neutral	passenger	No	No	No	No	Not tested
CS1-C	Carcinoma	ZBTB49	p.P422S	Private	4	4307873	C	T	Missense_Mutation	182	0.049450549	No	0.473	Subclonal	Disease_causing	Passenger	Neutral	passenger	No	No	No	No	Not tested
CS1-C	Carcinoma	ZBTB7B	p.R481C	Shared	1	154988880	C	T	Missense_Mutation	344	0.226744186	No	1	Clonal	Disease_causing	Passenger	Neutral	passenger	No	No	No	No	Not tested
CS1-S	Sarcoma	ZBTB7B	p.R481C	Shared	1	154988880	C	T	Missense_Mutation	532	0.27443609	No	1	Clonal	Disease_causing	Passenger	Neutral	passenger	No	No	No	No	Not tested
CS1-S	Sarcoma	ZC3H13	p.S1190N	Private	13	46543110	C	T	Missense_Mutation	541	0.040665434	No	0.143	Subclonal	Disease_causing	Passenger	Neutral	passenger	No	No	No	No	Not tested
CS1-S	Sarcoma	ZC3H18	p.A41V	Private	16	88643653	C	T	Missense_Mutation	126	0.46031746	No	1	Clonal	Neutral	Passenger	Neutral	passenger	No	No	No	No	Not tested

CS1-C	Carcinoma	ZCCHC12	p.G354D	Private	X	117960268	G	A	Missense_Mutation	[illegible]	[illegible]	No	0.792	Clonal	Neutral	Passenger	Disease_causing	passenger	No	No	No	No	Not tested
CS1-S	Sarcoma	ZCRB1	p.W185Mfs*13	Private	12	42706970	A	AT	Frame_Shift_Ins	[illegible]	[illegible]	No	1	Clonal	.	.	.	.	No	No	No	No	Not tested
CS1-S	Sarcoma	ZDHHC9	p.R58S	Private	X	128963113	G	T	Missense_Mutation	117	0.162393162	No	0.571	Subclonal	Disease_causing	Passenger	Neutral	passenger	No	No	No	No	Not tested
CS1-C	Carcinoma	ZFAND4	p.L624Cfs*8	Private	10	46121399	CA	C	Frame_Shift_Del	193	0.113989637	No	1	Clonal	.	.	.	.	No	No	No	No	Not tested
CS1-S	Sarcoma	ZFC3H1	p.K385Nfs*9	Private	12	72038780	AT	A	Frame_Shift_Del	169	0.100591716	No	0.354	Subclonal	.	.	.	.	No	No	No	No	Not tested
CS1-C	Carcinoma	ZFHX3	p.R1119*	Shared	16	72923723	G	A	Nonsense_Mutation	122	0.344262295	No	1	Clonal	Neutral	.	.	likely_pathogenic	No	Yes	No	Yes	Not tested
CS1-S	Sarcoma	ZFHX3	p.X907_splice	Private	16	72984865	C	T	Splice_Site	137	0.489051095	No	1	Clonal	Disease_causing	.	.	likely_pathogenic	No	Yes	No	Yes	Not tested
CS1-S	Sarcoma	ZFHX3	p.R1119*	Shared	16	72923723	G	A	Nonsense_Mutation	136	0.448529412	No	1	Clonal	Neutral	.	.	likely_pathogenic	No	Yes	No	Yes	Not tested
CS1-C	Carcinoma	ZFHX4	p.C1247Y	Private	8	77761842	G	A	Missense_Mutation	226	0.207964602	No	1	Clonal	Disease_causing	Passenger	Neutral	passenger	No	No	No	No	Not tested
CS1-S	Sarcoma	ZFP62	p.S574Efs*6	Private	5	180276773	CGA	C	Frame_Shift_Del	219	0.127853881	No	0.45	Subclonal	.	.	.	.	No	No	No	No	Not tested
CS1-S	Sarcoma	ZFYVE1	p.P423Sfs*3	Private	14	73448540	G	GA	Frame_Shift_Ins	221	0.402714932	No	1	Clonal	.	.	.	.	No	No	No	No	Not tested
CS1-C	Carcinoma	ZFYVE26	p.A1456V	Private	14	68249502	G	A	Missense_Mutation	245	0.057142857	No	0.546	Subclonal	Neutral	Passenger	Neutral	passenger	No	No	No	No	Not tested
CS1-C	Carcinoma	ZIC1	p.F272L	Private	3	147128715	C	G	Missense_Mutation	484	0.045454545	No	0.435	Subclonal	Disease_causing	Passenger	Disease_causing	likely_pathogenic	No	No	No	No	Not tested
CS1-S	Sarcoma	ZKSCAN5	p.A613S	Private	7	99129189	G	T	Missense_Mutation	436	0.247706422	No	0.872	Subclonal	Neutral	Passenger	Neutral	passenger	No	No	No	No	Not tested
CS1-S	Sarcoma	ZMYM2	p.Q526*	Private	13	20593750	C	T	Nonsense_Mutation	398	0.399497487	No	1	Clonal	Neutral	.	.	likely_pathogenic	No	Yes	No	No	Not tested
CS1-S	Sarcoma	ZNF132	p.A178T	Private	19	58946279	C	T	Missense_Mutation	327	0.412844037	No	1	Clonal	Neutral	Passenger	Neutral	passenger	No	No	No	No	Not tested
CS1-S	Sarcoma	ZNF134	p.A408V	Private	19	58132710	C	T	Missense_Mutation	328	0.298780488	No	1	Clonal	Neutral	Passenger	Neutral	passenger	No	No	No	No	Not tested
CS1-C	Carcinoma	ZNF136	p.L323I	Private	19	12298160	C	A	Missense_Mutation	116	0.043103448	No	0.412	Subclonal	Neutral	Passenger	Neutral	passenger	No	No	No	No	Not tested
CS1-C	Carcinoma	ZNF195	p.K352Nfs*23	Private	11	3381181	GT	G	Frame_Shift_Del	279	0.225806452	No	1	Clonal	.	.	.	.	No	No	No	No	Not tested
CS1-S	Sarcoma	ZNF408	p.Q108H	Private	11	46723220	G	T	Missense_Mutation	300	0.036666667	No	0.129	Subclonal	Neutral	Passenger	Neutral	passenger	No	No	No	No	Not tested
CS1-C	Carcinoma	ZNF416	p.P79H	Private	19	58085036	G	T	Missense_Mutation	335	0.035820896	No	0.342	Subclonal	Neutral	Passenger	Neutral	passenger	No	No	No	No	Not tested
CS1-C	Carcinoma	ZNF425	p.R245Q	Private	7	148802229	C	T	Missense_Mutation	301	0.202657807	No	1	Clonal	Neutral	Passenger	Neutral	passenger	No	No	No	No	Not tested
CS1-C	Carcinoma	ZNF431	p.V63G	Private	19	21349229	T	G	Missense_Mutation	59	0.101694915	No	0.972	Clonal	Neutral	Passenger	Neutral	passenger	No	No	No	No	Not tested
CS1-S	Sarcoma	ZNF449	p.P277Qfs*8	Private	X	134494267	AC	A	Frame_Shift_Del	362	0.218232044	No	0.768	Subclonal	.	.	.	.	No	No	No	No	Not tested
CS1-S	Sarcoma	ZNF460	p.P490S	Private	19	57803377	C	T	Missense_Mutation	411	0.194647202	No	0.685	Subclonal	Neutral	Passenger	Neutral	passenger	No	No	No	No	Not tested
CS1-C	Carcinoma	ZNF469	p.S2918G	Private	16	88502714	A	G	Missense_Mutation	180	0.055555556	No	0.531	Subclonal	Neutral	Passenger	Neutral	passenger	No	No	No	No	Not tested
CS1-S	Sarcoma	ZNF524	p.D88N	Private	19	56113740	G	A	Missense_Mutation	399	0.220551378	No	0.776	Subclonal	Neutral	Passenger	Neutral	passenger	No	No	No	No	Not tested
CS1-C	Carcinoma	ZNF615	p.K129T	Private	19	52497976	T	G	Missense_Mutation	282	0.226950355	No	1	Clonal	Neutral	Passenger	Neutral	passenger	No	No	No	No	Not tested
CS1-S	Sarcoma	ZNF628	p.T214A	Private	19	55993200	A	G	Missense_Mutation	335	0.367164179	No	1	Clonal	Disease_causing	Passenger	Neutral	passenger	No	No	No	No	Not tested
CS1-C	Carcinoma	ZNF638	p.G1305S	Private	2	71650557	G	A	Missense_Mutation	339	0.085545723	No	0.903	Clonal	Neutral	Passenger	Neutral	passenger	No	No	No	No	Not tested
CS1-S	Sarcoma	ZNF638	p.N1571Mfs*9	Private	2	71653706	CA	C	Frame_Shift_Del	280	0.371428571	No	1	Clonal	.	.	.	.	No	No	No	No	Not tested
CS1-C	Carcinoma	ZNF662	p.R258*	Private	3	42956259	C	T	Nonsense_Mutation	251	0.039840637	No	0.381	Subclonal	Disease_causing	.	.	passenger	No	No	No	No	Not tested
CS1-S	Sarcoma	ZNF668	p.A194T	Private	16	31075270	C	T	Missense_Mutation	235	0.365957447	No	1	Clonal	Neutral	Passenger	Neutral	passenger	No	No	No	No	Not tested
CS1-C	Carcinoma	ZNF710	p.L394F	Private	15	90611549	C	T	Missense_Mutation	216	0.064814815	No	0.62	Subclonal	Disease_causing	Passenger	Neutral	passenger	No	No	No	No	Not tested
CS1-C	Carcinoma	ZNF710	p.T651I	Private	15	90623018	C	T	Missense_Mutation	220	0.081818182	No	0.782	Clonal	Neutral	Passenger	Neutral	passenger	No	No	No	No	Not tested
CS1-C	Carcinoma	ZNF775	p.T158M	Private	7	150094042	C	T	Missense_Mutation	290	0.165517241	No	1	Clonal	Disease_causing	Passenger	Neutral	passenger	No	No	No	No	Not tested
CS1-S	Sarcoma	ZNF791	p.K569Nfs*3	Private	19	12740043	TA	T	Frame_Shift_Del	286	0.377622378	No	1	Clonal	.	.	.	.	No	No	No	No	Not tested
CS1-S	Sarcoma	ZNF862	p.A771V	Private	7	149558561	C	T	Missense_Mutation	241	0.165975104	No	0.584	Subclonal	Neutral	Passenger	Neutral	passenger	No	No	No	No	Not tested
CS1-C	Carcinoma	ZRSR2	p.R433H	Private	X	15841214	G	A	Missense_Mutation	210	0.033333333	No	0.319	Subclonal	Disease_causing	Passenger	Neutral	passenger	No	Yes	No	No	Not tested
CS1-C	Carcinoma	ZSCAN1	p.A277T	Private	19	58565021	G	A	Missense_Mutation	260	0.038461538	No	0.368	Subclonal	Neutral	Passenger	Neutral	passenger	No	No	No	No	Not tested
CS1-C	Carcinoma	ZSCAN29	p.Q469R	Private	15	43656397	T	C	Missense_Mutation	352	0.039772727	No	0.38	Subclonal	Disease_causing	Passenger	Neutral	passenger	No	No	No	No	Not tested
CS1-C	Carcinoma	ZSWIM5	p.S113P	Private	1	45671686	A	G	Missense_Mutation	144	0.25	No	1	Clonal	Disease_causing	Passenger	Neutral	passenger	No	No	No	No	Not tested
CS3-C	Carcinoma	ADCY9	p.R72W	Shared	16	4165230	G	A	Missense_Mutation	163	0.26993865	No	1	Clonal	Disease_causing	Passenger	Neutral	passenger	No	No	No	No	Not tested
CS3-S	Sarcoma	ADCY9	p.R72W	Shared	16	4165230	G	A	Missense_Mutation	174	0.275862069	No	1	Clonal	Disease_causing	Passenger	Neutral	passenger	No	No	No	No	Not tested
CS3-C	Carcinoma	ALCAM	p.N273S	Shared	3	105258906	A	G	Missense_Mutation	217	0.940092166	No	1	Clonal	Disease_causing	Passenger	Neutral	passenger	Yes	No	No	No	Not tested
CS3-S	Sarcoma	ALCAM	p.N273S	Shared	3	105258906	A	G	Missense_Mutation	304	0.782894737	No	1	Clonal	Disease_causing	Passenger	Neutral	passenger	Yes	No	No	No	Not tested
CS3-C	Carcinoma	ARID1A	p.Y534*	Shared	1	27057894	C	G	Nonsense_Mutation	282	0.393617021	No	1	Clonal	Disease_causing	.	.	likely_pathogenic	No	Yes	Yes	Yes	Not tested
CS3-S	Sarcoma	ARID1A	p.Y534*	Shared	1	27057894	C	G	Nonsense_Mutation	350	0.328571429	No	1	Clonal	Disease_causing	.	.	likely_pathogenic	No	Yes	Yes	Yes	Not tested
CS3-C	Carcinoma	CHD4	p.F1112S	Shared	12	6700637	A	G	Missense_Mutation	116	0.629310345	No	1	Clonal	Disease_causing	Passenger	Disease_causing	likely_pathogenic	No	Yes	No	Yes	Not tested
CS3-S	Sarcoma	CHD4	p.F1112S	Shared	12	6700637	A	G	Missense_Mutation	129	0.565891473	No	1	Clonal	Disease_causing	Passenger	Disease_causing	likely_pathogenic	No	Yes	No	Yes	Not tested
CS3-C	Carcinoma	CMYA5	p.V299F	Shared	5	79025483	G	T	Missense_Mutation	267	0.220973783	No	1	Clonal	Neutral	Passenger	Neutral	passenger	No	No	No	No	Not tested
CS3-S	Sarcoma	CMYA5	p.V299F	Shared	5	79025483	G	T	Missense_Mutation	380	0.194736842	No	1	Clonal	Neutral	Passenger	Neutral	passenger	No	No	No	No	Not tested
CS3-C	Carcinoma	CTCF	p.A127Pfs*4	Shared	16	67645111	GTTGC	G	Frame_Shift_Del	260	0.569230769	No	1	Clonal	.	.	.	.	No	Yes	Yes	Yes	Not tested
CS3-S	Sarcoma	CTCF	p.A127Pfs*4	Shared	16	67645111	GTTGC	G	Frame_Shift_Del	327	0.498470948	No	1	Clonal	.	.	.	.	No	Yes	Yes	Yes	Not tested
CS3-C	Carcinoma	DARS2	p.F355C	Shared	1	173810047	T	G	Missense_Mutation	239	0.209205021	No	1	Clonal	Disease_causing	Passenger	Disease_causing	likely_pathogenic	No	No	No	No	Not tested
CS3-S	Sarcoma	DARS2	p.F355C	Shared	1	173810047	T	G	Missense_Mutation	306	0.163398693	No	1	Clonal	Disease_causing	Passenger	Disease_causing	likely_pathogenic	No	No	No	No	Not tested
CS3-C	Carcinoma	DLG3	p.R497Q	Shared	X	69699084	G	A	Missense_Mutation	225	0.444444444	No	1	Clonal	Disease_causing	Passenger	Neutral	passenger	No	No	No	No	Not tested
CS3-S	Sarcoma	DLG3	p.R497Q	Shared	X	69699084	G	A	Missense_Mutation	264	0.416666667	No	1	Clonal	Disease_causing	Passenger	Neutral	passenger	No	No	No	No	Not tested
CS3-C	Carcinoma	DMD	p.S2065I	Shared	X	32305742	C	A	Missense_Mutation	489	0.263803681	No	1	Clonal	Disease_causing	Passenger	Neutral	passenger	No	No	No	No	Not tested
CS3-S	Sarcoma	DMD	p.S2065I	Shared	X	32305742	C	A	Missense_Mutation	588	0.272108844	No	1	Clonal	Disease_causing	Passenger	Neutral	passenger	No	No	No	No	Not tested
CS3-C	Carcinoma	DMTN	p.T203R	Shared	8	21929840	C	G	Missense_Mutation	110	0.154545455	No	0.978	Clonal	Disease_causing	Passenger	Neutral	passenger	No	No	No	No	Not tested
CS3-S	Sarcoma	DMTN	p.T203R	Shared	8	21929840	C	G	Missense_Mutation	167	0.101796407	No	0.788	Clonal	Disease_causing	Passenger	Neutral	passenger	No	No	No	No	Not tested
CS3-C	Carcinoma	DSCR3	p.E196K	Shared	21	38600596	C	T	Missense_Mutation	95	0.410526316	No	1	Clonal	Disease_causing	Passenger	.	passenger	No	No	No	No	Not tested
CS3-S	Sarcoma	DSCR3	p.E196K	Shared	21	38600596	C	T	Missense_Mutation	102	0.284313725	No	1	Clonal	Disease_causing	Passenger	.	passenger	No	No	No	No	Not tested
CS3-C	Carcinoma	EFHC1	p.R276Q	Shared	6	52318996	G	A	Missense_Mutation	88	0.215909091	No	1	Clonal	Disease_causing	Passenger	Neutral	passenger	No	No	No	No	Not tested
CS3-S	Sarcoma	EFHC1	p.R276Q	Shared	6	52318996	G	A	Missense_Mutation	127	0.204724409	No	1	Clonal	Disease_causing	Passenger	Neutral	passenger	No	No	No	No	Not tested
CS3-C	Carcinoma	EP300	p.L18Tfs*21	Shared	22	41489056	T	TA	Frame_Shift_Ins	274	0.321167883	No	1	Clonal	.	.	.	.	No	Yes	Yes	Yes	Not tested
CS3-S	Sarcoma	EP300	p.L18Tfs*21	Shared	22	41489056	T	TA	Frame_Shift_Ins	334	0.293413174	No	1	Clonal	.	.	.	.	No	Yes	Yes	Yes	Not tested
CS3-C	Carcinoma	EPB41	p.T301I	Shared	1	29342276	C	T	Missense_Mutation	258	0.174418605	No	1	Clonal	Disease_causing	Passenger	Neutral	passenger	No	No	No	No	Not tested
CS3-S	Sarcoma	EPB41	p.T301I	Shared	1	29342276	C	T	Missense_Mutation	324	0.160493827	No	1	Clonal	Disease_causing	Passenger	Neutral	passenger	No	No	No	No	Not tested
CS3-C	Carcinoma	FXYD5	p.R145W	Shared	19	35657174	C	T	Missense_Mutation	286	0.412587413	No	1	Clonal	Disease_causing	Passenger	Neutral	passenger	No	No	No	No	Not tested
CS3-S	Sarcoma	FXYD5	p.R145W	Shared	19	35657174	C	T	Missense_Mutation	404	0.415841584	No	1	Clonal	Disease_causing	Passenger	Neutral	passenger	No	No	No	No	Not tested
CS3-C	Carcinoma	GAS8	p.Q30R	Shared	16	90094129	A	G	Missense_Mutation	130	0.584615385	No	1	Clonal	Disease_causing	Passenger	Neutral	passenger	No	No	No	No	Not tested
CS3-S	Sarcoma	GAS8	p.Q30R	Shared	16	90094129	A	G	Missense_Mutation	193	0.518134715	No	1	Clonal	Disease_causing	Passenger	Neutral	passenger	No	No	No	No	Not tested
CS3-C	Carcinoma	GDF7	p.P255L	Shared	2	20870596	C	T	Missense_Mutation	73	0.630136986	No	1	Clonal	Neutral	Passenger	Neutral	passenger	Yes	No	No	No	Not tested
CS3-S	Sarcoma	GDF7	p.P255L	Shared	2	20870596	C	T	Missense_Mutation	126	0.69047619	No	1	Clonal	Neutral	Passenger	Neutral	passenger	Yes	No	No	No	Not tested
CS3-C	Carcinoma	GPAM	p.R278*	Shared	10	113928673	G	A	Nonsense_Mutation	319	0.764890282	No	1	Clonal	Neutral	.	.	passenger	No	No	No	No	Not tested
CS3-S	Sarcoma	GPAM	p.R278*	Shared	10	113928673	G	A	Nonsense_Mutation	446	0.661434978	No	1	Clonal	Neutral	.	.	passenger	No	No	No	No	Not tested
CS3-C	Carcinoma	GRIA1	p.V747M	Shared	5	153149914	G	A	Missense_Mutation	188	0.244680851	No	1	Clonal	Disease_causing	Passenger	Neutral	passenger	No	No	No	No	Not tested
CS3-S	Sarcoma	GRIA1	p.V747M	Shared	5	153149914	G	A	Missense_Mutation	277	0.19133574	No	1	Clonal	Disease_causing	Passenger	Neutral	passenger	No	No	No	No	Not tested
CS3-C	Carcinoma	HKDC1	p.R396C	Shared	10	71007270	C	T	Missense_Mutation	266	0.165413534	No	1	Clonal	Disease_causing	Passenger	Disease_causing	likely_pathogenic	No	No	No	No	Not tested
CS3-S	Sarcoma	HKDC1	p.R396C	Shared	10	71007270	C	T	Missense_Mutation	342	0.201754386	No	1	Clonal	Disease_causing	Passenger	Disease_causing	likely_pathogenic	No	No	No	No	Not tested
CS3-C	Carcinoma	KCND3	p.S560P	Shared	1	112319736	A	G	Missense_Mutation	84	0.154761905	No	1	Clonal	Disease_causing	Passenger	Disease_causing	likely_pathogenic	No	No	No	No	Not tested
CS3-S	Sarcoma	KCND3	p.S560P	Shared	1	112319736	A	G	Missense_Mutation	119	0.243697479	No	1	Clonal	Disease_causing	Passenger	Disease_causing	likely_pathogenic	No	No	No	No	Not tested
CS3-C	Carcinoma	KMT2B	p.P1820S	Private	19	36222829	C	T	Missense_Mutation	238	0.163865546	No	0.873	Clonal	Neutral	Passenger	Disease_causing	passenger	No	No	No	No	Not tested
CS3-C	Carcinoma	KMT2B	p.G1741D	Shared	19	36221463	G	A	Missense_Mutation	279	0.448028674	No	1	Clonal	Disease_causing	Passenger	Disease_causing	likely_pathogenic	No	No	No	No	Not tested
CS3-S	Sarcoma	KMT2B	p.G1741D	Shared	19	36221463	G	A	Missense_Mutation	392	0.413265306	No	1	Clonal	Disease_causing	Passenger	Disease_causing	likely_pathogenic	No	No	No	No	Not tested
CS3-C	Carcinoma	KRAS	p.G12V	Shared	12	25398284	C	A	Missense_Mutation	62	0.612903226	Yes	1	Clonal	Disease_causing	Driver	Neutral	likely_pathogenic	No	Yes	Yes	Yes	Not tested
CS3-S	Sarcoma	KRAS	p.G12V	Shared	12	25398284	C	A	Missense_Mutation	115	0.591304348	Yes	1	Clonal	Disease_causing	Driver	Neutral	likely_pathogenic	No	Yes	Yes	Yes	Not tested
CS3-S	Sarcoma	LAMA5	p.A907T	Private	20	60909262	C	T	Missense_Mutation	284	0.080985915	No	0.627	Subclonal	Disease_causing	Passenger	Neutral	passenger	No	No	No	No	Yes
CS3-C	Carcinoma	MAGED1	p.W436*	Private	X	51639890	G	A	Nonsense_Mutation	326	0.033742331	No	0.112	Subclonal	Neutral	.	.	passenger	No	No	No	No	Not tested
CS3-C	Carcinoma	NOB1	p.R282Q	Shared	16	69778900	C	T	Missense_Mutation	161	0.273291925	No	1	Clonal	Disease_causing	Passenger	Neutral	passenger	No	No	No	No	Not tested
CS3-S	Sarcoma	NOB1	p.R282Q	Shared	16	69778900	C	T	Missense_Mutation	267	0.27340824	No	1	Clonal	Disease_causing	Passenger	Neutral	passenger	No	No	No	No	Not tested
CS3-C	Carcinoma	NUP205	p.L1767F	Shared	7	135323338	C	T	Missense_Mutation	191	0.146596859	No	0.781	Clonal	Disease_causing	Passenger	Neutral	passenger	No	No	No	No	Not tested
CS3-S	Sarcoma	NUP205	p.L1767F	Shared	7	135323338	C	T	Missense_Mutation	245	0.244897959	No	1	Clonal	Disease_causing	Passenger	Neutral	passenger	No	No	No	No	Not tested
CS3-C	Carcinoma	OR8K3	p.I91V	Shared	11	56086053	A	G	Missense_Mutation	348	0.290229885	No	1	Clonal	Neutral	Passenger	Neutral	passenger	No	No	No	No	Not tested
CS3-S	Sarcoma	OR8K3	p.I91V	Shared	11	56086053	A	G	Missense_Mutation	439	0.23690205	No	1	Clonal	Neutral	Passenger	Neutral	passenger	No	No	No	No	Not tested
CS3-C	Carcinoma	PCDHA7	p.A720V	Shared	5	140216127	C	T	Missense_Mutation	300	0.693333333	No	1	Clonal	Neutral	Passenger	Neutral	passenger	No	No	No	No	Not tested
CS3-S	Sarcoma	PCDHA7	p.A720V	Shared	5	140216127	C	T	Missense_Mutation	412	0.597087379	No	1	Clonal	Neutral	Passenger	Neutral	passenger	No	No	No	No	Not tested
CS3-C	Carcinoma	PDHX	p.P246L	Shared	11	34988282	C	T	Missense_Mutation	161	0.124223602	No	0.91	Clonal	Disease_causing	Passenger	Neutral	passenger	No	No	No	No	Not tested
CS3-S	Sarcoma	PDHX	p.P246L	Shared	11	34988282	C	T	Missense_Mutation	247	0.157894737	No	1	Clonal	Disease_causing	Passenger	Neutral	passenger	No	No	No	No	Not tested
CS3-C	Carcinoma	PDK2	p.K209N	Private	17	48184459	G	C	Missense_Mutation	252	0.115079365	No	0.728	Subclonal	Disease_causing	Passenger	Neutral	passenger	No	No	No	No	Not tested
CS3-C	Carcinoma	PDK3	p.S346T	Shared	X	24549846	T	A	Missense_Mutation	630	0.211111111	No	1	Clonal	Disease_causing	Driver	Disease_causing	likely_pathogenic	No	No	No	No	Not tested
CS3-S	Sarcoma	PDK3	p.S346T	Shared	X	24549846	T	A	Missense_Mutation	813	0.247232472	No	1	Clonal	Disease_causing	Driver	Disease_causing	likely_pathogenic	No	No	No	No	Not tested
CS3-S	Sarcoma	PGRMC1	p.V97L	Private	X	118370615	G	T	Missense_Mutation	391	0.040920716	No	0.317	Subclonal	Disease_causing	Passenger	Disease_causing	likely_pathogenic	No	No	No	No	Yes
CS3-C	Carcinoma	PIK3CA	p.G106V	Shared	3	178916930	G	T	Missense_Mutation	194	0.927835052	Yes	1	Clonal	Disease_causing	Driver	Neutral	likely_pathogenic	Yes	Yes	Yes	Yes	Not tested
CS3-S	Sarcoma	PIK3CA	p.G106V	Shared	3	178916930	G	T	Missense_Mutation	276	0.77173913	Yes	1	Clonal	Disease_causing	Driver	Neutral	likely_pathogenic	Yes	Yes	Yes	Yes	Not tested
CS3-S	Sarcoma	PLXNA3	p.K1393R	Private	X	153696860	A	G	Missense_Mutation	145	0.04137931	No	0.321	Subclonal	Disease_causing	Passenger	Neutral	passenger	No	No	No	No	Yes
CS3-C	Carcinoma	PPP2R1A	p.S256Y	Shared	19	52716323	C	A	Missense_Mutation	330	0.448484848	No	1	Clonal	Disease_causing	Driver	Neutral	likely_pathogenic	No	Yes	Yes	Yes	Not tested
CS3-S	Sarcoma	PPP2R1A	p.S256Y	Shared	19	52716323	C	A	Missense_Mutation	397	0.44836272	No	1	Clonal	Disease_causing	Driver	Neutral	likely_pathogenic	No	Yes	Yes	Yes	Not tested
CS3-C	Carcinoma	PTEN	p.X342_splice	Shared	10	89720877	T	G	Splice_Site	89	0.247191011	No	1	Clonal	Disease_causing	.	.	likely_pathogenic	No	Yes	Yes	Yes	Not tested
CS3-S	Sarcoma	PTEN	p.X342_splice	Shared	10	89720877	T	G	Splice_Site	131	0.167938931	No	1	Clonal	Disease_causing	.	.	likely_pathogenic	No	Yes	Yes	Yes	Not tested
CS3-C	Carcinoma	RB1	p.T726_Y728del	Shared	13	49037934	TAACAGCATA	T	In_Frame_Del	84	0.630952381	No	1	Clonal	.	.	.	.	Yes	Yes	Yes	Yes	Not tested
CS3-S	Sarcoma	RB1	p.T726_Y728del	Shared	13	49037934	TAACAGCATA	T	In_Frame_Del	114	0.640350877	No	1	Clonal	.	.	.	.	Yes	Yes	Yes	Yes	Not tested
CS3-C	Carcinoma	RBBP7	p.A337V	Shared	X	16870879	G	A	Missense_Mutation	571	0.711033275	No	1	Clonal	Disease_causing	Passenger	Neutral	passenger	No	No	No	No	Not tested
CS3-S	Sarcoma	RBBP7	p.A337V	Shared	X	16870879	G	A	Missense_Mutation	650	0.638461538	No	1	Clonal	Disease_causing	Passenger	Neutral	passenger	No	No	No	No	Not tested
CS3-C	Carcinoma	RELN	p.C678F	Shared	7	103281026	C	A	Missense_Mutation	206	0.674757282	No	1	Clonal	Disease_causing	Driver	Neutral	likely_pathogenic	No	No	No	No	Not tested
CS3-S	Sarcoma	RELN	p.C678F	Shared	7	103281026	C	A	Missense_Mutation	322	0.580745342	No	1	Clonal	Disease_causing	Driver	Neutral	likely_pathogenic	No	No	No	No	Not tested
CS3-C	Carcinoma	ROBO2	p.D280H	Shared	3	77542517	G	C	Missense_Mutation	213	0.920187793	No	1	Clonal	Disease_causing	Passenger	Neutral	passenger	Yes	No	No	No	Not tested
CS3-S	Sarcoma	ROBO2	p.D280H	Shared	3	77542517	G	C	Missense_Mutation	351	0.84045584	No	1	Clonal	Disease_causing	Passenger	Neutral	passenger	Yes	No	No	No	Not tested
CS3-C	Carcinoma	SCP2	p.R105G	Shared	1	53416540	C	G	Missense_Mutation	200	0.725	No	1	Clonal	Disease_causing	Passenger	Disease_causing	likely_pathogenic	No	No	No	No	Not tested
CS3-S	Sarcoma	SCP2	p.R105G	Shared	1	53416540	C	G	Missense_Mutation	233	0.721030043	No	1	Clonal	Disease_causing	Passenger	Disease_causing	likely_pathogenic	No	No	No	No	Not tested
CS3-C	Carcinoma	SEMA5A	p.E290K	Shared	5	9202131	C	T	Missense_Mutation	126	0.206349206	No	1	Clonal	Disease_causing	Passenger	Neutral	passenger	No	No	No	No	Not tested
CS3-S	Sarcoma	SEMA5A	p.E290K	Shared	5	9202131	C	T	Missense_Mutation	219	0.155251142	No	1	Clonal	Disease_causing	Passenger	Neutral	passenger	No	No	No	No	Not tested
CS3-S	Sarcoma	SIPA1L2	p.Q1687L	Private	1	232534982	T	A	Missense_Mutation	209	0.04784689	No	0.371	Subclonal	Disease_causing	Passenger	Neutral	passenger	No	No	No	No	Yes
CS3-C	Carcinoma	SLC1A6	p.V365I	Shared	19	15067364	C	T	Missense_Mutation	172	0.453488372	No	1	Clonal	Disease_causing	Passenger	Neutral	passenger	No	No	No	No	Not tested
CS3-S	Sarcoma	SLC1A6	p.V365I	Shared	19	15067364	C	T	Missense_Mutation	225	0.368888889	No	1	Clonal	Disease_causing	Passenger	Neutral	passenger	No	No	No	No	Not tested
CS3-C	Carcinoma	SLC22A24	p.R230Q	Shared	11	62886525	C	T	Missense_Mutation	294	0.571428571	No	1	Clonal	Neutral	Passenger	Neutral	passenger	No	No	No	No	Not tested
CS3-S	Sarcoma	SLC22A24	p.R230Q	Shared	11	62886525	C	T	Missense_Mutation	421	0.574821853	No	1	Clonal	Neutral	Passenger	Neutral	passenger	No	No	No	No	Not tested
CS3-C	Carcinoma	SYNE1	p.K2884*	Shared	6	152706811	T	TA	Frame_Shift_Ins	188	0.45212766	No	1	Clonal	.	.	.	.	No	No	No	No	Not tested
CS3-S	Sarcoma	SYNE1	p.K2884*	Shared	6	152706811	T	TA	Frame_Shift_Ins	217	0.493087558	No	1	Clonal	.	.	.	.	No	No	No	No	Not tested
CS3-C	Carcinoma	TBX1	p.D167N	Shared	22	19750852	G	A	Missense_Mutation	322	0.605590062	No	1	Clonal	Disease_causing	Passenger	Disease_causing	likely_pathogenic	No	No	No	No	Not tested
CS3-S	Sarcoma	TBX1	p.D167N	Shared	22	19750852	G	A	Missense_Mutation	422	0.547393365	No	1	Clonal	Disease_causing	Passenger	Disease_causing	likely_pathogenic	No	No	No	No	Not tested
CS3-C	Carcinoma	TNIK	p.L1007I	Shared	3	170802094	G	T	Missense_Mutation	120	0.908333333	No	1	Clonal	Disease_causing	Passenger	Neutral	passenger	Yes	No	No	No	Not tested
CS3-S	Sarcoma	TNIK	p.L1007I	Shared	3	170802094	G	T	Missense_Mutation	176	0.829545455	No	1	Clonal	Disease_causing	Passenger	Neutral	passenger	Yes	No	No	No	Not tested
CS3-C	Carcinoma	TP53	p.R248Q	Shared	17	7577538	C	T	Missense_Mutation	78	0.858974359	No	1	Clonal	Neutral	Passenger	Disease_causing	likely_pathogenic	Yes	Yes	Yes	Yes	Not tested
CS3-S	Sarcoma	TP53	p.R248Q	Shared	17	7577538	C	T	Missense_Mutation	98	0.806122449	No	1	Clonal	Neutral	Passenger	Disease_causing	likely_pathogenic	Yes	Yes	Yes	Yes	Not tested
CS3-C	Carcinoma	TRMT10A	p.E322Q	Shared	4	100470301	C	G	Missense_Mutation	278	0.226618705	No	1	Clonal	Neutral	Passenger	Neutral	passenger	No	No	No	No	Not tested
CS3-S	Sarcoma	TRMT10A	p.E322Q	Shared	4	100470301	C	G	Missense_Mutation	434	0.177419355	No	1	Clonal	Neutral	Passenger	Neutral	passenger	No	No	No	No	Not tested
CS3-C	Carcinoma	TTC12	p.S446L	Shared	11	113222820	C	T	Missense_Mutation	186	0.279569892	No	1	Clonal	Neutral	Passenger	Neutral	passenger	No	No	No	No	Not tested
CS3-S	Sarcoma	TTC12	p.S446L	Shared	11	113222820	C	T	Missense_Mutation	247	0.287449393	No	1	Clonal	Neutral	Passenger	Neutral	passenger	No	No	No	No	Not tested
CS3-C	Carcinoma	TTC21A	p.E52*	Shared	3	39150702	G	T	Nonsense_Mutation	120	0.941666667	No	1	Clonal	Neutral	.	.	likely_pathogenic	Yes	No	No	No	Not tested
CS3-S	Sarcoma	TTC21A	p.E52*	Shared	3	39150702	G	T	Nonsense_Mutation	177	0.824858757	No	1	Clonal	Neutral	.	.	likely_pathogenic	Yes	No	No	No	Not tested
CS3-C	Carcinoma	WNK1	p.R668Q	Shared	12	971300	G	A	Missense_Mutation	220	0.677272727	No	1	Clonal	Neutral	Driver	Neutral	likely_pathogenic	No	No	No	No	Not tested
CS3-S	Sarcoma	WNK1	p.R668Q	Shared	12	971300	G	A	Missense_Mutation	389	0.565552699	No	1	Clonal	Neutral	Driver	Neutral	likely_pathogenic	No	No	No	No	Not tested
CS3-C	Carcinoma	WNK3	p.I879V	Shared	X	54276505	T	C	Missense_Mutation	137	0.372262774	No	1	Clonal	Neutral	Passenger	Neutral	passenger	No	No	No	No	Not tested
CS3-S	Sarcoma	WNK3	p.I879V	Shared	X	54276505	T	C	Missense_Mutation	154	0.220779221	No	1	Clonal	Neutral	Passenger	Neutral	passenger	No	No	No	No	Not tested
CS3-C	Carcinoma	WWP1	p.C629R	Shared	8	87454894	T	C	Missense_Mutation	171	0.157894737	No	1	Clonal	Disease_causing	Driver	Neutral	likely_pathogenic	Yes	No	No	No	Not tested
CS3-S	Sarcoma	WWP1	p.C629R	Shared	8	87454894	T	C	Missense_Mutation	273	0.10989011	No	0.961	Clonal	Disease_causing	Driver	Neutral	likely_pathogenic	Yes	No	No	No	Not tested
CS3-C	Carcinoma	ZNF444	p.R42C	Shared	19	56658404	C	T	Missense_Mutation	51	0.568627451	No	1	Clonal	Disease_causing	Passenger	Neutral	passenger	No	No	No	No	Not tested
CS3-S	Sarcoma	ZNF444	p.R42C	Shared	19	56658404	C	T	Missense_Mutation	53	0.509433962	No	1	Clonal	Disease_causing	Passenger	Neutral	passenger	No	No	No	No	Not tested
CS3-C	Carcinoma	ZNF492	p.A40T	Shared	19	22836805	G	A	Missense_Mutation	157	0.082802548	No	0.524	Subclonal	Neutral	Passenger	Neutral	passenger	No	No	No	No	Not tested
CS3-S	Sarcoma	ZNF492	p.A40T	Shared	19	22836805	G	A	Missense_Mutation	162	0.117283951	No	0.791	Subclonal	Neutral	Passenger	Neutral	passenger	No	No	No	No	Not tested
CS3-S	Sarcoma	ZNF561	p.D213H	Private	19	9721700	C	G	Missense_Mutation	84	0.05952381	No	0.402	Subclonal	Neutral	Passenger	Neutral	passenger	No	No	No	No	Not tested
CS3-C	Carcinoma	ZNF839	p.T663Rfs*22	Shared	14	102807717	CG	C	Frame_Shift_Del	454	0.356828194	No	1	Clonal	.	.	.	.	No	No	No	No	Not tested
CS3-S	Sarcoma	ZNF839	p.T663Rfs*22	Shared	14	102807717	CG	C	Frame_Shift_Del	570	0.324561404	No	1	Clonal	.	.	.	.	No	No	No	No	Not tested
CS3-C	Carcinoma	ZSWIM2	p.I242V	Shared	2	187702052	T	C	Missense_Mutation	180	0.705555556	No	1	Clonal	Disease_causing	Passenger	Disease_causing	likely_pathogenic	Yes	No	No	No	Not tested
CS3-S	Sarcoma	ZSWIM2	p.I242V	Shared	2	187702052	T	C	Missense_Mutation	284	0.661971831	No	1	Clonal	Disease_causing	Passenger	Disease_causing	likely_pathogenic	Yes	No	No	No	Not tested
CS4-C	Carcinoma	ABCA13	p.W683L	Shared	7	48308619	G	T	Missense_Mutation	131	0.404580153	No	1	Clonal	Disease_causing	Passenger	Disease_causing	likely_pathogenic	No	No	No	No	Not tested
CS4-S	Sarcoma	ABCA13	p.W683L	Shared	7	48308619	G	T	Missense_Mutation	140	0.4	No	1	Clonal	Disease_causing	Passenger	Disease_causing	likely_pathogenic	No	No	No	No	Not tested
CS4-C	Carcinoma	AEBP1	p.P281L	Shared	7	44147510	C	T	Missense_Mutation	183	0.344262295	No	1	Clonal	Neutral	Passenger	Disease_causing	passenger	No	No	No	No	Not tested
CS4-S	Sarcoma	AEBP1	p.P281L	Shared	7	44147510	C	T	Missense_Mutation	199	0.286432161	No	1	Clonal	Neutral	Passenger	Disease_causing	passenger	No	No	No	No	Not tested
CS4-C	Carcinoma	ANKRD31	p.E1541K	Private	5	74400763	C	T	Missense_Mutation	613	0.295269168	No	1	Clonal	Neutral	Passenger	Neutral	passenger	No	No	No	No	Not tested
CS4-S	Sarcoma	ARC	p.A316G	Private	8	143694686	G	C	Missense_Mutation	374	0.090909091	No	0.774	Subclonal	Disease_causing	Passenger	Neutral	passenger	No	No	No	No	Yes
CS4-C	Carcinoma	BCCIP	p.P20S	Private	10	127512184	C	T	Missense_Mutation	282	0.035460993	No	0.145	Subclonal	Neutral	Passenger	Neutral	passenger	No	No	No	No	Yes
CS4-C	Carcinoma	BZRAP1	p.S1417Kfs*40	Private	17	56386383	C	CT	Frame_Shift_Ins	549	0.183970856	No	0.75	Subclonal	.	.	.	.	No	No	No	No	Yes
CS4-C	Carcinoma	C12orf56	p.D57N	Shared	12	64784177	C	T	Missense_Mutation	413	0.617433414	No	1	Clonal	Disease_causing	Passenger	.	passenger	No	No	No	No	Not tested
CS4-S	Sarcoma	C12orf56	p.D57N	Shared	12	64784177	C	T	Missense_Mutation	267	0.415730337	No	1	Clonal	Disease_causing	Passenger	.	passenger	No	No	No	No	Not tested
CS4-C	Carcinoma	CD97	p.E307K	Shared	19	14512219	G	A	Missense_Mutation	227	0.330396476	No	1	Clonal	Neutral	Passenger	Neutral	passenger	No	No	No	No	Not tested
CS4-S	Sarcoma	CD97	p.E307K	Shared	19	14512219	G	A	Missense_Mutation	176	0.25	No	1	Clonal	Neutral	Passenger	Neutral	passenger	No	No	No	No	Not tested
CS4-C	Carcinoma	CIRH1A	p.F54V	Shared	16	69170599	T	G	Missense_Mutation	139	0.942446043	No	1	Clonal	Disease_causing	Passenger	Neutral	passenger	Yes	No	No	No	Not tested
CS4-S	Sarcoma	CIRH1A	p.F54V	Shared	16	69170599	T	G	Missense_Mutation	130	0.576923077	No	1	Clonal	Disease_causing	Passenger	Neutral	passenger	Yes	No	No	No	Not tested
CS4-C	Carcinoma	CLTC	p.R895H	Private	17	57754437	G	A	Missense_Mutation	317	0.268138801	No	1	Clonal	Disease_causing	Passenger	Neutral	passenger	No	Yes	No	No	Yes
CS4-C	Carcinoma	COL7A1	p.R843*	Shared	3	48626135	G	A	Nonsense_Mutation	486	0.308641975	No	1	Clonal	Neutral	.	.	passenger	Yes	No	No	No	Not tested
CS4-S	Sarcoma	COL7A1	p.R843*	Shared	3	48626135	G	A	Nonsense_Mutation	352	0.264204545	No	1	Clonal	Neutral	.	.	passenger	Yes	No	No	No	Not tested
CS4-S	Sarcoma	CPXM2	p.C136R	Private	10	125622237	A	G	Missense_Mutation	393	0.099236641	No	0.845	Subclonal	Disease_causing	Passenger	Disease_causing	likely_pathogenic	No	No	No	No	Yes
CS4-C	Carcinoma	CRHBP	p.T17M	Shared	5	76249009	C	T	Missense_Mutation	231	0.329004329	No	1	Clonal	Neutral	Passenger	.	passenger	No	No	No	No	Not tested
CS4-S	Sarcoma	CRHBP	p.T17M	Shared	5	76249009	C	T	Missense_Mutation	180	0.238888889	No	1	Clonal	Neutral	Passenger	.	passenger	No	No	No	No	Not tested
CS4-S	Sarcoma	CYLC1	p.K479E	Private	X	83129151	A	G	Missense_Mutation	322	0.031055901	No	0.202	Subclonal	Neutral	Passenger	Neutral	passenger	No	No	No	No	Yes
CS4-C	Carcinoma	CYP39A1	p.E78*	Shared	6	46609981	C	A	Nonsense_Mutation	248	0.241935484	No	0.986	Clonal	Neutral	.	.	passenger	No	No	No	No	Not tested
CS4-S	Sarcoma	CYP39A1	p.E78*	Shared	6	46609981	C	A	Nonsense_Mutation	212	0.372641509	No	1	Clonal	Neutral	.	.	passenger	No	No	No	No	Not tested
CS4-C	Carcinoma	DISP1	p.E1257Q	Shared	1	223178508	G	C	Missense_Mutation	504	0.466269841	No	1	Clonal	Neutral	Passenger	Disease_causing	passenger	No	No	No	No	Not tested
CS4-S	Sarcoma	DISP1	p.E1257Q	Shared	1	223178508	G	C	Missense_Mutation	599	0.238731219	No	1	Clonal	Neutral	Passenger	Disease_causing	passenger	No	No	No	No	Not tested
CS4-C	Carcinoma	DNAH2	p.R2768C	Private	17	7701546	C	T	Missense_Mutation	347	0.403458213	No	1	Clonal	Disease_causing	Passenger	Neutral	passenger	Yes	No	No	No	Yes
CS4-S	Sarcoma	EVI2A	p.V123I	Private	17	29645734	C	T	Missense_Mutation	507	0.078895464	No	0.75	Subclonal	Neutral	Passenger	.	passenger	No	Yes	Yes	Yes	Yes
CS4-C	Carcinoma	FBXW7	p.R465C	Shared	4	153249385	G	A	Missense_Mutation	459	0.276688453	Yes	1	Clonal	Disease_causing	Driver	Neutral	likely_pathogenic	No	Yes	Yes	Yes	Not tested
CS4-S	Sarcoma	FBXW7	p.R465C	Shared	4	153249385	G	A	Missense_Mutation	366	0.068306011	Yes	0.445	Clonal	Disease_causing	Driver	Neutral	likely_pathogenic	Yes	Yes	Yes	Yes	Not tested
CS4-C	Carcinoma	FIBCD1	p.R287H	Private	9	133787265	C	T	Missense_Mutation	219	0.324200913	No	1	Clonal	Disease_causing	Passenger	Neutral	passenger	No	No	No	No	Yes
CS4-C	Carcinoma	FPR2	p.V303I	Shared	19	52272818	G	A	Missense_Mutation	473	0.507399577	No	1	Clonal	Disease_causing	Passenger	Neutral	passenger	No	No	No	No	Not tested
CS4-S	Sarcoma	FPR2	p.V303I	Shared	19	52272818	G	A	Missense_Mutation	449	0.285077951	No	1	Clonal	Disease_causing	Passenger	Neutral	passenger	No	No	No	No	Not tested
CS4-C	Carcinoma	GATAD2A	p.D39H	Shared	19	19576269	G	C	Missense_Mutation	554	0.301444043	No	1	Clonal	Disease_causing	Passenger	Neutral	passenger	No	No	No	No	Not tested
CS4-S	Sarcoma	GATAD2A	p.D39H	Shared	19	19576269	G	C	Missense_Mutation	452	0.221238938	No	1	Clonal	Disease_causing	Passenger	Neutral	passenger	No	No	No	No	Not tested
CS4-C	Carcinoma	GLUD2	p.R231Q	Shared	X	120182230	G	A	Missense_Mutation	271	0.41697417	No	1	Clonal	Disease_causing	Passenger	Disease_causing	likely_pathogenic	No	No	No	No	Not tested
CS4-S	Sarcoma	GLUD2	p.R231Q	Shared	X	120182230	G	A	Missense_Mutation	242	0.231404959	No	1	Clonal	Disease_causing	Passenger	Disease_causing	likely_pathogenic	No	No	No	No	Not tested
CS4-C	Carcinoma	GNL2	p.W348S	Shared	1	38041296	C	G	Missense_Mutation	341	0.240469208	No	0.98	Clonal	Disease_causing	Passenger	Neutral	passenger	No	No	No	No	Not tested
CS4-S	Sarcoma	GNL2	p.W348S	Shared	1	38041296	C	G	Missense_Mutation	370	0.178378378	No	1	Clonal	Disease_causing	Passenger	Neutral	passenger	No	No	No	No	Not tested
CS4-S	Sarcoma	GOLGB1	p.K1662N	Private	3	121414384	C	G	Missense_Mutation	413	0.05811138	No	0.495	Subclonal	Disease_causing	Passenger	Neutral	passenger	No	No	No	No	Yes
CS4-S	Sarcoma	GPR98	p.I4787V	Private	5	90087005	A	G	Missense_Mutation	314	0.060509554	No	0.455	Subclonal	Neutral	Passenger	Neutral	passenger	No	No	No	No	Yes
CS4-C	Carcinoma	HCN1	p.R781W	Shared	5	45262355	G	A	Missense_Mutation	393	0.315521628	No	1	Clonal	Disease_causing	Passenger	Neutral	passenger	No	No	No	No	Not tested
CS4-S	Sarcoma	HCN1	p.R781W	Shared	5	45262355	G	A	Missense_Mutation	326	0.205521472	No	1	Clonal	Disease_causing	Passenger	Neutral	passenger	No	No	No	No	Not tested
CS4-C	Carcinoma	HELQ	p.L313R	Shared	4	84374458	A	C	Missense_Mutation	353	0.280453258	No	1	Clonal	Neutral	Passenger	Neutral	passenger	No	No	No	No	Not tested
CS4-S	Sarcoma	HELQ	p.L313R	Shared	4	84374458	A	C	Missense_Mutation	249	0.208835341	No	1	Clonal	Neutral	Passenger	Neutral	passenger	No	No	No	No	Not tested
CS4-C	Carcinoma	HTR1A	p.C187Y	Shared	5	63256987	C	T	Missense_Mutation	612	0.307189542	No	1	Clonal	Disease_causing	Driver	Neutral	likely_pathogenic	No	No	No	No	Not tested
CS4-S	Sarcoma	HTR1A	p.C187Y	Shared	5	63256987	C	T	Missense_Mutation	501	0.20758483	No	1	Clonal	Disease_causing	Driver	Neutral	likely_pathogenic	No	No	No	No	Not tested
CS4-S	Sarcoma	HYAL4	p.I75T	Private	7	123508551	T	C	Missense_Mutation	260	0.096153846	No	0.722	Subclonal	Neutral	Passenger	Neutral	passenger	No	No	No	No	Yes
CS4-S	Sarcoma	IGDCC4	p.T842P	Private	15	65681634	T	G	Missense_Mutation	103	0.058252427	No	0.438	Subclonal	Disease_causing	Passenger	Neutral	passenger	No	No	No	No	Not tested
CS4-C	Carcinoma	IGSF1	p.R342G	Shared	X	130416640	G	C	Missense_Mutation	100	0.45	No	1	Clonal	Neutral	Passenger	Neutral	passenger	No	No	No	No	Not tested
CS4-S	Sarcoma	IGSF1	p.R342G	Shared	X	130416640	G	C	Missense_Mutation	82	0.219512195	No	1	Clonal	Neutral	Passenger	Neutral	passenger	No	No	No	No	Not tested
CS4-C	Carcinoma	IL6R	p.A282T	Shared	1	154408481	G	A	Missense_Mutation	413	0.246973366	No	1	Clonal	Disease_causing	Passenger	Neutral	passenger	No	No	No	No	Not tested
CS4-S	Sarcoma	IL6R	p.A282T	Shared	1	154408481	G	A	Missense_Mutation	293	0.197952218	No	1	Clonal	Disease_causing	Passenger	Neutral	passenger	No	No	No	No	Not tested
CS4-C	Carcinoma	KIAA0825	p.A554G	Shared	5	93807231	G	C	Missense_Mutation	261	0.356321839	No	1	Clonal	Disease_causing	Passenger	Neutral	passenger	No	No	No	No	Not tested
CS4-S	Sarcoma	KIAA0825	p.A554G	Shared	5	93807231	G	C	Missense_Mutation	382	0.204188482	No	1	Clonal	Disease_causing	Passenger	Neutral	passenger	No	No	No	No	Not tested
CS4-C	Carcinoma	KIAA1551	p.P505Q	Private	12	32135403	C	A	Missense_Mutation	655	0.116030534	No	0.589	Subclonal	Neutral	Passenger	Neutral	passenger	No	No	No	No	Not tested
CS4-C	Carcinoma	LPXN	p.M349I	Shared	11	58295056	C	G	Missense_Mutation	389	0.444730077	No	1	Clonal	Disease_causing	Passenger	Disease_causing	likely_pathogenic	No	No	No	No	Not tested
CS4-S	Sarcoma	LPXN	p.M349I	Shared	11	58295056	C	G	Missense_Mutation	356	0.370786517	No	1	Clonal	Disease_causing	Passenger	Disease_causing	likely_pathogenic	No	No	No	No	Not tested
CS4-C	Carcinoma	MACF1	p.E515V	Private	1	39752978	A	T	Missense_Mutation	176	0.164772727	No	0.672	Subclonal	Disease_causing	Passenger	Disease_causing	likely_pathogenic	No	No	No	No	Yes
CS4-S	Sarcoma	MBIP	p.E132Q	Private	14	36783977	C	G	Missense_Mutation	596	0.045302013	No	0.34	Subclonal	Neutral	Passenger	Neutral	passenger	No	No	No	No	Yes
CS4-C	Carcinoma	METTL7B	p.H116D	Shared	12	56075884	C	G	Missense_Mutation	786	0.426208651	No	1	Clonal	Disease_causing	Passenger	Neutral	passenger	No	No	No	No	Not tested
CS4-S	Sarcoma	METTL7B	p.H116D	Shared	12	56075884	C	G	Missense_Mutation	555	0.295495495	No	1	Clonal	Disease_causing	Passenger	Neutral	passenger	No	No	No	No	Not tested
CS4-S	Sarcoma	MRAP2	p.M87R	Private	6	84798842	T	G	Missense_Mutation	235	0.068085106	No	0.58	Subclonal	Disease_causing	Passenger	Disease_causing	likely_pathogenic	No	No	No	No	Not tested
CS4-C	Carcinoma	MTTP	p.A577P	Private	4	100530094	G	C	Missense_Mutation	286	0.038461538	No	0.157	Subclonal	Neutral	Passenger	Neutral	passenger	No	No	No	No	Yes
CS4-C	Carcinoma	MXRA5	p.A2015V	Shared	X	3235678	G	A	Missense_Mutation	622	0.274919614	No	1	Clonal	Neutral	Passenger	Neutral	passenger	No	No	No	Yes	Not tested
CS4-S	Sarcoma	MXRA5	p.A2015V	Shared	X	3235678	G	A	Missense_Mutation	521	0.280230326	No	1	Clonal	Neutral	Passenger	Neutral	passenger	No	No	No	Yes	Not tested
CS4-S	Sarcoma	NLRP10	p.R492C	Private	11	7981685	G	A	Missense_Mutation	362	0.046961326	No	0.353	Subclonal	Neutral	Passenger	Neutral	passenger	No	No	No	No	Not tested
CS4-C	Carcinoma	NRXN1	p.T777M	Shared	2	50758502	G	A	Missense_Mutation	149	0.080536913	No	0.248	Subclonal	Disease_causing	Passenger	Neutral	passenger	Yes	No	No	No	Not tested
CS4-S	Sarcoma	NRXN1	p.T777M	Shared	2	50758502	G	A	Missense_Mutation	157	0.197452229	No	1	Clonal	Disease_causing	Passenger	Neutral	passenger	Yes	No	No	No	Not tested
CS4-S	Sarcoma	NUP107	p.N761T	Private	12	69128507	A	C	Missense_Mutation	285	0.063157895	No	0.474	Subclonal	Neutral	Passenger	.	passenger	No	No	No	No	Yes
CS4-C	Carcinoma	NUSAP1	p.R272H	Shared	15	41657754	G	A	Missense_Mutation	273	0.421245421	No	1	Clonal	Neutral	Passenger	Neutral	passenger	Yes	No	No	No	Not tested
CS4-S	Sarcoma	NUSAP1	p.R272H	Shared	15	41657754	G	A	Missense_Mutation	269	0.178438662	No	1	Clonal	Neutral	Passenger	Neutral	passenger	No	No	No	No	Not tested
CS4-C	Carcinoma	OR6B1	p.S188A	Private	7	143701651	T	G	Missense_Mutation	323	0.250773994	No	0.772	Clonal	Neutral	Passenger	Neutral	passenger	No	No	No	No	Yes
CS4-C	Carcinoma	OR8I2	p.C178Vfs*7	Shared	11	55861307	AT	A	Frame_Shift_Del	368	0.413043478	No	1	Clonal	.	.	.	.	No	No	No	No	Not tested
CS4-S	Sarcoma	OR8I2	p.C178Vfs*7	Shared	11	55861307	AT	A	Frame_Shift_Del	383	0.104438642	No	0.785	Clonal	.	.	.	.	No	No	No	No	Not tested
CS4-C	Carcinoma	PDZD4	p.A339V	Shared	X	153070102	G	A	Missense_Mutation	313	0.498402556	No	1	Clonal	Neutral	Passenger	Neutral	passenger	No	No	No	No	Not tested
CS4-S	Sarcoma	PDZD4	p.A339V	Shared	X	153070102	G	A	Missense_Mutation	282	0.287234043	No	1	Clonal	Neutral	Passenger	Neutral	passenger	No	No	No	No	Not tested
CS4-C	Carcinoma	PIK3CA	p.E542V	Shared	3	178936083	A	T	Missense_Mutation	363	0.953168044	Yes	1	Clonal	Disease_causing	Driver	Neutral	likely_pathogenic	Yes	Yes	Yes	Yes	Not tested
CS4-S	Sarcoma	PIK3CA	p.E542V	Shared	3	178936083	A	T	Missense_Mutation	310	0.567741935	Yes	1	Clonal	Disease_causing	Driver	Neutral	likely_pathogenic	Yes	Yes	Yes	Yes	Not tested
CS4-S	Sarcoma	PRAME	p.R263H	Private	22	22892313	C	T	Missense_Mutation	366	0.057377049	No	0.374	Subclonal	Neutral	Passenger	Neutral	passenger	No	No	No	No	Not tested
CS4-C	Carcinoma	PTK7	p.E751D	Private	6	43111336	G	T	Missense_Mutation	90	0.066666667	No	0.272	Subclonal	Disease_causing	Driver	Neutral	likely_pathogenic	No	No	No	No	Not tested
CS4-S	Sarcoma	RAB11FIP3	p.Y245S	Private	16	511423	A	C	Missense_Mutation	362	0.107734807	No	1	Clonal	Disease_causing	Passenger	Neutral	passenger	Yes	No	No	No	Not tested
CS4-S	Sarcoma	RAP1GDS1	p.L363R	Private	4	99339853	T	G	Missense_Mutation	165	0.066666667	No	0.434	Subclonal	Disease_causing	Driver	Disease_causing	likely_pathogenic	Yes	Yes	No	No	Not tested
CS4-S	Sarcoma	RCCD1	p.R161C	Private	15	91500657	C	T	Missense_Mutation	150	0.513333333	No	1	Clonal	Disease_causing	Passenger	Neutral	passenger	No	No	No	No	Not tested
CS4-C	Carcinoma	RCOR2	p.R230W	Shared	11	63681629	G	A	Missense_Mutation	258	0.449612403	No	1	Clonal	Neutral	Passenger	Neutral	passenger	No	No	No	No	Not tested
CS4-S	Sarcoma	RCOR2	p.R230W	Shared	11	63681629	G	A	Missense_Mutation	185	0.162162162	No	1	Clonal	Neutral	Passenger	Neutral	passenger	No	No	No	No	Not tested
CS4-S	Sarcoma	RTBDN	p.P114A	Private	19	12939693	G	C	Missense_Mutation	296	0.060810811	No	0.396	Subclonal	Neutral	Passenger	Neutral	passenger	Yes	No	No	No	Yes
CS4-C	Carcinoma	SCN10A	p.F1197S	Shared	3	38760235	A	G	Missense_Mutation	403	0.330024814	No	1	Clonal	Disease_causing	Passenger	Disease_causing	likely_pathogenic	Yes	No	No	No	Not tested
CS4-S	Sarcoma	SCN10A	p.F1197S	Shared	3	38760235	A	G	Missense_Mutation	302	0.245033113	No	1	Clonal	Disease_causing	Passenger	Disease_causing	likely_pathogenic	Yes	No	No	No	Not tested
CS4-S	Sarcoma	SCN2A	p.Y1588*	Private	2	166243468	C	G	Nonsense_Mutation	378	0.071428571	No	0.608	Subclonal	Neutral	.	.	passenger	Yes	No	No	No	Yes
CS4-C	Carcinoma	SMTN	p.T510I	Shared	22	31487730	C	T	Missense_Mutation	209	0.435406699	No	1	Clonal	Disease_causing	Passenger	Neutral	passenger	No	No	No	No	Not tested
CS4-S	Sarcoma	SMTN	p.T510I	Shared	22	31487730	C	T	Missense_Mutation	174	0.270114943	No	1	Clonal	Disease_causing	Passenger	Neutral	passenger	No	No	No	No	Not tested
CS4-C	Carcinoma	SPRED2	p.H191L	Shared	2	65543884	T	A	Missense_Mutation	123	0.357723577	No	1	Clonal	Disease_causing	Passenger	Neutral	passenger	Yes	No	No	No	Not tested
CS4-S	Sarcoma	SPRED2	p.H191L	Shared	2	65543884	T	A	Missense_Mutation	89	0.258426966	No	1	Clonal	Disease_causing	Passenger	Neutral	passenger	Yes	No	No	No	Not tested
CS4-C	Carcinoma	TFAP2D	p.R142H	Shared	6	50683214	G	A	Missense_Mutation	504	0.287698413	No	1	Clonal	Disease_causing	Passenger	Disease_causing	likely_pathogenic	No	No	No	No	Not tested
CS4-S	Sarcoma	TFAP2D	p.R142H	Shared	6	50683214	G	A	Missense_Mutation	466	0.169527897	No	1	Clonal	Disease_causing	Passenger	Disease_causing	likely_pathogenic	No	No	No	No	Not tested
CS4-C	Carcinoma	TNNI3	p.R192C	Shared	19	55663261	G	A	Missense_Mutation	226	0.442477876	No	1	Clonal	Disease_causing	Passenger	Disease_causing	likely_pathogenic	No	No	No	No	Not tested
CS4-S	Sarcoma	TNNI3	p.R192C	Shared	19	55663261	G	A	Missense_Mutation	198	0.242424242	No	1	Clonal	Disease_causing	Passenger	Disease_causing	likely_pathogenic	No	No	No	No	Not tested
CS4-C	Carcinoma	TP53	p.K132R	Shared	17	7578535	T	C	Missense_Mutation	232	0.935344828	Yes	1	Clonal	Disease_causing	Passenger	Disease_causing	likely_pathogenic	Yes	Yes	Yes	Yes	Not tested
CS4-S	Sarcoma	TP53	p.K132R	Shared	17	7578535	T	C	Missense_Mutation	173	0.676300578	Yes	1	Clonal	Disease_causing	Passenger	Disease_causing	likely_pathogenic	Yes	Yes	Yes	Yes	Not tested
CS4-S	Sarcoma	TRAF1	p.A263V	Private	9	123673709	G	A	Missense_Mutation	140	0.114285714	No	0.744	Subclonal	Disease_causing	Passenger	Neutral	passenger	Yes	No	No	No	Yes
CS4-S	Sarcoma	TRPV3	p.A218E	Private	17	3438998	G	T	Missense_Mutation	151	0.066225166	No	0.564	Subclonal	Disease_causing	Passenger	Neutral	passenger	Yes	No	No	No	Not tested
CS4-C	Carcinoma	UBL4A	p.E146K	Shared	X	153713916	C	T	Missense_Mutation	269	0.405204461	No	1	Clonal	Neutral	Passenger	Neutral	passenger	No	No	No	No	Not tested
CS4-S	Sarcoma	UBL4A	p.E146K	Shared	X	153713916	C	T	Missense_Mutation	245	0.26122449	No	1	Clonal	Neutral	Passenger	Neutral	passenger	No	No	No	No	Not tested
CS4-C	Carcinoma	VSIG4	p.P171L	Shared	X	65252492	G	A	Missense_Mutation	457	0.291028446	No	1	Clonal	Disease_causing	Passenger	Neutral	passenger	No	No	No	No	Not tested

CS4-S	Sarcoma	VSIG4	p.P171L	Shared	X	65252492	G	A	Missense_Mutation	[illegible]	[illegible]	No	1	Clonal	Disease_causing	Passenger	Neutral	passenger	No	No	No	No	Not tested
CS4-C	Carcinoma	YIPF7	p.C242G	Shared	4	44624550	A	C	Missense_Mutation	[illegible]	[illegible]	No	0.577	Subclonal	Disease_causing	Passenger	Neutral	passenger	Yes	No	No	No	Not tested
CS4-S	Sarcoma	YIPF7	p.C242G	Shared	4	44624550	A	C	Missense_Mutation	54	0.12962963	No	0.844	Clonal	Disease_causing	Passenger	Neutral	passenger	No	No	No	No	Not tested
CS4-S	Sarcoma	ZBTB8B	p.T349A	Private	1	32946545	A	G	Missense_Mutation	371	0.075471698	No	0.567	Subclonal	Disease_causing	Passenger	Neutral	passenger	No	No	No	No	Yes
CS4-C	Carcinoma	ZFP62	p.N19S	Shared	5	180278439	T	C	Missense_Mutation	348	0.451149425	No	1	Clonal	Disease_causing	Passenger	Neutral	passenger	No	No	No	No	Not tested
CS4-S	Sarcoma	ZFP62	p.N19S	Shared	5	180278439	T	C	Missense_Mutation	280	0.3	No	1	Clonal	Disease_causing	Passenger	Neutral	passenger	No	No	No	No	Not tested
CS4-S	Sarcoma	ZNF425	p.R424C	Private	7	148801693	G	A	Missense_Mutation	204	0.12745098	No	0.957	Clonal	Neutral	Passenger	Neutral	passenger	No	No	No	No	Yes
CS4-C	Carcinoma	ZNFX1	p.F373C	Shared	20	47887231	A	C	Missense_Mutation	1358	0.106774669	No	0.756	Subclonal	Disease_causing	Driver	Disease_causing	likely_pathogenic	No	No	No	No	Not tested
CS4-S	Sarcoma	ZNFX1	p.F373C	Shared	20	47887231	A	C	Missense_Mutation	745	0.077852349	No	0.812	Clonal	Disease_causing	Driver	Disease_causing	likely_pathogenic	No	No	No	No	Not tested
CS5-S	Sarcoma	ACAN	p.T1441A	Private	15	89400137	A	G	Missense_Mutation	108	0.055555556	No	0.207	Subclonal	Neutral	Passenger	Disease_causing	passenger	No	No	No	No	Not tested
CS5-C	Carcinoma	ACVR1B	p.N381I	Shared	12	52379015	A	T	Missense_Mutation	125	0.92	No	1	Clonal	Disease_causing	Passenger	Disease_causing	likely_pathogenic	Yes	No	Yes	Yes	Not tested
CS5-S	Sarcoma	ACVR1B	p.N381I	Shared	12	52379015	A	T	Missense_Mutation	133	0.729323308	No	1	Clonal	Disease_causing	Passenger	Disease_causing	likely_pathogenic	Yes	No	Yes	Yes	Not tested
CS5-C	Carcinoma	ADAM9	p.F452L	Private	8	38912053	T	A	Missense_Mutation	347	0.04610951	No	0.234	Subclonal	Disease_causing	Passenger	Neutral	passenger	No	No	No	No	Yes
CS5-C	Carcinoma	ADAMTS14	p.D297N	Private	10	72489068	G	A	Missense_Mutation	236	0.18220339	No	0.743	Subclonal	Disease_causing	Passenger	Disease_causing	likely_pathogenic	No	No	No	No	Not tested
CS5-C	Carcinoma	ANK2	p.I1209F	Shared	4	114257766	A	T	Missense_Mutation	128	0.7890625	No	1	Clonal	Disease_causing	Passenger	Neutral	passenger	Yes	No	No	No	Not tested
CS5-S	Sarcoma	ANK2	p.I1209F	Shared	4	114257766	A	T	Missense_Mutation	132	0.628787879	No	1	Clonal	Disease_causing	Passenger	Neutral	passenger	Yes	No	No	No	Not tested
CS5-C	Carcinoma	ATP13A2	p.G1019R	Shared	1	17313569	C	G	Missense_Mutation	154	0.305194805	No	0.939	Clonal	Disease_causing	Passenger	Disease_causing	likely_pathogenic	Yes	No	No	No	Not tested
CS5-S	Sarcoma	ATP13A2	p.G1019R	Shared	1	17313569	C	G	Missense_Mutation	129	0.310077519	No	1	Clonal	Disease_causing	Passenger	Disease_causing	likely_pathogenic	Yes	No	No	No	Not tested
CS5-C	Carcinoma	C10orf126	p.G115C	Shared	10	29169199	G	T	Missense_Mutation	329	0.671732523	No	1	Clonal	.	Passenger	.	passenger	No	No	No	No	Not tested
CS5-S	Sarcoma	C10orf126	p.G115C	Shared	10	29169199	G	T	Missense_Mutation	292	0.558219178	No	1	Clonal	.	Passenger	.	passenger	No	No	No	No	Not tested
CS5-C	Carcinoma	C3orf55	p.P127L	Shared	3	157318059	C	T	Missense_Mutation	528	0.227272727	No	1	Clonal	Neutral	Passenger	Neutral	passenger	Yes	No	No	No	Not tested
CS5-S	Sarcoma	C3orf55	p.P127L	Shared	3	157318059	C	T	Missense_Mutation	457	0.24726477	No	1	Clonal	Neutral	Passenger	Neutral	passenger	Yes	No	No	No	Not tested
CS5-C	Carcinoma	C3orf67	p.D397N	Shared	3	58849313	C	T	Missense_Mutation	279	0.265232975	No	0.816	Clonal	Disease_causing	Passenger	Neutral	passenger	Yes	No	No	No	Not tested
CS5-C	Carcinoma	C3orf67	p.D304N	Shared	3	58849592	C	T	Missense_Mutation	177	0.197740113	No	0.608	Subclonal	Neutral	Passenger	Neutral	passenger	Yes	No	No	No	Not tested
CS5-S	Sarcoma	C3orf67	p.D397N	Shared	3	58849313	C	T	Missense_Mutation	247	0.060728745	No	0.227	Clonal	Disease_causing	Passenger	Neutral	passenger	Yes	No	No	No	Not tested
CS5-S	Sarcoma	C3orf67	p.D304N	Shared	3	58849592	C	T	Missense_Mutation	149	0.060402685	No	0.226	Subclonal	Neutral	Passenger	Neutral	passenger	Yes	No	No	No	Not tested
CS5-C	Carcinoma	CACNB2	p.L446F	Shared	10	18827142	C	T	Missense_Mutation	273	0.307692308	No	1	Clonal	Disease_causing	Passenger	Neutral	passenger	No	No	No	No	Not tested
CS5-S	Sarcoma	CACNB2	p.L446F	Shared	10	18827142	C	T	Missense_Mutation	249	0.265060241	No	1	Clonal	Disease_causing	Passenger	Neutral	passenger	No	No	No	No	Not tested
CS5-C	Carcinoma	CACNB4	p.X207_splice	Shared	2	152727126	C	A	Splice_Site	441	0.58276644	No	1	Clonal	Disease_causing	.	.	passenger	No	No	No	No	Not tested
CS5-S	Sarcoma	CACNB4	p.X207_splice	Shared	2	152727126	C	A	Splice_Site	370	0.551351351	No	1	Clonal	Disease_causing	.	.	passenger	No	No	No	No	Not tested
CS5-C	Carcinoma	CD300LF	p.G34R	Private	17	72700899	C	G	Missense_Mutation	588	0.175170068	No	0.714	Subclonal	Neutral	Passenger	Neutral	passenger	Yes	No	No	No	Not tested
CS5-C	Carcinoma	CHD4	p.R887W	Shared	12	6701977	G	A	Missense_Mutation	229	0.96069869	No	1	Clonal	Disease_causing	Passenger	Disease_causing	likely_pathogenic	Yes	Yes	No	Yes	Not tested
CS5-S	Sarcoma	CHD4	p.R887W	Shared	12	6701977	G	A	Missense_Mutation	216	0.782407407	No	1	Clonal	Disease_causing	Passenger	Disease_causing	likely_pathogenic	Yes	Yes	No	Yes	Not tested
CS5-C	Carcinoma	CHST12	p.A31T	Shared	7	2472365	G	A	Missense_Mutation	577	0.49220104	No	1	Clonal	Neutral	Passenger	Neutral	passenger	No	No	No	No	Not tested
CS5-S	Sarcoma	CHST12	p.A31T	Shared	7	2472365	G	A	Missense_Mutation	462	0.430735931	No	1	Clonal	Neutral	Passenger	Neutral	passenger	No	No	No	No	Not tested
CS5-C	Carcinoma	CPED1	p.N996K	Shared	7	120935613	T	A	Missense_Mutation	575	0.467826087	No	1	Clonal	Neutral	Passenger	Neutral	passenger	No	No	No	No	Not tested
CS5-S	Sarcoma	CPED1	p.N996K	Shared	7	120935613	T	A	Missense_Mutation	417	0.426858513	No	1	Clonal	Neutral	Passenger	Neutral	passenger	No	No	No	No	Not tested
CS5-C	Carcinoma	DNAH11	p.P2205L	Shared	7	21747363	C	T	Missense_Mutation	566	0.243816254	No	1	Clonal	Disease_causing	Passenger	Neutral	passenger	No	No	No	No	Not tested
CS5-S	Sarcoma	DNAH11	p.P2205L	Shared	7	21747363	C	T	Missense_Mutation	456	0.225877193	No	1	Clonal	Disease_causing	Passenger	Neutral	passenger	No	No	No	No	Not tested
CS5-C	Carcinoma	EFNB3	p.S141L	Shared	17	7611759	C	T	Missense_Mutation	368	0.934782609	No	1	Clonal	Disease_causing	Passenger	Disease_causing	likely_pathogenic	Yes	No	No	No	Not tested
CS5-S	Sarcoma	EFNB3	p.S141L	Shared	17	7611759	C	T	Missense_Mutation	281	0.793594306	No	1	Clonal	Disease_causing	Passenger	Disease_causing	likely_pathogenic	Yes	No	No	No	Not tested
CS5-C	Carcinoma	ELL2	p.S324L	Shared	5	95234498	G	A	Missense_Mutation	263	0.395437262	No	1	Clonal	Disease_causing	Passenger	Neutral	passenger	No	No	No	No	Not tested
CS5-S	Sarcoma	ELL2	p.S324L	Shared	5	95234498	G	A	Missense_Mutation	264	0.321969697	No	1	Clonal	Disease_causing	Passenger	Neutral	passenger	No	No	No	No	Not tested
CS5-C	Carcinoma	FGFR2	p.S252W	Shared	10	123279677	G	C	Missense_Mutation	238	0.378151261	Yes	1	Clonal	Neutral	Passenger	Neutral	likely_pathogenic	No	Yes	Yes	Yes	Not tested
CS5-S	Sarcoma	FGFR2	p.S252W	Shared	10	123279677	G	C	Missense_Mutation	199	0.336683417	Yes	1	Clonal	Neutral	Passenger	Neutral	likely_pathogenic	No	Yes	Yes	Yes	Not tested
CS5-C	Carcinoma	GLYR1	p.V320L	Shared	16	4864597	C	G	Missense_Mutation	98	0.448979592	No	1	Clonal	Disease_causing	Passenger	Neutral	passenger	No	No	No	No	Not tested
CS5-S	Sarcoma	GLYR1	p.V320L	Shared	16	4864597	C	G	Missense_Mutation	97	0.381443299	No	1	Clonal	Disease_causing	Passenger	Neutral	passenger	No	No	No	No	Not tested
CS5-C	Carcinoma	GPR149	p.L313F	Shared	3	154146466	C	G	Missense_Mutation	230	0.226086957	No	1	Clonal	Disease_causing	Passenger	Neutral	passenger	Yes	No	No	No	Not tested
CS5-S	Sarcoma	GPR149	p.L313F	Shared	3	154146466	C	G	Missense_Mutation	214	0.186915888	No	1	Clonal	Disease_causing	Passenger	Neutral	passenger	Yes	No	No	No	Not tested
CS5-C	Carcinoma	ITGAM	p.T1125I	Shared	16	31342580	C	T	Missense_Mutation	232	0.254310345	No	1	Clonal	Disease_causing	Passenger	Neutral	passenger	No	No	No	No	Not tested
CS5-S	Sarcoma	ITGAM	p.T1125I	Shared	16	31342580	C	T	Missense_Mutation	205	0.2	No	0.947	Clonal	Disease_causing	Passenger	Neutral	passenger	No	No	No	No	Not tested
CS5-C	Carcinoma	KIAA1324L	p.T808A	Shared	7	86537797	T	C	Missense_Mutation	308	0.441558442	No	1	Clonal	Neutral	Passenger	Neutral	passenger	No	No	No	No	Not tested
CS5-S	Sarcoma	KIAA1324L	p.T808A	Shared	7	86537797	T	C	Missense_Mutation	303	0.412541254	No	1	Clonal	Neutral	Passenger	Neutral	passenger	No	No	No	No	Not tested
CS5-C	Carcinoma	LRP1B	p.K4168N	Shared	2	141081472	T	A	Missense_Mutation	152	0.348684211	No	1	Clonal	Disease_causing	Passenger	Disease_causing	likely_pathogenic	Yes	Yes	No	No	Not tested
CS5-S	Sarcoma	LRP1B	p.K4168N	Shared	2	141081472	T	A	Missense_Mutation	130	0.307692308	No	1	Clonal	Disease_causing	Passenger	Disease_causing	likely_pathogenic	Yes	Yes	No	No	Not tested
CS5-C	Carcinoma	MISP	p.R106C	Shared	19	757262	C	T	Missense_Mutation	316	0.939873418	No	1	Clonal	Neutral	Passenger	Neutral	passenger	Yes	No	No	No	Not tested
CS5-S	Sarcoma	MISP	p.R106C	Shared	19	757262	C	T	Missense_Mutation	241	0.838174274	No	1	Clonal	Neutral	Passenger	Neutral	passenger	Yes	No	No	No	Not tested
CS5-C	Carcinoma	PCDH15	p.D1322H	Shared	10	55600099	C	G	Missense_Mutation	406	0.28817734	No	1	Clonal	Neutral	Passenger	Neutral	passenger	No	No	No	No	Not tested
CS5-S	Sarcoma	PCDH15	p.D1322H	Shared	10	55600099	C	G	Missense_Mutation	330	0.278787879	No	1	Clonal	Neutral	Passenger	Neutral	passenger	No	No	No	No	Not tested
CS5-C	Carcinoma	PIK3R1	p.A48T	Shared	5	67522645	G	A	Missense_Mutation	254	0.031496063	No	0.097	Subclonal	Disease_causing	Passenger	Neutral	passenger	No	Yes	Yes	Yes	Not tested
CS5-S	Sarcoma	PIK3R1	p.A48T	Shared	5	67522645	G	A	Missense_Mutation	270	0.033333333	No	0.124	Subclonal	Disease_causing	Passenger	Neutral	passenger	No	Yes	Yes	Yes	Not tested
CS5-C	Carcinoma	POLR3B	p.Q1082H	Shared	12	106898006	G	T	Missense_Mutation	174	0.942528736	No	1	Clonal	Disease_causing	Passenger	Neutral	passenger	Yes	No	No	No	Not tested
CS5-S	Sarcoma	POLR3B	p.Q1082H	Shared	12	106898006	G	T	Missense_Mutation	193	0.78238342	No	1	Clonal	Disease_causing	Passenger	Neutral	passenger	Yes	No	No	No	Not tested
CS5-C	Carcinoma	PPP2R1A	p.P179R	Shared	19	52715971	C	G	Missense_Mutation	264	0.46969697	Yes	1	Clonal	Disease_causing	Driver	Neutral	likely_pathogenic	No	Yes	Yes	Yes	Not tested
CS5-S	Sarcoma	PPP2R1A	p.P179R	Shared	19	52715971	C	G	Missense_Mutation	212	0.476415094	Yes	1	Clonal	Disease_causing	Driver	Neutral	likely_pathogenic	No	Yes	Yes	Yes	Not tested
CS5-C	Carcinoma	PRNP	p.Q98K	Shared	20	4680158	C	A	Missense_Mutation	371	0.212938005	No	1	Clonal	Disease_causing	Passenger	Disease_causing	likely_pathogenic	No	No	No	No	Not tested
CS5-S	Sarcoma	PRNP	p.Q98K	Shared	20	4680158	C	A	Missense_Mutation	304	0.220394737	No	1	Clonal	Disease_causing	Passenger	Disease_causing	likely_pathogenic	No	No	No	No	Not tested
CS5-C	Carcinoma	PWWP2B	p.Q261*	Shared	10	134218785	C	T	Nonsense_Mutation	976	0.351434426	No	1	Clonal	Neutral	.	.	passenger	No	No	No	No	Not tested
CS5-S	Sarcoma	PWWP2B	p.Q261*	Shared	10	134218785	C	T	Nonsense_Mutation	718	0.348189415	No	1	Clonal	Neutral	.	.	passenger	No	No	No	No	Not tested
CS5-C	Carcinoma	RRAS	p.G18E	Shared	19	50143303	C	T	Missense_Mutation	206	0.194174757	No	1	Clonal	Neutral	Passenger	Neutral	passenger	No	No	No	No	Not tested
CS5-S	Sarcoma	RRAS	p.G18E	Shared	19	50143303	C	T	Missense_Mutation	194	0.206185567	No	1	Clonal	Neutral	Passenger	Neutral	passenger	No	No	No	No	Not tested
CS5-C	Carcinoma	SALL4	p.A922V	Shared	20	50401201	G	A	Missense_Mutation	598	0.493311037	No	1	Clonal	Neutral	Passenger	Neutral	passenger	No	Yes	No	No	Not tested
CS5-S	Sarcoma	SALL4	p.A922V	Shared	20	50401201	G	A	Missense_Mutation	467	0.443254818	No	1	Clonal	Neutral	Passenger	Neutral	passenger	No	Yes	No	No	Not tested
CS5-C	Carcinoma	SHCBP1	p.T191A	Shared	16	46649883	T	C	Missense_Mutation	523	0.653919694	No	1	Clonal	Disease_causing	Passenger	Neutral	passenger	No	No	No	No	Not tested
CS5-S	Sarcoma	SHCBP1	p.T191A	Shared	16	46649883	T	C	Missense_Mutation	484	0.55785124	No	1	Clonal	Disease_causing	Passenger	Neutral	passenger	No	No	No	No	Not tested
CS5-C	Carcinoma	STARD8	p.G662A	Shared	X	67940201	G	C	Missense_Mutation	155	0.064516129	No	0.199	Subclonal	Disease_causing	Passenger	Neutral	passenger	No	No	No	No	Not tested
CS5-S	Sarcoma	STARD8	p.G662A	Shared	X	67940201	G	C	Missense_Mutation	134	0.067164179	No	0.251	Subclonal	Disease_causing	Passenger	Neutral	passenger	No	No	No	No	Not tested
CS5-C	Carcinoma	TELO2	p.A466V	Shared	16	1551699	C	T	Missense_Mutation	320	0.5125	No	1	Clonal	Neutral	Passenger	Disease_causing	passenger	No	No	No	No	Not tested
CS5-S	Sarcoma	TELO2	p.A466V	Shared	16	1551699	C	T	Missense_Mutation	312	0.423076923	No	1	Clonal	Neutral	Passenger	Disease_causing	passenger	No	No	No	No	Not tested
CS5-C	Carcinoma	TIAM2	p.H36N	Shared	6	155450463	C	A	Missense_Mutation	338	0.423076923	No	1	Clonal	Disease_causing	Driver	Neutral	likely_pathogenic	No	No	No	No	Not tested
CS5-S	Sarcoma	TIAM2	p.H36N	Shared	6	155450463	C	A	Missense_Mutation	276	0.362318841	No	1	Clonal	Disease_causing	Driver	Neutral	likely_pathogenic	No	No	No	No	Not tested
CS5-C	Carcinoma	TMEM110	p.X206_splice	Shared	3	52877736	C	T	Splice_Site	412	0.458737864	No	1	Clonal	Disease_causing	.	.	passenger	No	No	No	No	Not tested
CS5-S	Sarcoma	TMEM110	p.X206_splice	Shared	3	52877736	C	T	Splice_Site	383	0.375979112	No	1	Clonal	Disease_causing	.	.	passenger	No	No	No	No	Not tested
CS5-C	Carcinoma	TMTC2	p.L177Q	Shared	12	83251235	T	A	Missense_Mutation	261	0.942528736	No	1	Clonal	Disease_causing	Passenger	Neutral	passenger	Yes	No	No	No	Not tested
CS5-S	Sarcoma	TMTC2	p.L177Q	Shared	12	83251235	T	A	Missense_Mutation	241	0.784232365	No	1	Clonal	Disease_causing	Passenger	Neutral	passenger	Yes	No	No	No	Not tested
CS5-C	Carcinoma	TP53	p.V157Pfs*23	Shared	17	7578460	ACG	A	Frame_Shift_Del	243	0.880658436	No	1	Clonal	.	.	.	.	Yes	Yes	Yes	Yes	Not tested
CS5-S	Sarcoma	TP53	p.V157Pfs*23	Shared	17	7578460	ACG	A	Frame_Shift_Del	227	0.748898678	No	1	Clonal	.	.	.	.	Yes	Yes	Yes	Yes	Not tested
CS5-C	Carcinoma	TRIM54	p.Q236E	Shared	2	27527893	C	G	Missense_Mutation	269	0.31598513	No	1	Clonal	Disease_causing	Passenger	Neutral	passenger	Yes	No	No	No	Not tested
CS5-S	Sarcoma	TRIM54	p.Q236E	Shared	2	27527893	C	G	Missense_Mutation	260	0.261538462	No	1	Clonal	Disease_causing	Passenger	Neutral	passenger	Yes	No	No	No	Not tested
CS5-C	Carcinoma	VWA5B2	p.A1191E	Shared	3	183959669	C	A	Missense_Mutation	586	0.257679181	No	1	Clonal	Disease_causing	Passenger	Neutral	passenger	Yes	No	No	No	Not tested
CS5-S	Sarcoma	VWA5B2	p.A1191E	Shared	3	183959669	C	A	Missense_Mutation	555	0.268468468	No	1	Clonal	Disease_causing	Passenger	Neutral	passenger	Yes	No	No	No	Not tested
CS5-C	Carcinoma	ZNF285	p.E292Q	Shared	19	44891533	C	G	Missense_Mutation	233	0.236051502	No	1	Clonal	Neutral	Passenger	Neutral	passenger	No	No	No	No	Not tested
CS5-S	Sarcoma	ZNF285	p.E292Q	Shared	19	44891533	C	G	Missense_Mutation	199	0.16080402	No	1	Clonal	Neutral	Passenger	Neutral	passenger	No	No	No	No	Not tested
CS5-C	Carcinoma	ZNF45	p.S18F	Shared	19	44423814	G	A	Missense_Mutation	591	0.497461929	No	1	Clonal	Neutral	Passenger	Neutral	passenger	No	No	No	No	Not tested
CS5-S	Sarcoma	ZNF45	p.S18F	Shared	19	44423814	G	A	Missense_Mutation	515	0.45631068	No	1	Clonal	Neutral	Passenger	Neutral	passenger	No	No	No	No	Not tested
CS6-C	Carcinoma	AK9	p.S172I	Shared	6	109980546	C	A	Missense_Mutation	216	0.148148148	No	0.604	Subclonal	Neutral	Passenger	Neutral	passenger	No	No	No	No	Not tested
CS6-S	Sarcoma	AK9	p.S172I	Shared	6	109980546	C	A	Missense_Mutation	571	0.224168126	No	1	Clonal	Neutral	Passenger	Neutral	passenger	No	No	No	No	Not tested
CS6-C	Carcinoma	BSN	p.S2813A	Shared	3	49695426	T	G	Missense_Mutation	175	0.348571429	No	1	Clonal	Disease_causing	Passenger	Neutral	passenger	Yes	No	No	No	Not tested
CS6-S	Sarcoma	BSN	p.S2813A	Shared	3	49695426	T	G	Missense_Mutation	303	0.894389439	No	1	Clonal	Disease_causing	Passenger	Neutral	passenger	Yes	No	No	No	Not tested
CS6-C	Carcinoma	BZRAP1	p.S1417Kfs*40	Private	17	56386383	C	CT	Frame_Shift_Ins	278	0.165467626	No	0.675	Subclonal	.	.	.	.	No	No	No	No	Not tested
CS6-S	Sarcoma	C11orf83	p.G17E	Private	11	62439267	G	A	Missense_Mutation	320	0.05	No	0.162	Subclonal	Neutral	Passenger	.	passenger	No	No	No	No	Yes
CS6-S	Sarcoma	C19orf40	p.K149*	Private	19	33467385	A	T	Nonsense_Mutation	333	0.486486486	No	1	Clonal	Disease_causing	.	.	passenger	No	No	No	No	Yes
CS6-C	Carcinoma	CCDC101	p.X25_splice	Shared	16	28592466	G	T	Splice_Site	252	0.226190476	No	1	Clonal	Disease_causing	.	.	passenger	No	No	No	No	Not tested
CS6-S	Sarcoma	CCDC101	p.X25_splice	Shared	16	28592466	G	T	Splice_Site	760	0.440789474	No	1	Clonal	Disease_causing	.	.	passenger	No	No	No	No	Not tested
CS6-S	Sarcoma	COL4A2	p.P975S	Private	13	111137272	C	T	Missense_Mutation	193	0.062176166	No	0.201	Subclonal	Disease_causing	Passenger	Disease_causing	likely_pathogenic	No	No	No	No	Yes
CS6-S	Sarcoma	CSMD3	p.K3687M	Private	8	113237064	T	A	Missense_Mutation	892	0.117713004	No	1	Clonal	Disease_causing	Passenger	Neutral	passenger	No	No	No	No	Yes
CS6-S	Sarcoma	CTU1	p.S217L	Private	19	51602255	G	A	Missense_Mutation	83	0.578313253	No	1	Clonal	Disease_causing	Passenger	Neutral	passenger	No	No	No	No	Not tested
CS6-C	Carcinoma	CYLC1	p.D425Gfs*2	Shared	X	83128980	G	GA	Frame_Shift_Ins	184	0.266304348	No	0.819	Clonal	.	.	.	.	No	No	No	No	Not tested
CS6-S	Sarcoma	CYLC1	p.D425Gfs*2	Shared	X	83128980	G	GA	Frame_Shift_Ins	539	0.60296846	No	1	Clonal	.	.	.	.	Yes	No	No	No	Not tested
CS6-S	Sarcoma	DOC2B	p.X125_splice	Private	17	22408	C	T	Splice_Site	127	0.433070866	No	1	Clonal	.	.	.	likely_pathogenic	No	No	No	No	Yes
CS6-C	Carcinoma	DOCK4	p.V1084A	Private	7	111430577	A	G	Missense_Mutation	89	0.08988764	No	0.366	Subclonal	Disease_causing	Passenger	Neutral	passenger	No	No	No	No	Yes
CS6-S	Sarcoma	FBXL19	p.G66R	Private	16	30937211	G	A	Missense_Mutation	131	0.496183206	No	1	Clonal	Disease_causing	Passenger	Neutral	passenger	No	No	No	No	Yes
CS6-C	Carcinoma	FOXK1	p.T683P	Private	7	4801940	A	C	Missense_Mutation	84	0.095238095	No	0.388	Subclonal	Neutral	Passenger	Disease_causing	passenger	No	No	No	No	Not tested
CS6-C	Carcinoma	GRID1	p.R225C	Shared	10	87898629	G	A	Missense_Mutation	86	0.279069767	No	1	Clonal	Disease_causing	Driver	Disease_causing	likely_pathogenic	Yes	No	No	No	Not tested
CS6-S	Sarcoma	GRID1	p.R225C	Shared	10	87898629	G	A	Missense_Mutation	100	0.89	No	1	Clonal	Disease_causing	Driver	Disease_causing	likely_pathogenic	No	No	No	No	Not tested
CS6-C	Carcinoma	HEATR2	p.R253*	Private	7	769461	C	T	Nonsense_Mutation	291	0.041237113	No	0.168	Subclonal	Neutral	.	.	passenger	No	No	No	No	Yes
CS6-C	Carcinoma	HIVEP1	p.K1766T	Shared	6	12125325	A	C	Missense_Mutation	251	0.3187251	No	1	Clonal	Disease_causing	Passenger	Neutral	passenger	No	No	No	No	Not tested
CS6-S	Sarcoma	HIVEP1	p.K1766T	Shared	6	12125325	A	C	Missense_Mutation	515	0.431067961	No	1	Clonal	Disease_causing	Passenger	Neutral	passenger	No	No	No	No	Not tested
CS6-C	Carcinoma	HNRNPF	p.V327M	Private	10	43882354	C	T	Missense_Mutation	217	0.110599078	No	0.451	Subclonal	Disease_causing	Passenger	Neutral	passenger	No	No	No	No	Yes
CS6-C	Carcinoma	ILK	p.M155L	Private	11	6630129	A	T	Missense_Mutation	228	0.065789474	No	0.202	Subclonal	Disease_causing	Passenger	Neutral	passenger	Yes	No	No	No	Yes
CS6-S	Sarcoma	ITGA8	p.Q438R	Private	10	15686115	T	C	Missense_Mutation	341	0.442815249	No	1	Clonal	Neutral	Passenger	Neutral	passenger	No	No	No	No	Yes
CS6-C	Carcinoma	KCNV1	p.C27F	Private	8	110986538	C	A	Missense_Mutation	388	0.06443299	No	0.52	Subclonal	Disease_causing	Passenger	Disease_causing	likely_pathogenic	No	No	No	No	Not tested
CS6-C	Carcinoma	KRAS	p.G12V	Shared	12	25398284	C	A	Missense_Mutation	142	0.605633803	Yes	1	Clonal	Disease_causing	Driver	Neutral	likely_pathogenic	Yes	Yes	Yes	Yes	Not tested
CS6-S	Sarcoma	KRAS	p.G12V	Shared	12	25398284	C	A	Missense_Mutation	91	0.868131868	Yes	1	Clonal	Disease_causing	Driver	Neutral	likely_pathogenic	No	Yes	Yes	Yes	Not tested
CS6-C	Carcinoma	LAMA5	p.A907T	Private	20	60909262	C	T	Missense_Mutation	324	0.132716049	No	0.541	Subclonal	Disease_causing	Passenger	Neutral	passenger	No	No	No	No	Not tested
CS6-S	Sarcoma	MAMDC2	p.G195R	Private	9	72727988	G	A	Missense_Mutation	245	0.440816327	No	1	Clonal	Disease_causing	Passenger	Neutral	passenger	Yes	No	No	No	Yes
CS6-S	Sarcoma	MYH13	p.R1701G	Private	17	10212619	G	C	Missense_Mutation	156	0.038461538	No	0.125	Subclonal	Neutral	Driver	Disease_causing	likely_pathogenic	No	No	No	No	Yes
CS6-C	Carcinoma	MYO3A	p.R1313H	Shared	10	26463131	G	A	Missense_Mutation	284	0.323943662	No	1	Clonal	Neutral	Passenger	Neutral	passenger	No	No	No	No	Not tested
CS6-S	Sarcoma	MYO3A	p.R1313H	Shared	10	26463131	G	A	Missense_Mutation	586	0.455631399	No	1	Clonal	Neutral	Passenger	Neutral	passenger	No	No	No	No	Not tested
CS6-C	Carcinoma	NEK1	p.I19V	Private	4	170523727	T	C	Missense_Mutation	217	0.059907834	No	0.184	Subclonal	Disease_causing	Passenger	Neutral	passenger	Yes	No	No	No	Yes
CS6-S	Sarcoma	NELL1	p.I597S	Private	11	21581738	T	G	Missense_Mutation	266	0.42481203	No	1	Clonal	Disease_causing	Passenger	Disease_causing	likely_pathogenic	Yes	No	No	No	Yes
CS6-C	Carcinoma	OR13C8	p.K91R	Shared	9	107331720	A	G	Missense_Mutation	246	0.353658537	No	1	Clonal	Neutral	Passenger	Neutral	passenger	Yes	No	No	No	Not tested
CS6-S	Sarcoma	OR13C8	p.K91R	Shared	9	107331720	A	G	Missense_Mutation	440	0.852272727	No	1	Clonal	Neutral	Passenger	Neutral	passenger	Yes	No	No	No	Not tested
CS6-C	Carcinoma	OR6M1	p.A272T	Shared	11	123676244	C	T	Missense_Mutation	252	0.301587302	No	1	Clonal	Neutral	Passenger	Neutral	passenger	No	No	No	No	Not tested
CS6-S	Sarcoma	OR6M1	p.A272T	Shared	11	123676244	C	T	Missense_Mutation	404	0.334158416	No	1	Clonal	Neutral	Passenger	Neutral	passenger	No	No	No	No	Not tested
CS6-C	Carcinoma	PLEKHO1	p.G26A	Shared	1	150123148	G	C	Missense_Mutation	157	0.414012739	No	1	Clonal	Disease_causing	Passenger	Disease_causing	likely_pathogenic	No	No	No	No	Not tested
CS6-S	Sarcoma	PLEKHO1	p.G26A	Shared	1	150123148	G	C	Missense_Mutation	350	0.585714286	No	1	Clonal	Disease_causing	Passenger	Disease_causing	likely_pathogenic	Yes	No	No	No	Not tested
CS6-C	Carcinoma	POLR2A	p.S1962N	Private	17	7417468	G	A	Missense_Mutation	213	0.220657277	No	0.679	Subclonal	Disease_causing	Passenger	Neutral	passenger	Yes	No	No	No	Yes
CS6-C	Carcinoma	RLIM	p.S501L	Private	X	73811648	G	A	Missense_Mutation	153	0.039215686	No	0.121	Subclonal	Disease_causing	Passenger	Neutral	passenger	Yes	No	No	No	Not tested
CS6-C	Carcinoma	SLC40A1	p.E243K	Shared	2	190430113	C	T	Missense_Mutation	272	0.209558824	No	1	Clonal	Disease_causing	Passenger	Disease_causing	likely_pathogenic	Yes	No	No	No	Not tested
CS6-S	Sarcoma	SLC40A1	p.E243K	Shared	2	190430113	C	T	Missense_Mutation	444	0.182432432	No	0.773	Clonal	Disease_causing	Passenger	Disease_causing	likely_pathogenic	Yes	No	No	No	Not tested
CS6-S	Sarcoma	SPAG17	p.E1870Q	Private	1	118530741	C	G	Missense_Mutation	238	0.24789916	No	0.803	Subclonal	Neutral	Passenger	Neutral	passenger	Yes	No	No	No	Yes
CS6-C	Carcinoma	SYNE1	p.E696K	Shared	6	152784499	C	T	Missense_Mutation	191	0.130890052	No	0.534	Subclonal	Disease_causing	Passenger	Neutral	passenger	No	No	No	No	Not tested
CS6-S	Sarcoma	SYNE1	p.E696K	Shared	6	152784499	C	T	Missense_Mutation	457	0.245076586	No	1	Clonal	Disease_causing	Passenger	Neutral	passenger	No	No	No	No	Not tested
CS6-C	Carcinoma	TAF1	p.T978M	Shared	X	70612510	C	T	Missense_Mutation	152	0.381578947	No	1	Clonal	Disease_causing	Passenger	Neutral	passenger	No	No	Yes	No	Not tested
CS6-S	Sarcoma	TAF1	p.T978M	Shared	X	70612510	C	T	Missense_Mutation	444	0.731981982	No	1	Clonal	Disease_causing	Passenger	Neutral	passenger	Yes	No	Yes	No	Not tested
CS6-S	Sarcoma	TNFRSF10A	p.E130V	Private	8	23069643	T	A	Missense_Mutation	426	0.899061033	No	1	Clonal	Neutral	Passenger	Neutral	passenger	Yes	No	No	No	Yes
CS6-C	Carcinoma	UNC5A	p.G175V	Shared	5	176295649	G	T	Missense_Mutation	208	0.264423077	No	1	Clonal	Disease_causing	Passenger	Neutral	passenger	No	No	No	No	Not tested
CS6-S	Sarcoma	UNC5A	p.G175V	Shared	5	176295649	G	T	Missense_Mutation	182	0.510989011	No	1	Clonal	Disease_causing	Passenger	Neutral	passenger	No	No	No	No	Not tested
CS6-C	Carcinoma	UTP3	p.E249K	Private	4	71555139	G	A	Missense_Mutation	218	0.165137615	No	0.508	Subclonal	Neutral	Passenger	Neutral	passenger	Yes	No	No	No	Yes
CS6-C	Carcinoma	VAT1L	p.X294_splice	Private	16	77913122	G	T	Splice_Site	132	0.265151515	No	0.816	Clonal	Disease_causing	.	.	passenger	No	No	No	No	Yes
CS6-C	Carcinoma	WDR33	p.D1286G	Shared	2	128464051	T	C	Missense_Mutation	276	0.130434783	No	0.793	Clonal	Disease_causing	Passenger	Disease_causing	likely_pathogenic	Yes	No	No	No	Not tested
CS6-S	Sarcoma	WDR33	p.D1286G	Shared	2	128464051	T	C	Missense_Mutation	674	0.37537092	No	1	Clonal	Disease_causing	Passenger	Disease_causing	likely_pathogenic	Yes	No	No	No	Not tested
CS6-C	Carcinoma	ZC3HAV1L	p.P286L	Shared	7	138711483	G	A	Missense_Mutation	144	0.305555556	No	1	Clonal	Neutral	Passenger	.	passenger	Yes	No	No	No	Not tested
CS6-S	Sarcoma	ZC3HAV1L	p.P286L	Shared	7	138711483	G	A	Missense_Mutation	148	0.891891892	No	1	Clonal	Neutral	Passenger	.	passenger	No	No	No	No	Not tested
CS6-C	Carcinoma	ZFP62	p.S183N	Private	5	180277947	C	T	Missense_Mutation	262	0.080152672	No	0.407	Subclonal	Neutral	Passenger	Neutral	passenger	No	No	No	No	Not tested
CS6-C	Carcinoma	ZNF292	p.Y2407C	Shared	6	87970567	A	G	Missense_Mutation	232	0.336206897	No	1	Clonal	Disease_causing	Passenger	Neutral	passenger	No	No	No	No	Not tested
CS6-S	Sarcoma	ZNF292	p.Y2407C	Shared	6	87970567	A	G	Missense_Mutation	358	0.558659218	No	1	Clonal	Disease_causing	Passenger	Neutral	passenger	No	No	No	No	Not tested
CS6-C	Carcinoma	ZNF444	p.P155L	Shared	19	56671050	C	T	Missense_Mutation	76	0.197368421	No	1	Clonal	Disease_causing	Passenger	Neutral	passenger	No	No	No	No	Not tested
CS6-S	Sarcoma	ZNF444	p.P155L	Shared	19	56671050	C	T	Missense_Mutation	78	0.448717949	No	1	Clonal	Disease_causing	Passenger	Neutral	passenger	No	No	No	No	Not tested
CS6-C	Carcinoma	ZSCAN1	p.G285W	Shared	19	58565045	G	T	Missense_Mutation	240	0.241666667	No	1	Clonal	Disease_causing	Passenger	Neutral	passenger	No	No	No	No	Not tested
CS6-S	Sarcoma	ZSCAN1	p.G285W	Shared	19	58565045	G	T	Missense_Mutation	469	0.405117271	No	1	Clonal	Disease_causing	Passenger	Neutral	passenger	No	No	No	No	Not tested
CS6-C	Carcinoma	ZZEF1	p.T2105Hfs*27	Shared	17	3937580	T	TG	Frame_Shift_Ins	156	0.391025641	No	1	Clonal	.	.	.	.	Yes	No	No	No	Not tested
CS6-S	Sarcoma	ZZEF1	p.T2105Hfs*27	Shared	17	3937580	T	TG	Frame_Shift_Ins	244	0.81147541	No	1	Clonal	.	.	.	.	Yes	No	No	No	Not tested
CS8-C	Carcinoma	ABTB2	p.D1007H	Shared	11	34173993	C	G	Missense_Mutation	173	0.150289017	No	0.613	Subclonal	Disease_causing	Passenger	Neutral	passenger	No	No	No	No	Not tested
CS8-S	Sarcoma	ABTB2	p.D1007H	Shared	11	34173993	C	G	Missense_Mutation	199	0.40201005	No	1	Clonal	Disease_causing	Passenger	Neutral	passenger	No	No	No	No	Not tested
CS8-C	Carcinoma	ACVR1B	p.R456*	Shared	12	52380708	C	T	Nonsense_Mutation	683	0.452415813	No	1	Clonal	Neutral	.	.	likely_pathogenic	No	No	Yes	Yes	Not tested
CS8-S	Sarcoma	ACVR1B	p.R456*	Shared	12	52380708	C	T	Nonsense_Mutation	624	0.408653846	No	1	Clonal	Neutral	.	.	likely_pathogenic	No	No	Yes	Yes	Not tested
CS8-C	Carcinoma	ANK1	p.S462L	Private	8	41575141	G	A	Missense_Mutation	717	0.051603905	No	0.572	Subclonal	Disease_causing	Passenger	Neutral	passenger	No	No	No	No	Yes
CS8-C	Carcinoma	ANXA13	p.R134W	Private	8	124710709	G	A	Missense_Mutation	437	0.146453089	No	0.89	Clonal	Disease_causing	Passenger	Neutral	passenger	No	No	No	No	Yes
CS8-C	Carcinoma	BAZ1A	p.S843A	Private	14	35245431	A	C	Missense_Mutation	503	0.236580517	No	0.965	Clonal	Disease_causing	Passenger	Neutral	passenger	No	No	No	No	Yes
CS8-C	Carcinoma	C16orf52	p.R92*	Private	16	22086975	C	T	Nonsense_Mutation	234	0.226495726	No	0.923	Clonal	.	.	.	passenger	No	No	No	No	Yes
CS8-S	Sarcoma	C1orf222	p.Q96K	Private	1	1896371	G	T	Missense_Mutation	253	0.051383399	No	0.284	Subclonal	Disease_causing	Passenger	.	passenger	No	No	No	No	Not tested
CS8-C	Carcinoma	C5orf42	p.E923Q	Shared	5	37213814	C	G	Missense_Mutation	531	0.239171375	No	1	Clonal	Neutral	Passenger	Neutral	passenger	No	No	No	No	Not tested
CS8-S	Sarcoma	C5orf42	p.E923Q	Shared	5	37213814	C	G	Missense_Mutation	887	0.065388952	No	0.688	Subclonal	Neutral	Passenger	Neutral	passenger	No	No	No	No	Not tested
CS8-C	Carcinoma	CD1E	p.F8L	Shared	1	158323802	C	G	Missense_Mutation	482	0.473029046	No	1	Clonal	Neutral	Passenger	Neutral	passenger	No	No	No	No	Not tested
CS8-S	Sarcoma	CD1E	p.F8L	Shared	1	158323802	C	G	Missense_Mutation	501	0.383233533	No	1	Clonal	Neutral	Passenger	Neutral	passenger	No	No	No	No	Not tested
CS8-C	Carcinoma	CDKN2A	p.M52I	Shared	9	21971202	C	T	Missense_Mutation	127	0.448818898	No	1	Clonal	Disease_causing	Driver	Neutral	likely_pathogenic	Yes	Yes	Yes	Yes	Not tested
CS8-S	Sarcoma	CDKN2A	p.M52I	Shared	9	21971202	C	T	Missense_Mutation	175	0.377142857	No	1	Clonal	Disease_causing	Driver	Neutral	likely_pathogenic	Yes	Yes	Yes	Yes	Not tested
CS8-C	Carcinoma	CEP290	p.R614T	Shared	12	88508943	C	G	Missense_Mutation	193	0.259067358	No	1	Clonal	Disease_causing	Passenger	Neutral	passenger	No	No	No	No	Not tested
CS8-S	Sarcoma	CEP290	p.R614T	Shared	12	88508943	C	G	Missense_Mutation	204	0.401960784	No	1	Clonal	Disease_causing	Passenger	Neutral	passenger	No	No	No	No	Not tested
CS8-C	Carcinoma	CERS4	p.R276*	Shared	19	8322847	C	T	Nonsense_Mutation	262	0.435114504	No	1	Clonal	Disease_causing	.	.	likely_pathogenic	Yes	No	No	No	Not tested
CS8-S	Sarcoma	CERS4	p.R276*	Shared	19	8322847	C	T	Nonsense_Mutation	362	0.403314917	No	1	Clonal	Disease_causing	.	.	likely_pathogenic	Yes	No	No	No	Not tested
CS8-S	Sarcoma	CNBD1	p.K33Sfs*10	Private	8	87899774	GA	G	Frame_Shift_Del	543	0.208103131	No	1	Clonal	.	.	.	.	No	No	No	Yes	Not tested
CS8-S	Sarcoma	COPB1	p.G52V	Private	11	14515922	C	A	Missense_Mutation	481	0.122661123	No	0.8	Subclonal	Disease_causing	Passenger	Neutral	passenger	No	No	No	No	Not tested
CS8-C	Carcinoma	CYP11B1	p.R138H	Shared	8	143958621	C	T	Missense_Mutation	637	0.03610675	No	0.219	Subclonal	Disease_causing	Passenger	Neutral	passenger	No	No	No	No	Not tested
CS8-S	Sarcoma	CYP11B1	p.R138H	Shared	8	143958621	C	T	Missense_Mutation	649	0.209553159	No	1	Clonal	Disease_causing	Passenger	Neutral	passenger	No	No	No	No	Not tested
CS8-C	Carcinoma	DCAF13	p.S236F	Private	8	104432672	C	T	Missense_Mutation	640	0.05625	No	0.454	Subclonal	Disease_causing	Passenger	Neutral	passenger	No	No	No	No	Yes
CS8-C	Carcinoma	DDI2	p.L353F	Shared	1	15978264	C	T	Missense_Mutation	383	0.302872063	No	1	Clonal	Disease_causing	Passenger	Neutral	passenger	No	No	No	No	Not tested
CS8-S	Sarcoma	DDI2	p.L353F	Shared	1	15978264	C	T	Missense_Mutation	391	0.245524297	No	1	Clonal	Disease_causing	Passenger	Neutral	passenger	No	No	No	No	Not tested
CS8-S	Sarcoma	DNMT3A	p.A903V	Private	2	25457179	G	A	Missense_Mutation	252	0.162698413	No	0.898	Subclonal	Disease_causing	Passenger	Disease_causing	likely_pathogenic	Yes	Yes	Yes	Yes	Not tested
CS8-C	Carcinoma	DOC2A	p.L56V	Shared	16	30021378	G	C	Missense_Mutation	238	0.361344538	No	1	Clonal	Neutral	Passenger	Neutral	passenger	No	No	No	No	Not tested
CS8-S	Sarcoma	DOC2A	p.L56V	Shared	16	30021378	G	C	Missense_Mutation	295	0.301694915	No	1	Clonal	Neutral	Passenger	Neutral	passenger	No	No	No	No	Not tested
CS8-C	Carcinoma	EDN3	p.S181R	Private	20	57897427	T	A	Missense_Mutation	447	0.239373602	No	1	Clonal	Neutral	Passenger	Disease_causing	passenger	No	No	No	No	Not tested
CS8-C	Carcinoma	EIF3A	p.D975N	Private	10	120802109	C	T	Missense_Mutation	724	0.226519337	No	1	Clonal	Disease_causing	Passenger	Neutral	passenger	No	No	No	No	Yes
CS8-C	Carcinoma	ERG	p.X198_splice	Private	21	39774560	C	A	Splice_Site	156	0.096153846	No	0.392	Subclonal	Disease_causing	.	.	likely_pathogenic	No	Yes	No	No	Not tested
CS8-C	Carcinoma	GCDH	p.R243Q	Shared	19	13007111	G	A	Missense_Mutation	227	0.105726872	No	0.325	Subclonal	Disease_causing	Passenger	Disease_causing	likely_pathogenic	Yes	No	No	No	Not tested
CS8-S	Sarcoma	GCDH	p.R243Q	Shared	19	13007111	G	A	Missense_Mutation	321	0.408099688	No	1	Clonal	Disease_causing	Passenger	Disease_causing	likely_pathogenic	Yes	No	No	No	Not tested
CS8-C	Carcinoma	GLUD2	p.L359V	Shared	X	120182613	C	G	Missense_Mutation	507	0.114398422	No	0.352	Subclonal	Neutral	Passenger	Disease_causing	passenger	No	No	No	No	Not tested
CS8-S	Sarcoma	GLUD2	p.L359V	Shared	X	120182613	C	G	Missense_Mutation	497	0.555331992	No	1	Clonal	Neutral	Passenger	Disease_causing	passenger	No	No	No	No	Not tested
CS8-S	Sarcoma	GLYATL2	p.L211I	Private	11	58602156	G	T	Missense_Mutation	284	0.045774648	No	0.207	Subclonal	Neutral	Passenger	Neutral	passenger	No	No	No	No	Yes
CS8-C	Carcinoma	GSPT2	p.S169*	Shared	X	51487228	C	A	Nonsense_Mutation	279	0.487455197	No	1	Clonal	Disease_causing	.	.	passenger	No	No	No	No	Not tested
CS8-S	Sarcoma	GSPT2	p.S169*	Shared	X	51487228	C	A	Nonsense_Mutation	341	0.519061584	No	1	Clonal	Disease_causing	.	.	passenger	No	No	No	No	Not tested
CS8-C	Carcinoma	GYS2	p.D340N	Shared	12	21715896	C	T	Missense_Mutation	493	0.257606491	No	1	Clonal	Disease_causing	Passenger	Neutral	passenger	No	No	No	No	Not tested
CS8-S	Sarcoma	GYS2	p.D340N	Shared	12	21715896	C	T	Missense_Mutation	408	0.409313725	No	1	Clonal	Disease_causing	Passenger	Neutral	passenger	No	No	No	No	Not tested
CS8-C	Carcinoma	HEATR1	p.S1141L	Shared	1	236737539	G	A	Missense_Mutation	339	0.439528024	No	1	Clonal	Disease_causing	Passenger	Neutral	passenger	No	No	No	No	Not tested
CS8-S	Sarcoma	HEATR1	p.S1141L	Shared	1	236737539	G	A	Missense_Mutation	313	0.383386581	No	1	Clonal	Disease_causing	Passenger	Neutral	passenger	No	No	No	No	Not tested
CS8-C	Carcinoma	HTR1E	p.E21Q	Shared	6	87725113	G	C	Missense_Mutation	792	0.448232323	No	1	Clonal	Disease_causing	Passenger	Neutral	passenger	No	No	No	No	Not tested
CS8-S	Sarcoma	HTR1E	p.E21Q	Shared	6	87725113	G	C	Missense_Mutation	691	0.455861071	No	1	Clonal	Disease_causing	Passenger	Neutral	passenger	No	No	No	No	Not tested
CS8-C	Carcinoma	IDE	p.R862K	Shared	10	94223664	C	T	Missense_Mutation	109	0.366972477	No	1	Clonal	Disease_causing	Driver	Neutral	likely_pathogenic	No	No	No	No	Not tested
CS8-S	Sarcoma	IDE	p.R862K	Shared	10	94223664	C	T	Missense_Mutation	92	0.326086957	No	1	Clonal	Disease_causing	Driver	Neutral	likely_pathogenic	No	No	No	No	Not tested
CS8-C	Carcinoma	ITGB7	p.F325V	Shared	12	53589827	A	C	Missense_Mutation	471	0.394904459	No	1	Clonal	Disease_causing	Passenger	Disease_causing	likely_pathogenic	No	No	No	Yes	Not tested
CS8-S	Sarcoma	ITGB7	p.F325V	Shared	12	53589827	A	C	Missense_Mutation	387	0.382428941	No	1	Clonal	Disease_causing	Passenger	Disease_causing	likely_pathogenic	No	No	No	Yes	Not tested
CS8-C	Carcinoma	JMJD1C	p.S874L	Shared	10	64973306	G	A	Missense_Mutation	340	0.305882353	No	1	Clonal	Disease_causing	Passenger	Neutral	passenger	No	No	No	No	Not tested
CS8-S	Sarcoma	JMJD1C	p.S874L	Shared	10	64973306	G	A	Missense_Mutation	300	0.32	No	1	Clonal	Disease_causing	Passenger	Neutral	passenger	No	No	No	No	Not tested
CS8-C	Carcinoma	KCNT2	p.D603Y	Shared	1	196303167	C	A	Missense_Mutation	124	0.516129032	No	1	Clonal	Disease_causing	Passenger	Neutral	passenger	No	No	No	No	Not tested
CS8-S	Sarcoma	KCNT2	p.D603Y	Shared	1	196303167	C	A	Missense_Mutation	158	0.537974684	No	1	Clonal	Disease_causing	Passenger	Neutral	passenger	No	No	No	No	Not tested
CS8-C	Carcinoma	LAMA2	p.S2128C	Shared	6	129766920	C	G	Missense_Mutation	255	0.043137255	No	0.219	Subclonal	Disease_causing	Driver	Neutral	likely_pathogenic	No	No	No	No	Not tested
CS8-S	Sarcoma	LAMA2	p.S2128C	Shared	6	129766920	C	G	Missense_Mutation	239	0.251046025	No	1	Clonal	Disease_causing	Driver	Neutral	likely_pathogenic	No	No	No	No	Not tested
CS8-C	Carcinoma	LRP1B	p.E3793Q	Shared	2	141115566	C	G	Missense_Mutation	423	0.307328605	No	1	Clonal	Disease_causing	Passenger	Disease_causing	likely_pathogenic	Yes	Yes	No	No	Not tested
CS8-S	Sarcoma	LRP1B	p.E3793Q	Shared	2	141115566	C	G	Missense_Mutation	452	0.376106195	No	1	Clonal	Disease_causing	Passenger	Disease_causing	likely_pathogenic	Yes	Yes	No	No	Not tested
CS8-C	Carcinoma	LRRC41	p.E62K	Shared	1	46768811	C	T	Missense_Mutation	589	0.631578947	No	1	Clonal	Disease_causing	Passenger	Disease_causing	likely_pathogenic	No	No	No	No	Not tested
CS8-S	Sarcoma	LRRC41	p.E62K	Shared	1	46768811	C	T	Missense_Mutation	610	0.686885246	No	1	Clonal	Disease_causing	Passenger	Disease_causing	likely_pathogenic	No	No	No	No	Not tested
CS8-C	Carcinoma	LRRN4CL	p.E170Q	Shared	11	62455473	C	G	Missense_Mutation	410	0.402439024	No	1	Clonal	Neutral	Passenger	.	passenger	No	No	No	No	Not tested
CS8-S	Sarcoma	LRRN4CL	p.E170Q	Shared	11	62455473	C	G	Missense_Mutation	386	0.287564767	No	1	Clonal	Neutral	Passenger	.	passenger	No	No	No	No	Not tested
CS8-C	Carcinoma	LTK	p.E374Q	Private	15	41800396	C	G	Missense_Mutation	261	0.275862069	No	1	Clonal	Neutral	Passenger	Neutral	passenger	No	No	No	No	Yes
CS8-C	Carcinoma	MAP4K2	p.R529C	Private	11	64564013	G	A	Missense_Mutation	377	0.047745358	No	0.195	Subclonal	Disease_causing	Passenger	Neutral	passenger	No	No	No	No	Yes
CS8-S	Sarcoma	MDGA2	p.D203N	Private	14	47530476	C	T	Missense_Mutation	384	0.0703125	No	0.388	Subclonal	Disease_causing	Passenger	Neutral	passenger	No	No	No	No	Not tested
CS8-S	Sarcoma	MUC6	p.A1471T	Private	11	1018390	C	T	Missense_Mutation	148	0.067567568	No	0.373	Subclonal	Neutral	Passenger	Neutral	passenger	No	No	No	No	Not tested
CS8-C	Carcinoma	MYH4	p.R1341C	Shared	17	10353930	G	A	Missense_Mutation	87	0.114942529	No	0.354	Subclonal	Disease_causing	Passenger	Disease_causing	likely_pathogenic	Yes	No	No	No	Not tested
CS8-S	Sarcoma	MYH4	p.R1341C	Shared	17	10353930	G	A	Missense_Mutation	182	0.489010989	No	1	Clonal	Disease_causing	Passenger	Disease_causing	likely_pathogenic	Yes	No	No	No	Not tested
CS8-C	Carcinoma	NCAPG	p.K816N	Private	4	17839406	G	C	Missense_Mutation	529	0.175803403	No	1	Clonal	Disease_causing	Passenger	Neutral	passenger	No	No	No	No	Yes
CS8-C	Carcinoma	NCOA2	p.S267*	Shared	8	71075732	G	C	Nonsense_Mutation	1054	0.164136622	No	1	Clonal	Disease_causing	.	.	likely_pathogenic	No	Yes	No	No	Not tested
CS8-S	Sarcoma	NCOA2	p.S267*	Shared	8	71075732	G	C	Nonsense_Mutation	1017	0.244837758	No	1	Clonal	Disease_causing	.	.	likely_pathogenic	No	Yes	No	No	Not tested
CS8-C	Carcinoma	NLRP9	p.D205H	Shared	19	56244584	C	G	Missense_Mutation	424	0.259433962	No	1	Clonal	Neutral	Passenger	Neutral	passenger	No	No	No	No	Not tested
CS8-C	Carcinoma	NLRP9	p.E358Q	Shared	19	56244125	C	G	Missense_Mutation	813	0.236162362	No	1	Clonal	Neutral	Passenger	Disease_causing	passenger	No	No	No	No	Not tested
CS8-S	Sarcoma	NLRP9	p.D205H	Shared	19	56244584	C	G	Missense_Mutation	401	0.311720698	No	1	Clonal	Neutral	Passenger	Neutral	passenger	No	No	No	No	Not tested
CS8-S	Sarcoma	NLRP9	p.E358Q	Shared	19	56244125	C	G	Missense_Mutation	924	0.306277056	No	1	Clonal	Neutral	Passenger	Disease_causing	passenger	No	No	No	No	Not tested
CS8-C	Carcinoma	NPEPPS	p.E353D	Shared	17	45664674	A	T	Missense_Mutation	76	0.118421053	No	0.601	Subclonal	Disease_causing	Passenger	Neutral	passenger	No	No	No	No	Not tested
CS8-S	Sarcoma	NPEPPS	p.E353D	Shared	17	45664674	A	T	Missense_Mutation	83	0.084337349	No	0.465	Subclonal	Disease_causing	Passenger	Neutral	passenger	No	No	No	No	Not tested
CS8-C	Carcinoma	PARPBP	p.S243L	Shared	12	102559568	C	T	Missense_Mutation	293	0.262798635	No	1	Clonal	Disease_causing	Passenger	Neutral	passenger	No	No	No	No	Not tested
CS8-S	Sarcoma	PARPBP	p.S243L	Shared	12	102559568	C	T	Missense_Mutation	295	0.423728814	No	1	Clonal	Disease_causing	Passenger	Neutral	passenger	No	No	No	No	Not tested
CS8-S	Sarcoma	POC5	p.S122N	Private	5	74998578	C	T	Missense_Mutation	182	0.038461538	No	0.212	Subclonal	Neutral	Passenger	Neutral	passenger	No	No	No	No	Yes
CS8-C	Carcinoma	PRG4	p.P851S	Shared	1	186277402	C	T	Missense_Mutation	763	0.229357798	No	1	Clonal	Neutral	Passenger	Neutral	passenger	No	No	No	No	Not tested
CS8-S	Sarcoma	PRG4	p.P851S	Shared	1	186277402	C	T	Missense_Mutation	811	0.23674476	No	1	Clonal	Neutral	Passenger	Neutral	passenger	No	No	No	No	Not tested
CS8-C	Carcinoma	PSD	p.R171W	Shared	10	104176285	G	A	Missense_Mutation	225	0.6	No	1	Clonal	Neutral	Passenger	Neutral	passenger	No	No	No	No	Not tested
CS8-S	Sarcoma	PSD	p.R171W	Shared	10	104176285	G	A	Missense_Mutation	225	0.346666667	No	1	Clonal	Neutral	Passenger	Neutral	passenger	No	No	No	No	Not tested
CS8-C	Carcinoma	QPCTL	p.K172N	Shared	19	46198859	G	C	Missense_Mutation	942	0.250530786	No	1	Clonal	Disease_causing	Passenger	Neutral	passenger	No	No	No	No	Not tested
CS8-S	Sarcoma	QPCTL	p.K172N	Shared	19	46198859	G	C	Missense_Mutation	936	0.303418803	No	1	Clonal	Disease_causing	Passenger	Neutral	passenger	No	No	No	No	Not tested
CS8-S	Sarcoma	RALGAPB	p.S470G	Private	20	37146634	A	G	Missense_Mutation	432	0.12962963	No	0.975	Clonal	Disease_causing	Passenger	.	passenger	No	No	No	No	Not tested
CS8-C	Carcinoma	SETD1A	p.I171M	Shared	16	30972854	C	G	Missense_Mutation	101	0.326732673	No	1	Clonal	Disease_causing	Passenger	Disease_causing	likely_pathogenic	No	No	No	No	Not tested
CS8-S	Sarcoma	SETD1A	p.I171M	Shared	16	30972854	C	G	Missense_Mutation	125	0.248	No	1	Clonal	Disease_causing	Passenger	Disease_causing	likely_pathogenic	No	No	No	No	Not tested
CS8-C	Carcinoma	SLITRK2	p.M328I	Shared	X	144904927	G	A	Missense_Mutation	554	0.034296029	No	0.106	Subclonal	Disease_causing	Passenger	Neutral	passenger	No	No	No	No	Not tested
CS8-S	Sarcoma	SLITRK2	p.M328I	Shared	X	144904927	G	A	Missense_Mutation	607	0.280065898	No	1	Clonal	Disease_causing	Passenger	Neutral	passenger	No	No	No	No	Not tested
CS8-S	Sarcoma	SOS2	p.L1108R	Private	14	50597233	A	C	Missense_Mutation	309	0.055016181	No	0.304	Subclonal	Disease_causing	Passenger	Neutral	passenger	No	No	No	No	Not tested
CS8-C	Carcinoma	SWAP70	p.F122L	Shared	11	9735138	C	G	Missense_Mutation	371	0.380053908	No	1	Clonal	Disease_causing	Passenger	Neutral	passenger	No	No	No	No	Not tested
CS8-S	Sarcoma	SWAP70	p.F122L	Shared	11	9735138	C	G	Missense_Mutation	601	0.146422629	No	0.955	Clonal	Disease_causing	Passenger	Neutral	passenger	No	No	No	No	Not tested
CS8-C	Carcinoma	TBC1D10A	p.Q88H	Shared	22	30700586	C	G	Missense_Mutation	548	0.233576642	No	1	Clonal	Disease_causing	Passenger	Neutral	passenger	No	No	No	No	Not tested
CS8-S	Sarcoma	TBC1D10A	p.Q88H	Shared	22	30700586	C	G	Missense_Mutation	337	0.181008902	No	0.999	Clonal	Disease_causing	Passenger	Neutral	passenger	No	No	No	No	Not tested
CS8-S	Sarcoma	TEX13B	p.A192P	Private	X	107224675	C	G	Missense_Mutation	496	0.116935484	No	0.645	Subclonal	Neutral	Passenger	.	passenger	No	No	No	No	Not tested
CS8-C	Carcinoma	THAP3	p.S217C	Shared	1	6693067	C	G	Missense_Mutation	252	0.297619048	No	1	Clonal	Neutral	Passenger	Disease_causing	passenger	No	No	No	No	Not tested
CS8-S	Sarcoma	THAP3	p.S217C	Shared	1	6693067	C	G	Missense_Mutation	275	0.250909091	No	1	Clonal	Neutral	Passenger	Disease_causing	passenger	No	No	No	No	Not tested

CS8-C	Carcinoma	THEMIS	p.Q651H	Private	6	128040891	C	G	Missense_Mutation	[illegible]	[illegible]	No	0.885	Clonal	Neutral	Passenger	Neutral	passenger	No	No	No	No	Yes
CS8-C	Carcinoma	TMEM232	p.V348I	Shared	5	109941044	C	T	Missense_Mutation	[illegible]	[illegible]	No	1	Clonal	Neutral	Passenger	.	passenger	No	No	No	No	Not tested
CS8-S	Sarcoma	TMEM232	p.V348I	Shared	5	109941044	C	T	Missense_Mutation	342	0.274853801	No	1	Clonal	Neutral	Passenger	.	passenger	No	No	No	No	Not tested
CS8-C	Carcinoma	TPR	p.E2170*	Shared	1	186289504	C	A	Nonsense_Mutation	366	0.224043716	No	1	Clonal	Neutral	.	.	likely_pathogenic	No	Yes	No	No	Not tested
CS8-S	Sarcoma	TPR	p.E2170*	Shared	1	186289504	C	A	Nonsense_Mutation	438	0.340182648	No	1	Clonal	Neutral	.	.	likely_pathogenic	No	Yes	No	No	Not tested
CS8-C	Carcinoma	UBQLN3	p.A556E	Shared	11	5529122	G	T	Missense_Mutation	412	0.046116505	No	0.188	Subclonal	Neutral	Passenger	Neutral	passenger	No	No	No	No	Not tested
CS8-S	Sarcoma	UBQLN3	p.A556E	Shared	11	5529122	G	T	Missense_Mutation	506	0.128458498	No	0.837	Clonal	Neutral	Passenger	Neutral	passenger	No	No	No	No	Not tested
CS8-C	Carcinoma	UGGT2	p.D1335H	Shared	13	96508417	C	G	Missense_Mutation	347	0.190201729	No	0.966	Clonal	Disease_causing	Passenger	Disease_causing	likely_pathogenic	No	No	No	No	Not tested
CS8-S	Sarcoma	UGGT2	p.D1335H	Shared	13	96508417	C	G	Missense_Mutation	267	0.333333333	No	1	Clonal	Disease_causing	Passenger	Disease_causing	likely_pathogenic	No	No	No	No	Not tested
CS8-C	Carcinoma	UXS1	p.I24M	Private	2	106810626	G	C	Missense_Mutation	157	0.292993631	No	1	Clonal	Neutral	Passenger	Disease_causing	passenger	Yes	No	No	No	Yes
CS8-C	Carcinoma	VPS13B	p.L2627F	Private	8	100796569	G	T	Missense_Mutation	742	0.074123989	No	0.599	Subclonal	Disease_causing	Passenger	Neutral	passenger	No	No	No	No	Not tested
CS8-C	Carcinoma	WWP1	p.S174L	Shared	8	87410849	C	T	Missense_Mutation	1089	0.132231405	No	0.407	Subclonal	Disease_causing	Passenger	Neutral	passenger	No	No	No	No	Not tested
CS8-S	Sarcoma	WWP1	p.S174L	Shared	8	87410849	C	T	Missense_Mutation	1055	0.114691943	No	1	Clonal	Disease_causing	Passenger	Neutral	passenger	No	No	No	No	Not tested
CS8-C	Carcinoma	ZC3H18	p.R436Q	Shared	16	88677776	G	A	Missense_Mutation	109	0.275229358	No	1	Clonal	Disease_causing	Passenger	Neutral	passenger	No	No	No	No	Not tested
CS8-S	Sarcoma	ZC3H18	p.R436Q	Shared	16	88677776	G	A	Missense_Mutation	95	0.189473684	No	1	Clonal	Disease_causing	Passenger	Neutral	passenger	No	No	No	No	Not tested
CS8-C	Carcinoma	ZCCHC8	p.E602K	Shared	12	122958364	C	T	Missense_Mutation	662	0.244712991	No	1	Clonal	Disease_causing	Passenger	Neutral	passenger	No	No	No	No	Not tested
CS8-C	Carcinoma	ZCCHC8	p.E569Q	Private	12	122958463	C	G	Missense_Mutation	741	0.241565452	No	1	Clonal	Neutral	Passenger	Neutral	passenger	No	No	No	No	Not tested
CS8-S	Sarcoma	ZCCHC8	p.E602K	Shared	12	122958364	C	T	Missense_Mutation	555	0.054054054	No	0.298	Clonal	Disease_causing	Passenger	Neutral	passenger	No	No	No	No	Not tested
CS8-C	Carcinoma	ZFHX2	p.V1924Gfs*3	Shared	14	23993380	A	AC	Frame_Shift_Ins	361	0.24099723	No	0.983	Clonal	.	.	.	.	No	No	No	No	Not tested
CS8-S	Sarcoma	ZFHX2	p.V1924Gfs*3	Shared	14	23993380	A	AC	Frame_Shift_Ins	347	0.178674352	No	0.986	Clonal	.	.	.	.	No	No	No	No	Not tested
CS8-S	Sarcoma	ZNF30	p.S59Y	Private	19	35424544	C	A	Missense_Mutation	305	0.095081967	No	0.715	Subclonal	Neutral	Passenger	Neutral	passenger	No	No	No	No	Not tested
CS8-C	Carcinoma	ZNF644	p.S648L	Shared	1	91404968	G	A	Missense_Mutation	421	0.270783848	No	1	Clonal	Disease_causing	Passenger	Neutral	passenger	No	No	No	No	Not tested
CS8-S	Sarcoma	ZNF644	p.S648L	Shared	1	91404968	G	A	Missense_Mutation	436	0.240825688	No	1	Clonal	Disease_causing	Passenger	Neutral	passenger	No	No	No	No	Not tested
CS8-C	Carcinoma	ZNF770	p.S546F	Shared	15	35273999	G	A	Missense_Mutation	204	0.294117647	No	1	Clonal	Neutral	Passenger	Neutral	passenger	No	No	No	No	Not tested
CS8-S	Sarcoma	ZNF770	p.S546F	Shared	15	35273999	G	A	Missense_Mutation	161	0.304347826	No	1	Clonal	Neutral	Passenger	Neutral	passenger	Yes	No	No	No	Not tested
CS8-C	Carcinoma	ZP2	p.P86L	Shared	16	21221025	G	A	Missense_Mutation	260	0.038461538	No	0.157	Subclonal	Neutral	Passenger	Neutral	passenger	No	No	No	No	Not tested
CS8-S	Sarcoma	ZP2	p.P86L	Shared	16	21221025	G	A	Missense_Mutation	287	0.216027875	No	1	Clonal	Neutral	Passenger	Neutral	passenger	No	No	No	No	Not tested

Supplementary Table S3. DAVID pathway analysis in 35 metaplastic breast cancers from Ng et al (Clin Cancer Res 2017) and 55 non-hypermutated uterine carcinosarcomas from Cherniack et al (TCGA, 2017).

DAVID pathway analysis in metaplastic breast carcinomas.

Database/ gene set ID	Description Term	Number of genes in gene set	Number of genes in overlap	*P* value
KEGG_PATHWAY_hsa05200	Pathways in cancer	393	45	3.34E-21
KEGG_PATHWAY_hsa05202	Transcriptional misregulation in cancer	168	27	5.12E-16
**KEGG_PATHWAY_hsa04115**	**p53 signaling pathway**	**67**	**14**	**9.32E-10**
KEGG_PATHWAY_hsa04110	Cell cycle	124	17	5.34E-09
**KEGG_PATHWAY_hsa04151**	**PI3K-Akt signaling pathway**	**345**	**27**	**1.02E-08**
KEGG_PATHWAY_hsa05230	Central carbon metabolism in cancer	64	11	7.44E-07
KEGG_PATHWAY_hsa04012	ErbB signaling pathway	87	12	1.82E-06
KEGG_PATHWAY_hsa04068	FoxO signaling pathway	134	14	4.32E-06
KEGG_PATHWAY_hsa04066	HIF-1 signaling pathway	98	11	3.72E-05
KEGG_PATHWAY_hsa04510	Focal adhesion	206	15	1.05E-04
KEGG_PATHWAY_hsa04550	Signaling pathways regulating pluripotency of stem cells	140	12	1.69E-04
KEGG_PATHWAY_hsa04630	Jak-STAT signaling pathway	145	11	9.41E-04
**KEGG_PATHWAY_hsa04310**	**Wnt signaling pathway**	**138**	**10**	**0.0024866**
KEGG_PATHWAY_hsa04520	Adherens junction	71	7	0.0037188
KEGG_PATHWAY_hsa04390	Hippo signaling pathway	151	10	0.0045419
KEGG_PATHWAY_hsa00310	Lysine degradation	52	6	0.0045992
KEGG_PATHWAY_hsa04015	Rap1 signaling pathway	210	12	0.0047454
KEGG_PATHWAY_hsa04150	mTOR signaling pathway	58	6	0.0073311
KEGG_PATHWAY_hsa04611	Platelet activation	130	8	0.0198681
KEGG_PATHWAY_hsa04014	Ras signaling pathway	226	11	0.0210506
KEGG_PATHWAY_hsa04370	VEGF signaling pathway	61	5	0.0394607
KEGG_PATHWAY_hsa04210	Apoptosis	62	5	0.0415144
KEGG_PATHWAY_hsa04650	Natural killer cell mediated cytotoxicity	122	7	0.0440307
BIOCARTA_h_ctcfPathway	CTCF: First Multivalent Nuclear Factor	25	12	4.59E-09
BIOCARTA_h_atrbrcaPathway	Role of BRCA1, BRCA2 and ATR in Cancer Susceptibility:	22	8	4.65E-05
BIOCARTA_h_g1Pathway	Cell Cycle: G1/S Check Point	30	9	5.36E-05
BIOCARTA_h_cellcyclePathway	Cyclins and Cell Cycle Regulation	25	8	1.16E-04
BIOCARTA_h_raccycdPathway	Influence of Ras and Rho proteins on G1 to S Transition	27	8	1.99E-04
BIOCARTA_h_g2Pathway	Cell Cycle: G2/M Checkpoint	25	7	9.21E-04
**BIOCARTA_h_p53Pathway**	**p53 Signaling Pathway**	**17**	**6**	**9.34E-04**
BIOCARTA_h_atmPathway	ATM Signaling Pathway	21	6	0.0026347
BIOCARTA_h_her2Pathway	Role of ERBB2 in Signal Transduction and Oncology	23	6	0.004035
BIOCARTA_h_gleevecPathway	Inhibition of Cellular Proliferation by Gleevec	23	6	0.004035
BIOCARTA_h_tpoPathway	TPO Signaling Pathway	24	6	0.0049053
BIOCARTA_h_plcPathway	Phospholipase C Signaling Pathway	9	4	0.0075159
BIOCARTA_h_il7Pathway	IL-7 Signal Transduction	17	5	0.007592
BIOCARTA_h_ghPathway	Growth Hormone Signaling Pathway	27	6	0.0083071
BIOCARTA_h_ptenPathway	PTEN dependent cell cycle arrest and apoptosis	**18**	**5**	**0.0094055**
BIOCARTA_h_il2rbPathway	IL-2 Receptor Beta Chain in T cell Activation	39	7	0.0098287
BIOCARTA_h_vegfPathway	VEGF, Hypoxia, and Angiogenesis	31	6	0.0150113
BIOCARTA_h_mtorPathway	mTOR Signaling Pathway	26	5	0.0342099
REACTOME_PATHWAY_R-HSA-2122947	NOTCH1 Intracellular Domain Regulates Transcription	47	7	1.26E-04
REACTOME_PATHWAY_R-HSA-2894862	Constitutive Signaling by NOTCH1 PEST Domain Mutants	57	7	3.72E-04
REACTOME_PATHWAY_R-HSA-2644606	Constitutive Signaling by NOTCH1 HD+PEST Domain Mutants	57	7	3.72E-04
REACTOME_PATHWAY_R-HSA-1963642	PI3K events in ERBB2 signaling	16	5	1.21E-04
REACTOME_PATHWAY_R-HSA-5617472	Activation of anterior HOX genes in hindbrain development during early embryogenesis	122	10	2.10E-04
REACTOME_PATHWAY_R-HSA-1257604	PIP3 activates AKT signaling	81	8	4.13E-04
REACTOME_PATHWAY_R-HSA-2219530	Constitutive Signaling by Aberrant PI3K in Cancer	61	7	5.38E-04
REACTOME_PATHWAY_R-HSA-3769402	Deactivation of the beta-catenin transactivating complex	42	6	6.56E-04
REACTOME_PATHWAY_R-HSA-5673001	RAF/MAP kinase cascade	116	9	7.42E-04
REACTOME_PATHWAY_R-HSA-381340	Transcriptional regulation of white adipocyte differentiation	79	7	0.0021021
REACTOME_PATHWAY_R-HSA-5693568	Resolution of D-loop Structures through Holliday Junction Intermediates	33	5	0.0021725
REACTOME_PATHWAY_R-HSA-114604	GPVI-mediated activation cascade	56	6	0.0024394
REACTOME_PATHWAY_R-HSA-3214858	RMTs methylate histone arginines	77	6	0.0095217
REACTOME_PATHWAY_R-HSA-5655253	Signaling by FGFR2 in disease	27	4	0.0102173
REACTOME_PATHWAY_R-HSA-1660499	Synthesis of PIPs at the plasma membrane	35	4	0.0207352
REACTOME_PATHWAY_R-HSA-5467340	AXIN missense mutants destabilize the destruction complex	14	3	0.0225016
REACTOME_PATHWAY_R-HSA-5467348	Truncations of AMER1 destabilize the destruction complex	14	3	0.0225016
REACTOME_PATHWAY_R-HSA-3232118	SUMOylation of transcription factors	17	3	0.0325409
REACTOME_PATHWAY_R-HSA-1236382	Constitutive Signaling by Ligand-Responsive EGFR Cancer Variants	19	3	0.0400312
REACTOME_PATHWAY_R-HSA-983231	Factors involved in megakaryocyte development and platelet production	112	6	0.040662
**REACTOME_PATHWAY_R-HSA-1912408**	**Pre-NOTCH Transcription and Translation**	**29**	**6**	**1.09E-04**
REACTOME_PATHWAY_R-HSA-2197563	Notch-HLH transcription pathway	12	3	0.0166828

DAVID pathway analysis in uterine carcinosarcomas.

Database/ gene set ID	Description Term	Number of genes in gene set	Number of genes in overlap	*P* value
KEGG_PATHWAY_hsa05202	Transcriptional misregulation in cancer	168	35	3.16E-21
KEGG_PATHWAY_hsa05200	Pathways in cancer	393	49	5.34E-20
KEGG_PATHWAY_hsa05230	Central carbon metabolism in cancer	64	16	7.47E-11
KEGG_PATHWAY_hsa04550	Signaling pathways regulating pluripotency of stem cells	140	20	4.32E-09
KEGG_PATHWAY_hsa04012	ErbB signaling pathway	87	16	7.19E-09
**KEGG_PATHWAY_hsa04151**	**PI3K-Akt signaling pathway**	**345**	**27**	**1.35E-06**
KEGG_PATHWAY_hsa05206	MicroRNAs in cancer	285	24	1.82E-06
KEGG_PATHWAY_hsa04014	Ras signaling pathway	226	21	2.20E-06
KEGG_PATHWAY_hsa04068	FoxO signaling pathway	134	16	2.48E-06
KEGG_PATHWAY_hsa04110	Cell cycle	124	15	4.91E-06
KEGG_PATHWAY_hsa04066	HIF-1 signaling pathway	98	13	1.03E-05
KEGG_PATHWAY_hsa04510	Focal adhesion	206	18	3.31E-05
KEGG_PATHWAY_hsa04520	Adherens junction	71	10	1.03E-04
KEGG_PATHWAY_hsa04015	Rap1 signaling pathway	210	17	1.46E-04
KEGG_PATHWAY_hsa04370	VEGF signaling pathway	61	9	1.98E-04
**KEGG_PATHWAY_hsa04310**	**Wnt signaling pathway**	**138**	**13**	**3.04E-04**
KEGG_PATHWAY_hsa04662	B cell receptor signaling pathway	69	9	4.69E-04
KEGG_PATHWAY_hsa04630	Jak-STAT signaling pathway	145	13	4.80E-04
KEGG_PATHWAY_hsa04917	Prolactin signaling pathway	71	9	5.71E-04
KEGG_PATHWAY_hsa05231	Choline metabolism in cancer	101	10	0.0014574
KEGG_PATHWAY_hsa04210	Apoptosis	62	7	0.00602
KEGG_PATHWAY_hsa04350	TGF-beta signaling pathway	84	8	0.0070225
KEGG_PATHWAY_hsa04611	Platelet activation	130	10	0.0079302
**KEGG_PATHWAY_hsa04115**	**p53 signaling pathway**	**67**	**7**	**0.0087581**
KEGG_PATHWAY_hsa04062	Chemokine signaling pathway	186	12	0.0111322
KEGG_PATHWAY_hsa04010	MAPK signaling pathway	255	14	0.0190157
BIOCARTA_h_ctcfPathway	CTCF: First Multivalent Nuclear Factor	25	10	5.62E-06
BIOCARTA_h_her2Pathway	Role of ERBB2 in Signal Transduction and Oncology:	23	9	2.61E-05
BIOCARTA_h_pdgfPathway	PDGF Signaling Pathway:	28	9	1.29E-04
BIOCARTA_h_il7Pathway	IL-7 Signal Transduction:	17	7	2.65E-04
BIOCARTA_h_tpoPathway	TPO Signaling Pathway:	24	8	3.05E-04
BIOCARTA_h_arfPathway	Tumor Suppressor Arf Inhibits Ribosomal Biogenesis:	18	7	3.79E-04
BIOCARTA_h_egfPathway	EGF Signaling Pathway:	27	8	6.75E-04
BIOCARTA_h_vegfPathway	VEGF, Hypoxia, and Angiogenesis:	31	8	0.0016445
BIOCARTA_h_erk5Pathway	Role of Erk5 in Neuronal Survival:	18	6	0.0030032
BIOCARTA_h_raccycdPathway	Influence of Ras and Rho proteins on G1 to S Transition:	27	7	0.0039089
BIOCARTA_h_atrbrcaPathway	Role of BRCA1, BRCA2 and ATR in Cancer Susceptibility:	22	6	0.0076413
BIOCARTA_h_rasPathway	Ras Signaling Pathway:	23	6	0.0093148
BIOCARTA_h_hSWI-SNFpathway	Chromatin Remodeling by hSWI/SNF ATP-dependent Complexes:	17	5	0.0150159
BIOCARTA_h_p53Pathway	p53 Signaling Pathway:	17	5	0.0150159
BIOCARTA_h_telPathway	Telomeres, Telomerase, Cellular Aging, and Immortality:	18	5	0.0184481
BIOCARTA_h_ghPathway	Growth Hormone Signaling Pathway:	27	6	0.0185223
BIOCARTA_h_il2rbPathway	IL-2 Receptor Beta Chain in T cell Activation:	39	7	0.024242
BIOCARTA_h_erkPathway	Erk1/Erk2 Mapk Signaling pathway:	30	6	0.0284197
BIOCARTA_h_atmPathway	ATM Signaling Pathway:	21	5	0.0314984
REACTOME_PATHWAY_R-HSA-186763:R-HSA-186763	Downstream signal transduction	29	9	1.57E-07
REACTOME_PATHWAY_R-HSA-2122947:R-HSA-2122947	NOTCH1 Intracellular Domain Regulates Transcription	**47**	**9**	**8.12E-06**
REACTOME_PATHWAY_R-HSA-2644606:R-HSA-2644606	Constitutive Signaling by NOTCH1 PEST Domain Mutants	**57**	**9**	**3.52E-05**
REACTOME_PATHWAY_R-HSA-2894862:R-HSA-2894862	Constitutive Signaling by NOTCH1 HD+PEST Domain Mutants	**57**	**9**	**3.52E-05**
REACTOME_PATHWAY_R-HSA-1257604:R-HSA-1257604	PIP3 activates AKT signaling	81	10	7.61E-05
REACTOME_PATHWAY_R-HSA-5617472:R-HSA-5617472	Activation of anterior HOX genes in hindbrain development during early embryogenesis	122	12	8.58E-05
REACTOME_PATHWAY_R-HSA-3769402:R-HSA-3769402	Deactivation of the beta-catenin transactivating complex	42	7	3.12E-04
REACTOME_PATHWAY_R-HSA-1963642:R-HSA-1963642	PI3K events in ERBB2 signaling	16	5	3.58E-04
REACTOME_PATHWAY_R-HSA-1250196:R-HSA-1250196	SHC1 events in ERBB2 signaling	19	5	7.24E-04
REACTOME_PATHWAY_R-HSA-198203:R-HSA-198203	PI3K/AKT activation	9	4	8.39E-04
REACTOME_PATHWAY_R-HSA-5218921:R-HSA-5218921	VEGFR2 mediated cell proliferation	21	5	0.001079
REACTOME_PATHWAY_R-HSA-5673001:R-HSA-5673001	RAF/MAP kinase cascade	116	10	0.0011349
REACTOME_PATHWAY_R-HSA-1433557:R-HSA-1433557	Signaling by SCF-KIT	37	6	0.0012979
REACTOME_PATHWAY_R-HSA-2173795:R-HSA-2173795	Downregulation of SMAD2/3:SMAD4 transcriptional activity	23	5	0.0015415
REACTOME_PATHWAY_R-HSA-5693579:R-HSA-5693579	Homologous DNA Pairing and Strand Exchange	25	5	0.0021266
REACTOME_PATHWAY_R-HSA-1358803:R-HSA-1358803	Downregulation of ERBB2:ERBB3 signaling	13	4	0.0026727
REACTOME_PATHWAY_R-HSA-3214841:R-HSA-3214841	PKMTs methylate histone lysines	64	7	0.0029663
REACTOME_PATHWAY_R-HSA-452723:R-HSA-452723	Transcriptional regulation of pluripotent stem cells	28	5	0.0032666
REACTOME_PATHWAY_R-HSA-69202:R-HSA-69202	Cyclin E associated events during G1/S transition	14	4	0.003346
REACTOME_PATHWAY_R-HSA-201722:R-HSA-201722	Formation of the beta-catenin:TCF transactivating complex	88	8	0.0034726
**REACTOME_PATHWAY_R-HSA-1912408:R-HSA-1912408**	**Pre-NOTCH Transcription and Translation**	**29**	**5**	**0.0037238**
REACTOME_PATHWAY_R-HSA-1306955:R-HSA-1306955	GRB7 events in ERBB2 signaling	5	3	0.0047627
REACTOME_PATHWAY_R-HSA-2559584:R-HSA-2559584	Formation of Senescence-Associated Heterochromatin Foci (SAHF)	16	4	0.0049808
REACTOME_PATHWAY_R-HSA-1963640:R-HSA-1963640	GRB2 events in ERBB2 signaling	16	4	0.0049808
REACTOME_PATHWAY_R-HSA-1234158:R-HSA-1234158	Regulation of gene expression by Hypoxia-inducible Factor	10	3	0.0199089
REACTOME_PATHWAY_R-HSA-167044:R-HSA-167044	Signalling to RAS	10	3	0.0199089
REACTOME_PATHWAY_R-HSA-1502540:R-HSA-1502540	Signaling by Activin	11	3	0.023979
REACTOME_PATHWAY_R-HSA-196025:R-HSA-196025	Formation of annular gap junctions	11	3	0.023979
REACTOME_PATHWAY_R-HSA-190873:R-HSA-190873	Gap junction degradation	12	3	0.0283567
REACTOME_PATHWAY_R-HSA-3108214:R-HSA-3108214	SUMOylation of DNA damage response and repair proteins	77	6	0.0287672
REACTOME_PATHWAY_R-HSA-75955:R-HSA-75955	RNA Polymerase II Transcription Elongation	54	5	0.0322269
REACTOME_PATHWAY_R-HSA-171007:R-HSA-171007	p38MAPK events	13	3	0.0330265
REACTOME_PATHWAY_R-HSA-198323:R-HSA-198323	AKT phosphorylates targets in the cytosol	13	3	0.0330265
REACTOME_PATHWAY_R-HSA-350054:R-HSA-350054	Notch-HLH transcription pathway	13	3	0.0330265
REACTOME_PATHWAY_R-HSA-5467337:R-HSA-5467337	APC truncation mutants have impaired AXIN binding	14	3	0.0379733
REACTOME_PATHWAY_R-HSA-5467348:R-HSA-5467348	Truncations of AMER1 destabilize the destruction complex	14	3	0.0379733
REACTOME_PATHWAY_R-HSA-5467340:R-HSA-5467340	AXIN missense mutants destabilize the destruction complex	14	3	0.0379733
REACTOME_PATHWAY_R-HSA-1250347:R-HSA-1250347	SHC1 events in ERBB4 signaling	14	3	0.0379733
REACTOME_PATHWAY_R-HSA-69473:R-HSA-69473	G2/M DNA damage checkpoint	84	6	0.0397302

Supplementary Table S4. Genetic alterations affecting homologous recombination genes in metaplastic breast carcinomas and uterine carcinosarcomas.

Sample name	Gene	Mutation	Effect	Chromo some	Position	Reference allele	Alternate allele	Sequencing depth at position	Loss of heterozygosity	Clinvar	Mutation status	Somatic homozygous deletion
META33	BRCA1	Q74*	Nonsense mutation	17	41256966	G	A	57	TRUE	Pathogenic	Germline	-
META39	BRCA2	Y3225fs	Frame_Shift_Ins	13	32972321	T	TA	156	-	Pathogenic	Germline	-
META39	RBBP8	R100W	Missense mutation	18	20548818	C	T	67	TRUE	Pathogenic	Germline	-
META52	RAD54B	S240A	Missense mutation	8	95419730	A	C	135	-	-	Somatic	-
MP1	BRCA2	S1982fs	Frame_Shift_Del	13	32914437	GT	G	173	-	Pathogenic	Germline	-
MP1	BRCA2	E2226Sfs*6	Frame_Shift_Del	13	32915166	CAG	C	507	-	Pathogenic	Somatic	-
MP11	EME1	S411T	Missense_Mutation	17	48456548	G	C	769	TRUE	-	Somatic	-
MP11	BRCA1	G1777fs	Frame_Shift_Ins	17	41209079	T	TG	169	TRUE	Pathogenic	Germline	-
MP15	BRIP1	A349P	Missense mutation	17	59878709	C	G	183	-	Pathogenic	Germline	-
MP19	BRCA1	L639*	Nonsense mutation	17	41245632	A	T	126	TRUE	Pathogenic	Germline	-
MP21	BRCA1	G1800Fs	Frame_Shift_Del	17	41201208	TG	T	84	TRUE	Pathogenic	Germline	-
MP27	SLX4	A1358G	Missense mutation	16	3639566	G	C	212	-	-	Somatic	-
MP27	POLQ	T948fs	Frame_Shift_Del	3	121208934	AT	A	68	-	-	Germline	-
MP8	BRCA1	Q23fs	Frame_Shift_Del	17	41276044	ACT	A	71	TRUE	Pathogenic	Germline	-
MTC04	EME1	N233K	Missense mutation	17	48453268	T	A	713	TRUE	-	Somatic	-
MTC06	BRCA1	E1346Fs	Frame_Shift_Del	17	41243512	CT	C	111	TRUE	Pathogenic	Germline	-
MTC14	BRIP1	D674G	Missense mutation	17	59853838	T	C	386	TRUE	-	Somatic	-
MP15	BRCA2	-	-	13	-	-	-	-	-	-	-	13q13.1
UCS11	WRN	-	-	8	-	-	-	-	-	-	-	8p12
UCS11	USP11	-	-	X	-	-	-	-	-	-	-	Xp11.23
UCS12	FANCA	-	-	16	-	-	-	-	-	-	-	16q24.3

Supplementary Table S5. Number of shared and unique mutations, mutational signatures and clonal relatedness index of the histologically distinct components of 11 metaplastic breast cancers and 6 uterine carcinosarcomas subjected to whole-exome sequencing.

Case ID	Shared non-synonymous somatic mutations carcinoma and mesenchymal components (n)	Shared non-synonymous somatic mutations between mesenchymal components (n)	Carcinoma unique non-synonymous somatic mutations (n)	Mesenchymal unique non-synonymous somatic mutations (n)	Mutational signature (dominant/secondary)	SigMA (dominant/secondary)	Clonal relatedness index
MP1-1	131	N/A	14	18	NP	Aging/APOBEC	66
MP1-2					NP	APOBEC/HRD	66
MP7-1	54	N/A	18	9	NP	Aging/HRD	48
MP7-2					NP	Aging/HRD	48
MP8-1	78	N/A	42	44	3 (HRD)/Sig. 22	HRD/Aging	66
MP8-2					3 (HRD)/13 (APOBEC)	HRD/Aging	66
MP11-A1	62	54	N/A	17	NP	HRD/Aging	66
MP11-A2				41	3 (HRD)/13 (APOBEC)	HRD/Aging	66
MP11-B			185	N/A	3 (HRD)/26 (MSI)	HRD/Aging	66
MP15-1	87	N/A	46	16	NP	HRD/Aging	66
MP15-2					3 (HRD)/7 (UV)	HRD/Aging	66
MP17-A1	17	20	N/A	4	NP	NP	19/25
MP17-A2				3	NP	NP	19/25
MP17-B1			2	N/A	NP	NP	19/25
MP18-1	29	N/A	35	62	3 (HRD)/1 (Aging)	HRD/Aging	66
MP18-2					3 (HRD)/13 (APOBEC)	HRD/Aging	66
MP19-1	109	N/A	17	38	NP	HRD/Aging	66
MP19-2					3 (HRD)/Sig. 24	HRD/Aging	66
MP21-R1	36	18	N/A	31	3 (HRD)/Sig. 11	HRD/Aging	66
MP21-R2				2	NP	NP	66
MP21-2			4	N/A	NP	NP	66
MP27-1	54	N/A	47	48	3 (HRD)/1 (Aging)	HRD/Aging	66
MP27-3					1 (Aging)/3 (HRD)	HRD/Aging	66
MP35-1	48	N/A	19	18	NP	Aging/HRD	33
MP35-2					NP	Aging/HRD	33
CS1-C	81	N/A	1047	865	6 (MSI)/15 (MSI)	MSI/Aging	57
CS1-S					6 (MSI)/15 (MSI)	MSI/Aging	57
CS3-C	67	N/A	3	6	NP	NP	47
CS3-S					NP	Aging/HRD	47
CS4-C	51	N/A	19	41	1 (Aging)/7 (UV)	Aging	30
CS4-S					3 (HRD)/1 (Aging)	Aging	30
CS5-C	50	N/A	4	1	NP	NP	30
CS5-S					NP	NP	30
CS6-C	25	N/A	19	17	NP	APOBEC/HRD	18
CS6-S					NP	Aging/HRD	18
CS8-C	67	N/A	21	20	3 (HRD)/1 (Aging)	APOBEC/HRD	57
CS8-S					8 (HRD)/Sig.29	Aging/HRD	57

N/A, not applicable; NP, not performed (mutational signatures using DeconstructSigs could only be defined in samples ≥20 single nucleotide variants (SNVs); SigMA signatures could only be defined for samples ≥5 SNVs).
